# Implementation and Results of Active Vaccine Safety Monitoring During the COVID-19 Pandemic in the UK: A Regulatory Perspective

**DOI:** 10.1007/s40264-025-01579-w

**Published:** 2025-09-03

**Authors:** Jenny Wong, Katherine Donegan, Kendal Harrison, Tahira Jan, Alison Cave, Phil Tregunno

**Affiliations:** Medicines and Healthcare products Regulatory Agency (MHRA), Safety and Surveillance, 10 South Colonnade, Canary Wharf, London, E14 4PU UK

## Abstract

**Introduction:**

Yellow Card Vaccine Monitor (YCVM) was established by the UK Medicines and Healthcare products Regulatory Agency (MHRA) to facilitate active monitoring of adverse drug reactions following COVID-19 vaccination and further characterise safety in populations under-represented in clinical trials.

**Objective:**

This study explored the profile of individuals registered to the YCVM platform and the suspected adverse drug reactions reported following a COVID-19 vaccination on this data platform.

**Methods:**

Using a stratified random selection approach, individuals were invited to register and actively contacted to seek further information on the vaccines received and adverse reactions they experienced. Exploratory analyses were conducted to characterise the demographics of individuals registered in the YCVM, and to summarise the adverse drug reaction data reported by recruited individuals between November 2020 and December 2022. Detailed analyses of the sub-cohort of pregnant and breastfeeding females were conducted to characterise these individuals. Data for two suspected adverse reactions, menstrual disorders and tinnitus, were extracted and analysed to demonstrate how YCVM supported regulatory assessment of these safety signals which originally arose from other data sources.

**Results:**

36,604 individuals registered, with 30,281 reporting vaccination. Median (interquartile range) follow-up was 184 days (14–367). Demographics of the recruited cohort reflected the vaccinated population and timing of invitations. 15,764 (52.1%) of those reporting vaccination reported experiencing at least one adverse reaction. However, nearly all were expected acute reactions and 4134 (13.7%) reported an event considered medically serious. The data raised no safety concerns in pregnant and breastfeeding females. Reporting of menstrual disorders appeared stimulated by media interest, as seen in spontaneous reporting systems. Data on the incidence of tinnitus were used to support regulatory action on this signal.

**Conclusion:**

Active surveillance using the YCVM provided a complementary data source for monitoring the safety of COVID-19 vaccines. However, further efforts are needed to recruit ethnic minorities. The technology developed has enhanced regulatory vigilance options and could be valuable in the future for actively monitoring the safety of innovative products used in small populations.

**Supplementary Information:**

The online version contains supplementary material available at 10.1007/s40264-025-01579-w.

## Key Points

**Table:** 

Active surveillance via a newly developed tool, the Yellow Card Vaccine Monitor, was successfully implemented in the UK as one of four pillars of the UK Medicines and Healthcare products Regulatory Agency (MHRA) COVID-19 Vaccine Safety Surveillance Strategy.
The data supported a favourable safety profile for the COVID-19 vaccines including in pregnant and breastfeeding individuals and were used in the regulatory assessment of safety signals.
The ability to actively recruit and follow up individuals in order to seek adverse reaction reporting data increases regulatory vigilance capabilities for the future.

## Introduction

Upon the introduction of any new vaccination programme, a large and diverse patient population receives the vaccine over a very short period. While clinical trials provide data on adverse events, particularly common and acute reactions in the trial population, there remains a clear need to continuously monitor safety and rapidly increase the evidence base on the benefits and risks [1]. This is in order to identify rare adverse events, which trials are underpowered to detect, as well as to further explore the overall safety profile of the vaccine in wider routine use, including in groups excluded from clinical trials.

Post-marketing safety surveillance of medicines and vaccines typically include passive and active approaches to monitor for suspected adverse reactions. Active surveillance using cohort event monitoring (CEM) had been increasingly used globally during the COVID-19 vaccination programmes [2–12]. This method aims to collect self-reported adverse reaction from vaccinated individuals who are enrolled at the point of their vaccination in a real-world setting. The cohort of individuals are then followed up over a defined period, with follow-up conducted at set time intervals post-vaccination. Active surveillance systems are utilised to complement passive spontaneous reporting of suspected adverse reactions [13].

The Yellow Card Vaccine Monitor (YCVM) was set up by the UK Medicines and Healthcare products Regulatory Agency (MHRA) to actively collect suspected adverse vaccine reactions reported in association with a COVID-19 vaccine within targeted populations in a real-world setting [14]. The YCVM was implemented by the MHRA under their statutory responsibility to operate a national pharmacovigilance system and is classed as surveillance.

This platform was one of the four complementary strands of the proactive vigilance strategy of the MHRA for COVID-19 vaccines [15]. It was designed to complement the existing Yellow Card scheme, through which healthcare providers, individuals and their carers can report incidences of suspected side effects of medicines, including vaccines. Established spontaneous reporting systems are critical in the detection of rare risks; however, they are limited in particular by under-reporting, meaning the data cannot be reliably used to estimate the frequency of an adverse reaction and, because of the challenges in obtaining follow-up, spontaneously reported data generally capture a snapshot in time, with ongoing longitudinal information and patient outcomes not routinely available. This can limit the use of the data in monitoring vaccine safety [16].

The primary purpose of the YCVM was not to detect safety signals or identify rare risks, although, if signals of potentially more common adverse reactions arose from other sources, it could be used as supportive evidence. Rather it was designed to compare the frequency and severity of adverse reactions seen in routine use to those seen in clinical trials to allow further characterisation of the safety profile, particularly in subgroups under-represented in clinical trials.

In response to potential signals identified by the MHRA through the data amassed through the different strands of the COVID-19 surveillance strategy, or from elsewhere, safety reviews were conducted to evaluate evidence from multiple sources such as clinical trial data, spontaneous adverse drug reaction (ADR) data, and epidemiological data from literature.

The YCVM was established prior to deployment of the first vaccine outside of a clinical trial which occurred on 8 December 2020. In England, the COVID-19 vaccination programme began by offering vaccinations to priority groups including those in residential care, frontline health and social care workers, older individuals, and those considered clinically vulnerable, before vaccinations were offered to younger individuals, and subsequently to those under 18 years old and pregnant females [17–20]. Those with severe immunosuppression, who may not have mounted a full immune response, were offered a third dose after their primary course (two doses) with other individuals offered a first booster [21]. Later doses were offered according to eligibility criteria recommended by the Joint Committee on Vaccination and Immunisation (JCVI).

In this paper, we present the data profile of the individuals registered to the YCVM along with the reports of suspected adverse reactions following a COVID-19 vaccination that they submitted. To further explore the value of the YCVM, we demonstrate how the data from YCVM contributed to safety evaluations using three case studies. This included two safety signals, menstrual disorders and tinnitus, which first arose from other data sources, and which were evaluated as part of the continuous benefit-risk assessment process of the MHRA during the pandemic, as well as the specific surveillance of pregnant females.

We discuss the learnings from the experience with implementing active surveillance using this technology in a regulatory setting and the proposals and opportunities for future development and use.

## Methods

### Description of Yellow Card Vaccine Monitor Platform

In England, a central National Health Service (NHS) system identified individuals who were eligible for the COVID-19 vaccine and invited them by letter to book their COVID-19 vaccination. Through this call/recall system, using a stratified random sampling approach, selected individuals were additionally invited to voluntarily register for follow-up via the YCVM digital platform. The groups of individuals sent invitations by the NHS to register with the YCVM aligned with the call-in priorities for vaccination during the vaccination programme, taking into account the distribution of individuals registered by region and ethnicity. The timing of invitations was largely determined by the deployment of the vaccine programme to new groups of individuals, and the number of invitations for each cohort was adjusted according to the registration response rates. In addition to random invites, pregnant females were also encouraged to register with the YCVM through printed information provided to them when they were considering, or having, their vaccination [22].

Enrolment was sought prior to vaccination and individuals, or their carers, were required to read and agree to the User Sign-up Agreement and the privacy policy, in order to participate. To support accessibility, a telephone service and an easy-read invitation letter were also offered.

At enrolment, information on individuals, including demographics, NHS number, and pregnancy and breastfeeding information, were collected, where relevant. Information on past medical history, receipt of a seasonal flu vaccine and COVID-19 infection status were also sought.

Once registered, individuals were contacted via email or SMS at set intervals to ask for further information, including about the vaccinations received and whether they had experienced any suspected ADRs. Follow-up was sought at 7 days and 14 days post vaccination, with longer-term follow-up sought at 2 months, 3 months, 6 months, 9 months and 1 year post vaccination. Additional follow-up was sought from pregnant women 10 weeks after the expected due date of their baby. They were also provided the opportunity to spontaneously update or add to their previously submitted information, including outcomes for any previously reported reactions. When new information was submitted by the individual, a new submission was recorded. The seriousness of an ADR could be reported by selecting from the optional seriousness categories. Where a suspected ADR was recorded, the data were entered onto the MHRA’s ADR reporting database and evaluated alongside all other data in safety surveillance activities as outlined in the COVID-19 vigilance strategy. The adverse reactions are recorded using the Medical Dictionary for Regulatory Activities (MedDRA), a clinically validated international medical terminology used for medicines regulation in the UK. MedDRA groups related ADR terms in a hierarchical structure with five levels, whereby the highest level 'System Organ Class' (SOC) groups together reactions that affect similar system/organ in the body and the ‘Lowest Level Term' (LLT) is the most specific term [23]. The ADRs reported in the YCVM were classified at the LLT level. The YCVM data specification met ICH E2B guidelines on the electronic transmission of individual case reports [24].

### Statistical Analysis

Data submitted in the YCVM up to 31 December 2022, with data locked at this point, were extracted. Only data reported directly through the YCVM were reported in this study, with any additional follow-up of individual cases undertaken separately excluded.

Descriptive analyses were conducted to evaluate the extent and timing of registrations with the YCVM and all data submissions and the duration of follow-up. In these analyses, where there were multiple submissions by an individual on a single day, only one submission per individual was counted. The follow-up time contributed per individual was derived from the later of the individual’s registration date or first reported vaccination date and the date of their last submission of information on the YCVM.

Characteristics were summarised for all registered individuals, individuals who reported vaccination data (further stratified by vaccine brand and dose), and individuals who also reported an ADR following a vaccination. To note, information on the sex of the individual, i.e. male/female, was requested within the YCVM as opposed to gender. Individuals were classified as having immunosuppression if they indicated that they had any of a prespecified list of medical conditions or were taking specific medications that might lower their immune response.

To explore those actively submitting ADR information to the YCVM over time, analyses were conducted to characterise the cohorts who had reported a first dose, those who reported a first and second dose, and also those who reported first, second and third booster doses. Amongst these cohorts, the proportion reporting an ADR or a MedDRA serious ADR was calculated and stratified by demographics of individuals. The dose sequence in the analyses was derived by the vaccination date reported by the individual or their carer. The YCVM makes no distinction between first booster and third doses in the data collection stage, therefore third dose in the analyses refers to the dose taken after their primary course (first and second dose). The analysis of the ADRs reported in the YCVM was conducted at the MedDRA Preferred Term (PT) level. The number of ADRs reported and the most common ADR events reported for each vaccine and each dose were extracted. These most commonly reported ADR events were explored using the corresponding MedDRA PT and SOC terms.

Reporters were able to indicate the seriousness of their ADRs against at least one of a number of non-mandatory options describing their impact. The most serious indicator was then assigned to the individual’s whole report regardless of which dose an ADR occurred in temporal association with. Note the categories for the level of seriousness (including added terms: mild or lightly uncomfortable; uncomfortable, a nuisance or irritation, but able to carry on with everyday activities; and had short-term effect that was bad enough to affect everyday activities) were adapted from CIOMS guidance to allow individuals to better describe events that were non-serious [25]. In addition to reporter seriousness, an ADR report could be considered serious if the reported reaction was serious according to the MedDRA medical dictionary. Seriousness of a reaction term in the MedDRA dictionary is assigned by the MHRA through agreement by an internal panel of medical assessors. The number of individuals reporting ADRs identified as MedDRA serious events and the number of these ADRs were analysed.

A subgroup analysis was conducted on females who reported being pregnant or breastfeeding at the time of vaccination or during their follow-up time in the YCVM. The proportion reporting an ADR after a COVID-19 vaccination dose was calculated and stratified by their demographics. In the pregnancy analysis, the reported stage of their pregnancy at the time of vaccination entered by a pregnant female is presented. These categories include first trimester (1–12 weeks), second trimester (13–28 weeks), and third trimester (29–40 weeks). When a pregnant female reports vaccination in more than one trimester, they are assigned into a category indicating more than one stage was reported. If no stage of pregnancy was reported, they were categorised as unknown.

The YCVM was used alongside other sources of data to support the assessment of emerging safety signals. To demonstrate how the YCVM was used as part of ongoing safety monitoring, examples of analyses conducted for two case studies were included in this study. For the two case studies, the numbers of individuals reporting an ADR relating to menstrual disorders (female individuals) or tinnitus were extracted. The MedDRA event search terms used to identify cases of menstrual disorders and tinnitus are given in Online Supplemental Material (OSM) Resource 1. The onset time for tinnitus was derived by subtracting the date of the ADR event from the date of the most recent vaccination. The outcomes of ADRs are based on the outcome as reported at the time of the individual’s last submission. The case study analyses in this paper are not a full safety assessment of these safety signals.

## Results

### Registered Individuals

**Fig. 1:**
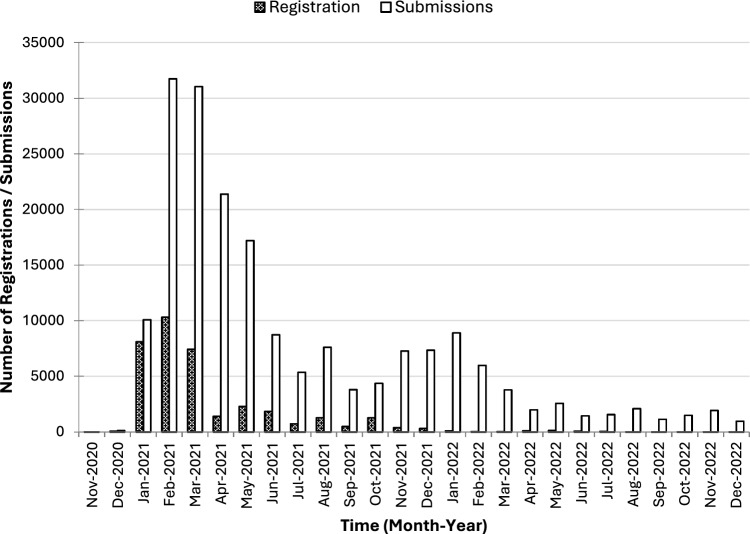
Number of registrations and submissions to the Yellow Card Vaccine Monitor up to 31 December 2022

Over 1.4 million invitations were sent out during the study period. A total of 36,604 individuals (2.5%) registered with the YCVM, with the first individual registering in November 2020 and the last registration within the study period in December 2022. The number of registrations and submissions by calendar month and year are shown in Fig. 1. The time period for the majority of registrations aligns with the period in which first doses were initially offered in the UK, while peaks in later submissions align with the offer of second and subsequent doses. A total of 190,034 submissions were submitted by the 36,604 registered individuals during the study period. The median number of submissions submitted was 3 (interquartile range (IQR): 2–7).

Table 1 presents the demographics of individuals registered in the YCVM and the subset of those who reported a vaccination during their follow-up time in the YCVM. The median age of the cohort reported at registration was 62 years (IQR: 42–73). 10,791 (35.6%) of those reporting a vaccination had a registration date before their first vaccination, while 14,369 (47.5%) registered after the date of their first vaccination. Despite vaccination only being recommended in individuals aged 5+ years in the UK within the study period, 63 individuals reported an age < 5 years. The median age amongst those who had reported a vaccination was 63 years (IQR: 43–74). Other demographics of those reporting a vaccination were also broadly the same as those registering. The median follow-up in those reporting a vaccination was 184 patient days (IQR: 14–367, range 1–697), and 82.1% of the total follow-up time was from those aged 50 years or older. Female individuals contributed slightly more to YCVM than males, approximately 55% of the total follow-up time. Females were predominant contributors amongst the younger age groups up until the 70- to 79-year age group, with particularly greater levels of follow-up in the 50- to 59-year and 30- to 39-year age groups compared to men (median 273 vs. 264 patient days and 60 vs. 52 patient days, respectively). In the older age groups, men contributed greater levels of follow-up than females (median 181 days vs. 93 patient days amongst the ≥ 80-year age group, and 324 vs. 286 patient days in the 70- to 79-year age group). The follow-up time stratified by the sex of the individuals is presented in OSM Resource 2 to support contextualisation of the analyses on vaccinations in pregnancy.

**Table 1:** Number of individuals registered in the Yellow Card Vaccine Monitor (YCVM) stratified by vaccination reporting status and the total follow-up time accumulated in vaccinated individuals

	Number of individuals, *n* (%)				Follow-up time (days)		
	All registered individuals		Individuals reporting vaccination data		Total	Median	(IQR)
Number of registered individuals	36 604		30 281		6 808 705	184	(14–367)
Sex							
Male	15 223	(41.6)	12 424	(41.0)	3 040 806	249	(16–383)
Female	20 868	(57.0)	17 463	(57.7)	3 701 682	179	(10–363)
Unknown	513	(1.4)	394	(1.3)	66 217	74	(1–333)
Age bands (years)							
Under 12	195	(0.5)	120	(0.4)	11 489	18	(1–183)
12–17	2109	(5.8)	1238	(4.1)	107 662	17	(2–127)
18–29	1747	(4.8)	1399	(4.6)	141 719	15	(1–184)
30–39	4133	(11.3)	3613	(11.9)	489 190	59	(3–244)
40–49	3286	(9.0)	2713	(9.0)	465 917	79	(8–285)
50–59	4926	(13.5)	3936	(13.0)	941 784	270	(17–365)
60–69	7118	(19.4)	6029	(19.9)	1 738 576	296	(57–450)
70–79	10 464	(28.6)	8899	(29.4)	2 435 668	304	(53–407)
80+	2596	(7.1)	2309	(7.6)	471 414	123	(8–365)
Unknown	30	(0.1)	25	(0.1)	5286	91	(1–380)
Ethnicity							
White British, White Irish, or any other White background	31 331	(85.6)	26 281	(86.8)	6 208 814	213	(15–372)
Other	2392	(6.5)	1759	(5.8)	262 287	57	(4–273)
Unknown	2881	(7.9)	2241	(7.4)	337 604	72	(2–276)
BMI category							
Underweight	842	(2.3)	667	(2.2)	146 265	183	(8–369)
Normal	10 471	(28.6)	8928	(29.5)	2 189 172	245	(16–381)
Overweight	9727	(26.6)	8184	(27.0)	2 008 210	248	(17–383)
Obese	5362	(14.6)	4436	(14.6)	1 005 914	184	(15–367)
Unknown	10 202	(27.9)	8066	(26.6)	1 459 144	88	(8–333)
Immunocompromised							
Yes	4162	(11.4)	3483	(11.5)	811 593	195	(16–368)
No/unknown	32 442	(88.6)	26 798	(88.5)	5 997 112	183	(13–367)
Reported as pregnant at the time of a vaccination							
Yes	2517	(6.9)	2416	(8.0)	330 018	73	(5–244)
No/unknown	34 087	(93.1)	27 865	(92.0)	6 478 687	189	(15–370)

*BMI* body mass index, *IQR* interquartile range

### COVID-19 Vaccinations Reported

The demographics of individuals stratified by the vaccination brand(s) they reported receiving across all doses (*N* = 56,371) are presented in Table 2. An individual could receive doses from different product brands during their COVID-19 vaccination regimen, therefore an individual could appear in more than one column. Data are presented for the Pfizer, AstraZeneca, and Moderna monovalent vaccines. Of those reporting receiving another vaccine brand, two individuals reported receiving a Moderna bivalent vaccine, ten a Novavax vaccine, and two a Janssen vaccine.

**Table 2:** Demographics of individuals stratified by vaccine brand administered (percentage of doses reported for the vaccine brand, %)

	Pfizer		AstraZeneca		Moderna		Unknown or other brand	
Number of individuals reporting a vaccine dose	16 509		17 713		4467		1125	
Number of doses (% of total doses)	24 405	(43.3)	25 515	(45.3)	5228	(9.3)	1223	(2.2)
Sex								
Male	6614	(40.1)	7738	(43.7)	1944	(43.5)	504	(44.8)
Female	9687	(58.7)	9763	(55.1)	2483	(55.6)	594	(52.8)
Unknown	208	(1.3)	212	(1.2)	40	(0.9)	27	(2.4)
Age bands (years)								
Under 12	90	(0.5)	22	(0.1)	12	(0.3)	11	(1.0)
12–17	1207	(7.3)	4	(0.0)	18	(0.4)	40	(3.6)
18–29	1023	(6.2)	286	(1.6)	199	(4.5)	22	(2.0)
30–39	2630	(15.9)	801	(4.5)	552	(12.4)	25	(2.2)
40–49	958	(5.8)	1960	(11.1)	394	(8.8)	37	(3.3)
50–59	1280	(7.8)	3476	(19.6)	592	(13.3)	103	(9.2)
60–69	2748	(16.6)	4732	(26.7)	1023	(22.9)	214	(19.0)
70–79	5156	(31.2)	5372	(30.3)	1451	(32.5)	512	(45.5)
80+	1405	(8.5)	1043	(5.9)	223	(5.0)	160	(14.2)
Unknown	12	(0.1)	17	(0.1)	3	(0.1)	1	(0.1)
Ethnicity								
White British, White Irish, or any other White background	14 218	(86.1)	15 833	(89.4)	4039	(90.4)	1016	(90.3)
Other	1111	(6.7)	720	(4.1)	183	(4.1)	28	(2.5)
Unknown	1180	(7.1)	1160	(6.5)	245	(5.5)	81	(7.2)
BMI category								
Underweight	423	(2.6)	323	(1.8)	84	(1.9)	33	(2.9)
Normal	5041	(30.5)	5229	(29.5)	1556	(34.8)	289	(25.7)
Overweight	4256	(25.8)	5205	(29.4)	1277	(28.6)	342	(30.4)
Obese	2222	(13.5)	2839	(16.0)	613	(13.7)	153	(13.6)
Unknown	4567	(27.7)	4117	(23.2)	937	(21.0)	308	(27.4)
Immunocompromised								
Yes	1916	(11.6)	2096	(11.8)	519	(11.6)	166	(14.8)
No/unknown	14 593	(88.4)	15 617	(88.2)	3948	(88.4)	959	(85.2)

*BMI* body mass index

Table 3 presents a summary of the number and demographics of individuals reporting a vaccination stratified by dose number and vaccine brand. In addition, three individuals reported a sixth dose. The median age of individuals in the subgroup reporting for fourth or fifth doses was older compared to those reporting primary vaccination doses. This reflects the narrowing eligibility criteria for booster doses. The higher median age of individuals reporting a first or second dose of the AstraZeneca vaccine reflects the advised preferential use of alternatives in individuals aged < 40 years following the signal of thrombosis with thrombocytopenia adverse reactions. Amongst those who reported receiving a first dose, 11.5% (*n* = 3146) were identified as immunocompromised. The proportion of immunocompromised individuals increased across the doses, with 11.5% (*n* = 1691) in second-dose, 12.2% (*n* = 1217) in third-dose, 15.5% (*n* = 527) in fourth-dose, and 25.1% (*n* = 242) in fifth-dose recipients. Again, this reflects the narrowing eligibility criteria for booster doses. The proportion of reported vaccines marked as unknown or ‘other’ brand increased slightly with dose number.

**Table 3:** The demographics of vaccinated individuals stratified by vaccine dose number and vaccine brand

	Number of individuals (% of those receiving dose)		Median age, years (IQR)		Number of females (%)		Number of immuno-compromised (%)	
Dose 1	27 403	(100)	63	(43–74)	15 895	(100)	3146	(100)
Pfizer	10 249	(37.4)	54	(31–75)	6300	(39.6)	1152	(36.6)
AstraZeneca	16 228	(59.2)	65	(55–73)	8988	(56.5)	1910	(60.7)
Moderna	550	(2.0)	34	(30–40)	402	(2.5)	24	(0.8)
Unknown/other	376	(1.3)	73	(57–77)	205	(1.3)	60	(1.9)
Dose 2	14 657	(100)	65	(50–74)	8344	(100)	1691	(100)
Pfizer	5023	(34.4)	66	(34–75)	3072	(36.8)	586	(34.7)
AstraZeneca	9105	(62.1)	65	(57–73)	4923	(59.0)	1060	(62.7)
Moderna	302	(2.1)	35.5	(31–42)	226	(2.7)	13	(0.8)
Unknown/other	227	(1.5)	73	(61–78)	123	(1.5)	32	(1.9)
Dose 3	9935	(100)	67	(56–74)	5389	(100)	1217	(100)
Pfizer	7505	(75.5)	69	(58–75)	4018	(74.6)	972	(79.9)
AstraZeneca	161	(1.6)	71	(62–75)	76	(1.4)	25	(2.1)
Moderna	1992	(20.1)	58	(46–65)	1157	(21.5)	176	(14.5)
Unknown/other	277	(2.8)	74	(66–77)	138	(2.6)	44	(3.6)
Dose 4	3409	(100)	72	(64–75)	1691	(100)	527	(100)
Pfizer	1386	(40.7)	68	(59–75)	728	(43.1)	245	(46.5)
AstraZeneca	18	(0.5)	73	(62–77)	8	(0.5)	3	(0.6)
Moderna	1753	(51.4)	73	(67–76)	830	(49.1)	244	(46.3)
Unknown/other	252	(7.4)	72	(63–75)	125	(7.4)	35	(6.6)
Dose 5	964	(100)	75	(74–78)	463	(100)	242	(100)
Pfizer	242	(25.1)	75	(74–78)	121	(26.1)	66	(27.3)
AstraZeneca	3	(0.3)	74	(73–78)	2	(0.4)	0	(0.0)
Moderna	628	(65.1)	75	(74–78)	296	(63.9)	160	(66.1)
Unknown/other	91	(9.4)	76	(74–78)	44	(9.5)	16	(6.6)

*IQR* interquartile range

Of the 30,281 individuals reporting a vaccine dose, 27,403 (90.5%) reported receiving a first dose, 12,508 (41.3%) reported receiving a first and second dose, and 7891 (26.1%) reported receiving a first, second and third dose. 2878 (9.5%) reported receiving a second or subsequent vaccine dose but did not report details for their first dose. Within the UK COVID-19 vaccine programme, it was recommended that an individual should receive the same vaccine product for their first and second doses (primary course). Of those reporting both primary course doses, 99% reported receiving homologous dose regimens (*n* = 12,070). Less than 1% of individuals reported receiving a heterogenous course (*n* = 148). Amongst the individuals who had reported a first dose and received a homogenous schedule, 63.4% (*n* = 7654) received the AstraZeneca vaccine whilst 34.8% (*n* = 4203) received the Pfizer vaccine and 1.8% (*n* = 213) the Moderna vaccine.

### Adverse Reactions Reported Following Vaccination

The demographics for those reporting an ADR are presented in Table 4. 15,764 individuals (52.1%) of those reporting a vaccine dose, reported experiencing at least one ADR following vaccination. The median age of those who reported an ADR was 59 years (IQR: 42–71) and amongst those who did not report an ADR was 68 years (IQR: 49–75). Female individuals more commonly reported ADRs, and the rate of reporting of ADRs decreased with increasing dose number. Demographics of individuals reporting an ADR for each dose stratified by vaccination brand are shown in the table in OSM Resource 3.

**Table 4:** Number of individuals reporting an adverse drug reaction (ADR) following a COVID-19 vaccination, stratified by demographics and vaccination dose number

	Individuals reporting an ADR (% of cohort reporting any vaccine dose)		Individuals reporting an ADR after the first dose (% of cohort reporting a first vaccine dose)		Individuals reporting an ADR after the second dose (% of cohort reporting first and second vaccine doses)		Individuals reporting an ADR after the third dose (% of cohort reporting first, second, and third vaccine doses)	
Sex								
Male	5407	(43.5)	4173	(37.4)	839	(16.0)	592	(17.1)
Female	10 145	(58.1)	8009	(50.4)	1764	(24.7)	1134	(26.1)
Unknown	212	(53.8)	145	(42.2)	22	(16.3)	16	(22.2)
Age bands (years)								
Under 12	45	(37.5)	36	(34.0)	4	(10.5)	1	(9.1)
12–17	592	(47.8)	519	(42.5)	81	(30.7)	15	(19.5)
18–29	829	(59.3)	669	(51.0)	141	(30.8)	64	(29.8)
30–39	1983	(54.9)	1567	(47.6)	359	(28.0)	133	(20.7)
40–49	1937	(71.4)	1668	(66.6)	275	(27.0)	201	(32.4)
50–59	2505	(63.6)	2041	(58.2)	379	(22.9)	367	(31.7)
60–69	3430	(56.9)	2639	(49.3)	618	(21.0)	523	(25.1)
70–79	3715	(41.7)	2666	(33.4)	666	(16.6)	391	(14.7)
80+	719	(31.1)	515	(24.5)	101	(12.3)	47	(11.2)
Unknown	9	(36.0)	7	(31.8)	1	(20.0)	0	(0.0)
Ethnicity								
White British, White Irish, or any other White background	13 705	(52.1)	10 770	(45.2)	2369	(21.1)	1574	(21.8)
Other	966	(54.9)	789	(48.3)	149	(26.5)	97	(30.8)
Unknown	1093	(48.8)	768	(39.1)	107	(14.7)	71	(19.8)
BMI category								
Underweight	386	(57.9)	291	(47.3)	79	(28.5)	50	(31.1)
Normal	4904	(54.9)	3840	(47.5)	906	(22.5)	594	(22.4)
Overweight	4177	(51.0)	3293	(44.5)	691	(19.1)	478	(20.5)
Obese	2315	(52.2)	1852	(46.0)	383	(21.6)	252	(22.0)
Unknown	3982	(49.4)	3051	(41.9)	566	(20.1)	368	(23.0)
Immunocompromised category								
Yes	1738	(49.9)	1318	(41.9)	306	(21.2)	175	(18.7)
No/unknown	14 026	(52.3)	11 009	(45.4)	2319	(21.0)	1567	(22.5)

Interpretation of the Percentages: Proportion of individuals who reported an ADR in association with a dose of a COVID-19 vaccine, stratified by demographics and by the cohort definition. Example: Amongst the male individuals who had reported a first dose and a second dose vaccination, 16.0% (*n* = 839) had reported an ADR that occurred after the second dose vaccination

*ADR* adverse drug reaction, *BMI* body mass index

Reporters were able to select at least one level of seriousness for their report to describe the impact of an ADR on their lives, although this was non-mandatory. The levels of seriousness indicated by reporters are given in OSM Resource 4. The number of individuals who had reported their ADRs with a level of seriousness was 9,670 (61.3%). These reports were associated with 22,920 events (60.1%). In the cohort of individuals who had reported a first dose in YCVM (*n* = 14,587), 8,955 (61.4%) reported their ADRs with a level of seriousness. There were 21,484 (60.3%) reactions associated with these reports. For each seriousness category, there was a high proportion of individuals who did not submit a response.

When the reported adverse reactions were categorised as medically serious according to the MedDRA classification, 4134 (13.7% of those reporting any vaccination dose) individuals reported an ADR considered medically serious. A summary table of the demographics for the individuals reporting a MedDRA serious ADR is given in OSM Resource 5.

**Fig. 2:**
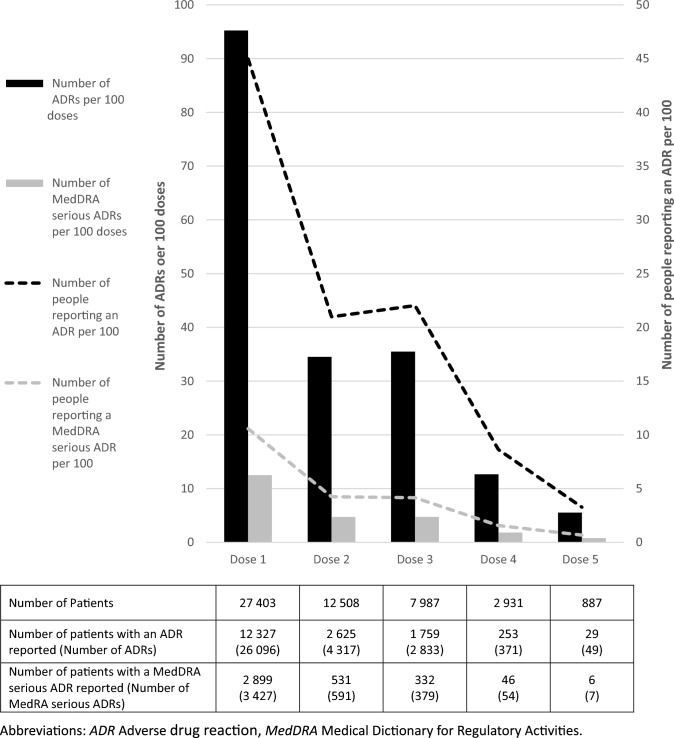
Average reporting rates of Adverse Drug Reactions (ADRs): Total ADRs and Medical Dictionary for Regulatory Activities (MedDRA) serious ADRs

Amongst the individuals who had reported at least their first dose of vaccination, 14,587 individuals had reported a total of 35,647 ADRs. Figure 2 presents the ADR reporting rates by dose for all ADRs reported and for MedDRA serious ADRs. Records for each dose are included if the individuals reported all previous doses. In addition, one individual reported a single ADR following a sixth dose. 1,093 individuals reported an ADR with no start date, therefore it could not be determined which dose the event followed.

The ten most prevalently reported ADRs for the first, second and third doses of a COVID-19 vaccination are given for each vaccine brand in OSM Resource 6. Of these ADRs, only arthralgia and myalgia were considered medically serious events according to the MedDRA medical terminology system.

OSM Resource 7 presents the number and proportion of events reported by MedDRA SOC level for each dose and by dose sequence for each vaccine brand. The ADR count listings at MedDRA SOC, HLGT, HLT and PT levels are given in OSM Resource 8.

Due to the approach taken to rollout of the national vaccination campaign to the UK population, analyses of ADR reporting across vaccine brands will potentially be confounded by age and other risk factors for severe outcomes following COVID-19 infection, with the AstraZeneca vaccine being predominantly used in individuals aged 40+ years and those with underlying health conditions and the Moderna vaccine being predominantly used as a third or subsequent dose again in an older population. Age-stratified analyses exploring the ADRs reported in those aged under 40 years and in those aged 40 years or older, presented in OSM Resource 9, do not indicate any particular differences in the ADR reporting patterns between the age groups.

As part of the COVID-19 vaccination surveillance strategy, a list of specific medical events was identified of adverse events of special interest (AESIs) which were subject to enhanced monitoring. In the YCVM, two cases of Guillain-Barre syndrome (GBS) were reported, both with a first dose of the AstraZeneca vaccine. One case of GBS was medically confirmed, while the remaining case was unconfirmed. There were also two cases of transverse myelitis (including ‘myelitis’ events) reported with a first dose of the AstraZeneca vaccine. Information provided was too limited to medically confirm these cases. Finally, there were two cases of Bell’s palsy reported. One occurred after receiving a first dose of the AstraZeneca vaccine but was not medically confirmed. The other case was confirmed by a healthcare professional and occurred after a second-dose vaccination using the Pfizer vaccine.

### COVID-19 Vaccination During Pregnancy and Breastfeeding

At the time of a COVID-19 vaccination, 2,517 females registered on the YCVM platform reported they were pregnant, although only 2,416 provided details of that vaccination. 641 females reported breastfeeding at the time of a COVID-19 vaccination. Demographics of these cohorts and those who experienced an ADR after a vaccination are presented in Table 5. The demographics of females registered, including those with no vaccination reported, are presented in OSM Resource 10.

**Table 5:** Number of vaccinated individuals reporting an ADR in females, pregnant females, and breastfeeding females registered in the Yellow Card Vaccine Monitor, stratified by demographics

	Female cohort			Pregnant female cohort			Breastfeeding cohort		
	Reported a vaccination (*n* = 17 463)	Reported an ADR after any vaccination (*n* = 10 145) (% of vaccinated females cohort)		Reported a vaccination (*n* = 2416)	Reported an ADR after any vaccination (n = 995) (% of vaccinated pregnant females cohort)		Reporting a vaccination (*n* = 641)	Reported an ADR after any vaccination (n = 343) (% of vaccinated breastfeeding females cohort)	
Age bands (years)									
Under 12	61	27	(44.3)	0	0	(0.0)	0	0	(0)
12–17	728	374	(51.4	2	0	(0.0)	0	0	(0)
18–29	1074	636	(59.2)	403	148	(36.7)	76	43	(56.6)
30–39	3057	1655	(54.1)	1857	781	(42.1)	487	260	(53.4)
40–49	1738	1302	(74.9)	153	66	(43.1)	77	40	(51.9)
50–59	2235	1563	(69.9)	1	0	(0.0)	1	0	(0.0)
60–69	3214	2051	(63.8)	0	0	(0.0)	0	0	(0.0)
70–79	4267	2110	(49.4)	0	0	(0.0)	0	0	(0.0)
80+	1077	422	(39.2)	0	0	(0.0)	0	0	(0.0)
Unknown	12	5	(41.7)	0	0	(0.0)	0	0	(0.0)
Ethnicity									
White British, White Irish, or any other White background	15 049	8815	(58.6)	1909	809	(42.4)	538	295	(54.8)
Other	1101	642	(58.3)	248	100	(40.3)	65	36	(55.4)
Unknown	1313	688	(52.4)	259	86	(33.2)	38	12	(31.6)
BMI category									
Underweight	433	278	(64.2)	26	9	(34.6)	5	3	(60.0)
Normal	5492	3298	(60.1)	690	325	(47.1)	204	115	(56.4)
Overweight	3772	2237	(59.3)	489	209	(42.7)	153	92	(60.1)
Obese	2405	1461	(60.7)	266	110	(41.4)	77	40	(51.9)
Unknown	5361	2871	(53.6)	945	342	(36.2)	202	93	(46.0)
Immunocompromised									
Yes	2195	1067	(58.2)	111	50	(45.0)	40	27	(67.5)
No	18 673	9078	(58.1)	2305	945	(41.0)	601	316	(52.6)

Interpretation of the percentages: proportion of females who reported an ADR in association with a dose of a COVID-19 vaccine, stratified by patient demographics and by the patient cohort definition. For example, amongst the pregnant females aged 30-39 years who had reported a vaccination, 42.1% (*n* = 781) had reported an ADR occurring after vaccination

*ADR* adverse drug reaction, *BMI* body mass index

In pregnant females and breastfeeding females reporting a vaccination, the median age was 33 years (IQR: 31–36) and 34 years (IQR: 31–37), respectively. The cohort of vaccinated pregnant females contributed 903.5 patient years follow-up with a median follow-up of 72.5 days (IQR: 5–244).

#### Pregnant Females

Of the vaccinated pregnant females, 41.2% (*n* = 995) reported a total of 1934 ADRs in the YCVM. There were 1255 ADRs reported in association with a first dose, 476 with a second dose, 118 with a third dose and three with a fourth dose. There were 82 ADRs with no onset date reported.

Due to the method in which pregnancy status data were collected and stored, it was not always possible to identify which dose or doses were received during pregnancy if they had reported more than one vaccination dose. As a result, all vaccination dose(s) and vaccine brand(s) reported in the YCVM by those who were pregnant are presented here. It does not confirm each reported dose was administered when they were pregnant. 1654 (68.5%) had reported the stage of their pregnancy at the time of vaccination, with 310 reporting more than one stage of pregnancy in which they were vaccinated. The stage of pregnancy reported by these pregnant females and those who also reported at least one ADR are presented in Table 6. The proportion of pregnant females experiencing an ADR after vaccination was similar across the different stages of vaccination during pregnancy.

**Table 6:** Reported stage of pregnancy during COVID-19 vaccination amongst the cohort of pregnant females

Stage of pregnancy	Number of vaccinated pregnant females (*N*=2416)	Number of vaccinated pregnant females with an ADR reported (*n* = 995) (% of vaccinated pregnant females)
First trimester (1–12 weeks)	281	132 (47.0)
Second trimester (13–28 weeks)	663	305 (46.0)
Third trimester (29–40 weeks)	400	174 (43.5)
More than one stage reported^*^	310	144 (46.5)
Unknown	762	240 (31.5)

More than one stage reported: 310 individuals reported exposure in more than one pregnancy stage and contributed the following counts: first trimester (110), second trimester (283) and third trimester (248)

*ADR* adverse drug reaction

The majority of doses reported by pregnant females were for the first dose of COVID-19 vaccination and the dominant vaccine brand was Pfizer BioNTech (Table 7). Amongst those who had reported a vaccination with AstraZeneca, 116 had reported receiving only one COVID-19 vaccination in their YCVM data and 71 reported at least one ADR. Based on the guidelines that Pfizer or Moderna vaccines were the preferred vaccines for pregnant females, it is more likely these AstraZeneca vaccinations were received prior to them becoming pregnant or when they were unaware they were pregnant during their first trimester. The median number of doses reported in the YCVM by a female who had indicated they were pregnant at the time of a vaccination was one dose (IQR: 1–2 doses). 499 females (20.7%) reported receiving a third dose or booster dose as per guidance, which recommends continued doses should be offered to pregnant females [26].

**Table 7:** Number of pregnant females registered by brand and dose and (number), [%], who reported an ADR

Dose number	Vaccine brand				Total
	Pfizer	AstraZeneca	Moderna	Other or unknown	
Dose 1	1729 (534), [30.9]	241 (129), [53.5]	183 (85), [46.4]	3 (2), [66.7]	2156 (750), [34.8]
Dose 2	901 (189), [21.0]	155 (34), [21.9]	104 (48), [46.2]	5 (0), [0.0]	1165 (271), [23.3]
Dose 3	321 (34), [10.6]	1 (0), [0.0]	169 (37), [21.9]	8 (1), [12.5]	499 (72), [14.4]
Dose 4	12 (1), [8.3]	0 (0), [0.0]	10 (1), [10.0]	0 (0), [0.0]	22 (2), [9.1]
Dose 5	0 (0), [0.0]	0 (0), [0.0]	3 (0), [0.0]	0 (0), [0.0]	3 (0), [0.0]

Data are *n* (number of vaccinated pregnant females who had reported an ADR) [% ADR reported amongst vaccinated pregnant females)

*ADR* adverse drug reaction

The most commonly reported ADRs in this cohort for the first, second and third doses of COVID-19 vaccination are given in OSM Resource 11. The reactions reported are common and recognised ADRs associated with vaccines. Pain in extremity, fatigue, headache and pyrexia are the reactions that appear in the top 4 reported ADRs across the three doses.

There were no reports of ADRs in the ‘Congenital, Familial and Genetic Disorders’ SOC. The reports of ADRs belonging to the ‘Pregnancy, Puerperium and Perinatal Conditions’ and ‘Reproductive System and Breast Disorders’ SOCs are presented in Table 8.

**Table 8:** MedDRA Preferred Terms reported in the ‘Pregnancy, Puerperium and Perinatal Conditions’ and ‘Reproductive System and Breast Disorders’ System Organ Classes (SOC) amongst pregnant females: Number of reactions reported (frequency, % of total number of ADRs) stratified by vaccine brand and dose

Total number of reactions by MedDRA SOC and PT term level	Total	Vaccine brand				Dose number			
		Pfizer	AstraZeneca	Moderna	Other or unknown brand	Dose 1	Dose 2	Dose 3	Missing ADR date
Pregnancy, Puerperium and Perinatal Conditions SOC	28 (1.4)	17 (1.5)	7 (1.9)	1 (0.3)	1 (20.0)	17 (1.4)	8 (1.7)	1 (0.8)	2 (2.4)
Abortion spontaneous	19 (1.0)	13 (1.1)	5 (1.3)	1 (0.3)	0 (0.0)	11 (0.9)	8 (1.7)	0 (0.0)	0 (0.0)
Foetal death	1 (< 0.1)	0 (0.0)	1 (0.3)	0 (0.0)	0 (0.0)	1 (< 0.1)	0 (0.0)	0 (0.0)	0 (0.0)
Foetal disorder	1 (< 0.1)	0 (0.0)	0 (0.0)	0 (0.0)	1 (20.0)	1 (< 0.1)	0 (0.0)	0 (0.0)	0 (0.0)
Foetal hypokinesia	2 (0.1)	2 (0.2)	0 (0.0)	0 (0.0)	0 (0.0)	1 (< 0.1)	0 (0.0)	1 (0.8)	0 (0.0)
Foetal macrosomia	1 (< 0.1)	1 (< 0.1)	0 (0.0)	0 (0.0)	0 (0.0)	1 (< 0.1)	0 (0.0)	0 (0.0)	0 (0.0)
Morning sickness	1 (< 0.1)	0 (0.0)	0 (0.0)	0 (0.0)	0 (0.0)	0 (0.0)	0 (0.0)	0 (0.0)	1 (1.2)
Placental infarction	1 (< 0.1)	0 (0.0)	1 (0.3)	0 (0.0)	0 (0.0)	1 (< 0.1)	0 (0.0)	0 (0.0)	0 (0.0)
Pregnancy	1 (< 0.1)	0 (0.0)	0 (0.0)	0 (0.0)	0 (0.0)	0 (0.0)	0 (0.0)	0 (0.0)	1 (1.2)
Uterine contractions during pregnancy	1 (< 0.1)	1 (< 0.1)	0 (0.0)	0 (0.0)	0 (0.0)	1 (< 0.1)	0 (0.0)	0 (0.0)	0 (0.0)
Reproductive system and breast disorders SOC	12 (0.6)	7 (0.6)	2 (0.5)	0 (0.0)	0 (0.0)	4 (0.3)	3 (0.6)	2 (1.7)	3 (3.7)
Menstrual disorder	1 (< 0.1)	1 (< 0.1)	0 (0.0)	0 (0.0)	0 (0.0)	0 (0.0)	0 (0.0)	1 (0.8)	0 (0.0)
Menstruation delayed	2 (0.1)	0 (0.0)	1 (0.3)	0 (0.0)	0 (0.0)	0 (0.0)	1 (0.2)	0 (0.0)	1 (1.2)
Oligomenorrhoea	1 (< 0.1)	0 (0.0)	0 (0.0)	0 (0.0)	0 (0.0)	0 (0.0)	0 (0.0)	0 (0.0)	1 (1.2)
Heavy menstrual bleeding	2 (0.1)	1 (< 0.1)	0 (0.0)	0 (0.0)	0 (0.0)	0 (0.0)	0 (0.0)	1 (0.8)	1 (1.2)
Vaginal haemorrhage	6 (0.3)	5 (0.4)	1 (0.3)	0 (0.0)	0 (0.0)	4 (0.3)	2 (0.4)	0 (0.0)	0 (0.0)
Total number of ADRs (all MedDRA SOCs)	1934	1161	377	309	5	1255	476	118	82

Data are *n* (% total number of ADRs reported for all SOCs)

*ADR* adverse drug reaction, *MedDRA* Medical Dictionary for Regulatory Activities*, PT* preferred term, *SOC* System Organ Class

There were 26 females who reported an ADR from the ‘Pregnancy, Puerperium and Perinatal Conditions’ SOC, with a total of 28 ADRs reported. Of the total 28 events reported in the ‘Pregnancy, Puerperium and Perinatal Conditions’ SOC category, 13 events (35.7%) were associated with 13 pregnant females who reported receiving a vaccination during their first trimester.

There were 19 reports of spontaneous abortion. The majority of these reports were reported in association with a first dose of COVID-19 vaccination (*n* = 11), seven with the Pfizer BioNTech vaccine, three with AstraZeneca and one with Moderna. The one case of foetal death was reported alongside a spontaneous abortion, and this was reported in association with a first dose of AstraZeneca. In eight (72.7%) of the reports occurring after a first dose vaccination, the pregnant females had reported vaccination during the first trimester, whilst three reports (27.3%) did not report the trimester in which they were vaccinated. The median time from the last reported vaccination to the reported date of spontaneous abortions occurring after receiving a first-dose vaccination (*n* = 11) was 27 days (IQR: 18–47 days), and in those occurring after a second-dose vaccination (*n* = 8) was 33.5 days (IQR: 24–53).

In the ‘Reproductive System and Breast Disorders’ SOC, there were 12 events associated with 12 females who had received a vaccination during their pregnancy. There were four events (33.3%) associated with four pregnant females who reported receiving a vaccination during their first trimester, three in second trimester, two in third trimester, and two with an unknown stage of pregnancy. There was one event reported by a female with multiple stages of pregnancy reported in their YCVM data.

At the time of the pregnant female’s last submission, there were seven events resolved, two recovering, nine ongoing, and ten unknowns in the ‘Pregnancy, Puerperium and Perinatal Conditions’ SOC. In the ‘Reproductive System and Breast Disorders’ SOC, there were seven resolved, two recovering, and three ongoing. The full list of ADRs reported in this cohort of pregnant females and also breastfeeding females is tabulated in OSM Resource 12.

For those who had reported an estimated pregnancy due date, a follow-up was sent 10 weeks after the due date requesting additional information on the outcome of the pregnancy such as full-term births or pre-term births. This information was recorded in the free-text narrative field of the YCVM report. There had been no reports of pre-term births reported as an ADR; however, a search of the free-text field identified four females amongst the vaccinated pregnant females who had reported a premature birth.

#### Breastfeeding Females

Of the 694 females reported as breastfeeding at the time of a vaccination, 641 had entered details of at least one COVID-19 vaccination; 53.5% (*n* = 343) of these vaccinated females who were breastfeeding reported a total of 900 ADRs in the YCVM. There were 564 ADRs reported in association with a first dose, 199 with a second dose, 81 with a third dose, and 56 with no onset date. Approximately 64.8% (*n* = 450) of breastfeeding females and 61.5% (*n* = 211) of those who went on to report an ADR had reported being vaccinated during their pregnancy.

The information collected on breastfeeding in the YCVM platform was derived from a binary ‘yes’ or ‘no’ question. As a result, where an individual recorded more than one dose, it is not possible to determine at which dose the breastfeeding occurred. The median number of doses reported in the YCVM by an individual who had indicated they were breastfeeding at the time of a vaccination was two doses (IQR: 1–3 doses). At least 235 individuals had reported receiving a third dose or booster dose.

Table 9 presents the number of doses received by individuals by sequence and by brand, during the whole follow-up time in the YCVM. The majority of reported doses were for the first-dose vaccination, with the most frequently reported vaccine brand being the Pfizer vaccine (*n* = 391).

**Table 9:** Number of breastfeeding females registered by brand and dose and (number), [%], who reported an ADR

Dose number	Vaccine brand				Total
	Pfizer	AstraZeneca	Moderna	Other or unknown	
Dose 1	391 (141), [36.1]	151 (103), [68.2]	50 (22), [44.0]	0 (0), [0.0]	592 (266), [44.9]
Dose 2	284 (64), [22.5]	57 (17), [29.8]	38 (20), [52.6]	1 (0), [0.0]	380 (101), [26.6]
Dose 3	15 (20), [12.7]	0 (0), [0.0]	75 (23), [30.7]	3 (1), [33.3]	235 (44), [18.7]
Dose 4	8 (0), [0.0]	0 (0), [0.0]	6 (0), [0.0]	0 (0), [0.0]	14 (0), [0.0]
Dose 5	0 (0), [0.0]	0 (0), [0.0]	3 (0), [0.0]	0 (0), [0.0]	3 (0), [0.0]

Data are *n* (number of vaccinated breastfeeding females who had reported an ADR) [% ADR reported amongst vaccinated breastfeeding females]

*ADR* adverse drug reaction

There were no reports of reactions relating to lactation disorders reported in this cohort. There was one report of mastitis occurring after a third-dose vaccination with the Moderna vaccine. In the ‘Pregnancy, Puerperium and Perinatal Conditions’ SOC there was one ADR reported where placental infarction occurred after a first COVID-19 vaccination with the AstraZeneca vaccine. In the ‘Reproductive System and Breast Disorders’ SOC, there were in total six reactions reported with one report of each of the following reactions: irregular menstruation, hypomenorrhea, delayed menstruation, oligomenorrhoea, heavy menstrual bleeding and vaginal haemorrhage. There were no reports of ADRs in the ‘Congenital, Familial and Genetic Disorders’ SOC.

It is possible a female individual may not indicate they were breastfeeding using the optional breastfeeding question. Amongst the ADRs reported by any female individual, there were no reports of lactation related ADRs. However, there were 25 reports of breast disorders – four reports of breast mass, one report of nipple enlargement, one report of breast discharge, 15 reports of breast pain, one report of breast swelling, one report of breast tenderness, and two reports of nipple pain.

## Case Studies

### Menstrual Disorders

The potential safety signal was first identified in spontaneous Yellow Card data through routine signal detection activities in January 2021. Of the 20,868 females registered in the YYCVM cohort, 219 (1.05%) reported 258 ADRs relating to menstrual disorder. There were 152 related reactions reported following a first dose, 45 reactions following a second dose and 23 reactions following a third dose. There were 38 reactions which did not have sufficient information to determine the preceding vaccination dose and brand.

The demographics of the individuals reporting menstrual disorder ADRs are presented in Table 10, including vaccine brand-specific demographics.

**Table 10:** Menstrual disorder case study: demographics of vaccinated females who reported menstrual disorder ADRs

	Vaccine brand				
	Total	Pfizer	AstraZeneca	Moderna	Other/unknown brand
Number of females	219	102	72	17	1
Age group (years)					
<18 years	12	11	0	0	1
18–49 years	196	88	67	17	0
50+ years	11	3	5	0	0
Ethnicity					
White British, White Irish, or any other White background	179	79	63	14	1
Other	23	15	4	2	0
Not stated	17	8	5	1	0

*ADR* adverse drug reaction

The menstrual disorder-related PT reactions reported are presented in Table 11. More than half of the three most commonly reported reactions, heavy menstrual bleeding, irregular or delayed menstruation, and dysmenorrhoea, occurred after a first-dose vaccination. The time-to-onset (TTO) after vaccination was not explored in this study because the interpretation of TTO would be complicated by the phase of the menstrual cycle an individual received their vaccination. The YCVM does not capture baseline characteristics on an individual’s menstrual cycle.

**Table 11:** Menstrual disorder case study: MedDRA Preferred Terms reported

Menstrual disorder-related reaction (MedDRA PT level)	Number of reactions reported (frequency %)	
Amenorrhoea	1	(0.4)
Dysmenorrhoea	29	(11.2)
Heavy menstrual bleeding	61	(23.6)
Hypomenorrhoea	9	(3.5)
Intermenstrual bleeding	13	(5.0)
Menstrual disorder	22	(8.5)
Menstruation delayed	47	(18.2)
Menstruation irregular	47	(18.2)
Oligomenorrhoea	3	(1.2)
Polymenorrhoea	9	(3.5)
Postmenopausal haemorrhage	1	(0.4)
Premenstrual pain	2	(0.8)
Retrograde menstruation	1	(0.4)
Vaginal haemorrhage	13	(5.0)
Total number of menstrual disorder-related ADRs	258	

*ADR* adverse drug reaction, *MedDRA* Medical Dictionary for Regulatory Activities*, PT* preferred term

At the time of the last submission submitted prior to or on 31 December 2022, 105 reactions were reported as resolved with or without sequalae, 40 as resolving, and 91 as ongoing. There were 22 reactions with no outcome reported.

**Fig. 3:**
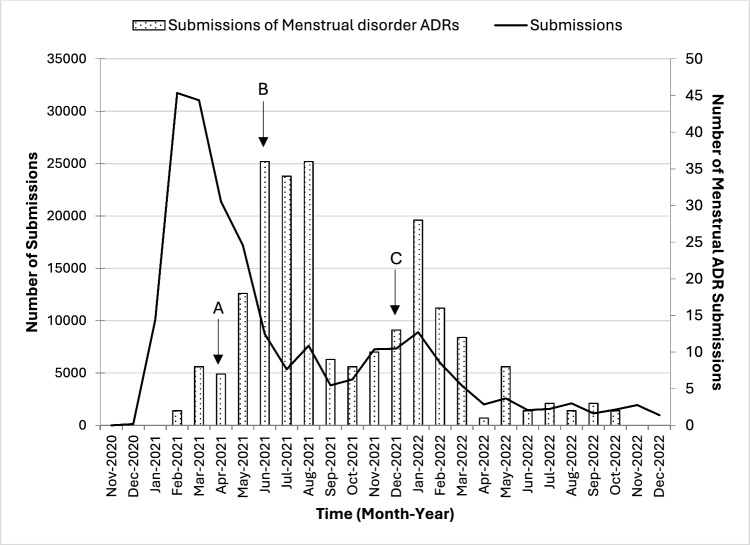
Number of Menstrual ADRs submissions in the Yellow Card Vaccine Monitor up to 31 December 2022**.** Arrows indicate key events: Arrow A for the first wave of published news stories. These included articles published by the Huffington Post website (“How The Covid-19 Vaccine May Affect Your Period (And What To Do)|HuffPost UK Life” ), the Euronews (“Experts cool claims about impact of vaccines on menstrual cycles|Euronews”), the BBC News (“Covid vaccine: Period changes could be a short-term side effect - BBC News”) and The Telegraph (“Post-menopausal females report periods coming back after having Covid vaccine (telegraph.co.uk)”). Arrow B for the key news article by Sunday Times, and C for rapid rollout of booster to under 50s. Article published by the Sunday Times on 20 June 2021, titled “4,000 females report period problems after Covid jab”. Weblink: “https://www.thetimes.com/uk/healthcare/article/4-000-females-report-period-problems-after-jab-3sdgwgx8v”. *ADR* adverse drug reaction

To explore time trends in the reporting of menstrual disorder related adverse reactions, the report submission dates of these reactions are shown in Fig. 3. The number of reports grew with increasing social media attention and the publication of several news stories on the internet (Fig. 3 Arrow A) with subsequent peaks coinciding with publication of a key major news article (Fig. 3 Arrow B) and a mass booster vaccination campaign where a large cohort of females aged 18–49 years of age were made eligible to receive their booster dose (Fig. 3 Arrow C).

### Tinnitus

Amongst the individuals who had reported receiving a first dose in the YCVM, 1.9% (*n* = 73) reported 83 tinnitus or related PT reactions (Table 12). Fifty-four reactions (from 50 individuals) were reported in association with the first dose, 16 (from 13 individuals) with the second dose, and six (from six individuals) with the third dose. There were seven reactions (from seven individuals) which did not have sufficient information to determine which dose the reaction followed.

**Table 12:** Tinnitus case study: demographics of vaccinated individuals who reported hearing loss and tinnitus ADRs

	Vaccine brand			
	Total individuals (*n* = 73)	Pfizer (*n* = 21)	AstraZeneca (*n* = 43)	Moderna (*n* = 4)
Sex				
Male	26	7	14	1
Female	46	14	28	3
Unknown	1	0	1	0
Age group (years)				
<65 years	58	17	32	4
65+ years	15	4	11	0
Ethnicity				
White British, White Irish, or any other White background	61	18	37	2
Other	6	2	3	1
Not stated	6	1	3	1

*ADR* adverse drug reaction

The reports of tinnitus were predominantly reported in association with an AstraZeneca vaccine, with ‘Tinnitus’ as the most commonly reported PT. Table 13 presents PT terms reported, the dose onset, vaccine brand and the median time to onset of the ADR after receiving the vaccination.

**Table 13:** Tinnitus Case Study: MedDRA Preferred Terms reported and onset time information by vaccination brand

	Total	Pfizer	AstraZeneca	Moderna
MedDRA PT Level (frequency % total tinnitus events)	83	22	50	4
Deafness	4 (4.8)	3 (13.6)	0 (0.0)	1 (25.0)
Deafness neurosensory	1 (1.2)	1 (4.5)	0 (0.0)	0 (0.0)
Deafness unilateral	2 (2.4)	0 (0.0)	2 (4.0)	0 (0.0)
Hyperacusis	1 (1.2)	0 (0.0)	1 (2.0)	0 (0.0)
Hypoacusis	3 (3.6)	1 (4.5)	2 (4.0)	0 (0.0)
Sudden hearing loss	1 (1.2)	0 (0.0)	1 (2.0)	0 (0.0)
Tinnitus	71 (85.5)	17 (77.3)	44 (88.0)	3 (75.0)
ADR timing (frequency % total tinnitus events)				
ADR after first dose	54 (65.1)	13 (59.1)	40 (80.0)	1 (25.0)
ADR after second dose	16 (19.3)	5 (22.7)	10 (20.0)	1 (25.0)
ADR after third dose	6 (7.2)	4 (18.2)	0 (0.0)	2 (50.0)
Timing unknown	7 (8.4)	0 (0.0)	0 (0.0)	0 (0.0)
Time to onset (median days, IQR)	2 (1–7.5)	4.5 (0–23)	2 (1–5)	0.5 (0–3.5)
First doses	1 (0–10)	1 (0–23)	1 (0.5–5)	1 (1–1)
Second doses	4 (1–5)	4 (0–4)	4 (1–5)	0 (0–0)
Third doses	5 (1–7)	6 (3–79.5)	–	3 (0–6)

Time to onset is in relation to the most recent dose administered prior to the onset of the ADR

*ADR* adverse drug reaction, *IQR* interquartile range, *MedDRA* Medical Dictionary for Regulatory Activities*, PT* preferred term

Approximately two-thirds of the reported tinnitus related reactions were ongoing at the most recent submission (*n* = 51) whilst less than a third were reported as resolved (*n* = 25), and the remaining reactions were resolving (*n* = 6) or unknown (*n* = 1).

## Discussion

The scale of the COVID-19 pandemic necessitated the implementation of a safety surveillance strategy utilising multiple data sources to provide the timely and robust data needed for regulatory decision making. While spontaneous reporting of adverse reactions through the UK Yellow Card (YC) scheme has been a central component of the MHRA’s approach to medicines and vaccines vigilance for the last 60 years, these data represent the first time that this digital form of active surveillance has been directly conducted by the MHRA and the first time the technology and infrastructure used have been available. Digital technology has played an important role in implementing active surveillance during the COVID-19 vaccination programmes globally [27]. Active surveillance using cohort event monitoring (CEM) systems was conducted for COVID-19 vaccines by other organisations, including by public health bodies and regulators. However, the majority of these were not particularly designed to be integrated with spontaneous reporting directly into a regulatory adverse drug reaction database.

### Recruitment and Engagement

Up to 31 December 2022, over 1.4 million individuals were invited to register with the YCVM. Female individuals and those aged over 50 years were more likely to register and provide vaccination data although the characteristics of individuals included will have been influenced by differences in vaccine uptake, timing of recruitment drives, and an individual’s propensity to report. Invitations to register were targeted to new at-risk groups through the system established for inviting people in England to book their vaccination, while pregnant females were targeted through printed materials given to them at their vaccine appointment. However, as with any voluntary reporting scheme, it is challenging to ensure a representative population, and we are unable to access data on those who were invited to register but did not. One of the aims of YCVM was to target enrolment of ethnic minorities under-represented in clinical trials. In the YCVM, approximately 86% of registered individuals belonged to the ‘White British, White Irish or Other White’ ethnicity group. This proportion is larger than the estimate across the UK population [28], suggesting that representation of the other ethnic groups was limited despite targeted recruitment. Approximately 35,000 invites were sent during one call for registrations in May 2021, with 80% of these sent to non-White British/non-White Irish individuals. These targeted invites to under-represented ethnic groups had little to no impact on the numbers registering from this group, reflecting possible lower levels of engagement with passive reporting systems seen in non-white populations. The need to increase the diversity of individuals reporting adverse reactions is well recognised even within spontaneous reporting systems and broader efforts to increase the visibility of, and engagement with, vigilance systems in different communities is needed.

Due to the system implemented during the pandemic period to directly invite people to book and receive a vaccination, there was a particular opportunity for prospective recruitment; however, nearly half of individuals registered had a registration date after their earliest vaccination date. This limits interpretation of the totality of the data with regard to acute reactogenicity reports as the propensity to register after vaccination may be influenced by the occurrence of early reactions. Consideration also needs to be given to how drop-out from reporting of second and subsequent doses can be reduced.

### Adverse Drug Reaction Reporting and Contribution to Evidence Base

Generally, the types of ADRs reported through the YCVM aligned with those reported through the YC passive reporting system [29]. Patterns of reporting also reflected those seen in pre-authorisation clinical trials with higher reporting of local and systemic events following administration of the Pfizer mRNA vaccine in younger individuals and after a second dose [30] and in younger individuals after a first dose following administration of the AstraZeneca adenoviral vectored vaccine [31]. However, the interpretation of these data, and those data for the Moderna mRNA vaccine, are complicated by the rollout of the vaccines in the UK with correlation between the vaccine brand used and the age of the individual and dose number.

Data from YCVM has supported signal assessment alongside routine signal detection activities using other data sources. It has not identified any additional safety signals in isolation, which is to be expected given the size of the cohort involved and the design. Information from ADR reports submitted via the YCVM has contributed to the evaluation of potential safety signals with a COVID-19 vaccine alongside that accrued through the other three pillars of the COVID-19 vaccine safety surveillance strategy.

Vaccination in pregnancy was not recommended in England until April 2021. Inclusion of YCVM data has contributed to the safety review for COVID-19 vaccines in pregnancy and breastfeeding, which concluded the YC data did not raise any safety concerns with COVID-19 vaccine exposure during pregnancy. However, as with passive spontaneous reporting, the completeness of reporting is unknown. The rate of early pregnancy loss is uncertain but is estimated to occur in around 15% of recognised pregnancies with a greater risk earlier in pregnancy and in older mothers, with certain long-term health conditions, weight, and lifestyle factors also being risk factors [32]. The absolute rate of pregnancy loss reported into the YCVM was lower than the expected rate. However, these figures cannot be directly compared as the underlying risks at the point of vaccination for these pregnant females are unknown. Longer follow-up would be required from pregnant and breastfeeding females to collect data on ADRs relating to their infants and developmental outcomes. Subsequent epidemiological studies demonstrate the safety of these vaccines in pregnancy, showing no evidence of an increased risk of adverse pregnancy outcomes [33, 34]. These studies enable comparative analyses across substantially larger cohorts of vaccinated and unvaccinated pregnant females and allow for the adjustment of key confounding variables including gestational and maternal age, meaning they present more robust evidence of vaccine safety in pregnancy.

In the menstrual disorders case study, YCVM data were used to support and facilitate discussions on the emerging safety signal. Whilst overall assessment at the time concluded there was no evidence of an increased risk of menstrual disorders, it was recognised that media interest played a possible role in the increased reporting of menstrual disorder reactions in the YCVM and the YC scheme. Data from the YCVM cannot be used to assess if there is an association between vaccination and menstrual events, but improvements to follow-up could support better assessment of patient experiences and outcomes. Heavy menstrual bleeding was included in the product information for the Pfizer and Moderna vaccines following review [35].

For tinnitus, the potential safety signal first arose from the YC data during routine signal detection activities. Data from YCVM was included in the MHRA reviews conducted on the available evidence, alongside spontaneous YC data, clinical trial data, and published literature. The number of individuals reporting a tinnitus-related ADR in the YCVM was small, which emphasised the use of YCVM data in a supportive role in signal assessment and not in isolation, as well as providing reassurance on the potential incidence rate. The average time to onset for the reports related to the AstraZeneca vaccine was slightly shorter compared to the reports with the Pfizer vaccine. Tinnitus was added as an adverse reaction to the product information for the AstraZeneca COVID-19 vaccine [36].

### Technology and Infrastructure

The YCVM platform benefits from an automated follow-up reminder system which aims to capture up-to-date information on an individual’s characteristics, vaccination records and any ADR(s) reported, and extend the active follow-up time for data collection. This supported a 70% successful follow-up rate in June 2021, which only dropped to 62% in March 2022, and can be compared to less than a 10% success rate for follow-up of spontaneous cases using traditional routes. It allows individuals to have control of the information they share and how frequently they update their information. These include acute but short-lived conditions that may not be well captured by electronic health records because medical assistance was not sought from a clinician. Although follow-up was substantially increased compared to that achieved for spontaneously reported data, improvement could be made to better capture final patient outcomes.

The YCVM is currently not automatically linked to other sources of health data, therefore it is not possible to medically confirm self-reported ADRs reported through the YCVM. This was of particular importance when adverse events of special interest (AESI) such as GBS and Bell’s palsy were reported.

The data architecture of the YCVM was driven by the processing and integration of data in YCVM into the MHRA’s ADR reporting database. This has an impact on the data model and consequently the structure of the YCVM dataset used in conducting data analysis outside of the MHRA’s standard signal detection procedures. In addition, a number of variables are captured once in each submission report, which makes relationship assertions between variables unclear.

The capture of data from pregnant individuals could be improved further to enable use of the YCVM as an active surveillance tool for prospective monitoring of pregnant females. For example, at present, it is not possible to differentiate between due dates and the date of the pregnancy outcome and is not possible to indicate if an individual experienced multiple pregnancy episodes during their time in the YCVM.

### Future Development and Use

The YCVM demonstrated the capability to collect longitudinal data from vaccinees through a user-friendly interface and engagement model. Data were collected in standard formats for medicines regulators, meaning that any reports of side effects could be rapidly evaluated alongside spontaneous reports received via the YC scheme. However, due to the nature of collecting reports in this format, analysis of the totality of data (specifically where there were no side effects, or where there was a change between follow-up cycles) was challenging.

Future developments will focus on improving data formats for this type of analysis and increasing levels of validation to reduce the level of data cleansing required. Updates to improve data quality and data collection and storage methods could facilitate the creation of a pregnancy outcome data set for the purpose of close monitoring of pregnancy outcomes and exposure to vaccines to support case assessment; such approaches, including smart forms and conditional questions based on the information provided by the reporter, could deliver data akin to registry data, but at significantly lower cost. The inclusion of mandatory questions such as the reporter seriousness categories would collect information on patient-reported outcomes (PROs) in relation to a patient’s tolerability of the suspected ADRs experienced with COVID-19 vaccination.

Key to the successful collection of data through the YCVM was that information was included within the system for vaccine invites. This was a unique opportunity during the pandemic, but even with this capability a substantial proportion registered post vaccination. For a genuine unbiased dataset stricter control would be required, either through exclusion criteria applied to the overall analysis or through a mandate for registration prior to receiving the product. This latter option would require careful coordination and management across the healthcare system.

More realistic may be an active follow-up approach where individuals who report spontaneously to the YC scheme are subsequently consented for ongoing engagement to enable better understanding of the long-term nature of any events experienced, and their recovery. An alternative, or additional, approach would be linkage of YC data to that within the electronic healthcare record for assessment of specific signals.

The technology behind the YC scheme now has the capability for all such types of engagement with reporter’s incidents related to medicines, vaccines and medical devices, substantially increasing the MHRA’s vigilance options, depending on the specific products under consideration.

In addition to validation of the side effect profiles for vaccines in situations such as a pandemic, the MHRA considers that active surveillance may be particularly valuable in situations where there is extremely low or niche usage of a product or where long follow-up is required. One example may be for products such as for Advanced Therapy Medicinal Products (ATMPs), where review of high-quality data for small numbers of individuals will be critical. Such situations are likely to have highly engaged patient populations and are less likely to require the volume of invites that was required to obtain a representative population in YCVM. Although ensuring representativeness of the enrolled population, for example across ethnicities, would be challenging, further efforts to increase engagement with vigilance systems through community leaders and through patient charities could be helpful.

## Conclusions

Overall, adverse reaction reporting into the YCVM provided a complementary data source to the other strands of the MHRA COVID-19 vaccine safety surveillance strategy, which utilised both spontaneous reports and large electronic healthcare record databases. However, the investment in infrastructure has advanced UK regulatory pharmacovigilance systems that can support targeted prospective monitoring of products in real-world use in the future, capturing data directly from individuals on their experiences.

## Supplementary Information

Below is the link to the electronic supplementary material.

Supplementary file1 (PDF 533 KB)

Supplementary file2 (PDF 711 KB)

Supplementary file3 (PDF 5776 KB)

Supplementary file4 (PDF 424 KB)

Supplementary file5 (PDF 550 KB)

Supplementary file6 (PDF 605 KB)

Supplementary file7 (PDF 555 KB)

Supplementary file8 (PDF 548 KB)

Supplementary file9 (PDF 661 KB)

Supplementary file10 (PDF 780 KB)

Supplementary file11 (PDF 4718 KB)

Supplementary file12 (PDF 726 KB)

## Declarations

### Availability of data and material

All data generated or analysed during this study are included in this published article (and its supplementary information files). The Agency provides information to the public in line with requirements of the Freedom of Information Act. In addition, organisations and individuals can apply for Yellow Card data for research purposes. Applications include provision of a protocol and will be considered by the UK Commission of Human Medicine's (CHM) Pharmacovigilance Expert Advisory Group.
