# Supplementary material for: Implementation and Results of Active Vaccine Safety Monitoring During the COVID-19 Pandemic in the UK: A Regulatory Perspective
Source: Drug Saf. 2025 Sep 3;48(12):1365–85. doi: 10.1007/s40264-025-01579-w (PMC12605443; doi:10.1007/s40264-025-01579-w)
Supplement: Supplementary file 1 — Supplementary file1 (PDF 533 KB) [file 40264_2025_1579_MOESM1_ESM.pdf]

Online Resource 1

Electronic Supplementary material

Article Title: Implementation and results of active vaccine safety monitoring during the COVID-19 pandemic in the UK: a regulatory perspective

Journal for Submission: Drug Safety (Springer Nature)

Authors: Jenny Wong, Katherine Donegan, Kendal Harrison, Tahira Jan, Alison Cave, and Phil Tregunno

Author Affiliation: Medicines and Healthcare products Regulatory Agency, London, UK

Corresponding Author: Phil Tregunno, phil.tregunno@mhra.gov.uk

MedDRA Event Search Terms for Case Studies

Supplementary Table 1. MedDRA Preferred Terms (PT) considered for Menstrual Disorders Case Study

All events within the following MedDRA High Level Term (HLT) fields were included in the search criteria: "MENSTRUATION AND UTERINE BLEEDING NEC", "MENSTRUATION WITH DECREASED BLEEDING", "MENSTRUATION WITH INCREASED BLEEDING", "VULVOVAGINAL DISORDERS NEC".

MedDRA Preferred Term (PT)
Postmenopausal haemorrhage
Uterine haemorrhage
Vaginal haemorrhage
Abnormal uterine bleeding
Amenorrhoea
Dysmenorrhoea
Heavy menstrual bleeding
Hypomenorrhoea
Intermenstrual bleeding
Menometrrorhagia
Menstrual discomfort

Menstrual disorder
Menstruation delayed
Menstruation irregular
Oligomenorrhoea
Polymenorrhoea
Premenstrual dysphoric disorder
Premenstrual headache
Premenstrual pain
Premenstrual syndrome
Withdrawal bleed
Retrograde menstruation

Table S1.2 MedDRA® Preferred Terms (PT) considered for Tinnitus Case Study

**MedDRA® Preferred Term (PT)**
Acoustic neuritis
Acoustic stimulation tests abnormal
Altered pitch perception
Audiogram abnormal
Auditory disorder
Auditory recruitment
Autophony
Barotitis media
Bone anchored hearing aid implantation
Cochlea implant
Conductive deafness
Deafness
Deafness bilateral
Deafness neurosensory
Deafness occupational
Deafness permanent
Deafness transitory
Deafness unilateral
Diplacusis

Dysacusis
Electrocochleogram abnormal
Eustachian tube disorder
Eustachian tube dysfunction
Eustachian tube obstruction
Haematotympanum
Hearing aid therapy
Hyperacusis
Hypoacusis
Middle ear adhesions
Middle ear effusion
Middle ear inflammation
Misophonia
Mixed deafness
Myringitis
Neonatal deafness
Neonatal hypoacusis
Neurosensory hypoacusis
Noninfective myringitis
Ossicle disorder
Otoacoustic emissions test abnormal
Otosalpingitis
Otosclerosis
Ototoxicity
Presbyacusis
Rinne tuning fork test abnormal
Sudden hearing loss
Tinnitus
Tinnitus retraining therapy
Tympanic membrane atrophic
Tympanic membrane disorder
Tympanic membrane perforation
Tympanic membrane scarring

Tympanometry abnormal
Tympanosclerosis
Weber tuning fork test abnormal
