# Supplementary material for: Implementation and Results of Active Vaccine Safety Monitoring During the COVID-19 Pandemic in the UK: A Regulatory Perspective
Source: Drug Saf. 2025 Sep 3;48(12):1365–85. doi: 10.1007/s40264-025-01579-w (PMC12605443; doi:10.1007/s40264-025-01579-w)
Supplement: Supplementary file 2 — Supplementary file2 (PDF 711 KB) [file 40264_2025_1579_MOESM2_ESM.pdf]

Online Resource 2

Electronic Supplementary material

Article Title: Implementation and results of active vaccine safety monitoring during the COVID-19 pandemic in the UK: a regulatory perspective

Journal for Submission: Drug Safety (Springer Nature)

Authors: Jenny Wong, Katherine Donegan, Kendal Harrison, Tahira Jan, Alison Cave, and Phil Tregunno

Author Affiliation: Medicines and Healthcare products Regulatory Agency, London, UK

Corresponding Author: Phil Tregunno, phil.tregunno@mhra.gov.uk

Follow-up Time contributed from Vaccinated Cohort.

Supplementary Table 2. Follow-up time amongst Vaccinated Cohort stratified by Sex of Individual

**Follow-up time (days)**	**Male Individuals (n=12,424)**		**Female Individuals (n=17,463)**		**Unknown sex (n=394)**	
	**Total**	**Median (IQR)**	**Total**	**Median (IQR)**	**Total**	**Median (IQR)**
Number of registered Individuals	3,040,806	248.5 (16-383)	3,701,682	179 (10-363)	66,217	73.5 (1-333)
**Age bands (years)**						
Under 12	3,979	23 (1-182.5)	6,749	18 (1-183)	761	8 (1-135)
12-17	43,521	16 (2-135)	63,311	18 (2-127)	830	12 (1.5-71)
18-29	32,875	16 (1-204)	107,257	15 (1-183)	1,587	8 (1-183)
30-39	71,421	52 (1-244)	412,934	60 (4-244)	4,835	16 (1-208)
40-49	173,242	81 (8.5-287)	289,443	79 (8-283)	3,232	23 (5-244)
50-59	391,706	264 (17-359)	541,444	273 (17-366)	8,634	141.5 (6.5-346)
60-69	796,961	297 (58-450)	925,917	296 (57-451)	15,698	121 (1-367)
70-79	1,262,335	324 (57-408)	1,148,674	286 (39-405)	24,659	183 (8-365)
80+	262,237	181 (11-369)	203,497	93 (6-360)	5,680	22 (1-173)
Unknown	2,529	54.5 (3-508)	2,456	181.5 (1-352)	301	301 (301-301)
**Ethnicity**						
White British, White Irish, or any other white background	2,811,518	273 (18-387)	3,361,621	184 (14-366)	35,675	220.5 (15.5-366)

Follow-up time (days)	Male Individuals (n=12,424)		Female Individuals (n=17,463)		Unknown sex (n=394)	
	**Total**	**Median (IQR)**	**Total**	**Median (IQR)**	**Total**	**Median (IQR)**
Other	106,435	69.5 (6-276)	153,617	57 (3-253)	2,235	82.5 (4-242)
Unknown	122,853	89 (8-317)	186,444	62 (1-259)	28,307	19 (1-205)
**BMI Category**						
Underweight	45,870	133 (5-366)	98,737	189 (9-375)	1,658	259.5 (14-367)
Normal	895,431	275 (23-404.5)	1,282,993	204 (15-367.5)	10,748	110 (7.5-366)
Overweight	1,126,374	275 (30-390)	871,081	204 (15-367)	10,755	184 (65-312)
Obese	490,424	269.5 (16-373.5)	509,143	172 (13-362)	6,347	312 (6-436)
Unknown	482,707	99 (9-358)	939,728	85 (7-317)	36,709	22 (1-296)
**Immunocompromised**						
Yes	397,508	270 (25-378)	405,225	183 (11-366)	8,860	312 (47-367)
No/Unknown	2,643,298	247 (16-384)	3,296,457	178 (10-363)	57,357	66 (1-312)
**Reported as pregnant at time of a vaccination**						
Yes	-	-	330,018	72.5 (5-244)	0	0 (0-0)
No/Unknown	3,040,806	248.5 (16-383)	3,371,664	183 (12-367)	66,217	73.5 (1-333)

Abbreviations: *ADR* Adverse Drug Reaction, *BMI* Body Mass Index, *IQR* Interquartile Range
