# Supplementary material for: Implementation and Results of Active Vaccine Safety Monitoring During the COVID-19 Pandemic in the UK: A Regulatory Perspective
Source: Drug Saf. 2025 Sep 3;48(12):1365–85. doi: 10.1007/s40264-025-01579-w (PMC12605443; doi:10.1007/s40264-025-01579-w)
Supplement: Supplementary file 3 — Supplementary file3 (PDF 5776 KB) [file 40264_2025_1579_MOESM3_ESM.pdf]

Online Resource 3

Electronic Supplementary material

Article Title: Implementation and results of active vaccine safety monitoring during the COVID-19 pandemic in the UK: a regulatory perspective

Journal for Submission: Drug Safety (Springer Nature)

Authors: Jenny Wong, Katherine Donegan, Kendal Harrison, Tahira Jan, Alison Cave, and Phil Tregunno

Author Affiliation: Medicines and Healthcare products Regulatory Agency, London, UK

Corresponding Author: Phil Tregunno, phil.tregunno@mhra.gov.uk

Demographics of Registered Individuals by Vaccine Brand

These tables display the proportion of individuals who reported an ADR in association with a dose of the branded vaccine – any dose, 1st dose, 2nd dose, and 3rd dose. The denominators are the total number of individuals who received the branded dose (i) at any dose, or for their 1st dose, or (ii) for their 2nd dose and also received any branded 1st dose, or for their third dose and had also received any branded 1st and 2nd doses.

Supplementary Table 3. Demographics of Individuals who received a Pfizer COVID-19 Vaccine by Dose and ADR reporting

	Individuals reporting an ADR after a Pfizer dose, n=5,385 (% of those reporting any vaccine dose)		Individuals reporting an ADR after Pfizer 1st dose, n=3,353 (% of those reporting a 1st vaccine dose)		Individuals reporting an ADR after Pfizer 2nd dose, n=977 (% of those reporting 1st and 2nd vaccine doses)		Individuals reporting an ADR after Pfizer 3rd dose, n= 1,124(% of those reporting 1st, 2nd, and 3rd vaccine doses)	
**Sex**								
Male	1,719	(26.0)	1,034	(27.1)	304	(18.4)	377	(14.1)
Female	3,598	(37.1)	2,274	(36.1)	662	(25.2)	736	(22.7)
Unknown	68	(32.7)	45	(32.4)	11	(22.4)	11	(19.3)

	Individuals reporting an ADR after a Pfizer dose, n=5,385 (% of those reporting any vaccine dose)		Individuals reporting an ADR after Pfizer 1st dose, n=3,353 (% of those reporting a 1st vaccine dose)		Individuals reporting an ADR after Pfizer 2nd dose, n=977 (% of those reporting 1st and 2nd vaccine doses)		Individuals reporting an ADR after Pfizer 3rd dose, n= 1,124(% of those reporting 1st, 2nd, and 3rd vaccine doses)	
**Age bands (years)**								
Under 12	30	(33.3)	28	(34.6)	4	(12.5)	1	(16.7)
12-17	559	(46.3)	510	(42.8)	76	(30.4)	11	(17.5)
18-29	488	(47.7)	396	(43.2)	99	(29.3)	30	(23.1)
30-39	1,135	(43.2)	896	(39.7)	245	(27.7)	66	(15.9)
40-49	396	(41.3)	229	(43.6)	61	(28.5)	114	(28.3)
50-59	406	(31.7)	132	(40.0)	59	(34.7)	189	(25.7)
60-69	842	(30.6)	316	(31.6)	129	(22.9)	357	(23.3)
70-79	1,237	(24.0)	652	(22.5)	250	(16.9)	311	(13.4)
80+	291	(20.7)	194	(18.6)	54	(13.4)	45	(12.1)
Unknown	1	(8.3)	0	(0.0)	0	(0.0)	0	(0.0)
**Ethnicity**								
White British, White Irish, or any other white background	4,570	(32.1)	2,753	(32.3)	847	(22.6)	1,019	(18.6)
Other	469	(42.2)	358	(40.4)	81	(28.7)	60	(26.9)
Unknown	346	(29.3)	242	(29.2)	49	(16.2)	45	(16.9)
**BMI Category**								
Underweight	170	(40.2)	114	(38.5)	31	(27.9)	34	(27.9)
Normal	1,737	(34.5)	1,008	(33.5)	330	(23.7)	399	(19.7)
Overweight	1,266	(29.7)	752	(31.4)	236	(21.1)	300	(16.6)
Obese	697	(31.4)	433	(33.0)	130	(23.3)	152	(18.1)
Unknown	1,515	(33.2)	1,046	(32.3)	250	(21.8)	239	(20.3)
**Immunocompromised**								
Yes	608	(31.7)	347	(30.1)	109	(21.4)	127	(17.0)
No/Unknown	4,777	(32.7)	3,006	(33.0)	868	(22.7)	997	(19.1)
**Reported as pregnant at time of vaccination**								
Yes	687	(34.3)	534	(30.9)	145	(20.0)	29	(10.2)
No/Unknown	4,698	(32.4)	2819	(33.1)	832	(23.1)	1095	(19.3)

Abbreviations: *ADR* Adverse Drug Reaction, *BMI* Body Mass Index, *IQR* Interquartile Range

Supplementary Table 4. Demographics of Individuals who received an AstraZeneca COVID-19 Vaccine by Dose and ADR reporting

	Individuals reporting an ADR after an AstraZeneca dose, n=9,631 (% of those reporting any vaccine dose)		Individuals reporting an ADR after an AstraZeneca 1st dose, n=8,547 (% of those reporting a 1st vaccine dose)		Individuals reporting an ADR after an AstraZeneca 2nd dose, n=1,508 (% of those reporting 1st and 2nd vaccine doses)		Individuals reporting an ADR after an AstraZeneca 3rd dose, n=6 (% of those reporting 1st, 2nd, and 3rd vaccine doses)	
**Sex**								
Male	3,436	(44.4)	3,021	(42.8)	499	(14.5)	1	(6.3)
Female	6,084	(62.3)	5,428	(60.4)	1,001	(23.9)	5	(14.3)
Unknown	111	(52.4)	98	(51.9)	8	(10.1)	0	(0.0)
**Age bands (years)**								
Under 12	7	(31.8)	7	(43.8)	0	(0.0)	0	(0.0)
12-17	0	(0.0)	0	(0.0)	0	(0.0)	0	(0.0)
18-29	207	(72.4)	197	(72.4)	17	(22.7)	0	(0.0)
30-39	582	(72.7)	533	(72.0)	52	(19.9)	0	(0.0)
40-49	1,449	(73.9)	1,345	(73.4)	185	(25.1)	0	(0.0)
50-59	2,110	(60.7)	1,883	(60.2)	316	(21.6)	0	(0.0)
60-69	2,624	(55.5)	2,284	(53.3)	485	(20.7)	3	(23.1)
70-79	2,298	(42.8)	1,982	(40.1)	405	(16.5)	3	(15.0)
80+	346	(33.2)	309	(31.1)	47	(12.4)	0	(0.0)
Unknown	8	(47.1)	7	(46.7)	1	(25.0)	0	(0.0)
**Ethnicity**								
White British, White Irish, or any other white background	8,649	(54.6)	7,658	(52.8)	1,401	(19.8)	6	(13.6)
Other	431	(59.9)	393	(59.0)	56	(22.6)	0	(0.0)
Unknown	551	(47.5)	496	(47.5)	51	(12.8)	0	(0.0)
**BMI Category**								
Underweight	208	(64.4)	173	(57.9)	46	(29.3)	1	(100.0)
Normal	3,023	(57.8)	2,686	(55.9)	525	(21.1)	3	(37.5)
Overweight	2,747	(52.8)	2,433	(51.0)	416	(17.6)	1	(7.7)
Obese	1,531	(53.9)	1,372	(52.6)	237	(20.2)	1	(10.0)
Unknown	2,122	(51.5)	1,883	(50.2)	284	(18.4)	0	(0.0)
**Immunocompromised**								
Yes	1,075	(51.3)	944	(49.4)	185	(20.8)	2	(16.7)
No/Unknown	8,556	(54.8)	7,603	(53.1)	1,323	(19.4)	4	(9.5)
**Reported as pregnant at time of vaccination**								
Yes	156	(54.5)	129	(53.5)	13	(11.7)	0	(0.0)
No/Unknown	9,475	(54.4)	8418	(52.7)	1495	(19.7)	6	(11.1)

Abbreviations: *ADR* Adverse Drug Reaction, *BMI* Body Mass Index, *IQR* Interquartile Range

Supplementary Table 5. Demographics of Individuals who received a Moderna COVID-19 Vaccine by Dose and ADR reporting

	Individuals reporting an ADR after a Moderna dose, n= 1,302 (% of those reporting any vaccine dose)		Individuals reporting an ADR after Moderna 1st dose, n=300 (% of those reporting a 1st vaccine dose)		Individuals reporting an ADR after Moderna 2nd dose, n=121 (% of those reporting 1st and 2nd vaccine doses)		Individuals reporting an ADR after Moderna 3rd dose, n=576 (% of those reporting 1st, 2nd, and 3rd vaccine doses)	
**Sex**								
Male	428	(22.0)	73	(51.0)	29	(47.5)	199	(29.7)
Female	864	(34.8)	225	(56.0)	90	(48.1)	373	(38.5)
Unknown	10	(25.0)	2	(40.0)	2	(66.7)	4	(44.4)
**Age bands (years)**								
Under 12	3	(25.0)	1	(50.0)	0	(0.0)	0	(0.0)
12-17	6	(33.3)	1	(20.0)	1	(100.0)	4	(44.4)
18-29	118	(59.3)	72	(63.7)	25	(58.1)	32	(41.6)
30-39	254	(46.0)	133	(46.8)	62	(48.4)	67	(30.0)
40-49	202	(51.3)	89	(65.9)	29	(46.0)	85	(40.9)
50-59	228	(38.5)	0	(0.0)	2	(33.3)	176	(43.2)
60-69	228	(22.3)	3	(100.0)	1	(20.0)	160	(31.3)
70-79	238	(16.4)	1	(20.0)	1	(33.3)	51	(26.6)
80+	25	(11.2)	0	(0.0)	0	(0.0)	1	(6.7)
Unknown	0	(0.0)	0	(0.0)	0	(0.0)	0	(0.0)
**Ethnicity**								
White British, White Irish, or any other white background	1,149	(28.4)	251	(57.2)	102	(49.8)	517	(34.8)
Other	78	(42.6)	30	(46.9)	12	(42.9)	36	(41.9)
Unknown	75	(30.6)	19	(40.4)	7	(38.9)	23	(30.7)
**BMI Category**								
Underweight	28	(33.3)	1	(20.0)	1	(50.0)	14	(42.4)
Normal	450	(28.9)	115	(58.4)	48	(52.7)	189	(33.9)
Overweight	349	(27.3)	75	(55.6)	31	(47.7)	161	(35.5)
Obese	176	(28.7)	26	(47.3)	13	(56.5)	95	(37.5)
Unknown	299	(31.9)	83	(52.5)	28	(40.0)	117	(33.5)
**Immunocompromised**								
Yes	103	(19.8)	8	(33.3)	6	(54.5)	43	(30.9)
No/Unknown	1,199	(30.4)	292	(55.5)	115	(47.9)	533	(35.3)
**Reported as pregnant at time of vaccination**								
Yes	149	(40.6)	85	(46.4)	38	(44.7)	33	(22.3)
No/Unknown	1,153	(28.1)	215	(58.6)	83	(50.0)	543	(36.2)

Abbreviations: *ADR* Adverse Drug Reaction, *BMI* Body Mass Index, *IQR* Interquartile Range

Supplementary Table 6. Demographics of Individuals who received any other remaining brands or unknown brands of a COVID-19 vaccine by Dose and ADR reporting

	Individuals reporting an ADR after an Other/unknown vaccine dose, n=200 (% of those reporting any vaccine dose)		Individuals reporting an ADR after Other/unknown 1st dose, n=127 (% of those reporting a 1st vaccine dose)		Individuals reporting an ADR after Other/unknown 2nd dose, n=19 (% of those reporting 1st and 2nd vaccine doses)		Individuals reporting an ADR after Other/unknown 3rd dose, n=36 (% of those reporting 1st, 2nd, and 3rd vaccine doses)	
**Sex**								
Male	75	(14.9)	45	(28.1)	7	(7.8)	15	(14.4)
Female	122	(20.5)	82	(40.0)	11	(9.6)	20	(17.9)
Unknown	3	(11.1)	0	(0.0)	1	(25.0)	1	(33.3)
**Age bands (years)**								
Under 12	0	(0.0)	0	(0.0)	0	(0.0)	0	(0.0)
12-17	13	(32.5)	8	(36.4)	4	(30.8)	0	(0.0)
18-29	7	(31.8)	4	(40.0)	0	(0.0)	2	(28.6)
30-39	5	(20.0)	5	(38.5)	0	(0.0)	0	(0.0)
40-49	7	(18.9)	5	(38.5)	0	(0.0)	2	(25.0)
50-59	34	(33.0)	26	(59.1)	2	(12.5)	2	(20.0)
60-69	47	(22.0)	36	(56.3)	3	(10.3)	3	(10.0)
70-79	73	(14.3)	31	(22.5)	10	(10.4)	26	(20.0)
80+	14	(8.8)	12	(18.5)	0	(0.0)	1	(4.3)
Unknown	0	(0.0)	0	(0.0)	0	(0.0)	0	(0.0)
**Ethnicity**								
White British, White Irish, or any other white background	176	(17.3)	108	(34.0)	19	(9.7)	32	(15.6)
Other	9	(32.1)	8	(50.0)	0	(0.0)	1	(25.0)
Unknown	15	(18.5)	11	(26.2)	0	(0.0)	3	(30.0)
**BMI Category**								
Underweight	6	(18.2)	3	(20.0)	1	(14.3)	2	(40.0)
Normal	42	(14.5)	31	(40.3)	3	(5.1)	5	(9.3)
Overweight	66	(19.3)	33	(32.7)	8	(11.3)	14	(21.2)
Obese	29	(19.0)	21	(42.0)	3	(15.0)	4	(10.3)
Unknown	57	(18.5)	39	(29.3)	4	(7.8)	11	(20.0)
**Immunocompromised**								
Yes	30	(18.1)	19	(31.7)	6	(20.0)	3	(8.1)
No	170	(17.7)	108	(34.2)	13	(7.3)	33	(18.1)
**Reported as pregnant at time of vaccination**								
Yes	3	(18.8)	2	(66.7)	0	(0.0)	1	(14.3)
No/Unknown	197	(17.8)	125	(33.5)	19	(9.4)	35	(16.5)

Abbreviations: *ADR* Adverse Drug Reaction, *BMI* Body Mass Index, *IQR* Interquartile Range
