# Supplementary material for: Implementation and Results of Active Vaccine Safety Monitoring During the COVID-19 Pandemic in the UK: A Regulatory Perspective
Source: Drug Saf. 2025 Sep 3;48(12):1365–85. doi: 10.1007/s40264-025-01579-w (PMC12605443; doi:10.1007/s40264-025-01579-w)
Supplement: Supplementary file 4 — Supplementary file4 (PDF 424 KB) [file 40264_2025_1579_MOESM4_ESM.pdf]

Online Resource 4

Electronic Supplementary material

Article Title: Implementation and results of active vaccine safety monitoring during the COVID-19 pandemic in the UK: a regulatory perspective

Journal for Submission: Drug Safety (Springer Nature)

Authors: Jenny Wong, Katherine Donegan, Kendal Harrison, Tahira Jan, Alison Cave, and Phil Tregunno

Author Affiliation: Medicines and Healthcare products Regulatory Agency, London, UK

Corresponding Author: Phil Tregunno, phil.tregunno@mhra.gov.uk

Self-Reported Adverse Drug Reaction (ADR) Seriousness

Supplementary Table 7. Impact of ADRs experienced using the self-reported seriousness categories, as reported by the Individual

Reporter Seriousness category	Number of Individuals reporting any vaccine dose and reporting an ADR (% of those reporting any dose)			Number of Individuals reporting a 1st dose vaccination and reporting an ADR report (% of those reporting a 1st dose)		
	Yes	No	No response	Yes	No	No response
Mild	3,996 (25.3)	971 (6.2)	10,797 (68.5)	3,697 (25.3)	916 (6.3)	9,974 (68.4)
Uncomfortable	3,741 (23.7)	976 (6.2)	11,047 (70.1)	3,479 (23.9)	914 (6.3)	10,194 (69.9)
Affect everyday activities	3,522 (22.3)	681 (4.3)	11,561 (73.3)	3,270 (22.4)	644 (4.4)	10,673 (73.2)
Medically significant enough to seek medical advice	558 (3.5)	1,272 (8.1)	13,934 (88.4)	502 (3.4)	1,259 (8.6)	12,846 (88.1)
Disabling	45 (0.3)	1,241 (7.9)	14,478 (91.8)	44 (0.3)	1,240 (8.5)	13,303 (91.2)

Hospitalization	84 (0.5)	1,223 (7.8)	14,457 (91.7)	79 (0.5)	1,222 (8.4)	13,286 (91.1)
Life-threatening	22 (0.1)	1,228 (7.8)	14,514 (92.1)	18 (0.1)	1,225 (8.4)	13,344 (91.5)
Congenital anomaly	8 (0.1)	1,219 (7.7)	14,537 (92.2)	8 (0.1)	1,217 (8.3)	13,362 (91.6)

Abbreviations: *ADR* Adverse Drug Reaction
