# Supplementary material for: Implementation and Results of Active Vaccine Safety Monitoring During the COVID-19 Pandemic in the UK: A Regulatory Perspective
Source: Drug Saf. 2025 Sep 3;48(12):1365–85. doi: 10.1007/s40264-025-01579-w (PMC12605443; doi:10.1007/s40264-025-01579-w)
Supplement: Supplementary file 5 — Supplementary file5 (PDF 550 KB) [file 40264_2025_1579_MOESM5_ESM.pdf]

Online Resource 5

Electronic Supplementary material

Article Title: Implementation and results of active vaccine safety monitoring during the COVID-19 pandemic in the UK: a regulatory perspective

Journal for Submission: Drug Safety (Springer Nature)

Authors: Jenny Wong, Katherine Donegan, Kendal Harrison, Tahira Jan, Alison Cave, and Phil Tregunno

Author Affiliation: Medicines and Healthcare products Regulatory Agency, London, UK

Corresponding Author: Phil Tregunno, phil.tregunno@mhra.gov.uk

MedDRA® Reaction Seriousness

Supplementary Table 8. Number of Individuals reporting and not reporting a MedDRA serious ADR following vaccination, stratified by demographics and vaccination dose reporting

	Individuals reporting a MedDRA serious ADR (% of the cohort reporting any vaccine dose)		Individuals reporting a MedDRA serious ADR after the 1st dose (% of the cohort reporting a 1st vaccine dose)		Individuals reporting a MedDRA serious ADR after the 2nd dose (% of the cohort reporting 1st and 2nd vaccine doses)		Individuals reporting a MedDRA serious ADR after the 3rd dose (% of the cohort reporting 1st, 2nd, and 3rd vaccine doses)	
**Sex**								
Male	1,387	(11.2)	978	(8.8)	182	(3.5)	118	(3.4)
Female	2,696	(15.4)	1,890	(11.9)	342	(4.8)	208	(4.8)
Unknown	51	(12.9)	31	(9.0)	7	(5.2)	3	(4.2)
**Age bands (years)**								
Under 12	3	(2.5)	3	(2.8)	0	(0.0)	0	(0.0)
12-17	111	(9.0)	86	(7.0)	15	(5.7)	5	(6.5)
18-29	250	(17.9)	178	(13.6)	33	(7.2)	13	(6.0)
30-39	498	(13.8)	357	(10.8)	69	(5.4)	16	(2.5)
40-49	581	(21.4)	462	(18.4)	52	(5.1)	37	(6.0)
50-59	731	(18.6)	539	(15.4)	68	(4.1)	72	(6.2)
60-69	893	(14.8)	600	(11.2)	126	(4.3)	98	(4.7)

	**Individuals reporting a MedDRA serious ADR (% of the cohort reporting any vaccine dose)**		**Individuals reporting a MedDRA serious ADR after the 1st dose (% of the cohort reporting a 1st vaccine dose)**		**Individuals reporting a MedDRA serious ADR after the 2nd dose (% of the cohort reporting 1st and 2nd vaccine doses)**		**Individuals reporting a MedDRA serious ADR after the 3rd dose (% of the cohort reporting 1st, 2nd, and 3rd vaccine doses)**	
70-79	910	(10.2)	576	(7.2)	141	(3.5)	83	(3.1)
80+	153	(6.6)	97	(4.6)	26	(3.2)	5	(1.2)
Unknown	4	(16.0)	1	(4.5)	1	(4.2)	0	(0.0)
**Ethnicity**								
White British, White Irish, or any other white background	3,592	(13.7)	2,522	(10.6)	477	(4.3)	298	(4.1)
Other	254	(14.4)	187	(11.5)	32	(5.7)	15	(4.8)
Unknown	288	(12.9)	190	(9.7)	22	(3.0)	16	(4.5)
**BMI Category**								
Underweight	107	(16.0)	70	(11.4)	19	(6.9)	14	(8.7)
Normal	1,304	(14.6)	907	(11.2)	193	(4.8)	101	(3.8)
Overweight	1,088	(13.3)	773	(10.4)	138	(3.8)	94	(4.0)
Obese	637	(14.4)	453	(11.3)	77	(4.3)	52	(4.5)
Unknown	998	(12.4)	696	(9.6)	104	(3.7)	68	(4.2)
**Immunocompromised**								
Yes	514	(14.8)	346	(11.0)	75	(5.2)	43	(4.6)
No/Unknown	3,620	(13.5)	2,553	(10.5)	456	(4.1)	286	(4.1)

Abbreviations: *ADR* Adverse Drug Reaction, *BMI* Body Mass Index, *MedDRA* Medical Dictionary for Regulatory Activities. Table Percentages: Proportion of Individuals who reported a MedDRA serious ADR in association with a dose of a COVID-19 vaccine, stratified by demographics and by the cohort definition. Example: Amongst the male individuals who had reported a 1st dose and a 2nd dose vaccination, 3.5% (n=182) had reported a MedDRA serious ADR occurring after the 2nd dose vaccination.
