# Supplementary material for: Implementation and Results of Active Vaccine Safety Monitoring During the COVID-19 Pandemic in the UK: A Regulatory Perspective
Source: Drug Saf. 2025 Sep 3;48(12):1365–85. doi: 10.1007/s40264-025-01579-w (PMC12605443; doi:10.1007/s40264-025-01579-w)
Supplement: Supplementary file 6 — Supplementary file6 (PDF 605 KB) [file 40264_2025_1579_MOESM6_ESM.pdf]

Online Resource 6

Electronic Supplementary material

Article Title: Implementation and results of active vaccine safety monitoring during the COVID-19 pandemic in the UK: a regulatory perspective

Journal for Submission: Drug Safety (Springer Nature)

Authors: Jenny Wong, Katherine Donegan, Kendal Harrison, Tahira Jan, Alison Cave, and Phil Tregunno

Author Affiliation: Medicines and Healthcare products Regulatory Agency, London, UK

Corresponding Author: Phil Tregunno, phil.tregunno@mhra.gov.uk

Commonly Reported Adverse Reactions (ADRs)

Supplementary Table 9. Most commonly reported ADRs (MedDRA® Preferred Term) by vaccine brand and dose (% of total ADRs reported for each dose/brand)

Vaccine Brand	Dose 1	Count (%)	Dose 2	Count (%)	Dose 3	Count (%)
**Pfizer**	Pain in extremity	1,059 (18.6)	Fatigue	230 (14.6)	Pain in extremity	235 (13.5)
	Fatigue	654 (11.5)	Pain in extremity	207 (13.1)	Headache	223 (12.9)
	Headache	618 (10.8)	Headache	166 (10.5)	Fatigue	186 (10.7)
	Myalgia	271 (4.8)	Pyrexia	74 (4.7)	Arthralgia	72 (4.1)

	Pain	217(3.8)	Myalgia	67 (4.3)	Myalgia	72 (4.1)
	Nausea	174 (3.0)	Pain	57 (3.6)	Pyrexia	69 (4.0)
	Arthralgia	174 (3.0)	Nausea	48 (3.0)	Chills	66 (3.8)
	Pyrexia	156 (2.7)	Arthralgia	47 (3.0)	Nausea	56 (3.2)
	Injection site pain	155 (2.7)	Limb discomfort	40 (2.5)	Pain	54 (3.1)
	Limb discomfort	150 (2.6)	Dizziness	33 (2.1)	Limb discomfort	38 (2.2)
	TOTAL ADR	***5,705***	***TOTAL ADR***	***1,576***	***TOTAL ADR***	***1,735***
**AstraZeneca**	Headache	3,063 (15.7)	Fatigue	359 (14.8)	Myalgia	2 (16.7)
	Fatigue	2,205 (11.3)	Headache	351 (14.4)	Pain in extremity	2 (16.7)
	Pyrexia	1,470 (7.5)	Pain in extremity	225 (9.2)	Fatigue	1 (8.3)
	Chills	1,235 (6.3)	Myalgia	123 (5.1)	Tenderness	1 (8.3)
	Pain in extremity	1,200 (6.1)	Arthralgia	91 (3.7)	Chills	1 (8.3)
	Myalgia	992 (5.1)	Pyrexia	90 (3.7)	Palpitations	1 (8.3)
	Nausea	789 (4.0)	Chills	74 (3.0)	Malaise	1 (8.3)
	Arthralgia	760 (3.9)	Nausea	71 (2.9)	Injection related reaction	1 (8.3)
	Pain	485 (2.5)	Dizziness	63 (2.6)	Body temp increased	1 (8.3)
	Influenza like illness	410 (2.1)	Limb discomfort	59 (2.4)	Arthralgia	1 (8.3)
	TOTAL ADR	***19,559***	***TOTAL ADR***	***2,433***	***TOTAL ADR***	***12***
**Moderna**	Pain in extremity	141 (22.6)	Pyrexia	39 (13.9)	Pain in extremity	155 (15.0)
	Fatigue	63 (10.1)	Fatigue	37 (13.2)	Fatigue	106 (10.3)
	Headache	57 (9.1)	Headache	28 (10.0)	Headache	106 (10.3)
	Pyrexia	33 (5.3)	Pain in extremity	17 (6.0)	Pyrexia	64 (6.2)
	Nausea	23 (3.7)	Chills	17 (6.0)	Chills	57 (5.5)
	Myalgia	21 (3.4)	Pain	15 (5.3)	Nausea	40 (3.9)
	Pain	20 (3.2)	Myalgia	11 (3.9)	Myalgia	40 (3.9)
	Arthralgia	16 (2.6)	Influenza	8 (2.8)	Arthralgia	37 (3.6)
	Rash	15 (2.4)	Arthralgia	8 (2.8)	Influenza like illness	25 (2.4)
	Injection site pain	15 (2.4)	Nausea	6 (2.1)	Limb discomfort	24 (2.2)
	TOTAL ADR	***623***	***TOTAL ADR***	***281***	***TOTAL ADR***	***1,034***

Abbreviations: *ADR* Adverse Drug Reaction, *MedDRA* Medical Dictionary for Regulatory Activities.
