# Supplementary material for: Implementation and Results of Active Vaccine Safety Monitoring During the COVID-19 Pandemic in the UK: A Regulatory Perspective
Source: Drug Saf. 2025 Sep 3;48(12):1365–85. doi: 10.1007/s40264-025-01579-w (PMC12605443; doi:10.1007/s40264-025-01579-w)
Supplement: Supplementary file 7 — Supplementary file7 (PDF 555 KB) [file 40264_2025_1579_MOESM7_ESM.pdf]

Online Resource 7

Electronic Supplementary material

Article Title: Implementation and results of active vaccine safety monitoring during the COVID-19 pandemic in the UK: a regulatory perspective

Journal for Submission: Drug Safety (Springer Nature)

Authors: Jenny Wong, Katherine Donegan, Kendal Harrison, Tahira Jan, Alison Cave, and Phil Tregunno

Author Affiliation: Medicines and Healthcare products Regulatory Agency, London, UK

Corresponding Author: Phil Tregunno, phil.tregunno@mhra.gov.uk

Adverse Reactions (ADRs) Reported in association with a COVID-19 Vaccination for Any Dose and by Dose Sequence for each Vaccine Brand.

Proportion of ADRs by MedDRA SOC level by vaccine brand and dose sequence

In this supplementary material, an overview of the proportion of events by SOC level by dose sequence is presented for each vaccine brand. Of all the adverse reactions reported, the majority of events belonged to three MedDRA system organ class (SOC) categories, the General Disorders and Administration Site Conditions SOC (35.50%, n=12,656), followed by Musculoskeletal and Connective Tissue Disorders (22.22%, n=7,921) and then Nervous System Disorders (20.61%, n=7,347) (Supplementary Figure 1 and Supplementary Table 10).

A similar trend in SOC event reporting was reflected in the dose specific analysis and the vaccine brand-specific analyses. In the dose-specific analyses, the top 3 most frequently reported SOCs remained the same. There were differences in the proportions of events in these SOC groups across the doses, however the use of branded vaccines differed over time and as a result impacted the dose patterns for each brand.

The most SOC categories with the most ADRs reported were similar to what was seen in the SOC level analysis for the overall cohort. The Nervous system SOC, Musculoskeletal and connective tissue conditions SOC and General disorders and Administration site conditions SOC remained the most reported SOC for each of the vaccine brands. For the Pfizer vaccine in Supplementary Figure 2 and Supplementary Table 11, the reporting of events in the Nervous system SOC remained consistent for each dose (17.4-17.8%). There was a higher proportion of reporting of musculoskeletal and connective tissue conditions with the 1st dose (30.9%) compared to the 2nd and 3rd doses (25.4% and 26.9%), whereas there was a higher proportion of reporting with 2nd dose for general disorders and administration site conditions (36.7%) compared to 1st and 3rd doses (29.5% vs 34.5%).

In the data presented for AstraZeneca (Supplementary Figure 3 and Supplementary Table 12, there was no events reported in the Nervous system SOC for the 3rd dose, but the proportion of reporting remained consistent for 1st and 2nd doses (22.3% vs 22.4%). For general or administration site events, the 1st dose saw a larger proportion of reporting compared to the 2nd and 3rd doses (37.4% vs 33.5% vs 33.3%). However, in contrast, the proportionate reporting of musculoskeletal events increased across the doses with an increase from 18.7% with 1st dose to 41.7% with 3rd dose. A similar increase in reporting was seen in the “Other SOC grouped” category (5.9% to 25.0%).

For the Moderna COVID-19 vaccine (Supplementary Figure 4 and Supplementary Table 13), the proportionate reporting in the Nervous system SOC were similar across the 3 doses (15.1% vs 16.7% vs 15.7%). In the Musculoskeletal and connective tissue SOC, the proportion of events reported with the 1st dose was larger than that reported for the other two doses (32.7% vs 18.1% vs 26.9%). In contrast, in the General and administration site disorders SOC, the proportion of events reported was the highest with the 2nd dose at 45.9%, followed by the 3rd dose at 37.1% and then the lowest at 30.3% with the 1st dose vaccination.

Supplementary Figure 5 and Supplementary Table 14 presents the distribution of reporting by SOC level for the other remaining vaccines or when the vaccine brand was unknown.

See ‘Online Resources 8’ for tables presenting the full listing of ADRs reported by MedDRA SOC, HLGT, HLT and PT level terms for each brand and dose.

SUPPLEMENTARY FIGURE 1. ALL COVID-19 VACCINES: TYPES OF ADR REPORTING BY SOC LEVEL

For ADRs reported for any dose, ADRs reported for 1st dose, for 2nd dose and 3rd doses.

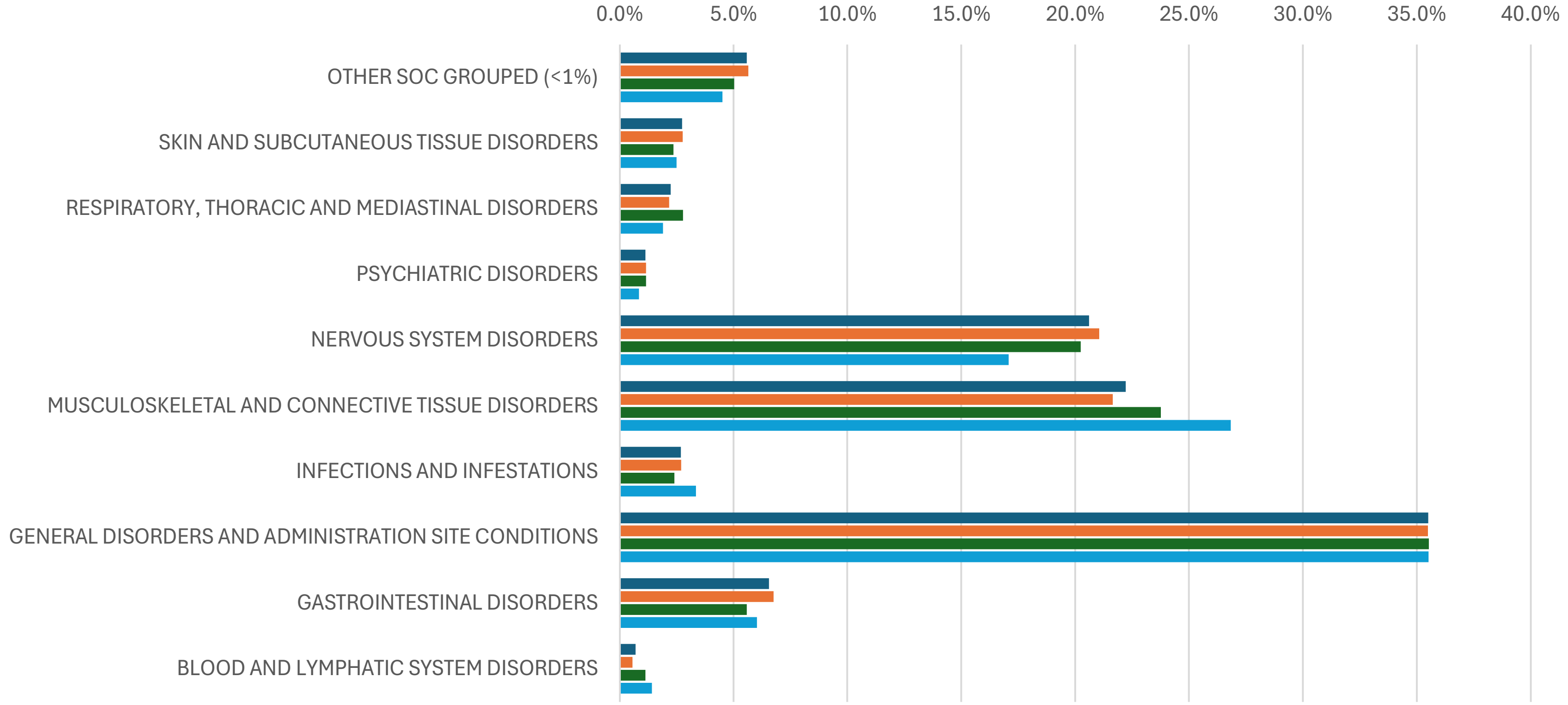

	BLOOD AND LYMPHATIC SYSTEM DISORDERS	GASTROINTESTINAL DISORDERS	GENERAL DISORDERS AND ADMINISTRATION SITE CONDITIONS	INFECTIONS AND INFESTATIONS	MUSCULOSKELETAL AND CONNECTIVE TISSUE DISORDERS	NERVOUS SYSTEM DISORDERS	PSYCHIATRIC DISORDERS	RESPIRATORY, THORACIC AND MEDIASTINAL DISORDERS	SKIN AND SUBCUTANEOUS TISSUE DISORDERS	OTHER SOC GROUPED (<1%)
All doses	0.7%	6.6%	35.5%	2.7%	22.2%	20.6%	1.1%	2.2%	2.7%	5.6%
Dose 1	0.6%	6.8%	35.5%	2.7%	21.7%	21.1%	1.2%	2.2%	2.8%	5.7%
Dose 2	1.1%	5.6%	35.5%	2.4%	23.8%	20.2%	1.2%	2.8%	2.4%	5.0%
Dose 3	1.4%	6.0%	35.5%	3.4%	26.8%	17.1%	0.8%	1.9%	2.5%	4.5%

Supplementary Table 10. Overall COVID-19 vaccines: Data table for Supplementary Figure 1.

ADRs reported (MedDRA SOC Level)	BLOOD & LYMPHATIC SYSTEM DISORDERS	GASTRO-INTESTINAL DISORDERS	GENERAL DISORDERS & ADMINISTRATION SITE CONDITIONS	INFECTIONS & INFESTATIONS	MUSCULOSKELETAL & CONNECTIVE TISSUE DISORDERS	NERVOUS SYSTEM DISORDERS	PSYCHIATRIC DISORDERS	RESPIRATORY, THORACIC & MEDIASTINAL DISORDERS	SKIN & SUBCUTANEOUS TISSUE DISORDERS	OTHER SOC GROUPED (<1%)
All doses (n=35,647)	251 (0.7)	2,339 (6.6)	12,656 (35.5)	961 (2.7)	7,921 (22.2)	7,347 (20.6)	405 (1.1)	799 (2.2)	976 (2.7)	1,992 (5.6)
Dose 1 (n=26,096)	148 (0.6)	1,764 (6.8)	9,263 (35.5)	707 (2.7)	5,651 (21.7)	5,496 (21.1)	303 (1.2)	566 (2.2)	723 (2.8)	1,475 (5.7)
Dose 2 (n=4,317)	49 (1.1)	241 (5.6)	1,534 (35.5)	104 (2.4)	1,026 (23.8)	874 (20.2)	50 (1.2)	120 (2.8)	102 (2.4)	217 (5.0)
Dose3 (n=2,833)	40 (1.4)	171 (6.0)	1,006 (35.5)	95 (3.4)	760 (26.8)	484 (17.1)	24 (0.8)	54 (1.9)	71 (2.5)	128 (4.5)

Abbreviations: *ADR* Adverse Drug Reaction, *MedDRA* Medical Dictionary for Regulatory Activities, *SOC* System Organ Class.

Percentages reflect the proportion of ADRs by SOC level reported for each dose.

SUPPLEMENTARY FIGURE 2. PFIZER BIONTECH VACCINE: TYPES OF ADR REPORTING BY SOC LEVEL

For ADRs reported for any dose, ADRs reported for 1st dose, for 2nd dose and 3rd doses.

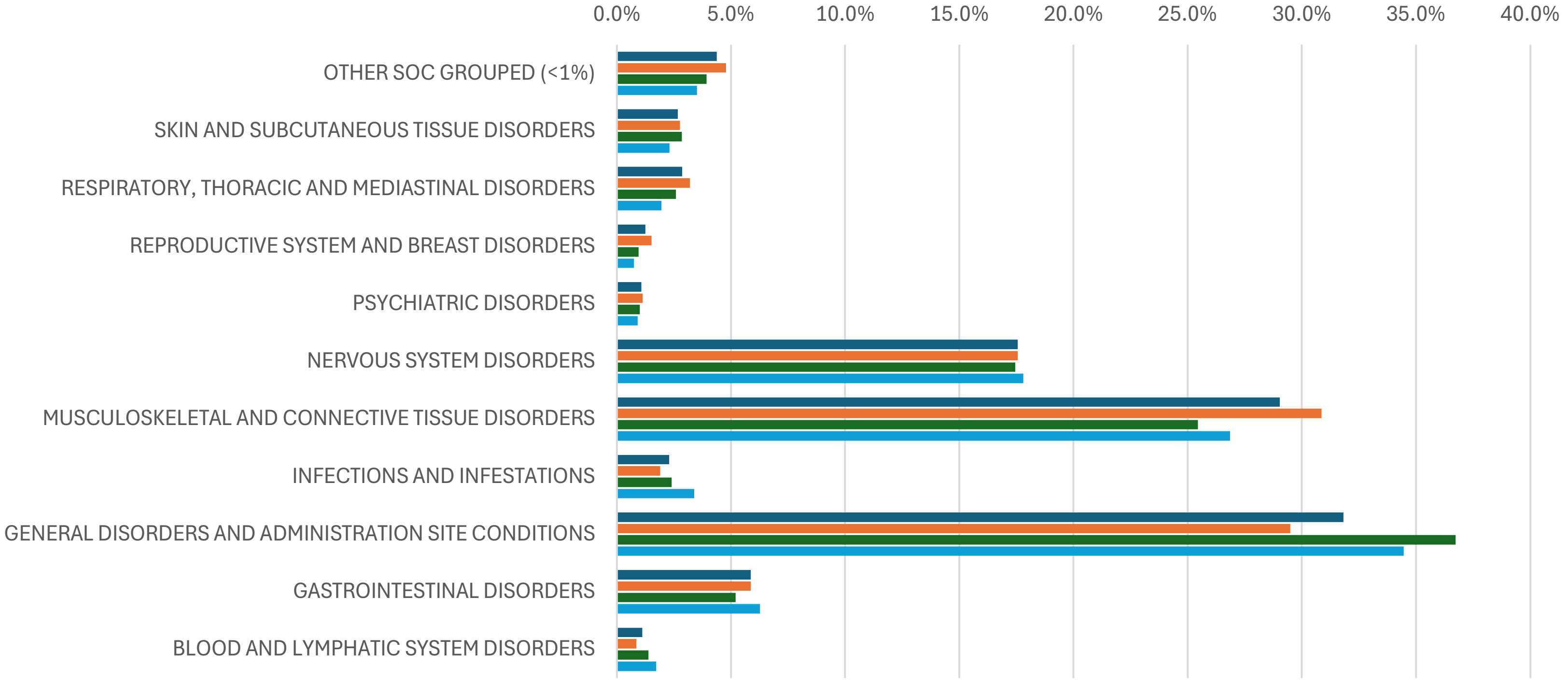

	BLOOD AND LYMPHATIC SYSTEM DISORDERS	GASTROINTESTINAL DISORDERS	GENERAL DISORDERS AND ADMINISTRATION SITE CONDITIONS	INFECTIONS AND INFESTATIONS	MUSCULOSKELETAL AND CONNECTIVE TISSUE DISORDERS	NERVOUS SYSTEM DISORDERS	PSYCHIATRIC DISORDERS	REPRODUCTIVE SYSTEM AND BREAST DISORDERS	RESPIRATORY, THORACIC AND MEDIASTINAL DISORDERS	SKIN AND SUBCUTANEOUS TISSUE DISORDERS	OTHER SOC GROUPED (<1%)
All doses	1.1%	5.9%	31.8%	2.3%	29.0%	17.6%	1.1%	1.3%	2.9%	2.7%	4.4%
Dose 1	0.9%	5.9%	29.5%	1.9%	30.9%	17.6%	1.1%	1.5%	3.2%	2.8%	4.8%
Dose 2	1.4%	5.2%	36.7%	2.4%	25.4%	17.4%	1.0%	1.0%	2.6%	2.9%	3.9%
Dose 3	1.7%	6.3%	34.5%	3.4%	26.9%	17.8%	0.9%	0.7%	2.0%	2.3%	3.5%

Supplementary Table 11. Pfizer BioNTech vaccine: Data table for Supplementary Figure 2

**ADRs reported (MedDRA SOC Level)**	BLOOD AND LYMPHATIC SYSTEM DISORDERS	GASTRO-INTESTINAL DISORDERS	GENERAL DISORDERS & ADMINISTRATION SITE CONDITIONS	INFECTIONS AND INFESTATIONS	MUSCULOSKELETAL & CONNECTIVE TISSUE DISORDERS	NERVOUS SYSTEM DISORDERS	PSYCHIATRIC DISORDERS	REPRODUCTIVE SYSTEM & BREAST DISORDERS	RESPIRATORY, THORACIC & MEDIASTINAL DISORDERS	SKIN & SUBCUTANEOUS TISSUE DISORDERS	OTHER SOC GROUPED (<1%)
All doses (n=9,139)	102 (1.1)	537 (5.9)	2,909 (31.8)	210 (2.3)	2,654 (29.0)	1,605 (17.6)	99 (1.1)	115 (1.3)	262 (2.9)	245 (2.7)	401 (4.4)
Dose 1 (n=5,705)	49 (0.9)	335 (5.9)	1,683 (29.5)	109 (1.9)	1,761 (30.9)	1,002 (17.6)	65 (1.1)	87 (1.5)	183 (3.2)	158 (2.8)	273 (4.8)
Dose 2 (n=1,576)	22 (1.4)	82 (5.2)	579 (36.7)	38 (2.4)	401 (25.4)	275 (17.4)	16 (1.0)	15 (1.0)	41 (2.6)	45 (2.9)	62 (3.9)
Dose3 (n=1,735)	30 (1.7)	109 (5.2)	598 (34.5)	59 (3.4)	466 (26.9)	309 (17.8)	16 (0.9)	13 (0.7)	34 (2.0)	40 (2.3)	61 (3.5)

Abbreviations: *ADR* Adverse Drug Reaction, *MedDRA* Medical Dictionary for Regulatory Activities, *SOC* System Organ Class.

Percentages reflect the proportion of ADRs by SOC level reported for each dose.

SUPPLEMENTARY FIGURE 3. ASTRAZENECA VACCINE: TYPES OF ADR REPORTING BY SOC LEVEL

For ADRs reported for any dose, ADRs reported for 1st dose, for 2nd dose and 3rd doses.

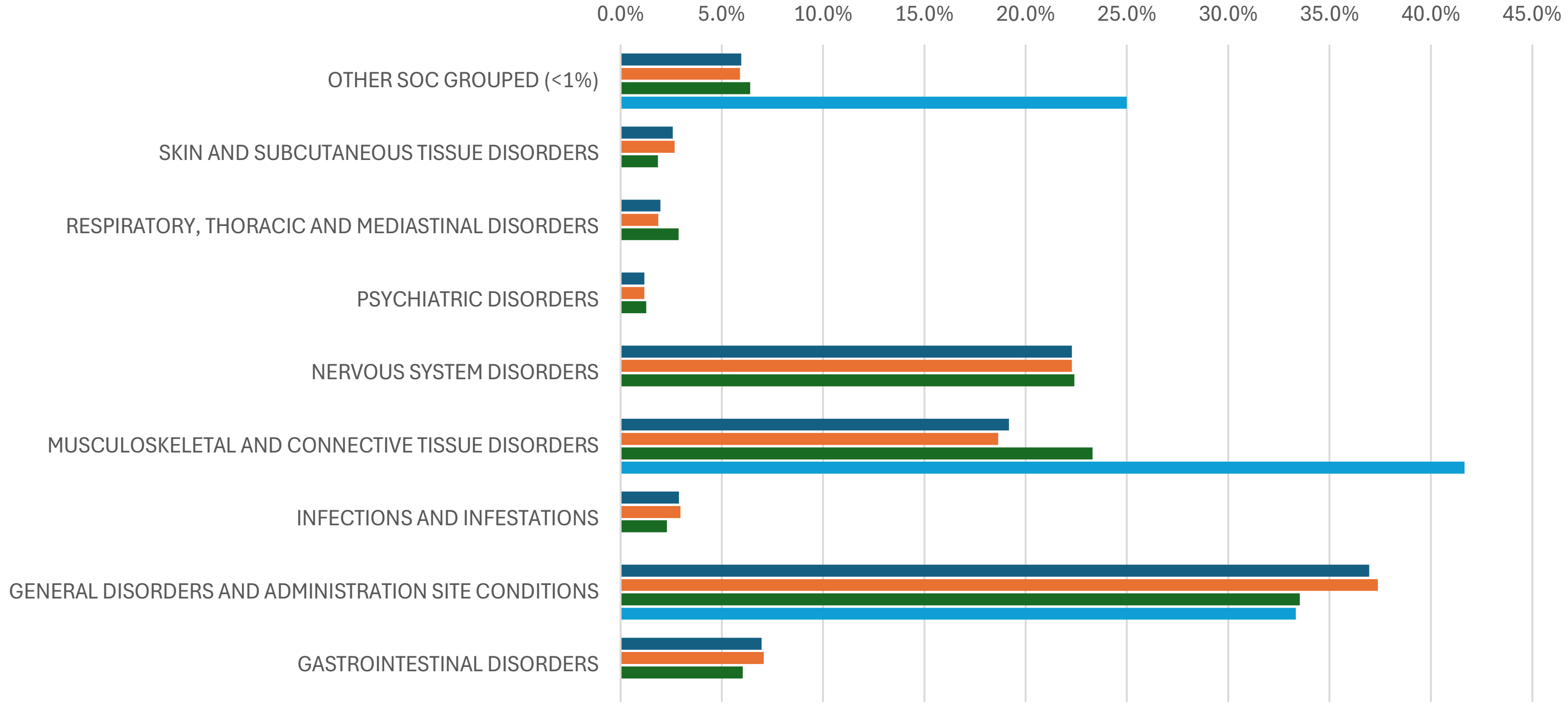

	GASTROINTESTINAL DISORDERS	GENERAL DISORDERS AND ADMINISTRATION SITE CONDITIONS	INFECTIONS AND INFESTATIONS	MUSCULOSKELETAL AND CONNECTIVE TISSUE DISORDERS	NERVOUS SYSTEM DISORDERS	PSYCHIATRIC DISORDERS	RESPIRATORY, THORACIC AND MEDIASTINAL DISORDERS	SKIN AND SUBCUTANEOUS TISSUE DISORDERS	OTHER SOC GROUPED (<1%)
All doses	7.0%	37.0%	2.9%	19.2%	22.3%	1.2%	2.0%	2.6%	6.0%
Dose 1	7.1%	37.4%	3.0%	18.7%	22.3%	1.2%	1.9%	2.7%	5.9%
Dose 2	6.0%	33.5%	2.3%	23.3%	22.4%	1.3%	2.9%	1.8%	6.4%
Dose 3	0.0%	33.3%	0.0%	41.7%	0.0%	0.0%	0.0%	0.0%	25.0%

Supplementary Table 12. AstraZeneca vaccine: Data table for Supplementary Figure 3

**ADRs reported (MedDRA SOC Level)**	GASTROINTESTINAL DISORDERS	GENERAL DISORDERS & ADMINISTRATION SITE CONDITIONS	INFECTIONS & INFESTATIONS	MUSCULOSKELETAL & CONNECTIVE TISSUE DISORDERS	NERVOUS SYSTEM DISORDERS	PSYCHIATRIC DISORDERS	RESPIRATORY, THORACIC & MEDIASTINAL DISORDERS	SKIN & SUBCUTANEOUS TISSUE DISORDERS	OTHER SOC GROUPED (<1%)
All doses (n=22,004)	1,532 (7.0)	8,132 (37.0)	635 (2.9)	4,220 (19.2)	4,904 (22.3)	262 (1.2)	435 (2.0)	570 (2.6)	1,314 (6.0)
Dose 1 (n=19,559)	1,385 (7.1)	7,312 (37.4)	579 (3.0)	3,648 (18.7)	4,359 (22.3)	231 (1.2)	365 (1.9)	525 (2.7)	1,155 (5.9)
Dose 2 (n=2,433)	147 (6.0)	816 (33.5)	56 (2.3)	567 (23.3)	545 (22.4)	31 (1.3)	70 (2.9)	45 (1.8)	156 (6.4)
Dose3 (n=12)	0 (0.0)	4 (33.3)	0 (0.0)	5 (41.7)	0 (0.0)	0 (0.0)	0 (0.0)	0 (0.0)	3 (25.0)

Abbreviations: *ADR* Adverse Drug Reaction, *MedDRA* Medical Dictionary for Regulatory Activities, *SOC* System Organ Class.

Percentages reflect the proportion of ADRs by SOC level reported for each dose.

SUPPLEMENTARY FIGURE 4. MODERNA VACCINE: TYPES OF ADR REPORTING BY SOC LEVEL

For ADRs reported for any dose, ADRs reported for 1st dose, for 2nd dose and 3rd doses.

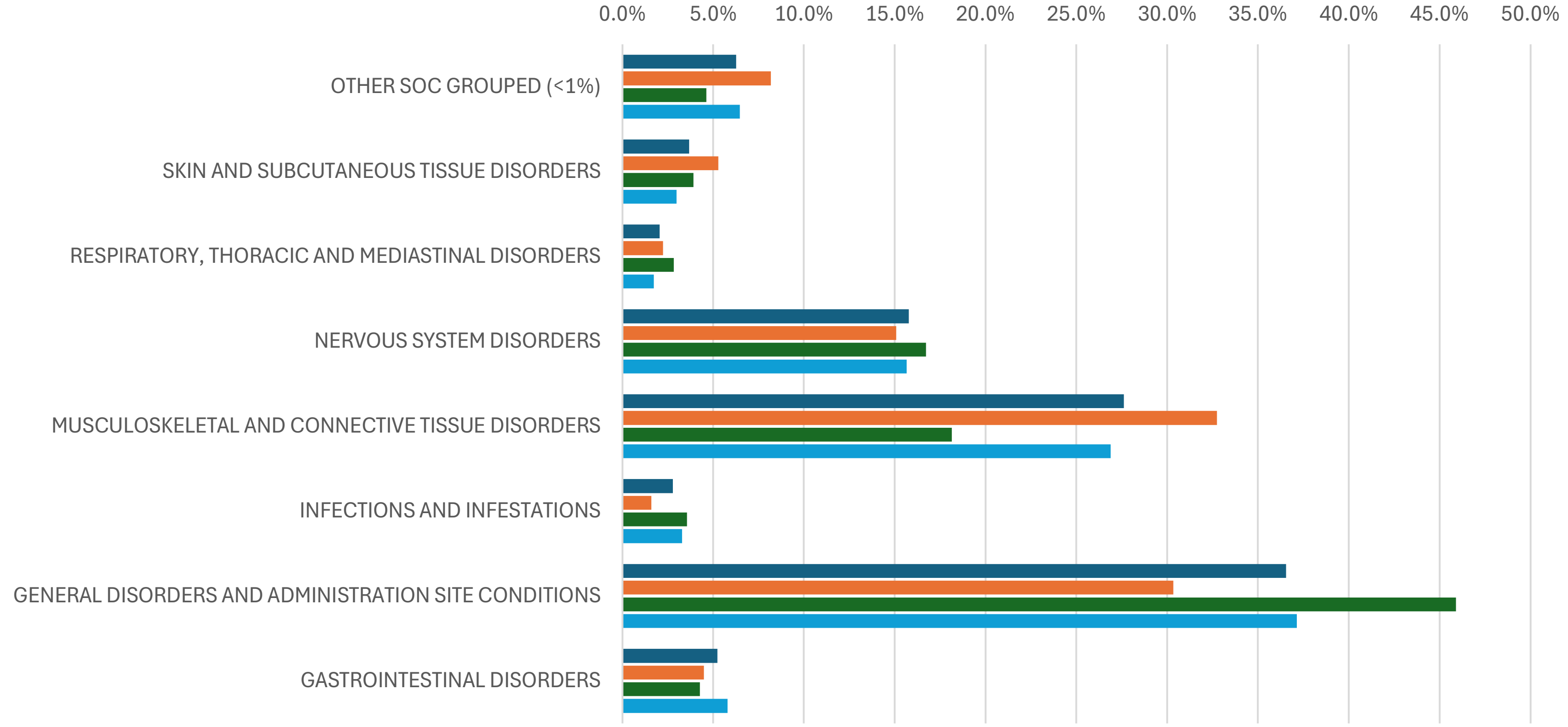

	GASTROINTESTINAL DISORDERS	GENERAL DISORDERS AND ADMINISTRATION SITE CONDITIONS	INFECTIONS AND INFESTATIONS	MUSCULOSKELETAL AND CONNECTIVE TISSUE DISORDERS	NERVOUS SYSTEM DISORDERS	RESPIRATORY, THORACIC AND MEDIASTINAL DISORDERS	SKIN AND SUBCUTANEOUS TISSUE DISORDERS	OTHER SOC GROUPED (<1%)
All doses	5.2%	36.5%	2.8%	27.6%	15.8%	2.1%	3.7%	6.3%
Dose 1	4.5%	30.3%	1.6%	32.7%	15.1%	2.2%	5.3%	8.2%
Dose 2	4.3%	45.9%	3.6%	18.1%	16.7%	2.8%	3.9%	4.6%
Dose 3	5.8%	37.1%	3.3%	26.9%	15.7%	1.7%	3.0%	6.5%

Supplementary Table 13. Moderna vaccine: Data table for Supplementary Figure 4

**ADRs reported (MedDRA SOC Level)**	GASTROINTESTINA L DISORDERS	GENERAL DISORDERS & ADMINISTRATION SITE CONDITIONS	INFECTIONS & INFESTATIONS	MUSCULOSKELETA L & CONNECTIVE TISSUE DISORDERS	NERVOUS SYSTEM DISORDERS	RESPIRATORY, THORACIC & MEDIASTINAL DISORDERS	SKIN & SUBCUTANEOUS TISSUE DISORDERS	OTHER SOC GROUPED (<1%)
All doses (n=2,230)	117 (5.2)	815 (36.5)	62 (2.8)	616 (27.6)	352 (15.8)	46 (2.1)	82 (3.7)	140 (6.3)
Dose 1 (n=623)	28 (4.5)	189 (30.3)	10 (1.6)	204 (32.7)	94 (15.1)	14 (2.2)	33 (5.3)	51 (8.2)
Dose 2 (n=281)	12 (4.3)	129 (45.9)	10 (3.6)	51 (18.1)	47 (16.7)	8 (2.8)	11 (3.9)	13 (4.6)
Dose3 (n=1034)	60 (5.8)	384 (37.1)	34 (3.3)	278 (26.9)	162 (15.7)	18 (1.7)	31 (3.0)	67 (6.5)

Abbreviations: *ADR* Adverse Drug Reaction, *MedDRA* Medical Dictionary for Regulatory Activities, *SOC* System Organ Class.

Percentages reflect the proportion of ADRs by SOC level reported for each dose.

SUPPLEMENTARY FIGURE 5. OTHER OR UNKNOWN BRANDED VACCINE: TYPES OF ADR REPORTING BY SOC LEVEL

For ADRs reported for any dose, ADRs reported for 1st dose, for 2nd dose and 3rd doses.

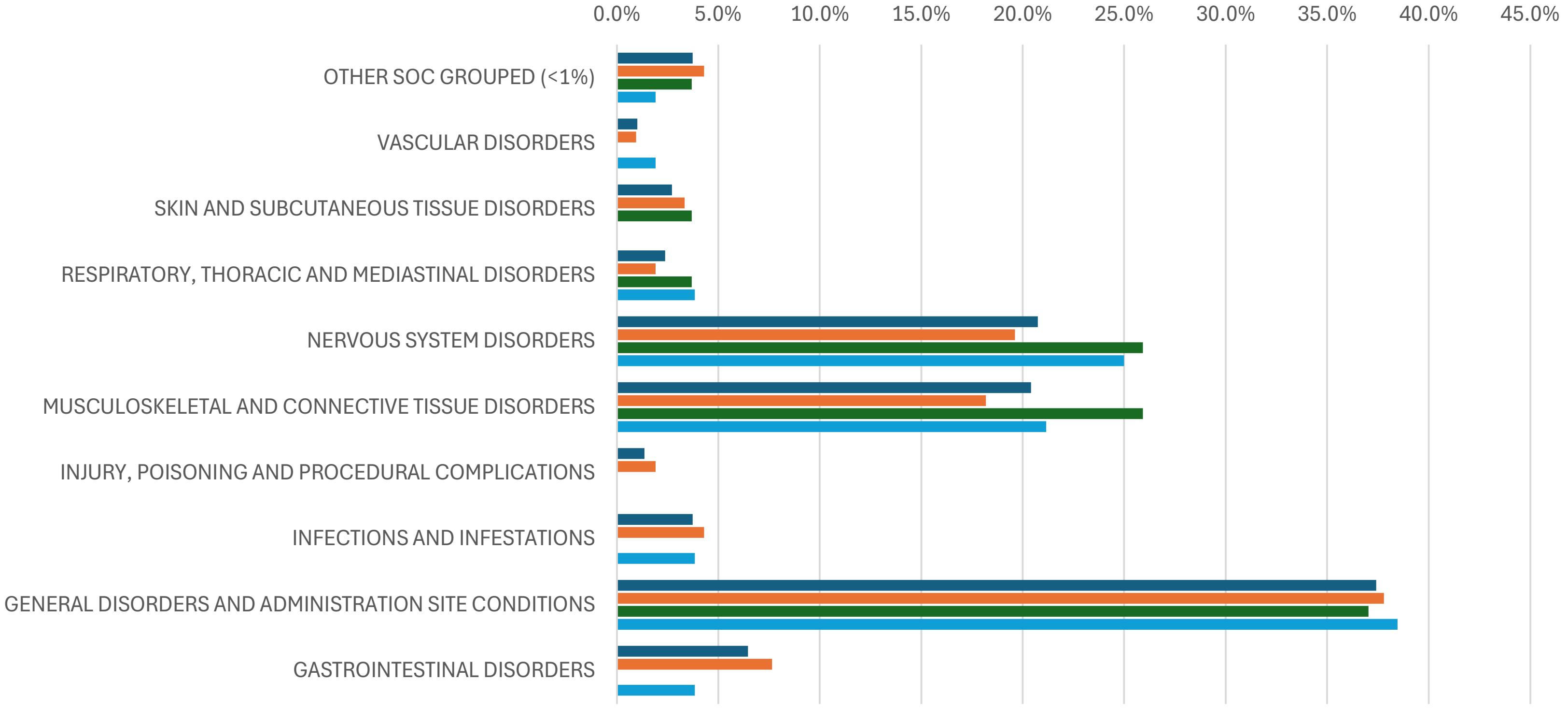

	GASTROINTESTINAL DISORDERS	GENERAL DISORDERS AND ADMINISTRATION SITE CONDITIONS	INFECTIONS AND INFESTATIONS	INJURY, POISONING AND PROCEDURAL COMPLICATIONS	MUSCULOSKELETAL AND CONNECTIVE TISSUE DISORDERS	NERVOUS SYSTEM DISORDERS	RESPIRATORY, THORACIC AND MEDIASTINAL DISORDERS	SKIN AND SUBCUTANEOUS TISSUE DISORDERS	VASCULAR DISORDERS	OTHER SOC GROUPED (<1%)
All doses	6.5%	37.4%	3.7%	1.4%	20.4%	20.7%	2.4%	2.7%	1.0%	3.7%
Dose 1	7.7%	37.8%	4.3%	1.9%	18.2%	19.6%	1.9%	3.3%	1.0%	4.3%
Dose 2	0.0%	37.0%	0.0%	0.0%	25.9%	25.9%	3.7%	3.7%	0.0%	3.7%
Dose 3	3.8%	38.5%	3.8%	0.0%	21.2%	25.0%	3.8%	0.0%	1.9%	1.9%

Supplementary Table 14. Other or unknown branded vaccine: Data table for Supplementary Figure 5

**ADRs reported (MedDRA SOC Level)**	GASTROINTESTINAL DISORDERS	GENERAL DISORDERS & ADMINISTRATION SITE CONDITIONS	INFECTIONS & INFESTATIONS	INJURY, POISONING AND PROCEDURAL COMPLICATIONS	MUSCULOSKELETAL & CONNECTIVE TISSUE DISORDERS	NERVOUS SYSTEM DISORDERS	RESPIRATORY, THORACIC & MEDIASTINAL DISORDERS	SKIN & SUBCUTANEOUS TISSUE DISORDERS	VASCULAR DISORDERS	OTHER SOC GROUPED (<1%)
All doses (n=294)	19 (6.5)	110 (37.4)	11 (3.7)	4 (1.4)	60 (20.4)	61 (20.7)	7 (2.4)	8 (2.7)	3 (1.0)	11 (3.7)
Dose 1 (n=209)	16 (7.7)	79 (37.8)	9 (4.3)	4 (1.9)	38 (18.2)	41 (19.6)	4 (1.9)	7 (3.3)	2 (1.0)	9 (4.3)
Dose 2 (n=27)	0 (0.0)	10 (37.0)	0 (0.0)	0 (0.0)	7 (25.9)	7 (25.9)	1 (3.7)	1 (3.7)	0 (0.0)	1 (3.7)
Dose3 (n=52)	2 (3.8)	20 (38.5)	2 (3.8)	0 (0.0)	11 (21.2)	13 (25.0)	2 (3.8)	0 (0.0)	1 (1.9)	1 (1.9)

Abbreviations: *ADR* Adverse Drug Reaction, *MedDRA* Medical Dictionary for Regulatory Activities, *SOC* System Organ Class.

Percentages reflect the proportion of ADRs by SOC level reported for each dose.
