# Supplementary material for: Implementation and Results of Active Vaccine Safety Monitoring During the COVID-19 Pandemic in the UK: A Regulatory Perspective
Source: Drug Saf. 2025 Sep 3;48(12):1365–85. doi: 10.1007/s40264-025-01579-w (PMC12605443; doi:10.1007/s40264-025-01579-w)
Supplement: Supplementary file 8 — Supplementary file8 (PDF 548 KB) [file 40264_2025_1579_MOESM8_ESM.pdf]

Online Resource 8

Electronic Supplementary material

Article Title: Implementation and results of active vaccine safety monitoring during the COVID-19 pandemic in the UK: a regulatory perspective

Journal for Submission: Drug Safety (Springer Nature)

Authors: Jenny Wong, Katherine Donegan, Kendal Harrison, Tahira Jan, Alison Cave, and Phil Tregunno

Author Affiliation: Medicines and Healthcare products Regulatory Agency, London, UK

Corresponding Author: Phil Tregunno, phil.tregunno@mhra.gov.uk

Adverse Reactions (ADRs) Reported in association with a COVID-19 Vaccination for Any Dose and by Dose Sequence for each Vaccine Brand.

Total ADRs reported

A description of the tables is presented below. Each of these tables capture the full listing of ADRs reported by MedDRA SOC, HLGT, HLT and PT level terms for each brand and dose.

**Supplementary Table 15:**	ADR listing for reactions reported in the YCVM in (a) those reporting any vaccination dose and (b) in those who had reported a 1st dose vaccination and any subsequent doses.
**Supplementary Table 16:**	Pfizer BioNTech COVID-19 vaccine: ADR listing for reactions reported in the YCVM in (a) those reporting any vaccination dose and (b) in those who had reported a 1st dose vaccination and any subsequent doses.
**Supplementary Table 17:**	AstraZeneca COVID-19 vaccine: ADR listing for reactions reported in the YCVM in (a) those reporting any vaccination dose and (b) in those who had reported a 1st dose vaccination and any subsequent doses.
**Supplementary Table 18:**	Moderna COVID-19 vaccine: ADR listing for reactions reported in the YCVM in (a) those reporting any vaccination dose and (b) in those who had reported a 1st dose vaccination and any subsequent doses.

**Supplementary Table 19:**	Others/Unknown COVID-19 vaccine: ADR listing for reactions reported in the YCVM in (a) those reporting any vaccination dose and (b) in those who had reported a 1st dose vaccination and any subsequent doses.
**Supplementary Table 20**	COVID-19 vaccine ADR listing for reactions reported in the YCVM with no dose identification information in (a) those reporting any vaccination dose and (b) in those who had reported a 1st dose vaccination and any subsequent doses.

Abbreviations

ADR	Adverse drug reaction
MedDRA	Medical Dictionary for Regulatory Activities
SOC	System Organ Class
HLGT	High-Level Group Term
HLT	High-Level Term
PT	Preferred Term

SUPPLEMENTARY TABLE 15. ADR listing for events reported in the YCVM in (a) those reporting any vaccination dose and (b) in those who had reported a 1st dose vaccination and any

	Individuals with any vaccination dose: ADR Counts						Individuals reporting a 1st dose: ADR Counts					
**MEDDRA REACTION TERM (SOC, *HLGT*, *HLT*, PT)**	All doses	1st dose	2nd dose	3rd dose	Other doses	Unknown	All doses	1st dose	2nd dose	3rd dose	Other doses	Unknown
(freetext)	70	65	2	0	0	3	69	65	1	0	0	3
**BLOOD AND LYMPHATIC SYSTEM DISORDERS**												
COAGULOPATHIES AND BLEEDING DIATHESES (EXCL THROMBOCYTOPENIC)												
BLEEDING TENDENCIES												
INCREASED TENDENCY TO BRUISE	2	0	1	0	0	1	2	0	1	0	0	1
HAEMOGLOBINOPATHIES												
SICKLE CELL TRAIT AND DISORDERS												
SICKLE CELL ANAEMIA WITH CRISIS	1	1	0	0	0	0	1	1	0	0	0	0
PLATELET DISORDERS												
THROMBOCYTOPENIAS												
IMMUNE THROMBOCYTOPENIA	1	0	1	0	0	0	0	0	0	0	0	0
SPLEEN, LYMPHATIC AND RETICULOENDOTHELIAL SYSTEM DISORDERS												
LYMPHATIC SYSTEM DISORDERS NEC												
LYMPH NODE PAIN	39	15	12	8	0	4	34	15	10	5	0	4
LYMPHADENITIS	3	1	1	1	0	0	3	1	1	1	0	0
LYMPHADENOPATHY	234	131	52	41	1	9	211	131	37	34	1	8
**CARDIAC DISORDERS**												
CARDIAC ARRHYTHMIAS												
RATE AND RHYTHM DISORDERS NEC												
ARRHYTHMIA	1	1	0	0	0	0	1	1	0	0	0	0
CARDIAC FLUTTER	12	4	5	3	0	0	9	4	3	2	0	0
EXTRASYSTOLES	2	0	1	1	0	0	2	0	1	1	0	0
TACHYCARDIA	16	14	2	0	0	0	14	14	0	0	0	0
SUPRAVENTRICULAR ARRHYTHMIAS												
ATRIAL FIBRILLATION	4	2	1	1	0	0	2	2	0	0	0	0
SUPRAVENTRICULAR TACHYCARDIA	1	1	0	0	0	0	1	1	0	0	0	0
CARDIAC DISORDERS, SIGNS AND SYMPTOMS NEC												

CARDIAC DISORDERS NEC												
CARDIOVASCULAR DISORDER	1	1	0	0	0	0	1	1	0	0	0	0
CARDIAC SIGNS AND SYMPTOMS NEC												
PALPITATIONS	100	61	17	11	1	10	92	61	12	10	1	8
CORONARY ARTERY DISORDERS												
ISCHAEMIC CORONARY ARTERY DISORDERS												
ANGINA PECTORIS	2	2	0	0	0	0	2	2	0	0	0	0
MYOCARDIAL INFARCTION	2	2	0	0	0	0	2	2	0	0	0	0
HEART FAILURES												
HEART FAILURES NEC												
CARDIAC FAILURE	1	0	0	1	0	0	0	0	0	0	0	0
MYOCARDIAL DISORDERS												
NONINFECTIOUS MYOCARDITIS												
MYOCARDITIS	5	1	1	0	1	2	4	1	0	0	1	2
PERICARDIAL DISORDERS												
NONINFECTIOUS PERICARDITIS												
PERICARDITIS	1	1	0	0	0	0	1	1	0	0	0	0
**CONGENITAL, FAMILIAL AND GENETIC DISORDERS**												
CARDIAC AND VASCULAR DISORDERS CONGENITAL												
CARDIAC DISORDERS CONGENITAL NEC												
HEART DISEASE CONGENITAL	1	0	0	1	0	0	1	0	0	1	0	0
METABOLIC AND NUTRITIONAL DISORDERS CONGENITAL												
INBORN ERRORS OF AMINO ACID METABOLISM												
HYPERGLYCINAEMIA	1	1	0	0	0	0	1	1	0	0	0	0
INBORN ERRORS OF STEROID SYNTHESIS												
11-BETA-HYDROXYLASE DEFICIENCY	1	1	0	0	0	0	1	1	0	0	0	0
NEUROLOGICAL DISORDERS CONGENITAL												
PERIPHERAL NERVOUS SYSTEM DISORDERS CONGENITAL NEC												
PAROXYSMAL EXTREME PAIN DISORDER	1	0	1	0	0	0	1	0	1	0	0	0
**EAR AND LABYRINTH DISORDERS**												
AURAL DISORDERS NEC												
EAR DISORDERS NEC												
EAR DISCOMFORT	4	2	2	0	0	0	4	2	2	0	0	0

EAR DISORDER	1	0	1	0	0	0	1	0	1	0	0	0
EAR PAIN	49	37	9	1	0	2	44	37	5	0	0	2
EAR SWELLING	1	1	0	0	0	0	1	1	0	0	0	0
EXTERNAL EAR DISORDERS (EXCL CONGENITAL)												
EXTERNAL EAR DISORDERS NEC												
EXCESSIVE CERUMEN PRODUCTION	2	2	0	0	0	0	2	2	0	0	0	0
HEARING DISORDERS												
HEARING LOSSES												
DEAFNESS	5	3	2	0	0	0	4	3	1	0	0	0
DEAFNESS NEUROSENSORY	1	1	0	0	0	0	1	1	0	0	0	0
DEAFNESS PERMANENT	1	0	1	0	0	0	0	0	0	0	0	0
DEAFNESS UNILATERAL	2	1	1	0	0	0	2	1	1	0	0	0
HYPOACUSIS	3	2	1	0	0	0	3	2	1	0	0	0
SUDDEN HEARING LOSS	1	1	0	0	0	0	1	1	0	0	0	0
HYPERACUSIA												
HYPERACUSIS	1	0	1	0	0	0	1	0	1	0	0	0
INNER EAR AND VIIITH CRANIAL NERVE DISORDERS												
INNER EAR SIGNS AND SYMPTOMS												
MOTION SICKNESS	2	2	0	0	0	0	2	2	0	0	0	0
TINNITUS	75	46	15	7	0	7	71	46	12	6	0	7
VERTIGO	47	30	10	3	1	3	45	30	9	2	1	3
VERTIGO LABYRINTHINE	2	2	0	0	0	0	2	2	0	0	0	0
VERTIGO POSITIONAL	5	4	1	0	0	0	5	4	1	0	0	0
**ENDOCRINE DISORDERS**												
THYROID GLAND DISORDERS												
THYROID HYPERFUNCTION DISORDERS												
HYPERTHYROIDISM	1	1	0	0	0	0	1	1	0	0	0	0
**EYE DISORDERS**												
EYE DISORDERS NEC												
LACRIMATION DISORDERS												
DRY EYE	8	3	1	4	0	0	7	3	1	3	0	0
LACRIMATION INCREASED	4	2	0	1	0	1	3	2	0	0	0	1
OCULAR DISORDERS NEC												
EYE OEDEMA	1	1	0	0	0	0	1	1	0	0	0	0
EYE PAIN	95	72	8	6	0	9	94	72	7	6	0	9
EYE SWELLING	5	3	0	2	0	0	3	3	0	0	0	0
EYE ULCER	1	1	0	0	0	0	1	1	0	0	0	0
EYELID PAIN	1	1	0	0	0	0	1	1	0	0	0	0

OCULAR DISCOMFORT	2	2	0	0	0	0	2	2	0	0	0	0
PERIORBITAL DISCOMFORT	1	0	1	0	0	0	0	0	0	0	0	0
PERIORBITAL SWELLING	2	0	1	1	0	0	2	0	1	1	0	0
OCULAR HAEMORRHAGES AND VASCULAR DISORDERS NEC												
CONJUNCTIVAL AND CORNEAL BLEEDING AND VASCULAR DISORDERS												
CONJUNCTIVAL HAEMORRHAGE	2	1	0	0	0	1	2	1	0	0	0	1
LID BLEEDING AND VASCULAR DISORDERS												
EYELID BLEEDING	1	1	0	0	0	0	1	1	0	0	0	0
OCULAR INFECTIONS, IRRITATIONS AND INFLAMMATIONS												
LID, LASH AND LACRIMAL INFECTIONS, IRRITATIONS AND INFLAMMATIONS												
BLEPHARITIS	1	1	0	0	0	0	1	1	0	0	0	0
ERYTHEMA OF EYELID	1	1	0	0	0	0	1	1	0	0	0	0
EYELID IRRITATION	1	1	0	0	0	0	1	1	0	0	0	0
OCULAR INFECTIONS, INFLAMMATIONS AND ASSOCIATED MANIFESTATIONS												
EYE DISCHARGE	1	1	0	0	0	0	1	1	0	0	0	0
EYE IRRITATION	1	1	0	0	0	0	1	1	0	0	0	0
EYE PRURITUS	9	8	0	1	0	0	8	8	0	0	0	0
LIMBAL SWELLING	1	0	0	1	0	0	1	0	0	1	0	0
OCULAR HYPERAEMIA	10	6	1	3	0	0	10	6	1	3	0	0
OCULAR NEUROMUSCULAR DISORDERS												
EYELID MOVEMENT DISORDERS												
BLEPHAROSPASM	2	2	0	0	0	0	2	2	0	0	0	0
OCULAR SENSORY SYMPTOMS NEC												
OCULAR SENSATION DISORDERS												
ABNORMAL SENSATION IN EYE	2	1	1	0	0	0	2	1	1	0	0	0
ASTHENOPIA	26	18	3	3	0	2	23	18	1	2	0	2
FOREIGN BODY SENSATION IN EYES	1	1	0	0	0	0	1	1	0	0	0	0
PHOTOPHOBIA	19	14	3	0	0	2	18	14	2	0	0	2
OCULAR STRUCTURAL CHANGE, DEPOSIT AND DEGENERATION NEC												
CHOROID AND VITREOUS STRUCTURAL CHANGE, DEPOSIT AND DEGENERATION												
VITREOUS DETACHMENT	1	1	0	0	0	0	1	1	0	0	0	0
VITREOUS FLOATERS	2	2	0	0	0	0	2	2	0	0	0	0

RETINA, CHOROID AND VITREOUS HAEMORRHAGES AND VASCULAR DISORDERS												
RETINAL BLEEDING AND VASCULAR DISORDERS (EXCL RETINOPATHY)												
RETINAL VEIN OCCLUSION	2	0	2	0	0	0	2	0	2	0	0	0
RETINOPATHIES NEC												
RETINAL EXUDATES	1	1	0	0	0	0	1	1	0	0	0	0
VISION DISORDERS												
VISUAL DISORDERS NEC												
DIPLOPIA	2	1	0	0	0	1	2	1	0	0	0	1
HALO VISION	2	1	1	0	0	0	2	1	1	0	0	0
METAMORPHOPSIA	1	1	0	0	0	0	1	1	0	0	0	0
PHOTOPSIA	4	2	0	0	0	2	4	2	0	0	0	2
VISION BLURRED	38	27	9	1	0	1	34	27	7	0	0	0
VISUAL IMPAIRMENT AND BLINDNESS (EXCL COLOUR BLINDNESS)												
BLINDNESS	1	0	1	0	0	0	1	0	1	0	0	0
BLINDNESS TRANSIENT	1	1	0	0	0	0	1	1	0	0	0	0
SUDDEN VISUAL LOSS	1	1	0	0	0	0	1	1	0	0	0	0
VISUAL IMPAIRMENT	16	16	0	0	0	0	16	16	0	0	0	0
**GASTROINTESTINAL DISORDERS**												
ANAL AND RECTAL CONDITIONS NEC												
ANAL AND RECTAL SIGNS AND SYMPTOMS												
ANAL PARAESTHESIA	1	1	0	0	0	0	1	1	0	0	0	0
BENIGN NEOPLASMS GASTROINTESTINAL												
BENIGN ORAL CAVITY NEOPLASMS												
MOUTH CYST	1	1	0	0	0	0	1	1	0	0	0	0
DENTAL AND GINGIVAL CONDITIONS												
DENTAL DISORDERS NEC	1	1	0	0	0	0	1	1	0	0	0	0
TEETHING												
DENTAL PAIN AND SENSATION DISORDERS												
DENTAL PARAESTHESIA	1	1	0	0	0	0	1	1	0	0	0	0
TOOTHACHE	11	4	5	2	0	0	9	4	3	2	0	0
GINGIVAL DISORDERS, SIGNS AND SYMPTOMS NEC												
GINGIVAL BLISTER	1	1	0	0	0	0	1	1	0	0	0	0
GINGIVAL PAIN	7	5	2	0	0	0	7	5	2	0	0	0
GINGIVAL SWELLING	1	1	0	0	0	0	1	1	0	0	0	0
GASTROINTESTINAL CONDITIONS NEC												

GASTROINTESTINAL MUCOSAL DYSTROPHIES AND SECRETION DISORDERS												
BARRETT'S OESOPHAGUS	1	1	0	0	0	0	1	1	0	0	0	0
GASTROINTESTINAL HAEMORRHAGES NEC												
INTESTINAL HAEMORRHAGES												
RECTAL HAEMORRHAGE	1	1	0	0	0	0	1	1	0	0	0	0
NON-SITE SPECIFIC GASTROINTESTINAL HAEMORRHAGES												
HAEMATEMESIS	1	1	0	0	0	0	1	1	0	0	0	0
GASTROINTESTINAL INFLAMMATORY CONDITIONS												
COLITIS (EXCL INFECTIVE)												
COLITIS	1	0	1	0	0	0	1	0	1	0	0	0
COLITIS MICROSCOPIC	1	0	1	0	0	0	1	0	1	0	0	0
COLITIS ULCERATIVE	2	1	1	0	0	0	1	1	0	0	0	0
GASTRITIS (EXCL INFECTIVE)												
GASTRITIS	2	2	0	0	0	0	2	2	0	0	0	0
REFLUX GASTRITIS	1	1	0	0	0	0	1	1	0	0	0	0
GASTROINTESTINAL INFLAMMATORY DISORDERS NEC												
GASTROINTESTINAL TRACT IRRITATION	1	0	0	1	0	0	1	0	0	1	0	0
GASTROINTESTINAL MOTILITY AND DEFAECATION CONDITIONS												
DIARRHOEA (EXCL INFECTIVE)												
DIARRHOEA	331	234	46	32	6	13	309	234	32	26	5	12
GASTROINTESTINAL ATONIC AND HYPOMOTILITY DISORDERS NEC												
CONSTIPATION	10	6	2	2	0	0	8	6	1	1	0	0
GASTROOESOPHAGEAL REFLUX DISEASE	3	1	1	0	0	1	3	1	1	0	0	1
GASTROINTESTINAL DYSKINETIC DISORDERS												
CHANGE OF BOWEL HABIT	2	1	1	0	0	0	2	1	1	0	0	0
GASTROINTESTINAL SPASTIC AND HYPERMOTILITY DISORDERS												
IRRITABLE BOWEL SYNDROME	3	1	1	1	0	0	3	1	1	1	0	0
GASTROINTESTINAL SIGNS AND SYMPTOMS												
DYSPEPTIC SIGNS AND SYMPTOMS												
DYSPEPSIA	36	24	5	4	1	2	33	24	3	4	1	1
ERUCTATION	3	3	0	0	0	0	3	3	0	0	0	0
FAECAL ABNORMALITIES NEC												
FAECALOMA	1	0	1	0	0	0	1	0	1	0	0	0
FAECES DISCOLOURED	1	1	0	0	0	0	1	1	0	0	0	0
FAECES SOFT	3	1	1	1	0	0	3	1	1	1	0	0
FLATULENCE, BLOATING AND DISTENSION												
ABDOMINAL DISTENSION	11	9	0	1	1	0	11	9	0	1	1	0
FLATULENCE	18	16	1	1	0	0	17	16	1	0	0	0

GASTROINTESTINAL AND ABDOMINAL PAINS (EXCL ORAL AND THROAT)												
ABDOMINAL PAIN	72	54	12	2	1	3	67	54	8	2	0	3
ABDOMINAL PAIN LOWER	4	3	1	0	0	0	3	3	0	0	0	0
ABDOMINAL PAIN UPPER	137	98	21	7	1	10	128	98	14	6	0	10
GASTROINTESTINAL PAIN	11	9	0	2	0	0	10	9	0	1	0	0
GASTROINTESTINAL SIGNS AND SYMPTOMS NEC												
ABDOMINAL DISCOMFORT	78	53	16	5	0	4	73	53	12	5	0	3
ABDOMINAL SYMPTOM	1	1	0	0	0	0	1	1	0	0	0	0
ACUTE ABDOMEN	1	1	0	0	0	0	1	1	0	0	0	0
ANAL INCONTINENCE	1	1	0	0	0	0	1	1	0	0	0	0
BREATH ODOUR	1	1	0	0	0	0	1	1	0	0	0	0
DYSPHAGIA	2	1	1	0	0	0	1	1	0	0	0	0
ODYNOPHAGIA	2	1	1	0	0	0	1	1	0	0	0	0
NAUSEA AND VOMITING SYMPTOMS												
NAUSEA	1376	993	175	112	17	79	1307	993	125	98	16	75
RETCHING	3	3	0	0	0	0	3	3	0	0	0	0
VOMITING	180	122	20	17	4	17	173	122	18	13	3	17
VOMITING PROJECTILE	2	2	0	0	0	0	2	2	0	0	0	0
MALABSORPTION CONDITIONS												
MALABSORPTION SYNDROMES												
COELIAC DISEASE	1	0	1	0	0	0	1	0	1	0	0	0
ORAL SOFT TISSUE CONDITIONS												
ORAL SOFT TISSUE DISORDERS NEC												
CHEILITIS	1	1	0	0	0	0	1	1	0	0	0	0
LIP BLISTER	2	2	0	0	0	0	2	2	0	0	0	0
ORAL LICHEN PLANUS	1	1	0	0	0	0	1	1	0	0	0	0
ORAL SOFT TISSUE INFECTIONS												
ANGULAR CHEILITIS	1	1	0	0	0	0	1	1	0	0	0	0
ORAL SOFT TISSUE SIGNS AND SYMPTOMS												
HYPOAESTHESIA ORAL	8	7	1	0	0	0	7	7	0	0	0	0
LIP PAIN	3	2	1	0	0	0	3	2	1	0	0	0
ORAL DISCOMFORT	3	2	1	0	0	0	2	2	0	0	0	0
ORAL MUCOSAL ROUGHENING	1	1	0	0	0	0	1	1	0	0	0	0
ORAL PAIN	10	7	2	1	0	0	9	7	1	1	0	0
PARAESTHESIA ORAL	27	23	3	0	0	1	26	23	2	0	0	1
LIP SWELLING	11	7	1	1	0	2	11	7	1	1	0	2
MOUTH SWELLING	1	1	0	0	0	0	1	1	0	0	0	0
STOMATITIS AND ULCERATION												

APHTHOUS ULCER	1	0	1	0	0	0	0	0	0	0	0	0
LIP ULCERATION	1	0	0	0	1	0	0	0	0	0	0	0
MOUTH ULCERATION	14	8	3	2	1	0	14	8	3	2	1	0
STOMATITIS	2	1	1	0	0	0	2	1	1	0	0	0
SALIVARY GLAND CONDITIONS												
ORAL DRYNESS AND SALIVA ALTERED												
DRY MOUTH	35	20	4	0	4	7	32	20	3	0	2	7
LIP DRY	6	3	1	1	0	1	6	3	1	1	0	1
SALIVARY HYPOSECRETION	1	1	0	0	0	0	1	1	0	0	0	0
TONGUE CONDITIONS												
TONGUE SIGNS AND SYMPTOMS												
GLOSSODYNIA	9	6	1	2	0	0	8	6	1	1	0	0
SWOLLEN TONGUE	6	4	0	1	0	1	6	4	0	1	0	1
TONGUE COATED	3	3	0	0	0	0	3	3	0	0	0	0
TONGUE DISCOMFORT	2	1	1	0	0	0	1	1	0	0	0	0
TONGUE OEDEMA	1	0	0	1	0	0	1	0	0	1	0	0
TONGUE SPASM	1	0	0	1	0	0	1	0	0	1	0	0
**GENERAL DISORDERS AND ADMINISTRATION SITE CONDITIONS**												
ADMINISTRATION SITE REACTIONS												
ADMINISTRATION SITE REACTIONS NEC												
ADMINISTRATION SITE BRUISE	2	2	0	0	0	0	2	2	0	0	0	0
ADMINISTRATION SITE PAIN	1	1	0	0	0	0	1	1	0	0	0	0
PUNCTURE SITE BRUISE	17	9	3	2	0	3	17	9	3	2	0	3
PUNCTURE SITE PAIN	3	2	0	0	0	1	3	2	0	0	0	1
APPLICATION SITE REACTIONS												
APPLICATION SITE BRUISE	9	5	2	1	1	0	8	5	1	1	1	0
APPLICATION SITE ERYTHEMA	2	1	1	0	0	0	2	1	1	0	0	0
APPLICATION SITE PAIN	6	2	1	3	0	0	5	2	1	2	0	0
IMPLANT AND CATHETER SITE REACTIONS												
IMPLANT SITE PAIN	2	0	2	0	0	0	1	0	1	0	0	0
IMPLANT SITE WARMTH	2	2	0	0	0	0	2	2	0	0	0	0
INFUSION SITE REACTIONS												
INFUSION SITE PAIN	2	1	0	1	0	0	2	1	0	1	0	0
INFUSION SITE SCAB	1	1	0	0	0	0	1	1	0	0	0	0
INFUSION SITE WARMTH	1	1	0	0	0	0	1	1	0	0	0	0
INJECTION SITE REACTIONS												
INJECTION SITE BRUISING	8	4	2	2	0	0	8	4	2	2	0	0
INJECTION SITE DISCOMFORT	3	2	1	0	0	0	2	2	0	0	0	0

INJECTION SITE ERYTHEMA	32	21	5	3	2	1	30	21	4	3	1	1
INJECTION SITE HYPERSENSITIVITY	1	0	1	0	0	0	1	0	1	0	0	0
INJECTION SITE INFLAMMATION	2	1	0	0	0	1	2	1	0	0	0	1
INJECTION SITE INJURY	1	1	0	0	0	0	1	1	0	0	0	0
INJECTION SITE IRRITATION	1	0	1	0	0	0	1	0	1	0	0	0
INJECTION SITE JOINT PAIN	3	1	1	0	1	0	1	1	0	0	0	0
INJECTION SITE MASS	46	29	9	4	2	2	44	29	8	3	2	2
INJECTION SITE NODULE	1	0	0	1	0	0	1	0	0	1	0	0
INJECTION SITE OEDEMA	1	1	0	0	0	0	1	1	0	0	0	0
INJECTION SITE PAIN	539	353	92	64	9	21	505	353	72	50	9	21
INJECTION SITE PAPULE	1	1	0	0	0	0	1	1	0	0	0	0
INJECTION SITE PARAESTHESIA	1	1	0	0	0	0	1	1	0	0	0	0
INJECTION SITE PRURITUS	16	13	1	2	0	0	15	13	0	2	0	0
INJECTION SITE RASH	9	7	1	1	0	0	9	7	1	1	0	0
INJECTION SITE REACTION	4	2	2	0	0	0	4	2	2	0	0	0
INJECTION SITE SCAB	2	1	1	0	0	0	2	1	1	0	0	0
INJECTION SITE SWELLING	18	10	1	5	0	2	18	10	1	5	0	2
INJECTION SITE URTICARIA	5	0	2	0	3	0	5	0	2	0	3	0
INJECTION SITE WARMTH	16	12	2	1	1	0	16	12	2	1	1	0
INSTILLATION SITE REACTIONS												
INSTILLATION SITE PRURITUS	1	0	1	0	0	0	1	0	1	0	0	0
INSTILLATION SITE WARMTH	3	2	0	0	0	1	3	2	0	0	0	1
VACCINATION SITE REACTIONS												
SHOULDER INJURY RELATED TO VACCINE ADMINIST	1	1	0	0	0	0	1	1	0	0	0	0
VACCINATION SITE BRUISING	4	1	3	0	0	0	3	1	2	0	0	0
VACCINATION SITE DISCOMFORT	1	1	0	0	0	0	1	1	0	0	0	0
VACCINATION SITE ERYTHEMA	2	1	0	0	0	1	2	1	0	0	0	1
VACCINATION SITE JOINT ERYTHEMA	1	1	0	0	0	0	1	1	0	0	0	0
VACCINATION SITE JOINT PAIN	6	0	0	1	0	5	6	0	0	1	0	5
VACCINATION SITE MASS	16	11	3	1	0	1	13	11	1	0	0	1
VACCINATION SITE PAIN	87	49	11	12	1	14	83	49	8	12	1	13
VACCINATION SITE RASH	2	1	0	0	0	1	2	1	0	0	0	1
VACCINATION SITE SWELLING	4	1	1	0	0	2	4	1	1	0	0	2
VACCINATION SITE WARMTH	6	4	1	0	0	1	5	4	0	0	0	1
BODY TEMPERATURE CONDITIONS												
BODY TEMPERATURE ALTERED												
HYPERTHERMIA	1	1	0	0	0	0	1	1	0	0	0	0
HYPOTHERMIA	1	1	0	0	0	0	1	1	0	0	0	0

FEBRILE DISORDERS												
PYREXIA	2274	1671	313	160	30	100	2134	1671	203	134	27	99
FATAL OUTCOMES												
DEATH AND SUDDEN DEATH												
DEATH	3	3	0	0	0	0	3	3	0	0	0	0
GENERAL SYSTEM DISORDERS NEC												
ADVERSE EFFECT ABSENT												
NO ADVERSE EVENT	1	0	0	0	0	1	1	0	0	0	0	1
ASTHENIC CONDITIONS												
ASTHENIA	130	89	21	9	2	9	118	89	15	5	1	8
CHRONIC FATIGUE SYNDROME	6	2	2	2	0	0	5	2	2	1	0	0
DECREASED ACTIVITY	1	1	0	0	0	0	1	1	0	0	0	0
FATIGUE	4468	2946	833	362	70	257	4182	2946	631	302	58	245
MALAISE	438	286	71	46	11	24	403	286	50	36	8	23
SLUGGISHNESS	2	0	1	0	1	0	2	0	1	0	1	0
FEELINGS AND SENSATIONS NEC												
CHILLS	1770	1340	190	144	13	83	1677	1340	122	126	10	79
FEELING ABNORMAL	123	75	22	12	5	9	112	75	14	9	5	9
FEELING COLD	353	258	51	25	7	12	316	258	25	17	5	11
FEELING HOT	89	61	15	8	1	4	85	61	12	8	0	4
FEELING JITTERY	1	0	1	0	0	0	1	0	1	0	0	0
FEELING OF BODY TEMPERATURE CHANGE	61	45	9	4	0	3	58	45	6	4	0	3
FEELING OF RELAXATION	1	1	0	0	0	0	1	1	0	0	0	0
HANGOVER	10	7	0	1	0	2	10	7	0	1	0	2
HUNGER	7	3	1	1	0	2	6	3	1	0	0	2
SENSATION OF BLOOD FLOW	1	1	0	0	0	0	1	1	0	0	0	0
THIRST	39	32	4	2	0	1	36	32	1	2	0	1
GAIT DISTURBANCES												
GAIT DISTURBANCE	9	3	3	0	0	3	9	3	3	0	0	3
GAIT INABILITY	3	3	0	0	0	0	3	3	0	0	0	0
GENERAL SIGNS AND SYMPTOMS NEC												
CRYING	1	1	0	0	0	0	1	1	0	0	0	0
ENERGY INCREASED	1	0	1	0	0	0	1	0	1	0	0	0
EXERCISE TOLERANCE DECREASED	2	1	0	0	0	1	2	1	0	0	0	1
GENERAL SYMPTOM	2	2	0	0	0	0	2	2	0	0	0	0
ILLNESS	146	93	22	20	0	11	134	93	14	16	0	11
INFLUENZA LIKE ILLNESS	665	442	100	77	9	37	599	442	54	60	8	35
LOCAL REACTION	5	4	1	0	0	0	5	4	1	0	0	0

PERIPHERAL SWELLING	236	139	41	39	5	12	221	139	36	30	4	12
SWELLING	80	50	13	13	0	4	73	50	10	10	0	3
SWELLING FACE	14	11	1	0	0	2	14	11	1	0	0	2
TISSUE IRRITATION	1	0	1	0	0	0	1	0	1	0	0	0
INFLAMMATIONS												
INFLAMMATION	13	7	6	0	0	0	10	7	3	0	0	0
SYSTEMIC INFLAMMATORY RESPONSE SYNDROME	1	0	1	0	0	0	0	0	0	0	0	0
OEDEMA NEC												
OEDEMA	1	1	0	0	0	0	1	1	0	0	0	0
OEDEMA PERIPHERAL	2	1	1	0	0	0	2	1	1	0	0	0
PAIN AND DISCOMFORT NEC												
AXILLARY PAIN	90	39	22	27	1	1	86	39	19	26	1	1
CHEST DISCOMFORT	39	28	2	7	0	2	38	28	1	7	0	2
CHEST PAIN	95	64	14	9	2	6	87	64	10	7	1	5
DISCOMFORT	22	8	5	5	2	2	17	8	3	4	0	2
FACIAL PAIN	3	2	0	1	0	0	2	2	0	0	0	0
HERNIA PAIN	1	1	0	0	0	0	1	1	0	0	0	0
INFLAMMATORY PAIN	3	2	0	0	1	0	2	2	0	0	0	0
NON-CARDIAC CHEST PAIN	1	0	0	1	0	0	1	0	0	1	0	0
PAIN	1075	727	187	97	15	49	994	727	130	78	12	47
TENDERNESS	364	247	54	39	4	20	349	247	44	35	4	19
THERAPEUTIC AND NONTHERAPEUTIC EFFECTS (EXCL TOXICITY)												
THERAPEUTIC AND NONTHERAPEUTIC RESPONSES												
ADVERSE DRUG REACTION	3	3	0	0	0	0	3	3	0	0	0	0
ADVERSE EVENT	5	0	0	0	0	5	4	0	0	0	0	4
ADVERSE REACTION	1	1	0	0	0	0	1	1	0	0	0	0
IMMEDIATE POST-INJECTION REACTION	1	0	1	0	0	0	1	0	1	0	0	0
**HEPATOBILIARY DISORDERS**												
HEPATIC AND HEPATOBILIARY DISORDERS												
HEPATOBILIARY SIGNS AND SYMPTOMS												
HEPATIC PAIN	2	2	0	0	0	0	2	2	0	0	0	0
HEPATOCELLULAR DAMAGE AND HEPATITIS NEC												
LIVER INJURY	1	1	0	0	0	0	1	1	0	0	0	0
**IMMUNE SYSTEM DISORDERS**												
ALLERGIC CONDITIONS												
ALLERGIC CONDITIONS NEC												
HYPERSENSITIVITY	8	4	3	1	0	0	6	4	1	1	0	0
MULTIPLE ALLERGIES	1	1	0	0	0	0	1	1	0	0	0	0

ALLERGIES TO FOODS, FOOD ADDITIVES, DRUGS AND OTHER CHEMICALS												
ALLERGY TO CHEMICALS	1	0	1	0	0	0	1	0	1	0	0	0
ANAPHYLACTIC AND ANAPHYLACTOID RESPONSES												
ANAPHYLACTIC REACTION	3	3	0	0	0	0	3	3	0	0	0	0
ATOPIC DISORDERS												
SEASONAL ALLERGY	1	0	1	0	0	0	1	0	1	0	0	0
IMMUNE DISORDERS NEC												
IMMUNE AND ASSOCIATED CONDITIONS NEC												
BACILLE CALMETTE-GUERIN SCAR REACTIVATION	1	1	0	0	0	0	1	1	0	0	0	0
SENSITISATION	1	1	0	0	0	0	1	1	0	0	0	0
**INFECTIONS AND INFESTATIONS**												
BACTERIAL INFECTIOUS DISORDERS												
BACTERIAL INFECTIONS NEC												
CELLULITIS	4	3	1	0	0	0	3	3	0	0	0	0
PERIORBITAL CELLULITIS	1	0	1	0	0	0	1	0	1	0	0	0
HELICOBACTER INFECTIONS												
HELICOBACTER GASTRITIS	1	0	0	1	0	0	1	0	0	1	0	0
STAPHYLOCOCCAL INFECTIONS												
FURUNCLE	4	2	1	0	0	1	3	2	0	0	0	1
FUNGAL INFECTIOUS DISORDERS												
CANDIDA INFECTIONS												
CANDIDA INFECTION	1	1	0	0	0	0	1	1	0	0	0	0
VULVOVAGINAL CANDIDIASIS	2	2	0	0	0	0	2	2	0	0	0	0
INFECTIONS - PATHOGEN UNSPECIFIED												
ABDOMINAL AND GASTROINTESTINAL INFECTIONS												
APPENDICITIS	1	1	0	0	0	0	1	1	0	0	0	0
DIARRHOEA INFECTIOUS	1	0	0	1	0	0	1	0	0	1	0	0
GASTROINTESTINAL INFECTION	1	1	0	0	0	0	1	1	0	0	0	0
BREAST INFECTIONS												
MASTITIS	3	0	1	2	0	0	3	0	1	2	0	0
CENTRAL NERVOUS SYSTEM AND SPINAL INFECTIONS												
MYELITIS	1	1	0	0	0	0	1	1	0	0	0	0
DENTAL AND ORAL SOFT TISSUE INFECTIONS												
ABSCESS ORAL	1	1	0	0	0	0	1	1	0	0	0	0
ORAL PUSTULE	1	1	0	0	0	0	1	1	0	0	0	0
PERICORONITIS	1	1	0	0	0	0	1	1	0	0	0	0
TOOTH ABSCESS	1	1	0	0	0	0	1	1	0	0	0	0

EAR INFECTIONS												
EAR INFECTION	2	1	0	0	0	1	2	1	0	0	0	1
LABYRINTHITIS	3	1	2	0	0	0	3	1	2	0	0	0
OTITIS EXTERNA	1	1	0	0	0	0	1	1	0	0	0	0
EYE AND EYELID INFECTIONS												
CONJUNCTIVITIS	2	0	2	0	0	0	1	0	1	0	0	0
HORDEOLUM	1	1	0	0	0	0	1	1	0	0	0	0
INFECTIONS NEC												
ABSCESS	1	1	0	0	0	0	1	1	0	0	0	0
INFECTION	2	1	0	0	0	1	2	1	0	0	0	1
INJECTION SITE INFECTION	1	1	0	0	0	0	1	1	0	0	0	0
LOCALISED INFECTION	6	5	0	0	0	1	5	5	0	0	0	0
WOUND INFECTION	1	0	1	0	0	0	0	0	0	0	0	0
LOWER RESPIRATORY TRACT AND LUNG INFECTIONS												
LOWER RESPIRATORY TRACT INFECTION	8	4	2	1	0	1	8	4	2	1	0	1
PNEUMONIA	5	2	1	0	0	2	4	2	0	0	0	2
MALE REPRODUCTIVE TRACT INFECTIONS												
ORCHITIS	1	1	0	0	0	0	1	1	0	0	0	0
SEPSIS, BACTERAEMIA, VIRAEMIA AND FUNGAEMIA NEC												
SEPTIC RASH	1	0	1	0	0	0	0	0	0	0	0	0
SKIN STRUCTURES AND SOFT TISSUE INFECTIONS												
INFECTED DERMAL CYST	1	1	0	0	0	0	1	1	0	0	0	0
INJECTION SITE PUSTULE	1	0	1	0	0	0	0	0	0	0	0	0
SKIN INFECTION	1	1	0	0	0	0	1	1	0	0	0	0
UPPER RESPIRATORY TRACT INFECTIONS												
LARYNGITIS	2	1	1	0	0	0	2	1	1	0	0	0
NASOPHARYNGITIS	208	129	37	32	1	9	191	129	26	27	1	8
PHARYNGITIS	1	1	0	0	0	0	1	1	0	0	0	0
RHINITIS	2	1	0	0	0	1	2	1	0	0	0	1
SINUSITIS	13	9	2	1	0	1	11	9	0	1	0	1
TONSILLITIS	1	0	0	0	0	1	1	0	0	0	0	1
URINARY TRACT INFECTIONS												
CYSTITIS	2	1	1	0	0	0	2	1	1	0	0	0
URINARY TRACT INFECTION	3	2	1	0	0	0	2	2	0	0	0	0
PROTOZOAL INFECTIOUS DISORDERS												
TRYPANOSOMAL INFECTIONS												
AFRICAN TRYPANOSOMIASIS	1	1	0	0	0	0	1	1	0	0	0	0
RICKETTSIAL INFECTIOUS DISORDERS												

COXIELLA INFECTIONS												
Q FEVER	5	5	0	0	0	0	5	5	0	0	0	0
VIRAL INFECTIOUS DISORDERS												
CORONAVIRUS INFECTIONS												
COVID-19	7	1	1	1	3	1	4	1	0	1	2	0
HERPES VIRAL INFECTIONS												
GENITAL HERPES	1	1	0	0	0	0	1	1	0	0	0	0
HERPES OPHTHALMIC	1	1	0	0	0	0	1	1	0	0	0	0
HERPES SIMPLEX	2	1	1	0	0	0	1	1	0	0	0	0
HERPES ZOSTER	15	8	4	1	0	2	14	8	4	0	0	2
NASAL HERPES	1	1	0	0	0	0	1	1	0	0	0	0
OPHTHALMIC HERPES ZOSTER	1	1	0	0	0	0	1	1	0	0	0	0
ORAL HERPES	33	26	5	1	0	1	32	26	4	1	0	1
INFLUENZA VIRAL INFECTIONS												
H2N2 INFLUENZA	1	1	0	0	0	0	1	1	0	0	0	0
INFLUENZA	668	464	91	82	9	22	614	464	61	60	8	21
RETROVIRAL INFECTIONS												
AIDS RELATED COMPLEX	1	1	0	0	0	0	1	1	0	0	0	0
VIRAL INFECTIONS NEC												
GASTROENTERITIS VIRAL	4	4	0	0	0	0	4	4	0	0	0	0
SWEATING FEVER	10	8	0	0	0	2	10	8	0	0	0	2
VESTIBULAR NEURONITIS	1	0	0	0	1	0	0	0	0	0	0	0
VIRAL DIARRHOEA	1	1	0	0	0	0	1	1	0	0	0	0
VIRAL RASH	2	1	0	0	1	0	2	1	0	0	1	0
VIRAL UPPER RESPIRATORY TRACT INFECTION	1	1	0	0	0	0	1	1	0	0	0	0
**INJURY, POISONING AND PROCEDURAL COMPLICATIONS**												
BONE AND JOINT INJURIES												
FRACTURES AND DISLOCATIONS NEC												
JOINT DISLOCATION	1	1	0	0	0	0	1	1	0	0	0	0
EXPOSURES, CHEMICAL INJURIES AND POISONING												
POISONING AND TOXICITY												
SYSTEMIC TOXICITY	1	1	0	0	0	0	1	1	0	0	0	0
TOXICITY TO VARIOUS AGENTS	2	2	0	0	0	0	2	2	0	0	0	0
INJURIES BY PHYSICAL AGENTS												
CONDITIONS CAUSED BY COLD												
CHILLBLAINS	3	2	0	0	0	1	3	2	0	0	0	1
HEAT INJURIES (EXCL THERMAL BURNS)												
HEAT EXHAUSTION	1	1	0	0	0	0	1	1	0	0	0	0

HEAT OEDEMA	1	1	0	0	0	0	1	1	0	0	0	0
RADIATION INJURIES												
SUNBURN	1	0	1	0	0	0	0	0	0	0	0	0
THERMAL BURNS												
THERMAL BURN	1	1	0	0	0	0	1	1	0	0	0	0
THERMAL BURNS OF EYE	1	1	0	0	0	0	1	1	0	0	0	0
INJURIES NEC												
CHEST AND RESPIRATORY TRACT INJURIES NEC												
CHEST CRUSHING	1	1	0	0	0	0	1	1	0	0	0	0
EYE INJURIES NEC												
EYE CONTUSION	1	1	0	0	0	0	1	1	0	0	0	0
MUSCLE, TENDON AND LIGAMENT INJURIES												
LIGAMENT SPRAIN	1	1	0	0	0	0	1	1	0	0	0	0
MUSCLE INJURY	2	1	0	1	0	0	1	1	0	0	0	0
MUSCLE STRAIN	2	1	1	0	0	0	2	1	1	0	0	0
NERVE INJURIES NEC												
NERVE INJURY	1	1	0	0	0	0	1	1	0	0	0	0
NON-SITE SPECIFIC INJURIES NEC												
ARTHROPOD STING	2	2	0	0	0	0	2	2	0	0	0	0
BITE	1	0	1	0	0	0	1	0	1	0	0	0
FALL	5	4	0	1	0	0	5	4	0	1	0	0
INFLAMMATION OF WOUND	2	2	0	0	0	0	2	2	0	0	0	0
TISSUE INJURY	1	1	0	0	0	0	1	1	0	0	0	0
WOUND COMPLICATION	1	1	0	0	0	0	1	1	0	0	0	0
WOUND SECRETION	1	1	0	0	0	0	1	1	0	0	0	0
SITE SPECIFIC INJURIES NEC												
LIMB INJURY	6	6	0	0	0	0	6	6	0	0	0	0
SKIN INJURIES NEC												
CONTUSION	145	85	27	18	3	12	132	85	18	15	3	11
SCAR	2	1	1	0	0	0	2	1	1	0	0	0
SKIN WOUND	1	0	1	0	0	0	0	0	0	0	0	0
PROCEDURAL RELATED INJURIES AND COMPLICATIONS NEC												
CARDIAC AND VASCULAR PROCEDURAL COMPLICATIONS												
CARDIAC PROCEDURE COMPLICATION	1	0	1	0	0	0	0	0	0	0	0	0
GASTROINTESTINAL AND HEPATOBILIARY PROCEDURAL COMPLICATIONS												
PROCEDURAL NAUSEA	6	6	0	0	0	0	6	6	0	0	0	0
NEUROLOGICAL AND PSYCHIATRIC PROCEDURAL COMPLICATIONS												
PROCEDURAL DIZZINESS	2	2	0	0	0	0	2	2	0	0	0	0

NON-SITE SPECIFIC PROCEDURAL COMPLICATIONS												
INCISION SITE PAIN	1	1	0	0	0	0	1	1	0	0	0	0
INCISION SITE SWELLING	1	0	1	0	0	0	1	0	1	0	0	0
INJECTION RELATED REACTION	49	41	3	2	3	0	49	41	3	2	3	0
POST PROCEDURAL COMPLICATION	10	6	2	1	1	0	6	6	0	0	0	0
PROCEDURAL PAIN	1	0	0	0	0	1	1	0	0	0	0	1
VACCINATION RELATED COMPLICATIONS												
IMMUNISATION REACTION	8	3	4	1	0	0	7	3	3	1	0	0
**INVESTIGATIONS**												
CARDIAC AND VASCULAR INVESTIGATIONS (EXCL ENZYME TESTS)												
HEART RATE AND PULSE INVESTIGATIONS												
HEART RATE	29	21	3	2	0	3	29	21	3	2	0	3
HEART RATE DECREASED	3	2	0	0	0	1	3	2	0	0	0	1
HEART RATE INCREASED	42	34	3	2	0	3	40	34	1	2	0	3
HEART RATE IRREGULAR	2	0	1	1	0	0	2	0	1	1	0	0
VASCULAR TESTS NEC (INCL BLOOD PRESSURE)												
BLOOD PRESSURE DECREASED	1	0	1	0	0	0	1	0	1	0	0	0
BLOOD PRESSURE INCREASED	7	5	1	1	0	0	7	5	1	1	0	0
BLOOD PRESSURE MEASUREMENT	3	1	0	1	0	1	2	1	0	0	0	1
ENDOCRINE INVESTIGATIONS (INCL SEX HORMONES)												
ENDOCRINE ANALYSES AND IMAGING NEC												
HORMONE LEVEL ABNORMAL	1	1	0	0	0	0	1	1	0	0	0	0
PITUITARY ANALYSES ANTERIOR												
BLOOD FOLLICLE STIMULATING HORMONE INCREA	1	0	0	1	0	0	1	0	0	1	0	0
BLOOD LUTEINISING HORMONE	1	1	0	0	0	0	1	1	0	0	0	0
THYROID ANALYSES												
TRI-IODOTHYRONINE	1	1	0	0	0	0	1	1	0	0	0	0
HAEMATOLOGY INVESTIGATIONS (INCL BLOOD GROUPS)												
COAGULATION AND BLEEDING ANALYSES												
BLEEDING TIME	1	0	0	0	0	1	1	0	0	0	0	1
INTERNATIONAL NORMALISED RATIO DECREASED	1	0	1	0	0	0	0	0	0	0	0	0
PLATELET ANALYSES												
PLATELET COUNT INCREASED	2	0	1	0	0	1	2	0	1	0	0	1
RED BLOOD CELL ANALYSES												
HAEMOGLOBIN	1	1	0	0	0	0	1	1	0	0	0	0
METABOLIC, NUTRITIONAL AND BLOOD GAS INVESTIGATIONS												
BLOOD GAS AND ACID BASE ANALYSES												
OXYGEN SATURATION DECREASED	2	2	0	0	0	0	2	2	0	0	0	0

CARBOHYDRATE TOLERANCE ANALYSES (INCL DIABETES)												
BLOOD GLUCOSE	3	3	0	0	0	0	3	3	0	0	0	0
BLOOD GLUCOSE ABNORMAL	1	1	0	0	0	0	1	1	0	0	0	0
BLOOD GLUCOSE DECREASED	1	1	0	0	0	0	1	1	0	0	0	0
BLOOD GLUCOSE INCREASED	6	6	0	0	0	0	6	6	0	0	0	0
MICROBIOLOGY AND SEROLOGY INVESTIGATIONS												
VIRUS IDENTIFICATION AND SEROLOGY												
SARS-COV-2 TEST	1	1	0	0	0	0	1	1	0	0	0	0
SARS-COV-2 TEST POSITIVE	1	0	0	1	0	0	1	0	0	1	0	0
MUSCULOSKELETAL AND SOFT TISSUE INVESTIGATIONS (EXCL ENZYME TESTS)												
MUSCULOSKELETAL AND SOFT TISSUE IMAGING PROCEDURES												
BONE SCAN	1	1	0	0	0	0	1	1	0	0	0	0
NEUROLOGICAL, SPECIAL SENSES AND PSYCHIATRIC INVESTIGATIONS												
CENTRAL NERVOUS SYSTEM IMAGING PROCEDURES												
MAGNETIC RESONANCE IMAGING HEAD	8	8	0	0	0	0	8	8	0	0	0	0
SCAN BRAIN	1	0	0	1	0	0	1	0	0	1	0	0
PHYSICAL EXAMINATION AND ORGAN SYSTEM STATUS TOPICS												
PHYSICAL EXAMINATION PROCEDURES AND ORGAN SYSTEM STATUS												
BODY TEMPERATURE	64	43	10	2	0	9	62	43	8	2	0	9
BODY TEMPERATURE ABNORMAL	3	2	0	1	0	0	3	2	0	1	0	0
BODY TEMPERATURE DECREASED	2	2	0	0	0	0	2	2	0	0	0	0
BODY TEMPERATURE FLUCTUATION	10	7	2	1	0	0	10	7	2	1	0	0
BODY TEMPERATURE INCREASED	64	43	9	10	1	1	59	43	6	9	0	1
GRIP STRENGTH DECREASED	1	1	0	0	0	0	1	1	0	0	0	0
HEAD LAG	2	2	0	0	0	0	2	2	0	0	0	0
LEFT-HANDEDNESS	1	1	0	0	0	0	1	1	0	0	0	0
LYMPH NODE PALPABLE	1	0	0	1	0	0	1	0	0	1	0	0
RESPIRATORY RATE DECREASED	1	0	0	0	0	1	1	0	0	0	0	1
SKIN TEMPERATURE	5	3	1	1	0	0	5	3	1	1	0	0
WEIGHT DECREASED	3	3	0	0	0	0	3	3	0	0	0	0
WEIGHT INCREASED	3	2	0	0	0	1	3	2	0	0	0	1
RENAL AND URINARY TRACT INVESTIGATIONS AND URINALYSES												
URINALYSIS NEC												
BLOOD URINE	2	2	0	0	0	0	2	2	0	0	0	0
NITRITE URINE PRESENT	1	1	0	0	0	0	1	1	0	0	0	0
PH URINE	2	1	0	0	0	1	2	1	0	0	0	1
URINARY TRACT FUNCTION ANALYSES NEC												
URINE OUTPUT	3	2	0	1	0	0	3	2	0	1	0	0

URINE OUTPUT INCREASED	1	1	0	0	0	0	1	1	0	0	0	0
REPRODUCTIVE ORGAN AND BREAST INVESTIGATIONS (EXCL HORMONE ANALYSES)												
REPRODUCTIVE ORGAN AND BREAST IMAGING PROCEDURES												
BREAST SCAN	1	0	1	0	0	0	1	0	1	0	0	0
RESPIRATORY AND PULMONARY INVESTIGATIONS (EXCL BLOOD GASES)												
RESPIRATORY AND PULMONARY FUNCTION DIAGNOSTIC PROCEDURES												
FORCED EXPIRATORY VOLUME DECREASED	1	1	0	0	0	0	1	1	0	0	0	0
FORCED EXPIRATORY VOLUME INCREASED	1	0	1	0	0	0	1	0	1	0	0	0
WATER, ELECTROLYTE AND MINERAL INVESTIGATIONS												
WATER AND ELECTROLYTE ANALYSES NEC												
VOLUME BLOOD	1	0	1	0	0	0	1	0	1	0	0	0
**METABOLISM AND NUTRITION DISORDERS**												
APPETITE AND GENERAL NUTRITIONAL DISORDERS												
APPETITE DISORDERS												
APPETITE DISORDER	1	0	0	0	0	1	1	0	0	0	0	1
DECREASED APPETITE	154	113	14	9	3	15	146	113	8	9	2	14
FOOD CRAVING	1	1	0	0	0	0	1	1	0	0	0	0
FOOD REFUSAL	2	1	0	1	0	0	1	1	0	0	0	0
INCREASED APPETITE	2	1	0	0	0	1	2	1	0	0	0	1
GENERAL NUTRITIONAL DISORDERS NEC												
FOOD AVERSION	2	2	0	0	0	0	2	2	0	0	0	0
ELECTROLYTE AND FLUID BALANCE CONDITIONS												
TOTAL FLUID VOLUME DECREASED												
DEHYDRATION	14	13	1	0	0	0	13	13	0	0	0	0
TOTAL FLUID VOLUME INCREASED												
FLUID RETENTION	1	0	0	1	0	0	0	0	0	0	0	0
FOOD INTOLERANCE SYNDROMES												
FOOD MALABSORPTION AND INTOLERANCE SYNDROMES (EXCL SUGAR INTOLERANCE)												
ALCOHOL INTOLERANCE	1	1	0	0	0	0	1	1	0	0	0	0
GLUCOSE METABOLISM DISORDERS (INCL DIABETES MELLITUS)												
DIABETES MELLITUS (INCL SUBTYPES)												
DIABETES MELLITUS	1	1	0	0	0	0	1	1	0	0	0	0
DIABETES MELLITUS INADEQUATE CONTROL	3	2	0	0	0	1	3	2	0	0	0	1
HYPERGLYCAEMIC CONDITIONS NEC												
HYPERGLYCAEMIA	1	1	0	0	0	0	1	1	0	0	0	0
HYPOGLYCAEMIC CONDITIONS NEC												
HYPOGLYCAEMIA	3	3	0	0	0	0	3	3	0	0	0	0
PURINE AND PYRIMIDINE METABOLISM DISORDERS												

DISORDERS OF PURINE METABOLISM												
GOUT	1	1	0	0	0	0	1	1	0	0	0	0
**MUSCULOSKELETAL AND CONNECTIVE TISSUE DISORDERS**												
BONE DISORDERS (EXCL CONGENITAL AND FRACTURES)												
BONE DISORDERS NEC												
JAW DISORDER	1	1	0	0	0	0	1	1	0	0	0	0
OSTEITIS	1	1	0	0	0	0	1	1	0	0	0	0
BONE RELATED SIGNS AND SYMPTOMS												
BONE PAIN	16	12	1	1	1	1	14	12	0	1	0	1
BONE SWELLING	1	0	0	0	0	1	1	0	0	0	0	1
PAIN IN JAW	15	10	4	1	0	0	15	10	4	1	0	0
PUBIC PAIN	1	1	0	0	0	0	1	1	0	0	0	0
SPINAL PAIN	3	2	1	0	0	0	3	2	1	0	0	0
CONNECTIVE TISSUE DISORDERS (EXCL CONGENITAL)												
CONNECTIVE TISSUE DISORDERS NEC												
POLYMYALGIA RHEUMATICA	4	1	1	1	1	0	4	1	1	1	1	0
LUPUS ERYTHEMATOSUS (INCL SUBTYPES)												
SYSTEMIC LUPUS ERYTHEMATOSUS	1	1	0	0	0	0	1	1	0	0	0	0
JOINT DISORDERS												
ARTHROPATHIES NEC												
ARTHRITIS	13	7	3	2	0	1	12	7	3	2	0	0
ARTHROPATHY	2	1	1	0	0	0	2	1	1	0	0	0
POLYARTHRITIS	1	0	1	0	0	0	1	0	1	0	0	0
RHEUMATIC FEVER	1	1	0	0	0	0	1	1	0	0	0	0
JOINT RELATED DISORDERS NEC												
JOINT LOCK	2	1	0	0	0	1	2	1	0	0	0	1
PERIARTHRITIS	6	2	2	2	0	0	6	2	2	2	0	0
TEMPOROMANDIBULAR PAIN AND DYSFUNCTION S	1	1	0	0	0	0	1	1	0	0	0	0
JOINT RELATED SIGNS AND SYMPTOMS												
ARTHRALGIA	1380	956	208	136	20	60	1285	956	149	111	15	54
JAW CLICKING	1	1	0	0	0	0	1	1	0	0	0	0
JOINT STIFFNESS	39	29	4	3	1	2	35	29	2	2	1	1
JOINT SWELLING	12	7	2	3	0	0	10	7	2	1	0	0
OSTEOARTHROPATHIES												
OSTEOARTHRITIS	2	1	1	0	0	0	1	1	0	0	0	0
PSORIATIC ARTHROPATHIES												
PSORIATIC ARTHROPATHY	1	1	0	0	0	0	1	1	0	0	0	0
RHEUMATOID ARTHROPATHIES												

RHEUMATOID ARTHRITIS	3	3	0	0	0	0	3	3	0	0	0	0
MUSCLE DISORDERS												
MUSCLE INFECTIONS AND INFLAMMATIONS												
MYOSITIS	1	0	1	0	0	0	1	0	1	0	0	0
MUSCLE PAINS												
FIBROMYALGIA	4	2	0	2	0	0	4	2	0	2	0	0
MYALGIA	1820	1294	280	150	31	65	1697	1294	201	114	27	61
MUSCLE RELATED SIGNS AND SYMPTOMS NEC												
MUSCLE ATROPHY	2	1	0	0	0	1	2	1	0	0	0	1
MUSCLE DISCOMFORT	1	1	0	0	0	0	1	1	0	0	0	0
MUSCLE FATIGUE	52	39	7	4	0	2	49	39	5	3	0	2
MUSCLE FIBROSIS	1	0	0	0	0	1	1	0	0	0	0	1
MUSCLE MASS	3	3	0	0	0	0	3	3	0	0	0	0
MUSCLE SPASMS	59	34	12	4	1	8	54	34	8	3	1	8
MUSCLE TIGHTNESS	3	2	1	0	0	0	3	2	1	0	0	0
MUSCLE TWITCHING	9	8	0	0	0	1	9	8	0	0	0	1
MUSCLE TONE ABNORMALITIES												
MUSCLE RIGIDITY	1	1	0	0	0	0	1	1	0	0	0	0
TRISMUS	2	1	1	0	0	0	2	1	1	0	0	0
MUSCLE WEAKNESS CONDITIONS												
MUSCULAR WEAKNESS	57	41	10	3	0	3	55	41	9	2	0	3
MUSCULOSKELETAL AND CONNECTIVE TISSUE DISORDERS NEC												
MUSCULOSKELETAL AND CONNECTIVE TISSUE CONDITIONS NEC												
MOBILITY DECREASED	3	1	1	0	1	0	2	1	0	0	1	0
MUSCULOSKELETAL STIFFNESS	222	149	34	30	6	3	207	149	28	22	5	3
BACK PAIN	194	133	29	19	0	13	181	133	22	14	0	12
FLANK PAIN	1	1	0	0	0	0	1	1	0	0	0	0
LIMB DISCOMFORT	623	399	121	73	2	28	595	399	102	64	2	28
MUSCULOSKELETAL CHEST PAIN	10	4	2	0	0	4	10	4	2	0	0	4
MUSCULOSKELETAL DISCOMFORT	17	10	3	4	0	0	16	10	2	4	0	0
MUSCULOSKELETAL PAIN	5	1	3	1	0	0	5	1	3	1	0	0
NECK PAIN	109	71	20	10	2	6	107	71	19	9	2	6
PAIN IN EXTREMITY	3757	2409	599	476	75	198	3502	2409	453	400	58	182
SOFT TISSUE DISORDERS NEC												
AXILLARY MASS	1	0	0	1	0	0	0	0	0	0	0	0
GROIN PAIN	5	3	1	0	0	1	5	3	1	0	0	1
SYNOVIAL AND BURSAL DISORDERS												
SYNOVIAL DISORDERS												

SYNOVITIS	1	0	1	0	0	0	1	0	1	0	0	0
TENDON, LIGAMENT AND CARTILAGE DISORDERS												
CARTILAGE DISORDERS												
COSTOCHONDRITIS	2	2	0	0	0	0	2	2	0	0	0	0
TENDON DISORDERS												
TENDONITIS	1	1	0	0	0	0	1	1	0	0	0	0
TENOSYNOVITIS	1	0	1	0	0	0	1	0	1	0	0	0
TRIGGER FINGER	1	0	0	1	0	0	1	0	0	1	0	0
**NEOPLASMS BENIGN, MALIGNANT AND UNSPECIFIED (INCL CYSTS AND POLYPS)**												
CUTANEOUS NEOPLASMS BENIGN												
SKIN NEOPLASMS BENIGN												
MELANOCYTIC NAEVUS	1	0	0	0	0	1	1	0	0	0	0	1
SEBORRHOEIC KERATOSIS	1	0	0	1	0	0	1	0	0	1	0	0
SKIN PAPILLOMA	1	1	0	0	0	0	1	1	0	0	0	0
**NERVOUS SYSTEM DISORDERS**												
CENTRAL NERVOUS SYSTEM INFECTIONS AND INFLAMMATIONS												
MYELITIS (INCL INFECTIVE)												
MYELITIS TRANSVERSE	1	1	0	0	0	0	1	1	0	0	0	0
CENTRAL NERVOUS SYSTEM VASCULAR DISORDERS												
CENTRAL NERVOUS SYSTEM HAEMORRHAGES AND CEREBROVASCULAR ACCIDENTS												
CEREBRAL HAEMORRHAGE	1	0	1	0	0	0	1	0	1	0	0	0
CEREBROVASCULAR ACCIDENT	6	4	1	1	0	0	6	4	1	1	0	0
TRANSIENT CEREBROVASCULAR EVENTS												
TRANSIENT ISCHAEMIC ATTACK	5	4	1	0	0	0	4	4	0	0	0	0
CRANIAL NERVE DISORDERS (EXCL NEOPLASMS)												
FACIAL CRANIAL NERVE DISORDERS												
BELL'S PALSY	2	1	1	0	0	0	2	1	1	0	0	0
FACIAL PARALYSIS	3	3	0	0	0	0	3	3	0	0	0	0
FACIAL PARESIS	1	0	0	1	0	0	1	0	0	1	0	0
OLFACTORY NERVE DISORDERS												
ANOSMIA	14	10	1	1	0	2	14	10	1	1	0	2
HYPOSMIA	1	1	0	0	0	0	1	1	0	0	0	0
PAROSMIA	16	9	6	1	0	0	15	9	5	1	0	0
TRIGEMINAL DISORDERS												
TRIGEMINAL NEURALGIA	3	3	0	0	0	0	3	3	0	0	0	0
TRIGEMINAL NEURITIS	1	0	0	1	0	0	1	0	0	1	0	0
DEMYELINATING DISORDERS												
MULTIPLE SCLEROSIS ACUTE AND PROGRESSIVE												

MULTIPLE SCLEROSIS RELAPSE	1	1	0	0	0	0	1	1	0	0	0	0
HEADACHES												
HEADACHES NEC												
CLUSTER HEADACHE	29	19	3	2	1	4	27	19	1	2	1	4
COLD-STIMULUS HEADACHE	1	1	0	0	0	0	1	1	0	0	0	0
DRUG WITHDRAWAL HEADACHE	1	0	1	0	0	0	1	0	1	0	0	0
HEADACHE	5317	3764	792	413	55	293	4969	3764	547	337	47	274
PRIMARY STABBING HEADACHE	3	3	0	0	0	0	3	3	0	0	0	0
SINUS HEADACHE	68	50	11	4	2	1	62	50	8	3	0	1
TENSION HEADACHE	126	87	22	11	4	2	119	87	17	9	4	2
THUNDERCLAP HEADACHE	1	1	0	0	0	0	1	1	0	0	0	0
VASCULAR HEADACHE	2	2	0	0	0	0	2	2	0	0	0	0
MIGRAINE HEADACHES												
MIGRAINE	154	113	22	13	0	6	144	113	15	10	0	6
MIGRAINE WITH AURA	8	5	3	0	0	0	7	5	2	0	0	0
RETINAL MIGRAINE	3	3	0	0	0	0	3	3	0	0	0	0
TYPICAL AURA WITHOUT HEADACHE	1	0	1	0	0	0	1	0	1	0	0	0
MENTAL IMPAIRMENT DISORDERS												
INTELLECTUAL DISABILITIES												
INTELLECTUAL DISABILITY	2	0	0	2	0	0	2	0	0	2	0	0
MEMORY LOSS (EXCL DEMENTIA)												
AMNESIA	4	2	1	0	0	1	4	2	1	0	0	1
MEMORY IMPAIRMENT	10	7	2	1	0	0	9	7	1	1	0	0
MENTAL IMPAIRMENT (EXCL DEMENTIA AND MEMORY LOSS)												
COGNITIVE DISORDER	3	2	0	1	0	0	3	2	0	1	0	0
DISTURBANCE IN ATTENTION	16	12	3	0	0	1	16	12	3	0	0	1
MENTAL IMPAIRMENT	4	3	0	1	0	0	3	3	0	0	0	0
MOVEMENT DISORDERS (INCL PARKINSONISM)												
DYSKINESIAS AND MOVEMENT DISORDERS NEC												
BRADYKINESIA	2	1	0	1	0	0	2	1	0	1	0	0
CLUMSINESS	1	1	0	0	0	0	1	1	0	0	0	0
DYSKINESIA	3	3	0	0	0	0	3	3	0	0	0	0
EXTRAPYRAMIDAL DISORDER	1	1	0	0	0	0	1	1	0	0	0	0
PSYCHOMOTOR HYPERACTIVITY	1	0	1	0	0	0	1	0	1	0	0	0
PARALYSIS AND PARESIS (EXCL CRANIAL NERVE)												
HEMIPLEGIA	2	0	0	1	0	1	2	0	0	1	0	1
MONOPARESIS	4	2	0	1	0	1	2	2	0	0	0	0
MONOPLEGIA	1	1	0	0	0	0	1	1	0	0	0	0

PARALYSIS	4	2	0	1	0	1	4	2	0	1	0	1
PARKINSON'S DISEASE AND PARKINSONISM												
FREEZING PHENOMENON	3	2	0	1	0	0	3	2	0	1	0	0
PARKINSON'S DISEASE	1	1	0	0	0	0	1	1	0	0	0	0
TREMOR (EXCL CONGENITAL)												
TREMOR	162	113	16	10	1	22	151	113	9	7	1	21
NEUROLOGICAL DISORDERS NEC												
COMA STATES												
DIABETIC HYPERGLYCAEMIC COMA	1	0	0	0	0	1	0	0	0	0	0	0
COORDINATION AND BALANCE DISTURBANCES												
BALANCE DISORDER	26	17	3	2	1	3	26	17	3	2	1	3
COORDINATION ABNORMAL	2	1	1	0	0	0	2	1	1	0	0	0
DYSSTASIA	1	1	0	0	0	0	1	1	0	0	0	0
VESTIBULAR NYSTAGMUS	1	1	0	0	0	0	1	1	0	0	0	0
DISTURBANCES IN CONSCIOUSNESS NEC												
DEPRESSED LEVEL OF CONSCIOUSNESS	1	0	0	0	0	1	1	0	0	0	0	1
LETHARGY	322	204	62	31	6	19	296	204	47	26	2	17
LOSS OF CONSCIOUSNESS	11	7	2	1	0	1	11	7	2	1	0	1
SEDATION	1	0	0	0	1	0	1	0	0	0	1	0
SOMNOLENCE	146	95	30	9	0	12	138	95	26	7	0	10
SYNCOPE	41	24	10	3	0	4	34	24	4	2	0	4
NERVOUS SYSTEM DISORDERS NEC												
NERVOUS SYSTEM DISORDER	1	0	0	0	0	1	1	0	0	0	0	1
NEUROLOGICAL SIGNS AND SYMPTOMS NEC												
AGITATION NEONATAL	1	0	0	0	0	1	0	0	0	0	0	0
BRAIN FOG	33	21	6	1	0	5	32	21	5	1	0	5
DIZZINESS	655	443	125	38	9	40	617	443	98	31	6	39
DIZZINESS EXERTIONAL	7	5	2	0	0	0	6	5	1	0	0	0
DIZZINESS POSTURAL	93	65	18	4	1	5	87	65	13	4	1	4
HEAD DISCOMFORT	17	11	4	1	0	1	16	11	4	1	0	0
INFANT IRRITABILITY	1	0	1	0	0	0	1	0	1	0	0	0
MENINGISM	1	1	0	0	0	0	1	1	0	0	0	0
MYOCLONUS	1	1	0	0	0	0	1	1	0	0	0	0
NEUROLOGICAL SYMPTOM	1	1	0	0	0	0	1	1	0	0	0	0
PERSISTENT POSTURAL-PERCEPTUAL DIZZINESS	1	0	1	0	0	0	0	0	0	0	0	0
PRESYNCOPE	17	14	0	3	0	0	17	14	0	3	0	0
PARAESTHESIAS AND DYSAESTHESIAS												
BURNING FEET SYNDROME	1	1	0	0	0	0	1	1	0	0	0	0

BURNING SENSATION	7	3	0	0	0	4	7	3	0	0	0	4
FORMICATION	1	0	1	0	0	0	1	0	1	0	0	0
HYPERAESTHESIA	1	1	0	0	0	0	1	1	0	0	0	0
HYPOAESTHESIA	112	96	8	5	0	3	107	96	4	5	0	2
PARAESTHESIA	157	114	26	4	2	11	152	114	22	3	2	11
REVERSED HOT-COLD SENSATION	1	1	0	0	0	0	1	1	0	0	0	0
SENSORY ABNORMALITIES NEC												
AGEUSIA	32	21	5	1	1	4	31	21	4	1	1	4
ALLODYNIA	2	1	1	0	0	0	1	1	0	0	0	0
DYSGEUSIA	70	49	14	5	1	1	67	49	11	5	1	1
HYPOGEUSIA	1	1	0	0	0	0	1	1	0	0	0	0
NEURALGIA	25	18	5	1	0	1	22	18	3	0	0	1
POST HERPETIC NEURALGIA	1	0	0	1	0	0	1	0	0	1	0	0
RESTLESS ARM SYNDROME	1	1	0	0	0	0	1	1	0	0	0	0
RESTLESS LEGS SYNDROME	5	4	0	0	0	1	5	4	0	0	0	1
SENSORY LOSS	2	2	0	0	0	0	2	2	0	0	0	0
TASTE DISORDER	17	10	5	1	0	1	16	10	4	1	0	1
SPEECH AND LANGUAGE ABNORMALITIES												
DYSARTHRIA	1	1	0	0	0	0	1	1	0	0	0	0
SPEECH DISORDER DEVELOPMENTAL	1	0	0	1	0	0	1	0	0	1	0	0
NEUROLOGICAL DISORDERS OF THE EYE												
NEUROLOGIC VISUAL PROBLEMS NEC												
TUNNEL VISION	2	1	1	0	0	0	2	1	1	0	0	0
NEUROMUSCULAR DISORDERS												
MUSCLE TONE ABNORMAL												
HYPOTONIA	1	1	0	0	0	0	1	1	0	0	0	0
STIFF LEG SYNDROME	1	0	1	0	0	0	1	0	1	0	0	0
NEUROMUSCULAR DISORDERS NEC												
MUSCLE SPASTICITY	1	1	0	0	0	0	1	1	0	0	0	0
PERIPHERAL NEUROPATHIES												
ACUTE POLYNEUROPATHIES												
GUILLAIN-BARRE SYNDROME	2	2	0	0	0	0	2	2	0	0	0	0
PERIPHERAL NEUROPATHIES NEC												
AXONAL NEUROPATHY	1	1	0	0	0	0	1	1	0	0	0	0
NEUROPATHY PERIPHERAL	3	1	0	2	0	0	3	1	0	2	0	0
SEIZURES (INCL SUBTYPES)												
SEIZURES AND SEIZURE DISORDERS NEC												
EPILEPSY	1	0	0	1	0	0	1	0	0	1	0	0

SEIZURE	10	8	1	1	0	0	10	8	1	1	0	0
SLEEP DISTURBANCES (INCL SUBTYPES)												
DISTURBANCES IN SLEEP PHASE RHYTHM												
CIRCADIAN RHYTHM SLEEP DISORDER	1	1	0	0	0	0	1	1	0	0	0	0
SLEEP DISTURBANCES NEC												
SLEEP DEFICIT	2	2	0	0	0	0	2	2	0	0	0	0
SPINAL CORD AND NERVE ROOT DISORDERS												
CERVICAL SPINAL CORD AND NERVE ROOT DISORDERS												
CERVICOBRACHIAL SYNDROME	1	0	0	1	0	0	1	0	0	1	0	0
LUMBAR SPINAL CORD AND NERVE ROOT DISORDERS												
CAUDA EQUINA SYNDROME	1	0	0	1	0	0	1	0	0	1	0	0
SCIATICA	8	4	1	2	0	1	8	4	1	2	0	1
**PREGNANCY, PUERPERIUM AND PERINATAL CONDITIONS**												
ABORTIONS AND STILLBIRTH												
ABORTIONS SPONTANEOUS												
ABORTION SPONTANEOUS	23	13	10	0	0	0	20	13	7	0	0	0
STILLBIRTH AND FOETAL DEATH												
FOETAL DEATH	1	1	0	0	0	0	1	1	0	0	0	0
FOETAL COMPLICATIONS												
FOETAL COMPLICATIONS NEC												
FOETAL DISORDER	1	1	0	0	0	0	1	1	0	0	0	0
FOETAL HYPOKINESIA	2	1	0	1	0	0	1	1	0	0	0	0
FOETAL GROWTH COMPLICATIONS												
FOETAL MACROSOMIA	1	1	0	0	0	0	1	1	0	0	0	0
MATERNAL COMPLICATIONS OF PREGNANCY												
MATERNAL COMPLICATIONS OF PREGNANCY NEC												
MORNING SICKNESS	3	2	0	0	0	1	2	2	0	0	0	0
PLACENTAL, AMNIOTIC AND CAVITY DISORDERS (EXCL HAEMORRHAGES)												
PLACENTAL ABNORMALITIES (EXCL NEOPLASMS)												
PLACENTAL INFARCTION	1	1	0	0	0	0	1	1	0	0	0	0
PREGNANCY, LABOUR, DELIVERY AND POSTPARTUM CONDITIONS												
NORMAL PREGNANCY, LABOUR AND DELIVERY												
PREGNANCY	1	0	0	0	0	1	0	0	0	0	0	0
UTERINE CONTRACTIONS DURING PREGNANCY	1	1	0	0	0	0	1	1	0	0	0	0
**PRODUCT ISSUES**												
DEVICE ISSUES												
DEVICE MALFUNCTION EVENTS NEC												
OVERSENSING	1	1	0	0	0	0	1	1	0	0	0	0

**PSYCHIATRIC DISORDERS**												
ANXIETY DISORDERS AND SYMPTOMS												
ANXIETY SYMPTOMS												
AGITATION	3	1	2	0	0	0	3	1	2	0	0	0
ANXIETY	23	13	4	2	0	4	22	13	3	2	0	4
NERVOUSNESS	14	11	1	0	0	2	14	11	1	0	0	2
TENSION	6	5	1	0	0	0	5	5	0	0	0	0
PANIC ATTACKS AND DISORDERS												
PANIC ATTACK	4	3	0	0	0	1	4	3	0	0	0	1
CHANGES IN PHYSICAL ACTIVITY												
INCREASED PHYSICAL ACTIVITY LEVELS												
RESTLESSNESS	9	6	1	0	1	1	9	6	1	0	1	1
STEREOTYPIES AND AUTOMATISMS												
BRUXISM	1	1	0	0	0	0	1	1	0	0	0	0
COGNITIVE AND ATTENTION DISORDERS AND DISTURBANCES												
COGNITIVE AND ATTENTION DISORDERS AND DISTURBANCES NEC												
DAYDREAMING	1	1	0	0	0	0	1	1	0	0	0	0
MENTAL FATIGUE	30	19	6	2	0	3	26	19	3	1	0	3
COMMUNICATION DISORDERS AND DISTURBANCES												
SPEECH ARTICULATION AND RHYTHM DISTURBANCES												
DYSPHEMIA	1	0	1	0	0	0	0	0	0	0	0	0
DELIRIA (INCL CONFUSION)												
CONFUSION AND DISORIENTATION												
CONFUSIONAL STATE	34	27	4	2	1	0	31	27	2	1	1	0
DISORIENTATION	7	5	1	0	0	1	7	5	1	0	0	1
DELIRIA												
DELIRIUM	2	1	0	0	0	1	2	1	0	0	0	1
DEPRESSED MOOD DISORDERS AND DISTURBANCES												
DEPRESSIVE DISORDERS												
DEPRESSION	12	10	2	0	0	0	11	10	1	0	0	0
MOOD ALTERATIONS WITH DEPRESSIVE SYMPTOMS												
DEPRESSED MOOD	16	10	5	1	0	0	16	10	5	1	0	0
TEARFULNESS	1	1	0	0	0	0	1	1	0	0	0	0
DISSOCIATIVE DISORDERS												
DISSOCIATIVE STATES												
DISSOCIATION	3	1	0	2	0	0	3	1	0	2	0	0
DISTURBANCES IN THINKING AND PERCEPTION												
DELUSIONAL SYMPTOMS												

DELUSION	1	0	0	0	0	1	1	0	0	0	0	1
HALLUCINATIONS (EXCL SLEEP-RELATED)												
HALLUCINATION	5	4	0	0	0	1	5	4	0	0	0	1
PERCEPTION DISTURBANCES NEC												
DEREALISATION	2	2	0	0	0	0	2	2	0	0	0	0
THINKING DISTURBANCES												
BRADYPHRENIA	4	1	2	1	0	0	4	1	2	1	0	0
THOUGHT BLOCKING	1	1	0	0	0	0	1	1	0	0	0	0
MOOD DISORDERS AND DISTURBANCES NEC												
AFFECT ALTERATIONS NEC												
AFFECT LABILITY	1	0	1	0	0	0	1	0	1	0	0	0
INAPPROPRIATE AFFECT	2	2	0	0	0	0	2	2	0	0	0	0
EMOTIONAL AND MOOD DISTURBANCES NEC												
ANGER	1	1	0	0	0	0	1	1	0	0	0	0
EMOTIONAL DISORDER	1	0	1	0	0	0	1	0	1	0	0	0
EMOTIONAL DISTRESS	1	1	0	0	0	0	1	1	0	0	0	0
EUPHORIC MOOD	2	2	0	0	0	0	2	2	0	0	0	0
IRRITABILITY	14	13	0	0	0	1	14	13	0	0	0	1
MOOD ALTERED	3	0	2	1	0	0	2	0	1	1	0	0
FLUCTUATING MOOD SYMPTOMS												
MOOD SWINGS	3	1	2	0	0	0	2	1	1	0	0	0
MOOD DISORDERS NEC												
APATHY	3	1	1	1	0	0	3	1	1	1	0	0
LISTLESS	3	1	1	0	1	0	3	1	1	0	1	0
PERSONALITY DISORDERS AND DISTURBANCES IN BEHAVIOUR												
BEHAVIOUR AND SOCIALISATION DISTURBANCES												
AGGRESSION	1	1	0	0	0	0	1	1	0	0	0	0
PSYCHIATRIC AND BEHAVIOURAL SYMPTOMS NEC												
PSYCHIATRIC SYMPTOMS NEC												
PSYCHIATRIC SYMPTOM	1	0	0	0	0	1	1	0	0	0	0	1
PSYCHIATRIC DISORDERS NEC												
MENTAL DISORDERS NEC												
MENTAL DISORDER	1	0	1	0	0	0	1	0	1	0	0	0
SCHIZOPHRENIA AND OTHER PSYCHOTIC DISORDERS												
PSYCHOTIC DISORDER NEC												
PSYCHOTIC DISORDER	1	1	0	0	0	0	1	1	0	0	0	0
SEXUAL DYSFUNCTIONS, DISTURBANCES AND GENDER IDENTITY DISORDERS												
SEXUAL DESIRE DISORDERS												

LOSS OF LIBIDO	3	3	0	0	0	0	3	3	0	0	0	0
SLEEP DISORDERS AND DISTURBANCES												
DISTURBANCES IN INITIATING AND MAINTAINING SLEEP												
INITIAL INSOMNIA	1	1	0	0	0	0	1	1	0	0	0	0
INSOMNIA	118	88	16	9	1	4	110	88	11	7	0	4
DYSSOMNIAS												
BREATHING-RELATED SLEEP DISORDER	1	1	0	0	0	0	1	1	0	0	0	0
POOR QUALITY SLEEP	37	30	4	3	0	0	36	30	3	3	0	0
PARASOMNIAS												
ABNORMAL DREAMS	18	11	2	1	1	3	17	11	2	1	0	3
EXPLODING HEAD SYNDROME	1	0	1	0	0	0	1	0	1	0	0	0
NIGHTMARE	13	7	3	2	0	1	13	7	3	2	0	1
SLEEP DISORDERS NEC												
SLEEP DISORDER	13	11	2	0	0	0	13	11	2	0	0	0
SOMATIC SYMPTOM AND RELATED DISORDERS												
SOMATIC SYMPTOM DISORDERS												
HABIT COUGH	4	3	0	1	0	0	4	3	0	1	0	0
SUICIDAL AND SELF-INJURIOUS BEHAVIOURS NEC												
SUICIDAL AND SELF-INJURIOUS BEHAVIOUR												
SUICIDAL IDEATION	1	1	0	0	0	0	1	1	0	0	0	0
**RENAL AND URINARY DISORDERS**												
RENAL DISORDERS (EXCL NEPHROPATHIES)												
RENAL FAILURE AND IMPAIRMENT												
RENAL FAILURE	1	1	0	0	0	0	1	1	0	0	0	0
URINARY TRACT SIGNS AND SYMPTOMS												
BLADDER AND URETHRAL SYMPTOMS												
BLADDER PAIN	2	1	0	0	0	1	2	1	0	0	0	1
MICTURITION URGENCY	3	1	0	2	0	0	3	1	0	2	0	0
POLLAKIURIA	12	10	1	0	0	1	12	10	1	0	0	1
URINARY INCONTINENCE	1	0	1	0	0	0	0	0	0	0	0	0
URINARY ABNORMALITIES												
HAEMATURIA	1	1	0	0	0	0	1	1	0	0	0	0
URINE ABNORMALITY	1	0	0	1	0	0	1	0	0	1	0	0
URINE ODOUR ABNORMAL	2	2	0	0	0	0	2	2	0	0	0	0
URINARY TRACT SIGNS AND SYMPTOMS NEC												
POLYURIA	3	2	0	0	1	0	3	2	0	0	1	0
RENAL PAIN	8	5	2	0	0	1	8	5	2	0	0	1
**REPRODUCTIVE SYSTEM AND BREAST DISORDERS**												

BREAST DISORDERS												
BREAST DISORDERS NEC												
BREAST MASS	4	3	0	0	0	1	4	3	0	0	0	1
NIPPLE ENLARGEMENT	1	1	0	0	0	0	1	1	0	0	0	0
BREAST SIGNS AND SYMPTOMS												
BREAST DISCHARGE	1	0	0	0	0	1	1	0	0	0	0	1
BREAST PAIN	16	7	4	3	0	2	14	7	3	2	0	2
BREAST SWELLING	1	1	0	0	0	0	1	1	0	0	0	0
BREAST TENDERNESS	1	1	0	0	0	0	1	1	0	0	0	0
NIPPLE PAIN	2	2	0	0	0	0	2	2	0	0	0	0
MENOPAUSE RELATED CONDITIONS												
MENOPAUSAL EFFECTS ON THE GENITOURINARY TRACT												
POSTMENOPAUSAL HAEMORRHAGE	1	1	0	0	0	0	1	1	0	0	0	0
MENSTRUAL CYCLE AND UTERINE BLEEDING DISORDERS												
MENSTRUATION AND UTERINE BLEEDING NEC												
DYSMENORRHOEA	29	20	4	2	0	3	25	20	2	0	0	3
INTERMENSTRUAL BLEEDING	13	9	1	2	0	1	12	9	1	1	0	1
MENSTRUAL DISORDER	22	12	2	7	0	1	22	12	2	7	0	1
MENSTRUATION IRREGULAR	47	27	11	2	0	7	43	27	8	2	0	6
PREMENSTRUAL PAIN	2	1	0	0	0	1	2	1	0	0	0	1
RETROGRADE MENSTRUATION	1	1	0	0	0	0	1	1	0	0	0	0
MENSTRUATION WITH DECREASED BLEEDING												
AMENORRHOEA	1	1	0	0	0	0	1	1	0	0	0	0
HYPOMENORRHOEA	9	4	2	0	0	3	8	4	1	0	0	3
MENSTRUATION DELAYED	47	23	11	1	0	12	43	23	8	1	0	11
OLIGOMENORRHOEA	3	1	1	0	0	1	3	1	1	0	0	1
MENSTRUATION WITH INCREASED BLEEDING												
HEAVY MENSTRUAL BLEEDING	61	37	8	7	0	9	57	37	7	6	0	7
POLYMENORRHOEA	9	8	0	1	0	0	9	8	0	1	0	0
PENILE AND SCROTAL DISORDERS (EXCL INFECTIONS AND INFLAMMATIONS)												
SCROTAL DISORDERS NEC												
SCROTAL SWELLING	1	0	1	0	0	0	1	0	1	0	0	0
REPRODUCTIVE TRACT DISORDERS NEC												
REPRODUCTIVE TRACT DISORDERS NEC (EXCL NEOPLASMS)												
GENITAL LESION	1	1	0	0	0	0	1	1	0	0	0	0
REPRODUCTIVE TRACT SIGNS AND SYMPTOMS NEC												
GENITAL DISCOMFORT	1	1	0	0	0	0	1	1	0	0	0	0
GENITAL PAIN	1	1	0	0	0	0	1	1	0	0	0	0

UTERINE, PELVIC AND BROAD LIGAMENT DISORDERS												
PELVIS AND BROAD LIGAMENT DISORDERS NEC												
ADNEXA UTERI PAIN	1	1	0	0	0	0	1	1	0	0	0	0
VULVOVAGINAL DISORDERS (EXCL INFECTIONS AND INFLAMMATIONS)												
VULVOVAGINAL DISORDERS NEC												
VAGINAL HAEMORRHAGE	13	7	5	1	0	0	9	7	2	0	0	0
VULVOVAGINAL SIGNS AND SYMPTOMS												
VAGINAL DISCHARGE	1	1	0	0	0	0	1	1	0	0	0	0
**RESPIRATORY, THORACIC AND MEDIASTINAL DISORDERS**												
BRONCHIAL DISORDERS (EXCL NEOPLASMS)												
BRONCHOSPASM AND OBSTRUCTION												
ASTHMA	6	4	2	0	0	0	5	4	1	0	0	0
WHEEZING	12	8	2	1	0	1	10	8	1	0	0	1
LOWER RESPIRATORY TRACT DISORDERS (EXCL OBSTRUCTION AND INFECTION)												
PULMONARY OEDEMAS												
PULMONARY CONGESTION	1	1	0	0	0	0	1	1	0	0	0	0
PULMONARY VASCULAR DISORDERS												
PULMONARY THROMBOTIC AND EMBOLIC CONDITIONS												
PULMONARY EMBOLISM	2	1	0	0	0	1	2	1	0	0	0	1
RESPIRATORY DISORDERS NEC												
BREATHING ABNORMALITIES												
DYSPNOEA	129	79	20	11	5	14	124	79	17	10	5	13
HYPERVENTILATION	2	2	0	0	0	0	2	2	0	0	0	0
HYPOPNOEA	7	4	1	1	0	1	7	4	1	1	0	1
IRREGULAR BREATHING	1	1	0	0	0	0	1	1	0	0	0	0
MOUTH BREATHING	1	1	0	0	0	0	1	1	0	0	0	0
RESPIRATION ABNORMAL	1	1	0	0	0	0	1	1	0	0	0	0
RESPIRATORY ARREST	1	1	0	0	0	0	1	1	0	0	0	0
RESPIRATORY FATIGUE	2	2	0	0	0	0	2	2	0	0	0	0
SLEEP APNOEA SYNDROME	1	0	1	0	0	0	1	0	1	0	0	0
COUGHING AND ASSOCIATED SYMPTOMS												
COUGH	132	87	20	16	3	6	126	87	18	14	1	6
PRODUCTIVE COUGH	9	3	4	0	0	2	8	3	4	0	0	1
RESPIRATORY TRACT DISORDERS NEC												
RESPIRATORY TRACT IRRITATION	1	1	0	0	0	0	1	1	0	0	0	0
RESPIRATORY TRACT SIGNS AND SYMPTOMS												
LOWER RESPIRATORY TRACT SIGNS AND SYMPTOMS												
PULMONARY PAIN	3	3	0	0	0	0	3	3	0	0	0	0

RESPIRATORY SIGNS AND SYMPTOMS NEC												
RESPIRATORY SYMPTOM	1	1	0	0	0	0	1	1	0	0	0	0
UPPER RESPIRATORY TRACT SIGNS AND SYMPTOMS												
APHONIA	3	2	0	1	0	0	3	2	0	1	0	0
CATARRH	7	3	1	2	0	1	6	3	1	1	0	1
DRY THROAT	11	7	3	0	0	1	9	7	1	0	0	1
DYSPHONIA	7	7	0	0	0	0	7	7	0	0	0	0
INCREASED UPPER AIRWAY SECRETION	1	1	0	0	0	0	1	1	0	0	0	0
INCREASED VISCOSITY OF UPPER RESPIRATORY SE(	1	0	0	1	0	0	1	0	0	1	0	0
NASAL DISCOMFORT	1	1	0	0	0	0	1	1	0	0	0	0
OROPHARYNGEAL DISCOMFORT	2	2	0	0	0	0	2	2	0	0	0	0
OROPHARYNGEAL PAIN	238	174	40	11	2	11	230	174	33	10	2	11
PARANASAL SINUS DISCOMFORT	3	3	0	0	0	0	3	3	0	0	0	0
RHINALGIA	2	1	1	0	0	0	2	1	1	0	0	0
RHINORRHOEA	126	76	25	15	2	8	112	76	15	12	1	8
SINUS PAIN	19	13	5	1	0	0	19	13	5	1	0	0
SNEEZING	31	22	5	1	1	2	30	22	4	1	1	2
THROAT CLEARING	1	1	0	0	0	0	1	1	0	0	0	0
THROAT IRRITATION	4	3	0	0	0	1	4	3	0	0	0	1
THROAT TIGHTNESS	5	4	1	0	0	0	5	4	1	0	0	0
UPPER-AIRWAY COUGH SYNDROME	1	0	0	1	0	0	1	0	0	1	0	0
YAWNING	3	2	0	0	0	1	3	2	0	0	0	1
UPPER RESPIRATORY TRACT DISORDERS (EXCL INFECTIONS)												
NASAL CONGESTION AND INFLAMMATIONS												
NASAL CONGESTION	18	11	6	1	0	0	16	11	4	1	0	0
RHINITIS ALLERGIC	2	2	0	0	0	0	2	2	0	0	0	0
RHINITIS ATROPHIC	1	0	1	0	0	0	1	0	1	0	0	0
NASAL DISORDERS NEC												
EPISTAXIS	32	21	9	2	0	0	29	21	8	0	0	0
NASAL DRYNESS	4	3	1	0	0	0	3	3	0	0	0	0
PARANASAL SINUS DISORDERS (EXCL INFECTIONS AND NEOPLASMS)												
SINUS CONGESTION	5	3	2	0	0	0	5	3	2	0	0	0
PHARYNGEAL DISORDERS (EXCL INFECTIONS AND NEOPLASMS)												
PHARYNGEAL SWELLING	3	2	1	0	0	0	3	2	1	0	0	0
PHARYNGEAL ULCERATION	1	1	0	0	0	0	1	1	0	0	0	0
TONSILLAR ERYTHEMA	1	0	0	0	0	1	1	0	0	0	0	1
TRACHEAL DISORDERS (EXCL INFECTIONS AND NEOPLASMS)												
TRACHEAL PAIN	1	1	0	0	0	0	1	1	0	0	0	0

**SKIN AND SUBCUTANEOUS TISSUE DISORDERS**												
ANGIOEDEMA AND URTICARIA												
ANGIOEDEMAS												
ANGIOEDEMA	3	3	0	0	0	0	3	3	0	0	0	0
URTICARIAS												
COLD URTICARIA	1	0	1	0	0	0	1	0	1	0	0	0
SOLAR URTICARIA	1	1	0	0	0	0	1	1	0	0	0	0
URTICARIA	21	12	2	7	0	0	21	12	2	7	0	0
URTICARIA CHRONIC	1	0	0	0	0	1	1	0	0	0	0	1
CORNIFICATION AND DYSTROPHIC SKIN DISORDERS												
SKIN DYSTROPHIES												
HYPERTROPHIC SCAR	1	1	0	0	0	0	1	1	0	0	0	0
CUTANEOUS NEOPLASMS BENIGN												
SKIN CYSTS AND POLYPS												
DERMAL CYST	1	1	0	0	0	0	1	1	0	0	0	0
EPIDERMAL AND DERMAL CONDITIONS												
BULLOUS CONDITIONS												
BLISTER	5	3	1	0	0	1	5	3	1	0	0	1
BLOOD BLISTER	1	0	0	0	0	1	1	0	0	0	0	1
PEMPHIGOID	1	0	0	0	1	0	1	0	0	0	1	0
TOXIC EPIDERMAL NECROLYSIS	2	2	0	0	0	0	2	2	0	0	0	0
DERMAL AND EPIDERMAL CONDITIONS NEC												
DRY SKIN	9	4	0	1	0	4	8	4	0	1	0	3
PAIN OF SKIN	37	26	3	4	1	3	35	26	2	4	0	3
SENSITIVE SKIN	28	22	2	1	1	2	26	22	1	1	0	2
SKIN BURNING SENSATION	13	9	1	1	0	2	13	9	1	1	0	2
SKIN DISCOLOURATION	1	1	0	0	0	0	1	1	0	0	0	0
SKIN FRAGILITY	1	1	0	0	0	0	1	1	0	0	0	0
SKIN LESION	1	0	0	0	0	1	0	0	0	0	0	0
SKIN ODOUR ABNORMAL	2	2	0	0	0	0	2	2	0	0	0	0
SKIN REACTION	3	2	0	1	0	0	3	2	0	1	0	0
SKIN SENSITISATION	1	1	0	0	0	0	1	1	0	0	0	0
SKIN WARM	33	23	4	5	0	1	31	23	2	5	0	1
DERMATITIS AND ECZEMA												
DERMATITIS	4	4	0	0	0	0	4	4	0	0	0	0
DERMATITIS ALLERGIC	4	4	0	0	0	0	4	4	0	0	0	0
DERMATITIS ATOPIC	2	2	0	0	0	0	2	2	0	0	0	0
DERMATITIS CONTACT	1	1	0	0	0	0	1	1	0	0	0	0

ECZEMA	6	3	1	0	0	2	5	3	1	0	0	1
ECZEMA ASTEATOTIC	1	0	0	1	0	0	1	0	0	1	0	0
NEURODERMATITIS	1	1	0	0	0	0	1	1	0	0	0	0
SEBORRHOEIC DERMATITIS	2	2	0	0	0	0	2	2	0	0	0	0
SKIN IRRITATION	9	6	2	1	0	0	8	6	1	1	0	0
ERYTHEMAS												
ERYTHEMA	59	39	11	3	1	5	54	39	9	2	0	4
EXFOLIATIVE CONDITIONS												
SKIN EXFOLIATION	2	1	0	0	1	0	1	1	0	0	0	0
PAPULOSQUAMOUS CONDITIONS												
LICHEN PLANUS	1	0	1	0	0	0	1	0	1	0	0	0
PITYRIASIS ROSEA	1	1	0	0	0	0	1	1	0	0	0	0
PHOTOSENSITIVITY AND PHOTODERMATOSIS CONDITIONS												
PHOTOSENSITIVITY REACTION	6	4	2	0	0	0	5	4	1	0	0	0
PRURITUS NEC												
PRURITUS	176	122	29	10	2	13	165	122	21	8	2	12
PSORIATIC CONDITIONS												
PSORIASIS	10	5	3	1	0	1	10	5	3	1	0	1
RASHES, ERUPTIONS AND EXANTHEMS NEC												
RASH	178	125	25	22	2	4	162	125	17	14	2	4
RASH ERYTHEMATOUS	39	26	3	6	0	4	37	26	2	6	0	3
RASH MACULAR	5	3	2	0	0	0	5	3	2	0	0	0
RASH MORBILLIFORM	1	0	0	1	0	0	0	0	0	0	0	0
RASH PAPULAR	13	10	2	0	0	1	13	10	2	0	0	1
RASH PRURITIC	36	22	4	5	2	3	35	22	4	5	1	3
SKIN INJURIES AND MECHANICAL DERMATOSES												
DECUBITUS ULCER	2	1	0	1	0	0	1	1	0	0	0	0
NEEDLE TRACK MARKS	1	1	0	0	0	0	1	1	0	0	0	0
SKIN AND SUBCUTANEOUS TISSUE DISORDERS NEC												
SKIN AND SUBCUTANEOUS TISSUE ULCERATIONS												
SKIN EROSION	2	1	1	0	0	0	1	1	0	0	0	0
SKIN APPENDAGE CONDITIONS												
ACNES												
ACNE	1	1	0	0	0	0	1	1	0	0	0	0
ACNE CYSTIC	1	0	1	0	0	0	1	0	1	0	0	0
ALOPECIAS												
ALOPECIA	14	6	2	1	0	5	12	6	1	1	0	4
APOCRINE AND ECCRINE GLAND DISORDERS												

COLD SWEAT	35	28	5	1	1	0	33	28	3	1	1	0
HYPERHIDROSIS	169	122	19	8	1	19	163	122	15	6	1	19
MILIARIA	6	3	2	0	0	1	5	3	1	0	0	1
NIGHT SWEATS	81	59	12	6	1	3	74	59	7	5	0	3
HYPERTRICHOSES												
HIRSUTISM	1	1	0	0	0	0	1	1	0	0	0	0
NAIL AND NAIL BED CONDITIONS (EXCL INFECTIONS AND INFESTATIONS)												
NAIL DISCOLOURATION	1	0	0	0	0	1	1	0	0	0	0	1
ONYCHOCLASIS	1	0	0	0	0	1	0	0	0	0	0	0
PILAR DISORDERS NEC												
PILOERECTION	3	2	1	0	0	0	2	2	0	0	0	0
SKIN VASCULAR ABNORMALITIES												
PURPURA AND RELATED CONDITIONS												
PETECHIAE	2	2	0	0	0	0	2	2	0	0	0	0
PURPURA	1	0	0	0	1	0	1	0	0	0	1	0
SKIN HAEMORRHAGES												
SKIN HAEMORRHAGE	1	1	0	0	0	0	1	1	0	0	0	0
**SOCIAL CIRCUMSTANCES**												
ECONOMIC AND HOUSING ISSUES												
EMPLOYMENT ISSUES												
RETIREMENT	2	2	0	0	0	0	2	2	0	0	0	0
ENVIRONMENTAL ISSUES												
NON-OCCUPATIONAL AND UNSPECIFIED ENVIRONMENTAL PROBLEMS												
POLLUTION	1	1	0	0	0	0	1	1	0	0	0	0
**SURGICAL AND MEDICAL PROCEDURES**												
BONE AND JOINT THERAPEUTIC PROCEDURES												
JOINT THERAPEUTIC PROCEDURES												
KNEE OPERATION	1	1	0	0	0	0	1	1	0	0	0	0
BREAST THERAPEUTIC PROCEDURES												
MASTECTOMIES												
BREAST CONSERVING SURGERY	1	0	1	0	0	0	1	0	1	0	0	0
GASTROINTESTINAL THERAPEUTIC PROCEDURES												
GASTROINTESTINAL THERAPEUTIC PROCEDURES NEC												
PROPHYLAXIS OF NAUSEA AND VOMITING	1	1	0	0	0	0	1	1	0	0	0	0
HEAD AND NECK THERAPEUTIC PROCEDURES												
DENTAL AND GINGIVAL THERAPEUTIC PROCEDURES												
DENTAL CARE	2	2	0	0	0	0	2	2	0	0	0	0
NERVOUS SYSTEM, SKULL AND SPINE THERAPEUTIC PROCEDURES												

SKULL AND BRAIN THERAPEUTIC PROCEDURES												
BRAIN TUMOUR OPERATION	1	1	0	0	0	0	1	1	0	0	0	0
OBSTETRIC AND GYNAECOLOGICAL THERAPEUTIC PROCEDURES												
FERTILITY AND FERTILISATION INTERVENTIONS FEMALE												
ENDOMETRIAL SCRATCHING	1	1	0	0	0	0	1	1	0	0	0	0
RESPIRATORY TRACT THERAPEUTIC PROCEDURES												
RESPIRATORY TRACT THERAPEUTIC PROCEDURES NEC												
OXYGEN THERAPY	1	0	0	0	0	1	1	0	0	0	0	1
THERAPEUTIC PROCEDURES AND SUPPORTIVE CARE NEC												
ANAESTHESIA AND ALLIED PROCEDURES												
NERVE BLOCK	2	2	0	0	0	0	2	2	0	0	0	0
DIETARY AND NUTRITIONAL THERAPIES												
NOTHING BY MOUTH ORDER	1	0	0	0	0	1	1	0	0	0	0	1
IMMUNISATIONS												
COVID-19 IMMUNISATION	1	0	1	0	0	0	0	0	0	0	0	0
THERAPEUTIC PROCEDURES NEC												
INJECTION	3	3	0	0	0	0	3	3	0	0	0	0
LOCALISED ALTERNATING HOT AND COLD THERAPY	2	2	0	0	0	0	2	2	0	0	0	0
MASS EXCISION	1	0	1	0	0	0	1	0	1	0	0	0
REINFUSION	1	0	0	1	0	0	1	0	0	1	0	0
**VASCULAR DISORDERS**												
ARTERIOSCLEROSIS, STENOSIS, VASCULAR INSUFFICIENCY AND NECROSIS												
NON-SITE SPECIFIC NECROSIS AND VASCULAR INSUFFICIENCY NEC												
VASOSPASM	1	1	0	0	0	0	1	1	0	0	0	0
PERIPHERAL VASOCONSTRICTION, NECROSIS AND VASCULAR INSUFFICIENCY												
PERIPHERAL COLDNESS	55	48	3	2	0	2	54	48	2	2	0	2
RAYNAUD'S PHENOMENON	1	1	0	0	0	0	1	1	0	0	0	0
DECREASED AND NONSPECIFIC BLOOD PRESSURE DISORDERS AND SHOCK												
BLOOD PRESSURE DISORDERS NEC												
BLOOD PRESSURE FLUCTUATION	1	1	0	0	0	0	1	1	0	0	0	0
VASCULAR HYPOTENSIVE DISORDERS												
CAPILLARY LEAK SYNDROME	1	1	0	0	0	0	1	1	0	0	0	0
HYPOTENSION	8	7	0	1	0	0	7	7	0	0	0	0
ORTHOSTATIC HYPOTENSION	2	1	1	0	0	0	2	1	1	0	0	0
EMBOLISM AND THROMBOSIS												
NON-SITE SPECIFIC EMBOLISM AND THROMBOSIS												
EMBOLISM	1	1	0	0	0	0	1	1	0	0	0	0
THROMBOSIS	11	9	1	0	0	1	11	9	1	0	0	1

VENOUS THROMBOSIS	1	0	1	0	0	0	0	0	0	0	0	0
PERIPHERAL EMBOLISM AND THROMBOSIS												
BLUE TOE SYNDROME	2	2	0	0	0	0	2	2	0	0	0	0
DEEP VEIN THROMBOSIS	5	3	1	0	0	1	4	3	0	0	0	1
SUPERFICIAL VEIN THROMBOSIS	1	0	0	0	0	1	1	0	0	0	0	1
LYMPHATIC VESSEL DISORDERS												
LYMPHOEDEMAS												
LYMPHOEDEMA	4	2	0	2	0	0	3	2	0	1	0	0
VASCULAR DISORDERS NEC												
NON-SITE SPECIFIC VASCULAR DISORDERS NEC												
VASCULAR PAIN	1	0	1	0	0	0	1	0	1	0	0	0
VEIN DISCOLOURATION	1	0	0	0	0	1	1	0	0	0	0	1
VEIN RUPTURE	1	0	1	0	0	0	1	0	1	0	0	0
PERIPHERAL VASCULAR DISORDERS NEC												
FLUSHING	30	24	4	1	0	1	28	24	2	1	0	1
HOT FLUSH	119	88	17	6	0	8	113	88	13	4	0	8
SITE SPECIFIC VASCULAR DISORDERS NEC												
PALLOR	9	5	2	0	0	2	7	5	0	0	0	2
VASCULAR HAEMORRHAGIC DISORDERS												
HAEMORRHAGES NEC												
HAEMATOMA	1	1	0	0	0	0	1	1	0	0	0	0
HAEMORRHAGE	7	3	1	2	0	1	6	3	0	2	0	1
VASCULAR HYPERTENSIVE DISORDERS												
ACCELERATED AND MALIGNANT HYPERTENSION												
HYPERTENSIVE URGENCY	1	1	0	0	0	0	1	1	0	0	0	0
VASCULAR HYPERTENSIVE DISORDERS NEC												
HYPERTENSION	15	9	4	1	0	1	14	9	3	1	0	1
SYSTOLIC HYPERTENSION	1	1	0	0	0	0	1	1	0	0	0	0
VASCULAR INFECTIONS AND INFLAMMATIONS												
ARTERIAL INFECTIONS AND INFLAMMATIONS												
GIANT CELL ARTERITIS	1	1	0	0	0	0	1	1	0	0	0	0
PHLEBITIS NEC												
PHLEBITIS	3	1	1	1	0	0	2	1	1	0	0	0
VASCULITIDES NEC												
VASCULITIS	1	0	0	0	1	0	1	0	0	0	1	0
VENOUS VARICES												
VARICOSE VEINS NEC												
SPIDER VEIN	1	1	0	0	0	0	1	1	0	0	0	0

VARICOSE VEIN	3	3	0	0	0	0	3	3	0	0	0	0
**TOTAL ADR EVENTS**	***38144***	***26096***	***5973***	***3446***	***527***	***2102***	***35647***	***26096***	***4317***	***2833***	***421***	***1980***

SUPPLEMENTARY TABLE 16. Pfizer BioNTech COVID-19 vaccine: ADR listing for events reported in the YCVM in (a) those reporting any vaccination dose and (b) in those who had reported a 1st dose vaccination and any

	Individuals with any vaccination dose: ADR Counts					Individuals reporting a 1st dose: ADR Counts				
**MEDDRA REACTION TERM (SOC, *HLGT*, *HLT*, PT)**	All doses	1st dose	2nd dose	3rd dose	Other doses	All doses	1st dose	2nd dose	3rd dose	Other doses
(freetext)	30	30	0	0	0	30	30	0	0	0
**BLOOD AND LYMPHATIC SYSTEM DISORDERS**										
COAGULOPATHIES AND BLEEDING DIATHESES (EXCL THROMBOCYTOPENIC)										
BLEEDING TENDENCIES										
INCREASED TENDENCY TO BRUISE	0	0	0	0	0	0	0	0	0	0
HAEMOGLOBINOPATHIES										
SICKLE CELL TRAIT AND DISORDERS										
SICKLE CELL ANAEMIA WITH CRISIS	0	0	0	0	0	0	0	0	0	0
PLATELET DISORDERS										
THROMBOCYTOPENIAS										
IMMUNE THROMBOCYTOPENIA	0	0	0	0	0	0	0	0	0	0
SPLEEN, LYMPHATIC AND RETICULOENDOTHELIAL SYSTEM DISORDERS										
LYMPHATIC SYSTEM DISORDERS NEC										
LYMPH NODE PAIN	18	4	7	7	0	13	4	5	4	0
LYMPHADENITIS	1	0	1	0	0	1	0	1	0	0
LYMPHADENOPATHY	98	45	21	31	1	88	45	16	26	1
**CARDIAC DISORDERS**										
CARDIAC ARRHYTHMIAS										
RATE AND RHYTHM DISORDERS NEC										
ARRHYTHMIA	0	0	0	0	0	0	0	0	0	0
CARDIAC FLUTTER	7	2	3	2	0	4	2	1	1	0
EXTRASYSTOLES	2	0	1	1	0	2	0	1	1	0
TACHYCARDIA	1	1	0	0	0	1	1	0	0	0
SUPRAVENTRICULAR ARRHYTHMIAS										
ATRIAL FIBRILLATION	0	0	0	0	0	0	0	0	0	0
SUPRAVENTRICULAR TACHYCARDIA	0	0	0	0	0	0	0	0	0	0
CARDIAC DISORDERS, SIGNS AND SYMPTOMS NEC										

CARDIAC DISORDERS NEC										
CARDIOVASCULAR DISORDER	0	0	0	0	0	0	0	0	0	0
CARDIAC SIGNS AND SYMPTOMS NEC										
PALPITATIONS	21	7	9	4	1	17	7	5	4	1
CORONARY ARTERY DISORDERS										
ISCHAEMIC CORONARY ARTERY DISORDERS										
ANGINA PECTORIS	1	1	0	0	0	1	1	0	0	0
MYOCARDIAL INFARCTION	1	1	0	0	0	1	1	0	0	0
HEART FAILURES										
HEART FAILURES NEC										
CARDIAC FAILURE	1	0	0	1	0	0	0	0	0	0
MYOCARDIAL DISORDERS										
NONINFECTIOUS MYOCARDITIS										
MYOCARDITIS	2	1	1	0	0	1	1	0	0	0
PERICARDIAL DISORDERS										
NONINFECTIOUS PERICARDITIS										
PERICARDITIS	1	1	0	0	0	1	1	0	0	0
**CONGENITAL, FAMILIAL AND GENETIC DISORDERS**										
CARDIAC AND VASCULAR DISORDERS CONGENITAL										
CARDIAC DISORDERS CONGENITAL NEC										
HEART DISEASE CONGENITAL	1	0	0	1	0	1	0	0	1	0
METABOLIC AND NUTRITIONAL DISORDERS CONGENITAL										
INBORN ERRORS OF AMINO ACID METABOLISM										
HYPERGLYCINAEMIA	0	0	0	0	0	0	0	0	0	0
INBORN ERRORS OF STEROID SYNTHESIS										
11-BETA-HYDROXYLASE DEFICIENCY	0	0	0	0	0	0	0	0	0	0
NEUROLOGICAL DISORDERS CONGENITAL										
PERIPHERAL NERVOUS SYSTEM DISORDERS CONGENITAL NEC										
PAROXYSMAL EXTREME PAIN DISORDER	0	0	0	0	0	0	0	0	0	0
**EAR AND LABYRINTH DISORDERS**										
AURAL DISORDERS NEC										
EAR DISORDERS NEC										
EAR DISCOMFORT	1	0	1	0	0	1	0	1	0	0

EAR DISORDER	0	0	0	0	0	0	0	0	0	0
EAR PAIN	9	7	1	1	0	8	7	1	0	0
EAR SWELLING	0	0	0	0	0	0	0	0	0	0
EXTERNAL EAR DISORDERS (EXCL CONGENITAL)										
EXTERNAL EAR DISORDERS NEC										
EXCESSIVE CERUMEN PRODUCTION	0	0	0	0	0	0	0	0	0	0
HEARING DISORDERS										
HEARING LOSSES										
DEAFNESS	3	2	1	0	0	3	2	1	0	0
DEAFNESS NEUROSENSORY	1	1	0	0	0	1	1	0	0	0
DEAFNESS PERMANENT	1	0	1	0	0	0	0	0	0	0
DEAFNESS UNILATERAL	0	0	0	0	0	0	0	0	0	0
HYPOACUSIS	1	1	0	0	0	1	1	0	0	0
SUDDEN HEARING LOSS	0	0	0	0	0	0	0	0	0	0
HYPERACUSIA										
HYPERACUSIS	0	0	0	0	0	0	0	0	0	0
INNER EAR AND VIIITH CRANIAL NERVE DISORDERS										
INNER EAR SIGNS AND SYMPTOMS										
MOTION SICKNESS	1	1	0	0	0	1	1	0	0	0
TINNITUS	18	9	4	5	0	17	9	4	4	0
VERTIGO	10	6	3	1	0	10	6	3	1	0
VERTIGO LABYRINTHINE	0	0	0	0	0	0	0	0	0	0
VERTIGO POSITIONAL	0	0	0	0	0	0	0	0	0	0
**ENDOCRINE DISORDERS**										
THYROID GLAND DISORDERS										
THYROID HYPERFUNCTION DISORDERS										
HYPERTHYROIDISM	0	0	0	0	0	0	0	0	0	0
**EYE DISORDERS**										
EYE DISORDERS NEC										
LACRIMATION DISORDERS										
DRY EYE	5	0	1	4	0	4	0	1	3	0
LACRIMATION INCREASED	3	2	0	1	0	2	2	0	0	0
OCULAR DISORDERS NEC										
EYE OEDEMA	0	0	0	0	0	0	0	0	0	0
EYE PAIN	22	14	3	5	0	22	14	3	5	0
EYE SWELLING	2	2	0	0	0	2	2	0	0	0
EYE ULCER	0	0	0	0	0	0	0	0	0	0
EYELID PAIN	0	0	0	0	0	0	0	0	0	0

OCULAR DISCOMFORT	0	0	0	0	0	0	0	0	0	0
PERIORBITAL DISCOMFORT	0	0	0	0	0	0	0	0	0	0
PERIORBITAL SWELLING	0	0	0	0	0	0	0	0	0	0
OCULAR HAEMORRHAGES AND VASCULAR DISORDERS NEC										
CONJUNCTIVAL AND CORNEAL BLEEDING AND VASCULAR DISORDERS										
CONJUNCTIVAL HAEMORRHAGE	1	1	0	0	0	1	1	0	0	0
LID BLEEDING AND VASCULAR DISORDERS										
EYELID BLEEDING	0	0	0	0	0	0	0	0	0	0
OCULAR INFECTIONS, IRRITATIONS AND INFLAMMATIONS										
LID, LASH AND LACRIMAL INFECTIONS, IRRITATIONS AND INFLAMMATIONS										
BLEPHARITIS	0	0	0	0	0	0	0	0	0	0
ERYTHEMA OF EYELID	0	0	0	0	0	0	0	0	0	0
EYELID IRRITATION	0	0	0	0	0	0	0	0	0	0
OCULAR INFECTIONS, INFLAMMATIONS AND ASSOCIATED MANIFESTATIONS										
EYE DISCHARGE	0	0	0	0	0	0	0	0	0	0
EYE IRRITATION	0	0	0	0	0	0	0	0	0	0
EYE PRURITUS	5	4	0	1	0	4	4	0	0	0
LIMBAL SWELLING	0	0	0	0	0	0	0	0	0	0
OCULAR HYPERAEMIA	2	1	1	0	0	2	1	1	0	0
OCULAR NEUROMUSCULAR DISORDERS										
EYELID MOVEMENT DISORDERS										
BLEPHAROSPASM	1	1	0	0	0	1	1	0	0	0
OCULAR SENSORY SYMPTOMS NEC										
OCULAR SENSATION DISORDERS										
ABNORMAL SENSATION IN EYE	2	1	1	0	0	2	1	1	0	0
ASTHENOPIA	11	6	2	3	0	8	6	0	2	0
FOREIGN BODY SENSATION IN EYES	0	0	0	0	0	0	0	0	0	0
PHOTOPHOBIA	1	1	0	0	0	1	1	0	0	0
OCULAR STRUCTURAL CHANGE, DEPOSIT AND DEGENERATION NEC										
CHOROID AND VITREOUS STRUCTURAL CHANGE, DEPOSIT AND DEGENERATION										
VITREOUS DETACHMENT	0	0	0	0	0	0	0	0	0	0
VITREOUS FLOATERS	0	0	0	0	0	0	0	0	0	0

RETINA, CHOROID AND VITREOUS HAEMORRHAGES AND VASCULAR DISORDERS										
RETINAL BLEEDING AND VASCULAR DISORDERS (EXCL RETINOPATHY)										
RETINAL VEIN OCCLUSION	1	0	1	0	0	1	0	1	0	0
RETINOPATHIES NEC										
RETINAL EXUDATES	0	0	0	0	0	0	0	0	0	0
VISION DISORDERS										
VISUAL DISORDERS NEC										
DIPLOPIA	0	0	0	0	0	0	0	0	0	0
HALO VISION	0	0	0	0	0	0	0	0	0	0
METAMORPHOPSIA	0	0	0	0	0	0	0	0	0	0
PHOTOPSIA	1	1	0	0	0	1	1	0	0	0
VISION BLURRED	10	6	4	0	0	9	6	3	0	0
VISUAL IMPAIRMENT AND BLINDNESS (EXCL COLOUR BLINDNESS)										
BLINDNESS	0	0	0	0	0	0	0	0	0	0
BLINDNESS TRANSIENT	0	0	0	0	0	0	0	0	0	0
SUDDEN VISUAL LOSS	0	0	0	0	0	0	0	0	0	0
VISUAL IMPAIRMENT	5	5	0	0	0	5	5	0	0	0
**GASTROINTESTINAL DISORDERS**										
ANAL AND RECTAL CONDITIONS NEC										
ANAL AND RECTAL SIGNS AND SYMPTOMS										
ANAL PARAESTHESIA	0	0	0	0	0	0	0	0	0	0
BENIGN NEOPLASMS GASTROINTESTINAL										
BENIGN ORAL CAVITY NEOPLASMS										
MOUTH CYST	0	0	0	0	0	0	0	0	0	0
DENTAL AND GINGIVAL CONDITIONS										
DENTAL DISORDERS NEC										
TEETHING	1	1	0	0	0	1	1	0	0	0
DENTAL PAIN AND SENSATION DISORDERS										
DENTAL PARAESTHESIA	0	0	0	0	0	0	0	0	0	0
TOOTHACHE	4	0	2	2	0	3	0	1	2	0
GINGIVAL DISORDERS, SIGNS AND SYMPTOMS NEC										
GINGIVAL BLISTER	0	0	0	0	0	0	0	0	0	0
GINGIVAL PAIN	0	0	0	0	0	0	0	0	0	0
GINGIVAL SWELLING	0	0	0	0	0	0	0	0	0	0
GASTROINTESTINAL CONDITIONS NEC										

GASTROINTESTINAL MUCOSAL DYSTROPHIES AND SECRETION DISORDERS										
BARRETT'S OESOPHAGUS	0	0	0	0	0	0	0	0	0	0
GASTROINTESTINAL HAEMORRHAGES NEC										
INTESTINAL HAEMORRHAGES	0	0	0	0	0	0	0	0	0	0
RECTAL HAEMORRHAGE	0	0	0	0	0	0	0	0	0	0
NON-SITE SPECIFIC GASTROINTESTINAL HAEMORRHAGES										
HAEMATEMESIS	0	0	0	0	0	0	0	0	0	0
GASTROINTESTINAL INFLAMMATORY CONDITIONS										
COLITIS (EXCL INFECTIVE)										
COLITIS	1	0	1	0	0	1	0	1	0	0
COLITIS MICROSCOPIC	0	0	0	0	0	0	0	0	0	0
COLITIS ULCERATIVE	1	0	1	0	0	0	0	0	0	0
GASTRITIS (EXCL INFECTIVE)										
GASTRITIS	0	0	0	0	0	0	0	0	0	0
REFLUX GASTRITIS	0	0	0	0	0	0	0	0	0	0
GASTROINTESTINAL INFLAMMATORY DISORDERS NEC										
GASTROINTESTINAL TRACT IRRITATION	1	0	0	1	0	1	0	0	1	0
GASTROINTESTINAL MOTILITY AND DEFAECATION CONDITIONS										
DIARRHOEA (EXCL INFECTIVE)										
DIARRHOEA	104	57	18	28	1	95	57	14	23	1
GASTROINTESTINAL ATONIC AND HYPOMOTILITY DISORDERS NEC										
CONSTIPATION	5	3	0	2	0	4	3	0	1	0
GASTROOESOPHAGEAL REFLUX DISEASE	0	0	0	0	0	0	0	0	0	0
GASTROINTESTINAL DYSKINETIC DISORDERS										
CHANGE OF BOWEL HABIT	2	1	1	0	0	2	1	1	0	0
GASTROINTESTINAL SPASTIC AND HYPERMOTILITY DISORDERS										
IRRITABLE BOWEL SYNDROME	1	0	0	1	0	1	0	0	1	0
GASTROINTESTINAL SIGNS AND SYMPTOMS										
DYSPEPTIC SIGNS AND SYMPTOMS										
DYSPEPSIA	5	2	0	3	0	5	2	0	3	0
ERUCTATION	2	2	0	0	0	2	2	0	0	0
FAECAL ABNORMALITIES NEC										
FAECALOMA	1	0	1	0	0	1	0	1	0	0
FAECES DISCOLOURED	1	1	0	0	0	1	1	0	0	0
FAECES SOFT	0	0	0	0	0	0	0	0	0	0
FLATULENCE, BLOATING AND DISTENSION										
ABDOMINAL DISTENSION	1	0	0	0	1	1	0	0	0	1

FLATULENCE	3	2	0	1	0	2	2	0	0	0
GASTROINTESTINAL AND ABDOMINAL PAINS (EXCL ORAL AND THROAT)										
ABDOMINAL PAIN	3	2	1	0	0	3	2	1	0	0
ABDOMINAL PAIN LOWER	0	0	0	0	0	0	0	0	0	0
ABDOMINAL PAIN UPPER	31	21	4	5	1	29	21	3	5	0
GASTROINTESTINAL PAIN	3	2	0	1	0	2	2	0	0	0
GASTROINTESTINAL SIGNS AND SYMPTOMS NEC										
ABDOMINAL DISCOMFORT	17	8	4	5	0	16	8	3	5	0
ABDOMINAL SYMPTOM	0	0	0	0	0	0	0	0	0	0
ACUTE ABDOMEN	0	0	0	0	0	0	0	0	0	0
ANAL INCONTINENCE	1	1	0	0	0	1	1	0	0	0
BREATH ODOUR	1	1	0	0	0	1	1	0	0	0
DYSPHAGIA	2	1	1	0	0	1	1	0	0	0
ODYNOPHAGIA	1	0	1	0	0	0	0	0	0	0
NAUSEA AND VOMITING SYMPTOMS										
NAUSEA	304	174	58	65	7	285	174	48	56	7
RETCHING	0	0	0	0	0	0	0	0	0	0
VOMITING	43	27	6	8	2	41	27	6	7	1
VOMITING PROJECTILE	0	0	0	0	0	0	0	0	0	0
MALABSORPTION CONDITIONS										
MALABSORPTION SYNDROMES										
COELIAC DISEASE	0	0	0	0	0	0	0	0	0	0
ORAL SOFT TISSUE CONDITIONS										
ORAL SOFT TISSUE DISORDERS NEC										
CHEILITIS	0	0	0	0	0	0	0	0	0	0
LIP BLISTER	0	0	0	0	0	0	0	0	0	0
ORAL LICHEN PLANUS	0	0	0	0	0	0	0	0	0	0
ORAL SOFT TISSUE INFECTIONS										
ANGULAR CHEILITIS	0	0	0	0	0	0	0	0	0	0
ORAL SOFT TISSUE SIGNS AND SYMPTOMS										
HYPOAESTHESIA ORAL	5	4	1	0	0	4	4	0	0	0
LIP PAIN	0	0	0	0	0	0	0	0	0	0
ORAL DISCOMFORT	1	0	1	0	0	0	0	0	0	0
ORAL MUCOSAL ROUGHENING	1	1	0	0	0	1	1	0	0	0
ORAL PAIN	2	0	1	1	0	2	0	1	1	0

PARAESTHESIA ORAL	8	7	1	0	0	8	7	1	0	0
LIP SWELLING	2	1	0	1	0	2	1	0	1	0
MOUTH SWELLING	0	0	0	0	0	0	0	0	0	0
STOMATITIS AND ULCERATION										
APHTHOUS ULCER	0	0	0	0	0	0	0	0	0	0
LIP ULCERATION	1	0	0	0	1	0	0	0	0	0
MOUTH ULCERATION	7	4	1	1	1	7	4	1	1	1
STOMATITIS	1	1	0	0	0	1	1	0	0	0
SALIVARY GLAND CONDITIONS										
ORAL DRYNESS AND SALIVA ALTERED										
DRY MOUTH	6	5	0	0	1	5	5	0	0	0
LIP DRY	0	0	0	0	0	0	0	0	0	0
SALIVARY HYPOSECRETION	0	0	0	0	0	0	0	0	0	0
TONGUE CONDITIONS										
TONGUE SIGNS AND SYMPTOMS										
GLOSSODYNIA	5	4	0	1	0	5	4	0	1	0
SWOLLEN TONGUE	0	0	0	0	0	0	0	0	0	0
TONGUE COATED	2	2	0	0	0	2	2	0	0	0
TONGUE DISCOMFORT	1	0	1	0	0	0	0	0	0	0
TONGUE OEDEMA	1	0	0	1	0	1	0	0	1	0
TONGUE SPASM	0	0	0	0	0	0	0	0	0	0
**GENERAL DISORDERS AND ADMINISTRATION SITE CONDITIONS**										
ADMINISTRATION SITE REACTIONS										
ADMINISTRATION SITE REACTIONS NEC										
ADMINISTRATION SITE BRUISE	0	0	0	0	0	0	0	0	0	0
ADMINISTRATION SITE PAIN	1	1	0	0	0	1	1	0	0	0
PUNCTURE SITE BRUISE	6	4	1	1	0	6	4	1	1	0
PUNCTURE SITE PAIN	1	1	0	0	0	1	1	0	0	0
APPLICATION SITE REACTIONS										
APPLICATION SITE BRUISE	3	1	1	1	0	2	1	0	1	0
APPLICATION SITE ERYTHEMA	0	0	0	0	0	0	0	0	0	0
APPLICATION SITE PAIN	1	0	0	1	0	0	0	0	0	0
IMPLANT AND CATHETER SITE REACTIONS										
IMPLANT SITE PAIN	1	0	1	0	0	0	0	0	0	0
IMPLANT SITE WARMTH	0	0	0	0	0	0	0	0	0	0

INFUSION SITE REACTIONS										
INFUSION SITE PAIN	1	0	0	1	0	1	0	0	1	0
INFUSION SITE SCAB	0	0	0	0	0	0	0	0	0	0
INFUSION SITE WARMTH	0	0	0	0	0	0	0	0	0	0
INJECTION SITE REACTIONS										
INJECTION SITE BRUISING	3	2	1	0	0	3	2	1	0	0
INJECTION SITE DISCOMFORT	2	1	1	0	0	1	1	0	0	0
INJECTION SITE ERYTHEMA	11	6	2	2	1	11	6	2	2	1
INJECTION SITE HYPERSENSITIVITY	1	0	1	0	0	1	0	1	0	0
INJECTION SITE INFLAMMATION	0	0	0	0	0	0	0	0	0	0
INJECTION SITE INJURY	1	1	0	0	0	1	1	0	0	0
INJECTION SITE IRRITATION	1	0	1	0	0	1	0	1	0	0
INJECTION SITE JOINT PAIN	0	0	0	0	0	0	0	0	0	0
INJECTION SITE MASS	12	2	5	4	1	11	2	5	3	1
INJECTION SITE NODULE	1	0	0	1	0	1	0	0	1	0
INJECTION SITE OEDEMA	0	0	0	0	0	0	0	0	0	0
INJECTION SITE PAIN	237	155	39	41	2	216	155	31	28	2
INJECTION SITE PAPULE	0	0	0	0	0	0	0	0	0	0
INJECTION SITE PARAESTHESIA	1	1	0	0	0	1	1	0	0	0
INJECTION SITE PRURITUS	4	3	0	1	0	4	3	0	1	0
INJECTION SITE RASH	2	1	0	1	0	2	1	0	1	0
INJECTION SITE REACTION	2	0	2	0	0	2	0	2	0	0
INJECTION SITE SCAB	0	0	0	0	0	0	0	0	0	0
INJECTION SITE SWELLING	6	3	0	3	0	6	3	0	3	0
INJECTION SITE URTICARIA	2	0	2	0	0	2	0	2	0	0
INJECTION SITE WARMTH	2	1	1	0	0	2	1	1	0	0
INSTILLATION SITE REACTIONS										
INSTILLATION SITE PRURITUS	1	0	1	0	0	1	0	1	0	0
INSTILLATION SITE WARMTH	1	1	0	0	0	1	1	0	0	0
VACCINATION SITE REACTIONS										
SHOULDER INJURY RELATED TO VACCINE ADMINISTRATION	0	0	0	0	0	0	0	0	0	0
VACCINATION SITE BRUISING	0	0	0	0	0	0	0	0	0	0
VACCINATION SITE DISCOMFORT	1	1	0	0	0	1	1	0	0	0
VACCINATION SITE ERYTHEMA	1	1	0	0	0	1	1	0	0	0

VACCINATION SITE JOINT ERYTHEMA	0	0	0	0	0	0	0	0	0	0
VACCINATION SITE JOINT PAIN	0	0	0	0	0	0	0	0	0	0
VACCINATION SITE MASS	2	1	0	1	0	1	1	0	0	0
VACCINATION SITE PAIN	27	17	2	8	0	27	17	2	8	0
VACCINATION SITE RASH	0	0	0	0	0	0	0	0	0	0
VACCINATION SITE SWELLING	2	1	1	0	0	2	1	1	0	0
VACCINATION SITE WARMTH	1	1	0	0	0	1	1	0	0	0
BODY TEMPERATURE CONDITIONS										
BODY TEMPERATURE ALTERED										
HYPERTHERMIA	0	0	0	0	0	0	0	0	0	0
HYPOTHERMIA	1	1	0	0	0	1	1	0	0	0
FEBRILE DISORDERS										
PYREXIA	338	156	88	84	10	307	156	74	69	8
FATAL OUTCOMES										
DEATH AND SUDDEN DEATH										
DEATH	1	1	0	0	0	1	1	0	0	0
GENERAL SYSTEM DISORDERS NEC										
ADVERSE EFFECT ABSENT										
NO ADVERSE EVENT	0	0	0	0	0	0	0	0	0	0
ASTHENIC CONDITIONS										
ASTHENIA	34	21	6	7	0	29	21	4	4	0
CHRONIC FATIGUE SYNDROME	5	1	2	2	0	4	1	2	1	0
DECREASED ACTIVITY	1	1	0	0	0	1	1	0	0	0
FATIGUE	1180	654	277	229	20	1089	654	230	186	19
MALAISE	107	49	28	27	3	93	49	21	20	3
SLUGGISHNESS	0	0	0	0	0	0	0	0	0	0
FEELINGS AND SENSATIONS NEC										
CHILLS	204	87	36	78	3	186	87	31	66	2
FEELING ABNORMAL	29	10	6	11	2	24	10	4	8	2
FEELING COLD	54	30	10	12	2	47	30	8	8	1
FEELING HOT	13	5	3	5	0	13	5	3	5	0
FEELING JITTERY	0	0	0	0	0	0	0	0	0	0
FEELING OF BODY TEMPERATURE CHANGE	12	8	2	2	0	12	8	2	2	0
FEELING OF RELAXATION	0	0	0	0	0	0	0	0	0	0
HANGOVER	2	1	0	1	0	2	1	0	1	0

HUNGER	1	1	0	0	0	1	1	0	0	0
SENSATION OF BLOOD FLOW	0	0	0	0	0	0	0	0	0	0
THIRST	6	3	1	2	0	5	3	0	2	0
GAIT DISTURBANCES										
GAIT DISTURBANCE	2	1	1	0	0	2	1	1	0	0
GAIT INABILITY	1	1	0	0	0	1	1	0	0	0
GENERAL SIGNS AND SYMPTOMS NEC										
CRYING	0	0	0	0	0	0	0	0	0	0
ENERGY INCREASED	0	0	0	0	0	0	0	0	0	0
EXERCISE TOLERANCE DECREASED	0	0	0	0	0	0	0	0	0	0
GENERAL SYMPTOM	1	1	0	0	0	1	1	0	0	0
ILLNESS	34	12	8	14	0	30	12	7	11	0
INFLUENZA LIKE ILLNESS	87	25	21	39	2	76	25	16	33	2
LOCAL REACTION	2	1	1	0	0	2	1	1	0	0
PERIPHERAL SWELLING	90	49	19	21	1	82	49	17	15	1
SWELLING	34	20	5	9	0	31	20	4	7	0
SWELLING FACE	4	4	0	0	0	4	4	0	0	0
TISSUE IRRITATION	0	0	0	0	0	0	0	0	0	0
INFLAMMATIONS										
INFLAMMATION	5	3	2	0	0	4	3	1	0	0
SYSTEMIC INFLAMMATORY RESPONSE SYNDROME	0	0	0	0	0	0	0	0	0	0
OEDEMA NEC										
OEDEMA	0	0	0	0	0	0	0	0	0	0
OEDEMA PERIPHERAL	1	0	1	0	0	1	0	1	0	0
PAIN AND DISCOMFORT NEC										
AXILLARY PAIN	50	12	16	21	1	47	12	14	20	1
CHEST DISCOMFORT	9	6	0	3	0	9	6	0	3	0
CHEST PAIN	34	23	6	5	0	32	23	5	4	0
DISCOMFORT	10	3	3	3	1	8	3	2	3	0
FACIAL PAIN	1	0	0	1	0	0	0	0	0	0
HERNIA PAIN	1	1	0	0	0	1	1	0	0	0
INFLAMMATORY PAIN	2	1	0	0	1	1	1	0	0	0
NON-CARDIAC CHEST PAIN	1	0	0	1	0	1	0	0	1	0
PAIN	363	217	74	66	6	333	217	57	54	5
TENDERNESS	121	67	26	27	1	115	67	22	25	1

THERAPEUTIC AND NONTHERAPEUTIC EFFECTS (EXCL TOXICITY)										
THERAPEUTIC AND NONTHERAPEUTIC RESPONSES										
ADVERSE DRUG REACTION	0	0	0	0	0	0	0	0	0	0
ADVERSE EVENT	0	0	0	0	0	0	0	0	0	0
ADVERSE REACTION	1	1	0	0	0	1	1	0	0	0
IMMEDIATE POST-INJECTION REACTION	1	0	1	0	0	1	0	1	0	0
**HEPATOBILIARY DISORDERS**										
HEPATIC AND HEPATOBILIARY DISORDERS										
HEPATOBILIARY SIGNS AND SYMPTOMS										
HEPATIC PAIN	0	0	0	0	0	0	0	0	0	0
HEPATOCELLULAR DAMAGE AND HEPATITIS NEC										
LIVER INJURY	0	0	0	0	0	0	0	0	0	0
**IMMUNE SYSTEM DISORDERS**										
ALLERGIC CONDITIONS										
ALLERGIC CONDITIONS NEC										
HYPERSENSITIVITY	1	0	0	1	0	1	0	0	1	0
MULTIPLE ALLERGIES	0	0	0	0	0	0	0	0	0	0
ALLERGIES TO FOODS, FOOD ADDITIVES, DRUGS AND OTHER CHEMICALS										
ALLERGY TO CHEMICALS	0	0	0	0	0	0	0	0	0	0
ANAPHYLACTIC AND ANAPHYLACTOID RESPONSES										
ANAPHYLACTIC REACTION	0	0	0	0	0	0	0	0	0	0
ATOPIC DISORDERS										
SEASONAL ALLERGY	0	0	0	0	0	0	0	0	0	0
IMMUNE DISORDERS NEC										
IMMUNE AND ASSOCIATED CONDITIONS NEC										
BACILLE CALMETTE-GUERIN SCAR REACTIVATION	0	0	0	0	0	0	0	0	0	0
SENSITISATION	0	0	0	0	0	0	0	0	0	0
**INFECTIONS AND INFESTATIONS**										
BACTERIAL INFECTIOUS DISORDERS										
BACTERIAL INFECTIONS NEC										
CELLULITIS	0	0	0	0	0	0	0	0	0	0
PERIORBITAL CELLULITIS	1	0	1	0	0	1	0	1	0	0
HELICOBACTER INFECTIONS										
HELICOBACTER GASTRITIS	1	0	0	1	0	1	0	0	1	0
STAPHYLOCOCCAL INFECTIONS										

FURUNCLE	1	1	0	0	0	1	1	0	0	0
FUNGAL INFECTIOUS DISORDERS										
CANDIDA INFECTIONS										
CANDIDA INFECTION	1	1	0	0	0	1	1	0	0	0
VULVOVAGINAL CANDIDIASIS	0	0	0	0	0	0	0	0	0	0
INFECTIONS - PATHOGEN UNSPECIFIED										
ABDOMINAL AND GASTROINTESTINAL INFECTIONS										
APPENDICITIS	0	0	0	0	0	0	0	0	0	0
DIARRHOEA INFECTIOUS	0	0	0	0	0	0	0	0	0	0
GASTROINTESTINAL INFECTION	0	0	0	0	0	0	0	0	0	0
BREAST INFECTIONS										
MASTITIS	1	0	0	1	0	1	0	0	1	0
CENTRAL NERVOUS SYSTEM AND SPINAL INFECTIONS										
MYELITIS	0	0	0	0	0	0	0	0	0	0
DENTAL AND ORAL SOFT TISSUE INFECTIONS										
ABSCESS ORAL	1	1	0	0	0	1	1	0	0	0
ORAL PUSTULE	0	0	0	0	0	0	0	0	0	0
PERICORONITIS	0	0	0	0	0	0	0	0	0	0
TOOTH ABSCESS	0	0	0	0	0	0	0	0	0	0
EAR INFECTIONS										
EAR INFECTION	0	0	0	0	0	0	0	0	0	0
LABYRINTHITIS	3	1	2	0	0	3	1	2	0	0
OTITIS EXTERNA	0	0	0	0	0	0	0	0	0	0
EYE AND EYELID INFECTIONS										
CONJUNCTIVITIS	0	0	0	0	0	0	0	0	0	0
HORDEOLUM	0	0	0	0	0	0	0	0	0	0
INFECTIONS NEC										
ABSCESS	0	0	0	0	0	0	0	0	0	0
INFECTION	0	0	0	0	0	0	0	0	0	0
INJECTION SITE INFECTION	0	0	0	0	0	0	0	0	0	0
LOCALISED INFECTION	1	1	0	0	0	1	1	0	0	0
WOUND INFECTION	0	0	0	0	0	0	0	0	0	0
LOWER RESPIRATORY TRACT AND LUNG INFECTIONS										
LOWER RESPIRATORY TRACT INFECTION	3	1	1	1	0	3	1	1	1	0
PNEUMONIA	0	0	0	0	0	0	0	0	0	0

MALE REPRODUCTIVE TRACT INFECTIONS										
ORCHITIS	0	0	0	0	0	0	0	0	0	0
SEPSIS, BACTERAEMIA, VIRAEMIA AND FUNGAEMIA NEC										
SEPTIC RASH	0	0	0	0	0	0	0	0	0	0
SKIN STRUCTURES AND SOFT TISSUE INFECTIONS										
INFECTED DERMAL CYST	1	1	0	0	0	1	1	0	0	0
INJECTION SITE PUSTULE	0	0	0	0	0	0	0	0	0	0
SKIN INFECTION	1	1	0	0	0	1	1	0	0	0
UPPER RESPIRATORY TRACT INFECTIONS										
LARYNGITIS	1	0	1	0	0	1	0	1	0	0
NASOPHARYNGITIS	58	30	9	19	0	52	30	6	16	0
PHARYNGITIS	0	0	0	0	0	0	0	0	0	0
RHINITIS	0	0	0	0	0	0	0	0	0	0
SINUSITIS	3	1	1	1	0	2	1	0	1	0
TONSILLITIS	0	0	0	0	0	0	0	0	0	0
URINARY TRACT INFECTIONS										
CYSTITIS	2	1	1	0	0	2	1	1	0	0
URINARY TRACT INFECTION	0	0	0	0	0	0	0	0	0	0
PROTOZOAL INFECTIOUS DISORDERS										
TRYPANOSOMAL INFECTIONS										
AFRICAN TRYPANOSOMIASIS	1	1	0	0	0	1	1	0	0	0
RICKETTSIAL INFECTIOUS DISORDERS										
COXIELLA INFECTIONS										
Q FEVER	1	1	0	0	0	1	1	0	0	0
VIRAL INFECTIOUS DISORDERS										
CORONAVIRUS INFECTIONS										
COVID-19	2	1	0	1	0	2	1	0	1	0
HERPES VIRAL INFECTIONS										
GENITAL HERPES	1	1	0	0	0	1	1	0	0	0
HERPES OPHTHALMIC	0	0	0	0	0	0	0	0	0	0
HERPES SIMPLEX	1	1	0	0	0	1	1	0	0	0
HERPES ZOSTER	7	4	2	1	0	6	4	2	0	0
NASAL HERPES	0	0	0	0	0	0	0	0	0	0
OPHTHALMIC HERPES ZOSTER	0	0	0	0	0	0	0	0	0	0
ORAL HERPES	13	10	2	1	0	13	10	2	1	0
INFLUENZA VIRAL INFECTIONS										

H2N2 INFLUENZA	0	0	0	0	0	0	0	0	0	0
INFLUENZA	129	48	27	51	3	110	48	22	37	3
RETROVIRAL INFECTIONS										
AIDS RELATED COMPLEX	0	0	0	0	0	0	0	0	0	0
VIRAL INFECTIONS NEC										
GASTROENTERITIS VIRAL	0	0	0	0	0	0	0	0	0	0
SWEATING FEVER	1	1	0	0	0	1	1	0	0	0
VESTIBULAR NEURONITIS	0	0	0	0	0	0	0	0	0	0
VIRAL DIARRHOEA	1	1	0	0	0	1	1	0	0	0
VIRAL RASH	1	0	0	0	1	1	0	0	0	1
VIRAL UPPER RESPIRATORY TRACT INFECTION	0	0	0	0	0	0	0	0	0	0
**INJURY, POISONING AND PROCEDURAL COMPLICATIONS**										
BONE AND JOINT INJURIES										
FRACTURES AND DISLOCATIONS NEC										
JOINT DISLOCATION	0	0	0	0	0	0	0	0	0	0
EXPOSURES, CHEMICAL INJURIES AND POISONING										
POISONING AND TOXICITY										
SYSTEMIC TOXICITY	0	0	0	0	0	0	0	0	0	0
TOXICITY TO VARIOUS AGENTS	1	1	0	0	0	1	1	0	0	0
INJURIES BY PHYSICAL AGENTS										
CONDITIONS CAUSED BY COLD										
CHILLBLAINS	1	1	0	0	0	1	1	0	0	0
HEAT INJURIES (EXCL THERMAL BURNS)										
HEAT EXHAUSTION	0	0	0	0	0	0	0	0	0	0
HEAT OEDEMA	0	0	0	0	0	0	0	0	0	0
RADIATION INJURIES										
SUNBURN	0	0	0	0	0	0	0	0	0	0
THERMAL BURNS										
THERMAL BURN	0	0	0	0	0	0	0	0	0	0
THERMAL BURNS OF EYE	0	0	0	0	0	0	0	0	0	0
INJURIES NEC										
CHEST AND RESPIRATORY TRACT INJURIES NEC										
CHEST CRUSHING	0	0	0	0	0	0	0	0	0	0
EYE INJURIES NEC										
EYE CONTUSION	1	1	0	0	0	1	1	0	0	0
MUSCLE, TENDON AND LIGAMENT INJURIES										

LIGAMENT SPRAIN	0	0	0	0	0	0	0	0	0	0
MUSCLE INJURY	1	0	0	1	0	0	0	0	0	0
MUSCLE STRAIN	0	0	0	0	0	0	0	0	0	0
NERVE INJURIES NEC										
NERVE INJURY	0	0	0	0	0	0	0	0	0	0
NON-SITE SPECIFIC INJURIES NEC										
ARTHROPOD STING	2	2	0	0	0	2	2	0	0	0
BITE	0	0	0	0	0	0	0	0	0	0
FALL	1	1	0	0	0	1	1	0	0	0
INFLAMMATION OF WOUND	1	1	0	0	0	1	1	0	0	0
TISSUE INJURY	0	0	0	0	0	0	0	0	0	0
WOUND COMPLICATION	0	0	0	0	0	0	0	0	0	0
WOUND SECRETION	0	0	0	0	0	0	0	0	0	0
SITE SPECIFIC INJURIES NEC										
LIMB INJURY	0	0	0	0	0	0	0	0	0	0
SKIN INJURIES NEC										
CONTUSION	56	29	10	14	3	52	29	8	12	3
SCAR	1	1	0	0	0	1	1	0	0	0
SKIN WOUND	0	0	0	0	0	0	0	0	0	0
PROCEDURAL RELATED INJURIES AND COMPLICATIONS NEC										
CARDIAC AND VASCULAR PROCEDURAL COMPLICATIONS										
CARDIAC PROCEDURE COMPLICATION	0	0	0	0	0	0	0	0	0	0
GASTROINTESTINAL AND HEPATOBILIARY PROCEDURAL COMPLICATIONS										
PROCEDURAL NAUSEA	0	0	0	0	0	0	0	0	0	0
NEUROLOGICAL AND PSYCHIATRIC PROCEDURAL COMPLICATIONS										
PROCEDURAL DIZZINESS	0	0	0	0	0	0	0	0	0	0
NON-SITE SPECIFIC PROCEDURAL COMPLICATIONS										
INCISION SITE PAIN	1	1	0	0	0	1	1	0	0	0
INCISION SITE SWELLING	1	0	1	0	0	1	0	1	0	0
INJECTION RELATED REACTION	1	1	0	0	0	1	1	0	0	0
POST PROCEDURAL COMPLICATION	0	0	0	0	0	0	0	0	0	0
PROCEDURAL PAIN	0	0	0	0	0	0	0	0	0	0
VACCINATION RELATED COMPLICATIONS										
IMMUNISATION REACTION	3	1	1	1	0	3	1	1	1	0
**INVESTIGATIONS**										

CARDIAC AND VASCULAR INVESTIGATIONS (EXCL ENZYME TESTS)										
HEART RATE AND PULSE INVESTIGATIONS										
HEART RATE	7	5	1	1	0	7	5	1	1	0
HEART RATE DECREASED	1	1	0	0	0	1	1	0	0	0
HEART RATE INCREASED	7	6	0	1	0	7	6	0	1	0
HEART RATE IRREGULAR	2	0	1	1	0	2	0	1	1	0
VASCULAR TESTS NEC (INCL BLOOD PRESSURE)										
BLOOD PRESSURE DECREASED	0	0	0	0	0	0	0	0	0	0
BLOOD PRESSURE INCREASED	3	2	0	1	0	3	2	0	1	0
BLOOD PRESSURE MEASUREMENT	0	0	0	0	0	0	0	0	0	0
ENDOCRINE INVESTIGATIONS (INCL SEX HORMONES)										
ENDOCRINE ANALYSES AND IMAGING NEC										
HORMONE LEVEL ABNORMAL	1	1	0	0	0	1	1	0	0	0
PITUITARY ANALYSES ANTERIOR										
BLOOD FOLLICLE STIMULATING HORMONE INCREASED	0	0	0	0	0	0	0	0	0	0
BLOOD LUTEINISING HORMONE	1	1	0	0	0	1	1	0	0	0
THYROID ANALYSES										
TRI-IODOTHYRONINE	0	0	0	0	0	0	0	0	0	0
HAEMATOLOGY INVESTIGATIONS (INCL BLOOD GROUPS)										
COAGULATION AND BLEEDING ANALYSES										
BLEEDING TIME	0	0	0	0	0	0	0	0	0	0
INTERNATIONAL NORMALISED RATIO DECREASED	0	0	0	0	0	0	0	0	0	0
PLATELET ANALYSES										
PLATELET COUNT INCREASED	0	0	0	0	0	0	0	0	0	0
RED BLOOD CELL ANALYSES										
HAEMOGLOBIN	0	0	0	0	0	0	0	0	0	0
METABOLIC, NUTRITIONAL AND BLOOD GAS INVESTIGATIONS										
BLOOD GAS AND ACID BASE ANALYSES										
OXYGEN SATURATION DECREASED	0	0	0	0	0	0	0	0	0	0
CARBOHYDRATE TOLERANCE ANALYSES (INCL DIABETES)										
BLOOD GLUCOSE	1	1	0	0	0	1	1	0	0	0
BLOOD GLUCOSE ABNORMAL	0	0	0	0	0	0	0	0	0	0
BLOOD GLUCOSE DECREASED	0	0	0	0	0	0	0	0	0	0
BLOOD GLUCOSE INCREASED	2	2	0	0	0	2	2	0	0	0
MICROBIOLOGY AND SEROLOGY INVESTIGATIONS										

VIRUS IDENTIFICATION AND SEROLOGY										
SARS-COV-2 TEST	1	1	0	0	0	1	1	0	0	0
SARS-COV-2 TEST POSITIVE	1	0	0	1	0	1	0	0	1	0
MUSCULOSKELETAL AND SOFT TISSUE INVESTIGATIONS (EXCL ENZYME TESTS)										
MUSCULOSKELETAL AND SOFT TISSUE IMAGING PROCEDURES										
BONE SCAN	0	0	0	0	0	0	0	0	0	0
NEUROLOGICAL, SPECIAL SENSES AND PSYCHIATRIC INVESTIGATIONS										
CENTRAL NERVOUS SYSTEM IMAGING PROCEDURES										
MAGNETIC RESONANCE IMAGING HEAD	2	2	0	0	0	2	2	0	0	0
SCAN BRAIN	1	0	0	1	0	1	0	0	1	0
PHYSICAL EXAMINATION AND ORGAN SYSTEM STATUS TOPICS										
PHYSICAL EXAMINATION PROCEDURES AND ORGAN SYSTEM STATUS										
BODY TEMPERATURE	4	0	3	1	0	4	0	3	1	0
BODY TEMPERATURE ABNORMAL	1	1	0	0	0	1	1	0	0	0
BODY TEMPERATURE DECREASED	0	0	0	0	0	0	0	0	0	0
BODY TEMPERATURE FLUCTUATION	0	0	0	0	0	0	0	0	0	0
BODY TEMPERATURE INCREASED	10	4	2	4	0	8	4	1	3	0
GRIP STRENGTH DECREASED	0	0	0	0	0	0	0	0	0	0
HEAD LAG	0	0	0	0	0	0	0	0	0	0
LEFT-HANDEDNESS	1	1	0	0	0	1	1	0	0	0
LYMPH NODE PALPABLE	0	0	0	0	0	0	0	0	0	0
RESPIRATORY RATE DECREASED	0	0	0	0	0	0	0	0	0	0
SKIN TEMPERATURE	1	0	0	1	0	1	0	0	1	0
WEIGHT DECREASED	2	2	0	0	0	2	2	0	0	0
WEIGHT INCREASED	0	0	0	0	0	0	0	0	0	0
RENAL AND URINARY TRACT INVESTIGATIONS AND URINALYSES										
URINALYSIS NEC										
BLOOD URINE	0	0	0	0	0	0	0	0	0	0
NITRITE URINE PRESENT	0	0	0	0	0	0	0	0	0	0
PH URINE	1	1	0	0	0	1	1	0	0	0
URINARY TRACT FUNCTION ANALYSES NEC										
URINE OUTPUT	1	0	0	1	0	1	0	0	1	0
URINE OUTPUT INCREASED	0	0	0	0	0	0	0	0	0	0
REPRODUCTIVE ORGAN AND BREAST INVESTIGATIONS (EXCL HORMONE ANALYSES)										
REPRODUCTIVE ORGAN AND BREAST IMAGING PROCEDURES										

BREAST SCAN	1	0	1	0	0	1	0	1	0	0
RESPIRATORY AND PULMONARY INVESTIGATIONS (EXCL BLOOD GASES)										
RESPIRATORY AND PULMONARY FUNCTION DIAGNOSTIC PROCEDURES										
FORCED EXPIRATORY VOLUME DECREASED	0	0	0	0	0	0	0	0	0	0
FORCED EXPIRATORY VOLUME INCREASED	1	0	1	0	0	1	0	1	0	0
WATER, ELECTROLYTE AND MINERAL INVESTIGATIONS										
WATER AND ELECTROLYTE ANALYSES NEC										
VOLUME BLOOD	1	0	1	0	0	1	0	1	0	0
**METABOLISM AND NUTRITION DISORDERS**										
APPETITE AND GENERAL NUTRITIONAL DISORDERS										
APPETITE DISORDERS										
APPETITE DISORDER	0	0	0	0	0	0	0	0	0	0
DECREASED APPETITE	23	14	2	5	2	22	14	2	5	1
FOOD CRAVING	1	1	0	0	0	1	1	0	0	0
FOOD REFUSAL	0	0	0	0	0	0	0	0	0	0
INCREASED APPETITE	0	0	0	0	0	0	0	0	0	0
GENERAL NUTRITIONAL DISORDERS NEC										
FOOD AVERSION	0	0	0	0	0	0	0	0	0	0
ELECTROLYTE AND FLUID BALANCE CONDITIONS										
TOTAL FLUID VOLUME DECREASED										
DEHYDRATION	0	0	0	0	0	0	0	0	0	0
TOTAL FLUID VOLUME INCREASED										
FLUID RETENTION	0	0	0	0	0	0	0	0	0	0
FOOD INTOLERANCE SYNDROMES										
FOOD MALABSORPTION AND INTOLERANCE SYNDROMES (EXCL SUGAR INTOLERANCE)										
ALCOHOL INTOLERANCE	0	0	0	0	0	0	0	0	0	0
GLUCOSE METABOLISM DISORDERS (INCL DIABETES MELLITUS)										
DIABETES MELLITUS (INCL SUBTYPES)										
DIABETES MELLITUS	1	1	0	0	0	1	1	0	0	0
DIABETES MELLITUS INADEQUATE CONTROL	1	1	0	0	0	1	1	0	0	0
HYPERGLYCAEMIC CONDITIONS NEC										
HYPERGLYCAEMIA	1	1	0	0	0	1	1	0	0	0
HYPOGLYCAEMIC CONDITIONS NEC										
HYPOGLYCAEMIA	1	1	0	0	0	1	1	0	0	0
PURINE AND PYRIMIDINE METABOLISM DISORDERS										
DISORDERS OF PURINE METABOLISM										

GOUT	0	0	0	0	0	0	0	0	0	0
**MUSCULOSKELETAL AND CONNECTIVE TISSUE DISORDERS**										
BONE DISORDERS (EXCL CONGENITAL AND FRACTURES)										
BONE DISORDERS NEC										
JAW DISORDER	0	0	0	0	0	0	0	0	0	0
OSTEITIS	0	0	0	0	0	0	0	0	0	0
BONE RELATED SIGNS AND SYMPTOMS										
BONE PAIN	2	1	0	1	0	2	1	0	1	0
BONE SWELLING	0	0	0	0	0	0	0	0	0	0
PAIN IN JAW	4	3	0	1	0	4	3	0	1	0
PUBIC PAIN	0	0	0	0	0	0	0	0	0	0
SPINAL PAIN	1	0	1	0	0	1	0	1	0	0
CONNECTIVE TISSUE DISORDERS (EXCL CONGENITAL)										
CONNECTIVE TISSUE DISORDERS NEC										
POLYMYALGIA RHEUMATICA	1	0	0	1	0	1	0	0	1	0
LUPUS ERYTHEMATOSUS (INCL SUBTYPES)										
SYSTEMIC LUPUS ERYTHEMATOSUS	0	0	0	0	0	0	0	0	0	0
JOINT DISORDERS										
ARTHROPATHIES NEC										
ARTHRITIS	5	1	2	2	0	5	1	2	2	0
ARTHROPATHY	1	0	1	0	0	1	0	1	0	0
POLYARTHRITIS	1	0	1	0	0	1	0	1	0	0
RHEUMATIC FEVER	0	0	0	0	0	0	0	0	0	0
JOINT RELATED DISORDERS NEC										
JOINT LOCK	0	0	0	0	0	0	0	0	0	0
PERIARTHRITIS	2	1	0	1	0	2	1	0	1	0
TEMPOROMANDIBULAR PAIN AND DYSFUNCTION SYNDROME	1	1	0	0	0	1	1	0	0	0
JOINT RELATED SIGNS AND SYMPTOMS										
ARTHRALGIA	332	174	64	87	7	298	174	47	72	5
JAW CLICKING	0	0	0	0	0	0	0	0	0	0
JOINT STIFFNESS	9	7	0	2	0	8	7	0	1	0
JOINT SWELLING	7	3	1	3	0	5	3	1	1	0
OSTEOARTHROPATHIES										
OSTEOARTHRITIS	1	1	0	0	0	1	1	0	0	0
PSORIATIC ARTHROPATHIES										

PSORIATIC ARTHROPATHY	0	0	0	0	0	0	0	0	0	0
RHEUMATOID ARTHROPATHIES										
RHEUMATOID ARTHRITIS	1	1	0	0	0	1	1	0	0	0
MUSCLE DISORDERS										
MUSCLE INFECTIONS AND INFLAMMATIONS										
MYOSITIS	1	0	1	0	0	1	0	1	0	0
MUSCLE PAINS										
FIBROMYALGIA	2	0	0	2	0	2	0	0	2	0
MYALGIA	457	271	82	96	8	417	271	67	72	7
MUSCLE RELATED SIGNS AND SYMPTOMS NEC										
MUSCLE ATROPHY	0	0	0	0	0	0	0	0	0	0
MUSCLE DISCOMFORT	1	1	0	0	0	1	1	0	0	0
MUSCLE FATIGUE	9	5	2	2	0	9	5	2	2	0
MUSCLE FIBROSIS	0	0	0	0	0	0	0	0	0	0
MUSCLE MASS	0	0	0	0	0	0	0	0	0	0
MUSCLE SPASMS	12	6	4	2	0	11	6	3	2	0
MUSCLE TIGHTNESS	0	0	0	0	0	0	0	0	0	0
MUSCLE TWITCHING	0	0	0	0	0	0	0	0	0	0
MUSCLE TONE ABNORMALITIES										
MUSCLE RIGIDITY	1	1	0	0	0	1	1	0	0	0
TRISMUS	0	0	0	0	0	0	0	0	0	0
MUSCLE WEAKNESS CONDITIONS										
MUSCULAR WEAKNESS	13	6	5	2	0	11	6	4	1	0
MUSCULOSKELETAL AND CONNECTIVE TISSUE DISORDERS NEC										
MUSCULOSKELETAL AND CONNECTIVE TISSUE CONDITIONS NEC										
MOBILITY DECREASED	1	0	1	0	0	0	0	0	0	0
MUSCULOSKELETAL STIFFNESS	78	44	9	25	0	71	44	9	18	0
BACK PAIN	34	16	8	10	0	30	16	7	7	0
FLANK PAIN	0	0	0	0	0	0	0	0	0	0
LIMB DISCOMFORT	242	150	47	45	0	228	150	40	38	0
MUSCULOSKELETAL CHEST PAIN	0	0	0	0	0	0	0	0	0	0
MUSCULOSKELETAL DISCOMFORT	2	0	1	1	0	1	0	0	1	0
MUSCULOSKELETAL PAIN	1	0	0	1	0	1	0	0	1	0
NECK PAIN	22	9	5	8	0	21	9	5	7	0
PAIN IN EXTREMITY	1622	1059	262	286	15	1515	1059	207	235	14

SOFT TISSUE DISORDERS NEC										
AXILLARY MASS	1	0	0	1	0	0	0	0	0	0
GROIN PAIN	1	0	1	0	0	1	0	1	0	0
SYNOVIAL AND BURSAL DISORDERS										
SYNOVIAL DISORDERS										
SYNOVITIS	1	0	1	0	0	1	0	1	0	0
TENDON, LIGAMENT AND CARTILAGE DISORDERS										
CARTILAGE DISORDERS										
COSTOCHONDRITIS	0	0	0	0	0	0	0	0	0	0
TENDON DISORDERS										
TENDONITIS	0	0	0	0	0	0	0	0	0	0
TENOSYNOVITIS	1	0	1	0	0	1	0	1	0	0
TRIGGER FINGER	0	0	0	0	0	0	0	0	0	0
**NEOPLASMS BENIGN, MALIGNANT AND UNSPECIFIED (INCL CYSTS AND POLYPS)**										
CUTANEOUS NEOPLASMS BENIGN										
SKIN NEOPLASMS BENIGN										
MELANOCYTIC NAEVUS	0	0	0	0	0	0	0	0	0	0
SEBORRHOEIC KERATOSIS	0	0	0	0	0	0	0	0	0	0
SKIN PAPILLOMA	1	1	0	0	0	1	1	0	0	0
**NERVOUS SYSTEM DISORDERS**										
CENTRAL NERVOUS SYSTEM INFECTIONS AND INFLAMMATIONS										
MYELITIS (INCL INFECTIVE)										
MYELITIS TRANSVERSE	0	0	0	0	0	0	0	0	0	0
CENTRAL NERVOUS SYSTEM VASCULAR DISORDERS										
CENTRAL NERVOUS SYSTEM HAEMORRHAGES AND CEREBROVASCULAR ACCIDENTS										
CEREBRAL HAEMORRHAGE	0	0	0	0	0	0	0	0	0	0
CEREBROVASCULAR ACCIDENT	1	0	0	1	0	1	0	0	1	0
TRANSIENT CEREBROVASCULAR EVENTS										
TRANSIENT ISCHAEMIC ATTACK	0	0	0	0	0	0	0	0	0	0
CRANIAL NERVE DISORDERS (EXCL NEOPLASMS)										
FACIAL CRANIAL NERVE DISORDERS										
BELL'S PALSY	1	0	1	0	0	1	0	1	0	0
FACIAL PARALYSIS	3	3	0	0	0	3	3	0	0	0
FACIAL PARESIS	0	0	0	0	0	0	0	0	0	0
OLFACTORY NERVE DISORDERS										
ANOSMIA	5	4	1	0	0	5	4	1	0	0

HYPOSMIA	0	0	0	0	0	0	0	0	0	0
PAROSMIA	6	4	1	1	0	6	4	1	1	0
TRIGEMINAL DISORDERS										
TRIGEMINAL NEURALGIA	1	1	0	0	0	1	1	0	0	0
TRIGEMINAL NEURITIS	1	0	0	1	0	1	0	0	1	0
DEMYELINATING DISORDERS										
MULTIPLE SCLEROSIS ACUTE AND PROGRESSIVE										
MULTIPLE SCLEROSIS RELAPSE	0	0	0	0	0	0	0	0	0	0
HEADACHES										
HEADACHES NEC										
CLUSTER HEADACHE	5	0	3	2	0	3	0	1	2	0
COLD-STIMULUS HEADACHE	0	0	0	0	0	0	0	0	0	0
DRUG WITHDRAWAL HEADACHE	0	0	0	0	0	0	0	0	0	0
HEADACHE	1119	618	207	274	20	1024	618	166	223	17
PRIMARY STABBING HEADACHE	1	1	0	0	0	1	1	0	0	0
SINUS HEADACHE	18	11	4	3	0	16	11	3	2	0
TENSION HEADACHE	27	16	4	7	0	24	16	3	5	0
THUNDERCLAP HEADACHE	0	0	0	0	0	0	0	0	0	0
VASCULAR HEADACHE	1	1	0	0	0	1	1	0	0	0
MIGRAINE HEADACHES										
MIGRAINE	45	26	9	10	0	41	26	8	7	0
MIGRAINE WITH AURA	4	4	0	0	0	4	4	0	0	0
RETINAL MIGRAINE	1	1	0	0	0	1	1	0	0	0
TYPICAL AURA WITHOUT HEADACHE	1	0	1	0	0	1	0	1	0	0
MENTAL IMPAIRMENT DISORDERS										
INTELLECTUAL DISABILITIES										
INTELLECTUAL DISABILITY	1	0	0	1	0	1	0	0	1	0
MEMORY LOSS (EXCL DEMENTIA)										
AMNESIA	0	0	0	0	0	0	0	0	0	0
MEMORY IMPAIRMENT	3	2	1	0	0	2	2	0	0	0
MENTAL IMPAIRMENT (EXCL DEMENTIA AND MEMORY LOSS)										
COGNITIVE DISORDER	2	1	0	1	0	2	1	0	1	0
DISTURBANCE IN ATTENTION	3	1	2	0	0	3	1	2	0	0
MENTAL IMPAIRMENT	3	2	0	1	0	2	2	0	0	0
MOVEMENT DISORDERS (INCL PARKINSONISM)										

DYSKINESIAS AND MOVEMENT DISORDERS NEC										
BRADYKINESIA	0	0	0	0	0	0	0	0	0	0
CLUMSINESS	0	0	0	0	0	0	0	0	0	0
DYSKINESIA	0	0	0	0	0	0	0	0	0	0
EXTRAPYRAMIDAL DISORDER	0	0	0	0	0	0	0	0	0	0
PSYCHOMOTOR HYPERACTIVITY	0	0	0	0	0	0	0	0	0	0
PARALYSIS AND PARESIS (EXCL CRANIAL NERVE)										
HEMIPLEGIA	0	0	0	0	0	0	0	0	0	0
MONOPARESIS	2	2	0	0	0	2	2	0	0	0
MONOPLEGIA	0	0	0	0	0	0	0	0	0	0
PARALYSIS	1	1	0	0	0	1	1	0	0	0
PARKINSON'S DISEASE AND PARKINSONISM										
FREEZING PHENOMENON	0	0	0	0	0	0	0	0	0	0
PARKINSON'S DISEASE	1	1	0	0	0	1	1	0	0	0
TREMOR (EXCL CONGENITAL)										
TREMOR	19	10	2	7	0	16	10	1	5	0
NEUROLOGICAL DISORDERS NEC										
COMA STATES										
DIABETIC HYPERGLYCAEMIC COMA	0	0	0	0	0	0	0	0	0	0
COORDINATION AND BALANCE DISTURBANCES										
BALANCE DISORDER	6	3	1	2	0	6	3	1	2	0
COORDINATION ABNORMAL	1	0	1	0	0	1	0	1	0	0
DYSSTASIA	1	1	0	0	0	1	1	0	0	0
VESTIBULAR NYSTAGMUS	0	0	0	0	0	0	0	0	0	0
DISTURBANCES IN CONSCIOUSNESS NEC										
DEPRESSED LEVEL OF CONSCIOUSNESS	0	0	0	0	0	0	0	0	0	0
LETHARGY	75	35	20	20	0	65	35	15	15	0
LOSS OF CONSCIOUSNESS	2	1	1	0	0	2	1	1	0	0
SEDATION	0	0	0	0	0	0	0	0	0	0
SOMNOLENCE	32	19	9	4	0	30	19	7	4	0
SYNCOPE	12	8	4	0	0	10	8	2	0	0
NERVOUS SYSTEM DISORDERS NEC										
NERVOUS SYSTEM DISORDER	0	0	0	0	0	0	0	0	0	0
NEUROLOGICAL SIGNS AND SYMPTOMS NEC										
AGITATION NEONATAL	0	0	0	0	0	0	0	0	0	0

BRAIN FOG	7	5	1	1	0	7	5	1	1	0
DIZZINESS	172	112	38	21	1	163	112	33	18	0
DIZZINESS EXERTIONAL	1	0	1	0	0	0	0	0	0	0
DIZZINESS POSTURAL	21	13	5	3	0	19	13	3	3	0
HEAD DISCOMFORT	4	3	0	1	0	4	3	0	1	0
INFANT IRRITABILITY	0	0	0	0	0	0	0	0	0	0
MENINGISM	0	0	0	0	0	0	0	0	0	0
MYOCLONUS	1	1	0	0	0	1	1	0	0	0
NEUROLOGICAL SYMPTOM	0	0	0	0	0	0	0	0	0	0
PERSISTENT POSTURAL-PERCEPTUAL DIZZINESS	0	0	0	0	0	0	0	0	0	0
PRESYNCOPE	8	7	0	1	0	8	7	0	1	0
PARAESTHESIAS AND DYSAESTHESIAS										
BURNING FEET SYNDROME	1	1	0	0	0	1	1	0	0	0
BURNING SENSATION	0	0	0	0	0	0	0	0	0	0
FORMICATION	1	0	1	0	0	1	0	1	0	0
HYPERAESTHESIA	0	0	0	0	0	0	0	0	0	0
HYPOAESTHESIA	45	37	3	5	0	44	37	2	5	0
PARAESTHESIA	35	22	9	3	1	34	22	9	2	1
REVERSED HOT-COLD SENSATION	0	0	0	0	0	0	0	0	0	0
SENSORY ABNORMALITIES NEC										
AGEUSIA	8	5	2	0	1	8	5	2	0	1
ALLODYNIA	0	0	0	0	0	0	0	0	0	0
DYSGEUSIA	17	9	5	3	0	16	9	4	3	0
HYPOGEUSIA	0	0	0	0	0	0	0	0	0	0
NEURALGIA	7	4	2	1	0	6	4	2	0	0
POST HERPETIC NEURALGIA	1	0	0	1	0	1	0	0	1	0
RESTLESS ARM SYNDROME	0	0	0	0	0	0	0	0	0	0
RESTLESS LEGS SYNDROME	0	0	0	0	0	0	0	0	0	0
SENSORY LOSS	1	1	0	0	0	1	1	0	0	0
TASTE DISORDER	7	2	4	1	0	6	2	3	1	0
SPEECH AND LANGUAGE ABNORMALITIES										
DYSARTHRIA	0	0	0	0	0	0	0	0	0	0
SPEECH DISORDER DEVELOPMENTAL	0	0	0	0	0	0	0	0	0	0
NEUROLOGICAL DISORDERS OF THE EYE										
NEUROLOGIC VISUAL PROBLEMS NEC										

TUNNEL VISION	0	0	0	0	0	0	0	0	0	0
NEUROMUSCULAR DISORDERS										
MUSCLE TONE ABNORMAL										
HYPOTONIA	0	0	0	0	0	0	0	0	0	0
STIFF LEG SYNDROME	0	0	0	0	0	0	0	0	0	0
NEUROMUSCULAR DISORDERS NEC										
MUSCLE SPASTICITY	0	0	0	0	0	0	0	0	0	0
PERIPHERAL NEUROPATHIES										
ACUTE POLYNEUROPATHIES										
GUILLAIN-BARRE SYNDROME	0	0	0	0	0	0	0	0	0	0
PERIPHERAL NEUROPATHIES NEC										
AXONAL NEUROPATHY	0	0	0	0	0	0	0	0	0	0
NEUROPATHY PERIPHERAL	1	0	0	1	0	1	0	0	1	0
SEIZURES (INCL SUBTYPES)										
SEIZURES AND SEIZURE DISORDERS NEC										
EPILEPSY	0	0	0	0	0	0	0	0	0	0
SEIZURE	2	2	0	0	0	2	2	0	0	0
SLEEP DISTURBANCES (INCL SUBTYPES)										
DISTURBANCES IN SLEEP PHASE RHYTHM										
CIRCADIAN RHYTHM SLEEP DISORDER	0	0	0	0	0	0	0	0	0	0
SLEEP DISTURBANCES NEC										
SLEEP DEFICIT	1	1	0	0	0	1	1	0	0	0
SPINAL CORD AND NERVE ROOT DISORDERS										
CERVICAL SPINAL CORD AND NERVE ROOT DISORDERS										
CERVICOBRACHIAL SYNDROME	0	0	0	0	0	0	0	0	0	0
LUMBAR SPINAL CORD AND NERVE ROOT DISORDERS										
CAUDA EQUINA SYNDROME	0	0	0	0	0	0	0	0	0	0
SCIATICA	2	0	0	2	0	2	0	0	2	0
**PREGNANCY, PUERPERIUM AND PERINATAL CONDITIONS**										
ABORTIONS AND STILLBIRTH										
ABORTIONS SPONTANEOUS										
ABORTION SPONTANEOUS	15	8	7	0	0	14	8	6	0	0
STILLBIRTH AND FOETAL DEATH										
FOETAL DEATH	0	0	0	0	0	0	0	0	0	0
FOETAL COMPLICATIONS										
FOETAL COMPLICATIONS NEC										

FOETAL DISORDER	0	0	0	0	0	0	0	0	0	0
FOETAL HYPOKINESIA	2	1	0	1	0	1	1	0	0	0
FOETAL GROWTH COMPLICATIONS										
FOETAL MACROSOMIA	1	1	0	0	0	1	1	0	0	0
MATERNAL COMPLICATIONS OF PREGNANCY										
MATERNAL COMPLICATIONS OF PREGNANCY NEC										
MORNING SICKNESS	1	1	0	0	0	1	1	0	0	0
PLACENTAL, AMNIOTIC AND CAVITY DISORDERS (EXCL HAEMORRHAGES)										
PLACENTAL ABNORMALITIES (EXCL NEOPLASMS)										
PLACENTAL INFARCTION	0	0	0	0	0	0	0	0	0	0
PREGNANCY, LABOUR, DELIVERY AND POSTPARTUM CONDITIONS										
NORMAL PREGNANCY, LABOUR AND DELIVERY										
PREGNANCY	0	0	0	0	0	0	0	0	0	0
UTERINE CONTRACTIONS DURING PREGNANCY	1	1	0	0	0	1	1	0	0	0
**PRODUCT ISSUES**										
DEVICE ISSUES										
DEVICE MALFUNCTION EVENTS NEC										
OVERSENSING	0	0	0	0	0	0	0	0	0	0
**PSYCHIATRIC DISORDERS**										
ANXIETY DISORDERS AND SYMPTOMS										
ANXIETY SYMPTOMS										
AGITATION	2	0	2	0	0	2	0	2	0	0
ANXIETY	6	5	1	0	0	5	5	0	0	0
NERVOUSNESS	1	1	0	0	0	1	1	0	0	0
TENSION	1	1	0	0	0	1	1	0	0	0
PANIC ATTACKS AND DISORDERS										
PANIC ATTACK	2	2	0	0	0	2	2	0	0	0
CHANGES IN PHYSICAL ACTIVITY										
INCREASED PHYSICAL ACTIVITY LEVELS										
RESTLESSNESS	2	2	0	0	0	2	2	0	0	0
STEREOTYPIES AND AUTOMATISMS										
BRUXISM	0	0	0	0	0	0	0	0	0	0
COGNITIVE AND ATTENTION DISORDERS AND DISTURBANCES										
COGNITIVE AND ATTENTION DISORDERS AND DISTURBANCES NEC										
DAYDREAMING	0	0	0	0	0	0	0	0	0	0
MENTAL FATIGUE	10	7	1	2	0	9	7	1	1	0

COMMUNICATION DISORDERS AND DISTURBANCES										
SPEECH ARTICULATION AND RHYTHM DISTURBANCES										
DYSPHEMIA	0	0	0	0	0	0	0	0	0	0
DELIRIA (INCL CONFUSION)										
CONFUSION AND DISORIENTATION										
CONFUSIONAL STATE	10	7	1	1	1	9	7	0	1	1
DISORIENTATION	0	0	0	0	0	0	0	0	0	0
DELIRIA										
DELIRIUM	0	0	0	0	0	0	0	0	0	0
DEPRESSED MOOD DISORDERS AND DISTURBANCES										
DEPRESSIVE DISORDERS										
DEPRESSION	5	4	1	0	0	5	4	1	0	0
MOOD ALTERATIONS WITH DEPRESSIVE SYMPTOMS										
DEPRESSED MOOD	3	1	1	1	0	3	1	1	1	0
TEARFULNESS	0	0	0	0	0	0	0	0	0	0
DISSOCIATIVE DISORDERS										
DISSOCIATIVE STATES										
DISSOCIATION	1	0	0	1	0	1	0	0	1	0
DISTURBANCES IN THINKING AND PERCEPTION										
DELUSIONAL SYMPTOMS										
DELUSION	0	0	0	0	0	0	0	0	0	0
HALLUCINATIONS (EXCL SLEEP-RELATED)										
HALLUCINATION	2	2	0	0	0	2	2	0	0	0
PERCEPTION DISTURBANCES NEC										
DEREALISATION	1	1	0	0	0	1	1	0	0	0
THINKING DISTURBANCES										
BRADYPHRENIA	0	0	0	0	0	0	0	0	0	0
THOUGHT BLOCKING	0	0	0	0	0	0	0	0	0	0
MOOD DISORDERS AND DISTURBANCES NEC										
AFFECT ALTERATIONS NEC										
AFFECT LABILITY	0	0	0	0	0	0	0	0	0	0
INAPPROPRIATE AFFECT	1	1	0	0	0	1	1	0	0	0
EMOTIONAL AND MOOD DISTURBANCES NEC										
ANGER	0	0	0	0	0	0	0	0	0	0
EMOTIONAL DISORDER	1	0	1	0	0	1	0	1	0	0
EMOTIONAL DISTRESS	0	0	0	0	0	0	0	0	0	0

EUPHORIC MOOD	0	0	0	0	0	0	0	0	0	0
IRRITABILITY	5	5	0	0	0	5	5	0	0	0
MOOD ALTERED	1	0	1	0	0	0	0	0	0	0
FLUCTUATING MOOD SYMPTOMS										
MOOD SWINGS	1	0	1	0	0	1	0	1	0	0
MOOD DISORDERS NEC										
APATHY	2	1	0	1	0	2	1	0	1	0
LISTLESS	1	0	0	0	1	1	0	0	0	1
PERSONALITY DISORDERS AND DISTURBANCES IN BEHAVIOUR										
BEHAVIOUR AND SOCIALISATION DISTURBANCES										
AGGRESSION	0	0	0	0	0	0	0	0	0	0
PSYCHIATRIC AND BEHAVIOURAL SYMPTOMS NEC										
PSYCHIATRIC SYMPTOMS NEC										
PSYCHIATRIC SYMPTOM	0	0	0	0	0	0	0	0	0	0
PSYCHIATRIC DISORDERS NEC										
MENTAL DISORDERS NEC										
MENTAL DISORDER	0	0	0	0	0	0	0	0	0	0
SCHIZOPHRENIA AND OTHER PSYCHOTIC DISORDERS										
PSYCHOTIC DISORDER NEC										
PSYCHOTIC DISORDER	0	0	0	0	0	0	0	0	0	0
SEXUAL DYSFUNCTIONS, DISTURBANCES AND GENDER IDENTITY DISORDERS										
SEXUAL DESIRE DISORDERS										
LOSS OF LIBIDO	1	1	0	0	0	1	1	0	0	0
SLEEP DISORDERS AND DISTURBANCES										
DISTURBANCES IN INITIATING AND MAINTAINING SLEEP										
INITIAL INSOMNIA	1	1	0	0	0	1	1	0	0	0
INSOMNIA	22	11	4	6	1	19	11	3	5	0
DYSSOMNIAS										
BREATHING-RELATED SLEEP DISORDER	0	0	0	0	0	0	0	0	0	0
POOR QUALITY SLEEP	9	5	1	3	0	9	5	1	3	0
PARASOMNIAS										
ABNORMAL DREAMS	6	4	1	0	1	5	4	1	0	0
EXPLODING HEAD SYNDROME	1	0	1	0	0	1	0	1	0	0
NIGHTMARE	5	1	2	2	0	5	1	2	2	0
SLEEP DISORDERS NEC										
SLEEP DISORDER	2	1	1	0	0	2	1	1	0	0

SOMATIC SYMPTOM AND RELATED DISORDERS										
SOMATIC SYMPTOM DISORDERS										
HABIT COUGH	1	0	0	1	0	1	0	0	1	0
SUICIDAL AND SELF-INJURIOUS BEHAVIOURS NEC										
SUICIDAL AND SELF-INJURIOUS BEHAVIOUR										
SUICIDAL IDEATION	1	1	0	0	0	1	1	0	0	0
**RENAL AND URINARY DISORDERS**										
RENAL DISORDERS (EXCL NEPHROPATHIES)										
RENAL FAILURE AND IMPAIRMENT										
RENAL FAILURE	1	1	0	0	0	1	1	0	0	0
URINARY TRACT SIGNS AND SYMPTOMS										
BLADDER AND URETHRAL SYMPTOMS										
BLADDER PAIN	0	0	0	0	0	0	0	0	0	0
MICTURITION URGENCY	1	0	0	1	0	1	0	0	1	0
POLLAKIURIA	1	1	0	0	0	1	1	0	0	0
URINARY INCONTINENCE	0	0	0	0	0	0	0	0	0	0
URINARY ABNORMALITIES										
HAEMATURIA	1	1	0	0	0	1	1	0	0	0
URINE ABNORMALITY	0	0	0	0	0	0	0	0	0	0
URINE ODOUR ABNORMAL	0	0	0	0	0	0	0	0	0	0
URINARY TRACT SIGNS AND SYMPTOMS NEC										
POLYURIA	0	0	0	0	0	0	0	0	0	0
RENAL PAIN	0	0	0	0	0	0	0	0	0	0
**REPRODUCTIVE SYSTEM AND BREAST DISORDERS**										
BREAST DISORDERS										
BREAST DISORDERS NEC										
BREAST MASS	1	1	0	0	0	1	1	0	0	0
NIPPLE ENLARGEMENT	0	0	0	0	0	0	0	0	0	0
BREAST SIGNS AND SYMPTOMS										
BREAST DISCHARGE	0	0	0	0	0	0	0	0	0	0
BREAST PAIN	4	1	1	2	0	2	1	0	1	0
BREAST SWELLING	0	0	0	0	0	0	0	0	0	0
BREAST TENDERNESS	0	0	0	0	0	0	0	0	0	0
NIPPLE PAIN	0	0	0	0	0	0	0	0	0	0
MENOPAUSE RELATED CONDITIONS										
MENOPAUSAL EFFECTS ON THE GENITOURINARY TRACT										

POSTMENOPAUSAL HAEMORRHAGE	0	0	0	0	0	0	0	0	0	0
MENSTRUAL CYCLE AND UTERINE BLEEDING DISORDERS										
MENSTRUATION AND UTERINE BLEEDING NEC										
DYSMENORRHOEA	19	15	2	2	0	16	15	1	0	0
INTERMENSTRUAL BLEEDING	5	5	0	0	0	5	5	0	0	0
MENSTRUAL DISORDER	12	7	0	5	0	12	7	0	5	0
MENSTRUATION IRREGULAR	23	16	6	1	0	21	16	4	1	0
PREMENSTRUAL PAIN	0	0	0	0	0	0	0	0	0	0
RETROGRADE MENSTRUATION	0	0	0	0	0	0	0	0	0	0
MENSTRUATION WITH DECREASED BLEEDING										
AMENORRHOEA	1	1	0	0	0	1	1	0	0	0
HYPOMENORRHOEA	2	1	1	0	0	2	1	1	0	0
MENSTRUATION DELAYED	20	14	5	1	0	19	14	4	1	0
OLIGOMENORRHOEA	2	1	1	0	0	2	1	1	0	0
MENSTRUATION WITH INCREASED BLEEDING										
HEAVY MENSTRUAL BLEEDING	24	17	2	5	0	23	17	2	4	0
POLYMENORRHOEA	5	4	0	1	0	5	4	0	1	0
PENILE AND SCROTAL DISORDERS (EXCL INFECTIONS AND INFLAMMATIONS)										
SCROTAL DISORDERS NEC										
SCROTAL SWELLING	0	0	0	0	0	0	0	0	0	0
REPRODUCTIVE TRACT DISORDERS NEC										
REPRODUCTIVE TRACT DISORDERS NEC (EXCL NEOPLASMS)										
GENITAL LESION	0	0	0	0	0	0	0	0	0	0
REPRODUCTIVE TRACT SIGNS AND SYMPTOMS NEC										
GENITAL DISCOMFORT	0	0	0	0	0	0	0	0	0	0
GENITAL PAIN	0	0	0	0	0	0	0	0	0	0
UTERINE, PELVIC AND BROAD LIGAMENT DISORDERS										
PELVIS AND BROAD LIGAMENT DISORDERS NEC										
ADNEXA UTERI PAIN	0	0	0	0	0	0	0	0	0	0
VULVOVAGINAL DISORDERS (EXCL INFECTIONS AND INFLAMMATIONS)										
VULVOVAGINAL DISORDERS NEC										
VAGINAL HAEMORRHAGE	9	4	4	1	0	6	4	2	0	0
VULVOVAGINAL SIGNS AND SYMPTOMS										
VAGINAL DISCHARGE	0	0	0	0	0	0	0	0	0	0
**RESPIRATORY, THORACIC AND MEDIASTINAL DISORDERS**										
BRONCHIAL DISORDERS (EXCL NEOPLASMS)										

BRONCHOSPASM AND OBSTRUCTION										
ASTHMA	1	1	0	0	0	1	1	0	0	0
WHEEZING	1	0	0	1	0	0	0	0	0	0
LOWER RESPIRATORY TRACT DISORDERS (EXCL OBSTRUCTION AND INFECTION)										
PULMONARY OEDEMAS										
PULMONARY CONGESTION	1	1	0	0	0	1	1	0	0	0
PULMONARY VASCULAR DISORDERS										
PULMONARY THROMBOTIC AND EMBOLIC CONDITIONS										
PULMONARY EMBOLISM	0	0	0	0	0	0	0	0	0	0
RESPIRATORY DISORDERS NEC										
BREATHING ABNORMALITIES										
DYSPNOEA	36	20	6	8	2	35	20	5	8	2
HYPERVENTILATION	1	1	0	0	0	1	1	0	0	0
HYPOPNOEA	1	0	0	1	0	1	0	0	1	0
IRREGULAR BREATHING	0	0	0	0	0	0	0	0	0	0
MOUTH BREATHING	0	0	0	0	0	0	0	0	0	0
RESPIRATION ABNORMAL	1	1	0	0	0	1	1	0	0	0
RESPIRATORY ARREST	1	1	0	0	0	1	1	0	0	0
RESPIRATORY FATIGUE	0	0	0	0	0	0	0	0	0	0
SLEEP APNOEA SYNDROME	0	0	0	0	0	0	0	0	0	0
COUGHING AND ASSOCIATED SYMPTOMS										
COUGH	49	32	5	11	1	47	32	5	10	0
PRODUCTIVE COUGH	5	2	3	0	0	5	2	3	0	0
RESPIRATORY TRACT DISORDERS NEC										
RESPIRATORY TRACT IRRITATION	0	0	0	0	0	0	0	0	0	0
RESPIRATORY TRACT SIGNS AND SYMPTOMS										
LOWER RESPIRATORY TRACT SIGNS AND SYMPTOMS										
PULMONARY PAIN	1	1	0	0	0	1	1	0	0	0
RESPIRATORY SIGNS AND SYMPTOMS NEC										
RESPIRATORY SYMPTOM	1	1	0	0	0	1	1	0	0	0
UPPER RESPIRATORY TRACT SIGNS AND SYMPTOMS										
APHONIA	1	0	0	1	0	1	0	0	1	0
CATARRH	2	2	0	0	0	2	2	0	0	0
DRY THROAT	4	2	2	0	0	3	2	1	0	0
DYSPHONIA	2	2	0	0	0	2	2	0	0	0
INCREASED UPPER AIRWAY SECRETION	1	1	0	0	0	1	1	0	0	0

INCREASED VISCOSITY OF UPPER RESPIRATORY SE(	1	0	0	1	0	1	0	0	1	0
NASAL DISCOMFORT	0	0	0	0	0	0	0	0	0	0
OROPHARYNGEAL DISCOMFORT	0	0	0	0	0	0	0	0	0	0
OROPHARYNGEAL PAIN	82	57	18	7	0	79	57	16	6	0
PARANASAL SINUS DISCOMFORT	1	1	0	0	0	1	1	0	0	0
RHINALGIA	0	0	0	0	0	0	0	0	0	0
RHINORRHOEA	48	30	8	9	1	41	30	4	6	1
SINUS PAIN	5	3	2	0	0	5	3	2	0	0
SNEEZING	13	10	2	0	1	13	10	2	0	1
THROAT CLEARING	1	1	0	0	0	1	1	0	0	0
THROAT IRRITATION	1	1	0	0	0	1	1	0	0	0
THROAT TIGHTNESS	1	1	0	0	0	1	1	0	0	0
UPPER-AIRWAY COUGH SYNDROME	1	0	0	1	0	1	0	0	1	0
YAWNING	0	0	0	0	0	0	0	0	0	0
UPPER RESPIRATORY TRACT DISORDERS (EXCL INFECTIONS)										
NASAL CONGESTION AND INFLAMMATIONS										
NASAL CONGESTION	5	2	3	0	0	4	2	2	0	0
RHINITIS ALLERGIC	1	1	0	0	0	1	1	0	0	0
RHINITIS ATROPHIC	0	0	0	0	0	0	0	0	0	0
NASAL DISORDERS NEC										
EPISTAXIS	8	5	1	2	0	6	5	1	0	0
NASAL DRYNESS	2	2	0	0	0	2	2	0	0	0
PARANASAL SINUS DISORDERS (EXCL INFECTIONS AND NEOPLASMS)										
SINUS CONGESTION	1	1	0	0	0	1	1	0	0	0
PHARYNGEAL DISORDERS (EXCL INFECTIONS AND NEOPLASMS)										
PHARYNGEAL SWELLING	0	0	0	0	0	0	0	0	0	0
PHARYNGEAL ULCERATION	0	0	0	0	0	0	0	0	0	0
TONSILLAR ERYTHEMA	0	0	0	0	0	0	0	0	0	0
TRACHEAL DISORDERS (EXCL INFECTIONS AND NEOPLASMS)										
TRACHEAL PAIN	0	0	0	0	0	0	0	0	0	0
**SKIN AND SUBCUTANEOUS TISSUE DISORDERS**										
ANGIOEDEMA AND URTICARIA										
ANGIOEDEMAS										
ANGIOEDEMA	0	0	0	0	0	0	0	0	0	0
URTICARIAS										

COLD URTICARIA	0	0	0	0	0	0	0	0	0	0
SOLAR URTICARIA	0	0	0	0	0	0	0	0	0	0
URTICARIA	10	4	1	5	0	10	4	1	5	0
URTICARIA CHRONIC	0	0	0	0	0	0	0	0	0	0
CORNIFICATION AND DYSTROPHIC SKIN DISORDERS										
SKIN DYSTROPHIES										
HYPERTROPHIC SCAR	1	1	0	0	0	1	1	0	0	0
CUTANEOUS NEOPLASMS BENIGN										
SKIN CYSTS AND POLYPS										
DERMAL CYST	1	1	0	0	0	1	1	0	0	0
EPIDERMAL AND DERMAL CONDITIONS										
BULLOUS CONDITIONS										
BLISTER	1	1	0	0	0	1	1	0	0	0
BLOOD BLISTER	0	0	0	0	0	0	0	0	0	0
PEMPHIGOID	1	0	0	0	1	1	0	0	0	1
TOXIC EPIDERMAL NECROLYSIS	1	1	0	0	0	1	1	0	0	0
DERMAL AND EPIDERMAL CONDITIONS NEC										
DRY SKIN	2	2	0	0	0	2	2	0	0	0
PAIN OF SKIN	12	8	1	3	0	12	8	1	3	0
SENSITIVE SKIN	7	3	2	1	1	5	3	1	1	0
SKIN BURNING SENSATION	3	2	0	1	0	3	2	0	1	0
SKIN DISCOLOURATION	1	1	0	0	0	1	1	0	0	0
SKIN FRAGILITY	1	1	0	0	0	1	1	0	0	0
SKIN LESION	0	0	0	0	0	0	0	0	0	0
SKIN ODOUR ABNORMAL	0	0	0	0	0	0	0	0	0	0
SKIN REACTION	1	0	0	1	0	1	0	0	1	0
SKIN SENSITISATION	0	0	0	0	0	0	0	0	0	0
SKIN WARM	14	9	1	4	0	14	9	1	4	0
DERMATITIS AND ECZEMA										
DERMATITIS	2	2	0	0	0	2	2	0	0	0
DERMATITIS ALLERGIC	0	0	0	0	0	0	0	0	0	0
DERMATITIS ATOPIC	0	0	0	0	0	0	0	0	0	0
DERMATITIS CONTACT	0	0	0	0	0	0	0	0	0	0
ECZEMA	4	3	1	0	0	4	3	1	0	0
ECZEMA ASTEATOTIC	1	0	0	1	0	1	0	0	1	0

NEURODERMATITIS	0	0	0	0	0	0	0	0	0	0
SEBORRHOEIC DERMATITIS	0	0	0	0	0	0	0	0	0	0
SKIN IRRITATION	2	0	1	1	0	1	0	0	1	0
ERYTHEMAS										
ERYTHEMA	21	12	8	1	0	20	12	8	0	0
EXFOLIATIVE CONDITIONS										
SKIN EXFOLIATION	0	0	0	0	0	0	0	0	0	0
PAPULOSQUAMOUS CONDITIONS										
LICHEN PLANUS	0	0	0	0	0	0	0	0	0	0
PITYRIASIS ROSEA	0	0	0	0	0	0	0	0	0	0
PHOTOSENSITIVITY AND PHOTODERMATOSIS CONDITIONS										
PHOTOSENSITIVITY REACTION	1	1	0	0	0	1	1	0	0	0
PRURITUS NEC										
PRURITUS	51	30	13	8	0	45	30	9	6	0
PSORIATIC CONDITIONS										
PSORIASIS	2	0	1	1	0	2	0	1	1	0
RASHES, ERUPTIONS AND EXANTHEMS NEC										
RASH	46	31	5	10	0	42	31	5	6	0
RASH ERYTHEMATOUS	9	4	1	4	0	9	4	1	4	0
RASH MACULAR	2	2	0	0	0	2	2	0	0	0
RASH MORBILLIFORM	1	0	0	1	0	0	0	0	0	0
RASH PAPULAR	2	1	1	0	0	2	1	1	0	0
RASH PRURITIC	6	3	2	1	0	6	3	2	1	0
SKIN INJURIES AND MECHANICAL DERMATOSES										
DECUBITUS ULCER	1	0	0	1	0	0	0	0	0	0
NEEDLE TRACK MARKS	0	0	0	0	0	0	0	0	0	0
SKIN AND SUBCUTANEOUS TISSUE DISORDERS NEC										
SKIN AND SUBCUTANEOUS TISSUE ULCERATIONS										
SKIN EROSION	0	0	0	0	0	0	0	0	0	0
SKIN APPENDAGE CONDITIONS										
ACNES										
ACNE	0	0	0	0	0	0	0	0	0	0
ACNE CYSTIC	0	0	0	0	0	0	0	0	0	0
ALOPECIAS										
ALOPECIA	3	3	0	0	0	3	3	0	0	0
APOCRINE AND ECCRINE GLAND DISORDERS										

COLD SWEAT	6	2	3	0	1	5	2	2	0	1
HYPERHIDROSIS	27	19	5	3	0	26	19	5	2	0
MILIARIA	3	1	2	0	0	2	1	1	0	0
NIGHT SWEATS	20	9	6	4	1	17	9	5	3	0
HYPERTRICHOSES										
HIRSUTISM	1	1	0	0	0	1	1	0	0	0
NAIL AND NAIL BED CONDITIONS (EXCL INFECTIONS AND INFESTATIONS)										
NAIL DISCOLOURATION	0	0	0	0	0	0	0	0	0	0
ONYCHOCLASIS	0	0	0	0	0	0	0	0	0	0
PILAR DISORDERS NEC										
PILOERECTION	0	0	0	0	0	0	0	0	0	0
SKIN VASCULAR ABNORMALITIES										
PURPURA AND RELATED CONDITIONS										
PETECHIAE	0	0	0	0	0	0	0	0	0	0
PURPURA	0	0	0	0	0	0	0	0	0	0
SKIN HAEMORRHAGES										
SKIN HAEMORRHAGE	0	0	0	0	0	0	0	0	0	0
**SOCIAL CIRCUMSTANCES**										
ECONOMIC AND HOUSING ISSUES										
EMPLOYMENT ISSUES										
RETIREMENT	1	1	0	0	0	1	1	0	0	0
ENVIRONMENTAL ISSUES										
NON-OCCUPATIONAL AND UNSPECIFIED ENVIRONMENTAL PROBLEMS										
POLLUTION	0	0	0	0	0	0	0	0	0	0
**SURGICAL AND MEDICAL PROCEDURES**										
BONE AND JOINT THERAPEUTIC PROCEDURES										
JOINT THERAPEUTIC PROCEDURES										
KNEE OPERATION	0	0	0	0	0	0	0	0	0	0
BREAST THERAPEUTIC PROCEDURES										
MASTECTOMIES										
BREAST CONSERVING SURGERY	0	0	0	0	0	0	0	0	0	0
GASTROINTESTINAL THERAPEUTIC PROCEDURES										
GASTROINTESTINAL THERAPEUTIC PROCEDURES NEC										
PROPHYLAXIS OF NAUSEA AND VOMITING	0	0	0	0	0	0	0	0	0	0
HEAD AND NECK THERAPEUTIC PROCEDURES										
DENTAL AND GINGIVAL THERAPEUTIC PROCEDURES										

DENTAL CARE	0	0	0	0	0	0	0	0	0	0
NERVOUS SYSTEM, SKULL AND SPINE THERAPEUTIC PROCEDURES										
SKULL AND BRAIN THERAPEUTIC PROCEDURES										
BRAIN TUMOUR OPERATION	0	0	0	0	0	0	0	0	0	0
OBSTETRIC AND GYNAECOLOGICAL THERAPEUTIC PROCEDURES										
FERTILITY AND FERTILISATION INTERVENTIONS FEMALE										
ENDOMETRIAL SCRATCHING	1	1	0	0	0	1	1	0	0	0
RESPIRATORY TRACT THERAPEUTIC PROCEDURES										
RESPIRATORY TRACT THERAPEUTIC PROCEDURES NEC										
OXYGEN THERAPY	0	0	0	0	0	0	0	0	0	0
THERAPEUTIC PROCEDURES AND SUPPORTIVE CARE NEC										
ANAESTHESIA AND ALLIED PROCEDURES										
NERVE BLOCK	0	0	0	0	0	0	0	0	0	0
DIETARY AND NUTRITIONAL THERAPIES										
NOTHING BY MOUTH ORDER	0	0	0	0	0	0	0	0	0	0
IMMUNISATIONS										
COVID-19 IMMUNISATION	0	0	0	0	0	0	0	0	0	0
THERAPEUTIC PROCEDURES NEC										
INJECTION	1	1	0	0	0	1	1	0	0	0
LOCALISED ALTERNATING HOT AND COLD THERAPY	0	0	0	0	0	0	0	0	0	0
MASS EXCISION	0	0	0	0	0	0	0	0	0	0
REINFUSION	0	0	0	0	0	0	0	0	0	0
**VASCULAR DISORDERS**										
ARTERIOSCLEROSIS, STENOSIS, VASCULAR INSUFFICIENCY AND NECROSIS										
NON-SITE SPECIFIC NECROSIS AND VASCULAR INSUFFICIENCY NEC										
VASOSPASM	0	0	0	0	0	0	0	0	0	0
PERIPHERAL VASOCONSTRICTION, NECROSIS AND VASCULAR INSUFFICIENCY										
PERIPHERAL COLDNESS	4	3	1	0	0	4	3	1	0	0
RAYNAUD'S PHENOMENON	0	0	0	0	0	0	0	0	0	0
DECREASED AND NONSPECIFIC BLOOD PRESSURE DISORDERS AND SHOCK										
BLOOD PRESSURE DISORDERS NEC										
BLOOD PRESSURE FLUCTUATION	0	0	0	0	0	0	0	0	0	0
VASCULAR HYPOTENSIVE DISORDERS										
CAPILLARY LEAK SYNDROME	0	0	0	0	0	0	0	0	0	0
HYPOTENSION	4	4	0	0	0	4	4	0	0	0

ORTHOSTATIC HYPOTENSION	1	0	1	0	0	1	0	1	0	0
EMBOLISM AND THROMBOSIS										
NON-SITE SPECIFIC EMBOLISM AND THROMBOSIS										
EMBOLISM	0	0	0	0	0	0	0	0	0	0
THROMBOSIS	3	3	0	0	0	3	3	0	0	0
VENOUS THROMBOSIS	0	0	0	0	0	0	0	0	0	0
PERIPHERAL EMBOLISM AND THROMBOSIS										
BLUE TOE SYNDROME	0	0	0	0	0	0	0	0	0	0
DEEP VEIN THROMBOSIS	0	0	0	0	0	0	0	0	0	0
SUPERFICIAL VEIN THROMBOSIS	0	0	0	0	0	0	0	0	0	0
LYMPHATIC VESSEL DISORDERS										
LYMPHOEDEMAS										
LYMPHOEDEMA	4	2	0	2	0	3	2	0	1	0
VASCULAR DISORDERS NEC										
NON-SITE SPECIFIC VASCULAR DISORDERS NEC										
VASCULAR PAIN	1	0	1	0	0	1	0	1	0	0
VEIN DISCOLOURATION	0	0	0	0	0	0	0	0	0	0
VEIN RUPTURE	0	0	0	0	0	0	0	0	0	0
PERIPHERAL VASCULAR DISORDERS NEC										
FLUSHING	7	5	1	1	0	6	5	0	1	0
HOT FLUSH	27	19	6	2	0	26	19	5	2	0
SITE SPECIFIC VASCULAR DISORDERS NEC										
PALLOR	2	2	0	0	0	2	2	0	0	0
VASCULAR HAEMORRHAGIC DISORDERS										
HAEMORRHAGES NEC										
HAEMATOMA	0	0	0	0	0	0	0	0	0	0
HAEMORRHAGE	4	2	0	2	0	4	2	0	2	0
VASCULAR HYPERTENSIVE DISORDERS										
ACCELERATED AND MALIGNANT HYPERTENSION										
HYPERTENSIVE URGENCY	1	1	0	0	0	1	1	0	0	0
VASCULAR HYPERTENSIVE DISORDERS NEC										
HYPERTENSION	6	5	0	1	0	6	5	0	1	0
SYSTOLIC HYPERTENSION	0	0	0	0	0	0	0	0	0	0
VASCULAR INFECTIONS AND INFLAMMATIONS										
ARTERIAL INFECTIONS AND INFLAMMATIONS										
GIANT CELL ARTERITIS	0	0	0	0	0	0	0	0	0	0

PHLEBITIS NEC										
PHLEBITIS	1	0	0	1	0	0	0	0	0	0
VASCULITIDES NEC										
VASCULITIS	0	0	0	0	0	0	0	0	0	0
VENOUS VARICES										
VARICOSE VEINS NEC										
SPIDER VEIN	1	1	0	0	0	1	1	0	0	0
VARICOSE VEIN	1	1	0	0	0	1	1	0	0	0
**TOTAL ADR EVENTS**	***9949***	***5705***	***1954***	***2141***	***149***	***9139***	***5705***	***1576***	***1735***	***123***

SUPPLEMENTARY TABLE 17. AstraZeneca COVID-19 vaccine: ADR listing for events reported in the YCVM in (a) those reporting any vaccination dose and (b) in those who had reported a 1st dose vaccination and any

	Individuals with any vaccination dose: ADR Counts					Individuals reporting a 1st dose: ADR Counts				
**MEDDRA REACTION TERM (SOC, *HLGT*, *HLT*, PT)**	All doses	1st dose	2nd dose	3rd dose	Other doses	All doses	1st dose	2nd dose	3rd dose	Other doses
(freetext)	35	33	2	0	0	34	33	1	0	0
**BLOOD AND LYMPHATIC SYSTEM DISORDERS**										
COAGULOPATHIES AND BLEEDING DIATHESES (EXCL THROMBOCYTOPENIC)										
BLEEDING TENDENCIES										
INCREASED TENDENCY TO BRUISE	1	0	1	0	0	1	0	1	0	0
HAEMOGLOBINOPATHIES										
SICKLE CELL TRAIT AND DISORDERS										
SICKLE CELL ANAEMIA WITH CRISIS	1	1	0	0	0	1	1	0	0	0
PLATELET DISORDERS										
THROMBOCYTOPENIAS										
IMMUNE THROMBOCYTOPENIA	1	0	1	0	0	0	0	0	0	0
SPLEEN, LYMPHATIC AND RETICULOENDOTHELIAL SYSTEM DISORDERS										
LYMPHATIC SYSTEM DISORDERS NEC										
LYMPH NODE PAIN	14	9	5	0	0	14	9	5	0	0
LYMPHADENITIS	0	0	0	0	0	0	0	0	0	0
LYMPHADENOPATHY	108	79	29	0	0	98	79	19	0	0
**CARDIAC DISORDERS**										
CARDIAC ARRHYTHMIAS										
RATE AND RHYTHM DISORDERS NEC										
ARRHYTHMIA	1	1	0	0	0	1	1	0	0	0
CARDIAC FLUTTER	4	2	2	0	0	4	2	2	0	0
EXTRASYSTOLES	0	0	0	0	0	0	0	0	0	0
TACHYCARDIA	15	13	2	0	0	13	13	0	0	0
SUPRAVENTRICULAR ARRHYTHMIAS										
ATRIAL FIBRILLATION	3	2	1	0	0	2	2	0	0	0
SUPRAVENTRICULAR TACHYCARDIA	1	1	0	0	0	1	1	0	0	0

CARDIAC DISORDERS, SIGNS AND SYMPTOMS NEC										
CARDIAC DISORDERS NEC										
CARDIOVASCULAR DISORDER	1	1	0	0	0	1	1	0	0	0
CARDIAC SIGNS AND SYMPTOMS NEC										
PALPITATIONS	58	50	7	1	0	57	50	6	1	0
CORONARY ARTERY DISORDERS										
ISCHAEMIC CORONARY ARTERY DISORDERS										
ANGINA PECTORIS	1	1	0	0	0	1	1	0	0	0
MYOCARDIAL INFARCTION	1	1	0	0	0	1	1	0	0	0
HEART FAILURES										
HEART FAILURES NEC										
CARDIAC FAILURE	0	0	0	0	0	0	0	0	0	0
MYOCARDIAL DISORDERS										
NONINFECTIOUS MYOCARDITIS										
MYOCARDITIS	0	0	0	0	0	0	0	0	0	0
PERICARDIAL DISORDERS										
NONINFECTIOUS PERICARDITIS										
PERICARDITIS	0	0	0	0	0	0	0	0	0	0
**CONGENITAL, FAMILIAL AND GENETIC DISORDERS**										
CARDIAC AND VASCULAR DISORDERS CONGENITAL										
CARDIAC DISORDERS CONGENITAL NEC										
HEART DISEASE CONGENITAL	0	0	0	0	0	0	0	0	0	0
METABOLIC AND NUTRITIONAL DISORDERS CONGENITAL										
INBORN ERRORS OF AMINO ACID METABOLISM										
HYPERGLYCINAEMIA	1	1	0	0	0	1	1	0	0	0
INBORN ERRORS OF STEROID SYNTHESIS										
11-BETA-HYDROXYLASE DEFICIENCY	0	0	0	0	0	0	0	0	0	0
NEUROLOGICAL DISORDERS CONGENITAL										
PERIPHERAL NERVOUS SYSTEM DISORDERS CONGENITAL NEC										
PAROXYSMAL EXTREME PAIN DISORDER	1	0	1	0	0	1	0	1	0	0
**EAR AND LABYRINTH DISORDERS**										

AURAL DISORDERS NEC										
EAR DISORDERS NEC										
EAR DISCOMFORT	3	2	1	0	0	3	2	1	0	0
EAR DISORDER	1	0	1	0	0	1	0	1	0	0
EAR PAIN	36	28	8	0	0	32	28	4	0	0
EAR SWELLING	1	1	0	0	0	1	1	0	0	0
EXTERNAL EAR DISORDERS (EXCL CONGENITAL)										
EXTERNAL EAR DISORDERS NEC										
EXCESSIVE CERUMEN PRODUCTION	2	2	0	0	0	2	2	0	0	0
HEARING DISORDERS										
HEARING LOSSES										
DEAFNESS	1	0	1	0	0	0	0	0	0	0
DEAFNESS NEUROSENSORY	0	0	0	0	0	0	0	0	0	0
DEAFNESS PERMANENT	0	0	0	0	0	0	0	0	0	0
DEAFNESS UNILATERAL	2	1	1	0	0	2	1	1	0	0
HYPOACUSIS	2	1	1	0	0	2	1	1	0	0
SUDDEN HEARING LOSS	1	1	0	0	0	1	1	0	0	0
HYPERACUSIA										
HYPERACUSIS	1	0	1	0	0	1	0	1	0	0
INNER EAR AND VIIITH CRANIAL NERVE DISORDERS										
INNER EAR SIGNS AND SYMPTOMS										
MOTION SICKNESS	1	1	0	0	0	1	1	0	0	0
TINNITUS	47	37	10	0	0	44	37	7	0	0
VERTIGO	31	24	7	0	0	30	24	6	0	0
VERTIGO LABYRINTHINE	2	2	0	0	0	2	2	0	0	0
VERTIGO POSITIONAL	4	3	1	0	0	4	3	1	0	0
**ENDOCRINE DISORDERS**										
THYROID GLAND DISORDERS										
THYROID HYPERFUNCTION DISORDERS										
HYPERTHYROIDISM	1	1	0	0	0	1	1	0	0	0
**EYE DISORDERS**										
EYE DISORDERS NEC										
LACRIMATION DISORDERS										
DRY EYE	3	3	0	0	0	3	3	0	0	0
LACRIMATION INCREASED	0	0	0	0	0	0	0	0	0	0
OCULAR DISORDERS NEC										

EYE OEDEMA	1	1	0	0	0	1	1	0	0	0
EYE PAIN	61	57	4	0	0	60	57	3	0	0
EYE SWELLING	1	1	0	0	0	1	1	0	0	0
EYE ULCER	1	1	0	0	0	1	1	0	0	0
EYELID PAIN	1	1	0	0	0	1	1	0	0	0
OCULAR DISCOMFORT	2	2	0	0	0	2	2	0	0	0
PERIORBITAL DISCOMFORT	1	0	1	0	0	0	0	0	0	0
PERIORBITAL SWELLING	1	0	1	0	0	1	0	1	0	0
OCULAR HAEMORRHAGES AND VASCULAR DISORDERS NEC										
CONJUNCTIVAL AND CORNEAL BLEEDING AND VASCULAR DISORDERS										
CONJUNCTIVAL HAEMORRHAGE	0	0	0	0	0	0	0	0	0	0
LID BLEEDING AND VASCULAR DISORDERS										
EYELID BLEEDING	1	1	0	0	0	1	1	0	0	0
OCULAR INFECTIONS, IRRITATIONS AND INFLAMMATIONS										
LID, LASH AND LACRIMAL INFECTIONS, IRRITATIONS AND INFLAMMATIONS										
BLEPHARITIS	1	1	0	0	0	1	1	0	0	0
ERYTHEMA OF EYELID	1	1	0	0	0	1	1	0	0	0
EYELID IRRITATION	1	1	0	0	0	1	1	0	0	0
OCULAR INFECTIONS, INFLAMMATIONS AND ASSOCIATED MANIFESTATIONS										
EYE DISCHARGE	1	1	0	0	0	1	1	0	0	0
EYE IRRITATION	0	0	0	0	0	0	0	0	0	0
EYE PRURITUS	4	4	0	0	0	4	4	0	0	0
LIMBAL SWELLING	0	0	0	0	0	0	0	0	0	0
OCULAR HYPERAEMIA	5	5	0	0	0	5	5	0	0	0
OCULAR NEUROMUSCULAR DISORDERS										
EYELID MOVEMENT DISORDERS										
BLEPHAROSPASM	1	1	0	0	0	1	1	0	0	0
OCULAR SENSORY SYMPTOMS NEC										
OCULAR SENSATION DISORDERS										
ABNORMAL SENSATION IN EYE	0	0	0	0	0	0	0	0	0	0
ASTHENOPIA	11	10	1	0	0	11	10	1	0	0
FOREIGN BODY SENSATION IN EYES	1	1	0	0	0	1	1	0	0	0
PHOTOPHOBIA	16	13	3	0	0	15	13	2	0	0

OCULAR STRUCTURAL CHANGE, DEPOSIT AND DEGENERATION NEC										
CHOROID AND VITREOUS STRUCTURAL CHANGE, DEPOSIT AND DEGENERATION										
VITREOUS DETACHMENT	1	1	0	0	0	1	1	0	0	0
VITREOUS FLOATERS	2	2	0	0	0	2	2	0	0	0
RETINA, CHOROID AND VITREOUS HAEMORRHAGES AND VASCULAR DISORDERS										
RETINAL BLEEDING AND VASCULAR DISORDERS (EXCL RETINOPATHY)										
RETINAL VEIN OCCLUSION	1	0	1	0	0	1	0	1	0	0
RETINOPATHIES NEC										
RETINAL EXUDATES	1	1	0	0	0	1	1	0	0	0
VISION DISORDERS										
VISUAL DISORDERS NEC										
DIPLOPIA	1	1	0	0	0	1	1	0	0	0
HALO VISION	2	1	1	0	0	2	1	1	0	0
METAMORPHOPSIA	1	1	0	0	0	1	1	0	0	0
PHOTOPSIA	1	1	0	0	0	1	1	0	0	0
VISION BLURRED	25	20	5	0	0	24	20	4	0	0
VISUAL IMPAIRMENT AND BLINDNESS (EXCL COLOUR BLINDNESS)										
BLINDNESS	1	0	1	0	0	1	0	1	0	0
BLINDNESS TRANSIENT	1	1	0	0	0	1	1	0	0	0
SUDDEN VISUAL LOSS	1	1	0	0	0	1	1	0	0	0
VISUAL IMPAIRMENT	10	10	0	0	0	10	10	0	0	0
**GASTROINTESTINAL DISORDERS**										
ANAL AND RECTAL CONDITIONS NEC										
ANAL AND RECTAL SIGNS AND SYMPTOMS										
ANAL PARAESTHESIA	1	1	0	0	0	1	1	0	0	0
BENIGN NEOPLASMS GASTROINTESTINAL										
BENIGN ORAL CAVITY NEOPLASMS										
MOUTH CYST	1	1	0	0	0	1	1	0	0	0
DENTAL AND GINGIVAL CONDITIONS										
DENTAL DISORDERS NEC										
TEETHING	0	0	0	0	0	0	0	0	0	0

DENTAL PAIN AND SENSATION DISORDERS										
DENTAL PARAESTHESIA	1	1	0	0	0	1	1	0	0	0
TOOTHACHE	6	4	2	0	0	6	4	2	0	0
GINGIVAL DISORDERS, SIGNS AND SYMPTOMS NEC										
GINGIVAL BLISTER	1	1	0	0	0	1	1	0	0	0
GINGIVAL PAIN	6	4	2	0	0	6	4	2	0	0
GINGIVAL SWELLING	1	1	0	0	0	1	1	0	0	0
GASTROINTESTINAL CONDITIONS NEC										
GASTROINTESTINAL MUCOSAL DYSTROPHIES AND SECRETION DISORDERS										
BARRETT'S OESOPHAGUS	1	1	0	0	0	1	1	0	0	0
GASTROINTESTINAL HAEMORRHAGES NEC										
INTESTINAL HAEMORRHAGES										
RECTAL HAEMORRHAGE	1	1	0	0	0	1	1	0	0	0
NON-SITE SPECIFIC GASTROINTESTINAL HAEMORRHAGES										
HAEMATEMESIS	1	1	0	0	0	1	1	0	0	0
GASTROINTESTINAL INFLAMMATORY CONDITIONS										
COLITIS (EXCL INFECTIVE)										
COLITIS	0	0	0	0	0	0	0	0	0	0
COLITIS MICROSCOPIC	1	0	1	0	0	1	0	1	0	0
COLITIS ULCERATIVE	1	1	0	0	0	1	1	0	0	0
GASTRITIS (EXCL INFECTIVE)										
GASTRITIS	2	2	0	0	0	2	2	0	0	0
REFLUX GASTRITIS	1	1	0	0	0	1	1	0	0	0
GASTROINTESTINAL INFLAMMATORY DISORDERS NEC										
GASTROINTESTINAL TRACT IRRITATION	0	0	0	0	0	0	0	0	0	0
GASTROINTESTINAL MOTILITY AND DEFAECATION CONDITIONS										
DIARRHOEA (EXCL INFECTIVE)										
DIARRHOEA	201	173	28	0	0	191	173	18	0	0
GASTROINTESTINAL ATONIC AND HYPOMOTILITY DISORDERS NEC										
CONSTIPATION	4	2	2	0	0	3	2	1	0	0
GASTROOESOPHAGEAL REFLUX DISEASE	2	1	1	0	0	2	1	1	0	0
GASTROINTESTINAL DYSKINETIC DISORDERS										
CHANGE OF BOWEL HABIT	0	0	0	0	0	0	0	0	0	0
GASTROINTESTINAL SPASTIC AND HYPERMOTILITY DISORDERS										
IRRITABLE BOWEL SYNDROME	2	1	1	0	0	2	1	1	0	0
GASTROINTESTINAL SIGNS AND SYMPTOMS										

DYSPEPTIC SIGNS AND SYMPTOMS										
DYSPEPSIA	26	21	5	0	0	24	21	3	0	0
ERUCTATION	1	1	0	0	0	1	1	0	0	0
FAECAL ABNORMALITIES NEC										
FAECALOMA	0	0	0	0	0	0	0	0	0	0
FAECES DISCOLOURED	0	0	0	0	0	0	0	0	0	0
FAECES SOFT	2	1	1	0	0	2	1	1	0	0
FLATULENCE, BLOATING AND DISTENSION										
ABDOMINAL DISTENSION	9	9	0	0	0	9	9	0	0	0
FLATULENCE	14	13	1	0	0	14	13	1	0	0
GASTROINTESTINAL AND ABDOMINAL PAINS (EXCL ORAL AND THROAT)										
ABDOMINAL PAIN	61	50	11	0	0	57	50	7	0	0
ABDOMINAL PAIN LOWER	4	3	1	0	0	3	3	0	0	0
ABDOMINAL PAIN UPPER	91	76	15	0	0	86	76	10	0	0
GASTROINTESTINAL PAIN	7	7	0	0	0	7	7	0	0	0
GASTROINTESTINAL SIGNS AND SYMPTOMS NEC										
ABDOMINAL DISCOMFORT	57	45	12	0	0	54	45	9	0	0
ABDOMINAL SYMPTOM	1	1	0	0	0	1	1	0	0	0
ACUTE ABDOMEN	1	1	0	0	0	1	1	0	0	0
ANAL INCONTINENCE	0	0	0	0	0	0	0	0	0	0
BREATH ODOUR	0	0	0	0	0	0	0	0	0	0
DYSPHAGIA	0	0	0	0	0	0	0	0	0	0
ODYNOPHAGIA	1	1	0	0	0	1	1	0	0	0
NAUSEA AND VOMITING SYMPTOMS										
NAUSEA	897	789	108	0	0	860	789	71	0	0
RETCHING	3	3	0	0	0	3	3	0	0	0
VOMITING	102	93	9	0	0	101	93	8	0	0
VOMITING PROJECTILE	2	2	0	0	0	2	2	0	0	0
MALABSORPTION CONDITIONS										
MALABSORPTION SYNDROMES										
COELIAC DISEASE	1	0	1	0	0	1	0	1	0	0
ORAL SOFT TISSUE CONDITIONS										
ORAL SOFT TISSUE DISORDERS NEC										
CHEILITIS	1	1	0	0	0	1	1	0	0	0
LIP BLISTER	2	2	0	0	0	2	2	0	0	0

ORAL LICHEN PLANUS	1	1	0	0	0	1	1	0	0	0
ORAL SOFT TISSUE INFECTIONS										
ANGULAR CHEILITIS	1	1	0	0	0	1	1	0	0	0
ORAL SOFT TISSUE SIGNS AND SYMPTOMS										
HYPOAESTHESIA ORAL	2	2	0	0	0	2	2	0	0	0
LIP PAIN	3	2	1	0	0	3	2	1	0	0
ORAL DISCOMFORT	2	2	0	0	0	2	2	0	0	0
ORAL MUCOSAL ROUGHENING	0	0	0	0	0	0	0	0	0	0
ORAL PAIN	8	7	1	0	0	7	7	0	0	0
PARAESTHESIA ORAL	18	16	2	0	0	17	16	1	0	0
LIP SWELLING	6	6	0	0	0	6	6	0	0	0
MOUTH SWELLING	1	1	0	0	0	1	1	0	0	0
STOMATITIS AND ULCERATION										
APHTHOUS ULCER	1	0	1	0	0	0	0	0	0	0
LIP ULCERATION	0	0	0	0	0	0	0	0	0	0
MOUTH ULCERATION	6	4	2	0	0	6	4	2	0	0
STOMATITIS	1	0	1	0	0	1	0	1	0	0
SALIVARY GLAND CONDITIONS										
ORAL DRYNESS AND SALIVA ALTERED										
DRY MOUTH	19	15	4	0	0	18	15	3	0	0
LIP DRY	4	3	1	0	0	4	3	1	0	0
SALIVARY HYPOSECRETION	1	1	0	0	0	1	1	0	0	0
TONGUE CONDITIONS										
TONGUE SIGNS AND SYMPTOMS										
GLOSSODYNIA	3	2	1	0	0	3	2	1	0	0
SWOLLEN TONGUE	4	4	0	0	0	4	4	0	0	0
TONGUE COATED	1	1	0	0	0	1	1	0	0	0
TONGUE DISCOMFORT	1	1	0	0	0	1	1	0	0	0
TONGUE OEDEMA	0	0	0	0	0	0	0	0	0	0
TONGUE SPASM	0	0	0	0	0	0	0	0	0	0
**GENERAL DISORDERS AND ADMINISTRATION SITE CONDITIONS**										
ADMINISTRATION SITE REACTIONS										
ADMINISTRATION SITE REACTIONS NEC										
ADMINISTRATION SITE BRUISE	2	2	0	0	0	2	2	0	0	0
ADMINISTRATION SITE PAIN	0	0	0	0	0	0	0	0	0	0

PUNCTURE SITE BRUISE	7	5	2	0	0	7	5	2	0	0
PUNCTURE SITE PAIN	1	1	0	0	0	1	1	0	0	0
APPLICATION SITE REACTIONS										
APPLICATION SITE BRUISE	5	4	1	0	0	5	4	1	0	0
APPLICATION SITE ERYTHEMA	2	1	1	0	0	2	1	1	0	0
APPLICATION SITE PAIN	3	2	1	0	0	3	2	1	0	0
IMPLANT AND CATHETER SITE REACTIONS										
IMPLANT SITE PAIN	1	0	1	0	0	1	0	1	0	0
IMPLANT SITE WARMTH	2	2	0	0	0	2	2	0	0	0
INFUSION SITE REACTIONS										
INFUSION SITE PAIN	1	1	0	0	0	1	1	0	0	0
INFUSION SITE SCAB	1	1	0	0	0	1	1	0	0	0
INFUSION SITE WARMTH	1	1	0	0	0	1	1	0	0	0
INJECTION SITE REACTIONS										
INJECTION SITE BRUISING	2	1	1	0	0	2	1	1	0	0
INJECTION SITE DISCOMFORT	1	1	0	0	0	1	1	0	0	0
INJECTION SITE ERYTHEMA	15	13	2	0	0	14	13	1	0	0
INJECTION SITE HYPERSENSITIVITY	0	0	0	0	0	0	0	0	0	0
INJECTION SITE INFLAMMATION	1	1	0	0	0	1	1	0	0	0
INJECTION SITE INJURY	0	0	0	0	0	0	0	0	0	0
INJECTION SITE IRRITATION	0	0	0	0	0	0	0	0	0	0
INJECTION SITE JOINT PAIN	2	1	1	0	0	1	1	0	0	0
INJECTION SITE MASS	28	25	3	0	0	27	25	2	0	0
INJECTION SITE NODULE	0	0	0	0	0	0	0	0	0	0
INJECTION SITE OEDEMA	1	1	0	0	0	1	1	0	0	0
INJECTION SITE PAIN	229	180	49	0	0	219	180	39	0	0
INJECTION SITE PAPULE	1	1	0	0	0	1	1	0	0	0
INJECTION SITE PARAESTHESIA	0	0	0	0	0	0	0	0	0	0
INJECTION SITE PRURITUS	8	7	1	0	0	7	7	0	0	0
INJECTION SITE RASH	4	4	0	0	0	4	4	0	0	0
INJECTION SITE REACTION	2	2	0	0	0	2	2	0	0	0
INJECTION SITE SCAB	2	1	1	0	0	2	1	1	0	0
INJECTION SITE SWELLING	8	7	1	0	0	8	7	1	0	0
INJECTION SITE URTICARIA	0	0	0	0	0	0	0	0	0	0
INJECTION SITE WARMTH	10	9	1	0	0	10	9	1	0	0

INSTILLATION SITE REACTIONS										
INSTILLATION SITE PRURITUS	0	0	0	0	0	0	0	0	0	0
INSTILLATION SITE WARMTH	1	1	0	0	0	1	1	0	0	0
VACCINATION SITE REACTIONS										
SHOULDER INJURY RELATED TO VACCINE ADMINISTRATION	1	1	0	0	0	1	1	0	0	0
VACCINATION SITE BRUISING	4	1	3	0	0	3	1	2	0	0
VACCINATION SITE DISCOMFORT	0	0	0	0	0	0	0	0	0	0
VACCINATION SITE ERYTHEMA	0	0	0	0	0	0	0	0	0	0
VACCINATION SITE JOINT ERYTHEMA	1	1	0	0	0	1	1	0	0	0
VACCINATION SITE JOINT PAIN	0	0	0	0	0	0	0	0	0	0
VACCINATION SITE MASS	12	9	3	0	0	10	9	1	0	0
VACCINATION SITE PAIN	41	32	9	0	0	38	32	6	0	0
VACCINATION SITE RASH	1	1	0	0	0	1	1	0	0	0
VACCINATION SITE SWELLING	0	0	0	0	0	0	0	0	0	0
VACCINATION SITE WARMTH	4	3	1	0	0	3	3	0	0	0
BODY TEMPERATURE CONDITIONS										
BODY TEMPERATURE ALTERED										
HYPERTHERMIA	1	1	0	0	0	1	1	0	0	0
HYPOTHERMIA	0	0	0	0	0	0	0	0	0	0
FEBRILE DISORDERS										
PYREXIA	1644	1470	174	0	0	1560	1470	90	0	0
FATAL OUTCOMES										
DEATH AND SUDDEN DEATH										
DEATH	2	2	0	0	0	2	2	0	0	0
GENERAL SYSTEM DISORDERS NEC										
ADVERSE EFFECT ABSENT										
NO ADVERSE EVENT	0	0	0	0	0	0	0	0	0	0
ASTHENIC CONDITIONS										
ASTHENIA	74	60	14	0	0	71	60	11	0	0
CHRONIC FATIGUE SYNDROME	1	1	0	0	0	1	1	0	0	0
DECREASED ACTIVITY	0	0	0	0	0	0	0	0	0	0
FATIGUE	2711	2205	503	3	0	2565	2205	359	1	0
MALAISE	270	229	40	1	0	256	229	26	1	0
SLUGGISHNESS	1	0	1	0	0	1	0	1	0	0
FEELINGS AND SENSATIONS NEC										

CHILLS	1369	1235	132	2	0	1310	1235	74	1	0
FEELING ABNORMAL	78	63	15	0	0	72	63	9	0	0
FEELING COLD	264	226	37	1	0	242	226	16	0	0
FEELING HOT	65	54	11	0	0	62	54	8	0	0
FEELING JITTERY	1	0	1	0	0	1	0	1	0	0
FEELING OF BODY TEMPERATURE CHANGE	42	37	5	0	0	39	37	2	0	0
FEELING OF RELAXATION	1	1	0	0	0	1	1	0	0	0
HANGOVER	6	6	0	0	0	6	6	0	0	0
HUNGER	3	2	0	1	0	2	2	0	0	0
SENSATION OF BLOOD FLOW	1	1	0	0	0	1	1	0	0	0
THIRST	32	29	3	0	0	30	29	1	0	0
GAIT DISTURBANCES										
GAIT DISTURBANCE	3	2	1	0	0	3	2	1	0	0
GAIT INABILITY	2	2	0	0	0	2	2	0	0	0
GENERAL SIGNS AND SYMPTOMS NEC										
CRYING	1	1	0	0	0	1	1	0	0	0
ENERGY INCREASED	1	0	1	0	0	1	0	1	0	0
EXERCISE TOLERANCE DECREASED	1	1	0	0	0	1	1	0	0	0
GENERAL SYMPTOM	1	1	0	0	0	1	1	0	0	0
ILLNESS	85	75	10	0	0	79	75	4	0	0
INFLUENZA LIKE ILLNESS	488	410	77	1	0	447	410	37	0	0
LOCAL REACTION	3	3	0	0	0	3	3	0	0	0
PERIPHERAL SWELLING	103	84	19	0	0	100	84	16	0	0
SWELLING	34	26	8	0	0	32	26	6	0	0
SWELLING FACE	8	7	1	0	0	8	7	1	0	0
TISSUE IRRITATION	1	0	1	0	0	1	0	1	0	0
INFLAMMATIONS										
INFLAMMATION	7	4	3	0	0	5	4	1	0	0
SYSTEMIC INFLAMMATORY RESPONSE SYNDROME	1	0	1	0	0	0	0	0	0	0
OEDEMA NEC										
OEDEMA	1	1	0	0	0	1	1	0	0	0
OEDEMA PERIPHERAL	1	1	0	0	0	1	1	0	0	0
PAIN AND DISCOMFORT NEC										
AXILLARY PAIN	25	20	5	0	0	24	20	4	0	0

CHEST DISCOMFORT	24	22	2	0	0	23	22	1	0	0
CHEST PAIN	47	40	7	0	0	44	40	4	0	0
DISCOMFORT	7	5	2	0	0	6	5	1	0	0
FACIAL PAIN	2	2	0	0	0	2	2	0	0	0
HERNIA PAIN	0	0	0	0	0	0	0	0	0	0
INFLAMMATORY PAIN	1	1	0	0	0	1	1	0	0	0
NON-CARDIAC CHEST PAIN	0	0	0	0	0	0	0	0	0	0
PAIN	578	485	93	0	0	542	485	57	0	0
TENDERNESS	202	173	28	1	0	196	173	22	1	0
THERAPEUTIC AND NONTHERAPEUTIC EFFECTS (EXCL TOXICITY)										
THERAPEUTIC AND NONTHERAPEUTIC RESPONSES										
ADVERSE DRUG REACTION	1	1	0	0	0	1	1	0	0	0
ADVERSE EVENT	0	0	0	0	0	0	0	0	0	0
ADVERSE REACTION	0	0	0	0	0	0	0	0	0	0
IMMEDIATE POST-INJECTION REACTION	0	0	0	0	0	0	0	0	0	0
**HEPATOBILIARY DISORDERS**										
HEPATIC AND HEPATOBILIARY DISORDERS										
HEPATOBILIARY SIGNS AND SYMPTOMS										
HEPATIC PAIN	2	2	0	0	0	2	2	0	0	0
HEPATOCELLULAR DAMAGE AND HEPATITIS NEC										
LIVER INJURY	1	1	0	0	0	1	1	0	0	0
**IMMUNE SYSTEM DISORDERS**										
ALLERGIC CONDITIONS										
ALLERGIC CONDITIONS NEC										
HYPERSENSITIVITY	7	4	3	0	0	5	4	1	0	0
MULTIPLE ALLERGIES	1	1	0	0	0	1	1	0	0	0
ALLERGIES TO FOODS, FOOD ADDITIVES, DRUGS AND OTHER CHEMICALS										
ALLERGY TO CHEMICALS	1	0	1	0	0	1	0	1	0	0
ANAPHYLACTIC AND ANAPHYLACTOID RESPONSES										
ANAPHYLACTIC REACTION	3	3	0	0	0	3	3	0	0	0
ATOPIC DISORDERS										
SEASONAL ALLERGY	1	0	1	0	0	1	0	1	0	0
IMMUNE DISORDERS NEC										
IMMUNE AND ASSOCIATED CONDITIONS NEC										
BACILLE CALMETTE-GUERIN SCAR REACTIVATION	1	1	0	0	0	1	1	0	0	0

SENSITISATION	1	1	0	0	0	1	1	0	0	0
**INFECTIONS AND INFESTATIONS**										
BACTERIAL INFECTIOUS DISORDERS										
BACTERIAL INFECTIONS NEC										
CELLULITIS	2	2	0	0	0	2	2	0	0	0
PERIORBITAL CELLULITIS	0	0	0	0	0	0	0	0	0	0
HELICOBACTER INFECTIONS										
HELICOBACTER GASTRITIS	0	0	0	0	0	0	0	0	0	0
STAPHYLOCOCCAL INFECTIONS										
FURUNCLE	2	1	1	0	0	1	1	0	0	0
FUNGAL INFECTIOUS DISORDERS										
CANDIDA INFECTIONS										
CANDIDA INFECTION	0	0	0	0	0	0	0	0	0	0
VULVOVAGINAL CANDIDIASIS	2	2	0	0	0	2	2	0	0	0
INFECTIONS - PATHOGEN UNSPECIFIED										
ABDOMINAL AND GASTROINTESTINAL INFECTIONS										
APPENDICITIS	1	1	0	0	0	1	1	0	0	0
DIARRHOEA INFECTIOUS	0	0	0	0	0	0	0	0	0	0
GASTROINTESTINAL INFECTION	0	0	0	0	0	0	0	0	0	0
BREAST INFECTIONS										
MASTITIS	1	0	1	0	0	1	0	1	0	0
CENTRAL NERVOUS SYSTEM AND SPINAL INFECTIONS										
MYELITIS	1	1	0	0	0	1	1	0	0	0
DENTAL AND ORAL SOFT TISSUE INFECTIONS										
ABSCESS ORAL	0	0	0	0	0	0	0	0	0	0
ORAL PUSTULE	1	1	0	0	0	1	1	0	0	0
PERICORONITIS	1	1	0	0	0	1	1	0	0	0
TOOTH ABSCESS	1	1	0	0	0	1	1	0	0	0
EAR INFECTIONS										
EAR INFECTION	1	1	0	0	0	1	1	0	0	0
LABYRINTHITIS	0	0	0	0	0	0	0	0	0	0
OTITIS EXTERNA	1	1	0	0	0	1	1	0	0	0
EYE AND EYELID INFECTIONS										
CONJUNCTIVITIS	1	0	1	0	0	1	0	1	0	0
HORDEOLUM	1	1	0	0	0	1	1	0	0	0
INFECTIONS NEC										

ABSCESS	1	1	0	0	0	1	1	0	0	0
INFECTION	0	0	0	0	0	0	0	0	0	0
INJECTION SITE INFECTION	1	1	0	0	0	1	1	0	0	0
LOCALISED INFECTION	3	3	0	0	0	3	3	0	0	0
WOUND INFECTION	1	0	1	0	0	0	0	0	0	0
LOWER RESPIRATORY TRACT AND LUNG INFECTIONS										
LOWER RESPIRATORY TRACT INFECTION	3	2	1	0	0	3	2	1	0	0
PNEUMONIA	3	2	1	0	0	2	2	0	0	0
MALE REPRODUCTIVE TRACT INFECTIONS										
ORCHITIS	1	1	0	0	0	1	1	0	0	0
SEPSIS, BACTERAEMIA, VIRAEMIA AND FUNGAEMIA NEC										
SEPTIC RASH	1	0	1	0	0	0	0	0	0	0
SKIN STRUCTURES AND SOFT TISSUE INFECTIONS										
INFECTED DERMAL CYST	0	0	0	0	0	0	0	0	0	0
INJECTION SITE PUSTULE	1	0	1	0	0	0	0	0	0	0
SKIN INFECTION	0	0	0	0	0	0	0	0	0	0
UPPER RESPIRATORY TRACT INFECTIONS										
LARYNGITIS	0	0	0	0	0	0	0	0	0	0
NASOPHARYNGITIS	120	94	26	0	0	112	94	18	0	0
PHARYNGITIS	1	1	0	0	0	1	1	0	0	0
RHINITIS	1	1	0	0	0	1	1	0	0	0
SINUSITIS	9	8	1	0	0	8	8	0	0	0
TONSILLITIS	0	0	0	0	0	0	0	0	0	0
URINARY TRACT INFECTIONS										
CYSTITIS	0	0	0	0	0	0	0	0	0	0
URINARY TRACT INFECTION	3	2	1	0	0	2	2	0	0	0
PROTOZOAL INFECTIOUS DISORDERS										
TRYPANOSOMAL INFECTIONS										
AFRICAN TRYPANOSOMIASIS	0	0	0	0	0	0	0	0	0	0
RICKETTSIAL INFECTIOUS DISORDERS										
COXIELLA INFECTIONS										
Q FEVER	4	4	0	0	0	4	4	0	0	0
VIRAL INFECTIOUS DISORDERS										
CORONAVIRUS INFECTIONS										
COVID-19	1	0	1	0	0	0	0	0	0	0
HERPES VIRAL INFECTIONS										

GENITAL HERPES	0	0	0	0	0	0	0	0	0	0
HERPES OPHTHALMIC	1	1	0	0	0	1	1	0	0	0
HERPES SIMPLEX	1	0	1	0	0	0	0	0	0	0
HERPES ZOSTER	6	4	2	0	0	6	4	2	0	0
NASAL HERPES	1	1	0	0	0	1	1	0	0	0
OPHTHALMIC HERPES ZOSTER	1	1	0	0	0	1	1	0	0	0
ORAL HERPES	18	15	3	0	0	17	15	2	0	0
INFLUENZA VIRAL INFECTIONS										
H2N2 INFLUENZA	1	1	0	0	0	1	1	0	0	0
INFLUENZA	463	409	54	0	0	440	409	31	0	0
RETROVIRAL INFECTIONS										
AIDS RELATED COMPLEX	1	1	0	0	0	1	1	0	0	0
VIRAL INFECTIONS NEC										
GASTROENTERITIS VIRAL	4	4	0	0	0	4	4	0	0	0
SWEATING FEVER	7	7	0	0	0	7	7	0	0	0
VESTIBULAR NEURONITIS	0	0	0	0	0	0	0	0	0	0
VIRAL DIARRHOEA	0	0	0	0	0	0	0	0	0	0
VIRAL RASH	1	1	0	0	0	1	1	0	0	0
VIRAL UPPER RESPIRATORY TRACT INFECTION	1	1	0	0	0	1	1	0	0	0
**INJURY, POISONING AND PROCEDURAL COMPLICATIONS**										
BONE AND JOINT INJURIES										
FRACTURES AND DISLOCATIONS NEC										
JOINT DISLOCATION	1	1	0	0	0	1	1	0	0	0
EXPOSURES, CHEMICAL INJURIES AND POISONING										
POISONING AND TOXICITY										
SYSTEMIC TOXICITY	1	1	0	0	0	1	1	0	0	0
TOXICITY TO VARIOUS AGENTS	1	1	0	0	0	1	1	0	0	0
INJURIES BY PHYSICAL AGENTS										
CONDITIONS CAUSED BY COLD										
CHILLBLAINS	1	1	0	0	0	1	1	0	0	0
HEAT INJURIES (EXCL THERMAL BURNS)										
HEAT EXHAUSTION	1	1	0	0	0	1	1	0	0	0
HEAT OEDEMA	1	1	0	0	0	1	1	0	0	0
RADIATION INJURIES										
SUNBURN	1	0	1	0	0	0	0	0	0	0
THERMAL BURNS										

THERMAL BURN	1	1	0	0	0	1	1	0	0	0
THERMAL BURNS OF EYE	1	1	0	0	0	1	1	0	0	0
INJURIES NEC										
CHEST AND RESPIRATORY TRACT INJURIES NEC										
CHEST CRUSHING	1	1	0	0	0	1	1	0	0	0
EYE INJURIES NEC										
EYE CONTUSION	0	0	0	0	0	0	0	0	0	0
MUSCLE, TENDON AND LIGAMENT INJURIES										
LIGAMENT SPRAIN	1	1	0	0	0	1	1	0	0	0
MUSCLE INJURY	1	1	0	0	0	1	1	0	0	0
MUSCLE STRAIN	2	1	1	0	0	2	1	1	0	0
NERVE INJURIES NEC										
NERVE INJURY	1	1	0	0	0	1	1	0	0	0
NON-SITE SPECIFIC INJURIES NEC										
ARTHROPOD STING	0	0	0	0	0	0	0	0	0	0
BITE	1	0	1	0	0	1	0	1	0	0
FALL	3	3	0	0	0	3	3	0	0	0
INFLAMMATION OF WOUND	1	1	0	0	0	1	1	0	0	0
TISSUE INJURY	1	1	0	0	0	1	1	0	0	0
WOUND COMPLICATION	1	1	0	0	0	1	1	0	0	0
WOUND SECRETION	1	1	0	0	0	1	1	0	0	0
SITE SPECIFIC INJURIES NEC										
LIMB INJURY	6	6	0	0	0	6	6	0	0	0
SKIN INJURIES NEC										
CONTUSION	70	53	17	0	0	63	53	10	0	0
SCAR	1	0	1	0	0	1	0	1	0	0
SKIN WOUND	1	0	1	0	0	0	0	0	0	0
PROCEDURAL RELATED INJURIES AND COMPLICATIONS NEC										
CARDIAC AND VASCULAR PROCEDURAL COMPLICATIONS										
CARDIAC PROCEDURE COMPLICATION	1	0	1	0	0	0	0	0	0	0
GASTROINTESTINAL AND HEPATOBILIARY PROCEDURAL COMPLICATIONS										
PROCEDURAL NAUSEA	6	6	0	0	0	6	6	0	0	0
NEUROLOGICAL AND PSYCHIATRIC PROCEDURAL COMPLICATIONS										
PROCEDURAL DIZZINESS	2	2	0	0	0	2	2	0	0	0
NON-SITE SPECIFIC PROCEDURAL COMPLICATIONS										
INCISION SITE PAIN	0	0	0	0	0	0	0	0	0	0

INCISION SITE SWELLING	0	0	0	0	0	0	0	0	0	0
INJECTION RELATED REACTION	43	39	3	1	0	43	39	3	1	0
POST PROCEDURAL COMPLICATION	5	3	2	0	0	3	3	0	0	0
PROCEDURAL PAIN	0	0	0	0	0	0	0	0	0	0
VACCINATION RELATED COMPLICATIONS										
IMMUNISATION REACTION	5	2	3	0	0	4	2	2	0	0
**INVESTIGATIONS**										
CARDIAC AND VASCULAR INVESTIGATIONS (EXCL ENZYME TESTS)										
HEART RATE AND PULSE INVESTIGATIONS										
HEART RATE	18	16	2	0	0	18	16	2	0	0
HEART RATE DECREASED	1	1	0	0	0	1	1	0	0	0
HEART RATE INCREASED	28	25	3	0	0	26	25	1	0	0
HEART RATE IRREGULAR	0	0	0	0	0	0	0	0	0	0
VASCULAR TESTS NEC (INCL BLOOD PRESSURE)										
BLOOD PRESSURE DECREASED	1	0	1	0	0	1	0	1	0	0
BLOOD PRESSURE INCREASED	4	3	1	0	0	4	3	1	0	0
BLOOD PRESSURE MEASUREMENT	1	1	0	0	0	1	1	0	0	0
ENDOCRINE INVESTIGATIONS (INCL SEX HORMONES)										
ENDOCRINE ANALYSES AND IMAGING NEC										
HORMONE LEVEL ABNORMAL	0	0	0	0	0	0	0	0	0	0
PITUITARY ANALYSES ANTERIOR										
BLOOD FOLLICLE STIMULATING HORMONE INCREASED	0	0	0	0	0	0	0	0	0	0
BLOOD LUTEINISING HORMONE	0	0	0	0	0	0	0	0	0	0
THYROID ANALYSES										
TRI-IODOTHYRONINE	1	1	0	0	0	1	1	0	0	0
HAEMATOLOGY INVESTIGATIONS (INCL BLOOD GROUPS)										
COAGULATION AND BLEEDING ANALYSES										
BLEEDING TIME	0	0	0	0	0	0	0	0	0	0
INTERNATIONAL NORMALISED RATIO DECREASED	1	0	1	0	0	0	0	0	0	0
PLATELET ANALYSES										
PLATELET COUNT INCREASED	1	0	1	0	0	1	0	1	0	0
RED BLOOD CELL ANALYSES										
HAEMOGLOBIN	1	1	0	0	0	1	1	0	0	0
METABOLIC, NUTRITIONAL AND BLOOD GAS INVESTIGATIONS										
BLOOD GAS AND ACID BASE ANALYSES										

OXYGEN SATURATION DECREASED	2	2	0	0	0	2	2	0	0	0
CARBOHYDRATE TOLERANCE ANALYSES (INCL DIABETES)										
BLOOD GLUCOSE	2	2	0	0	0	2	2	0	0	0
BLOOD GLUCOSE ABNORMAL	1	1	0	0	0	1	1	0	0	0
BLOOD GLUCOSE DECREASED	1	1	0	0	0	1	1	0	0	0
BLOOD GLUCOSE INCREASED	4	4	0	0	0	4	4	0	0	0
MICROBIOLOGY AND SEROLOGY INVESTIGATIONS										
VIRUS IDENTIFICATION AND SEROLOGY										
SARS-COV-2 TEST	0	0	0	0	0	0	0	0	0	0
SARS-COV-2 TEST POSITIVE	0	0	0	0	0	0	0	0	0	0
MUSCULOSKELETAL AND SOFT TISSUE INVESTIGATIONS (EXCL ENZYME TESTS)										
MUSCULOSKELETAL AND SOFT TISSUE IMAGING PROCEDURES										
BONE SCAN	1	1	0	0	0	1	1	0	0	0
NEUROLOGICAL, SPECIAL SENSES AND PSYCHIATRIC INVESTIGATIONS										
CENTRAL NERVOUS SYSTEM IMAGING PROCEDURES										
MAGNETIC RESONANCE IMAGING HEAD	6	6	0	0	0	6	6	0	0	0
SCAN BRAIN	0	0	0	0	0	0	0	0	0	0
PHYSICAL EXAMINATION AND ORGAN SYSTEM STATUS TOPICS										
PHYSICAL EXAMINATION PROCEDURES AND ORGAN SYSTEM STATUS										
BODY TEMPERATURE	49	42	7	0	0	47	42	5	0	0
BODY TEMPERATURE ABNORMAL	1	1	0	0	0	1	1	0	0	0
BODY TEMPERATURE DECREASED	2	2	0	0	0	2	2	0	0	0
BODY TEMPERATURE FLUCTUATION	8	7	1	0	0	8	7	1	0	0
BODY TEMPERATURE INCREASED	45	36	7	1	1	42	36	5	1	0
GRIP STRENGTH DECREASED	1	1	0	0	0	1	1	0	0	0
HEAD LAG	1	1	0	0	0	1	1	0	0	0
LEFT-HANDEDNESS	0	0	0	0	0	0	0	0	0	0
LYMPH NODE PALPABLE	0	0	0	0	0	0	0	0	0	0
RESPIRATORY RATE DECREASED	0	0	0	0	0	0	0	0	0	0
SKIN TEMPERATURE	4	3	1	0	0	4	3	1	0	0
WEIGHT DECREASED	1	1	0	0	0	1	1	0	0	0
WEIGHT INCREASED	2	2	0	0	0	2	2	0	0	0
RENAL AND URINARY TRACT INVESTIGATIONS AND URINALYSES										
URINALYSIS NEC										
BLOOD URINE	2	2	0	0	0	2	2	0	0	0

NITRITE URINE PRESENT	1	1	0	0	0	1	1	0	0	0
PH URINE	0	0	0	0	0	0	0	0	0	0
URINARY TRACT FUNCTION ANALYSES NEC										
URINE OUTPUT	2	2	0	0	0	2	2	0	0	0
URINE OUTPUT INCREASED	1	1	0	0	0	1	1	0	0	0
REPRODUCTIVE ORGAN AND BREAST INVESTIGATIONS (EXCL HORMONE ANALYSES)										
REPRODUCTIVE ORGAN AND BREAST IMAGING PROCEDURES										
BREAST SCAN	0	0	0	0	0	0	0	0	0	0
RESPIRATORY AND PULMONARY INVESTIGATIONS (EXCL BLOOD GASES)										
RESPIRATORY AND PULMONARY FUNCTION DIAGNOSTIC PROCEDURES										
FORCED EXPIRATORY VOLUME DECREASED	1	1	0	0	0	1	1	0	0	0
FORCED EXPIRATORY VOLUME INCREASED	0	0	0	0	0	0	0	0	0	0
WATER, ELECTROLYTE AND MINERAL INVESTIGATIONS										
WATER AND ELECTROLYTE ANALYSES NEC										
VOLUME BLOOD	0	0	0	0	0	0	0	0	0	0
**METABOLISM AND NUTRITION DISORDERS**										
APPETITE AND GENERAL NUTRITIONAL DISORDERS										
APPETITE DISORDERS										
APPETITE DISORDER	0	0	0	0	0	0	0	0	0	0
DECREASED APPETITE	108	97	11	0	0	102	97	5	0	0
FOOD CRAVING	0	0	0	0	0	0	0	0	0	0
FOOD REFUSAL	1	1	0	0	0	1	1	0	0	0
INCREASED APPETITE	1	1	0	0	0	1	1	0	0	0
GENERAL NUTRITIONAL DISORDERS NEC										
FOOD AVERSION	2	2	0	0	0	2	2	0	0	0
ELECTROLYTE AND FLUID BALANCE CONDITIONS										
TOTAL FLUID VOLUME DECREASED										
DEHYDRATION	13	12	1	0	0	12	12	0	0	0
TOTAL FLUID VOLUME INCREASED										
FLUID RETENTION	1	0	0	1	0	0	0	0	0	0
FOOD INTOLERANCE SYNDROMES										
FOOD MALABSORPTION AND INTOLERANCE SYNDROMES (EXCL SUGAR INTOLERANCE)										
ALCOHOL INTOLERANCE	1	1	0	0	0	1	1	0	0	0
GLUCOSE METABOLISM DISORDERS (INCL DIABETES MELLITUS)										
DIABETES MELLITUS (INCL SUBTYPES)										
DIABETES MELLITUS	0	0	0	0	0	0	0	0	0	0

DIABETES MELLITUS INADEQUATE CONTROL	1	1	0	0	0	1	1	0	0	0
HYPERGLYCAEMIC CONDITIONS NEC										
HYPERGLYCAEMIA	0	0	0	0	0	0	0	0	0	0
HYPOGLYCAEMIC CONDITIONS NEC										
HYPOGLYCAEMIA	2	2	0	0	0	2	2	0	0	0
PURINE AND PYRIMIDINE METABOLISM DISORDERS										
DISORDERS OF PURINE METABOLISM										
GOUT	1	1	0	0	0	1	1	0	0	0
**MUSCULOSKELETAL AND CONNECTIVE TISSUE DISORDERS**										
BONE DISORDERS (EXCL CONGENITAL AND FRACTURES)										
BONE DISORDERS NEC										
JAW DISORDER	1	1	0	0	0	1	1	0	0	0
OSTEITIS	1	1	0	0	0	1	1	0	0	0
BONE RELATED SIGNS AND SYMPTOMS										
BONE PAIN	12	11	1	0	0	11	11	0	0	0
BONE SWELLING	0	0	0	0	0	0	0	0	0	0
PAIN IN JAW	10	7	3	0	0	10	7	3	0	0
PUBIC PAIN	1	1	0	0	0	1	1	0	0	0
SPINAL PAIN	2	2	0	0	0	2	2	0	0	0
CONNECTIVE TISSUE DISORDERS (EXCL CONGENITAL)										
CONNECTIVE TISSUE DISORDERS NEC										
POLYMYALGIA RHEUMATICA	2	1	1	0	0	2	1	1	0	0
LUPUS ERYTHEMATOSUS (INCL SUBTYPES)										
SYSTEMIC LUPUS ERYTHEMATOSUS	1	1	0	0	0	1	1	0	0	0
JOINT DISORDERS										
ARTHROPATHIES NEC										
ARTHRITIS	7	6	1	0	0	7	6	1	0	0
ARTHROPATHY	1	1	0	0	0	1	1	0	0	0
POLYARTHRITIS	0	0	0	0	0	0	0	0	0	0
RHEUMATIC FEVER	1	1	0	0	0	1	1	0	0	0
JOINT RELATED DISORDERS NEC										
JOINT LOCK	1	1	0	0	0	1	1	0	0	0
PERIARTHRITIS	3	1	2	0	0	3	1	2	0	0
TEMPOROMANDIBULAR PAIN AND DYSFUNCTION SYNDROME	0	0	0	0	0	0	0	0	0	0
JOINT RELATED SIGNS AND SYMPTOMS										

ARTHRALGIA	892	760	131	1	0	852	760	91	1	0
JAW CLICKING	1	1	0	0	0	1	1	0	0	0
JOINT STIFFNESS	25	21	4	0	0	23	21	2	0	0
JOINT SWELLING	5	4	1	0	0	5	4	1	0	0
OSTEOARTHROPATHIES										
OSTEOARTHRITIS	1	0	1	0	0	0	0	0	0	0
PSORIATIC ARTHROPATHIES										
PSORIATIC ARTHROPATHY	1	1	0	0	0	1	1	0	0	0
RHEUMATOID ARTHROPATHIES										
RHEUMATOID ARTHRITIS	2	2	0	0	0	2	2	0	0	0
MUSCLE DISORDERS										
MUSCLE INFECTIONS AND INFLAMMATIONS										
MYOSITIS	0	0	0	0	0	0	0	0	0	0
MUSCLE PAINS										
FIBROMYALGIA	2	2	0	0	0	2	2	0	0	0
MYALGIA	1174	992	179	3	0	1117	992	123	2	0
MUSCLE RELATED SIGNS AND SYMPTOMS NEC										
MUSCLE ATROPHY	1	1	0	0	0	1	1	0	0	0
MUSCLE DISCOMFORT	0	0	0	0	0	0	0	0	0	0
MUSCLE FATIGUE	31	27	4	0	0	30	27	3	0	0
MUSCLE FIBROSIS	0	0	0	0	0	0	0	0	0	0
MUSCLE MASS	3	3	0	0	0	3	3	0	0	0
MUSCLE SPASMS	36	27	8	1	0	32	27	5	0	0
MUSCLE TIGHTNESS	3	2	1	0	0	3	2	1	0	0
MUSCLE TWITCHING	8	8	0	0	0	8	8	0	0	0
MUSCLE TONE ABNORMALITIES										
MUSCLE RIGIDITY	0	0	0	0	0	0	0	0	0	0
TRISMUS	1	1	0	0	0	1	1	0	0	0
MUSCLE WEAKNESS CONDITIONS										
MUSCULAR WEAKNESS	36	32	4	0	0	36	32	4	0	0
MUSCULOSKELETAL AND CONNECTIVE TISSUE DISORDERS NEC										
MUSCULOSKELETAL AND CONNECTIVE TISSUE CONDITIONS NEC										
MOBILITY DECREASED	1	1	0	0	0	1	1	0	0	0
MUSCULOSKELETAL STIFFNESS	123	101	22	0	0	117	101	16	0	0
BACK PAIN	128	112	16	0	0	122	112	10	0	0

FLANK PAIN	1	1	0	0	0	1	1	0	0	0
LIMB DISCOMFORT	307	235	71	1	0	294	235	59	0	0
MUSCULOSKELETAL CHEST PAIN	6	4	2	0	0	6	4	2	0	0
MUSCULOSKELETAL DISCOMFORT	12	10	2	0	0	12	10	2	0	0
MUSCULOSKELETAL PAIN	4	1	3	0	0	4	1	3	0	0
NECK PAIN	72	58	14	0	0	71	58	13	0	0
PAIN IN EXTREMITY	1509	1200	306	3	0	1427	1200	225	2	0
SOFT TISSUE DISORDERS NEC										
AXILLARY MASS	0	0	0	0	0	0	0	0	0	0
GROIN PAIN	3	3	0	0	0	3	3	0	0	0
SYNOVIAL AND BURSAL DISORDERS										
SYNOVIAL DISORDERS										
SYNOVITIS	0	0	0	0	0	0	0	0	0	0
TENDON, LIGAMENT AND CARTILAGE DISORDERS										
CARTILAGE DISORDERS										
COSTOCHONDRITIS	2	2	0	0	0	2	2	0	0	0
TENDON DISORDERS										
TENDONITIS	1	1	0	0	0	1	1	0	0	0
TENOSYNOVITIS	0	0	0	0	0	0	0	0	0	0
TRIGGER FINGER	0	0	0	0	0	0	0	0	0	0
**NEOPLASMS BENIGN, MALIGNANT AND UNSPECIFIED (INCL CYSTS AND POLYPS)**										
CUTANEOUS NEOPLASMS BENIGN										
SKIN NEOPLASMS BENIGN										
MELANOCYTIC NAEVUS	0	0	0	0	0	0	0	0	0	0
SEBORRHOEIC KERATOSIS	0	0	0	0	0	0	0	0	0	0
SKIN PAPILLOMA	0	0	0	0	0	0	0	0	0	0
**NERVOUS SYSTEM DISORDERS**										
CENTRAL NERVOUS SYSTEM INFECTIONS AND INFLAMMATIONS										
MYELITIS (INCL INFECTIVE)										
MYELITIS TRANSVERSE	1	1	0	0	0	1	1	0	0	0
CENTRAL NERVOUS SYSTEM VASCULAR DISORDERS										
CENTRAL NERVOUS SYSTEM HAEMORRHAGES AND CEREBROVASCULAR ACCIDENTS										
CEREBRAL HAEMORRHAGE	1	0	1	0	0	1	0	1	0	0
CEREBROVASCULAR ACCIDENT	5	4	1	0	0	5	4	1	0	0
TRANSIENT CEREBROVASCULAR EVENTS										
TRANSIENT ISCHAEMIC ATTACK	5	4	1	0	0	4	4	0	0	0

CRANIAL NERVE DISORDERS (EXCL NEOPLASMS)										
FACIAL CRANIAL NERVE DISORDERS										
BELL'S PALSY	1	1	0	0	0	1	1	0	0	0
FACIAL PARALYSIS	0	0	0	0	0	0	0	0	0	0
FACIAL PARESIS	0	0	0	0	0	0	0	0	0	0
OLFACTORY NERVE DISORDERS										
ANOSMIA	6	6	0	0	0	6	6	0	0	0
HYPOSMIA	1	1	0	0	0	1	1	0	0	0
PAROSMIA	10	5	5	0	0	9	5	4	0	0
TRIGEMINAL DISORDERS										
TRIGEMINAL NEURALGIA	2	2	0	0	0	2	2	0	0	0
TRIGEMINAL NEURITIS	0	0	0	0	0	0	0	0	0	0
DEMYELINATING DISORDERS										
MULTIPLE SCLEROSIS ACUTE AND PROGRESSIVE										
MULTIPLE SCLEROSIS RELAPSE	1	1	0	0	0	1	1	0	0	0
HEADACHES										
HEADACHES NEC										
CLUSTER HEADACHE	19	19	0	0	0	19	19	0	0	0
COLD-STIMULUS HEADACHE	1	1	0	0	0	1	1	0	0	0
DRUG WITHDRAWAL HEADACHE	1	0	1	0	0	1	0	1	0	0
HEADACHE	3605	3063	541	1	0	3414	3063	351	0	0
PRIMARY STABBING HEADACHE	2	2	0	0	0	2	2	0	0	0
SINUS HEADACHE	42	37	5	0	0	40	37	3	0	0
TENSION HEADACHE	82	66	16	0	0	78	66	12	0	0
THUNDERCLAP HEADACHE	1	1	0	0	0	1	1	0	0	0
VASCULAR HEADACHE	1	1	0	0	0	1	1	0	0	0
MIGRAINE HEADACHES										
MIGRAINE	94	81	13	0	0	88	81	7	0	0
MIGRAINE WITH AURA	3	1	2	0	0	2	1	1	0	0
RETINAL MIGRAINE	2	2	0	0	0	2	2	0	0	0
TYPICAL AURA WITHOUT HEADACHE	0	0	0	0	0	0	0	0	0	0
MENTAL IMPAIRMENT DISORDERS										
INTELLECTUAL DISABILITIES										
INTELLECTUAL DISABILITY	0	0	0	0	0	0	0	0	0	0
MEMORY LOSS (EXCL DEMENTIA)										

AMNESIA	3	2	1	0	0	3	2	1	0	0
MEMORY IMPAIRMENT	6	5	1	0	0	6	5	1	0	0
MENTAL IMPAIRMENT (EXCL DEMENTIA AND MEMORY LOSS)										
COGNITIVE DISORDER	1	1	0	0	0	1	1	0	0	0
DISTURBANCE IN ATTENTION	12	11	1	0	0	12	11	1	0	0
MENTAL IMPAIRMENT	1	1	0	0	0	1	1	0	0	0
MOVEMENT DISORDERS (INCL PARKINSONISM)										
DYSKINESIAS AND MOVEMENT DISORDERS NEC										
BRADYKINESIA	0	0	0	0	0	0	0	0	0	0
CLUMSINESS	1	1	0	0	0	1	1	0	0	0
DYSKINESIA	3	3	0	0	0	3	3	0	0	0
EXTRAPYRAMIDAL DISORDER	1	1	0	0	0	1	1	0	0	0
PSYCHOMOTOR HYPERACTIVITY	1	0	1	0	0	1	0	1	0	0
PARALYSIS AND PARESIS (EXCL CRANIAL NERVE)										
HEMIPLEGIA	0	0	0	0	0	0	0	0	0	0
MONOPARESIS	0	0	0	0	0	0	0	0	0	0
MONOPLEGIA	1	1	0	0	0	1	1	0	0	0
PARALYSIS	1	1	0	0	0	1	1	0	0	0
PARKINSON'S DISEASE AND PARKINSONISM										
FREEZING PHENOMENON	2	2	0	0	0	2	2	0	0	0
PARKINSON'S DISEASE	0	0	0	0	0	0	0	0	0	0
TREMOR (EXCL CONGENITAL)										
TREMOR	111	100	11	0	0	105	100	5	0	0
NEUROLOGICAL DISORDERS NEC										
COMA STATES										
DIABETIC HYPERGLYCAEMIC COMA	0	0	0	0	0	0	0	0	0	0
COORDINATION AND BALANCE DISTURBANCES										
BALANCE DISORDER	16	14	2	0	0	16	14	2	0	0
COORDINATION ABNORMAL	1	1	0	0	0	1	1	0	0	0
DYSSTASIA	0	0	0	0	0	0	0	0	0	0
VESTIBULAR NYSTAGMUS	1	1	0	0	0	1	1	0	0	0
DISTURBANCES IN CONSCIOUSNESS NEC										
DEPRESSED LEVEL OF CONSCIOUSNESS	0	0	0	0	0	0	0	0	0	0
LETHARGY	204	165	39	0	0	194	165	29	0	0
LOSS OF CONSCIOUSNESS	7	6	1	0	0	7	6	1	0	0

SEDATION	0	0	0	0	0	0	0	0	0	0
SOMNOLENCE	86	72	14	0	0	84	72	12	0	0
SYNCOPE	20	14	6	0	0	16	14	2	0	0
NERVOUS SYSTEM DISORDERS NEC										
NERVOUS SYSTEM DISORDER	0	0	0	0	0	0	0	0	0	0
NEUROLOGICAL SIGNS AND SYMPTOMS NEC										
AGITATION NEONATAL	0	0	0	0	0	0	0	0	0	0
BRAIN FOG	20	15	5	0	0	19	15	4	0	0
DIZZINESS	401	319	82	0	0	382	319	63	0	0
DIZZINESS EXERTIONAL	5	4	1	0	0	5	4	1	0	0
DIZZINESS POSTURAL	61	49	12	0	0	58	49	9	0	0
HEAD DISCOMFORT	10	8	2	0	0	10	8	2	0	0
INFANT IRRITABILITY	1	0	1	0	0	1	0	1	0	0
MENINGISM	1	1	0	0	0	1	1	0	0	0
MYOCLONUS	0	0	0	0	0	0	0	0	0	0
NEUROLOGICAL SYMPTOM	1	1	0	0	0	1	1	0	0	0
PERSISTENT POSTURAL-PERCEPTUAL DIZZINESS	1	0	1	0	0	0	0	0	0	0
PRESYNCOPE	7	7	0	0	0	7	7	0	0	0
PARAESTHESIAS AND DYSAESTHESIAS										
BURNING FEET SYNDROME	0	0	0	0	0	0	0	0	0	0
BURNING SENSATION	3	3	0	0	0	3	3	0	0	0
FORMICATION	0	0	0	0	0	0	0	0	0	0
HYPERAESTHESIA	1	1	0	0	0	1	1	0	0	0
HYPOAESTHESIA	62	57	5	0	0	59	57	2	0	0
PARAESTHESIA	105	89	16	0	0	101	89	12	0	0
REVERSED HOT-COLD SENSATION	1	1	0	0	0	1	1	0	0	0
SENSORY ABNORMALITIES NEC										
AGEUSIA	19	16	3	0	0	18	16	2	0	0
ALLODYNIA	1	1	0	0	0	1	1	0	0	0
DYSGEUSIA	49	40	9	0	0	47	40	7	0	0
HYPOGEUSIA	1	1	0	0	0	1	1	0	0	0
NEURALGIA	17	14	3	0	0	15	14	1	0	0
POST HERPETIC NEURALGIA	0	0	0	0	0	0	0	0	0	0
RESTLESS ARM SYNDROME	1	1	0	0	0	1	1	0	0	0
RESTLESS LEGS SYNDROME	4	4	0	0	0	4	4	0	0	0

SENSORY LOSS	1	1	0	0	0	1	1	0	0	0
TASTE DISORDER	8	7	1	0	0	8	7	1	0	0
SPEECH AND LANGUAGE ABNORMALITIES										
DYSARTHRIA	0	0	0	0	0	0	0	0	0	0
SPEECH DISORDER DEVELOPMENTAL	0	0	0	0	0	0	0	0	0	0
NEUROLOGICAL DISORDERS OF THE EYE										
NEUROLOGIC VISUAL PROBLEMS NEC										
TUNNEL VISION	2	1	1	0	0	2	1	1	0	0
NEUROMUSCULAR DISORDERS										
MUSCLE TONE ABNORMAL										
HYPOTONIA	1	1	0	0	0	1	1	0	0	0
STIFF LEG SYNDROME	1	0	1	0	0	1	0	1	0	0
NEUROMUSCULAR DISORDERS NEC										
MUSCLE SPASTICITY	1	1	0	0	0	1	1	0	0	0
PERIPHERAL NEUROPATHIES										
ACUTE POLYNEUROPATHIES										
GUILLAIN-BARRE SYNDROME	2	2	0	0	0	2	2	0	0	0
PERIPHERAL NEUROPATHIES NEC										
AXONAL NEUROPATHY	0	0	0	0	0	0	0	0	0	0
NEUROPATHY PERIPHERAL	1	1	0	0	0	1	1	0	0	0
SEIZURES (INCL SUBTYPES)										
SEIZURES AND SEIZURE DISORDERS NEC										
EPILEPSY	0	0	0	0	0	0	0	0	0	0
SEIZURE	7	6	1	0	0	7	6	1	0	0
SLEEP DISTURBANCES (INCL SUBTYPES)										
DISTURBANCES IN SLEEP PHASE RHYTHM										
CIRCADIAN RHYTHM SLEEP DISORDER	1	1	0	0	0	1	1	0	0	0
SLEEP DISTURBANCES NEC										
SLEEP DEFICIT	1	1	0	0	0	1	1	0	0	0
SPINAL CORD AND NERVE ROOT DISORDERS										
CERVICAL SPINAL CORD AND NERVE ROOT DISORDERS										
CERVICOBRACHIAL SYNDROME	0	0	0	0	0	0	0	0	0	0
LUMBAR SPINAL CORD AND NERVE ROOT DISORDERS										
CAUDA EQUINA SYNDROME	0	0	0	0	0	0	0	0	0	0
SCIATICA	5	4	1	0	0	5	4	1	0	0
**PREGNANCY, PUERPERIUM AND PERINATAL CONDITIONS**										

ABORTIONS AND STILLBIRTH										
ABORTIONS SPONTANEOUS										
ABORTION SPONTANEOUS	7	4	3	0	0	5	4	1	0	0
STILLBIRTH AND FOETAL DEATH										
FOETAL DEATH	1	1	0	0	0	1	1	0	0	0
FOETAL COMPLICATIONS										
FOETAL COMPLICATIONS NEC										
FOETAL DISORDER	0	0	0	0	0	0	0	0	0	0
FOETAL HYPOKINESIA	0	0	0	0	0	0	0	0	0	0
FOETAL GROWTH COMPLICATIONS										
FOETAL MACROSOMIA	0	0	0	0	0	0	0	0	0	0
MATERNAL COMPLICATIONS OF PREGNANCY										
MATERNAL COMPLICATIONS OF PREGNANCY NEC										
MORNING SICKNESS	1	1	0	0	0	1	1	0	0	0
PLACENTAL, AMNIOTIC AND CAVITY DISORDERS (EXCL HAEMORRHAGES)										
PLACENTAL ABNORMALITIES (EXCL NEOPLASMS)										
PLACENTAL INFARCTION	1	1	0	0	0	1	1	0	0	0
PREGNANCY, LABOUR, DELIVERY AND POSTPARTUM CONDITIONS										
NORMAL PREGNANCY, LABOUR AND DELIVERY										
PREGNANCY	0	0	0	0	0	0	0	0	0	0
UTERINE CONTRACTIONS DURING PREGNANCY	0	0	0	0	0	0	0	0	0	0
**PRODUCT ISSUES**										
DEVICE ISSUES										
DEVICE MALFUNCTION EVENTS NEC										
OVERSENSING	1	1	0	0	0	1	1	0	0	0
**PSYCHIATRIC DISORDERS**										
ANXIETY DISORDERS AND SYMPTOMS										
ANXIETY SYMPTOMS										
AGITATION	1	1	0	0	0	1	1	0	0	0
ANXIETY	11	8	3	0	0	11	8	3	0	0
NERVOUSNESS	11	10	1	0	0	11	10	1	0	0
TENSION	5	4	1	0	0	4	4	0	0	0
PANIC ATTACKS AND DISORDERS										
PANIC ATTACK	1	1	0	0	0	1	1	0	0	0
CHANGES IN PHYSICAL ACTIVITY										
INCREASED PHYSICAL ACTIVITY LEVELS										

RESTLESSNESS	5	4	1	0	0	5	4	1	0	0
STEREOTYPIES AND AUTOMATISMS										
BRUXISM	1	1	0	0	0	1	1	0	0	0
COGNITIVE AND ATTENTION DISORDERS AND DISTURBANCES										
COGNITIVE AND ATTENTION DISORDERS AND DISTURBANCES NEC										
DAYDREAMING	1	1	0	0	0	1	1	0	0	0
MENTAL FATIGUE	15	10	5	0	0	12	10	2	0	0
COMMUNICATION DISORDERS AND DISTURBANCES										
SPEECH ARTICULATION AND RHYTHM DISTURBANCES										
DYSPHEMIA	1	0	1	0	0	0	0	0	0	0
DELIRIA (INCL CONFUSION)										
CONFUSION AND DISORIENTATION										
CONFUSIONAL STATE	21	19	2	0	0	20	19	1	0	0
DISORIENTATION	6	5	1	0	0	6	5	1	0	0
DELIRIA										
DELIRIUM	1	1	0	0	0	1	1	0	0	0
DEPRESSED MOOD DISORDERS AND DISTURBANCES										
DEPRESSIVE DISORDERS										
DEPRESSION	7	6	1	0	0	6	6	0	0	0
MOOD ALTERATIONS WITH DEPRESSIVE SYMPTOMS										
DEPRESSED MOOD	12	8	4	0	0	12	8	4	0	0
TEARFULNESS	1	1	0	0	0	1	1	0	0	0
DISSOCIATIVE DISORDERS										
DISSOCIATIVE STATES										
DISSOCIATION	1	1	0	0	0	1	1	0	0	0
DISTURBANCES IN THINKING AND PERCEPTION										
DELUSIONAL SYMPTOMS										
DELUSION	0	0	0	0	0	0	0	0	0	0
HALLUCINATIONS (EXCL SLEEP-RELATED)										
HALLUCINATION	2	2	0	0	0	2	2	0	0	0
PERCEPTION DISTURBANCES NEC										
DEREALISATION	1	1	0	0	0	1	1	0	0	0
THINKING DISTURBANCES										
BRADYPHRENIA	2	0	2	0	0	2	0	2	0	0
THOUGHT BLOCKING	1	1	0	0	0	1	1	0	0	0
MOOD DISORDERS AND DISTURBANCES NEC										

AFFECT ALTERATIONS NEC										
AFFECT LABILITY	0	0	0	0	0	0	0	0	0	0
INAPPROPRIATE AFFECT	1	1	0	0	0	1	1	0	0	0
EMOTIONAL AND MOOD DISTURBANCES NEC										
ANGER	1	1	0	0	0	1	1	0	0	0
EMOTIONAL DISORDER	0	0	0	0	0	0	0	0	0	0
EMOTIONAL DISTRESS	1	1	0	0	0	1	1	0	0	0
EUPHORIC MOOD	2	2	0	0	0	2	2	0	0	0
IRRITABILITY	8	8	0	0	0	8	8	0	0	0
MOOD ALTERED	1	0	1	0	0	1	0	1	0	0
FLUCTUATING MOOD SYMPTOMS										
MOOD SWINGS	2	1	1	0	0	1	1	0	0	0
MOOD DISORDERS NEC										
APATHY	1	0	1	0	0	1	0	1	0	0
LISTLESS	2	1	1	0	0	2	1	1	0	0
PERSONALITY DISORDERS AND DISTURBANCES IN BEHAVIOUR										
BEHAVIOUR AND SOCIALISATION DISTURBANCES										
AGGRESSION	1	1	0	0	0	1	1	0	0	0
PSYCHIATRIC AND BEHAVIOURAL SYMPTOMS NEC										
PSYCHIATRIC SYMPTOMS NEC										
PSYCHIATRIC SYMPTOM	0	0	0	0	0	0	0	0	0	0
PSYCHIATRIC DISORDERS NEC										
MENTAL DISORDERS NEC										
MENTAL DISORDER	1	0	1	0	0	1	0	1	0	0
SCHIZOPHRENIA AND OTHER PSYCHOTIC DISORDERS										
PSYCHOTIC DISORDER NEC										
PSYCHOTIC DISORDER	1	1	0	0	0	1	1	0	0	0
SEXUAL DYSFUNCTIONS, DISTURBANCES AND GENDER IDENTITY DISORDERS										
SEXUAL DESIRE DISORDERS										
LOSS OF LIBIDO	2	2	0	0	0	2	2	0	0	0
SLEEP DISORDERS AND DISTURBANCES										
DISTURBANCES IN INITIATING AND MAINTAINING SLEEP										
INITIAL INSOMNIA	0	0	0	0	0	0	0	0	0	0
INSOMNIA	87	76	11	0	0	83	76	7	0	0
DYSSOMNIAS										
BREATHING-RELATED SLEEP DISORDER	1	1	0	0	0	1	1	0	0	0

POOR QUALITY SLEEP	28	25	3	0	0	27	25	2	0	0
PARASOMNIAS										
ABNORMAL DREAMS	8	7	1	0	0	8	7	1	0	0
EXPLODING HEAD SYNDROME	0	0	0	0	0	0	0	0	0	0
NIGHTMARE	6	5	1	0	0	6	5	1	0	0
SLEEP DISORDERS NEC										
SLEEP DISORDER	11	10	1	0	0	11	10	1	0	0
SOMATIC SYMPTOM AND RELATED DISORDERS										
SOMATIC SYMPTOM DISORDERS										
HABIT COUGH	3	3	0	0	0	3	3	0	0	0
SUICIDAL AND SELF-INJURIOUS BEHAVIOURS NEC										
SUICIDAL AND SELF-INJURIOUS BEHAVIOUR										
SUICIDAL IDEATION	0	0	0	0	0	0	0	0	0	0
**RENAL AND URINARY DISORDERS**										
RENAL DISORDERS (EXCL NEPHROPATHIES)										
RENAL FAILURE AND IMPAIRMENT										
RENAL FAILURE	0	0	0	0	0	0	0	0	0	0
URINARY TRACT SIGNS AND SYMPTOMS										
BLADDER AND URETHRAL SYMPTOMS										
BLADDER PAIN	1	1	0	0	0	1	1	0	0	0
MICTURITION URGENCY	1	1	0	0	0	1	1	0	0	0
POLLAKIURIA	10	9	1	0	0	10	9	1	0	0
URINARY INCONTINENCE	1	0	1	0	0	0	0	0	0	0
URINARY ABNORMALITIES										
HAEMATURIA	0	0	0	0	0	0	0	0	0	0
URINE ABNORMALITY	0	0	0	0	0	0	0	0	0	0
URINE ODOUR ABNORMAL	2	2	0	0	0	2	2	0	0	0
URINARY TRACT SIGNS AND SYMPTOMS NEC										
POLYURIA	2	2	0	0	0	2	2	0	0	0
RENAL PAIN	7	5	2	0	0	7	5	2	0	0
**REPRODUCTIVE SYSTEM AND BREAST DISORDERS**										
BREAST DISORDERS										
BREAST DISORDERS NEC										
BREAST MASS	2	2	0	0	0	2	2	0	0	0
NIPPLE ENLARGEMENT	1	1	0	0	0	1	1	0	0	0
BREAST SIGNS AND SYMPTOMS										

BREAST DISCHARGE	0	0	0	0	0	0	0	0	0	0
BREAST PAIN	8	6	2	0	0	8	6	2	0	0
BREAST SWELLING	1	1	0	0	0	1	1	0	0	0
BREAST TENDERNESS	1	1	0	0	0	1	1	0	0	0
NIPPLE PAIN	2	2	0	0	0	2	2	0	0	0
MENOPAUSE RELATED CONDITIONS										
MENOPAUSAL EFFECTS ON THE GENITOURINARY TRACT										
POSTMENOPAUSAL HAEMORRHAGE	1	1	0	0	0	1	1	0	0	0
MENSTRUAL CYCLE AND UTERINE BLEEDING DISORDERS										
MENSTRUATION AND UTERINE BLEEDING NEC										
DYSMENORRHOEA	5	4	1	0	0	5	4	1	0	0
INTERMENSTRUAL BLEEDING	5	4	1	0	0	5	4	1	0	0
MENSTRUAL DISORDER	7	5	2	0	0	7	5	2	0	0
MENSTRUATION IRREGULAR	12	9	3	0	0	12	9	3	0	0
PREMENSTRUAL PAIN	0	0	0	0	0	0	0	0	0	0
RETROGRADE MENSTRUATION	1	1	0	0	0	1	1	0	0	0
MENSTRUATION WITH DECREASED BLEEDING										
AMENORRHOEA	0	0	0	0	0	0	0	0	0	0
HYPOMENORRHOEA	4	3	1	0	0	3	3	0	0	0
MENSTRUATION DELAYED	14	9	5	0	0	12	9	3	0	0
OLIGOMENORRHOEA	0	0	0	0	0	0	0	0	0	0
MENSTRUATION WITH INCREASED BLEEDING										
HEAVY MENSTRUAL BLEEDING	20	16	4	0	0	20	16	4	0	0
POLYMENORRHOEA	4	4	0	0	0	4	4	0	0	0
PENILE AND SCROTAL DISORDERS (EXCL INFECTIONS AND INFLAMMATIONS)										
SCROTAL DISORDERS NEC										
SCROTAL SWELLING	1	0	1	0	0	1	0	1	0	0
REPRODUCTIVE TRACT DISORDERS NEC										
REPRODUCTIVE TRACT DISORDERS NEC (EXCL NEOPLASMS)										
GENITAL LESION	1	1	0	0	0	1	1	0	0	0
REPRODUCTIVE TRACT SIGNS AND SYMPTOMS NEC										
GENITAL DISCOMFORT	1	1	0	0	0	1	1	0	0	0
GENITAL PAIN	1	1	0	0	0	1	1	0	0	0
UTERINE, PELVIC AND BROAD LIGAMENT DISORDERS										
PELVIS AND BROAD LIGAMENT DISORDERS NEC										
ADNEXA UTERI PAIN	1	1	0	0	0	1	1	0	0	0

VULVOVAGINAL DISORDERS (EXCL INFECTIONS AND INFLAMMATIONS)										
VULVOVAGINAL DISORDERS NEC										
VAGINAL HAEMORRHAGE	4	3	1	0	0	3	3	0	0	0
VULVOVAGINAL SIGNS AND SYMPTOMS										
VAGINAL DISCHARGE	1	1	0	0	0	1	1	0	0	0
**RESPIRATORY, THORACIC AND MEDIASTINAL DISORDERS**										
BRONCHIAL DISORDERS (EXCL NEOPLASMS)										
BRONCHOSPASM AND OBSTRUCTION										
ASTHMA	5	3	2	0	0	4	3	1	0	0
WHEEZING	9	8	1	0	0	8	8	0	0	0
LOWER RESPIRATORY TRACT DISORDERS (EXCL OBSTRUCTION AND INFECTION)										
PULMONARY OEDEMAS										
PULMONARY CONGESTION	0	0	0	0	0	0	0	0	0	0
PULMONARY VASCULAR DISORDERS										
PULMONARY THROMBOTIC AND EMBOLIC CONDITIONS										
PULMONARY EMBOLISM	1	1	0	0	0	1	1	0	0	0
RESPIRATORY DISORDERS NEC										
BREATHING ABNORMALITIES										
DYSPNOEA	68	56	12	0	0	66	56	10	0	0
HYPERVENTILATION	1	1	0	0	0	1	1	0	0	0
HYPOPNOEA	4	4	0	0	0	4	4	0	0	0
IRREGULAR BREATHING	1	1	0	0	0	1	1	0	0	0
MOUTH BREATHING	1	1	0	0	0	1	1	0	0	0
RESPIRATION ABNORMAL	0	0	0	0	0	0	0	0	0	0
RESPIRATORY ARREST	0	0	0	0	0	0	0	0	0	0
RESPIRATORY FATIGUE	2	2	0	0	0	2	2	0	0	0
SLEEP APNOEA SYNDROME	1	0	1	0	0	1	0	1	0	0
COUGHING AND ASSOCIATED SYMPTOMS										
COUGH	66	51	15	0	0	64	51	13	0	0
PRODUCTIVE COUGH	2	1	1	0	0	2	1	1	0	0
RESPIRATORY TRACT DISORDERS NEC										
RESPIRATORY TRACT IRRITATION	1	1	0	0	0	1	1	0	0	0
RESPIRATORY TRACT SIGNS AND SYMPTOMS										
LOWER RESPIRATORY TRACT SIGNS AND SYMPTOMS										
PULMONARY PAIN	2	2	0	0	0	2	2	0	0	0
RESPIRATORY SIGNS AND SYMPTOMS NEC										

RESPIRATORY SYMPTOM	0	0	0	0	0	0	0	0	0	0
UPPER RESPIRATORY TRACT SIGNS AND SYMPTOMS										
APHONIA	2	2	0	0	0	2	2	0	0	0
CATARRH	2	1	1	0	0	2	1	1	0	0
DRY THROAT	6	5	1	0	0	5	5	0	0	0
DYSPHONIA	5	5	0	0	0	5	5	0	0	0
INCREASED UPPER AIRWAY SECRETION	0	0	0	0	0	0	0	0	0	0
INCREASED VISCOSITY OF UPPER RESPIRATORY SE(	0	0	0	0	0	0	0	0	0	0
NASAL DISCOMFORT	1	1	0	0	0	1	1	0	0	0
OROPHARYNGEAL DISCOMFORT	2	2	0	0	0	2	2	0	0	0
OROPHARYNGEAL PAIN	131	110	21	0	0	126	110	16	0	0
PARANASAL SINUS DISCOMFORT	2	2	0	0	0	2	2	0	0	0
RHINALGIA	1	0	1	0	0	1	0	1	0	0
RHINORRHOEA	63	46	17	0	0	57	46	11	0	0
SINUS PAIN	11	9	2	0	0	11	9	2	0	0
SNEEZING	15	12	3	0	0	14	12	2	0	0
THROAT CLEARING	0	0	0	0	0	0	0	0	0	0
THROAT IRRITATION	1	1	0	0	0	1	1	0	0	0
THROAT TIGHTNESS	4	3	1	0	0	4	3	1	0	0
UPPER-AIRWAY COUGH SYNDROME	0	0	0	0	0	0	0	0	0	0
YAWNING	2	2	0	0	0	2	2	0	0	0
UPPER RESPIRATORY TRACT DISORDERS (EXCL INFECTIONS)										
NASAL CONGESTION AND INFLAMMATIONS										
NASAL CONGESTION	11	9	2	0	0	10	9	1	0	0
RHINITIS ALLERGIC	1	1	0	0	0	1	1	0	0	0
RHINITIS ATROPHIC	1	0	1	0	0	1	0	1	0	0
NASAL DISORDERS NEC										
EPISTAXIS	22	15	7	0	0	21	15	6	0	0
NASAL DRYNESS	2	1	1	0	0	1	1	0	0	0
PARANASAL SINUS DISORDERS (EXCL INFECTIONS AND NEOPLASMS)										
SINUS CONGESTION	4	2	2	0	0	4	2	2	0	0
PHARYNGEAL DISORDERS (EXCL INFECTIONS AND NEOPLASMS)										
PHARYNGEAL SWELLING	2	2	0	0	0	2	2	0	0	0
PHARYNGEAL ULCERATION	1	1	0	0	0	1	1	0	0	0
TONSILLAR ERYTHEMA	0	0	0	0	0	0	0	0	0	0

TRACHEAL DISORDERS (EXCL INFECTIONS AND NEOPLASMS)										
TRACHEAL PAIN	1	1	0	0	0	1	1	0	0	0
**SKIN AND SUBCUTANEOUS TISSUE DISORDERS**										
ANGIOEDEMA AND URTICARIA										
ANGIOEDEMAS										
ANGIOEDEMA	3	3	0	0	0	3	3	0	0	0
URTICARIAS										
COLD URTICARIA	1	0	1	0	0	1	0	1	0	0
SOLAR URTICARIA	1	1	0	0	0	1	1	0	0	0
URTICARIA	9	8	1	0	0	9	8	1	0	0
URTICARIA CHRONIC	0	0	0	0	0	0	0	0	0	0
CORNIFICATION AND DYSTROPHIC SKIN DISORDERS										
SKIN DYSTROPHIES										
HYPERTROPHIC SCAR	0	0	0	0	0	0	0	0	0	0
CUTANEOUS NEOPLASMS BENIGN										
SKIN CYSTS AND POLYPS										
DERMAL CYST	0	0	0	0	0	0	0	0	0	0
EPIDERMAL AND DERMAL CONDITIONS										
BULLOUS CONDITIONS										
BLISTER	3	2	1	0	0	3	2	1	0	0
BLOOD BLISTER	0	0	0	0	0	0	0	0	0	0
PEMPHIGOID	0	0	0	0	0	0	0	0	0	0
TOXIC EPIDERMAL NECROLYSIS	1	1	0	0	0	1	1	0	0	0
DERMAL AND EPIDERMAL CONDITIONS NEC										
DRY SKIN	2	2	0	0	0	2	2	0	0	0
PAIN OF SKIN	20	18	2	0	0	19	18	1	0	0
SENSITIVE SKIN	18	18	0	0	0	18	18	0	0	0
SKIN BURNING SENSATION	8	7	1	0	0	8	7	1	0	0
SKIN DISCOLOURATION	0	0	0	0	0	0	0	0	0	0
SKIN FRAGILITY	0	0	0	0	0	0	0	0	0	0
SKIN LESION	0	0	0	0	0	0	0	0	0	0
SKIN ODOUR ABNORMAL	2	2	0	0	0	2	2	0	0	0
SKIN REACTION	1	1	0	0	0	1	1	0	0	0
SKIN SENSITISATION	1	1	0	0	0	1	1	0	0	0
SKIN WARM	16	14	2	0	0	15	14	1	0	0
DERMATITIS AND ECZEMA										

DERMATITIS	2	2	0	0	0	2	2	0	0	0
DERMATITIS ALLERGIC	3	3	0	0	0	3	3	0	0	0
DERMATITIS ATOPIC	2	2	0	0	0	2	2	0	0	0
DERMATITIS CONTACT	1	1	0	0	0	1	1	0	0	0
ECZEMA	0	0	0	0	0	0	0	0	0	0
ECZEMA ASTEATOTIC	0	0	0	0	0	0	0	0	0	0
NEURODERMATITIS	1	1	0	0	0	1	1	0	0	0
SEBORRHOEIC DERMATITIS	2	2	0	0	0	2	2	0	0	0
SKIN IRRITATION	6	5	1	0	0	6	5	1	0	0
ERYTHEMAS										
ERYTHEMA	29	26	3	0	0	27	26	1	0	0
EXFOLIATIVE CONDITIONS										
SKIN EXFOLIATION	1	1	0	0	0	1	1	0	0	0
PAPULOSQUAMOUS CONDITIONS										
LICHEN PLANUS	1	0	1	0	0	1	0	1	0	0
PITYRIASIS ROSEA	1	1	0	0	0	1	1	0	0	0
PHOTOSENSITIVITY AND PHOTODERMATOSIS CONDITIONS										
PHOTOSENSITIVITY REACTION	5	3	2	0	0	4	3	1	0	0
PRURITUS NEC										
PRURITUS	101	88	13	0	0	97	88	9	0	0
PSORIATIC CONDITIONS										
PSORIASIS	6	5	1	0	0	6	5	1	0	0
RASHES, ERUPTIONS AND EXANTHEMS NEC										
RASH	93	78	15	0	0	88	78	10	0	0
RASH ERYTHEMATOUS	21	19	2	0	0	20	19	1	0	0
RASH MACULAR	2	0	2	0	0	2	0	2	0	0
RASH MORBILLIFORM	0	0	0	0	0	0	0	0	0	0
RASH PAPULAR	9	8	1	0	0	9	8	1	0	0
RASH PRURITIC	19	17	2	0	0	19	17	2	0	0
SKIN INJURIES AND MECHANICAL DERMATOSES										
DECUBITUS ULCER	1	1	0	0	0	1	1	0	0	0
NEEDLE TRACK MARKS	1	1	0	0	0	1	1	0	0	0
SKIN AND SUBCUTANEOUS TISSUE DISORDERS NEC										
SKIN AND SUBCUTANEOUS TISSUE ULCERATIONS										
SKIN EROSION	2	1	1	0	0	1	1	0	0	0

SKIN APPENDAGE CONDITIONS										
ACNES										
ACNE	1	1	0	0	0	1	1	0	0	0
ACNE CYSTIC	1	0	1	0	0	1	0	1	0	0
ALOPECIAS										
ALOPECIA	5	3	2	0	0	4	3	1	0	0
APOCRINE AND ECCRINE GLAND DISORDERS										
COLD SWEAT	23	22	1	0	0	22	22	0	0	0
HYPERHIDROSIS	111	100	10	1	0	107	100	7	0	0
MILIARIA	2	2	0	0	0	2	2	0	0	0
NIGHT SWEATS	53	49	4	0	0	49	49	0	0	0
HYPERTRICHOSES										
HIRSUTISM	0	0	0	0	0	0	0	0	0	0
NAIL AND NAIL BED CONDITIONS (EXCL INFECTIONS AND INFESTATIONS)										
NAIL DISCOLOURATION	0	0	0	0	0	0	0	0	0	0
ONYCHOCLASIS	0	0	0	0	0	0	0	0	0	0
PILAR DISORDERS NEC										
PILOERECTION	3	2	1	0	0	2	2	0	0	0
SKIN VASCULAR ABNORMALITIES										
PURPURA AND RELATED CONDITIONS										
PETECHIAE	2	2	0	0	0	2	2	0	0	0
PURPURA	0	0	0	0	0	0	0	0	0	0
SKIN HAEMORRHAGES										
SKIN HAEMORRHAGE	1	1	0	0	0	1	1	0	0	0
**SOCIAL CIRCUMSTANCES**										
ECONOMIC AND HOUSING ISSUES										
EMPLOYMENT ISSUES										
RETIREMENT	1	1	0	0	0	1	1	0	0	0
ENVIRONMENTAL ISSUES										
NON-OCCUPATIONAL AND UNSPECIFIED ENVIRONMENTAL PROBLEMS										
POLLUTION	1	1	0	0	0	1	1	0	0	0
**SURGICAL AND MEDICAL PROCEDURES**										
BONE AND JOINT THERAPEUTIC PROCEDURES										
JOINT THERAPEUTIC PROCEDURES										
KNEE OPERATION	1	1	0	0	0	1	1	0	0	0
BREAST THERAPEUTIC PROCEDURES										

MASTECTOMIES										
BREAST CONSERVING SURGERY	1	0	1	0	0	1	0	1	0	0
GASTROINTESTINAL THERAPEUTIC PROCEDURES										
GASTROINTESTINAL THERAPEUTIC PROCEDURES NEC										
PROPHYLAXIS OF NAUSEA AND VOMITING	1	1	0	0	0	1	1	0	0	0
HEAD AND NECK THERAPEUTIC PROCEDURES										
DENTAL AND GINGIVAL THERAPEUTIC PROCEDURES										
DENTAL CARE	2	2	0	0	0	2	2	0	0	0
NERVOUS SYSTEM, SKULL AND SPINE THERAPEUTIC PROCEDURES										
SKULL AND BRAIN THERAPEUTIC PROCEDURES										
BRAIN TUMOUR OPERATION	1	1	0	0	0	1	1	0	0	0
OBSTETRIC AND GYNAECOLOGICAL THERAPEUTIC PROCEDURES										
FERTILITY AND FERTILISATION INTERVENTIONS FEMALE										
ENDOMETRIAL SCRATCHING	0	0	0	0	0	0	0	0	0	0
RESPIRATORY TRACT THERAPEUTIC PROCEDURES										
RESPIRATORY TRACT THERAPEUTIC PROCEDURES NEC										
OXYGEN THERAPY	0	0	0	0	0	0	0	0	0	0
THERAPEUTIC PROCEDURES AND SUPPORTIVE CARE NEC										
ANAESTHESIA AND ALLIED PROCEDURES										
NERVE BLOCK	2	2	0	0	0	2	2	0	0	0
DIETARY AND NUTRITIONAL THERAPIES										
NOTHING BY MOUTH ORDER	0	0	0	0	0	0	0	0	0	0
IMMUNISATIONS										
COVID-19 IMMUNISATION	1	0	1	0	0	0	0	0	0	0
THERAPEUTIC PROCEDURES NEC										
INJECTION	2	2	0	0	0	2	2	0	0	0
LOCALISED ALTERNATING HOT AND COLD THERAPY	2	2	0	0	0	2	2	0	0	0
MASS EXCISION	1	0	1	0	0	1	0	1	0	0
REINFUSION	0	0	0	0	0	0	0	0	0	0
**VASCULAR DISORDERS**										
ARTERIOSCLEROSIS, STENOSIS, VASCULAR INSUFFICIENCY AND NECROSIS										
NON-SITE SPECIFIC NECROSIS AND VASCULAR INSUFFICIENCY NEC										
VASOSPASM	1	1	0	0	0	1	1	0	0	0
PERIPHERAL VASOCONSTRICTION, NECROSIS AND VASCULAR INSUFFICIENCY										
PERIPHERAL COLDNESS	47	45	2	0	0	46	45	1	0	0

RAYNAUD'S PHENOMENON	1	1	0	0	0	1	1	0	0	0
DECREASED AND NONSPECIFIC BLOOD PRESSURE DISORDERS AND SHOCK										
BLOOD PRESSURE DISORDERS NEC										
BLOOD PRESSURE FLUCTUATION	1	1	0	0	0	1	1	0	0	0
VASCULAR HYPOTENSIVE DISORDERS										
CAPILLARY LEAK SYNDROME	1	1	0	0	0	1	1	0	0	0
HYPOTENSION	3	3	0	0	0	3	3	0	0	0
ORTHOSTATIC HYPOTENSION	1	1	0	0	0	1	1	0	0	0
EMBOLISM AND THROMBOSIS										
NON-SITE SPECIFIC EMBOLISM AND THROMBOSIS										
EMBOLISM	0	0	0	0	0	0	0	0	0	0
THROMBOSIS	7	6	1	0	0	7	6	1	0	0
VENOUS THROMBOSIS	1	0	1	0	0	0	0	0	0	0
PERIPHERAL EMBOLISM AND THROMBOSIS										
BLUE TOE SYNDROME	2	2	0	0	0	2	2	0	0	0
DEEP VEIN THROMBOSIS	4	3	1	0	0	3	3	0	0	0
SUPERFICIAL VEIN THROMBOSIS	0	0	0	0	0	0	0	0	0	0
LYMPHATIC VESSEL DISORDERS										
LYMPHOEDEMAS										
LYMPHOEDEMA	0	0	0	0	0	0	0	0	0	0
VASCULAR DISORDERS NEC										
NON-SITE SPECIFIC VASCULAR DISORDERS NEC										
VASCULAR PAIN	0	0	0	0	0	0	0	0	0	0
VEIN DISCOLOURATION	0	0	0	0	0	0	0	0	0	0
VEIN RUPTURE	1	0	1	0	0	1	0	1	0	0
PERIPHERAL VASCULAR DISORDERS NEC										
FLUSHING	22	19	3	0	0	21	19	2	0	0
HOT FLUSH	78	67	11	0	0	75	67	8	0	0
SITE SPECIFIC VASCULAR DISORDERS NEC										
PALLOR	4	3	1	0	0	3	3	0	0	0
VASCULAR HAEMORRHAGIC DISORDERS										
HAEMORRHAGES NEC										
HAEMATOMA	1	1	0	0	0	1	1	0	0	0
HAEMORRHAGE	2	1	1	0	0	1	1	0	0	0
VASCULAR HYPERTENSIVE DISORDERS										
ACCELERATED AND MALIGNANT HYPERTENSION										

HYPERTENSIVE URGENCY	0	0	0	0	0	0	0	0	0	0
VASCULAR HYPERTENSIVE DISORDERS NEC										
HYPERTENSION	7	3	4	0	0	6	3	3	0	0
SYSTOLIC HYPERTENSION	1	1	0	0	0	1	1	0	0	0
VASCULAR INFECTIONS AND INFLAMMATIONS										
ARTERIAL INFECTIONS AND INFLAMMATIONS										
GIANT CELL ARTERITIS	1	1	0	0	0	1	1	0	0	0
PHLEBITIS NEC										
PHLEBITIS	2	1	1	0	0	2	1	1	0	0
VASCULITIDES NEC										
VASCULITIS	0	0	0	0	0	0	0	0	0	0
VENOUS VARICES										
VARICOSE VEINS NEC										
SPIDER VEIN	0	0	0	0	0	0	0	0	0	0
VARICOSE VEIN	2	2	0	0	0	2	2	0	0	0
**TOTAL ADR EVENTS**	***23198***	***19559***	***3613***	***25***	***1***	***22004***	***19559***	***2433***	***12***	***0***

SUPPLEMENTARY TABLE 18. Moderna COVID-19 vaccine: ADR listing for events reported in the YCVM in (a) those reporting any vaccination dose and (b) in those who had reported a 1st dose vaccination and any subsequent

	Individuals with any vaccination dose: ADR Counts					Individuals reporting a 1st dose: ADR Counts				
**MEDDRA REACTION TERM (SOC, *HLGT*, *HLT*, PT)**	All doses	1st dose	2nd dose	3rd dose	Other doses	All doses	1st dose	2nd dose	3rd dose	Other doses
(freetext)	0	0	0	0	0	0	0	0	0	0
**BLOOD AND LYMPHATIC SYSTEM DISORDERS**										
COAGULOPATHIES AND BLEEDING DIATHESES (EXCL THROMBOCYTOPENIC)										
BLEEDING TENDENCIES										
INCREASED TENDENCY TO BRUISE	0	0	0	0	0	0	0	0	0	0
HAEMOGLOBINOPATHIES										
SICKLE CELL TRAIT AND DISORDERS										
SICKLE CELL ANAEMIA WITH CRISIS	0	0	0	0	0	0	0	0	0	0
PLATELET DISORDERS										
THROMBOCYTOPENIAS										
IMMUNE THROMBOCYTOPENIA	0	0	0	0	0	0	0	0	0	0
SPLEEN, LYMPHATIC AND RETICULOENDOTHELIAL SYSTEM DISORDERS										
LYMPHATIC SYSTEM DISORDERS NEC										
LYMPH NODE PAIN	3	2	0	1	0	3	2	0	1	0
LYMPHADENITIS	2	1	0	1	0	2	1	0	1	0
LYMPHADENOPATHY	19	7	2	10	0	17	7	2	8	0
**CARDIAC DISORDERS**										
CARDIAC ARRHYTHMIAS										
RATE AND RHYTHM DISORDERS NEC										
ARRHYTHMIA	0	0	0	0	0	0	0	0	0	0
CARDIAC FLUTTER	1	0	0	1	0	1	0	0	1	0
EXTRASYSTOLES	0	0	0	0	0	0	0	0	0	0
TACHYCARDIA	0	0	0	0	0	0	0	0	0	0
SUPRAVENTRICULAR ARRHYTHMIAS										
ATRIAL FIBRILLATION	1	0	0	1	0	0	0	0	0	0
SUPRAVENTRICULAR TACHYCARDIA	0	0	0	0	0	0	0	0	0	0

CARDIAC DISORDERS, SIGNS AND SYMPTOMS NEC										
CARDIAC DISORDERS NEC										
CARDIOVASCULAR DISORDER	0	0	0	0	0	0	0	0	0	0
CARDIAC SIGNS AND SYMPTOMS NEC										
PALPITATIONS	10	3	1	6	0	9	3	1	5	0
CORONARY ARTERY DISORDERS										
ISCHAEMIC CORONARY ARTERY DISORDERS										
ANGINA PECTORIS	0	0	0	0	0	0	0	0	0	0
MYOCARDIAL INFARCTION	0	0	0	0	0	0	0	0	0	0
HEART FAILURES										
HEART FAILURES NEC										
CARDIAC FAILURE	0	0	0	0	0	0	0	0	0	0
MYOCARDIAL DISORDERS										
NONINFECTIOUS MYOCARDITIS										
MYOCARDITIS	1	0	0	0	1	1	0	0	0	1
PERICARDIAL DISORDERS										
NONINFECTIOUS PERICARDITIS										
PERICARDITIS	0	0	0	0	0	0	0	0	0	0
**CONGENITAL, FAMILIAL AND GENETIC DISORDERS**										
CARDIAC AND VASCULAR DISORDERS CONGENITAL										
CARDIAC DISORDERS CONGENITAL NEC										
HEART DISEASE CONGENITAL	0	0	0	0	0	0	0	0	0	0
METABOLIC AND NUTRITIONAL DISORDERS CONGENITAL										
INBORN ERRORS OF AMINO ACID METABOLISM										
HYPERGLYCINAEMIA	0	0	0	0	0	0	0	0	0	0
INBORN ERRORS OF STEROID SYNTHESIS										
11-BETA-HYDROXYLASE DEFICIENCY	0	0	0	0	0	0	0	0	0	0
NEUROLOGICAL DISORDERS CONGENITAL										
PERIPHERAL NERVOUS SYSTEM DISORDERS CONGENITAL NEC										
PAROXYSMAL EXTREME PAIN DISORDER	0	0	0	0	0	0	0	0	0	0
**EAR AND LABYRINTH DISORDERS**										

AURAL DISORDERS NEC										
EAR DISORDERS NEC										
EAR DISCOMFORT	0	0	0	0	0	0	0	0	0	0
EAR DISORDER	0	0	0	0	0	0	0	0	0	0
EAR PAIN	2	2	0	0	0	2	2	0	0	0
EAR SWELLING	0	0	0	0	0	0	0	0	0	0
EXTERNAL EAR DISORDERS (EXCL CONGENITAL)										
EXTERNAL EAR DISORDERS NEC										
EXCESSIVE CERUMEN PRODUCTION	0	0	0	0	0	0	0	0	0	0
HEARING DISORDERS										
HEARING LOSSES										
DEAFNESS	1	1	0	0	0	1	1	0	0	0
DEAFNESS NEUROSENSORY	0	0	0	0	0	0	0	0	0	0
DEAFNESS PERMANENT	0	0	0	0	0	0	0	0	0	0
DEAFNESS UNILATERAL	0	0	0	0	0	0	0	0	0	0
HYPOACUSIS	0	0	0	0	0	0	0	0	0	0
SUDDEN HEARING LOSS	0	0	0	0	0	0	0	0	0	0
HYPERACUSIA										
HYPERACUSIS	0	0	0	0	0	0	0	0	0	0
INNER EAR AND VIIITH CRANIAL NERVE DISORDERS										
INNER EAR SIGNS AND SYMPTOMS										
MOTION SICKNESS	0	0	0	0	0	0	0	0	0	0
TINNITUS	3	0	1	2	0	3	0	1	2	0
VERTIGO	2	0	0	1	1	1	0	0	0	1
VERTIGO LABYRINTHINE	0	0	0	0	0	0	0	0	0	0
VERTIGO POSITIONAL	1	1	0	0	0	1	1	0	0	0
**ENDOCRINE DISORDERS**										
THYROID GLAND DISORDERS										
THYROID HYPERFUNCTION DISORDERS										
HYPERTHYROIDISM	0	0	0	0	0	0	0	0	0	0
**EYE DISORDERS**										
EYE DISORDERS NEC										
LACRIMATION DISORDERS										
DRY EYE	0	0	0	0	0	0	0	0	0	0
LACRIMATION INCREASED	0	0	0	0	0	0	0	0	0	0
OCULAR DISORDERS NEC										

EYE OEDEMA	0	0	0	0	0	0	0	0	0	0
EYE PAIN	3	1	1	1	0	3	1	1	1	0
EYE SWELLING	2	0	0	2	0	0	0	0	0	0
EYE ULCER	0	0	0	0	0	0	0	0	0	0
EYELID PAIN	0	0	0	0	0	0	0	0	0	0
OCULAR DISCOMFORT	0	0	0	0	0	0	0	0	0	0
PERIORBITAL DISCOMFORT	0	0	0	0	0	0	0	0	0	0
PERIORBITAL SWELLING	1	0	0	1	0	1	0	0	1	0
OCULAR HAEMORRHAGES AND VASCULAR DISORDERS NEC										
CONJUNCTIVAL AND CORNEAL BLEEDING AND VASCULAR DISORDERS										
CONJUNCTIVAL HAEMORRHAGE	0	0	0	0	0	0	0	0	0	0
LID BLEEDING AND VASCULAR DISORDERS										
EYELID BLEEDING	0	0	0	0	0	0	0	0	0	0
OCULAR INFECTIONS, IRRITATIONS AND INFLAMMATIONS										
LID, LASH AND LACRIMAL INFECTIONS, IRRITATIONS AND INFLAMMATIONS										
BLEPHARITIS	0	0	0	0	0	0	0	0	0	0
ERYTHEMA OF EYELID	0	0	0	0	0	0	0	0	0	0
EYELID IRRITATION	0	0	0	0	0	0	0	0	0	0
OCULAR INFECTIONS, INFLAMMATIONS AND ASSOCIATED MANIFESTATIONS										
EYE DISCHARGE	0	0	0	0	0	0	0	0	0	0
EYE IRRITATION	1	1	0	0	0	1	1	0	0	0
EYE PRURITUS	0	0	0	0	0	0	0	0	0	0
LIMBAL SWELLING	1	0	0	1	0	1	0	0	1	0
OCULAR HYPERAEMIA	3	0	0	3	0	3	0	0	3	0
OCULAR NEUROMUSCULAR DISORDERS										
EYELID MOVEMENT DISORDERS										
BLEPHAROSPASM	0	0	0	0	0	0	0	0	0	0
OCULAR SENSORY SYMPTOMS NEC										
OCULAR SENSATION DISORDERS										
ABNORMAL SENSATION IN EYE	0	0	0	0	0	0	0	0	0	0
ASTHENOPIA	2	2	0	0	0	2	2	0	0	0
FOREIGN BODY SENSATION IN EYES	0	0	0	0	0	0	0	0	0	0
PHOTOPHOBIA	0	0	0	0	0	0	0	0	0	0

OCULAR STRUCTURAL CHANGE, DEPOSIT AND DEGENERATION NEC										
CHOROID AND VITREOUS STRUCTURAL CHANGE, DEPOSIT AND DEGENERATION										
VITREOUS DETACHMENT	0	0	0	0	0	0	0	0	0	0
VITREOUS FLOATERS	0	0	0	0	0	0	0	0	0	0
RETINA, CHOROID AND VITREOUS HAEMORRHAGES AND VASCULAR DISORDERS										
RETINAL BLEEDING AND VASCULAR DISORDERS (EXCL RETINOPATHY)										
RETINAL VEIN OCCLUSION	0	0	0	0	0	0	0	0	0	0
RETINOPATHIES NEC										
RETINAL EXUDATES	0	0	0	0	0	0	0	0	0	0
VISION DISORDERS										
VISUAL DISORDERS NEC										
DIPLOPIA	0	0	0	0	0	0	0	0	0	0
HALO VISION	0	0	0	0	0	0	0	0	0	0
METAMORPHOPSIA	0	0	0	0	0	0	0	0	0	0
PHOTOPSIA	0	0	0	0	0	0	0	0	0	0
VISION BLURRED	2	1	0	1	0	1	1	0	0	0
VISUAL IMPAIRMENT AND BLINDNESS (EXCL COLOUR BLINDNESS)										
BLINDNESS	0	0	0	0	0	0	0	0	0	0
BLINDNESS TRANSIENT	0	0	0	0	0	0	0	0	0	0
SUDDEN VISUAL LOSS	0	0	0	0	0	0	0	0	0	0
VISUAL IMPAIRMENT	1	1	0	0	0	1	1	0	0	0
**GASTROINTESTINAL DISORDERS**										
ANAL AND RECTAL CONDITIONS NEC										
ANAL AND RECTAL SIGNS AND SYMPTOMS										
ANAL PARAESTHESIA	0	0	0	0	0	0	0	0	0	0
BENIGN NEOPLASMS GASTROINTESTINAL										
BENIGN ORAL CAVITY NEOPLASMS										
MOUTH CYST	0	0	0	0	0	0	0	0	0	0
DENTAL AND GINGIVAL CONDITIONS										
DENTAL DISORDERS NEC										
TEETHING	0	0	0	0	0	0	0	0	0	0

DENTAL PAIN AND SENSATION DISORDERS										
DENTAL PARAESTHESIA	0	0	0	0	0	0	0	0	0	0
TOOTHACHE	1	0	1	0	0	0	0	0	0	0
GINGIVAL DISORDERS, SIGNS AND SYMPTOMS NEC										
GINGIVAL BLISTER	0	0	0	0	0	0	0	0	0	0
GINGIVAL PAIN	1	1	0	0	0	1	1	0	0	0
GINGIVAL SWELLING	0	0	0	0	0	0	0	0	0	0
GASTROINTESTINAL CONDITIONS NEC										
GASTROINTESTINAL MUCOSAL DYSTROPHIES AND SECRETION DISORDERS										
BARRETT'S OESOPHAGUS	0	0	0	0	0	0	0	0	0	0
GASTROINTESTINAL HAEMORRHAGES NEC										
INTESTINAL HAEMORRHAGES										
RECTAL HAEMORRHAGE	0	0	0	0	0	0	0	0	0	0
NON-SITE SPECIFIC GASTROINTESTINAL HAEMORRHAGES										
HAEMATEMESIS	0	0	0	0	0	0	0	0	0	0
GASTROINTESTINAL INFLAMMATORY CONDITIONS										
COLITIS (EXCL INFECTIVE)										
COLITIS	0	0	0	0	0	0	0	0	0	0
COLITIS MICROSCOPIC	0	0	0	0	0	0	0	0	0	0
COLITIS ULCERATIVE	0	0	0	0	0	0	0	0	0	0
GASTRITIS (EXCL INFECTIVE)										
GASTRITIS	0	0	0	0	0	0	0	0	0	0
REFLUX GASTRITIS	0	0	0	0	0	0	0	0	0	0
GASTROINTESTINAL INFLAMMATORY DISORDERS NEC										
GASTROINTESTINAL TRACT IRRITATION	0	0	0	0	0	0	0	0	0	0
GASTROINTESTINAL MOTILITY AND DEFAECATION CONDITIONS										
DIARRHOEA (EXCL INFECTIVE)										
DIARRHOEA	11	2	0	4	5	9	2	0	3	4
GASTROINTESTINAL ATONIC AND HYPOMOTILITY DISORDERS NEC										
CONSTIPATION	0	0	0	0	0	0	0	0	0	0
GASTROOESOPHAGEAL REFLUX DISEASE	0	0	0	0	0	0	0	0	0	0
GASTROINTESTINAL DYSKINETIC DISORDERS										
CHANGE OF BOWEL HABIT	0	0	0	0	0	0	0	0	0	0
GASTROINTESTINAL SPASTIC AND HYPERMOTILITY DISORDERS										
IRRITABLE BOWEL SYNDROME	0	0	0	0	0	0	0	0	0	0
GASTROINTESTINAL SIGNS AND SYMPTOMS										

DYSPEPTIC SIGNS AND SYMPTOMS										
DYSPEPSIA	2	0	0	1	1	2	0	0	1	1
ERUCTATION	0	0	0	0	0	0	0	0	0	0
FAECAL ABNORMALITIES NEC										
FAECALOMA	0	0	0	0	0	0	0	0	0	0
FAECES DISCOLOURED	0	0	0	0	0	0	0	0	0	0
FAECES SOFT	1	0	0	1	0	1	0	0	1	0
FLATULENCE, BLOATING AND DISTENSION										
ABDOMINAL DISTENSION	1	0	0	1	0	1	0	0	1	0
FLATULENCE	0	0	0	0	0	0	0	0	0	0
GASTROINTESTINAL AND ABDOMINAL PAINS (EXCL ORAL AND THROAT)										
ABDOMINAL PAIN	3	0	0	2	1	2	0	0	2	0
ABDOMINAL PAIN LOWER	0	0	0	0	0	0	0	0	0	0
ABDOMINAL PAIN UPPER	5	1	2	2	0	3	1	1	1	0
GASTROINTESTINAL PAIN	1	0	0	1	0	1	0	0	1	0
GASTROINTESTINAL SIGNS AND SYMPTOMS NEC										
ABDOMINAL DISCOMFORT	0	0	0	0	0	0	0	0	0	0
ABDOMINAL SYMPTOM	0	0	0	0	0	0	0	0	0	0
ACUTE ABDOMEN	0	0	0	0	0	0	0	0	0	0
ANAL INCONTINENCE	0	0	0	0	0	0	0	0	0	0
BREATH ODOUR	0	0	0	0	0	0	0	0	0	0
DYSPHAGIA	0	0	0	0	0	0	0	0	0	0
ODYNOPHAGIA	0	0	0	0	0	0	0	0	0	0
NAUSEA AND VOMITING SYMPTOMS										
NAUSEA	87	23	9	45	10	78	23	6	40	9
RETCHING	0	0	0	0	0	0	0	0	0	0
VOMITING	16	1	5	9	1	12	1	4	6	1
VOMITING PROJECTILE	0	0	0	0	0	0	0	0	0	0
MALABSORPTION CONDITIONS										
MALABSORPTION SYNDROMES										
COELIAC DISEASE	0	0	0	0	0	0	0	0	0	0
ORAL SOFT TISSUE CONDITIONS										
ORAL SOFT TISSUE DISORDERS NEC										
CHEILITIS	0	0	0	0	0	0	0	0	0	0
LIP BLISTER	0	0	0	0	0	0	0	0	0	0

ORAL LICHEN PLANUS	0	0	0	0	0	0	0	0	0	0
ORAL SOFT TISSUE INFECTIONS										
ANGULAR CHEILITIS	0	0	0	0	0	0	0	0	0	0
ORAL SOFT TISSUE SIGNS AND SYMPTOMS										
HYPOAESTHESIA ORAL	0	0	0	0	0	0	0	0	0	0
LIP PAIN	0	0	0	0	0	0	0	0	0	0
ORAL DISCOMFORT	0	0	0	0	0	0	0	0	0	0
ORAL MUCOSAL ROUGHENING	0	0	0	0	0	0	0	0	0	0
ORAL PAIN	0	0	0	0	0	0	0	0	0	0
PARAESTHESIA ORAL	0	0	0	0	0	0	0	0	0	0
LIP SWELLING	1	0	1	0	0	1	0	1	0	0
MOUTH SWELLING	0	0	0	0	0	0	0	0	0	0
STOMATITIS AND ULCERATION										
APHTHOUS ULCER	0	0	0	0	0	0	0	0	0	0
LIP ULCERATION	0	0	0	0	0	0	0	0	0	0
MOUTH ULCERATION	1	0	0	1	0	1	0	0	1	0
STOMATITIS	0	0	0	0	0	0	0	0	0	0
SALIVARY GLAND CONDITIONS										
ORAL DRYNESS AND SALIVA ALTERED										
DRY MOUTH	3	0	0	0	3	2	0	0	0	2
LIP DRY	1	0	0	1	0	1	0	0	1	0
SALIVARY HYPOSECRETION	0	0	0	0	0	0	0	0	0	0
TONGUE CONDITIONS										
TONGUE SIGNS AND SYMPTOMS										
GLOSSODYNIA	1	0	0	1	0	0	0	0	0	0
SWOLLEN TONGUE	1	0	0	1	0	1	0	0	1	0
TONGUE COATED	0	0	0	0	0	0	0	0	0	0
TONGUE DISCOMFORT	0	0	0	0	0	0	0	0	0	0
TONGUE OEDEMA	0	0	0	0	0	0	0	0	0	0
TONGUE SPASM	1	0	0	1	0	1	0	0	1	0
**GENERAL DISORDERS AND ADMINISTRATION SITE CONDITIONS**										
ADMINISTRATION SITE REACTIONS										
ADMINISTRATION SITE REACTIONS NEC										
ADMINISTRATION SITE BRUISE	0	0	0	0	0	0	0	0	0	0
ADMINISTRATION SITE PAIN	0	0	0	0	0	0	0	0	0	0

PUNCTURE SITE BRUISE	1	0	0	1	0	1	0	0	1	0
PUNCTURE SITE PAIN	0	0	0	0	0	0	0	0	0	0
APPLICATION SITE REACTIONS										
APPLICATION SITE BRUISE	1	0	0	0	1	1	0	0	0	1
APPLICATION SITE ERYTHEMA	0	0	0	0	0	0	0	0	0	0
APPLICATION SITE PAIN	2	0	0	2	0	2	0	0	2	0
IMPLANT AND CATHETER SITE REACTIONS										
IMPLANT SITE PAIN	0	0	0	0	0	0	0	0	0	0
IMPLANT SITE WARMTH	0	0	0	0	0	0	0	0	0	0
INFUSION SITE REACTIONS										
INFUSION SITE PAIN	0	0	0	0	0	0	0	0	0	0
INFUSION SITE SCAB	0	0	0	0	0	0	0	0	0	0
INFUSION SITE WARMTH	0	0	0	0	0	0	0	0	0	0
INJECTION SITE REACTIONS										
INJECTION SITE BRUISING	2	0	0	2	0	2	0	0	2	0
INJECTION SITE DISCOMFORT	0	0	0	0	0	0	0	0	0	0
INJECTION SITE ERYTHEMA	5	2	1	1	1	4	2	1	1	0
INJECTION SITE HYPERSENSITIVITY	0	0	0	0	0	0	0	0	0	0
INJECTION SITE INFLAMMATION	0	0	0	0	0	0	0	0	0	0
INJECTION SITE INJURY	0	0	0	0	0	0	0	0	0	0
INJECTION SITE IRRITATION	0	0	0	0	0	0	0	0	0	0
INJECTION SITE JOINT PAIN	1	0	0	0	1	0	0	0	0	0
INJECTION SITE MASS	4	2	1	0	1	4	2	1	0	1
INJECTION SITE NODULE	0	0	0	0	0	0	0	0	0	0
INJECTION SITE OEDEMA	0	0	0	0	0	0	0	0	0	0
INJECTION SITE PAIN	49	15	4	23	7	46	15	2	22	7
INJECTION SITE PAPULE	0	0	0	0	0	0	0	0	0	0
INJECTION SITE PARAESTHESIA	0	0	0	0	0	0	0	0	0	0
INJECTION SITE PRURITUS	3	2	0	1	0	3	2	0	1	0
INJECTION SITE RASH	3	2	1	0	0	3	2	1	0	0
INJECTION SITE REACTION	0	0	0	0	0	0	0	0	0	0
INJECTION SITE SCAB	0	0	0	0	0	0	0	0	0	0
INJECTION SITE SWELLING	2	0	0	2	0	2	0	0	2	0
INJECTION SITE URTICARIA	3	0	0	0	3	3	0	0	0	3
INJECTION SITE WARMTH	4	2	0	1	1	4	2	0	1	1

INSTILLATION SITE REACTIONS										
INSTILLATION SITE PRURITUS	0	0	0	0	0	0	0	0	0	0
INSTILLATION SITE WARMTH	0	0	0	0	0	0	0	0	0	0
VACCINATION SITE REACTIONS										
SHOULDER INJURY RELATED TO VACCINE ADMINISTRATION	0	0	0	0	0	0	0	0	0	0
VACCINATION SITE BRUISING	0	0	0	0	0	0	0	0	0	0
VACCINATION SITE DISCOMFORT	0	0	0	0	0	0	0	0	0	0
VACCINATION SITE ERYTHEMA	0	0	0	0	0	0	0	0	0	0
VACCINATION SITE JOINT ERYTHEMA	0	0	0	0	0	0	0	0	0	0
VACCINATION SITE JOINT PAIN	1	0	0	1	0	1	0	0	1	0
VACCINATION SITE MASS	1	1	0	0	0	1	1	0	0	0
VACCINATION SITE PAIN	5	0	0	4	1	5	0	0	4	1
VACCINATION SITE RASH	0	0	0	0	0	0	0	0	0	0
VACCINATION SITE SWELLING	0	0	0	0	0	0	0	0	0	0
VACCINATION SITE WARMTH	0	0	0	0	0	0	0	0	0	0
BODY TEMPERATURE CONDITIONS										
BODY TEMPERATURE ALTERED										
HYPERTHERMIA	0	0	0	0	0	0	0	0	0	0
HYPOTHERMIA	0	0	0	0	0	0	0	0	0	0
FEBRILE DISORDERS										
PYREXIA	176	33	48	75	20	155	33	39	64	19
FATAL OUTCOMES										
DEATH AND SUDDEN DEATH										
DEATH	0	0	0	0	0	0	0	0	0	0
GENERAL SYSTEM DISORDERS NEC										
ADVERSE EFFECT ABSENT										
NO ADVERSE EVENT	0	0	0	0	0	0	0	0	0	0
ASTHENIC CONDITIONS										
ASTHENIA	10	5	1	2	2	7	5	0	1	1
CHRONIC FATIGUE SYNDROME	0	0	0	0	0	0	0	0	0	0
DECREASED ACTIVITY	0	0	0	0	0	0	0	0	0	0
FATIGUE	278	63	46	121	48	245	63	37	106	39
MALAISE	33	4	3	18	8	27	4	3	15	5
SLUGGISHNESS	1	0	0	0	1	1	0	0	0	1
FEELINGS AND SENSATIONS NEC										

CHILLS	104	10	22	62	10	92	10	17	57	8
FEELING ABNORMAL	6	1	1	1	3	6	1	1	1	3
FEELING COLD	19	0	3	11	5	12	0	0	8	4
FEELING HOT	5	1	1	2	1	4	1	1	2	0
FEELING JITTERY	0	0	0	0	0	0	0	0	0	0
FEELING OF BODY TEMPERATURE CHANGE	4	0	2	2	0	4	0	2	2	0
FEELING OF RELAXATION	0	0	0	0	0	0	0	0	0	0
HANGOVER	0	0	0	0	0	0	0	0	0	0
HUNGER	1	0	1	0	0	1	0	1	0	0
SENSATION OF BLOOD FLOW	0	0	0	0	0	0	0	0	0	0
THIRST	0	0	0	0	0	0	0	0	0	0
GAIT DISTURBANCES										
GAIT DISTURBANCE	0	0	0	0	0	0	0	0	0	0
GAIT INABILITY	0	0	0	0	0	0	0	0	0	0
GENERAL SIGNS AND SYMPTOMS NEC										
CRYING	0	0	0	0	0	0	0	0	0	0
ENERGY INCREASED	0	0	0	0	0	0	0	0	0	0
EXERCISE TOLERANCE DECREASED	0	0	0	0	0	0	0	0	0	0
GENERAL SYMPTOM	0	0	0	0	0	0	0	0	0	0
ILLNESS	14	5	4	5	0	13	5	3	5	0
INFLUENZA LIKE ILLNESS	45	1	2	35	7	33	1	1	25	6
LOCAL REACTION	0	0	0	0	0	0	0	0	0	0
PERIPHERAL SWELLING	30	5	3	18	4	26	5	3	15	3
SWELLING	6	3	0	3	0	5	3	0	2	0
SWELLING FACE	0	0	0	0	0	0	0	0	0	0
TISSUE IRRITATION	0	0	0	0	0	0	0	0	0	0
INFLAMMATIONS										
INFLAMMATION	0	0	0	0	0	0	0	0	0	0
SYSTEMIC INFLAMMATORY RESPONSE SYNDROME	0	0	0	0	0	0	0	0	0	0
OEDEMA NEC										
OEDEMA	0	0	0	0	0	0	0	0	0	0
OEDEMA PERIPHERAL	0	0	0	0	0	0	0	0	0	0
PAIN AND DISCOMFORT NEC										
AXILLARY PAIN	14	7	1	6	0	14	7	1	6	0

CHEST DISCOMFORT	4	0	0	4	0	4	0	0	4	0
CHEST PAIN	7	1	0	4	2	5	1	0	3	1
DISCOMFORT	3	0	0	2	1	1	0	0	1	0
FACIAL PAIN	0	0	0	0	0	0	0	0	0	0
HERNIA PAIN	0	0	0	0	0	0	0	0	0	0
INFLAMMATORY PAIN	0	0	0	0	0	0	0	0	0	0
NON-CARDIAC CHEST PAIN	0	0	0	0	0	0	0	0	0	0
PAIN	75	20	18	28	9	63	20	15	21	7
TENDERNESS	17	4	0	11	2	15	4	0	9	2
THERAPEUTIC AND NONTHERAPEUTIC EFFECTS (EXCL TOXICITY)										
THERAPEUTIC AND NONTHERAPEUTIC RESPONSES										
ADVERSE DRUG REACTION	0	0	0	0	0	0	0	0	0	0
ADVERSE EVENT	0	0	0	0	0	0	0	0	0	0
ADVERSE REACTION	0	0	0	0	0	0	0	0	0	0
IMMEDIATE POST-INJECTION REACTION	0	0	0	0	0	0	0	0	0	0
**HEPATOBILIARY DISORDERS**										
HEPATIC AND HEPATOBILIARY DISORDERS										
HEPATOBILIARY SIGNS AND SYMPTOMS										
HEPATIC PAIN	0	0	0	0	0	0	0	0	0	0
HEPATOCELLULAR DAMAGE AND HEPATITIS NEC										
LIVER INJURY	0	0	0	0	0	0	0	0	0	0
**IMMUNE SYSTEM DISORDERS**										
ALLERGIC CONDITIONS										
ALLERGIC CONDITIONS NEC										
HYPERSENSITIVITY	0	0	0	0	0	0	0	0	0	0
MULTIPLE ALLERGIES	0	0	0	0	0	0	0	0	0	0
ALLERGIES TO FOODS, FOOD ADDITIVES, DRUGS AND OTHER CHEMICALS										
ALLERGY TO CHEMICALS	0	0	0	0	0	0	0	0	0	0
ANAPHYLACTIC AND ANAPHYLACTOID RESPONSES										
ANAPHYLACTIC REACTION	0	0	0	0	0	0	0	0	0	0
ATOPIC DISORDERS										
SEASONAL ALLERGY	0	0	0	0	0	0	0	0	0	0
IMMUNE DISORDERS NEC										
IMMUNE AND ASSOCIATED CONDITIONS NEC										

BACILLE CALMETTE-GUERIN SCAR REACTIVATION	0	0	0	0	0	0	0	0	0	0
SENSITISATION	0	0	0	0	0	0	0	0	0	0
**INFECTIONS AND INFESTATIONS**										
BACTERIAL INFECTIOUS DISORDERS										
BACTERIAL INFECTIONS NEC										
CELLULITIS	2	1	1	0	0	1	1	0	0	0
PERIORBITAL CELLULITIS	0	0	0	0	0	0	0	0	0	0
HELICOBACTER INFECTIONS										
HELICOBACTER GASTRITIS	0	0	0	0	0	0	0	0	0	0
STAPHYLOCOCCAL INFECTIONS										
FURUNCLE	0	0	0	0	0	0	0	0	0	0
FUNGAL INFECTIOUS DISORDERS										
CANDIDA INFECTIONS										
CANDIDA INFECTION	0	0	0	0	0	0	0	0	0	0
VULVOVAGINAL CANDIDIASIS	0	0	0	0	0	0	0	0	0	0
INFECTIONS - PATHOGEN UNSPECIFIED										
ABDOMINAL AND GASTROINTESTINAL INFECTIONS										
APPENDICITIS	0	0	0	0	0	0	0	0	0	0
DIARRHOEA INFECTIOUS	1	0	0	1	0	1	0	0	1	0
GASTROINTESTINAL INFECTION	0	0	0	0	0	0	0	0	0	0
BREAST INFECTIONS										
MASTITIS	1	0	0	1	0	1	0	0	1	0
CENTRAL NERVOUS SYSTEM AND SPINAL INFECTIONS										
MYELITIS	0	0	0	0	0	0	0	0	0	0
DENTAL AND ORAL SOFT TISSUE INFECTIONS										
ABSCESS ORAL	0	0	0	0	0	0	0	0	0	0
ORAL PUSTULE	0	0	0	0	0	0	0	0	0	0
PERICORONITIS	0	0	0	0	0	0	0	0	0	0
TOOTH ABSCESS	0	0	0	0	0	0	0	0	0	0
EAR INFECTIONS										
EAR INFECTION	0	0	0	0	0	0	0	0	0	0
LABYRINTHITIS	0	0	0	0	0	0	0	0	0	0
OTITIS EXTERNA	0	0	0	0	0	0	0	0	0	0
EYE AND EYELID INFECTIONS										
CONJUNCTIVITIS	1	0	1	0	0	0	0	0	0	0

HORDEOLUM	0	0	0	0	0	0	0	0	0	0
INFECTIONS NEC										
ABSCESS	0	0	0	0	0	0	0	0	0	0
INFECTION	1	1	0	0	0	1	1	0	0	0
INJECTION SITE INFECTION	0	0	0	0	0	0	0	0	0	0
LOCALISED INFECTION	1	1	0	0	0	1	1	0	0	0
WOUND INFECTION	0	0	0	0	0	0	0	0	0	0
LOWER RESPIRATORY TRACT AND LUNG INFECTIONS										
LOWER RESPIRATORY TRACT INFECTION	1	1	0	0	0	1	1	0	0	0
PNEUMONIA	0	0	0	0	0	0	0	0	0	0
MALE REPRODUCTIVE TRACT INFECTIONS										
ORCHITIS	0	0	0	0	0	0	0	0	0	0
SEPSIS, BACTERAEMIA, VIRAEMIA AND FUNGAEMIA NEC										
SEPTIC RASH	0	0	0	0	0	0	0	0	0	0
SKIN STRUCTURES AND SOFT TISSUE INFECTIONS										
INFECTED DERMAL CYST	0	0	0	0	0	0	0	0	0	0
INJECTION SITE PUSTULE	0	0	0	0	0	0	0	0	0	0
SKIN INFECTION	0	0	0	0	0	0	0	0	0	0
UPPER RESPIRATORY TRACT INFECTIONS										
LARYNGITIS	1	1	0	0	0	1	1	0	0	0
NASOPHARYNGITIS	18	2	2	13	1	16	2	2	11	1
PHARYNGITIS	0	0	0	0	0	0	0	0	0	0
RHINITIS	0	0	0	0	0	0	0	0	0	0
SINUSITIS	0	0	0	0	0	0	0	0	0	0
TONSILLITIS	0	0	0	0	0	0	0	0	0	0
URINARY TRACT INFECTIONS										
CYSTITIS	0	0	0	0	0	0	0	0	0	0
URINARY TRACT INFECTION	0	0	0	0	0	0	0	0	0	0
PROTOZOAL INFECTIOUS DISORDERS										
TRYPANOSOMAL INFECTIONS										
AFRICAN TRYPANOSOMIASIS	0	0	0	0	0	0	0	0	0	0
RICKETTSIAL INFECTIOUS DISORDERS										
COXIELLA INFECTIONS										
Q FEVER	0	0	0	0	0	0	0	0	0	0
VIRAL INFECTIOUS DISORDERS										
CORONAVIRUS INFECTIONS										

COVID-19	3	0	0	0	3	2	0	0	0	2
HERPES VIRAL INFECTIONS										
GENITAL HERPES	0	0	0	0	0	0	0	0	0	0
HERPES OPHTHALMIC	0	0	0	0	0	0	0	0	0	0
HERPES SIMPLEX	0	0	0	0	0	0	0	0	0	0
HERPES ZOSTER	0	0	0	0	0	0	0	0	0	0
NASAL HERPES	0	0	0	0	0	0	0	0	0	0
OPHTHALMIC HERPES ZOSTER	0	0	0	0	0	0	0	0	0	0
ORAL HERPES	1	1	0	0	0	1	1	0	0	0
INFLUENZA VIRAL INFECTIONS										
H2N2 INFLUENZA	0	0	0	0	0	0	0	0	0	0
INFLUENZA	47	2	10	29	6	36	2	8	21	5
RETROVIRAL INFECTIONS										
AIDS RELATED COMPLEX	0	0	0	0	0	0	0	0	0	0
VIRAL INFECTIONS NEC										
GASTROENTERITIS VIRAL	0	0	0	0	0	0	0	0	0	0
SWEATING FEVER	0	0	0	0	0	0	0	0	0	0
VESTIBULAR NEURONITIS	1	0	0	0	1	0	0	0	0	0
VIRAL DIARRHOEA	0	0	0	0	0	0	0	0	0	0
VIRAL RASH	0	0	0	0	0	0	0	0	0	0
VIRAL UPPER RESPIRATORY TRACT INFECTION	0	0	0	0	0	0	0	0	0	0
**INJURY, POISONING AND PROCEDURAL COMPLICATIONS**										
BONE AND JOINT INJURIES										
FRACTURES AND DISLOCATIONS NEC										
JOINT DISLOCATION	0	0	0	0	0	0	0	0	0	0
EXPOSURES, CHEMICAL INJURIES AND POISONING										
POISONING AND TOXICITY										
SYSTEMIC TOXICITY	0	0	0	0	0	0	0	0	0	0
TOXICITY TO VARIOUS AGENTS	0	0	0	0	0	0	0	0	0	0
INJURIES BY PHYSICAL AGENTS										
CONDITIONS CAUSED BY COLD										
CHILLBLAINS	0	0	0	0	0	0	0	0	0	0
HEAT INJURIES (EXCL THERMAL BURNS)										
HEAT EXHAUSTION	0	0	0	0	0	0	0	0	0	0
HEAT OEDEMA	0	0	0	0	0	0	0	0	0	0
RADIATION INJURIES										

SUNBURN	0	0	0	0	0	0	0	0	0	0
THERMAL BURNS										
THERMAL BURN	0	0	0	0	0	0	0	0	0	0
THERMAL BURNS OF EYE	0	0	0	0	0	0	0	0	0	0
INJURIES NEC										
CHEST AND RESPIRATORY TRACT INJURIES NEC										
CHEST CRUSHING	0	0	0	0	0	0	0	0	0	0
EYE INJURIES NEC										
EYE CONTUSION	0	0	0	0	0	0	0	0	0	0
MUSCLE, TENDON AND LIGAMENT INJURIES										
LIGAMENT SPRAIN	0	0	0	0	0	0	0	0	0	0
MUSCLE INJURY	0	0	0	0	0	0	0	0	0	0
MUSCLE STRAIN	0	0	0	0	0	0	0	0	0	0
NERVE INJURIES NEC										
NERVE INJURY	0	0	0	0	0	0	0	0	0	0
NON-SITE SPECIFIC INJURIES NEC										
ARTHROPOD STING	0	0	0	0	0	0	0	0	0	0
BITE	0	0	0	0	0	0	0	0	0	0
FALL	1	0	0	1	0	1	0	0	1	0
INFLAMMATION OF WOUND	0	0	0	0	0	0	0	0	0	0
TISSUE INJURY	0	0	0	0	0	0	0	0	0	0
WOUND COMPLICATION	0	0	0	0	0	0	0	0	0	0
WOUND SECRETION	0	0	0	0	0	0	0	0	0	0
SITE SPECIFIC INJURIES NEC										
LIMB INJURY	0	0	0	0	0	0	0	0	0	0
SKIN INJURIES NEC										
CONTUSION	7	3	0	4	0	6	3	0	3	0
SCAR	0	0	0	0	0	0	0	0	0	0
SKIN WOUND	0	0	0	0	0	0	0	0	0	0
PROCEDURAL RELATED INJURIES AND COMPLICATIONS NEC										
CARDIAC AND VASCULAR PROCEDURAL COMPLICATIONS										
CARDIAC PROCEDURE COMPLICATION	0	0	0	0	0	0	0	0	0	0
GASTROINTESTINAL AND HEPATOBILIARY PROCEDURAL COMPLICATIONS										
PROCEDURAL NAUSEA	0	0	0	0	0	0	0	0	0	0
NEUROLOGICAL AND PSYCHIATRIC PROCEDURAL COMPLICATIONS										
PROCEDURAL DIZZINESS	0	0	0	0	0	0	0	0	0	0

NON-SITE SPECIFIC PROCEDURAL COMPLICATIONS										
INCISION SITE PAIN	0	0	0	0	0	0	0	0	0	0
INCISION SITE SWELLING	0	0	0	0	0	0	0	0	0	0
INJECTION RELATED REACTION	4	0	0	1	3	4	0	0	1	3
POST PROCEDURAL COMPLICATION	2	0	0	1	1	0	0	0	0	0
PROCEDURAL PAIN	0	0	0	0	0	0	0	0	0	0
VACCINATION RELATED COMPLICATIONS										
IMMUNISATION REACTION	0	0	0	0	0	0	0	0	0	0
**INVESTIGATIONS**										
CARDIAC AND VASCULAR INVESTIGATIONS (EXCL ENZYME TESTS)										
HEART RATE AND PULSE INVESTIGATIONS										
HEART RATE	1	0	0	1	0	1	0	0	1	0
HEART RATE DECREASED	0	0	0	0	0	0	0	0	0	0
HEART RATE INCREASED	3	2	0	1	0	3	2	0	1	0
HEART RATE IRREGULAR	0	0	0	0	0	0	0	0	0	0
VASCULAR TESTS NEC (INCL BLOOD PRESSURE)										
BLOOD PRESSURE DECREASED	0	0	0	0	0	0	0	0	0	0
BLOOD PRESSURE INCREASED	0	0	0	0	0	0	0	0	0	0
BLOOD PRESSURE MEASUREMENT	1	0	0	1	0	0	0	0	0	0
ENDOCRINE INVESTIGATIONS (INCL SEX HORMONES)										
ENDOCRINE ANALYSES AND IMAGING NEC										
HORMONE LEVEL ABNORMAL	0	0	0	0	0	0	0	0	0	0
PITUITARY ANALYSES ANTERIOR										
BLOOD FOLLICLE STIMULATING HORMONE INCREASED	1	0	0	1	0	1	0	0	1	0
BLOOD LUTEINISING HORMONE	0	0	0	0	0	0	0	0	0	0
THYROID ANALYSES										
TRI-IODOTHYRONINE	0	0	0	0	0	0	0	0	0	0
HAEMATOLOGY INVESTIGATIONS (INCL BLOOD GROUPS)										
COAGULATION AND BLEEDING ANALYSES										
BLEEDING TIME	0	0	0	0	0	0	0	0	0	0
INTERNATIONAL NORMALISED RATIO DECREASED	0	0	0	0	0	0	0	0	0	0
PLATELET ANALYSES										
PLATELET COUNT INCREASED	0	0	0	0	0	0	0	0	0	0
RED BLOOD CELL ANALYSES										
HAEMOGLOBIN	0	0	0	0	0	0	0	0	0	0

METABOLIC, NUTRITIONAL AND BLOOD GAS INVESTIGATIONS										
BLOOD GAS AND ACID BASE ANALYSES										
OXYGEN SATURATION DECREASED	0	0	0	0	0	0	0	0	0	0
CARBOHYDRATE TOLERANCE ANALYSES (INCL DIABETES)										
BLOOD GLUCOSE	0	0	0	0	0	0	0	0	0	0
BLOOD GLUCOSE ABNORMAL	0	0	0	0	0	0	0	0	0	0
BLOOD GLUCOSE DECREASED	0	0	0	0	0	0	0	0	0	0
BLOOD GLUCOSE INCREASED	0	0	0	0	0	0	0	0	0	0
MICROBIOLOGY AND SEROLOGY INVESTIGATIONS										
VIRUS IDENTIFICATION AND SEROLOGY										
SARS-COV-2 TEST	0	0	0	0	0	0	0	0	0	0
SARS-COV-2 TEST POSITIVE	0	0	0	0	0	0	0	0	0	0
MUSCULOSKELETAL AND SOFT TISSUE INVESTIGATIONS (EXCL ENZYME TESTS)										
MUSCULOSKELETAL AND SOFT TISSUE IMAGING PROCEDURES										
BONE SCAN	0	0	0	0	0	0	0	0	0	0
NEUROLOGICAL, SPECIAL SENSES AND PSYCHIATRIC INVESTIGATIONS										
CENTRAL NERVOUS SYSTEM IMAGING PROCEDURES										
MAGNETIC RESONANCE IMAGING HEAD	0	0	0	0	0	0	0	0	0	0
SCAN BRAIN	0	0	0	0	0	0	0	0	0	0
PHYSICAL EXAMINATION AND ORGAN SYSTEM STATUS TOPICS										
PHYSICAL EXAMINATION PROCEDURES AND ORGAN SYSTEM STATUS										
BODY TEMPERATURE	2	1	0	1	0	2	1	0	1	0
BODY TEMPERATURE ABNORMAL	1	0	0	1	0	1	0	0	1	0
BODY TEMPERATURE DECREASED	0	0	0	0	0	0	0	0	0	0
BODY TEMPERATURE FLUCTUATION	2	0	1	1	0	2	0	1	1	0
BODY TEMPERATURE INCREASED	7	2	0	5	0	7	2	0	5	0
GRIP STRENGTH DECREASED	0	0	0	0	0	0	0	0	0	0
HEAD LAG	1	1	0	0	0	1	1	0	0	0
LEFT-HANDEDNESS	0	0	0	0	0	0	0	0	0	0
LYMPH NODE PALPABLE	1	0	0	1	0	1	0	0	1	0
RESPIRATORY RATE DECREASED	0	0	0	0	0	0	0	0	0	0
SKIN TEMPERATURE	0	0	0	0	0	0	0	0	0	0
WEIGHT DECREASED	0	0	0	0	0	0	0	0	0	0
WEIGHT INCREASED	0	0	0	0	0	0	0	0	0	0
RENAL AND URINARY TRACT INVESTIGATIONS AND URINALYSES										
URINALYSIS NEC										

BLOOD URINE	0	0	0	0	0	0	0	0	0	0
NITRITE URINE PRESENT	0	0	0	0	0	0	0	0	0	0
PH URINE	0	0	0	0	0	0	0	0	0	0
URINARY TRACT FUNCTION ANALYSES NEC										
URINE OUTPUT	0	0	0	0	0	0	0	0	0	0
URINE OUTPUT INCREASED	0	0	0	0	0	0	0	0	0	0
REPRODUCTIVE ORGAN AND BREAST INVESTIGATIONS (EXCL HORMONE ANALYSES)										
REPRODUCTIVE ORGAN AND BREAST IMAGING PROCEDURES										
BREAST SCAN	0	0	0	0	0	0	0	0	0	0
RESPIRATORY AND PULMONARY INVESTIGATIONS (EXCL BLOOD GASES)										
RESPIRATORY AND PULMONARY FUNCTION DIAGNOSTIC PROCEDURES										
FORCED EXPIRATORY VOLUME DECREASED	0	0	0	0	0	0	0	0	0	0
FORCED EXPIRATORY VOLUME INCREASED	0	0	0	0	0	0	0	0	0	0
WATER, ELECTROLYTE AND MINERAL INVESTIGATIONS										
WATER AND ELECTROLYTE ANALYSES NEC										
VOLUME BLOOD	0	0	0	0	0	0	0	0	0	0
**METABOLISM AND NUTRITION DISORDERS**										
APPETITE AND GENERAL NUTRITIONAL DISORDERS										
APPETITE DISORDERS										
APPETITE DISORDER	0	0	0	0	0	0	0	0	0	0
DECREASED APPETITE	8	2	1	4	1	8	2	1	4	1
FOOD CRAVING	0	0	0	0	0	0	0	0	0	0
FOOD REFUSAL	1	0	0	1	0	0	0	0	0	0
INCREASED APPETITE	0	0	0	0	0	0	0	0	0	0
GENERAL NUTRITIONAL DISORDERS NEC										
FOOD AVERSION	0	0	0	0	0	0	0	0	0	0
ELECTROLYTE AND FLUID BALANCE CONDITIONS										
TOTAL FLUID VOLUME DECREASED										
DEHYDRATION	1	1	0	0	0	1	1	0	0	0
TOTAL FLUID VOLUME INCREASED										
FLUID RETENTION	0	0	0	0	0	0	0	0	0	0
FOOD INTOLERANCE SYNDROMES										
FOOD MALABSORPTION AND INTOLERANCE SYNDROMES (EXCL SUGAR INTOLERANCE)										
ALCOHOL INTOLERANCE	0	0	0	0	0	0	0	0	0	0
GLUCOSE METABOLISM DISORDERS (INCL DIABETES MELLITUS)										
DIABETES MELLITUS (INCL SUBTYPES)										

DIABETES MELLITUS	0	0	0	0	0	0	0	0	0	0
DIABETES MELLITUS INADEQUATE CONTROL	0	0	0	0	0	0	0	0	0	0
HYPERGLYCAEMIC CONDITIONS NEC										
HYPERGLYCAEMIA	0	0	0	0	0	0	0	0	0	0
HYPOGLYCAEMIC CONDITIONS NEC										
HYPOGLYCAEMIA	0	0	0	0	0	0	0	0	0	0
PURINE AND PYRIMIDINE METABOLISM DISORDERS										
DISORDERS OF PURINE METABOLISM										
GOUT	0	0	0	0	0	0	0	0	0	0
**MUSCULOSKELETAL AND CONNECTIVE TISSUE DISORDERS**										
BONE DISORDERS (EXCL CONGENITAL AND FRACTURES)										
BONE DISORDERS NEC										
JAW DISORDER	0	0	0	0	0	0	0	0	0	0
OSTEITIS	0	0	0	0	0	0	0	0	0	0
BONE RELATED SIGNS AND SYMPTOMS										
BONE PAIN	1	0	0	0	1	0	0	0	0	0
BONE SWELLING	0	0	0	0	0	0	0	0	0	0
PAIN IN JAW	1	0	1	0	0	1	0	1	0	0
PUBIC PAIN	0	0	0	0	0	0	0	0	0	0
SPINAL PAIN	0	0	0	0	0	0	0	0	0	0
CONNECTIVE TISSUE DISORDERS (EXCL CONGENITAL)										
CONNECTIVE TISSUE DISORDERS NEC										
POLYMYALGIA RHEUMATICA	1	0	0	0	1	1	0	0	0	1
LUPUS ERYTHEMATOSUS (INCL SUBTYPES)										
SYSTEMIC LUPUS ERYTHEMATOSUS	0	0	0	0	0	0	0	0	0	0
JOINT DISORDERS										
ARTHROPATHIES NEC										
ARTHRITIS	0	0	0	0	0	0	0	0	0	0
ARTHROPATHY	0	0	0	0	0	0	0	0	0	0
POLYARTHRITIS	0	0	0	0	0	0	0	0	0	0
RHEUMATIC FEVER	0	0	0	0	0	0	0	0	0	0
JOINT RELATED DISORDERS NEC										
JOINT LOCK	0	0	0	0	0	0	0	0	0	0
PERIARTHRITIS	1	0	0	1	0	1	0	0	1	0
TEMPOROMANDIBULAR PAIN AND DYSFUNCTION SYNDROME	0	0	0	0	0	0	0	0	0	0

JOINT RELATED SIGNS AND SYMPTOMS										
ARTHRALGIA	84	16	9	47	12	70	16	8	37	9
JAW CLICKING	0	0	0	0	0	0	0	0	0	0
JOINT STIFFNESS	3	1	0	1	1	3	1	0	1	1
JOINT SWELLING	0	0	0	0	0	0	0	0	0	0
OSTEOARTHROPATHIES										
OSTEOARTHRITIS	0	0	0	0	0	0	0	0	0	0
PSORIATIC ARTHROPATHIES										
PSORIATIC ARTHROPATHY	0	0	0	0	0	0	0	0	0	0
RHEUMATOID ARTHROPATHIES										
RHEUMATOID ARTHRITIS	0	0	0	0	0	0	0	0	0	0
MUSCLE DISORDERS										
MUSCLE INFECTIONS AND INFLAMMATIONS										
MYOSITIS	0	0	0	0	0	0	0	0	0	0
MUSCLE PAINS										
FIBROMYALGIA	0	0	0	0	0	0	0	0	0	0
MYALGIA	112	21	17	51	23	92	21	11	40	20
MUSCLE RELATED SIGNS AND SYMPTOMS NEC										
MUSCLE ATROPHY	0	0	0	0	0	0	0	0	0	0
MUSCLE DISCOMFORT	0	0	0	0	0	0	0	0	0	0
MUSCLE FATIGUE	4	2	0	2	0	3	2	0	1	0
MUSCLE FIBROSIS	0	0	0	0	0	0	0	0	0	0
MUSCLE MASS	0	0	0	0	0	0	0	0	0	0
MUSCLE SPASMS	2	0	0	1	1	2	0	0	1	1
MUSCLE TIGHTNESS	0	0	0	0	0	0	0	0	0	0
MUSCLE TWITCHING	0	0	0	0	0	0	0	0	0	0
MUSCLE TONE ABNORMALITIES										
MUSCLE RIGIDITY	0	0	0	0	0	0	0	0	0	0
TRISMUS	1	0	1	0	0	1	0	1	0	0
MUSCLE WEAKNESS CONDITIONS										
MUSCULAR WEAKNESS	4	2	1	1	0	4	2	1	1	0
MUSCULOSKELETAL AND CONNECTIVE TISSUE DISORDERS NEC										
MUSCULOSKELETAL AND CONNECTIVE TISSUE CONDITIONS NEC										
MOBILITY DECREASED	1	0	0	0	1	1	0	0	0	1
MUSCULOSKELETAL STIFFNESS	17	3	3	5	6	15	3	3	4	5

BACK PAIN	17	3	5	9	0	15	3	5	7	0
FLANK PAIN	0	0	0	0	0	0	0	0	0	0
LIMB DISCOMFORT	43	13	3	25	2	42	13	3	24	2
MUSCULOSKELETAL CHEST PAIN	0	0	0	0	0	0	0	0	0	0
MUSCULOSKELETAL DISCOMFORT	3	0	0	3	0	3	0	0	3	0
MUSCULOSKELETAL PAIN	0	0	0	0	0	0	0	0	0	0
NECK PAIN	7	2	1	2	2	7	2	1	2	2
PAIN IN EXTREMITY	399	141	26	179	53	354	141	17	155	41
SOFT TISSUE DISORDERS NEC										
AXILLARY MASS	0	0	0	0	0	0	0	0	0	0
GROIN PAIN	0	0	0	0	0	0	0	0	0	0
SYNOVIAL AND BURSAL DISORDERS										
SYNOVIAL DISORDERS										
SYNOVITIS	0	0	0	0	0	0	0	0	0	0
TENDON, LIGAMENT AND CARTILAGE DISORDERS										
CARTILAGE DISORDERS										
COSTOCHONDRITIS	0	0	0	0	0	0	0	0	0	0
TENDON DISORDERS										
TENDONITIS	0	0	0	0	0	0	0	0	0	0
TENOSYNOVITIS	0	0	0	0	0	0	0	0	0	0
TRIGGER FINGER	1	0	0	1	0	1	0	0	1	0
**NEOPLASMS BENIGN, MALIGNANT AND UNSPECIFIED (INCL CYSTS AND POLYPS)**										
CUTANEOUS NEOPLASMS BENIGN										
SKIN NEOPLASMS BENIGN										
MELANOCYTIC NAEVUS	0	0	0	0	0	0	0	0	0	0
SEBORRHOEIC KERATOSIS	1	0	0	1	0	1	0	0	1	0
SKIN PAPILLOMA	0	0	0	0	0	0	0	0	0	0
**NERVOUS SYSTEM DISORDERS**										
CENTRAL NERVOUS SYSTEM INFECTIONS AND INFLAMMATIONS										
MYELITIS (INCL INFECTIVE)										
MYELITIS TRANSVERSE	0	0	0	0	0	0	0	0	0	0
CENTRAL NERVOUS SYSTEM VASCULAR DISORDERS										
CENTRAL NERVOUS SYSTEM HAEMORRHAGES AND CEREBROVASCULAR ACCIDENTS										
CEREBRAL HAEMORRHAGE	0	0	0	0	0	0	0	0	0	0
CEREBROVASCULAR ACCIDENT	0	0	0	0	0	0	0	0	0	0
TRANSIENT CEREBROVASCULAR EVENTS										

TRANSIENT ISCHAEMIC ATTACK	0	0	0	0	0	0	0	0	0	0
CRANIAL NERVE DISORDERS (EXCL NEOPLASMS)										
FACIAL CRANIAL NERVE DISORDERS										
BELL'S PALSY	0	0	0	0	0	0	0	0	0	0
FACIAL PARALYSIS	0	0	0	0	0	0	0	0	0	0
FACIAL PARESIS	1	0	0	1	0	1	0	0	1	0
OLFACTORY NERVE DISORDERS										
ANOSMIA	1	0	0	1	0	1	0	0	1	0
HYPOSMIA	0	0	0	0	0	0	0	0	0	0
PAROSMIA	0	0	0	0	0	0	0	0	0	0
TRIGEMINAL DISORDERS										
TRIGEMINAL NEURALGIA	0	0	0	0	0	0	0	0	0	0
TRIGEMINAL NEURITIS	0	0	0	0	0	0	0	0	0	0
DEMYELINATING DISORDERS										
MULTIPLE SCLEROSIS ACUTE AND PROGRESSIVE										
MULTIPLE SCLEROSIS RELAPSE	0	0	0	0	0	0	0	0	0	0
HEADACHES										
HEADACHES NEC										
CLUSTER HEADACHE	1	0	0	0	1	1	0	0	0	1
COLD-STIMULUS HEADACHE	0	0	0	0	0	0	0	0	0	0
DRUG WITHDRAWAL HEADACHE	0	0	0	0	0	0	0	0	0	0
HEADACHE	263	57	41	130	35	221	57	28	106	30
PRIMARY STABBING HEADACHE	0	0	0	0	0	0	0	0	0	0
SINUS HEADACHE	5	1	1	1	2	3	1	1	1	0
TENSION HEADACHE	10	3	2	1	4	10	3	2	1	4
THUNDERCLAP HEADACHE	0	0	0	0	0	0	0	0	0	0
VASCULAR HEADACHE	0	0	0	0	0	0	0	0	0	0
MIGRAINE HEADACHES										
MIGRAINE	6	3	0	3	0	6	3	0	3	0
MIGRAINE WITH AURA	1	0	1	0	0	1	0	1	0	0
RETINAL MIGRAINE	0	0	0	0	0	0	0	0	0	0
TYPICAL AURA WITHOUT HEADACHE	0	0	0	0	0	0	0	0	0	0
MENTAL IMPAIRMENT DISORDERS										
INTELLECTUAL DISABILITIES										
INTELLECTUAL DISABILITY	1	0	0	1	0	1	0	0	1	0

MEMORY LOSS (EXCL DEMENTIA)										
AMNESIA	0	0	0	0	0	0	0	0	0	0
MEMORY IMPAIRMENT	1	0	0	1	0	1	0	0	1	0
MENTAL IMPAIRMENT (EXCL DEMENTIA AND MEMORY LOSS)										
COGNITIVE DISORDER	0	0	0	0	0	0	0	0	0	0
DISTURBANCE IN ATTENTION	0	0	0	0	0	0	0	0	0	0
MENTAL IMPAIRMENT	0	0	0	0	0	0	0	0	0	0
MOVEMENT DISORDERS (INCL PARKINSONISM)										
DYSKINESIAS AND MOVEMENT DISORDERS NEC										
BRADYKINESIA	2	1	0	1	0	2	1	0	1	0
CLUMSINESS	0	0	0	0	0	0	0	0	0	0
DYSKINESIA	0	0	0	0	0	0	0	0	0	0
EXTRAPYRAMIDAL DISORDER	0	0	0	0	0	0	0	0	0	0
PSYCHOMOTOR HYPERACTIVITY	0	0	0	0	0	0	0	0	0	0
PARALYSIS AND PARESIS (EXCL CRANIAL NERVE)										
HEMIPLEGIA	1	0	0	1	0	1	0	0	1	0
MONOPARESIS	1	0	0	1	0	0	0	0	0	0
MONOPLEGIA	0	0	0	0	0	0	0	0	0	0
PARALYSIS	1	0	0	1	0	1	0	0	1	0
PARKINSON'S DISEASE AND PARKINSONISM										
FREEZING PHENOMENON	1	0	0	1	0	1	0	0	1	0
PARKINSON'S DISEASE	0	0	0	0	0	0	0	0	0	0
TREMOR (EXCL CONGENITAL)										
TREMOR	7	1	3	2	1	6	1	3	1	1
NEUROLOGICAL DISORDERS NEC										
COMA STATES										
DIABETIC HYPERGLYCAEMIC COMA	0	0	0	0	0	0	0	0	0	0
COORDINATION AND BALANCE DISTURBANCES										
BALANCE DISORDER	1	0	0	0	1	1	0	0	0	1
COORDINATION ABNORMAL	0	0	0	0	0	0	0	0	0	0
DYSSTASIA	0	0	0	0	0	0	0	0	0	0
VESTIBULAR NYSTAGMUS	0	0	0	0	0	0	0	0	0	0
DISTURBANCES IN CONSCIOUSNESS NEC										
DEPRESSED LEVEL OF CONSCIOUSNESS	0	0	0	0	0	0	0	0	0	0
LETHARGY	23	4	2	11	6	19	4	2	11	2

LOSS OF CONSCIOUSNESS	1	0	0	1	0	1	0	0	1	0
SEDATION	1	0	0	0	1	1	0	0	0	1
SOMNOLENCE	14	4	5	5	0	12	4	5	3	0
SYNCOPE	5	2	0	3	0	4	2	0	2	0
NERVOUS SYSTEM DISORDERS NEC										
NERVOUS SYSTEM DISORDER	0	0	0	0	0	0	0	0	0	0
NEUROLOGICAL SIGNS AND SYMPTOMS NEC										
AGITATION NEONATAL	0	0	0	0	0	0	0	0	0	0
BRAIN FOG	0	0	0	0	0	0	0	0	0	0
DIZZINESS	40	11	5	16	8	31	11	2	12	6
DIZZINESS EXERTIONAL	1	1	0	0	0	1	1	0	0	0
DIZZINESS POSTURAL	3	1	0	1	1	3	1	0	1	1
HEAD DISCOMFORT	2	0	2	0	0	2	0	2	0	0
INFANT IRRITABILITY	0	0	0	0	0	0	0	0	0	0
MENINGISM	0	0	0	0	0	0	0	0	0	0
MYOCLONUS	0	0	0	0	0	0	0	0	0	0
NEUROLOGICAL SYMPTOM	0	0	0	0	0	0	0	0	0	0
PERSISTENT POSTURAL-PERCEPTUAL DIZZINESS	0	0	0	0	0	0	0	0	0	0
PRESYNCOPE	2	0	0	2	0	2	0	0	2	0
PARAESTHESIAS AND DYSAESTHESIAS										
BURNING FEET SYNDROME	0	0	0	0	0	0	0	0	0	0
BURNING SENSATION	0	0	0	0	0	0	0	0	0	0
FORMICATION	0	0	0	0	0	0	0	0	0	0
HYPERAESTHESIA	0	0	0	0	0	0	0	0	0	0
HYPOAESTHESIA	2	2	0	0	0	2	2	0	0	0
PARAESTHESIA	4	1	1	1	1	4	1	1	1	1
REVERSED HOT-COLD SENSATION	0	0	0	0	0	0	0	0	0	0
SENSORY ABNORMALITIES NEC										
AGEUSIA	1	0	0	1	0	1	0	0	1	0
ALLODYNIA	1	0	1	0	0	0	0	0	0	0
DYSGEUSIA	3	0	0	2	1	3	0	0	2	1
HYPOGEUSIA	0	0	0	0	0	0	0	0	0	0
NEURALGIA	0	0	0	0	0	0	0	0	0	0
POST HERPETIC NEURALGIA	0	0	0	0	0	0	0	0	0	0
RESTLESS ARM SYNDROME	0	0	0	0	0	0	0	0	0	0

RESTLESS LEGS SYNDROME	0	0	0	0	0	0	0	0	0	0
SENSORY LOSS	0	0	0	0	0	0	0	0	0	0
TASTE DISORDER	1	1	0	0	0	1	1	0	0	0
SPEECH AND LANGUAGE ABNORMALITIES										
DYSARTHRIA	1	1	0	0	0	1	1	0	0	0
SPEECH DISORDER DEVELOPMENTAL	1	0	0	1	0	1	0	0	1	0
NEUROLOGICAL DISORDERS OF THE EYE										
NEUROLOGIC VISUAL PROBLEMS NEC										
TUNNEL VISION	0	0	0	0	0	0	0	0	0	0
NEUROMUSCULAR DISORDERS										
MUSCLE TONE ABNORMAL										
HYPOTONIA	0	0	0	0	0	0	0	0	0	0
STIFF LEG SYNDROME	0	0	0	0	0	0	0	0	0	0
NEUROMUSCULAR DISORDERS NEC										
MUSCLE SPASTICITY	0	0	0	0	0	0	0	0	0	0
PERIPHERAL NEUROPATHIES										
ACUTE POLYNEUROPATHIES										
GUILLAIN-BARRE SYNDROME	0	0	0	0	0	0	0	0	0	0
PERIPHERAL NEUROPATHIES NEC										
AXONAL NEUROPATHY	0	0	0	0	0	0	0	0	0	0
NEUROPATHY PERIPHERAL	1	0	0	1	0	1	0	0	1	0
SEIZURES (INCL SUBTYPES)										
SEIZURES AND SEIZURE DISORDERS NEC										
EPILEPSY	1	0	0	1	0	1	0	0	1	0
SEIZURE	1	0	0	1	0	1	0	0	1	0
SLEEP DISTURBANCES (INCL SUBTYPES)										
DISTURBANCES IN SLEEP PHASE RHYTHM										
CIRCADIAN RHYTHM SLEEP DISORDER	0	0	0	0	0	0	0	0	0	0
SLEEP DISTURBANCES NEC										
SLEEP DEFICIT	0	0	0	0	0	0	0	0	0	0
SPINAL CORD AND NERVE ROOT DISORDERS										
CERVICAL SPINAL CORD AND NERVE ROOT DISORDERS										
CERVICOBRACHIAL SYNDROME	1	0	0	1	0	1	0	0	1	0
LUMBAR SPINAL CORD AND NERVE ROOT DISORDERS										
CAUDA EQUINA SYNDROME	1	0	0	1	0	1	0	0	1	0
SCIATICA	0	0	0	0	0	0	0	0	0	0

**PREGNANCY, PUERPERIUM AND PERINATAL CONDITIONS**										
ABORTIONS AND STILLBIRTH										
ABORTIONS SPONTANEOUS										
ABORTION SPONTANEOUS	1	1	0	0	0	1	1	0	0	0
STILLBIRTH AND FOETAL DEATH										
FOETAL DEATH	0	0	0	0	0	0	0	0	0	0
FOETAL COMPLICATIONS										
FOETAL COMPLICATIONS NEC										
FOETAL DISORDER	0	0	0	0	0	0	0	0	0	0
FOETAL HYPOKINESIA	0	0	0	0	0	0	0	0	0	0
FOETAL GROWTH COMPLICATIONS										
FOETAL MACROSOMIA	0	0	0	0	0	0	0	0	0	0
MATERNAL COMPLICATIONS OF PREGNANCY										
MATERNAL COMPLICATIONS OF PREGNANCY NEC										
MORNING SICKNESS	0	0	0	0	0	0	0	0	0	0
PLACENTAL, AMNIOTIC AND CAVITY DISORDERS (EXCL HAEMORRHAGES)										
PLACENTAL ABNORMALITIES (EXCL NEOPLASMS)										
PLACENTAL INFARCTION	0	0	0	0	0	0	0	0	0	0
PREGNANCY, LABOUR, DELIVERY AND POSTPARTUM CONDITIONS										
NORMAL PREGNANCY, LABOUR AND DELIVERY										
PREGNANCY	0	0	0	0	0	0	0	0	0	0
UTERINE CONTRACTIONS DURING PREGNANCY	0	0	0	0	0	0	0	0	0	0
**PRODUCT ISSUES**										
DEVICE ISSUES										
DEVICE MALFUNCTION EVENTS NEC										
OVERSENSING	0	0	0	0	0	0	0	0	0	0
**PSYCHIATRIC DISORDERS**										
ANXIETY DISORDERS AND SYMPTOMS										
ANXIETY SYMPTOMS										
AGITATION	0	0	0	0	0	0	0	0	0	0
ANXIETY	2	0	0	2	0	2	0	0	2	0
NERVOUSNESS	0	0	0	0	0	0	0	0	0	0
TENSION	0	0	0	0	0	0	0	0	0	0
PANIC ATTACKS AND DISORDERS										
PANIC ATTACK	0	0	0	0	0	0	0	0	0	0
CHANGES IN PHYSICAL ACTIVITY										

INCREASED PHYSICAL ACTIVITY LEVELS										
RESTLESSNESS	1	0	0	0	1	1	0	0	0	1
STEREOTYPIES AND AUTOMATISMS										
BRUXISM	0	0	0	0	0	0	0	0	0	0
COGNITIVE AND ATTENTION DISORDERS AND DISTURBANCES										
COGNITIVE AND ATTENTION DISORDERS AND DISTURBANCES NEC										
DAYDREAMING	0	0	0	0	0	0	0	0	0	0
MENTAL FATIGUE	1	1	0	0	0	1	1	0	0	0
COMMUNICATION DISORDERS AND DISTURBANCES										
SPEECH ARTICULATION AND RHYTHM DISTURBANCES										
DYSPHEMIA	0	0	0	0	0	0	0	0	0	0
DELIRIA (INCL CONFUSION)										
CONFUSION AND DISORIENTATION										
CONFUSIONAL STATE	3	1	1	1	0	2	1	1	0	0
DISORIENTATION	0	0	0	0	0	0	0	0	0	0
DELIRIA										
DELIRIUM	0	0	0	0	0	0	0	0	0	0
DEPRESSED MOOD DISORDERS AND DISTURBANCES										
DEPRESSIVE DISORDERS										
DEPRESSION	0	0	0	0	0	0	0	0	0	0
MOOD ALTERATIONS WITH DEPRESSIVE SYMPTOMS										
DEPRESSED MOOD	0	0	0	0	0	0	0	0	0	0
TEARFULNESS	0	0	0	0	0	0	0	0	0	0
DISSOCIATIVE DISORDERS										
DISSOCIATIVE STATES										
DISSOCIATION	1	0	0	1	0	1	0	0	1	0
DISTURBANCES IN THINKING AND PERCEPTION										
DELUSIONAL SYMPTOMS										
DELUSION	0	0	0	0	0	0	0	0	0	0
HALLUCINATIONS (EXCL SLEEP-RELATED)										
HALLUCINATION	0	0	0	0	0	0	0	0	0	0
PERCEPTION DISTURBANCES NEC										
DEREALISATION	0	0	0	0	0	0	0	0	0	0
THINKING DISTURBANCES										
BRADYPHRENIA	2	1	0	1	0	2	1	0	1	0
THOUGHT BLOCKING	0	0	0	0	0	0	0	0	0	0

MOOD DISORDERS AND DISTURBANCES NEC										
AFFECT ALTERATIONS NEC										
AFFECT LABILITY	1	0	1	0	0	1	0	1	0	0
INAPPROPRIATE AFFECT	0	0	0	0	0	0	0	0	0	0
EMOTIONAL AND MOOD DISTURBANCES NEC										
ANGER	0	0	0	0	0	0	0	0	0	0
EMOTIONAL DISORDER	0	0	0	0	0	0	0	0	0	0
EMOTIONAL DISTRESS	0	0	0	0	0	0	0	0	0	0
EUPHORIC MOOD	0	0	0	0	0	0	0	0	0	0
IRRITABILITY	0	0	0	0	0	0	0	0	0	0
MOOD ALTERED	1	0	0	1	0	1	0	0	1	0
FLUCTUATING MOOD SYMPTOMS										
MOOD SWINGS	0	0	0	0	0	0	0	0	0	0
MOOD DISORDERS NEC										
APATHY	0	0	0	0	0	0	0	0	0	0
LISTLESS	0	0	0	0	0	0	0	0	0	0
PERSONALITY DISORDERS AND DISTURBANCES IN BEHAVIOUR										
BEHAVIOUR AND SOCIALISATION DISTURBANCES										
AGGRESSION	0	0	0	0	0	0	0	0	0	0
PSYCHIATRIC AND BEHAVIOURAL SYMPTOMS NEC										
PSYCHIATRIC SYMPTOMS NEC										
PSYCHIATRIC SYMPTOM	0	0	0	0	0	0	0	0	0	0
PSYCHIATRIC DISORDERS NEC										
MENTAL DISORDERS NEC										
MENTAL DISORDER	0	0	0	0	0	0	0	0	0	0
SCHIZOPHRENIA AND OTHER PSYCHOTIC DISORDERS										
PSYCHOTIC DISORDER NEC										
PSYCHOTIC DISORDER	0	0	0	0	0	0	0	0	0	0
SEXUAL DYSFUNCTIONS, DISTURBANCES AND GENDER IDENTITY DISORDERS										
SEXUAL DESIRE DISORDERS										
LOSS OF LIBIDO	0	0	0	0	0	0	0	0	0	0
SLEEP DISORDERS AND DISTURBANCES										
DISTURBANCES IN INITIATING AND MAINTAINING SLEEP										
INITIAL INSOMNIA	0	0	0	0	0	0	0	0	0	0
INSOMNIA	5	1	1	3	0	4	1	1	2	0
DYSSOMNIAS										

BREATHING-RELATED SLEEP DISORDER	0	0	0	0	0	0	0	0	0	0
POOR QUALITY SLEEP	0	0	0	0	0	0	0	0	0	0
PARASOMNIAS										
ABNORMAL DREAMS	1	0	0	1	0	1	0	0	1	0
EXPLODING HEAD SYNDROME	0	0	0	0	0	0	0	0	0	0
NIGHTMARE	1	1	0	0	0	1	1	0	0	0
SLEEP DISORDERS NEC										
SLEEP DISORDER	0	0	0	0	0	0	0	0	0	0
SOMATIC SYMPTOM AND RELATED DISORDERS										
SOMATIC SYMPTOM DISORDERS										
HABIT COUGH	0	0	0	0	0	0	0	0	0	0
SUICIDAL AND SELF-INJURIOUS BEHAVIOURS NEC										
SUICIDAL AND SELF-INJURIOUS BEHAVIOUR										
SUICIDAL IDEATION	0	0	0	0	0	0	0	0	0	0
**RENAL AND URINARY DISORDERS**										
RENAL DISORDERS (EXCL NEPHROPATHIES)										
RENAL FAILURE AND IMPAIRMENT										
RENAL FAILURE	0	0	0	0	0	0	0	0	0	0
URINARY TRACT SIGNS AND SYMPTOMS										
BLADDER AND URETHRAL SYMPTOMS										
BLADDER PAIN	0	0	0	0	0	0	0	0	0	0
MICTURITION URGENCY	1	0	0	1	0	1	0	0	1	0
POLLAKIURIA	0	0	0	0	0	0	0	0	0	0
URINARY INCONTINENCE	0	0	0	0	0	0	0	0	0	0
URINARY ABNORMALITIES										
HAEMATURIA	0	0	0	0	0	0	0	0	0	0
URINE ABNORMALITY	1	0	0	1	0	1	0	0	1	0
URINE ODOUR ABNORMAL	0	0	0	0	0	0	0	0	0	0
URINARY TRACT SIGNS AND SYMPTOMS NEC										
POLYURIA	1	0	0	0	1	1	0	0	0	1
RENAL PAIN	0	0	0	0	0	0	0	0	0	0
**REPRODUCTIVE SYSTEM AND BREAST DISORDERS**										
BREAST DISORDERS										
BREAST DISORDERS NEC										
BREAST MASS	0	0	0	0	0	0	0	0	0	0
NIPPLE ENLARGEMENT	0	0	0	0	0	0	0	0	0	0

BREAST SIGNS AND SYMPTOMS										
BREAST DISCHARGE	0	0	0	0	0	0	0	0	0	0
BREAST PAIN	2	0	1	1	0	2	0	1	1	0
BREAST SWELLING	0	0	0	0	0	0	0	0	0	0
BREAST TENDERNESS	0	0	0	0	0	0	0	0	0	0
NIPPLE PAIN	0	0	0	0	0	0	0	0	0	0
MENOPAUSE RELATED CONDITIONS										
MENOPAUSAL EFFECTS ON THE GENITOURINARY TRACT										
POSTMENOPAUSAL HAEMORRHAGE	0	0	0	0	0	0	0	0	0	0
MENSTRUAL CYCLE AND UTERINE BLEEDING DISORDERS										
MENSTRUATION AND UTERINE BLEEDING NEC										
DYSMENORRHOEA	2	1	1	0	0	1	1	0	0	0
INTERMENSTRUAL BLEEDING	2	0	0	2	0	1	0	0	1	0
MENSTRUAL DISORDER	2	0	0	2	0	2	0	0	2	0
MENSTRUATION IRREGULAR	5	2	2	1	0	4	2	1	1	0
PREMENSTRUAL PAIN	1	1	0	0	0	1	1	0	0	0
RETROGRADE MENSTRUATION	0	0	0	0	0	0	0	0	0	0
MENSTRUATION WITH DECREASED BLEEDING										
AMENORRHOEA	0	0	0	0	0	0	0	0	0	0
HYPOMENORRHOEA	0	0	0	0	0	0	0	0	0	0
MENSTRUATION DELAYED	1	0	1	0	0	1	0	1	0	0
OLIGOMENORRHOEA	0	0	0	0	0	0	0	0	0	0
MENSTRUATION WITH INCREASED BLEEDING										
HEAVY MENSTRUAL BLEEDING	7	4	1	2	0	6	4	0	2	0
POLYMENORRHOEA	0	0	0	0	0	0	0	0	0	0
PENILE AND SCROTAL DISORDERS (EXCL INFECTIONS AND INFLAMMATIONS)										
SCROTAL DISORDERS NEC										
SCROTAL SWELLING	0	0	0	0	0	0	0	0	0	0
REPRODUCTIVE TRACT DISORDERS NEC										
REPRODUCTIVE TRACT DISORDERS NEC (EXCL NEOPLASMS)										
GENITAL LESION	0	0	0	0	0	0	0	0	0	0
REPRODUCTIVE TRACT SIGNS AND SYMPTOMS NEC										
GENITAL DISCOMFORT	0	0	0	0	0	0	0	0	0	0
GENITAL PAIN	0	0	0	0	0	0	0	0	0	0
UTERINE, PELVIC AND BROAD LIGAMENT DISORDERS										
PELVIS AND BROAD LIGAMENT DISORDERS NEC										

ADNEXA UTERI PAIN	0	0	0	0	0	0	0	0	0	0
VULVOVAGINAL DISORDERS (EXCL INFECTIONS AND INFLAMMATIONS)										
VULVOVAGINAL DISORDERS NEC										
VAGINAL HAEMORRHAGE	0	0	0	0	0	0	0	0	0	0
VULVOVAGINAL SIGNS AND SYMPTOMS										
VAGINAL DISCHARGE	0	0	0	0	0	0	0	0	0	0
**RESPIRATORY, THORACIC AND MEDIASTINAL DISORDERS**										
BRONCHIAL DISORDERS (EXCL NEOPLASMS)										
BRONCHOSPASM AND OBSTRUCTION										
ASTHMA	0	0	0	0	0	0	0	0	0	0
WHEEZING	1	0	1	0	0	1	0	1	0	0
LOWER RESPIRATORY TRACT DISORDERS (EXCL OBSTRUCTION AND INFECTION)										
PULMONARY OEDEMAS										
PULMONARY CONGESTION	0	0	0	0	0	0	0	0	0	0
PULMONARY VASCULAR DISORDERS										
PULMONARY THROMBOTIC AND EMBOLIC CONDITIONS										
PULMONARY EMBOLISM	0	0	0	0	0	0	0	0	0	0
RESPIRATORY DISORDERS NEC										
BREATHING ABNORMALITIES										
DYSPNOEA	10	2	2	3	3	9	2	2	2	3
HYPERVENTILATION	0	0	0	0	0	0	0	0	0	0
HYPOPNOEA	1	0	1	0	0	1	0	1	0	0
IRREGULAR BREATHING	0	0	0	0	0	0	0	0	0	0
MOUTH BREATHING	0	0	0	0	0	0	0	0	0	0
RESPIRATION ABNORMAL	0	0	0	0	0	0	0	0	0	0
RESPIRATORY ARREST	0	0	0	0	0	0	0	0	0	0
RESPIRATORY FATIGUE	0	0	0	0	0	0	0	0	0	0
SLEEP APNOEA SYNDROME	0	0	0	0	0	0	0	0	0	0
COUGHING AND ASSOCIATED SYMPTOMS										
COUGH	10	3	0	5	2	8	3	0	4	1
PRODUCTIVE COUGH	0	0	0	0	0	0	0	0	0	0
RESPIRATORY TRACT DISORDERS NEC										
RESPIRATORY TRACT IRRITATION	0	0	0	0	0	0	0	0	0	0
RESPIRATORY TRACT SIGNS AND SYMPTOMS										
LOWER RESPIRATORY TRACT SIGNS AND SYMPTOMS										
PULMONARY PAIN	0	0	0	0	0	0	0	0	0	0

RESPIRATORY SIGNS AND SYMPTOMS NEC										
RESPIRATORY SYMPTOM	0	0	0	0	0	0	0	0	0	0
UPPER RESPIRATORY TRACT SIGNS AND SYMPTOMS										
APHONIA	0	0	0	0	0	0	0	0	0	0
CATARRH	2	0	0	2	0	1	0	0	1	0
DRY THROAT	0	0	0	0	0	0	0	0	0	0
DYSPHONIA	0	0	0	0	0	0	0	0	0	0
INCREASED UPPER AIRWAY SECRETION	0	0	0	0	0	0	0	0	0	0
INCREASED VISCOSITY OF UPPER RESPIRATORY SE(	0	0	0	0	0	0	0	0	0	0
NASAL DISCOMFORT	0	0	0	0	0	0	0	0	0	0
OROPHARYNGEAL DISCOMFORT	0	0	0	0	0	0	0	0	0	0
OROPHARYNGEAL PAIN	13	6	1	4	2	13	6	1	4	2
PARANASAL SINUS DISCOMFORT	0	0	0	0	0	0	0	0	0	0
RHINALGIA	0	0	0	0	0	0	0	0	0	0
RHINORRHOEA	6	0	0	5	1	5	0	0	5	0
SINUS PAIN	2	1	1	0	0	2	1	1	0	0
SNEEZING	1	0	0	1	0	1	0	0	1	0
THROAT CLEARING	0	0	0	0	0	0	0	0	0	0
THROAT IRRITATION	1	1	0	0	0	1	1	0	0	0
THROAT TIGHTNESS	0	0	0	0	0	0	0	0	0	0
UPPER-AIRWAY COUGH SYNDROME	0	0	0	0	0	0	0	0	0	0
YAWNING	0	0	0	0	0	0	0	0	0	0
UPPER RESPIRATORY TRACT DISORDERS (EXCL INFECTIONS)										
NASAL CONGESTION AND INFLAMMATIONS										
NASAL CONGESTION	2	0	1	1	0	2	0	1	1	0
RHINITIS ALLERGIC	0	0	0	0	0	0	0	0	0	0
RHINITIS ATROPHIC	0	0	0	0	0	0	0	0	0	0
NASAL DISORDERS NEC										
EPISTAXIS	1	1	0	0	0	1	1	0	0	0
NASAL DRYNESS	0	0	0	0	0	0	0	0	0	0
PARANASAL SINUS DISORDERS (EXCL INFECTIONS AND NEOPLASMS)										
SINUS CONGESTION	0	0	0	0	0	0	0	0	0	0
PHARYNGEAL DISORDERS (EXCL INFECTIONS AND NEOPLASMS)										
PHARYNGEAL SWELLING	1	0	1	0	0	1	0	1	0	0
PHARYNGEAL ULCERATION	0	0	0	0	0	0	0	0	0	0

TONSILLAR ERYTHEMA	0	0	0	0	0	0	0	0	0	0
TRACHEAL DISORDERS (EXCL INFECTIONS AND NEOPLASMS)										
TRACHEAL PAIN	0	0	0	0	0	0	0	0	0	0
**SKIN AND SUBCUTANEOUS TISSUE DISORDERS**										
ANGIOEDEMA AND URTICARIA										
ANGIOEDEMAS										
ANGIOEDEMA	0	0	0	0	0	0	0	0	0	0
URTICARIAS										
COLD URTICARIA	0	0	0	0	0	0	0	0	0	0
SOLAR URTICARIA	0	0	0	0	0	0	0	0	0	0
URTICARIA	2	0	0	2	0	2	0	0	2	0
URTICARIA CHRONIC	0	0	0	0	0	0	0	0	0	0
CORNIFICATION AND DYSTROPHIC SKIN DISORDERS										
SKIN DYSTROPHIES										
HYPERTROPHIC SCAR	0	0	0	0	0	0	0	0	0	0
CUTANEOUS NEOPLASMS BENIGN										
SKIN CYSTS AND POLYPS										
DERMAL CYST	0	0	0	0	0	0	0	0	0	0
EPIDERMAL AND DERMAL CONDITIONS										
BULLOUS CONDITIONS										
BLISTER	0	0	0	0	0	0	0	0	0	0
BLOOD BLISTER	0	0	0	0	0	0	0	0	0	0
PEMPHIGOID	0	0	0	0	0	0	0	0	0	0
TOXIC EPIDERMAL NECROLYSIS	0	0	0	0	0	0	0	0	0	0
DERMAL AND EPIDERMAL CONDITIONS NEC										
DRY SKIN	1	0	0	1	0	1	0	0	1	0
PAIN OF SKIN	2	0	0	1	1	1	0	0	1	0
SENSITIVE SKIN	1	1	0	0	0	1	1	0	0	0
SKIN BURNING SENSATION	0	0	0	0	0	0	0	0	0	0
SKIN DISCOLOURATION	0	0	0	0	0	0	0	0	0	0
SKIN FRAGILITY	0	0	0	0	0	0	0	0	0	0
SKIN LESION	0	0	0	0	0	0	0	0	0	0
SKIN ODOUR ABNORMAL	0	0	0	0	0	0	0	0	0	0
SKIN REACTION	1	1	0	0	0	1	1	0	0	0
SKIN SENSITISATION	0	0	0	0	0	0	0	0	0	0

SKIN WARM	2	0	1	1	0	1	0	0	1	0
DERMATITIS AND ECZEMA										
DERMATITIS	0	0	0	0	0	0	0	0	0	0
DERMATITIS ALLERGIC	0	0	0	0	0	0	0	0	0	0
DERMATITIS ATOPIC	0	0	0	0	0	0	0	0	0	0
DERMATITIS CONTACT	0	0	0	0	0	0	0	0	0	0
ECZEMA	0	0	0	0	0	0	0	0	0	0
ECZEMA ASTEATOTIC	0	0	0	0	0	0	0	0	0	0
NEURODERMATITIS	0	0	0	0	0	0	0	0	0	0
SEBORRHOEIC DERMATITIS	0	0	0	0	0	0	0	0	0	0
SKIN IRRITATION	0	0	0	0	0	0	0	0	0	0
ERYTHEMAS										
ERYTHEMA	4	1	0	2	1	3	1	0	2	0
EXFOLIATIVE CONDITIONS										
SKIN EXFOLIATION	1	0	0	0	1	0	0	0	0	0
PAPULOSQUAMOUS CONDITIONS										
LICHEN PLANUS	0	0	0	0	0	0	0	0	0	0
PITYRIASIS ROSEA	0	0	0	0	0	0	0	0	0	0
PHOTOSENSITIVITY AND PHOTODERMATOSIS CONDITIONS										
PHOTOSENSITIVITY REACTION	0	0	0	0	0	0	0	0	0	0
PRURITUS NEC										
PRURITUS	11	4	3	2	2	11	4	3	2	2
PSORIATIC CONDITIONS										
PSORIASIS	1	0	1	0	0	1	0	1	0	0
RASHES, ERUPTIONS AND EXANTHEMS NEC										
RASH	33	15	4	12	2	26	15	1	8	2
RASH ERYTHEMATOUS	5	3	0	2	0	5	3	0	2	0
RASH MACULAR	1	1	0	0	0	1	1	0	0	0
RASH MORBILLIFORM	0	0	0	0	0	0	0	0	0	0
RASH PAPULAR	1	1	0	0	0	1	1	0	0	0
RASH PRURITIC	6	1	0	4	1	6	1	0	4	1
SKIN INJURIES AND MECHANICAL DERMATOSES										
DECUBITUS ULCER	0	0	0	0	0	0	0	0	0	0
NEEDLE TRACK MARKS	0	0	0	0	0	0	0	0	0	0
SKIN AND SUBCUTANEOUS TISSUE DISORDERS NEC										

SKIN AND SUBCUTANEOUS TISSUE ULCERATIONS										
SKIN EROSION	0	0	0	0	0	0	0	0	0	0
SKIN APPENDAGE CONDITIONS										
ACNES										
ACNE	0	0	0	0	0	0	0	0	0	0
ACNE CYSTIC	0	0	0	0	0	0	0	0	0	0
ALOPECIAS										
ALOPECIA	1	0	0	1	0	1	0	0	1	0
APOCRINE AND ECCRINE GLAND DISORDERS										
COLD SWEAT	5	3	1	1	0	5	3	1	1	0
HYPERHIDROSIS	10	1	4	4	1	9	1	3	4	1
MILIARIA	0	0	0	0	0	0	0	0	0	0
NIGHT SWEATS	5	1	2	2	0	5	1	2	2	0
HYPERTRICHOSES										
HIRSUTISM	0	0	0	0	0	0	0	0	0	0
NAIL AND NAIL BED CONDITIONS (EXCL INFECTIONS AND INFESTATIONS)										
NAIL DISCOLOURATION	0	0	0	0	0	0	0	0	0	0
ONYCHOCLASIS	0	0	0	0	0	0	0	0	0	0
PILAR DISORDERS NEC										
PILOERECTION	0	0	0	0	0	0	0	0	0	0
SKIN VASCULAR ABNORMALITIES										
PURPURA AND RELATED CONDITIONS										
PETECHIAE	0	0	0	0	0	0	0	0	0	0
PURPURA	1	0	0	0	1	1	0	0	0	1
SKIN HAEMORRHAGES										
SKIN HAEMORRHAGE	0	0	0	0	0	0	0	0	0	0
**SOCIAL CIRCUMSTANCES**										
ECONOMIC AND HOUSING ISSUES										
EMPLOYMENT ISSUES										
RETIREMENT	0	0	0	0	0	0	0	0	0	0
ENVIRONMENTAL ISSUES										
NON-OCCUPATIONAL AND UNSPECIFIED ENVIRONMENTAL PROBLEMS										
POLLUTION	0	0	0	0	0	0	0	0	0	0
**SURGICAL AND MEDICAL PROCEDURES**										
BONE AND JOINT THERAPEUTIC PROCEDURES										
JOINT THERAPEUTIC PROCEDURES										

KNEE OPERATION	0	0	0	0	0	0	0	0	0	0
BREAST THERAPEUTIC PROCEDURES										
MASTECTOMIES										
BREAST CONSERVING SURGERY	0	0	0	0	0	0	0	0	0	0
GASTROINTESTINAL THERAPEUTIC PROCEDURES										
GASTROINTESTINAL THERAPEUTIC PROCEDURES NEC										
PROPHYLAXIS OF NAUSEA AND VOMITING	0	0	0	0	0	0	0	0	0	0
HEAD AND NECK THERAPEUTIC PROCEDURES										
DENTAL AND GINGIVAL THERAPEUTIC PROCEDURES										
DENTAL CARE	0	0	0	0	0	0	0	0	0	0
NERVOUS SYSTEM, SKULL AND SPINE THERAPEUTIC PROCEDURES										
SKULL AND BRAIN THERAPEUTIC PROCEDURES										
BRAIN TUMOUR OPERATION	0	0	0	0	0	0	0	0	0	0
OBSTETRIC AND GYNAECOLOGICAL THERAPEUTIC PROCEDURES										
FERTILITY AND FERTILISATION INTERVENTIONS FEMALE										
ENDOMETRIAL SCRATCHING	0	0	0	0	0	0	0	0	0	0
RESPIRATORY TRACT THERAPEUTIC PROCEDURES										
RESPIRATORY TRACT THERAPEUTIC PROCEDURES NEC										
OXYGEN THERAPY	0	0	0	0	0	0	0	0	0	0
THERAPEUTIC PROCEDURES AND SUPPORTIVE CARE NEC										
ANAESTHESIA AND ALLIED PROCEDURES										
NERVE BLOCK	0	0	0	0	0	0	0	0	0	0
DIETARY AND NUTRITIONAL THERAPIES										
NOTHING BY MOUTH ORDER	0	0	0	0	0	0	0	0	0	0
IMMUNISATIONS										
COVID-19 IMMUNISATION	0	0	0	0	0	0	0	0	0	0
THERAPEUTIC PROCEDURES NEC										
INJECTION	0	0	0	0	0	0	0	0	0	0
LOCALISED ALTERNATING HOT AND COLD THERAPY	0	0	0	0	0	0	0	0	0	0
MASS EXCISION	0	0	0	0	0	0	0	0	0	0
REINFUSION	1	0	0	1	0	1	0	0	1	0
**VASCULAR DISORDERS**										
ARTERIOSCLEROSIS, STENOSIS, VASCULAR INSUFFICIENCY AND NECROSIS										
NON-SITE SPECIFIC NECROSIS AND VASCULAR INSUFFICIENCY NEC										
VASOSPASM	0	0	0	0	0	0	0	0	0	0
PERIPHERAL VASOCONSTRICTION, NECROSIS AND VASCULAR INSUFFICIENCY										

PERIPHERAL COLDNESS	2	0	0	2	0	2	0	0	2	0
RAYNAUD'S PHENOMENON	0	0	0	0	0	0	0	0	0	0
DECREASED AND NONSPECIFIC BLOOD PRESSURE DISORDERS AND SHOCK										
BLOOD PRESSURE DISORDERS NEC										
BLOOD PRESSURE FLUCTUATION	0	0	0	0	0	0	0	0	0	0
VASCULAR HYPOTENSIVE DISORDERS										
CAPILLARY LEAK SYNDROME	0	0	0	0	0	0	0	0	0	0
HYPOTENSION	1	0	0	1	0	0	0	0	0	0
ORTHOSTATIC HYPOTENSION	0	0	0	0	0	0	0	0	0	0
EMBOLISM AND THROMBOSIS										
NON-SITE SPECIFIC EMBOLISM AND THROMBOSIS										
EMBOLISM	1	1	0	0	0	1	1	0	0	0
THROMBOSIS	0	0	0	0	0	0	0	0	0	0
VENOUS THROMBOSIS	0	0	0	0	0	0	0	0	0	0
PERIPHERAL EMBOLISM AND THROMBOSIS										
BLUE TOE SYNDROME	0	0	0	0	0	0	0	0	0	0
DEEP VEIN THROMBOSIS	0	0	0	0	0	0	0	0	0	0
SUPERFICIAL VEIN THROMBOSIS	0	0	0	0	0	0	0	0	0	0
LYMPHATIC VESSEL DISORDERS										
LYMPHOEDEMAS										
LYMPHOEDEMA	0	0	0	0	0	0	0	0	0	0
VASCULAR DISORDERS NEC										
NON-SITE SPECIFIC VASCULAR DISORDERS NEC										
VASCULAR PAIN	0	0	0	0	0	0	0	0	0	0
VEIN DISCOLOURATION	0	0	0	0	0	0	0	0	0	0
VEIN RUPTURE	0	0	0	0	0	0	0	0	0	0
PERIPHERAL VASCULAR DISORDERS NEC										
FLUSHING	0	0	0	0	0	0	0	0	0	0
HOT FLUSH	4	1	0	3	0	2	1	0	1	0
SITE SPECIFIC VASCULAR DISORDERS NEC										
PALLOR	1	0	1	0	0	0	0	0	0	0
VASCULAR HAEMORRHAGIC DISORDERS										
HAEMORRHAGES NEC										
HAEMATOMA	0	0	0	0	0	0	0	0	0	0
HAEMORRHAGE	0	0	0	0	0	0	0	0	0	0
VASCULAR HYPERTENSIVE DISORDERS										

ACCELERATED AND MALIGNANT HYPERTENSION										
HYPERTENSIVE URGENCY	0	0	0	0	0	0	0	0	0	0
VASCULAR HYPERTENSIVE DISORDERS NEC										
HYPERTENSION	0	0	0	0	0	0	0	0	0	0
SYSTOLIC HYPERTENSION	0	0	0	0	0	0	0	0	0	0
VASCULAR INFECTIONS AND INFLAMMATIONS										
ARTERIAL INFECTIONS AND INFLAMMATIONS										
GIANT CELL ARTERITIS	0	0	0	0	0	0	0	0	0	0
PHLEBITIS NEC										
PHLEBITIS	0	0	0	0	0	0	0	0	0	0
VASCULITIDES NEC										
VASCULITIS	1	0	0	0	1	1	0	0	0	1
VENOUS VARICES										
VARICOSE VEINS NEC										
SPIDER VEIN	0	0	0	0	0	0	0	0	0	0
VARICOSE VEIN	0	0	0	0	0	0	0	0	0	0
**TOTAL ADR EVENTS**	***2581***	***623***	***367***	***1227***	***364***	***2230***	***623***	***281***	***1034***	***292***

SUPPLEMENTARY TABLE 19. Others/Unknown COVID-19 vaccine: ADR listings for events reported in the YCVM in (a) those reporting any vaccination dose and (b) in those who had reported a 1st dose vaccination and any

	Individuals with any vaccination dose: ADR Counts					Individuals reporting a 1st dose: ADR Counts				
**MEDDRA REACTION TERM (SOC, *HLGT*, *HLT*, PT)**	All doses	1st dose	2nd dose	3rd dose	Other doses	All doses	1st dose	2nd dose	3rd dose	Other doses
(freetext)	2	2	0	0	0	2	2	0	0	0
**BLOOD AND LYMPHATIC SYSTEM DISORDERS**										
COAGULOPATHIES AND BLEEDING DIATHESES (EXCL THROMBOCYTOPENIC)										
BLEEDING TENDENCIES										
INCREASED TENDENCY TO BRUISE	0	0	0	0	0	0	0	0	0	0
HAEMOGLOBINOPATHIES										
SICKLE CELL TRAIT AND DISORDERS										
SICKLE CELL ANAEMIA WITH CRISIS	0	0	0	0	0	0	0	0	0	0
PLATELET DISORDERS										
THROMBOCYTOPENIAS										
IMMUNE THROMBOCYTOPENIA	0	0	0	0	0	0	0	0	0	0
SPLEEN, LYMPHATIC AND RETICULOENDOTHELIAL SYSTEM DISORDERS										
LYMPHATIC SYSTEM DISORDERS NEC										
LYMPH NODE PAIN	0	0	0	0	0	0	0	0	0	0
LYMPHADENITIS	0	0	0	0	0	0	0	0	0	0
LYMPHADENOPATHY	0	0	0	0	0	0	0	0	0	0
**CARDIAC DISORDERS**										
CARDIAC ARRHYTHMIAS										
RATE AND RHYTHM DISORDERS NEC										
ARRHYTHMIA	0	0	0	0	0	0	0	0	0	0
CARDIAC FLUTTER	0	0	0	0	0	0	0	0	0	0
EXTRASYSTOLES	0	0	0	0	0	0	0	0	0	0
TACHYCARDIA	0	0	0	0	0	0	0	0	0	0
SUPRAVENTRICULAR ARRHYTHMIAS										
ATRIAL FIBRILLATION	0	0	0	0	0	0	0	0	0	0
SUPRAVENTRICULAR TACHYCARDIA	0	0	0	0	0	0	0	0	0	0

CARDIAC DISORDERS, SIGNS AND SYMPTOMS NEC										
CARDIAC DISORDERS NEC										
CARDIOVASCULAR DISORDER	0	0	0	0	0	0	0	0	0	0
CARDIAC SIGNS AND SYMPTOMS NEC										
PALPITATIONS	1	1	0	0	0	1	1	0	0	0
CORONARY ARTERY DISORDERS										
ISCHAEMIC CORONARY ARTERY DISORDERS										
ANGINA PECTORIS	0	0	0	0	0	0	0	0	0	0
MYOCARDIAL INFARCTION	0	0	0	0	0	0	0	0	0	0
HEART FAILURES										
HEART FAILURES NEC										
CARDIAC FAILURE	0	0	0	0	0	0	0	0	0	0
MYOCARDIAL DISORDERS										
NONINFECTIOUS MYOCARDITIS										
MYOCARDITIS	0	0	0	0	0	0	0	0	0	0
PERICARDIAL DISORDERS										
NONINFECTIOUS PERICARDITIS										
PERICARDITIS	0	0	0	0	0	0	0	0	0	0
**CONGENITAL, FAMILIAL AND GENETIC DISORDERS**										
CARDIAC AND VASCULAR DISORDERS CONGENITAL										
CARDIAC DISORDERS CONGENITAL NEC										
HEART DISEASE CONGENITAL	0	0	0	0	0	0	0	0	0	0
METABOLIC AND NUTRITIONAL DISORDERS CONGENITAL										
INBORN ERRORS OF AMINO ACID METABOLISM										
HYPERGLYCINAEMIA	0	0	0	0	0	0	0	0	0	0
INBORN ERRORS OF STEROID SYNTHESIS										
11-BETA-HYDROXYLASE DEFICIENCY	1	1	0	0	0	1	1	0	0	0
NEUROLOGICAL DISORDERS CONGENITAL										
PERIPHERAL NERVOUS SYSTEM DISORDERS CONGENITAL NEC										
PAROXYSMAL EXTREME PAIN DISORDER	0	0	0	0	0	0	0	0	0	0
**EAR AND LABYRINTH DISORDERS**										

AURAL DISORDERS NEC										
EAR DISORDERS NEC										
EAR DISCOMFORT	0	0	0	0	0	0	0	0	0	0
EAR DISORDER	0	0	0	0	0	0	0	0	0	0
EAR PAIN	0	0	0	0	0	0	0	0	0	0
EAR SWELLING	0	0	0	0	0	0	0	0	0	0
EXTERNAL EAR DISORDERS (EXCL CONGENITAL)										
EXTERNAL EAR DISORDERS NEC										
EXCESSIVE CERUMEN PRODUCTION	0	0	0	0	0	0	0	0	0	0
HEARING DISORDERS										
HEARING LOSSES										
DEAFNESS	0	0	0	0	0	0	0	0	0	0
DEAFNESS NEUROSENSORY	0	0	0	0	0	0	0	0	0	0
DEAFNESS PERMANENT	0	0	0	0	0	0	0	0	0	0
DEAFNESS UNILATERAL	0	0	0	0	0	0	0	0	0	0
HYPOACUSIS	0	0	0	0	0	0	0	0	0	0
SUDDEN HEARING LOSS	0	0	0	0	0	0	0	0	0	0
HYPERACUSIA										
HYPERACUSIS	0	0	0	0	0	0	0	0	0	0
INNER EAR AND VIIITH CRANIAL NERVE DISORDERS										
INNER EAR SIGNS AND SYMPTOMS										
MOTION SICKNESS	0	0	0	0	0	0	0	0	0	0
TINNITUS	0	0	0	0	0	0	0	0	0	0
VERTIGO	1	0	0	1	0	1	0	0	1	0
VERTIGO LABYRINTHINE	0	0	0	0	0	0	0	0	0	0
VERTIGO POSITIONAL	0	0	0	0	0	0	0	0	0	0
**ENDOCRINE DISORDERS**										
THYROID GLAND DISORDERS										
THYROID HYPERFUNCTION DISORDERS										
HYPERTHYROIDISM	0	0	0	0	0	0	0	0	0	0
**EYE DISORDERS**										
EYE DISORDERS NEC										
LACRIMATION DISORDERS										
DRY EYE	0	0	0	0	0	0	0	0	0	0
LACRIMATION INCREASED	0	0	0	0	0	0	0	0	0	0
OCULAR DISORDERS NEC										

EYE OEDEMA	0	0	0	0	0	0	0	0	0	0
EYE PAIN	0	0	0	0	0	0	0	0	0	0
EYE SWELLING	0	0	0	0	0	0	0	0	0	0
EYE ULCER	0	0	0	0	0	0	0	0	0	0
EYELID PAIN	0	0	0	0	0	0	0	0	0	0
OCULAR DISCOMFORT	0	0	0	0	0	0	0	0	0	0
PERIORBITAL DISCOMFORT	0	0	0	0	0	0	0	0	0	0
PERIORBITAL SWELLING	0	0	0	0	0	0	0	0	0	0
OCULAR HAEMORRHAGES AND VASCULAR DISORDERS NEC										
CONJUNCTIVAL AND CORNEAL BLEEDING AND VASCULAR DISORDERS										
CONJUNCTIVAL HAEMORRHAGE	0	0	0	0	0	0	0	0	0	0
LID BLEEDING AND VASCULAR DISORDERS										
EYELID BLEEDING	0	0	0	0	0	0	0	0	0	0
OCULAR INFECTIONS, IRRITATIONS AND INFLAMMATIONS										
LID, LASH AND LACRIMAL INFECTIONS, IRRITATIONS AND INFLAMMATIONS										
BLEPHARITIS	0	0	0	0	0	0	0	0	0	0
ERYTHEMA OF EYELID	0	0	0	0	0	0	0	0	0	0
EYELID IRRITATION	0	0	0	0	0	0	0	0	0	0
OCULAR INFECTIONS, INFLAMMATIONS AND ASSOCIATED MANIFESTATIONS										
EYE DISCHARGE	0	0	0	0	0	0	0	0	0	0
EYE IRRITATION	0	0	0	0	0	0	0	0	0	0
EYE PRURITUS	0	0	0	0	0	0	0	0	0	0
LIMBAL SWELLING	0	0	0	0	0	0	0	0	0	0
OCULAR HYPERAEMIA	0	0	0	0	0	0	0	0	0	0
OCULAR NEUROMUSCULAR DISORDERS										
EYELID MOVEMENT DISORDERS										
BLEPHAROSPASM	0	0	0	0	0	0	0	0	0	0
OCULAR SENSORY SYMPTOMS NEC										
OCULAR SENSATION DISORDERS										
ABNORMAL SENSATION IN EYE	0	0	0	0	0	0	0	0	0	0
ASTHENOPIA	0	0	0	0	0	0	0	0	0	0
FOREIGN BODY SENSATION IN EYES	0	0	0	0	0	0	0	0	0	0
PHOTOPHOBIA	0	0	0	0	0	0	0	0	0	0

OCULAR STRUCTURAL CHANGE, DEPOSIT AND DEGENERATION NEC										
CHOROID AND VITREOUS STRUCTURAL CHANGE, DEPOSIT AND DEGENERATION										
VITREOUS DETACHMENT	0	0	0	0	0	0	0	0	0	0
VITREOUS FLOATERS	0	0	0	0	0	0	0	0	0	0
RETINA, CHOROID AND VITREOUS HAEMORRHAGES AND VASCULAR DISORDERS										
RETINAL BLEEDING AND VASCULAR DISORDERS (EXCL RETINOPATHY)										
RETINAL VEIN OCCLUSION	0	0	0	0	0	0	0	0	0	0
RETINOPATHIES NEC										
RETINAL EXUDATES	0	0	0	0	0	0	0	0	0	0
VISION DISORDERS										
VISUAL DISORDERS NEC										
DIPLOPIA	0	0	0	0	0	0	0	0	0	0
HALO VISION	0	0	0	0	0	0	0	0	0	0
METAMORPHOPSIA	0	0	0	0	0	0	0	0	0	0
PHOTOPSIA	0	0	0	0	0	0	0	0	0	0
VISION BLURRED	0	0	0	0	0	0	0	0	0	0
VISUAL IMPAIRMENT AND BLINDNESS (EXCL COLOUR BLINDNESS)										
BLINDNESS	0	0	0	0	0	0	0	0	0	0
BLINDNESS TRANSIENT	0	0	0	0	0	0	0	0	0	0
SUDDEN VISUAL LOSS	0	0	0	0	0	0	0	0	0	0
VISUAL IMPAIRMENT	0	0	0	0	0	0	0	0	0	0
**GASTROINTESTINAL DISORDERS**										
ANAL AND RECTAL CONDITIONS NEC										
ANAL AND RECTAL SIGNS AND SYMPTOMS										
ANAL PARAESTHESIA	0	0	0	0	0	0	0	0	0	0
BENIGN NEOPLASMS GASTROINTESTINAL										
BENIGN ORAL CAVITY NEOPLASMS										
MOUTH CYST	0	0	0	0	0	0	0	0	0	0
DENTAL AND GINGIVAL CONDITIONS										
DENTAL DISORDERS NEC										
TEETHING	0	0	0	0	0	0	0	0	0	0

DENTAL PAIN AND SENSATION DISORDERS										
DENTAL PARAESTHESIA	0	0	0	0	0	0	0	0	0	0
TOOTHACHE	0	0	0	0	0	0	0	0	0	0
GINGIVAL DISORDERS, SIGNS AND SYMPTOMS NEC										
GINGIVAL BLISTER	0	0	0	0	0	0	0	0	0	0
GINGIVAL PAIN	0	0	0	0	0	0	0	0	0	0
GINGIVAL SWELLING	0	0	0	0	0	0	0	0	0	0
GASTROINTESTINAL CONDITIONS NEC										
GASTROINTESTINAL MUCOSAL DYSTROPHIES AND SECRETION DISORDERS										
BARRETT'S OESOPHAGUS	0	0	0	0	0	0	0	0	0	0
GASTROINTESTINAL HAEMORRHAGES NEC										
INTESTINAL HAEMORRHAGES										
RECTAL HAEMORRHAGE	0	0	0	0	0	0	0	0	0	0
NON-SITE SPECIFIC GASTROINTESTINAL HAEMORRHAGES										
HAEMATEMESIS	0	0	0	0	0	0	0	0	0	0
GASTROINTESTINAL INFLAMMATORY CONDITIONS										
COLITIS (EXCL INFECTIVE)										
COLITIS	0	0	0	0	0	0	0	0	0	0
COLITIS MICROSCOPIC	0	0	0	0	0	0	0	0	0	0
COLITIS ULCERATIVE	0	0	0	0	0	0	0	0	0	0
GASTRITIS (EXCL INFECTIVE)										
GASTRITIS	0	0	0	0	0	0	0	0	0	0
REFLUX GASTRITIS	0	0	0	0	0	0	0	0	0	0
GASTROINTESTINAL INFLAMMATORY DISORDERS NEC										
GASTROINTESTINAL TRACT IRRITATION	0	0	0	0	0	0	0	0	0	0
GASTROINTESTINAL MOTILITY AND DEFAECATION CONDITIONS										
DIARRHOEA (EXCL INFECTIVE)										
DIARRHOEA	2	2	0	0	0	2	2	0	0	0
GASTROINTESTINAL ATONIC AND HYPOMOTILITY DISORDERS NEC										
CONSTIPATION	1	1	0	0	0	1	1	0	0	0
GASTROOESOPHAGEAL REFLUX DISEASE	0	0	0	0	0	0	0	0	0	0
GASTROINTESTINAL DYSKINETIC DISORDERS										
CHANGE OF BOWEL HABIT	0	0	0	0	0	0	0	0	0	0
GASTROINTESTINAL SPASTIC AND HYPERMOTILITY DISORDERS										
IRRITABLE BOWEL SYNDROME	0	0	0	0	0	0	0	0	0	0
GASTROINTESTINAL SIGNS AND SYMPTOMS										

DYSPEPTIC SIGNS AND SYMPTOMS										
DYSPEPSIA	1	1	0	0	0	1	1	0	0	0
ERUCTATION	0	0	0	0	0	0	0	0	0	0
FAECAL ABNORMALITIES NEC										
FAECALOMA	0	0	0	0	0	0	0	0	0	0
FAECES DISCOLOURED	0	0	0	0	0	0	0	0	0	0
FAECES SOFT	0	0	0	0	0	0	0	0	0	0
FLATULENCE, BLOATING AND DISTENSION										
ABDOMINAL DISTENSION	0	0	0	0	0	0	0	0	0	0
FLATULENCE	1	1	0	0	0	1	1	0	0	0
GASTROINTESTINAL AND ABDOMINAL PAINS (EXCL ORAL AND THROAT)										
ABDOMINAL PAIN	2	2	0	0	0	2	2	0	0	0
ABDOMINAL PAIN LOWER	0	0	0	0	0	0	0	0	0	0
ABDOMINAL PAIN UPPER	0	0	0	0	0	0	0	0	0	0
GASTROINTESTINAL PAIN	0	0	0	0	0	0	0	0	0	0
GASTROINTESTINAL SIGNS AND SYMPTOMS NEC										
ABDOMINAL DISCOMFORT	0	0	0	0	0	0	0	0	0	0
ABDOMINAL SYMPTOM	0	0	0	0	0	0	0	0	0	0
ACUTE ABDOMEN	0	0	0	0	0	0	0	0	0	0
ANAL INCONTINENCE	0	0	0	0	0	0	0	0	0	0
BREATH ODOUR	0	0	0	0	0	0	0	0	0	0
DYSPHAGIA	0	0	0	0	0	0	0	0	0	0
ODYNOPHAGIA	0	0	0	0	0	0	0	0	0	0
NAUSEA AND VOMITING SYMPTOMS										
NAUSEA	9	7	0	2	0	9	7	0	2	0
RETCHING	0	0	0	0	0	0	0	0	0	0
VOMITING	2	1	0	0	1	2	1	0	0	1
VOMITING PROJECTILE	0	0	0	0	0	0	0	0	0	0
MALABSORPTION CONDITIONS										
MALABSORPTION SYNDROMES										
COELIAC DISEASE	0	0	0	0	0	0	0	0	0	0
ORAL SOFT TISSUE CONDITIONS										
ORAL SOFT TISSUE DISORDERS NEC										
CHEILITIS	0	0	0	0	0	0	0	0	0	0
LIP BLISTER	0	0	0	0	0	0	0	0	0	0

ORAL LICHEN PLANUS	0	0	0	0	0	0	0	0	0	0
ORAL SOFT TISSUE INFECTIONS										
ANGULAR CHEILITIS	0	0	0	0	0	0	0	0	0	0
ORAL SOFT TISSUE SIGNS AND SYMPTOMS										
HYPOAESTHESIA ORAL	1	1	0	0	0	1	1	0	0	0
LIP PAIN	0	0	0	0	0	0	0	0	0	0
ORAL DISCOMFORT	0	0	0	0	0	0	0	0	0	0
ORAL MUCOSAL ROUGHENING	0	0	0	0	0	0	0	0	0	0
ORAL PAIN	0	0	0	0	0	0	0	0	0	0
PARAESTHESIA ORAL	0	0	0	0	0	0	0	0	0	0
LIP SWELLING	0	0	0	0	0	0	0	0	0	0
MOUTH SWELLING	0	0	0	0	0	0	0	0	0	0
STOMATITIS AND ULCERATION										
APHTHOUS ULCER	0	0	0	0	0	0	0	0	0	0
LIP ULCERATION	0	0	0	0	0	0	0	0	0	0
MOUTH ULCERATION	0	0	0	0	0	0	0	0	0	0
STOMATITIS	0	0	0	0	0	0	0	0	0	0
SALIVARY GLAND CONDITIONS										
ORAL DRYNESS AND SALIVA ALTERED										
DRY MOUTH	0	0	0	0	0	0	0	0	0	0
LIP DRY	0	0	0	0	0	0	0	0	0	0
SALIVARY HYPOSECRETION	0	0	0	0	0	0	0	0	0	0
TONGUE CONDITIONS										
TONGUE SIGNS AND SYMPTOMS										
GLOSSODYNIA	0	0	0	0	0	0	0	0	0	0
SWOLLEN TONGUE	0	0	0	0	0	0	0	0	0	0
TONGUE COATED	0	0	0	0	0	0	0	0	0	0
TONGUE DISCOMFORT	0	0	0	0	0	0	0	0	0	0
TONGUE OEDEMA	0	0	0	0	0	0	0	0	0	0
TONGUE SPASM	0	0	0	0	0	0	0	0	0	0
**GENERAL DISORDERS AND ADMINISTRATION SITE CONDITIONS**										
ADMINISTRATION SITE REACTIONS										
ADMINISTRATION SITE REACTIONS NEC										
ADMINISTRATION SITE BRUISE	0	0	0	0	0	0	0	0	0	0
ADMINISTRATION SITE PAIN	0	0	0	0	0	0	0	0	0	0

PUNCTURE SITE BRUISE	0	0	0	0	0	0	0	0	0	0
PUNCTURE SITE PAIN	0	0	0	0	0	0	0	0	0	0
APPLICATION SITE REACTIONS										
APPLICATION SITE BRUISE	0	0	0	0	0	0	0	0	0	0
APPLICATION SITE ERYTHEMA	0	0	0	0	0	0	0	0	0	0
APPLICATION SITE PAIN	0	0	0	0	0	0	0	0	0	0
IMPLANT AND CATHETER SITE REACTIONS										
IMPLANT SITE PAIN	0	0	0	0	0	0	0	0	0	0
IMPLANT SITE WARMTH	0	0	0	0	0	0	0	0	0	0
INFUSION SITE REACTIONS										
INFUSION SITE PAIN	0	0	0	0	0	0	0	0	0	0
INFUSION SITE SCAB	0	0	0	0	0	0	0	0	0	0
INFUSION SITE WARMTH	0	0	0	0	0	0	0	0	0	0
INJECTION SITE REACTIONS										
INJECTION SITE BRUISING	1	1	0	0	0	1	1	0	0	0
INJECTION SITE DISCOMFORT	0	0	0	0	0	0	0	0	0	0
INJECTION SITE ERYTHEMA	0	0	0	0	0	0	0	0	0	0
INJECTION SITE HYPERSENSITIVITY	0	0	0	0	0	0	0	0	0	0
INJECTION SITE INFLAMMATION	0	0	0	0	0	0	0	0	0	0
INJECTION SITE INJURY	0	0	0	0	0	0	0	0	0	0
INJECTION SITE IRRITATION	0	0	0	0	0	0	0	0	0	0
INJECTION SITE JOINT PAIN	0	0	0	0	0	0	0	0	0	0
INJECTION SITE MASS	0	0	0	0	0	0	0	0	0	0
INJECTION SITE NODULE	0	0	0	0	0	0	0	0	0	0
INJECTION SITE OEDEMA	0	0	0	0	0	0	0	0	0	0
INJECTION SITE PAIN	3	3	0	0	0	3	3	0	0	0
INJECTION SITE PAPULE	0	0	0	0	0	0	0	0	0	0
INJECTION SITE PARAESTHESIA	0	0	0	0	0	0	0	0	0	0
INJECTION SITE PRURITUS	1	1	0	0	0	1	1	0	0	0
INJECTION SITE RASH	0	0	0	0	0	0	0	0	0	0
INJECTION SITE REACTION	0	0	0	0	0	0	0	0	0	0
INJECTION SITE SCAB	0	0	0	0	0	0	0	0	0	0
INJECTION SITE SWELLING	0	0	0	0	0	0	0	0	0	0
INJECTION SITE URTICARIA	0	0	0	0	0	0	0	0	0	0
INJECTION SITE WARMTH	0	0	0	0	0	0	0	0	0	0

INSTILLATION SITE REACTIONS										
INSTILLATION SITE PRURITUS	0	0	0	0	0	0	0	0	0	0
INSTILLATION SITE WARMTH	0	0	0	0	0	0	0	0	0	0
VACCINATION SITE REACTIONS										
SHOULDER INJURY RELATED TO VACCINE ADMINISTRATION	0	0	0	0	0	0	0	0	0	0
VACCINATION SITE BRUISING	0	0	0	0	0	0	0	0	0	0
VACCINATION SITE DISCOMFORT	0	0	0	0	0	0	0	0	0	0
VACCINATION SITE ERYTHEMA	0	0	0	0	0	0	0	0	0	0
VACCINATION SITE JOINT ERYTHEMA	0	0	0	0	0	0	0	0	0	0
VACCINATION SITE JOINT PAIN	0	0	0	0	0	0	0	0	0	0
VACCINATION SITE MASS	0	0	0	0	0	0	0	0	0	0
VACCINATION SITE PAIN	0	0	0	0	0	0	0	0	0	0
VACCINATION SITE RASH	0	0	0	0	0	0	0	0	0	0
VACCINATION SITE SWELLING	0	0	0	0	0	0	0	0	0	0
VACCINATION SITE WARMTH	0	0	0	0	0	0	0	0	0	0
BODY TEMPERATURE CONDITIONS										
BODY TEMPERATURE ALTERED										
HYPERTHERMIA	0	0	0	0	0	0	0	0	0	0
HYPOTHERMIA	0	0	0	0	0	0	0	0	0	0
FEBRILE DISORDERS										
PYREXIA	16	12	3	1	0	13	12	0	1	0
FATAL OUTCOMES										
DEATH AND SUDDEN DEATH										
DEATH	0	0	0	0	0	0	0	0	0	0
GENERAL SYSTEM DISORDERS NEC										
ADVERSE EFFECT ABSENT										
NO ADVERSE EVENT	0	0	0	0	0	0	0	0	0	0
ASTHENIC CONDITIONS										
ASTHENIA	3	3	0	0	0	3	3	0	0	0
CHRONIC FATIGUE SYNDROME	0	0	0	0	0	0	0	0	0	0
DECREASED ACTIVITY	0	0	0	0	0	0	0	0	0	0
FATIGUE	42	24	7	9	2	38	24	5	9	0
MALAISE	4	4	0	0	0	4	4	0	0	0
SLUGGISHNESS	0	0	0	0	0	0	0	0	0	0
FEELINGS AND SENSATIONS NEC										

CHILLS	10	8	0	2	0	10	8	0	2	0
FEELING ABNORMAL	1	1	0	0	0	1	1	0	0	0
FEELING COLD	4	2	1	1	0	4	2	1	1	0
FEELING HOT	2	1	0	1	0	2	1	0	1	0
FEELING JITTERY	0	0	0	0	0	0	0	0	0	0
FEELING OF BODY TEMPERATURE CHANGE	0	0	0	0	0	0	0	0	0	0
FEELING OF RELAXATION	0	0	0	0	0	0	0	0	0	0
HANGOVER	0	0	0	0	0	0	0	0	0	0
HUNGER	0	0	0	0	0	0	0	0	0	0
SENSATION OF BLOOD FLOW	0	0	0	0	0	0	0	0	0	0
THIRST	0	0	0	0	0	0	0	0	0	0
GAIT DISTURBANCES										
GAIT DISTURBANCE	1	0	1	0	0	1	0	1	0	0
GAIT INABILITY	0	0	0	0	0	0	0	0	0	0
GENERAL SIGNS AND SYMPTOMS NEC										
CRYING	0	0	0	0	0	0	0	0	0	0
ENERGY INCREASED	0	0	0	0	0	0	0	0	0	0
EXERCISE TOLERANCE DECREASED	0	0	0	0	0	0	0	0	0	0
GENERAL SYMPTOM	0	0	0	0	0	0	0	0	0	0
ILLNESS	2	1	0	1	0	1	1	0	0	0
INFLUENZA LIKE ILLNESS	8	6	0	2	0	8	6	0	2	0
LOCAL REACTION	0	0	0	0	0	0	0	0	0	0
PERIPHERAL SWELLING	1	1	0	0	0	1	1	0	0	0
SWELLING	2	1	0	1	0	2	1	0	1	0
SWELLING FACE	0	0	0	0	0	0	0	0	0	0
TISSUE IRRITATION	0	0	0	0	0	0	0	0	0	0
INFLAMMATIONS										
INFLAMMATION	1	0	1	0	0	1	0	1	0	0
SYSTEMIC INFLAMMATORY RESPONSE SYNDROME	0	0	0	0	0	0	0	0	0	0
OEDEMA NEC										
OEDEMA	0	0	0	0	0	0	0	0	0	0
OEDEMA PERIPHERAL	0	0	0	0	0	0	0	0	0	0
PAIN AND DISCOMFORT NEC										
AXILLARY PAIN	0	0	0	0	0	0	0	0	0	0

CHEST DISCOMFORT	0	0	0	0	0	0	0	0	0	0
CHEST PAIN	1	0	1	0	0	1	0	1	0	0
DISCOMFORT	0	0	0	0	0	0	0	0	0	0
FACIAL PAIN	0	0	0	0	0	0	0	0	0	0
HERNIA PAIN	0	0	0	0	0	0	0	0	0	0
INFLAMMATORY PAIN	0	0	0	0	0	0	0	0	0	0
NON-CARDIAC CHEST PAIN	0	0	0	0	0	0	0	0	0	0
PAIN	10	5	2	3	0	9	5	1	3	0
TENDERNESS	4	3	0	0	1	4	3	0	0	1
THERAPEUTIC AND NONTHERAPEUTIC EFFECTS (EXCL TOXICITY)										
THERAPEUTIC AND NONTHERAPEUTIC RESPONSES										
ADVERSE DRUG REACTION	2	2	0	0	0	2	2	0	0	0
ADVERSE EVENT	0	0	0	0	0	0	0	0	0	0
ADVERSE REACTION	0	0	0	0	0	0	0	0	0	0
IMMEDIATE POST-INJECTION REACTION	0	0	0	0	0	0	0	0	0	0
**HEPATOBILIARY DISORDERS**										
HEPATIC AND HEPATOBILIARY DISORDERS										
HEPATOBILIARY SIGNS AND SYMPTOMS										
HEPATIC PAIN	0	0	0	0	0	0	0	0	0	0
HEPATOCELLULAR DAMAGE AND HEPATITIS NEC										
LIVER INJURY	0	0	0	0	0	0	0	0	0	0
**IMMUNE SYSTEM DISORDERS**										
ALLERGIC CONDITIONS										
ALLERGIC CONDITIONS NEC										
HYPERSENSITIVITY	0	0	0	0	0	0	0	0	0	0
MULTIPLE ALLERGIES	0	0	0	0	0	0	0	0	0	0
ALLERGIES TO FOODS, FOOD ADDITIVES, DRUGS AND OTHER CHEMICALS										
ALLERGY TO CHEMICALS	0	0	0	0	0	0	0	0	0	0
ANAPHYLACTIC AND ANAPHYLACTOID RESPONSES										
ANAPHYLACTIC REACTION	0	0	0	0	0	0	0	0	0	0
ATOPIC DISORDERS										
SEASONAL ALLERGY	0	0	0	0	0	0	0	0	0	0
IMMUNE DISORDERS NEC										
IMMUNE AND ASSOCIATED CONDITIONS NEC										
BACILLE CALMETTE-GUERIN SCAR REACTIVATION	0	0	0	0	0	0	0	0	0	0

SENSITISATION	0	0	0	0	0	0	0	0	0	0
**INFECTIONS AND INFESTATIONS**										
BACTERIAL INFECTIOUS DISORDERS										
BACTERIAL INFECTIONS NEC										
CELLULITIS	0	0	0	0	0	0	0	0	0	0
PERIORBITAL CELLULITIS	0	0	0	0	0	0	0	0	0	0
HELICOBACTER INFECTIONS										
HELICOBACTER GASTRITIS	0	0	0	0	0	0	0	0	0	0
STAPHYLOCOCCAL INFECTIONS										
FURUNCLE	0	0	0	0	0	0	0	0	0	0
FUNGAL INFECTIOUS DISORDERS										
CANDIDA INFECTIONS										
CANDIDA INFECTION	0	0	0	0	0	0	0	0	0	0
VULVOVAGINAL CANDIDIASIS	0	0	0	0	0	0	0	0	0	0
INFECTIONS - PATHOGEN UNSPECIFIED										
ABDOMINAL AND GASTROINTESTINAL INFECTIONS										
APPENDICITIS	0	0	0	0	0	0	0	0	0	0
DIARRHOEA INFECTIOUS	0	0	0	0	0	0	0	0	0	0
GASTROINTESTINAL INFECTION	1	1	0	0	0	1	1	0	0	0
BREAST INFECTIONS										
MASTITIS	0	0	0	0	0	0	0	0	0	0
CENTRAL NERVOUS SYSTEM AND SPINAL INFECTIONS										
MYELITIS	0	0	0	0	0	0	0	0	0	0
DENTAL AND ORAL SOFT TISSUE INFECTIONS										
ABSCESS ORAL	0	0	0	0	0	0	0	0	0	0
ORAL PUSTULE	0	0	0	0	0	0	0	0	0	0
PERICORONITIS	0	0	0	0	0	0	0	0	0	0
TOOTH ABSCESS	0	0	0	0	0	0	0	0	0	0
EAR INFECTIONS										
EAR INFECTION	0	0	0	0	0	0	0	0	0	0
LABYRINTHITIS	0	0	0	0	0	0	0	0	0	0
OTITIS EXTERNA	0	0	0	0	0	0	0	0	0	0
EYE AND EYELID INFECTIONS										
CONJUNCTIVITIS	0	0	0	0	0	0	0	0	0	0
HORDEOLUM	0	0	0	0	0	0	0	0	0	0
INFECTIONS NEC										

ABSCESS	0	0	0	0	0	0	0	0	0	0
INFECTION	0	0	0	0	0	0	0	0	0	0
INJECTION SITE INFECTION	0	0	0	0	0	0	0	0	0	0
LOCALISED INFECTION	0	0	0	0	0	0	0	0	0	0
WOUND INFECTION	0	0	0	0	0	0	0	0	0	0
LOWER RESPIRATORY TRACT AND LUNG INFECTIONS										
LOWER RESPIRATORY TRACT INFECTION	0	0	0	0	0	0	0	0	0	0
PNEUMONIA	0	0	0	0	0	0	0	0	0	0
MALE REPRODUCTIVE TRACT INFECTIONS										
ORCHITIS	0	0	0	0	0	0	0	0	0	0
SEPSIS, BACTERAEMIA, VIRAEMIA AND FUNGAEMIA NEC										
SEPTIC RASH	0	0	0	0	0	0	0	0	0	0
SKIN STRUCTURES AND SOFT TISSUE INFECTIONS										
INFECTED DERMAL CYST	0	0	0	0	0	0	0	0	0	0
INJECTION SITE PUSTULE	0	0	0	0	0	0	0	0	0	0
SKIN INFECTION	0	0	0	0	0	0	0	0	0	0
UPPER RESPIRATORY TRACT INFECTIONS										
LARYNGITIS	0	0	0	0	0	0	0	0	0	0
NASOPHARYNGITIS	3	3	0	0	0	3	3	0	0	0
PHARYNGITIS	0	0	0	0	0	0	0	0	0	0
RHINITIS	0	0	0	0	0	0	0	0	0	0
SINUSITIS	0	0	0	0	0	0	0	0	0	0
TONSILLITIS	0	0	0	0	0	0	0	0	0	0
URINARY TRACT INFECTIONS										
CYSTITIS	0	0	0	0	0	0	0	0	0	0
URINARY TRACT INFECTION	0	0	0	0	0	0	0	0	0	0
PROTOZOAL INFECTIOUS DISORDERS										
TRYPANOSOMAL INFECTIONS										
AFRICAN TRYPANOSOMIASIS	0	0	0	0	0	0	0	0	0	0
RICKETTSIAL INFECTIOUS DISORDERS										
COXIELLA INFECTIONS										
Q FEVER	0	0	0	0	0	0	0	0	0	0
VIRAL INFECTIOUS DISORDERS										
CORONAVIRUS INFECTIONS										
COVID-19	0	0	0	0	0	0	0	0	0	0
HERPES VIRAL INFECTIONS										

GENITAL HERPES	0	0	0	0	0	0	0	0	0	0
HERPES OPHTHALMIC	0	0	0	0	0	0	0	0	0	0
HERPES SIMPLEX	0	0	0	0	0	0	0	0	0	0
HERPES ZOSTER	0	0	0	0	0	0	0	0	0	0
NASAL HERPES	0	0	0	0	0	0	0	0	0	0
OPHTHALMIC HERPES ZOSTER	0	0	0	0	0	0	0	0	0	0
ORAL HERPES	0	0	0	0	0	0	0	0	0	0
INFLUENZA VIRAL INFECTIONS										
H2N2 INFLUENZA	0	0	0	0	0	0	0	0	0	0
INFLUENZA	7	5	0	2	0	7	5	0	2	0
RETROVIRAL INFECTIONS										
AIDS RELATED COMPLEX	0	0	0	0	0	0	0	0	0	0
VIRAL INFECTIONS NEC										
GASTROENTERITIS VIRAL	0	0	0	0	0	0	0	0	0	0
SWEATING FEVER	0	0	0	0	0	0	0	0	0	0
VESTIBULAR NEURONITIS	0	0	0	0	0	0	0	0	0	0
VIRAL DIARRHOEA	0	0	0	0	0	0	0	0	0	0
VIRAL RASH	0	0	0	0	0	0	0	0	0	0
VIRAL UPPER RESPIRATORY TRACT INFECTION	0	0	0	0	0	0	0	0	0	0
**INJURY, POISONING AND PROCEDURAL COMPLICATIONS**										
BONE AND JOINT INJURIES										
FRACTURES AND DISLOCATIONS NEC										
JOINT DISLOCATION	0	0	0	0	0	0	0	0	0	0
EXPOSURES, CHEMICAL INJURIES AND POISONING										
POISONING AND TOXICITY										
SYSTEMIC TOXICITY	0	0	0	0	0	0	0	0	0	0
TOXICITY TO VARIOUS AGENTS	0	0	0	0	0	0	0	0	0	0
INJURIES BY PHYSICAL AGENTS										
CONDITIONS CAUSED BY COLD										
CHILLBLAINS	0	0	0	0	0	0	0	0	0	0
HEAT INJURIES (EXCL THERMAL BURNS)										
HEAT EXHAUSTION	0	0	0	0	0	0	0	0	0	0
HEAT OEDEMA	0	0	0	0	0	0	0	0	0	0
RADIATION INJURIES										
SUNBURN	0	0	0	0	0	0	0	0	0	0
THERMAL BURNS										

THERMAL BURN	0	0	0	0	0	0	0	0	0	0
THERMAL BURNS OF EYE	0	0	0	0	0	0	0	0	0	0
INJURIES NEC										
CHEST AND RESPIRATORY TRACT INJURIES NEC										
CHEST CRUSHING	0	0	0	0	0	0	0	0	0	0
EYE INJURIES NEC										
EYE CONTUSION	0	0	0	0	0	0	0	0	0	0
MUSCLE, TENDON AND LIGAMENT INJURIES										
LIGAMENT SPRAIN	0	0	0	0	0	0	0	0	0	0
MUSCLE INJURY	0	0	0	0	0	0	0	0	0	0
MUSCLE STRAIN	0	0	0	0	0	0	0	0	0	0
NERVE INJURIES NEC										
NERVE INJURY	0	0	0	0	0	0	0	0	0	0
NON-SITE SPECIFIC INJURIES NEC										
ARTHROPOD STING	0	0	0	0	0	0	0	0	0	0
BITE	0	0	0	0	0	0	0	0	0	0
FALL	0	0	0	0	0	0	0	0	0	0
INFLAMMATION OF WOUND	0	0	0	0	0	0	0	0	0	0
TISSUE INJURY	0	0	0	0	0	0	0	0	0	0
WOUND COMPLICATION	0	0	0	0	0	0	0	0	0	0
WOUND SECRETION	0	0	0	0	0	0	0	0	0	0
SITE SPECIFIC INJURIES NEC										
LIMB INJURY	0	0	0	0	0	0	0	0	0	0
SKIN INJURIES NEC										
CONTUSION	0	0	0	0	0	0	0	0	0	0
SCAR	0	0	0	0	0	0	0	0	0	0
SKIN WOUND	0	0	0	0	0	0	0	0	0	0
PROCEDURAL RELATED INJURIES AND COMPLICATIONS NEC										
CARDIAC AND VASCULAR PROCEDURAL COMPLICATIONS										
CARDIAC PROCEDURE COMPLICATION	0	0	0	0	0	0	0	0	0	0
GASTROINTESTINAL AND HEPATOBILIARY PROCEDURAL COMPLICATIONS										
PROCEDURAL NAUSEA	0	0	0	0	0	0	0	0	0	0
NEUROLOGICAL AND PSYCHIATRIC PROCEDURAL COMPLICATIONS										
PROCEDURAL DIZZINESS	0	0	0	0	0	0	0	0	0	0
NON-SITE SPECIFIC PROCEDURAL COMPLICATIONS										
INCISION SITE PAIN	0	0	0	0	0	0	0	0	0	0

INCISION SITE SWELLING	0	0	0	0	0	0	0	0	0	0
INJECTION RELATED REACTION	1	1	0	0	0	1	1	0	0	0
POST PROCEDURAL COMPLICATION	3	3	0	0	0	3	3	0	0	0
PROCEDURAL PAIN	0	0	0	0	0	0	0	0	0	0
VACCINATION RELATED COMPLICATIONS										
IMMUNISATION REACTION	0	0	0	0	0	0	0	0	0	0
**INVESTIGATIONS**										
CARDIAC AND VASCULAR INVESTIGATIONS (EXCL ENZYME TESTS)										
HEART RATE AND PULSE INVESTIGATIONS										
HEART RATE	0	0	0	0	0	0	0	0	0	0
HEART RATE DECREASED	0	0	0	0	0	0	0	0	0	0
HEART RATE INCREASED	1	1	0	0	0	1	1	0	0	0
HEART RATE IRREGULAR	0	0	0	0	0	0	0	0	0	0
VASCULAR TESTS NEC (INCL BLOOD PRESSURE)										
BLOOD PRESSURE DECREASED	0	0	0	0	0	0	0	0	0	0
BLOOD PRESSURE INCREASED	0	0	0	0	0	0	0	0	0	0
BLOOD PRESSURE MEASUREMENT	0	0	0	0	0	0	0	0	0	0
ENDOCRINE INVESTIGATIONS (INCL SEX HORMONES)										
ENDOCRINE ANALYSES AND IMAGING NEC										
HORMONE LEVEL ABNORMAL	0	0	0	0	0	0	0	0	0	0
PITUITARY ANALYSES ANTERIOR										
BLOOD FOLLICLE STIMULATING HORMONE INCREASED	0	0	0	0	0	0	0	0	0	0
BLOOD LUTEINISING HORMONE	0	0	0	0	0	0	0	0	0	0
THYROID ANALYSES										
TRI-IODOTHYRONINE	0	0	0	0	0	0	0	0	0	0
HAEMATOLOGY INVESTIGATIONS (INCL BLOOD GROUPS)										
COAGULATION AND BLEEDING ANALYSES										
BLEEDING TIME	0	0	0	0	0	0	0	0	0	0
INTERNATIONAL NORMALISED RATIO DECREASED	0	0	0	0	0	0	0	0	0	0
PLATELET ANALYSES										
PLATELET COUNT INCREASED	0	0	0	0	0	0	0	0	0	0
RED BLOOD CELL ANALYSES										
HAEMOGLOBIN	0	0	0	0	0	0	0	0	0	0
METABOLIC, NUTRITIONAL AND BLOOD GAS INVESTIGATIONS										
BLOOD GAS AND ACID BASE ANALYSES										

OXYGEN SATURATION DECREASED	0	0	0	0	0	0	0	0	0	0
CARBOHYDRATE TOLERANCE ANALYSES (INCL DIABETES)										
BLOOD GLUCOSE	0	0	0	0	0	0	0	0	0	0
BLOOD GLUCOSE ABNORMAL	0	0	0	0	0	0	0	0	0	0
BLOOD GLUCOSE DECREASED	0	0	0	0	0	0	0	0	0	0
BLOOD GLUCOSE INCREASED	0	0	0	0	0	0	0	0	0	0
MICROBIOLOGY AND SEROLOGY INVESTIGATIONS										
VIRUS IDENTIFICATION AND SEROLOGY										
SARS-COV-2 TEST	0	0	0	0	0	0	0	0	0	0
SARS-COV-2 TEST POSITIVE	0	0	0	0	0	0	0	0	0	0
MUSCULOSKELETAL AND SOFT TISSUE INVESTIGATIONS (EXCL ENZYME TESTS)										
MUSCULOSKELETAL AND SOFT TISSUE IMAGING PROCEDURES										
BONE SCAN	0	0	0	0	0	0	0	0	0	0
NEUROLOGICAL, SPECIAL SENSES AND PSYCHIATRIC INVESTIGATIONS										
CENTRAL NERVOUS SYSTEM IMAGING PROCEDURES										
MAGNETIC RESONANCE IMAGING HEAD	0	0	0	0	0	0	0	0	0	0
SCAN BRAIN	0	0	0	0	0	0	0	0	0	0
PHYSICAL EXAMINATION AND ORGAN SYSTEM STATUS TOPICS										
PHYSICAL EXAMINATION PROCEDURES AND ORGAN SYSTEM STATUS										
BODY TEMPERATURE	0	0	0	0	0	0	0	0	0	0
BODY TEMPERATURE ABNORMAL	0	0	0	0	0	0	0	0	0	0
BODY TEMPERATURE DECREASED	0	0	0	0	0	0	0	0	0	0
BODY TEMPERATURE FLUCTUATION	0	0	0	0	0	0	0	0	0	0
BODY TEMPERATURE INCREASED	1	1	0	0	0	1	1	0	0	0
GRIP STRENGTH DECREASED	0	0	0	0	0	0	0	0	0	0
HEAD LAG	0	0	0	0	0	0	0	0	0	0
LEFT-HANDEDNESS	0	0	0	0	0	0	0	0	0	0
LYMPH NODE PALPABLE	0	0	0	0	0	0	0	0	0	0
RESPIRATORY RATE DECREASED	0	0	0	0	0	0	0	0	0	0
SKIN TEMPERATURE	0	0	0	0	0	0	0	0	0	0
WEIGHT DECREASED	0	0	0	0	0	0	0	0	0	0
WEIGHT INCREASED	0	0	0	0	0	0	0	0	0	0
RENAL AND URINARY TRACT INVESTIGATIONS AND URINALYSES										
URINALYSIS NEC										
BLOOD URINE	0	0	0	0	0	0	0	0	0	0

NITRITE URINE PRESENT	0	0	0	0	0	0	0	0	0	0
PH URINE	0	0	0	0	0	0	0	0	0	0
URINARY TRACT FUNCTION ANALYSES NEC										
URINE OUTPUT	0	0	0	0	0	0	0	0	0	0
URINE OUTPUT INCREASED	0	0	0	0	0	0	0	0	0	0
REPRODUCTIVE ORGAN AND BREAST INVESTIGATIONS (EXCL HORMONE ANALYSES)										
REPRODUCTIVE ORGAN AND BREAST IMAGING PROCEDURES										
BREAST SCAN	0	0	0	0	0	0	0	0	0	0
RESPIRATORY AND PULMONARY INVESTIGATIONS (EXCL BLOOD GASES)										
RESPIRATORY AND PULMONARY FUNCTION DIAGNOSTIC PROCEDURES										
FORCED EXPIRATORY VOLUME DECREASED	0	0	0	0	0	0	0	0	0	0
FORCED EXPIRATORY VOLUME INCREASED	0	0	0	0	0	0	0	0	0	0
WATER, ELECTROLYTE AND MINERAL INVESTIGATIONS										
WATER AND ELECTROLYTE ANALYSES NEC										
VOLUME BLOOD	0	0	0	0	0	0	0	0	0	0
**METABOLISM AND NUTRITION DISORDERS**										
APPETITE AND GENERAL NUTRITIONAL DISORDERS										
APPETITE DISORDERS										
APPETITE DISORDER	0	0	0	0	0	0	0	0	0	0
DECREASED APPETITE	0	0	0	0	0	0	0	0	0	0
FOOD CRAVING	0	0	0	0	0	0	0	0	0	0
FOOD REFUSAL	0	0	0	0	0	0	0	0	0	0
INCREASED APPETITE	0	0	0	0	0	0	0	0	0	0
GENERAL NUTRITIONAL DISORDERS NEC										
FOOD AVERSION	0	0	0	0	0	0	0	0	0	0
ELECTROLYTE AND FLUID BALANCE CONDITIONS										
TOTAL FLUID VOLUME DECREASED										
DEHYDRATION	0	0	0	0	0	0	0	0	0	0
TOTAL FLUID VOLUME INCREASED										
FLUID RETENTION	0	0	0	0	0	0	0	0	0	0
FOOD INTOLERANCE SYNDROMES										
FOOD MALABSORPTION AND INTOLERANCE SYNDROMES (EXCL SUGAR INTOLERANCE)										
ALCOHOL INTOLERANCE	0	0	0	0	0	0	0	0	0	0
GLUCOSE METABOLISM DISORDERS (INCL DIABETES MELLITUS)										
DIABETES MELLITUS (INCL SUBTYPES)										
DIABETES MELLITUS	0	0	0	0	0	0	0	0	0	0

DIABETES MELLITUS INADEQUATE CONTROL	0	0	0	0	0	0	0	0	0	0
HYPERGLYCAEMIC CONDITIONS NEC										
HYPERGLYCAEMIA	0	0	0	0	0	0	0	0	0	0
HYPOGLYCAEMIC CONDITIONS NEC										
HYPOGLYCAEMIA	0	0	0	0	0	0	0	0	0	0
PURINE AND PYRIMIDINE METABOLISM DISORDERS										
DISORDERS OF PURINE METABOLISM										
GOUT	0	0	0	0	0	0	0	0	0	0
**MUSCULOSKELETAL AND CONNECTIVE TISSUE DISORDERS**										
BONE DISORDERS (EXCL CONGENITAL AND FRACTURES)										
BONE DISORDERS NEC										
JAW DISORDER	0	0	0	0	0	0	0	0	0	0
OSTEITIS	0	0	0	0	0	0	0	0	0	0
BONE RELATED SIGNS AND SYMPTOMS										
BONE PAIN	0	0	0	0	0	0	0	0	0	0
BONE SWELLING	0	0	0	0	0	0	0	0	0	0
PAIN IN JAW	0	0	0	0	0	0	0	0	0	0
PUBIC PAIN	0	0	0	0	0	0	0	0	0	0
SPINAL PAIN	0	0	0	0	0	0	0	0	0	0
CONNECTIVE TISSUE DISORDERS (EXCL CONGENITAL)										
CONNECTIVE TISSUE DISORDERS NEC										
POLYMYALGIA RHEUMATICA	0	0	0	0	0	0	0	0	0	0
LUPUS ERYTHEMATOSUS (INCL SUBTYPES)										
SYSTEMIC LUPUS ERYTHEMATOSUS	0	0	0	0	0	0	0	0	0	0
JOINT DISORDERS										
ARTHROPATHIES NEC										
ARTHRITIS	0	0	0	0	0	0	0	0	0	0
ARTHROPATHY	0	0	0	0	0	0	0	0	0	0
POLYARTHRITIS	0	0	0	0	0	0	0	0	0	0
RHEUMATIC FEVER	0	0	0	0	0	0	0	0	0	0
JOINT RELATED DISORDERS NEC										
JOINT LOCK	0	0	0	0	0	0	0	0	0	0
PERIARTHRITIS	0	0	0	0	0	0	0	0	0	0
TEMPOROMANDIBULAR PAIN AND DYSFUNCTION SYNDROME	0	0	0	0	0	0	0	0	0	0
JOINT RELATED SIGNS AND SYMPTOMS										

ARTHRALGIA	12	6	4	1	1	11	6	3	1	1
JAW CLICKING	0	0	0	0	0	0	0	0	0	0
JOINT STIFFNESS	0	0	0	0	0	0	0	0	0	0
JOINT SWELLING	0	0	0	0	0	0	0	0	0	0
OSTEOARTHROPATHIES										
OSTEOARTHRITIS	0	0	0	0	0	0	0	0	0	0
PSORIATIC ARTHROPATHIES										
PSORIATIC ARTHROPATHY	0	0	0	0	0	0	0	0	0	0
RHEUMATOID ARTHROPATHIES										
RHEUMATOID ARTHRITIS	0	0	0	0	0	0	0	0	0	0
MUSCLE DISORDERS										
MUSCLE INFECTIONS AND INFLAMMATIONS										
MYOSITIS	0	0	0	0	0	0	0	0	0	0
MUSCLE PAINS										
FIBROMYALGIA	0	0	0	0	0	0	0	0	0	0
MYALGIA	12	10	2	0	0	10	10	0	0	0
MUSCLE RELATED SIGNS AND SYMPTOMS NEC										
MUSCLE ATROPHY	0	0	0	0	0	0	0	0	0	0
MUSCLE DISCOMFORT	0	0	0	0	0	0	0	0	0	0
MUSCLE FATIGUE	6	5	1	0	0	5	5	0	0	0
MUSCLE FIBROSIS	0	0	0	0	0	0	0	0	0	0
MUSCLE MASS	0	0	0	0	0	0	0	0	0	0
MUSCLE SPASMS	1	1	0	0	0	1	1	0	0	0
MUSCLE TIGHTNESS	0	0	0	0	0	0	0	0	0	0
MUSCLE TWITCHING	0	0	0	0	0	0	0	0	0	0
MUSCLE TONE ABNORMALITIES										
MUSCLE RIGIDITY	0	0	0	0	0	0	0	0	0	0
TRISMUS	0	0	0	0	0	0	0	0	0	0
MUSCLE WEAKNESS CONDITIONS										
MUSCULAR WEAKNESS	1	1	0	0	0	1	1	0	0	0
MUSCULOSKELETAL AND CONNECTIVE TISSUE DISORDERS NEC										
MUSCULOSKELETAL AND CONNECTIVE TISSUE CONDITIONS NEC										
MOBILITY DECREASED	0	0	0	0	0	0	0	0	0	0
MUSCULOSKELETAL STIFFNESS	1	1	0	0	0	1	1	0	0	0
BACK PAIN	2	2	0	0	0	2	2	0	0	0

FLANK PAIN	0	0	0	0	0	0	0	0	0	0
LIMB DISCOMFORT	3	1	0	2	0	3	1	0	2	0
MUSCULOSKELETAL CHEST PAIN	0	0	0	0	0	0	0	0	0	0
MUSCULOSKELETAL DISCOMFORT	0	0	0	0	0	0	0	0	0	0
MUSCULOSKELETAL PAIN	0	0	0	0	0	0	0	0	0	0
NECK PAIN	2	2	0	0	0	2	2	0	0	0
PAIN IN EXTREMITY	29	9	5	8	7	24	9	4	8	3
SOFT TISSUE DISORDERS NEC										
AXILLARY MASS	0	0	0	0	0	0	0	0	0	0
GROIN PAIN	0	0	0	0	0	0	0	0	0	0
SYNOVIAL AND BURSAL DISORDERS										
SYNOVIAL DISORDERS										
SYNOVITIS	0	0	0	0	0	0	0	0	0	0
TENDON, LIGAMENT AND CARTILAGE DISORDERS										
CARTILAGE DISORDERS										
COSTOCHONDRITIS	0	0	0	0	0	0	0	0	0	0
TENDON DISORDERS										
TENDONITIS	0	0	0	0	0	0	0	0	0	0
TENOSYNOVITIS	0	0	0	0	0	0	0	0	0	0
TRIGGER FINGER	0	0	0	0	0	0	0	0	0	0
**NEOPLASMS BENIGN, MALIGNANT AND UNSPECIFIED (INCL CYSTS AND POLYPS)**										
CUTANEOUS NEOPLASMS BENIGN										
SKIN NEOPLASMS BENIGN										
MELANOCYTIC NAEVUS	0	0	0	0	0	0	0	0	0	0
SEBORRHOEIC KERATOSIS	0	0	0	0	0	0	0	0	0	0
SKIN PAPILLOMA	0	0	0	0	0	0	0	0	0	0
**NERVOUS SYSTEM DISORDERS**										
CENTRAL NERVOUS SYSTEM INFECTIONS AND INFLAMMATIONS										
MYELITIS (INCL INFECTIVE)										
MYELITIS TRANSVERSE	0	0	0	0	0	0	0	0	0	0
CENTRAL NERVOUS SYSTEM VASCULAR DISORDERS										
CENTRAL NERVOUS SYSTEM HAEMORRHAGES AND CEREBROVASCULAR ACCIDENTS										
CEREBRAL HAEMORRHAGE	0	0	0	0	0	0	0	0	0	0
CEREBROVASCULAR ACCIDENT	0	0	0	0	0	0	0	0	0	0
TRANSIENT CEREBROVASCULAR EVENTS										
TRANSIENT ISCHAEMIC ATTACK	0	0	0	0	0	0	0	0	0	0

CRANIAL NERVE DISORDERS (EXCL NEOPLASMS)										
FACIAL CRANIAL NERVE DISORDERS										
BELL'S PALSY	0	0	0	0	0	0	0	0	0	0
FACIAL PARALYSIS	0	0	0	0	0	0	0	0	0	0
FACIAL PARESIS	0	0	0	0	0	0	0	0	0	0
OLFACTORY NERVE DISORDERS										
ANOSMIA	0	0	0	0	0	0	0	0	0	0
HYPOSMIA	0	0	0	0	0	0	0	0	0	0
PAROSMIA	0	0	0	0	0	0	0	0	0	0
TRIGEMINAL DISORDERS										
TRIGEMINAL NEURALGIA	0	0	0	0	0	0	0	0	0	0
TRIGEMINAL NEURITIS	0	0	0	0	0	0	0	0	0	0
DEMYELINATING DISORDERS										
MULTIPLE SCLEROSIS ACUTE AND PROGRESSIVE										
MULTIPLE SCLEROSIS RELAPSE	0	0	0	0	0	0	0	0	0	0
HEADACHES										
HEADACHES NEC										
CLUSTER HEADACHE	0	0	0	0	0	0	0	0	0	0
COLD-STIMULUS HEADACHE	0	0	0	0	0	0	0	0	0	0
DRUG WITHDRAWAL HEADACHE	0	0	0	0	0	0	0	0	0	0
HEADACHE	37	26	3	8	0	36	26	2	8	0
PRIMARY STABBING HEADACHE	0	0	0	0	0	0	0	0	0	0
SINUS HEADACHE	2	1	1	0	0	2	1	1	0	0
TENSION HEADACHE	5	2	0	3	0	5	2	0	3	0
THUNDERCLAP HEADACHE	0	0	0	0	0	0	0	0	0	0
VASCULAR HEADACHE	0	0	0	0	0	0	0	0	0	0
MIGRAINE HEADACHES										
MIGRAINE	3	3	0	0	0	3	3	0	0	0
MIGRAINE WITH AURA	0	0	0	0	0	0	0	0	0	0
RETINAL MIGRAINE	0	0	0	0	0	0	0	0	0	0
TYPICAL AURA WITHOUT HEADACHE	0	0	0	0	0	0	0	0	0	0
MENTAL IMPAIRMENT DISORDERS										
INTELLECTUAL DISABILITIES										
INTELLECTUAL DISABILITY	0	0	0	0	0	0	0	0	0	0
MEMORY LOSS (EXCL DEMENTIA)										

AMNESIA	0	0	0	0	0	0	0	0	0	0
MEMORY IMPAIRMENT	0	0	0	0	0	0	0	0	0	0
MENTAL IMPAIRMENT (EXCL DEMENTIA AND MEMORY LOSS)										
COGNITIVE DISORDER	0	0	0	0	0	0	0	0	0	0
DISTURBANCE IN ATTENTION	0	0	0	0	0	0	0	0	0	0
MENTAL IMPAIRMENT	0	0	0	0	0	0	0	0	0	0
MOVEMENT DISORDERS (INCL PARKINSONISM)										
DYSKINESIAS AND MOVEMENT DISORDERS NEC										
BRADYKINESIA	0	0	0	0	0	0	0	0	0	0
CLUMSINESS	0	0	0	0	0	0	0	0	0	0
DYSKINESIA	0	0	0	0	0	0	0	0	0	0
EXTRAPYRAMIDAL DISORDER	0	0	0	0	0	0	0	0	0	0
PSYCHOMOTOR HYPERACTIVITY	0	0	0	0	0	0	0	0	0	0
PARALYSIS AND PARESIS (EXCL CRANIAL NERVE)										
HEMIPLEGIA	0	0	0	0	0	0	0	0	0	0
MONOPARESIS	0	0	0	0	0	0	0	0	0	0
MONOPLEGIA	0	0	0	0	0	0	0	0	0	0
PARALYSIS	0	0	0	0	0	0	0	0	0	0
PARKINSON'S DISEASE AND PARKINSONISM										
FREEZING PHENOMENON	0	0	0	0	0	0	0	0	0	0
PARKINSON'S DISEASE	0	0	0	0	0	0	0	0	0	0
TREMOR (EXCL CONGENITAL)										
TREMOR	3	2	0	1	0	3	2	0	1	0
NEUROLOGICAL DISORDERS NEC										
COMA STATES										
DIABETIC HYPERGLYCAEMIC COMA	0	0	0	0	0	0	0	0	0	0
COORDINATION AND BALANCE DISTURBANCES										
BALANCE DISORDER	0	0	0	0	0	0	0	0	0	0
COORDINATION ABNORMAL	0	0	0	0	0	0	0	0	0	0
DYSSTASIA	0	0	0	0	0	0	0	0	0	0
VESTIBULAR NYSTAGMUS	0	0	0	0	0	0	0	0	0	0
DISTURBANCES IN CONSCIOUSNESS NEC										
DEPRESSED LEVEL OF CONSCIOUSNESS	0	0	0	0	0	0	0	0	0	0
LETHARGY	1	0	1	0	0	1	0	1	0	0
LOSS OF CONSCIOUSNESS	0	0	0	0	0	0	0	0	0	0

SEDATION	0	0	0	0	0	0	0	0	0	0
SOMNOLENCE	2	0	2	0	0	2	0	2	0	0
SYNCOPE	0	0	0	0	0	0	0	0	0	0
NERVOUS SYSTEM DISORDERS NEC										
NERVOUS SYSTEM DISORDER	0	0	0	0	0	0	0	0	0	0
NEUROLOGICAL SIGNS AND SYMPTOMS NEC										
AGITATION NEONATAL	0	0	0	0	0	0	0	0	0	0
BRAIN FOG	1	1	0	0	0	1	1	0	0	0
DIZZINESS	2	1	0	1	0	2	1	0	1	0
DIZZINESS EXERTIONAL	0	0	0	0	0	0	0	0	0	0
DIZZINESS POSTURAL	3	2	1	0	0	3	2	1	0	0
HEAD DISCOMFORT	0	0	0	0	0	0	0	0	0	0
INFANT IRRITABILITY	0	0	0	0	0	0	0	0	0	0
MENINGISM	0	0	0	0	0	0	0	0	0	0
MYOCLONUS	0	0	0	0	0	0	0	0	0	0
NEUROLOGICAL SYMPTOM	0	0	0	0	0	0	0	0	0	0
PERSISTENT POSTURAL-PERCEPTUAL DIZZINESS	0	0	0	0	0	0	0	0	0	0
PRESYNCOPE	0	0	0	0	0	0	0	0	0	0
PARAESTHESIAS AND DYSAESTHESIAS										
BURNING FEET SYNDROME	0	0	0	0	0	0	0	0	0	0
BURNING SENSATION	0	0	0	0	0	0	0	0	0	0
FORMICATION	0	0	0	0	0	0	0	0	0	0
HYPERAESTHESIA	0	0	0	0	0	0	0	0	0	0
HYPOAESTHESIA	0	0	0	0	0	0	0	0	0	0
PARAESTHESIA	2	2	0	0	0	2	2	0	0	0
REVERSED HOT-COLD SENSATION	0	0	0	0	0	0	0	0	0	0
SENSORY ABNORMALITIES NEC										
AGEUSIA	0	0	0	0	0	0	0	0	0	0
ALLODYNIA	0	0	0	0	0	0	0	0	0	0
DYSGEUSIA	0	0	0	0	0	0	0	0	0	0
HYPOGEUSIA	0	0	0	0	0	0	0	0	0	0
NEURALGIA	0	0	0	0	0	0	0	0	0	0
POST HERPETIC NEURALGIA	0	0	0	0	0	0	0	0	0	0
RESTLESS ARM SYNDROME	0	0	0	0	0	0	0	0	0	0
RESTLESS LEGS SYNDROME	0	0	0	0	0	0	0	0	0	0

SENSORY LOSS	0	0	0	0	0	0	0	0	0	0
TASTE DISORDER	0	0	0	0	0	0	0	0	0	0
SPEECH AND LANGUAGE ABNORMALITIES										
DYSARTHRIA	0	0	0	0	0	0	0	0	0	0
SPEECH DISORDER DEVELOPMENTAL	0	0	0	0	0	0	0	0	0	0
NEUROLOGICAL DISORDERS OF THE EYE										
NEUROLOGIC VISUAL PROBLEMS NEC										
TUNNEL VISION	0	0	0	0	0	0	0	0	0	0
NEUROMUSCULAR DISORDERS										
MUSCLE TONE ABNORMAL										
HYPOTONIA	0	0	0	0	0	0	0	0	0	0
STIFF LEG SYNDROME	0	0	0	0	0	0	0	0	0	0
NEUROMUSCULAR DISORDERS NEC										
MUSCLE SPASTICITY	0	0	0	0	0	0	0	0	0	0
PERIPHERAL NEUROPATHIES										
ACUTE POLYNEUROPATHIES										
GUILLAIN-BARRE SYNDROME	0	0	0	0	0	0	0	0	0	0
PERIPHERAL NEUROPATHIES NEC										
AXONAL NEUROPATHY	1	1	0	0	0	1	1	0	0	0
NEUROPATHY PERIPHERAL	0	0	0	0	0	0	0	0	0	0
SEIZURES (INCL SUBTYPES)										
SEIZURES AND SEIZURE DISORDERS NEC										
EPILEPSY	0	0	0	0	0	0	0	0	0	0
SEIZURE	0	0	0	0	0	0	0	0	0	0
SLEEP DISTURBANCES (INCL SUBTYPES)										
DISTURBANCES IN SLEEP PHASE RHYTHM										
CIRCADIAN RHYTHM SLEEP DISORDER	0	0	0	0	0	0	0	0	0	0
SLEEP DISTURBANCES NEC										
SLEEP DEFICIT	0	0	0	0	0	0	0	0	0	0
SPINAL CORD AND NERVE ROOT DISORDERS										
CERVICAL SPINAL CORD AND NERVE ROOT DISORDERS										
CERVICOBRACHIAL SYNDROME	0	0	0	0	0	0	0	0	0	0
LUMBAR SPINAL CORD AND NERVE ROOT DISORDERS										
CAUDA EQUINA SYNDROME	0	0	0	0	0	0	0	0	0	0
SCIATICA	0	0	0	0	0	0	0	0	0	0
**PREGNANCY, PUERPERIUM AND PERINATAL CONDITIONS**										

ABORTIONS AND STILLBIRTH										
ABORTIONS SPONTANEOUS										
ABORTION SPONTANEOUS	0	0	0	0	0	0	0	0	0	0
STILLBIRTH AND FOETAL DEATH										
FOETAL DEATH	0	0	0	0	0	0	0	0	0	0
FOETAL COMPLICATIONS										
FOETAL COMPLICATIONS NEC										
FOETAL DISORDER	1	1	0	0	0	1	1	0	0	0
FOETAL HYPOKINESIA	0	0	0	0	0	0	0	0	0	0
FOETAL GROWTH COMPLICATIONS										
FOETAL MACROSOMIA	0	0	0	0	0	0	0	0	0	0
MATERNAL COMPLICATIONS OF PREGNANCY										
MATERNAL COMPLICATIONS OF PREGNANCY NEC										
MORNING SICKNESS	0	0	0	0	0	0	0	0	0	0
PLACENTAL, AMNIOTIC AND CAVITY DISORDERS (EXCL HAEMORRHAGES)										
PLACENTAL ABNORMALITIES (EXCL NEOPLASMS)										
PLACENTAL INFARCTION	0	0	0	0	0	0	0	0	0	0
PREGNANCY, LABOUR, DELIVERY AND POSTPARTUM CONDITIONS										
NORMAL PREGNANCY, LABOUR AND DELIVERY										
PREGNANCY	0	0	0	0	0	0	0	0	0	0
UTERINE CONTRACTIONS DURING PREGNANCY	0	0	0	0	0	0	0	0	0	0
**PRODUCT ISSUES**										
DEVICE ISSUES										
DEVICE MALFUNCTION EVENTS NEC										
OVERSENSING	0	0	0	0	0	0	0	0	0	0
**PSYCHIATRIC DISORDERS**										
ANXIETY DISORDERS AND SYMPTOMS										
ANXIETY SYMPTOMS										
AGITATION	0	0	0	0	0	0	0	0	0	0
ANXIETY	0	0	0	0	0	0	0	0	0	0
NERVOUSNESS	0	0	0	0	0	0	0	0	0	0
TENSION	0	0	0	0	0	0	0	0	0	0
PANIC ATTACKS AND DISORDERS										
PANIC ATTACK	0	0	0	0	0	0	0	0	0	0
CHANGES IN PHYSICAL ACTIVITY										
INCREASED PHYSICAL ACTIVITY LEVELS										

RESTLESSNESS	0	0	0	0	0	0	0	0	0	0
STEREOTYPIES AND AUTOMATISMS										
BRUXISM	0	0	0	0	0	0	0	0	0	0
COGNITIVE AND ATTENTION DISORDERS AND DISTURBANCES										
COGNITIVE AND ATTENTION DISORDERS AND DISTURBANCES NEC										
DAYDREAMING	0	0	0	0	0	0	0	0	0	0
MENTAL FATIGUE	1	1	0	0	0	1	1	0	0	0
COMMUNICATION DISORDERS AND DISTURBANCES										
SPEECH ARTICULATION AND RHYTHM DISTURBANCES										
DYSPHEMIA	0	0	0	0	0	0	0	0	0	0
DELIRIA (INCL CONFUSION)										
CONFUSION AND DISORIENTATION										
CONFUSIONAL STATE	0	0	0	0	0	0	0	0	0	0
DISORIENTATION	0	0	0	0	0	0	0	0	0	0
DELIRIA										
DELIRIUM	0	0	0	0	0	0	0	0	0	0
DEPRESSED MOOD DISORDERS AND DISTURBANCES										
DEPRESSIVE DISORDERS										
DEPRESSION	0	0	0	0	0	0	0	0	0	0
MOOD ALTERATIONS WITH DEPRESSIVE SYMPTOMS										
DEPRESSED MOOD	1	1	0	0	0	1	1	0	0	0
TEARFULNESS	0	0	0	0	0	0	0	0	0	0
DISSOCIATIVE DISORDERS										
DISSOCIATIVE STATES										
DISSOCIATION	0	0	0	0	0	0	0	0	0	0
DISTURBANCES IN THINKING AND PERCEPTION										
DELUSIONAL SYMPTOMS										
DELUSION	0	0	0	0	0	0	0	0	0	0
HALLUCINATIONS (EXCL SLEEP-RELATED)										
HALLUCINATION	0	0	0	0	0	0	0	0	0	0
PERCEPTION DISTURBANCES NEC										
DEREALISATION	0	0	0	0	0	0	0	0	0	0
THINKING DISTURBANCES										
BRADYPHRENIA	0	0	0	0	0	0	0	0	0	0
THOUGHT BLOCKING	0	0	0	0	0	0	0	0	0	0
MOOD DISORDERS AND DISTURBANCES NEC										

AFFECT ALTERATIONS NEC										
AFFECT LABILITY	0	0	0	0	0	0	0	0	0	0
INAPPROPRIATE AFFECT	0	0	0	0	0	0	0	0	0	0
EMOTIONAL AND MOOD DISTURBANCES NEC										
ANGER	0	0	0	0	0	0	0	0	0	0
EMOTIONAL DISORDER	0	0	0	0	0	0	0	0	0	0
EMOTIONAL DISTRESS	0	0	0	0	0	0	0	0	0	0
EUPHORIC MOOD	0	0	0	0	0	0	0	0	0	0
IRRITABILITY	0	0	0	0	0	0	0	0	0	0
MOOD ALTERED	0	0	0	0	0	0	0	0	0	0
FLUCTUATING MOOD SYMPTOMS										
MOOD SWINGS	0	0	0	0	0	0	0	0	0	0
MOOD DISORDERS NEC										
APATHY	0	0	0	0	0	0	0	0	0	0
LISTLESS	0	0	0	0	0	0	0	0	0	0
PERSONALITY DISORDERS AND DISTURBANCES IN BEHAVIOUR										
BEHAVIOUR AND SOCIALISATION DISTURBANCES										
AGGRESSION	0	0	0	0	0	0	0	0	0	0
PSYCHIATRIC AND BEHAVIOURAL SYMPTOMS NEC										
PSYCHIATRIC SYMPTOMS NEC										
PSYCHIATRIC SYMPTOM	0	0	0	0	0	0	0	0	0	0
PSYCHIATRIC DISORDERS NEC										
MENTAL DISORDERS NEC										
MENTAL DISORDER	0	0	0	0	0	0	0	0	0	0
SCHIZOPHRENIA AND OTHER PSYCHOTIC DISORDERS										
PSYCHOTIC DISORDER NEC										
PSYCHOTIC DISORDER	0	0	0	0	0	0	0	0	0	0
SEXUAL DYSFUNCTIONS, DISTURBANCES AND GENDER IDENTITY DISORDERS										
SEXUAL DESIRE DISORDERS										
LOSS OF LIBIDO	0	0	0	0	0	0	0	0	0	0
SLEEP DISORDERS AND DISTURBANCES										
DISTURBANCES IN INITIATING AND MAINTAINING SLEEP										
INITIAL INSOMNIA	0	0	0	0	0	0	0	0	0	0
INSOMNIA	0	0	0	0	0	0	0	0	0	0
DYSSOMNIAS										
BREATHING-RELATED SLEEP DISORDER	0	0	0	0	0	0	0	0	0	0

POOR QUALITY SLEEP	0	0	0	0	0	0	0	0	0	0
PARASOMNIAS										
ABNORMAL DREAMS	0	0	0	0	0	0	0	0	0	0
EXPLODING HEAD SYNDROME	0	0	0	0	0	0	0	0	0	0
NIGHTMARE	0	0	0	0	0	0	0	0	0	0
SLEEP DISORDERS NEC										
SLEEP DISORDER	0	0	0	0	0	0	0	0	0	0
SOMATIC SYMPTOM AND RELATED DISORDERS										
SOMATIC SYMPTOM DISORDERS										
HABIT COUGH	0	0	0	0	0	0	0	0	0	0
SUICIDAL AND SELF-INJURIOUS BEHAVIOURS NEC										
SUICIDAL AND SELF-INJURIOUS BEHAVIOUR										
SUICIDAL IDEATION	0	0	0	0	0	0	0	0	0	0
**RENAL AND URINARY DISORDERS**										
RENAL DISORDERS (EXCL NEPHROPATHIES)										
RENAL FAILURE AND IMPAIRMENT										
RENAL FAILURE	0	0	0	0	0	0	0	0	0	0
URINARY TRACT SIGNS AND SYMPTOMS										
BLADDER AND URETHRAL SYMPTOMS										
BLADDER PAIN	0	0	0	0	0	0	0	0	0	0
MICTURITION URGENCY	0	0	0	0	0	0	0	0	0	0
POLLAKIURIA	0	0	0	0	0	0	0	0	0	0
URINARY INCONTINENCE	0	0	0	0	0	0	0	0	0	0
URINARY ABNORMALITIES										
HAEMATURIA	0	0	0	0	0	0	0	0	0	0
URINE ABNORMALITY	0	0	0	0	0	0	0	0	0	0
URINE ODOUR ABNORMAL	0	0	0	0	0	0	0	0	0	0
URINARY TRACT SIGNS AND SYMPTOMS NEC										
POLYURIA	0	0	0	0	0	0	0	0	0	0
RENAL PAIN	0	0	0	0	0	0	0	0	0	0
**REPRODUCTIVE SYSTEM AND BREAST DISORDERS**										
BREAST DISORDERS										
BREAST DISORDERS NEC										
BREAST MASS	0	0	0	0	0	0	0	0	0	0
NIPPLE ENLARGEMENT	0	0	0	0	0	0	0	0	0	0
BREAST SIGNS AND SYMPTOMS										

BREAST DISCHARGE	0	0	0	0	0	0	0	0	0	0
BREAST PAIN	0	0	0	0	0	0	0	0	0	0
BREAST SWELLING	0	0	0	0	0	0	0	0	0	0
BREAST TENDERNESS	0	0	0	0	0	0	0	0	0	0
NIPPLE PAIN	0	0	0	0	0	0	0	0	0	0
MENOPAUSE RELATED CONDITIONS										
MENOPAUSAL EFFECTS ON THE GENITOURINARY TRACT										
POSTMENOPAUSAL HAEMORRHAGE	0	0	0	0	0	0	0	0	0	0
MENSTRUAL CYCLE AND UTERINE BLEEDING DISORDERS										
MENSTRUATION AND UTERINE BLEEDING NEC										
DYSMENORRHOEA	0	0	0	0	0	0	0	0	0	0
INTERMENSTRUAL BLEEDING	0	0	0	0	0	0	0	0	0	0
MENSTRUAL DISORDER	0	0	0	0	0	0	0	0	0	0
MENSTRUATION IRREGULAR	0	0	0	0	0	0	0	0	0	0
PREMENSTRUAL PAIN	0	0	0	0	0	0	0	0	0	0
RETROGRADE MENSTRUATION	0	0	0	0	0	0	0	0	0	0
MENSTRUATION WITH DECREASED BLEEDING										
AMENORRHOEA	0	0	0	0	0	0	0	0	0	0
HYPOMENORRHOEA	0	0	0	0	0	0	0	0	0	0
MENSTRUATION DELAYED	0	0	0	0	0	0	0	0	0	0
OLIGOMENORRHOEA	0	0	0	0	0	0	0	0	0	0
MENSTRUATION WITH INCREASED BLEEDING										
HEAVY MENSTRUAL BLEEDING	1	0	1	0	0	1	0	1	0	0
POLYMENORRHOEA	0	0	0	0	0	0	0	0	0	0
PENILE AND SCROTAL DISORDERS (EXCL INFECTIONS AND INFLAMMATIONS)										
SCROTAL DISORDERS NEC										
SCROTAL SWELLING	0	0	0	0	0	0	0	0	0	0
REPRODUCTIVE TRACT DISORDERS NEC										
REPRODUCTIVE TRACT DISORDERS NEC (EXCL NEOPLASMS)										
GENITAL LESION	0	0	0	0	0	0	0	0	0	0
REPRODUCTIVE TRACT SIGNS AND SYMPTOMS NEC										
GENITAL DISCOMFORT	0	0	0	0	0	0	0	0	0	0
GENITAL PAIN	0	0	0	0	0	0	0	0	0	0
UTERINE, PELVIC AND BROAD LIGAMENT DISORDERS										
PELVIS AND BROAD LIGAMENT DISORDERS NEC										
ADNEXA UTERI PAIN	0	0	0	0	0	0	0	0	0	0

VULVOVAGINAL DISORDERS (EXCL INFECTIONS AND INFLAMMATIONS)										
VULVOVAGINAL DISORDERS NEC										
VAGINAL HAEMORRHAGE	0	0	0	0	0	0	0	0	0	0
VULVOVAGINAL SIGNS AND SYMPTOMS										
VAGINAL DISCHARGE	0	0	0	0	0	0	0	0	0	0
**RESPIRATORY, THORACIC AND MEDIASTINAL DISORDERS**										
BRONCHIAL DISORDERS (EXCL NEOPLASMS)										
BRONCHOSPASM AND OBSTRUCTION										
ASTHMA	0	0	0	0	0	0	0	0	0	0
WHEEZING	0	0	0	0	0	0	0	0	0	0
LOWER RESPIRATORY TRACT DISORDERS (EXCL OBSTRUCTION AND INFECTION)										
PULMONARY OEDEMAS										
PULMONARY CONGESTION	0	0	0	0	0	0	0	0	0	0
PULMONARY VASCULAR DISORDERS										
PULMONARY THROMBOTIC AND EMBOLIC CONDITIONS										
PULMONARY EMBOLISM	0	0	0	0	0	0	0	0	0	0
RESPIRATORY DISORDERS NEC										
BREATHING ABNORMALITIES										
DYSPNOEA	1	1	0	0	0	1	1	0	0	0
HYPERVENTILATION	0	0	0	0	0	0	0	0	0	0
HYPOPNOEA	0	0	0	0	0	0	0	0	0	0
IRREGULAR BREATHING	0	0	0	0	0	0	0	0	0	0
MOUTH BREATHING	0	0	0	0	0	0	0	0	0	0
RESPIRATION ABNORMAL	0	0	0	0	0	0	0	0	0	0
RESPIRATORY ARREST	0	0	0	0	0	0	0	0	0	0
RESPIRATORY FATIGUE	0	0	0	0	0	0	0	0	0	0
SLEEP APNOEA SYNDROME	0	0	0	0	0	0	0	0	0	0
COUGHING AND ASSOCIATED SYMPTOMS										
COUGH	1	1	0	0	0	1	1	0	0	0
PRODUCTIVE COUGH	0	0	0	0	0	0	0	0	0	0
RESPIRATORY TRACT DISORDERS NEC										
RESPIRATORY TRACT IRRITATION	0	0	0	0	0	0	0	0	0	0
RESPIRATORY TRACT SIGNS AND SYMPTOMS										
LOWER RESPIRATORY TRACT SIGNS AND SYMPTOMS										
PULMONARY PAIN	0	0	0	0	0	0	0	0	0	0
RESPIRATORY SIGNS AND SYMPTOMS NEC										

RESPIRATORY SYMPTOM	0	0	0	0	0	0	0	0	0	0
UPPER RESPIRATORY TRACT SIGNS AND SYMPTOMS										
APHONIA	0	0	0	0	0	0	0	0	0	0
CATARRH	0	0	0	0	0	0	0	0	0	0
DRY THROAT	0	0	0	0	0	0	0	0	0	0
DYSPHONIA	0	0	0	0	0	0	0	0	0	0
INCREASED UPPER AIRWAY SECRETION	0	0	0	0	0	0	0	0	0	0
INCREASED VISCOSITY OF UPPER RESPIRATORY SE(	0	0	0	0	0	0	0	0	0	0
NASAL DISCOMFORT	0	0	0	0	0	0	0	0	0	0
OROPHARYNGEAL DISCOMFORT	0	0	0	0	0	0	0	0	0	0
OROPHARYNGEAL PAIN	1	1	0	0	0	1	1	0	0	0
PARANASAL SINUS DISCOMFORT	0	0	0	0	0	0	0	0	0	0
RHINALGIA	1	1	0	0	0	1	1	0	0	0
RHINORRHOEA	1	0	0	1	0	1	0	0	1	0
SINUS PAIN	1	0	0	1	0	1	0	0	1	0
SNEEZING	0	0	0	0	0	0	0	0	0	0
THROAT CLEARING	0	0	0	0	0	0	0	0	0	0
THROAT IRRITATION	0	0	0	0	0	0	0	0	0	0
THROAT TIGHTNESS	0	0	0	0	0	0	0	0	0	0
UPPER-AIRWAY COUGH SYNDROME	0	0	0	0	0	0	0	0	0	0
YAWNING	0	0	0	0	0	0	0	0	0	0
UPPER RESPIRATORY TRACT DISORDERS (EXCL INFECTIONS)										
NASAL CONGESTION AND INFLAMMATIONS										
NASAL CONGESTION	0	0	0	0	0	0	0	0	0	0
RHINITIS ALLERGIC	0	0	0	0	0	0	0	0	0	0
RHINITIS ATROPHIC	0	0	0	0	0	0	0	0	0	0
NASAL DISORDERS NEC										
EPISTAXIS	1	0	1	0	0	1	0	1	0	0
NASAL DRYNESS	0	0	0	0	0	0	0	0	0	0
PARANASAL SINUS DISORDERS (EXCL INFECTIONS AND NEOPLASMS)										
SINUS CONGESTION	0	0	0	0	0	0	0	0	0	0
PHARYNGEAL DISORDERS (EXCL INFECTIONS AND NEOPLASMS)										
PHARYNGEAL SWELLING	0	0	0	0	0	0	0	0	0	0
PHARYNGEAL ULCERATION	0	0	0	0	0	0	0	0	0	0
TONSILLAR ERYTHEMA	0	0	0	0	0	0	0	0	0	0

TRACHEAL DISORDERS (EXCL INFECTIONS AND NEOPLASMS)										
TRACHEAL PAIN	0	0	0	0	0	0	0	0	0	0
**SKIN AND SUBCUTANEOUS TISSUE DISORDERS**										
ANGIOEDEMA AND URTICARIA										
ANGIOEDEMAS										
ANGIOEDEMA	0	0	0	0	0	0	0	0	0	0
URTICARIAS										
COLD URTICARIA	0	0	0	0	0	0	0	0	0	0
SOLAR URTICARIA	0	0	0	0	0	0	0	0	0	0
URTICARIA	0	0	0	0	0	0	0	0	0	0
URTICARIA CHRONIC	0	0	0	0	0	0	0	0	0	0
CORNIFICATION AND DYSTROPHIC SKIN DISORDERS										
SKIN DYSTROPHIES										
HYPERTROPHIC SCAR	0	0	0	0	0	0	0	0	0	0
CUTANEOUS NEOPLASMS BENIGN										
SKIN CYSTS AND POLYPS										
DERMAL CYST	0	0	0	0	0	0	0	0	0	0
EPIDERMAL AND DERMAL CONDITIONS										
BULLOUS CONDITIONS										
BLISTER	0	0	0	0	0	0	0	0	0	0
BLOOD BLISTER	0	0	0	0	0	0	0	0	0	0
PEMPHIGOID	0	0	0	0	0	0	0	0	0	0
TOXIC EPIDERMAL NECROLYSIS	0	0	0	0	0	0	0	0	0	0
DERMAL AND EPIDERMAL CONDITIONS NEC										
DRY SKIN	0	0	0	0	0	0	0	0	0	0
PAIN OF SKIN	0	0	0	0	0	0	0	0	0	0
SENSITIVE SKIN	0	0	0	0	0	0	0	0	0	0
SKIN BURNING SENSATION	0	0	0	0	0	0	0	0	0	0
SKIN DISCOLOURATION	0	0	0	0	0	0	0	0	0	0
SKIN FRAGILITY	0	0	0	0	0	0	0	0	0	0
SKIN LESION	0	0	0	0	0	0	0	0	0	0
SKIN ODOUR ABNORMAL	0	0	0	0	0	0	0	0	0	0
SKIN REACTION	0	0	0	0	0	0	0	0	0	0
SKIN SENSITISATION	0	0	0	0	0	0	0	0	0	0
SKIN WARM	0	0	0	0	0	0	0	0	0	0
DERMATITIS AND ECZEMA										

DERMATITIS	0	0	0	0	0	0	0	0	0	0
DERMATITIS ALLERGIC	1	1	0	0	0	1	1	0	0	0
DERMATITIS ATOPIC	0	0	0	0	0	0	0	0	0	0
DERMATITIS CONTACT	0	0	0	0	0	0	0	0	0	0
ECZEMA	0	0	0	0	0	0	0	0	0	0
ECZEMA ASTEATOTIC	0	0	0	0	0	0	0	0	0	0
NEURODERMATITIS	0	0	0	0	0	0	0	0	0	0
SEBORRHOEIC DERMATITIS	0	0	0	0	0	0	0	0	0	0
SKIN IRRITATION	1	1	0	0	0	1	1	0	0	0
ERYTHEMAS										
ERYTHEMA	0	0	0	0	0	0	0	0	0	0
EXFOLIATIVE CONDITIONS										
SKIN EXFOLIATION	0	0	0	0	0	0	0	0	0	0
PAPULOSQUAMOUS CONDITIONS										
LICHEN PLANUS	0	0	0	0	0	0	0	0	0	0
PITYRIASIS ROSEA	0	0	0	0	0	0	0	0	0	0
PHOTOSENSITIVITY AND PHOTODERMATOSIS CONDITIONS										
PHOTOSENSITIVITY REACTION	0	0	0	0	0	0	0	0	0	0
PRURITUS NEC										
PRURITUS	0	0	0	0	0	0	0	0	0	0
PSORIATIC CONDITIONS										
PSORIASIS	0	0	0	0	0	0	0	0	0	0
RASHES, ERUPTIONS AND EXANTHEMS NEC										
RASH	2	1	1	0	0	2	1	1	0	0
RASH ERYTHEMATOUS	0	0	0	0	0	0	0	0	0	0
RASH MACULAR	0	0	0	0	0	0	0	0	0	0
RASH MORBILLIFORM	0	0	0	0	0	0	0	0	0	0
RASH PAPULAR	0	0	0	0	0	0	0	0	0	0
RASH PRURITIC	2	1	0	0	1	1	1	0	0	0
SKIN INJURIES AND MECHANICAL DERMATOSES										
DECUBITUS ULCER	0	0	0	0	0	0	0	0	0	0
NEEDLE TRACK MARKS	0	0	0	0	0	0	0	0	0	0
SKIN AND SUBCUTANEOUS TISSUE DISORDERS NEC										
SKIN AND SUBCUTANEOUS TISSUE ULCERATIONS										
SKIN EROSION	0	0	0	0	0	0	0	0	0	0

SKIN APPENDAGE CONDITIONS										
ACNES										
ACNE	0	0	0	0	0	0	0	0	0	0
ACNE CYSTIC	0	0	0	0	0	0	0	0	0	0
ALOPECIAS										
ALOPECIA	0	0	0	0	0	0	0	0	0	0
APOCRINE AND ECCRINE GLAND DISORDERS										
COLD SWEAT	1	1	0	0	0	1	1	0	0	0
HYPERHIDROSIS	2	2	0	0	0	2	2	0	0	0
MILIARIA	0	0	0	0	0	0	0	0	0	0
NIGHT SWEATS	0	0	0	0	0	0	0	0	0	0
HYPERTRICHOSES										
HIRSUTISM	0	0	0	0	0	0	0	0	0	0
NAIL AND NAIL BED CONDITIONS (EXCL INFECTIONS AND INFESTATIONS)										
NAIL DISCOLOURATION	0	0	0	0	0	0	0	0	0	0
ONYCHOCLASIS	0	0	0	0	0	0	0	0	0	0
PILAR DISORDERS NEC										
PILOERECTION	0	0	0	0	0	0	0	0	0	0
SKIN VASCULAR ABNORMALITIES										
PURPURA AND RELATED CONDITIONS										
PETECHIAE	0	0	0	0	0	0	0	0	0	0
PURPURA	0	0	0	0	0	0	0	0	0	0
SKIN HAEMORRHAGES										
SKIN HAEMORRHAGE	0	0	0	0	0	0	0	0	0	0
**SOCIAL CIRCUMSTANCES**										
ECONOMIC AND HOUSING ISSUES										
EMPLOYMENT ISSUES										
RETIREMENT	0	0	0	0	0	0	0	0	0	0
ENVIRONMENTAL ISSUES										
NON-OCCUPATIONAL AND UNSPECIFIED ENVIRONMENTAL PROBLEMS										
POLLUTION	0	0	0	0	0	0	0	0	0	0
**SURGICAL AND MEDICAL PROCEDURES**										
BONE AND JOINT THERAPEUTIC PROCEDURES										
JOINT THERAPEUTIC PROCEDURES										
KNEE OPERATION	0	0	0	0	0	0	0	0	0	0
BREAST THERAPEUTIC PROCEDURES										

MASTECTOMIES										
BREAST CONSERVING SURGERY	0	0	0	0	0	0	0	0	0	0
GASTROINTESTINAL THERAPEUTIC PROCEDURES										
GASTROINTESTINAL THERAPEUTIC PROCEDURES NEC										
PROPHYLAXIS OF NAUSEA AND VOMITING	0	0	0	0	0	0	0	0	0	0
HEAD AND NECK THERAPEUTIC PROCEDURES										
DENTAL AND GINGIVAL THERAPEUTIC PROCEDURES										
DENTAL CARE	0	0	0	0	0	0	0	0	0	0
NERVOUS SYSTEM, SKULL AND SPINE THERAPEUTIC PROCEDURES										
SKULL AND BRAIN THERAPEUTIC PROCEDURES										
BRAIN TUMOUR OPERATION	0	0	0	0	0	0	0	0	0	0
OBSTETRIC AND GYNAECOLOGICAL THERAPEUTIC PROCEDURES										
FERTILITY AND FERTILISATION INTERVENTIONS FEMALE										
ENDOMETRIAL SCRATCHING	0	0	0	0	0	0	0	0	0	0
RESPIRATORY TRACT THERAPEUTIC PROCEDURES										
RESPIRATORY TRACT THERAPEUTIC PROCEDURES NEC										
OXYGEN THERAPY	0	0	0	0	0	0	0	0	0	0
THERAPEUTIC PROCEDURES AND SUPPORTIVE CARE NEC										
ANAESTHESIA AND ALLIED PROCEDURES										
NERVE BLOCK	0	0	0	0	0	0	0	0	0	0
DIETARY AND NUTRITIONAL THERAPIES										
NOTHING BY MOUTH ORDER	0	0	0	0	0	0	0	0	0	0
IMMUNISATIONS										
COVID-19 IMMUNISATION	0	0	0	0	0	0	0	0	0	0
THERAPEUTIC PROCEDURES NEC										
INJECTION	0	0	0	0	0	0	0	0	0	0
LOCALISED ALTERNATING HOT AND COLD THERAPY	0	0	0	0	0	0	0	0	0	0
MASS EXCISION	0	0	0	0	0	0	0	0	0	0
REINFUSION	0	0	0	0	0	0	0	0	0	0
**VASCULAR DISORDERS**										
ARTERIOSCLEROSIS, STENOSIS, VASCULAR INSUFFICIENCY AND NECROSIS										
NON-SITE SPECIFIC NECROSIS AND VASCULAR INSUFFICIENCY NEC										
VASOSPASM	0	0	0	0	0	0	0	0	0	0
PERIPHERAL VASOCONSTRICTION, NECROSIS AND VASCULAR INSUFFICIENCY										
PERIPHERAL COLDNESS	0	0	0	0	0	0	0	0	0	0

RAYNAUD'S PHENOMENON	0	0	0	0	0	0	0	0	0	0
DECREASED AND NONSPECIFIC BLOOD PRESSURE DISORDERS AND SHOCK										
BLOOD PRESSURE DISORDERS NEC										
BLOOD PRESSURE FLUCTUATION	0	0	0	0	0	0	0	0	0	0
VASCULAR HYPOTENSIVE DISORDERS										
CAPILLARY LEAK SYNDROME	0	0	0	0	0	0	0	0	0	0
HYPOTENSION	0	0	0	0	0	0	0	0	0	0
ORTHOSTATIC HYPOTENSION	0	0	0	0	0	0	0	0	0	0
EMBOLISM AND THROMBOSIS										
NON-SITE SPECIFIC EMBOLISM AND THROMBOSIS										
EMBOLISM	0	0	0	0	0	0	0	0	0	0
THROMBOSIS	0	0	0	0	0	0	0	0	0	0
VENOUS THROMBOSIS	0	0	0	0	0	0	0	0	0	0
PERIPHERAL EMBOLISM AND THROMBOSIS										
BLUE TOE SYNDROME	0	0	0	0	0	0	0	0	0	0
DEEP VEIN THROMBOSIS	0	0	0	0	0	0	0	0	0	0
SUPERFICIAL VEIN THROMBOSIS	0	0	0	0	0	0	0	0	0	0
LYMPHATIC VESSEL DISORDERS										
LYMPHOEDEMAS										
LYMPHOEDEMA	0	0	0	0	0	0	0	0	0	0
VASCULAR DISORDERS NEC										
NON-SITE SPECIFIC VASCULAR DISORDERS NEC										
VASCULAR PAIN	0	0	0	0	0	0	0	0	0	0
VEIN DISCOLOURATION	0	0	0	0	0	0	0	0	0	0
VEIN RUPTURE	0	0	0	0	0	0	0	0	0	0
PERIPHERAL VASCULAR DISORDERS NEC										
FLUSHING	0	0	0	0	0	0	0	0	0	0
HOT FLUSH	2	1	0	1	0	2	1	0	1	0
SITE SPECIFIC VASCULAR DISORDERS NEC										
PALLOR	0	0	0	0	0	0	0	0	0	0
VASCULAR HAEMORRHAGIC DISORDERS										
HAEMORRHAGES NEC										
HAEMATOMA	0	0	0	0	0	0	0	0	0	0
HAEMORRHAGE	0	0	0	0	0	0	0	0	0	0
VASCULAR HYPERTENSIVE DISORDERS										
ACCELERATED AND MALIGNANT HYPERTENSION										

HYPERTENSIVE URGENCY	0	0	0	0	0	0	0	0	0	0
VASCULAR HYPERTENSIVE DISORDERS NEC										
HYPERTENSION	1	1	0	0	0	1	1	0	0	0
SYSTOLIC HYPERTENSION	0	0	0	0	0	0	0	0	0	0
VASCULAR INFECTIONS AND INFLAMMATIONS										
ARTERIAL INFECTIONS AND INFLAMMATIONS										
GIANT CELL ARTERITIS	0	0	0	0	0	0	0	0	0	0
PHLEBITIS NEC										
PHLEBITIS	0	0	0	0	0	0	0	0	0	0
VASCULITIDES NEC										
VASCULITIS	0	0	0	0	0	0	0	0	0	0
VENOUS VARICES										
VARICOSE VEINS NEC										
SPIDER VEIN	0	0	0	0	0	0	0	0	0	0
VARICOSE VEIN	0	0	0	0	0	0	0	0	0	0
**TOTAL ADR EVENTS**	***314***	***209***	***39***	***53***	***13***	***294***	***209***	***27***	***52***	***6***

SUPPLEMENTARY TABLE 20. COVID-19 vaccine ADR listings for events reported in the YCVM with no dose identification information in (a) those reporting any vaccination dose and (b) in those who had reported a 1st dose vaccination and any subsequent doses.

	Individuals with any vaccination dose: ADR Counts	Individuals reporting a 1st dose: ADR Counts
**MEDDRA REACTION TERM (SOC, *HLGT*, *HLT*, PT)**	Unknown	Unknown
(freetext)	3	3
**BLOOD AND LYMPHATIC SYSTEM DISORDERS**		
COAGULOPATHIES AND BLEEDING DIATHESES (EXCL THROMBOCYTOPENIC)		
BLEEDING TENDENCIES		
INCREASED TENDENCY TO BRUISE	1	1
HAEMOGLOBINOPATHIES		
SICKLE CELL TRAIT AND DISORDERS		
SICKLE CELL ANAEMIA WITH CRISIS	0	0
PLATELET DISORDERS		
THROMBOCYTOPENIAS		
IMMUNE THROMBOCYTOPENIA	0	0
SPLEEN, LYMPHATIC AND RETICULOENDOTHELIAL SYSTEM DISORDERS		
LYMPHATIC SYSTEM DISORDERS NEC		
LYMPH NODE PAIN	4	4
LYMPHADENITIS	0	0
LYMPHADENOPATHY	9	8
**CARDIAC DISORDERS**		
CARDIAC ARRHYTHMIAS		
RATE AND RHYTHM DISORDERS NEC		
ARRHYTHMIA	0	0
CARDIAC FLUTTER	0	0
EXTRASYSTOLES	0	0
TACHYCARDIA	0	0
SUPRAVENTRICULAR ARRHYTHMIAS		
ATRIAL FIBRILLATION	0	0
SUPRAVENTRICULAR TACHYCARDIA	0	0
CARDIAC DISORDERS, SIGNS AND SYMPTOMS NEC		
CARDIAC DISORDERS NEC		
CARDIOVASCULAR DISORDER	0	0
CARDIAC SIGNS AND SYMPTOMS NEC		
PALPITATIONS	10	8
CORONARY ARTERY DISORDERS		
ISCHAEMIC CORONARY ARTERY DISORDERS		
ANGINA PECTORIS	0	0
MYOCARDIAL INFARCTION	0	0
HEART FAILURES		
HEART FAILURES NEC		
CARDIAC FAILURE	0	0
MYOCARDIAL DISORDERS		
NONINFECTIOUS MYOCARDITIS		

MYOCARDITIS	2	2
PERICARDIAL DISORDERS		
NONINFECTIOUS PERICARDITIS		
PERICARDITIS	0	0
**CONGENITAL, FAMILIAL AND GENETIC DISORDERS**		
CARDIAC AND VASCULAR DISORDERS CONGENITAL		
CARDIAC DISORDERS CONGENITAL NEC		
HEART DISEASE CONGENITAL	0	0
METABOLIC AND NUTRITIONAL DISORDERS CONGENITAL		
INBORN ERRORS OF AMINO ACID METABOLISM		
HYPERGLYCINAEMIA	0	0
INBORN ERRORS OF STEROID SYNTHESIS		
11-BETA-HYDROXYLASE DEFICIENCY	0	0
NEUROLOGICAL DISORDERS CONGENITAL		
PERIPHERAL NERVOUS SYSTEM DISORDERS CONGENITAL NEC		
PAROXYSMAL EXTREME PAIN DISORDER	0	0
**EAR AND LABYRINTH DISORDERS**		
AURAL DISORDERS NEC		
EAR DISORDERS NEC		
EAR DISCOMFORT	0	0
EAR DISORDER	0	0
EAR PAIN	2	2
EAR SWELLING	0	0
EXTERNAL EAR DISORDERS (EXCL CONGENITAL)		
EXTERNAL EAR DISORDERS NEC		
EXCESSIVE CERUMEN PRODUCTION	0	0
HEARING DISORDERS		
HEARING LOSSES		
DEAFNESS	0	0
DEAFNESS NEUROSENSORY	0	0
DEAFNESS PERMANENT	0	0
DEAFNESS UNILATERAL	0	0
HYPOACUSIS	0	0
SUDDEN HEARING LOSS	0	0
HYPERACUSIA		
HYPERACUSIS	0	0
INNER EAR AND VIIITH CRANIAL NERVE DISORDERS		
INNER EAR SIGNS AND SYMPTOMS		
MOTION SICKNESS	0	0
TINNITUS	7	7
VERTIGO	3	3
VERTIGO LABYRINTHINE	0	0
VERTIGO POSITIONAL	0	0
**ENDOCRINE DISORDERS**		
THYROID GLAND DISORDERS		
THYROID HYPERFUNCTION DISORDERS		
HYPERTHYROIDISM	0	0
**EYE DISORDERS**		
EYE DISORDERS NEC		

LACRIMATION DISORDERS		
DRY EYE	0	0
LACRIMATION INCREASED	1	1
OCULAR DISORDERS NEC		
EYE OEDEMA	0	0
EYE PAIN	9	9
EYE SWELLING	0	0
EYE ULCER	0	0
EYELID PAIN	0	0
OCULAR DISCOMFORT	0	0
PERIORBITAL DISCOMFORT	0	0
PERIORBITAL SWELLING	0	0
OCULAR HAEMORRHAGES AND VASCULAR DISORDERS NEC		
CONJUNCTIVAL AND CORNEAL BLEEDING AND VASCULAR DISORDERS		
CONJUNCTIVAL HAEMORRHAGE	1	1
LID BLEEDING AND VASCULAR DISORDERS		
EYELID BLEEDING	0	0
OCULAR INFECTIONS, IRRITATIONS AND INFLAMMATIONS		
LID, LASH AND LACRIMAL INFECTIONS, IRRITATIONS AND INFLAMMATIONS		
BLEPHARITIS	0	0
ERYTHEMA OF EYELID	0	0
EYELID IRRITATION	0	0
OCULAR INFECTIONS, INFLAMMATIONS AND ASSOCIATED MANIFESTATIONS		
EYE DISCHARGE	0	0
EYE IRRITATION	0	0
EYE PRURITUS	0	0
LIMBAL SWELLING	0	0
OCULAR HYPERAEMIA	0	0
OCULAR NEUROMUSCULAR DISORDERS		
EYELID MOVEMENT DISORDERS		
BLEPHAROSPASM	0	0
OCULAR SENSORY SYMPTOMS NEC		
OCULAR SENSATION DISORDERS		
ABNORMAL SENSATION IN EYE	0	0
ASTHENOPIA	2	2
FOREIGN BODY SENSATION IN EYES	0	0
PHOTOPHOBIA	2	2
OCULAR STRUCTURAL CHANGE, DEPOSIT AND DEGENERATION NEC		
CHOROID AND VITREOUS STRUCTURAL CHANGE, DEPOSIT AND DEGENERATION		
VITREOUS DETACHMENT	0	0
VITREOUS FLOATERS	0	0
RETINA, CHOROID AND VITREOUS HAEMORRHAGES AND VASCULAR DISORDERS		
RETINAL BLEEDING AND VASCULAR DISORDERS (EXCL RETINOPATHY)		
RETINAL VEIN OCCLUSION	0	0
RETINOPATHIES NEC		
RETINAL EXUDATES	0	0

VISION DISORDERS		
VISUAL DISORDERS NEC		
DIPLOPIA	1	1
HALO VISION	0	0
METAMORPHOPSIA	0	0
PHOTOPSIA	2	2
VISION BLURRED	1	0
VISUAL IMPAIRMENT AND BLINDNESS (EXCL COLOUR BLINDNESS)		
BLINDNESS	0	0
BLINDNESS TRANSIENT	0	0
SUDDEN VISUAL LOSS	0	0
VISUAL IMPAIRMENT	0	0
**GASTROINTESTINAL DISORDERS**		
ANAL AND RECTAL CONDITIONS NEC		
ANAL AND RECTAL SIGNS AND SYMPTOMS		
ANAL PARAESTHESIA	0	0
BENIGN NEOPLASMS GASTROINTESTINAL		
BENIGN ORAL CAVITY NEOPLASMS		
MOUTH CYST	0	0
DENTAL AND GINGIVAL CONDITIONS		
DENTAL DISORDERS NEC		
TEETHING	0	0
DENTAL PAIN AND SENSATION DISORDERS		
DENTAL PARAESTHESIA	0	0
TOOTHACHE	0	0
GINGIVAL DISORDERS, SIGNS AND SYMPTOMS NEC		
GINGIVAL BLISTER	0	0
GINGIVAL PAIN	0	0
GINGIVAL SWELLING	0	0
GASTROINTESTINAL CONDITIONS NEC		
GASTROINTESTINAL MUCOSAL DYSTROPHIES AND SECRETION DISORDERS		
BARRETT'S OESOPHAGUS	0	0
GASTROINTESTINAL HAEMORRHAGES NEC		
INTESTINAL HAEMORRHAGES		
RECTAL HAEMORRHAGE	0	0
NON-SITE SPECIFIC GASTROINTESTINAL HAEMORRHAGES		
HAEMATEMESIS	0	0
GASTROINTESTINAL INFLAMMATORY CONDITIONS		
COLITIS (EXCL INFECTIVE)		
COLITIS	0	0
COLITIS MICROSCOPIC	0	0
COLITIS ULCERATIVE	0	0
GASTRITIS (EXCL INFECTIVE)		
GASTRITIS	0	0
REFLUX GASTRITIS	0	0
GASTROINTESTINAL INFLAMMATORY DISORDERS NEC		
GASTROINTESTINAL TRACT IRRITATION	0	0
GASTROINTESTINAL MOTILITY AND DEFAECATION CONDITIONS		
DIARRHOEA (EXCL INFECTIVE)		
DIARRHOEA	13	12
GASTROINTESTINAL ATONIC AND HYPOMOTILITY DISORDERS NEC		
CONSTIPATION	0	0

GASTROOESOPHAGEAL REFLUX DISEASE	1	1
GASTROINTESTINAL DYSKINETIC DISORDERS		
CHANGE OF BOWEL HABIT	0	0
GASTROINTESTINAL SPASTIC AND HYPERMOTILITY DISORDERS		
IRRITABLE BOWEL SYNDROME	0	0
GASTROINTESTINAL SIGNS AND SYMPTOMS		
DYSPEPTIC SIGNS AND SYMPTOMS		
DYSPEPSIA	2	1
ERUCTATION	0	0
FAECAL ABNORMALITIES NEC		
FAECALOMA	0	0
FAECES DISCOLOURED	0	0
FAECES SOFT	0	0
FLATULENCE, BLOATING AND DISTENSION		
ABDOMINAL DISTENSION	0	0
FLATULENCE	0	0
GASTROINTESTINAL AND ABDOMINAL PAINS (EXCL ORAL AND THROAT)		
ABDOMINAL PAIN	3	3
ABDOMINAL PAIN LOWER	0	0
ABDOMINAL PAIN UPPER	10	10
GASTROINTESTINAL PAIN	0	0
GASTROINTESTINAL SIGNS AND SYMPTOMS NEC		
ABDOMINAL DISCOMFORT	4	3
ABDOMINAL SYMPTOM	0	0
ACUTE ABDOMEN	0	0
ANAL INCONTINENCE	0	0
BREATH ODOUR	0	0
DYSPHAGIA	0	0
ODYNOPHAGIA	0	0
NAUSEA AND VOMITING SYMPTOMS		
NAUSEA	79	75
RETCHING	0	0
VOMITING	17	17
VOMITING PROJECTILE	0	0
MALABSORPTION CONDITIONS		
MALABSORPTION SYNDROMES		
COELIAC DISEASE	0	0
ORAL SOFT TISSUE CONDITIONS		
ORAL SOFT TISSUE DISORDERS NEC		
CHEILITIS	0	0
LIP BLISTER	0	0
ORAL LICHEN PLANUS	0	0
ORAL SOFT TISSUE INFECTIONS		
ANGULAR CHEILITIS	0	0
ORAL SOFT TISSUE SIGNS AND SYMPTOMS		
HYPOAESTHESIA ORAL	0	0
LIP PAIN	0	0
ORAL DISCOMFORT	0	0
ORAL MUCOSAL ROUGHENING	0	0
ORAL PAIN	0	0
PARAESTHESIA ORAL	1	1
LIP SWELLING	2	2

MOUTH SWELLING	0	0
STOMATITIS AND ULCERATION		
APHTHOUS ULCER	0	0
LIP ULCERATION	0	0
MOUTH ULCERATION	0	0
STOMATITIS	0	0
SALIVARY GLAND CONDITIONS		
ORAL DRYNESS AND SALIVA ALTERED		
DRY MOUTH	7	7
LIP DRY	1	1
SALIVARY HYPOSECRETION	0	0
TONGUE CONDITIONS		
TONGUE SIGNS AND SYMPTOMS		
GLOSSODYNIA	0	0
SWOLLEN TONGUE	1	1
TONGUE COATED	0	0
TONGUE DISCOMFORT	0	0
TONGUE OEDEMA	0	0
TONGUE SPASM	0	0
**GENERAL DISORDERS AND ADMINISTRATION SITE CONDITIONS**		
ADMINISTRATION SITE REACTIONS		
ADMINISTRATION SITE REACTIONS NEC		
ADMINISTRATION SITE BRUISE	0	0
ADMINISTRATION SITE PAIN	0	0
PUNCTURE SITE BRUISE	3	3
PUNCTURE SITE PAIN	1	1
APPLICATION SITE REACTIONS		
APPLICATION SITE BRUISE	0	0
APPLICATION SITE ERYTHEMA	0	0
APPLICATION SITE PAIN	0	0
IMPLANT AND CATHETER SITE REACTIONS		
IMPLANT SITE PAIN	0	0
IMPLANT SITE WARMTH	0	0
INFUSION SITE REACTIONS		
INFUSION SITE PAIN	0	0
INFUSION SITE SCAB	0	0
INFUSION SITE WARMTH	0	0
INJECTION SITE REACTIONS		
INJECTION SITE BRUISING	0	0
INJECTION SITE DISCOMFORT	0	0
INJECTION SITE ERYTHEMA	1	1
INJECTION SITE HYPERSENSITIVITY	0	0
INJECTION SITE INFLAMMATION	1	1
INJECTION SITE INJURY	0	0
INJECTION SITE IRRITATION	0	0
INJECTION SITE JOINT PAIN	0	0
INJECTION SITE MASS	2	2
INJECTION SITE NODULE	0	0
INJECTION SITE OEDEMA	0	0
INJECTION SITE PAIN	21	21
INJECTION SITE PAPULE	0	0
INJECTION SITE PARAESTHESIA	0	0

INJECTION SITE PRURITUS	0	0
INJECTION SITE RASH	0	0
INJECTION SITE REACTION	0	0
INJECTION SITE SCAB	0	0
INJECTION SITE SWELLING	2	2
INJECTION SITE URTICARIA	0	0
INJECTION SITE WARMTH	0	0
INSTILLATION SITE REACTIONS		
INSTILLATION SITE PRURITUS	0	0
INSTILLATION SITE WARMTH	1	1
VACCINATION SITE REACTIONS		
SHOULDER INJURY RELATED TO VACCINE ADMINISTRATION	0	0
VACCINATION SITE BRUISING	0	0
VACCINATION SITE DISCOMFORT	0	0
VACCINATION SITE ERYTHEMA	1	1
VACCINATION SITE JOINT ERYTHEMA	0	0
VACCINATION SITE JOINT PAIN	5	5
VACCINATION SITE MASS	1	1
VACCINATION SITE PAIN	14	13
VACCINATION SITE RASH	1	1
VACCINATION SITE SWELLING	2	2
VACCINATION SITE WARMTH	1	1
BODY TEMPERATURE CONDITIONS		
BODY TEMPERATURE ALTERED		
HYPERTHERMIA	0	0
HYPOTHERMIA	0	0
FEBRILE DISORDERS		
PYREXIA	100	99
FATAL OUTCOMES		
DEATH AND SUDDEN DEATH		
DEATH	0	0
GENERAL SYSTEM DISORDERS NEC		
ADVERSE EFFECT ABSENT		
NO ADVERSE EVENT	1	1
ASTHENIC CONDITIONS		
ASTHENIA	9	8
CHRONIC FATIGUE SYNDROME	0	0
DECREASED ACTIVITY	0	0
FATIGUE	257	245
MALAISE	24	23
SLUGGISHNESS	0	0
FEELINGS AND SENSATIONS NEC		
CHILLS	83	79
FEELING ABNORMAL	9	9
FEELING COLD	12	11
FEELING HOT	4	4
FEELING JITTERY	0	0
FEELING OF BODY TEMPERATURE CHANGE	3	3
FEELING OF RELAXATION	0	0
HANGOVER	2	2
HUNGER	2	2

SENSATION OF BLOOD FLOW	0	0
THIRST	1	1
GAIT DISTURBANCES		
GAIT DISTURBANCE	3	3
GAIT INABILITY	0	0
GENERAL SIGNS AND SYMPTOMS NEC		
CRYING	0	0
ENERGY INCREASED	0	0
EXERCISE TOLERANCE DECREASED	1	1
GENERAL SYMPTOM	0	0
ILLNESS	11	11
INFLUENZA LIKE ILLNESS	37	35
LOCAL REACTION	0	0
PERIPHERAL SWELLING	12	12
SWELLING	4	3
SWELLING FACE	2	2
TISSUE IRRITATION	0	0
INFLAMMATIONS		
INFLAMMATION	0	0
SYSTEMIC INFLAMMATORY RESPONSE SYNDROME	0	0
OEDEMA NEC		
OEDEMA	0	0
OEDEMA PERIPHERAL	0	0
PAIN AND DISCOMFORT NEC		
AXILLARY PAIN	1	1
CHEST DISCOMFORT	2	2
CHEST PAIN	6	5
DISCOMFORT	2	2
FACIAL PAIN	0	0
HERNIA PAIN	0	0
INFLAMMATORY PAIN	0	0
NON-CARDIAC CHEST PAIN	0	0
PAIN	49	47
TENDERNESS	20	19
THERAPEUTIC AND NONTHERAPEUTIC EFFECTS (EXCL TOXICITY)		
THERAPEUTIC AND NONTHERAPEUTIC RESPONSES		
ADVERSE DRUG REACTION	0	0
ADVERSE EVENT	5	4
ADVERSE REACTION	0	0
IMMEDIATE POST-INJECTION REACTION	0	0
**HEPATOBILIARY DISORDERS**		
HEPATIC AND HEPATOBILIARY DISORDERS		
HEPATOBILIARY SIGNS AND SYMPTOMS		
HEPATIC PAIN	0	0
HEPATOCELLULAR DAMAGE AND HEPATITIS NEC		
LIVER INJURY	0	0
**IMMUNE SYSTEM DISORDERS**		
ALLERGIC CONDITIONS		
ALLERGIC CONDITIONS NEC		
HYPERSENSITIVITY	0	0
MULTIPLE ALLERGIES	0	0

ALLERGIES TO FOODS, FOOD ADDITIVES, DRUGS AND OTHER CHEMICALS		
ALLERGY TO CHEMICALS	0	0
ANAPHYLACTIC AND ANAPHYLACTOID RESPONSES		
ANAPHYLACTIC REACTION	0	0
ATOPIC DISORDERS		
SEASONAL ALLERGY	0	0
IMMUNE DISORDERS NEC		
IMMUNE AND ASSOCIATED CONDITIONS NEC		
BACILLE CALMETTE-GUERIN SCAR REACTIVATION		
SENSITISATION	0	0
**INFECTIONS AND INFESTATIONS**		
BACTERIAL INFECTIOUS DISORDERS		
BACTERIAL INFECTIONS NEC		
CELLULITIS	0	0
PERIORBITAL CELLULITIS	0	0
HELICOBACTER INFECTIONS		
HELICOBACTER GASTRITIS	0	0
STAPHYLOCOCCAL INFECTIONS		
FURUNCLE	1	1
FUNGAL INFECTIOUS DISORDERS		
CANDIDA INFECTIONS		
CANDIDA INFECTION	0	0
VULVOVAGINAL CANDIDIASIS	0	0
INFECTIONS - PATHOGEN UNSPECIFIED		
ABDOMINAL AND GASTROINTESTINAL INFECTIONS		
APPENDICITIS	0	0
DIARRHOEA INFECTIOUS	0	0
GASTROINTESTINAL INFECTION	0	0
BREAST INFECTIONS		
MASTITIS	0	0
CENTRAL NERVOUS SYSTEM AND SPINAL INFECTIONS		
MYELITIS	0	0
DENTAL AND ORAL SOFT TISSUE INFECTIONS		
ABSCESS ORAL	0	0
ORAL PUSTULE	0	0
PERICORONITIS	0	0
TOOTH ABSCESS	0	0
EAR INFECTIONS		
EAR INFECTION	1	1
LABYRINTHITIS	0	0
OTITIS EXTERNA	0	0
EYE AND EYELID INFECTIONS		
CONJUNCTIVITIS	0	0
HORDEOLUM	0	0
INFECTIONS NEC		
ABSCESS	0	0
INFECTION	1	1
INJECTION SITE INFECTION	0	0
LOCALISED INFECTION	1	0
WOUND INFECTION	0	0
LOWER RESPIRATORY TRACT AND LUNG INFECTIONS		
LOWER RESPIRATORY TRACT INFECTION	1	1

PNEUMONIA	2	2
MALE REPRODUCTIVE TRACT INFECTIONS		
ORCHITIS	0	0
SEPSIS, BACTERAEMIA, VIRAEMIA AND FUNGAEMIA NEC		
SEPTIC RASH	0	0
SKIN STRUCTURES AND SOFT TISSUE INFECTIONS		
INFECTED DERMAL CYST	0	0
INJECTION SITE PUSTULE	0	0
SKIN INFECTION	0	0
UPPER RESPIRATORY TRACT INFECTIONS		
LARYNGITIS	0	0
NASOPHARYNGITIS	9	8
PHARYNGITIS	0	0
RHINITIS	1	1
SINUSITIS	1	1
TONSILLITIS	1	1
URINARY TRACT INFECTIONS		
CYSTITIS	0	0
URINARY TRACT INFECTION	0	0
PROTOZOAL INFECTIOUS DISORDERS		
TRYPANOSOMAL INFECTIONS		
AFRICAN TRYPANOSOMIASIS	0	0
RICKETTSIAL INFECTIOUS DISORDERS		
COXIELLA INFECTIONS		
Q FEVER	0	0
VIRAL INFECTIOUS DISORDERS		
CORONAVIRUS INFECTIONS		
COVID-19	1	0
HERPES VIRAL INFECTIONS		
GENITAL HERPES	0	0
HERPES OPHTHALMIC	0	0
HERPES SIMPLEX	0	0
HERPES ZOSTER	2	2
NASAL HERPES	0	0
OPHTHALMIC HERPES ZOSTER	0	0
ORAL HERPES	1	1
INFLUENZA VIRAL INFECTIONS		
H2N2 INFLUENZA	0	0
INFLUENZA	22	21
RETROVIRAL INFECTIONS		
AIDS RELATED COMPLEX	0	0
VIRAL INFECTIONS NEC		
GASTROENTERITIS VIRAL	0	0
SWEATING FEVER	2	2
VESTIBULAR NEURONITIS	0	0
VIRAL DIARRHOEA	0	0
VIRAL RASH	0	0
VIRAL UPPER RESPIRATORY TRACT INFECTION	0	0
**INJURY, POISONING AND PROCEDURAL COMPLICATIONS**		
BONE AND JOINT INJURIES		
FRACTURES AND DISLOCATIONS NEC		
JOINT DISLOCATION	0	0

EXPOSURES, CHEMICAL INJURIES AND POISONING		
POISONING AND TOXICITY		
SYSTEMIC TOXICITY	0	0
TOXICITY TO VARIOUS AGENTS	0	0
INJURIES BY PHYSICAL AGENTS		
CONDITIONS CAUSED BY COLD		
CHILLBLAINS	1	1
HEAT INJURIES (EXCL THERMAL BURNS)		
HEAT EXHAUSTION	0	0
HEAT OEDEMA	0	0
RADIATION INJURIES		
SUNBURN	0	0
THERMAL BURNS		
THERMAL BURN	0	0
THERMAL BURNS OF EYE	0	0
INJURIES NEC		
CHEST AND RESPIRATORY TRACT INJURIES NEC		
CHEST CRUSHING	0	0
EYE INJURIES NEC		
EYE CONTUSION	0	0
MUSCLE, TENDON AND LIGAMENT INJURIES		
LIGAMENT SPRAIN	0	0
MUSCLE INJURY	0	0
MUSCLE STRAIN	0	0
NERVE INJURIES NEC		
NERVE INJURY	0	0
NON-SITE SPECIFIC INJURIES NEC		
ARTHROPOD STING	0	0
BITE	0	0
FALL	0	0
INFLAMMATION OF WOUND	0	0
TISSUE INJURY	0	0
WOUND COMPLICATION	0	0
WOUND SECRETION	0	0
SITE SPECIFIC INJURIES NEC		
LIMB INJURY	0	0
SKIN INJURIES NEC		
CONTUSION	12	11
SCAR	0	0
SKIN WOUND	0	0
PROCEDURAL RELATED INJURIES AND COMPLICATIONS NEC		
CARDIAC AND VASCULAR PROCEDURAL COMPLICATIONS		
CARDIAC PROCEDURE COMPLICATION	0	0
GASTROINTESTINAL AND HEPATOBILIARY PROCEDURAL COMPLICATIONS		
PROCEDURAL NAUSEA	0	0
NEUROLOGICAL AND PSYCHIATRIC PROCEDURAL COMPLICATIONS		
PROCEDURAL DIZZINESS	0	0
NON-SITE SPECIFIC PROCEDURAL COMPLICATIONS		
INCISION SITE PAIN	0	0
INCISION SITE SWELLING	0	0
INJECTION RELATED REACTION	0	0
POST PROCEDURAL COMPLICATION	0	0

PROCEDURAL PAIN	1	1
VACCINATION RELATED COMPLICATIONS		
IMMUNISATION REACTION	0	0
**INVESTIGATIONS**		
CARDIAC AND VASCULAR INVESTIGATIONS (EXCL ENZYME TESTS)		
HEART RATE AND PULSE INVESTIGATIONS		
HEART RATE	3	3
HEART RATE DECREASED	1	1
HEART RATE INCREASED	3	3
HEART RATE IRREGULAR	0	0
VASCULAR TESTS NEC (INCL BLOOD PRESSURE)		
BLOOD PRESSURE DECREASED	0	0
BLOOD PRESSURE INCREASED	0	0
BLOOD PRESSURE MEASUREMENT	1	1
ENDOCRINE INVESTIGATIONS (INCL SEX HORMONES)		
ENDOCRINE ANALYSES AND IMAGING NEC		
HORMONE LEVEL ABNORMAL	0	0
PITUITARY ANALYSES ANTERIOR		
BLOOD FOLLICLE STIMULATING HORMONE INCREASED	0	0
BLOOD LUTEINISING HORMONE	0	0
THYROID ANALYSES		
TRI-IODOTHYRONINE	0	0
HAEMATOLOGY INVESTIGATIONS (INCL BLOOD GROUPS)		
COAGULATION AND BLEEDING ANALYSES		
BLEEDING TIME	1	1
INTERNATIONAL NORMALISED RATIO DECREASED	0	0
PLATELET ANALYSES		
PLATELET COUNT INCREASED	1	1
RED BLOOD CELL ANALYSES		
HAEMOGLOBIN	0	0
METABOLIC, NUTRITIONAL AND BLOOD GAS INVESTIGATIONS		
BLOOD GAS AND ACID BASE ANALYSES		
OXYGEN SATURATION DECREASED	0	0
CARBOHYDRATE TOLERANCE ANALYSES (INCL DIABETES)		
BLOOD GLUCOSE	0	0
BLOOD GLUCOSE ABNORMAL	0	0
BLOOD GLUCOSE DECREASED	0	0
BLOOD GLUCOSE INCREASED	0	0
MICROBIOLOGY AND SEROLOGY INVESTIGATIONS		
VIRUS IDENTIFICATION AND SEROLOGY		
SARS-COV-2 TEST	0	0
SARS-COV-2 TEST POSITIVE	0	0
MUSCULOSKELETAL AND SOFT TISSUE INVESTIGATIONS (EXCL ENZYME TESTS)		
MUSCULOSKELETAL AND SOFT TISSUE IMAGING PROCEDURES		
BONE SCAN	0	0
NEUROLOGICAL, SPECIAL SENSES AND PSYCHIATRIC INVESTIGATIONS		
CENTRAL NERVOUS SYSTEM IMAGING PROCEDURES		
MAGNETIC RESONANCE IMAGING HEAD	0	0
SCAN BRAIN	0	0
PHYSICAL EXAMINATION AND ORGAN SYSTEM STATUS TOPICS		
PHYSICAL EXAMINATION PROCEDURES AND ORGAN SYSTEM STATUS		
BODY TEMPERATURE	9	9

BODY TEMPERATURE ABNORMAL	0	0
BODY TEMPERATURE DECREASED	0	0
BODY TEMPERATURE FLUCTUATION	0	0
BODY TEMPERATURE INCREASED	1	1
GRIP STRENGTH DECREASED	0	0
HEAD LAG	0	0
LEFT-HANDEDNESS	0	0
LYMPH NODE PALPABLE	0	0
RESPIRATORY RATE DECREASED	1	1
SKIN TEMPERATURE	0	0
WEIGHT DECREASED	0	0
WEIGHT INCREASED	1	1
RENAL AND URINARY TRACT INVESTIGATIONS AND URINALYSES		
URINALYSIS NEC		
BLOOD URINE	0	0
NITRITE URINE PRESENT	0	0
PH URINE	1	1
URINARY TRACT FUNCTION ANALYSES NEC		
URINE OUTPUT	0	0
URINE OUTPUT INCREASED	0	0
REPRODUCTIVE ORGAN AND BREAST INVESTIGATIONS (EXCL HORMONE ANALYSES)		
REPRODUCTIVE ORGAN AND BREAST IMAGING PROCEDURES		
BREAST SCAN	0	0
RESPIRATORY AND PULMONARY INVESTIGATIONS (EXCL BLOOD GASES)		
RESPIRATORY AND PULMONARY FUNCTION DIAGNOSTIC PROCEDURES		
FORCED EXPIRATORY VOLUME DECREASED	0	0
FORCED EXPIRATORY VOLUME INCREASED	0	0
WATER, ELECTROLYTE AND MINERAL INVESTIGATIONS		
WATER AND ELECTROLYTE ANALYSES NEC		
VOLUME BLOOD	0	0
**METABOLISM AND NUTRITION DISORDERS**		
APPETITE AND GENERAL NUTRITIONAL DISORDERS		
APPETITE DISORDERS		
APPETITE DISORDER	1	1
DECREASED APPETITE	15	14
FOOD CRAVING	0	0
FOOD REFUSAL	0	0
INCREASED APPETITE	1	1
GENERAL NUTRITIONAL DISORDERS NEC		
FOOD AVERSION	0	0
ELECTROLYTE AND FLUID BALANCE CONDITIONS		
TOTAL FLUID VOLUME DECREASED		
DEHYDRATION	0	0
TOTAL FLUID VOLUME INCREASED		
FLUID RETENTION	0	0
FOOD INTOLERANCE SYNDROMES		
FOOD MALABSORPTION AND INTOLERANCE SYNDROMES (EXCL SUGAR INTOLERANCE)		
ALCOHOL INTOLERANCE	0	0
GLUCOSE METABOLISM DISORDERS (INCL DIABETES MELLITUS)		
DIABETES MELLITUS (INCL SUBTYPES)		
DIABETES MELLITUS	0	0
DIABETES MELLITUS INADEQUATE CONTROL	1	1
HYPERGLYCAEMIC CONDITIONS NEC		

HYPERGLYCAEMIA	0	0
HYPOGLYCAEMIC CONDITIONS NEC		
HYPOGLYCAEMIA	0	0
PURINE AND PYRIMIDINE METABOLISM DISORDERS		
DISORDERS OF PURINE METABOLISM		
GOUT	0	0
**MUSCULOSKELETAL AND CONNECTIVE TISSUE DISORDERS**		
BONE DISORDERS (EXCL CONGENITAL AND FRACTURES)		
BONE DISORDERS NEC		
JAW DISORDER	0	0
OSTEITIS	0	0
BONE RELATED SIGNS AND SYMPTOMS		
BONE PAIN	1	1
BONE SWELLING	1	1
PAIN IN JAW	0	0
PUBIC PAIN	0	0
SPINAL PAIN	0	0
CONNECTIVE TISSUE DISORDERS (EXCL CONGENITAL)		
CONNECTIVE TISSUE DISORDERS NEC		
POLYMYALGIA RHEUMATICA	0	0
LUPUS ERYTHEMATOSUS (INCL SUBTYPES)		
SYSTEMIC LUPUS ERYTHEMATOSUS	0	0
JOINT DISORDERS		
ARTHROPATHIES NEC		
ARTHRITIS	1	0
ARTHROPATHY	0	0
POLYARTHRITIS	0	0
RHEUMATIC FEVER	0	0
JOINT RELATED DISORDERS NEC		
JOINT LOCK	1	1
PERIARTHRITIS	0	0
TEMPOROMANDIBULAR PAIN AND DYSFUNCTION SYNDROME	0	0
JOINT RELATED SIGNS AND SYMPTOMS		
ARTHRALGIA	60	54
JAW CLICKING	0	0
JOINT STIFFNESS	2	1
JOINT SWELLING	0	0
OSTEOARTHROPATHIES		
OSTEOARTHRITIS	0	0
PSORIATIC ARTHROPATHIES		
PSORIATIC ARTHROPATHY	0	0
RHEUMATOID ARTHROPATHIES		
RHEUMATOID ARTHRITIS	0	0
MUSCLE DISORDERS		
MUSCLE INFECTIONS AND INFLAMMATIONS		
MYOSITIS	0	0
MUSCLE PAINS		
FIBROMYALGIA	0	0
MYALGIA	65	61
MUSCLE RELATED SIGNS AND SYMPTOMS NEC		
MUSCLE ATROPHY	1	1
MUSCLE DISCOMFORT	0	0

MUSCLE FATIGUE	2	2
MUSCLE FIBROSIS	1	1
MUSCLE MASS	0	0
MUSCLE SPASMS	8	8
MUSCLE TIGHTNESS	0	0
MUSCLE TWITCHING	1	1
MUSCLE TONE ABNORMALITIES		
MUSCLE RIGIDITY	0	0
TRISMUS	0	0
MUSCLE WEAKNESS CONDITIONS		
MUSCULAR WEAKNESS	3	3
MUSCULOSKELETAL AND CONNECTIVE TISSUE DISORDERS NEC		
MUSCULOSKELETAL AND CONNECTIVE TISSUE CONDITIONS NEC		
MOBILITY DECREASED	0	0
MUSCULOSKELETAL STIFFNESS	3	3
BACK PAIN	13	12
FLANK PAIN	0	0
LIMB DISCOMFORT	28	28
MUSCULOSKELETAL CHEST PAIN	4	4
MUSCULOSKELETAL DISCOMFORT	0	0
MUSCULOSKELETAL PAIN	0	0
NECK PAIN	6	6
PAIN IN EXTREMITY	198	182
SOFT TISSUE DISORDERS NEC		
AXILLARY MASS	0	0
GROIN PAIN	1	1
SYNOVIAL AND BURSAL DISORDERS		
SYNOVIAL DISORDERS		
SYNOVITIS	0	0
TENDON, LIGAMENT AND CARTILAGE DISORDERS		
CARTILAGE DISORDERS		
COSTOCHONDRITIS	0	0
TENDON DISORDERS		
TENDONITIS	0	0
TENOSYNOVITIS	0	0
TRIGGER FINGER	0	0
**NEOPLASMS BENIGN, MALIGNANT AND UNSPECIFIED (INCL CYSTS AND POLYPS)**		
CUTANEOUS NEOPLASMS BENIGN		
SKIN NEOPLASMS BENIGN		
MELANOCYTIC NAEVUS	1	1
SEBORRHOEIC KERATOSIS	0	0
SKIN PAPILLOMA	0	0
**NERVOUS SYSTEM DISORDERS**		
CENTRAL NERVOUS SYSTEM INFECTIONS AND INFLAMMATIONS		
MYELITIS (INCL INFECTIVE)		
MYELITIS TRANSVERSE	0	0
CENTRAL NERVOUS SYSTEM VASCULAR DISORDERS		
CENTRAL NERVOUS SYSTEM HAEMORRHAGES AND CEREBROVASCULAR ACCIDENTS		
CEREBRAL HAEMORRHAGE	0	0
CEREBROVASCULAR ACCIDENT	0	0
TRANSIENT CEREBROVASCULAR EVENTS		
TRANSIENT ISCHAEMIC ATTACK	0	0

CRANIAL NERVE DISORDERS (EXCL NEOPLASMS)		
FACIAL CRANIAL NERVE DISORDERS		
BELL'S PALSY	0	0
FACIAL PARALYSIS	0	0
FACIAL PARESIS	0	0
OLFACTORY NERVE DISORDERS		
ANOSMIA	2	2
HYPOSMIA	0	0
PAROSMIA	0	0
TRIGEMINAL DISORDERS		
TRIGEMINAL NEURALGIA	0	0
TRIGEMINAL NEURITIS	0	0
DEMYELINATING DISORDERS		
MULTIPLE SCLEROSIS ACUTE AND PROGRESSIVE		
MULTIPLE SCLEROSIS RELAPSE	0	0
HEADACHES		
HEADACHES NEC		
CLUSTER HEADACHE	4	4
COLD-STIMULUS HEADACHE	0	0
DRUG WITHDRAWAL HEADACHE	0	0
HEADACHE	293	274
PRIMARY STABBING HEADACHE	0	0
SINUS HEADACHE	1	1
TENSION HEADACHE	2	2
THUNDERCLAP HEADACHE	0	0
VASCULAR HEADACHE	0	0
MIGRAINE HEADACHES		
MIGRAINE	6	6
MIGRAINE WITH AURA	0	0
RETINAL MIGRAINE	0	0
TYPICAL AURA WITHOUT HEADACHE	0	0
MENTAL IMPAIRMENT DISORDERS		
INTELLECTUAL DISABILITIES		
INTELLECTUAL DISABILITY	0	0
MEMORY LOSS (EXCL DEMENTIA)		
AMNESIA	1	1
MEMORY IMPAIRMENT	0	0
MENTAL IMPAIRMENT (EXCL DEMENTIA AND MEMORY LOSS)		
COGNITIVE DISORDER	0	0
DISTURBANCE IN ATTENTION	1	1
MENTAL IMPAIRMENT	0	0
MOVEMENT DISORDERS (INCL PARKINSONISM)		
DYSKINESIAS AND MOVEMENT DISORDERS NEC		
BRADYKINESIA	0	0
CLUMSINESS	0	0
DYSKINESIA	0	0
EXTRAPYRAMIDAL DISORDER	0	0
PSYCHOMOTOR HYPERACTIVITY	0	0
PARALYSIS AND PARESIS (EXCL CRANIAL NERVE)		
HEMIPLEGIA	1	1
MONOPARESIS	1	0
MONOPLEGIA	0	0

PARALYSIS	1	1
PARKINSON'S DISEASE AND PARKINSONISM		
FREEZING PHENOMENON	0	0
PARKINSON'S DISEASE	0	0
TREMOR (EXCL CONGENITAL)		
TREMOR	22	21
NEUROLOGICAL DISORDERS NEC		
COMA STATES		
DIABETIC HYPERGLYCAEMIC COMA	1	0
COORDINATION AND BALANCE DISTURBANCES		
BALANCE DISORDER	3	3
COORDINATION ABNORMAL	0	0
DYSSTASIA	0	0
VESTIBULAR NYSTAGMUS	0	0
DISTURBANCES IN CONSCIOUSNESS NEC		
DEPRESSED LEVEL OF CONSCIOUSNESS	1	1
LETHARGY	19	17
LOSS OF CONSCIOUSNESS	1	1
SEDATION	0	0
SOMNOLENCE	12	10
SYNCOPE	4	4
NERVOUS SYSTEM DISORDERS NEC		
NERVOUS SYSTEM DISORDER	1	1
NEUROLOGICAL SIGNS AND SYMPTOMS NEC		
AGITATION NEONATAL	1	0
BRAIN FOG	5	5
DIZZINESS	40	39
DIZZINESS EXERTIONAL	0	0
DIZZINESS POSTURAL	5	4
HEAD DISCOMFORT	1	0
INFANT IRRITABILITY	0	0
MENINGISM	0	0
MYOCLONUS	0	0
NEUROLOGICAL SYMPTOM	0	0
PERSISTENT POSTURAL-PERCEPTUAL DIZZINESS	0	0
PRESYNCOPE	0	0
PARAESTHESIAS AND DYSAESTHESIAS		
BURNING FEET SYNDROME	0	0
BURNING SENSATION	4	4
FORMICATION	0	0
HYPERAESTHESIA	0	0
HYPOAESTHESIA	3	2
PARAESTHESIA	11	11
REVERSED HOT-COLD SENSATION	0	0
SENSORY ABNORMALITIES NEC		
AGEUSIA	4	4
ALLODYNIA	0	0
DYSGEUSIA	1	1
HYPOGEUSIA	0	0
NEURALGIA	1	1
POST HERPETIC NEURALGIA	0	0

RESTLESS ARM SYNDROME	0	0
RESTLESS LEGS SYNDROME	1	1
SENSORY LOSS	0	0
TASTE DISORDER	1	1
SPEECH AND LANGUAGE ABNORMALITIES		
DYSARTHRIA	0	0
SPEECH DISORDER DEVELOPMENTAL	0	0
NEUROLOGICAL DISORDERS OF THE EYE		
NEUROLOGIC VISUAL PROBLEMS NEC		
TUNNEL VISION	0	0
NEUROMUSCULAR DISORDERS		
MUSCLE TONE ABNORMAL		
HYPOTONIA	0	0
STIFF LEG SYNDROME	0	0
NEUROMUSCULAR DISORDERS NEC		
MUSCLE SPASTICITY	0	0
PERIPHERAL NEUROPATHIES		
ACUTE POLYNEUROPATHIES		
GUILLAIN-BARRE SYNDROME	0	0
PERIPHERAL NEUROPATHIES NEC		
AXONAL NEUROPATHY	0	0
NEUROPATHY PERIPHERAL	0	0
SEIZURES (INCL SUBTYPES)		
SEIZURES AND SEIZURE DISORDERS NEC		
EPILEPSY	0	0
SEIZURE	0	0
SLEEP DISTURBANCES (INCL SUBTYPES)		
DISTURBANCES IN SLEEP PHASE RHYTHM		
CIRCADIAN RHYTHM SLEEP DISORDER	0	0
SLEEP DISTURBANCES NEC		
SLEEP DEFICIT	0	0
SPINAL CORD AND NERVE ROOT DISORDERS		
CERVICAL SPINAL CORD AND NERVE ROOT DISORDERS		
CERVICOBRACHIAL SYNDROME	0	0
LUMBAR SPINAL CORD AND NERVE ROOT DISORDERS		
CAUDA EQUINA SYNDROME	0	0
SCIATICA	1	1
**PREGNANCY, PUERPERIUM AND PERINATAL CONDITIONS**		
ABORTIONS AND STILLBIRTH		
ABORTIONS SPONTANEOUS		
ABORTION SPONTANEOUS	0	0
STILLBIRTH AND FOETAL DEATH		
FOETAL DEATH	0	0
FOETAL COMPLICATIONS		
FOETAL COMPLICATIONS NEC		
FOETAL DISORDER	0	0
FOETAL HYPOKINESIA	0	0
FOETAL GROWTH COMPLICATIONS		
FOETAL MACROSOMIA	0	0
MATERNAL COMPLICATIONS OF PREGNANCY		
MATERNAL COMPLICATIONS OF PREGNANCY NEC		
MORNING SICKNESS	1	0
PLACENTAL, AMNIOTIC AND CAVITY DISORDERS (EXCL HAEMORRHAGES)		

PLACENTAL ABNORMALITIES (EXCL NEOPLASMS)		
PLACENTAL INFARCTION	0	0
PREGNANCY, LABOUR, DELIVERY AND POSTPARTUM CONDITIONS		
NORMAL PREGNANCY, LABOUR AND DELIVERY		
PREGNANCY	1	0
UTERINE CONTRACTIONS DURING PREGNANCY	0	0
**PRODUCT ISSUES**		
DEVICE ISSUES		
DEVICE MALFUNCTION EVENTS NEC		
OVERSENSING	0	0
**PSYCHIATRIC DISORDERS**		
ANXIETY DISORDERS AND SYMPTOMS		
ANXIETY SYMPTOMS		
AGITATION	0	0
ANXIETY	4	4
NERVOUSNESS	2	2
TENSION	0	0
PANIC ATTACKS AND DISORDERS		
PANIC ATTACK	1	1
CHANGES IN PHYSICAL ACTIVITY		
INCREASED PHYSICAL ACTIVITY LEVELS		
RESTLESSNESS	1	1
STEREOTYPIES AND AUTOMATISMS		
BRUXISM	0	0
COGNITIVE AND ATTENTION DISORDERS AND DISTURBANCES		
COGNITIVE AND ATTENTION DISORDERS AND DISTURBANCES NEC		
DAYDREAMING	0	0
MENTAL FATIGUE	3	3
COMMUNICATION DISORDERS AND DISTURBANCES		
SPEECH ARTICULATION AND RHYTHM DISTURBANCES		
DYSPHEMIA	0	0
DELIRIA (INCL CONFUSION)		
CONFUSION AND DISORIENTATION		
CONFUSIONAL STATE	0	0
DISORIENTATION	1	1
DELIRIA		
DELIRIUM	1	1
DEPRESSED MOOD DISORDERS AND DISTURBANCES		
DEPRESSIVE DISORDERS		
DEPRESSION	0	0
MOOD ALTERATIONS WITH DEPRESSIVE SYMPTOMS		
DEPRESSED MOOD	0	0
TEARFULNESS	0	0
DISSOCIATIVE DISORDERS		
DISSOCIATIVE STATES		
DISSOCIATION	0	0
DISTURBANCES IN THINKING AND PERCEPTION		
DELUSIONAL SYMPTOMS		
DELUSION	1	1
HALLUCINATIONS (EXCL SLEEP-RELATED)		
HALLUCINATION	1	1
PERCEPTION DISTURBANCES NEC		
DEREALISATION	0	0

THINKING DISTURBANCES		
BRADYPHRENIA	0	0
THOUGHT BLOCKING	0	0
MOOD DISORDERS AND DISTURBANCES NEC		
AFFECT ALTERATIONS NEC		
AFFECT LABILITY	0	0
INAPPROPRIATE AFFECT	0	0
EMOTIONAL AND MOOD DISTURBANCES NEC		
ANGER	0	0
EMOTIONAL DISORDER	0	0
EMOTIONAL DISTRESS	0	0
EUPHORIC MOOD	0	0
IRRITABILITY	1	1
MOOD ALTERED	0	0
FLUCTUATING MOOD SYMPTOMS		
MOOD SWINGS	0	0
MOOD DISORDERS NEC		
APATHY	0	0
LISTLESS	0	0
PERSONALITY DISORDERS AND DISTURBANCES IN BEHAVIOUR		
BEHAVIOUR AND SOCIALISATION DISTURBANCES		
AGGRESSION	0	0
PSYCHIATRIC AND BEHAVIOURAL SYMPTOMS NEC		
PSYCHIATRIC SYMPTOMS NEC		
PSYCHIATRIC SYMPTOM	1	1
PSYCHIATRIC DISORDERS NEC		
MENTAL DISORDERS NEC		
MENTAL DISORDER	0	0
SCHIZOPHRENIA AND OTHER PSYCHOTIC DISORDERS		
PSYCHOTIC DISORDER NEC		
PSYCHOTIC DISORDER	0	0
SEXUAL DYSFUNCTIONS, DISTURBANCES AND GENDER IDENTITY DISORDERS		
SEXUAL DESIRE DISORDERS		
LOSS OF LIBIDO	0	0
SLEEP DISORDERS AND DISTURBANCES		
DISTURBANCES IN INITIATING AND MAINTAINING SLEEP		
INITIAL INSOMNIA	0	0
INSOMNIA	4	4
DYSSOMNIAS		
BREATHING-RELATED SLEEP DISORDER	0	0
POOR QUALITY SLEEP	0	0
PARASOMNIAS		
ABNORMAL DREAMS	3	3
EXPLODING HEAD SYNDROME	0	0
NIGHTMARE	1	1
SLEEP DISORDERS NEC		
SLEEP DISORDER	0	0
SOMATIC SYMPTOM AND RELATED DISORDERS		
SOMATIC SYMPTOM DISORDERS		
HABIT COUGH	0	0
SUICIDAL AND SELF-INJURIOUS BEHAVIOURS NEC		
SUICIDAL AND SELF-INJURIOUS BEHAVIOUR		
SUICIDAL IDEATION	0	0

**RENAL AND URINARY DISORDERS**		
RENAL DISORDERS (EXCL NEPHROPATHIES)		
RENAL FAILURE AND IMPAIRMENT		
RENAL FAILURE	0	0
URINARY TRACT SIGNS AND SYMPTOMS		
BLADDER AND URETHRAL SYMPTOMS		
BLADDER PAIN	1	1
MICTURITION URGENCY	0	0
POLLAKIURIA	1	1
URINARY INCONTINENCE	0	0
URINARY ABNORMALITIES		
HAEMATURIA	0	0
URINE ABNORMALITY	0	0
URINE ODOUR ABNORMAL	0	0
URINARY TRACT SIGNS AND SYMPTOMS NEC		
POLYURIA	0	0
RENAL PAIN	1	1
**REPRODUCTIVE SYSTEM AND BREAST DISORDERS**		
BREAST DISORDERS		
BREAST DISORDERS NEC		
BREAST MASS	1	1
NIPPLE ENLARGEMENT	0	0
BREAST SIGNS AND SYMPTOMS		
BREAST DISCHARGE	1	1
BREAST PAIN	2	2
BREAST SWELLING	0	0
BREAST TENDERNESS	0	0
NIPPLE PAIN	0	0
MENOPAUSE RELATED CONDITIONS		
MENOPAUSAL EFFECTS ON THE GENITOURINARY TRACT		
POSTMENOPAUSAL HAEMORRHAGE	0	0
MENSTRUAL CYCLE AND UTERINE BLEEDING DISORDERS		
MENSTRUATION AND UTERINE BLEEDING NEC		
DYSMENORRHOEA	3	3
INTERMENSTRUAL BLEEDING	1	1
MENSTRUAL DISORDER	1	1
MENSTRUATION IRREGULAR	7	6
PREMENSTRUAL PAIN	1	1
RETROGRADE MENSTRUATION	0	0
MENSTRUATION WITH DECREASED BLEEDING		
AMENORRHOEA	0	0
HYPOMENORRHOEA	3	3
MENSTRUATION DELAYED	12	11
OLIGOMENORRHOEA	1	1
MENSTRUATION WITH INCREASED BLEEDING		
HEAVY MENSTRUAL BLEEDING	9	7
POLYMENORRHOEA	0	0
PENILE AND SCROTAL DISORDERS (EXCL INFECTIONS AND INFLAMMATIONS)		
SCROTAL DISORDERS NEC		
SCROTAL SWELLING	0	0
REPRODUCTIVE TRACT DISORDERS NEC		
REPRODUCTIVE TRACT DISORDERS NEC (EXCL NEOPLASMS)		
GENITAL LESION	0	0

REPRODUCTIVE TRACT SIGNS AND SYMPTOMS NEC		
GENITAL DISCOMFORT	0	0
GENITAL PAIN	0	0
UTERINE, PELVIC AND BROAD LIGAMENT DISORDERS		
PELVIS AND BROAD LIGAMENT DISORDERS NEC		
ADNEXA UTERI PAIN	0	0
VULVOVAGINAL DISORDERS (EXCL INFECTIONS AND INFLAMMATIONS)		
VULVOVAGINAL DISORDERS NEC		
VAGINAL HAEMORRHAGE	0	0
VULVOVAGINAL SIGNS AND SYMPTOMS		
VAGINAL DISCHARGE	0	0
**RESPIRATORY, THORACIC AND MEDIASTINAL DISORDERS**		
BRONCHIAL DISORDERS (EXCL NEOPLASMS)		
BRONCHOSPASM AND OBSTRUCTION		
ASTHMA	0	0
WHEEZING	1	1
LOWER RESPIRATORY TRACT DISORDERS (EXCL OBSTRUCTION AND INFECTION)		
PULMONARY OEDEMAS		
PULMONARY CONGESTION	0	0
PULMONARY VASCULAR DISORDERS		
PULMONARY THROMBOTIC AND EMBOLIC CONDITIONS		
PULMONARY EMBOLISM	1	1
RESPIRATORY DISORDERS NEC		
BREATHING ABNORMALITIES		
DYSPNOEA	14	13
HYPERVENTILATION	0	0
HYPOPNOEA	1	1
IRREGULAR BREATHING	0	0
MOUTH BREATHING	0	0
RESPIRATION ABNORMAL	0	0
RESPIRATORY ARREST	0	0
RESPIRATORY FATIGUE	0	0
SLEEP APNOEA SYNDROME	0	0
COUGHING AND ASSOCIATED SYMPTOMS		
COUGH	6	6
PRODUCTIVE COUGH	2	1
RESPIRATORY TRACT DISORDERS NEC		
RESPIRATORY TRACT IRRITATION	0	0
RESPIRATORY TRACT SIGNS AND SYMPTOMS		
LOWER RESPIRATORY TRACT SIGNS AND SYMPTOMS		
PULMONARY PAIN	0	0
RESPIRATORY SIGNS AND SYMPTOMS NEC		
RESPIRATORY SYMPTOM	0	0
UPPER RESPIRATORY TRACT SIGNS AND SYMPTOMS		
APHONIA	0	0
CATARRH	1	1
DRY THROAT	1	1
DYSPHONIA	0	0
INCREASED UPPER AIRWAY SECRETION	0	0
INCREASED VISCOSITY OF UPPER RESPIRATORY SE	0	0
NASAL DISCOMFORT	0	0
OROPHARYNGEAL DISCOMFORT	0	0
OROPHARYNGEAL PAIN	11	11

PARANASAL SINUS DISCOMFORT	0	0
RHINALGIA	0	0
RHINORRHOEA	8	8
SINUS PAIN	0	0
SNEEZING	2	2
THROAT CLEARING	0	0
THROAT IRRITATION	1	1
THROAT TIGHTNESS	0	0
UPPER-AIRWAY COUGH SYNDROME	0	0
YAWNING	1	1
UPPER RESPIRATORY TRACT DISORDERS (EXCL INFECTIONS)		
NASAL CONGESTION AND INFLAMMATIONS		
NASAL CONGESTION	0	0
RHINITIS ALLERGIC	0	0
RHINITIS ATROPHIC	0	0
NASAL DISORDERS NEC		
EPISTAXIS	0	0
NASAL DRYNESS	0	0
PARANASAL SINUS DISORDERS (EXCL INFECTIONS AND NEOPLASMS)		
SINUS CONGESTION	0	0
PHARYNGEAL DISORDERS (EXCL INFECTIONS AND NEOPLASMS)		
PHARYNGEAL SWELLING	0	0
PHARYNGEAL ULCERATION	0	0
TONSILLAR ERYTHEMA	1	1
TRACHEAL DISORDERS (EXCL INFECTIONS AND NEOPLASMS)		
TRACHEAL PAIN	0	0
**SKIN AND SUBCUTANEOUS TISSUE DISORDERS**		
ANGIOEDEMA AND URTICARIA		
ANGIOEDEMAS		
ANGIOEDEMA	0	0
URTICARIAS		
COLD URTICARIA	0	0
SOLAR URTICARIA	0	0
URTICARIA	0	0
URTICARIA CHRONIC	1	1
CORNIFICATION AND DYSTROPHIC SKIN DISORDERS		
SKIN DYSTROPHIES		
HYPERTROPHIC SCAR	0	0
CUTANEOUS NEOPLASMS BENIGN		
SKIN CYSTS AND POLYPS		
DERMAL CYST	0	0
EPIDERMAL AND DERMAL CONDITIONS		
BULLOUS CONDITIONS		
BLISTER	1	1
BLOOD BLISTER	1	1
PEMPHIGOID	0	0
TOXIC EPIDERMAL NECROLYSIS	0	0
DERMAL AND EPIDERMAL CONDITIONS NEC		
DRY SKIN	4	3
PAIN OF SKIN	3	3
SENSITIVE SKIN	2	2
SKIN BURNING SENSATION	2	2

SKIN DISCOLOURATION	0	0
SKIN FRAGILITY	0	0
SKIN LESION	1	0
SKIN ODOUR ABNORMAL	0	0
SKIN REACTION	0	0
SKIN SENSITISATION	0	0
SKIN WARM	1	1
DERMATITIS AND ECZEMA		
DERMATITIS	0	0
DERMATITIS ALLERGIC	0	0
DERMATITIS ATOPIC	0	0
DERMATITIS CONTACT	0	0
ECZEMA	2	1
ECZEMA ASTEATOTIC	0	0
NEURODERMATITIS	0	0
SEBORRHOEIC DERMATITIS	0	0
SKIN IRRITATION	0	0
ERYTHEMAS		
ERYTHEMA	5	4
EXFOLIATIVE CONDITIONS		
SKIN EXFOLIATION	0	0
PAPULOSQUAMOUS CONDITIONS		
LICHEN PLANUS	0	0
PITYRIASIS ROSEA	0	0
PHOTOSENSITIVITY AND PHOTODERMATOSIS CONDITIONS		
PHOTOSENSITIVITY REACTION	0	0
PRURITUS NEC		
PRURITUS	13	12
PSORIATIC CONDITIONS		
PSORIASIS	1	1
RASHES, ERUPTIONS AND EXANTHEMS NEC		
RASH	4	4
RASH ERYTHEMATOUS	4	3
RASH MACULAR	0	0
RASH MORBILLIFORM	0	0
RASH PAPULAR	1	1
RASH PRURITIC	3	3
SKIN INJURIES AND MECHANICAL DERMATOSES		
DECUBITUS ULCER	0	0
NEEDLE TRACK MARKS	0	0
SKIN AND SUBCUTANEOUS TISSUE DISORDERS NEC		
SKIN AND SUBCUTANEOUS TISSUE ULCERATIONS		
SKIN EROSION	0	0
SKIN APPENDAGE CONDITIONS		
ACNES		
ACNE	0	0
ACNE CYSTIC	0	0
ALOPECIAS		
ALOPECIA	5	4
APOCRINE AND ECCRINE GLAND DISORDERS		
COLD SWEAT	0	0
HYPERHIDROSIS	19	19

MILIARIA	1	1
NIGHT SWEATS	3	3
HYPERTRICHOSES		
HIRSUTISM	0	0
NAIL AND NAIL BED CONDITIONS (EXCL INFECTIONS AND INFESTATIONS)		
NAIL DISCOLOURATION	1	1
ONYCHOCLASIS	1	0
PILAR DISORDERS NEC		
PILOERECTION	0	0
SKIN VASCULAR ABNORMALITIES		
PURPURA AND RELATED CONDITIONS		
PETECHIAE	0	0
PURPURA	0	0
SKIN HAEMORRHAGES		
SKIN HAEMORRHAGE	0	0
**SOCIAL CIRCUMSTANCES**		
ECONOMIC AND HOUSING ISSUES		
EMPLOYMENT ISSUES		
RETIREMENT	0	0
ENVIRONMENTAL ISSUES		
NON-OCCUPATIONAL AND UNSPECIFIED ENVIRONMENTAL PROBLEMS		
POLLUTION	0	0
**SURGICAL AND MEDICAL PROCEDURES**		
BONE AND JOINT THERAPEUTIC PROCEDURES		
JOINT THERAPEUTIC PROCEDURES		
KNEE OPERATION	0	0
BREAST THERAPEUTIC PROCEDURES		
MASTECTOMIES		
BREAST CONSERVING SURGERY	0	0
GASTROINTESTINAL THERAPEUTIC PROCEDURES		
GASTROINTESTINAL THERAPEUTIC PROCEDURES NEC		
PROPHYLAXIS OF NAUSEA AND VOMITING	0	0
HEAD AND NECK THERAPEUTIC PROCEDURES		
DENTAL AND GINGIVAL THERAPEUTIC PROCEDURES		
DENTAL CARE	0	0
NERVOUS SYSTEM, SKULL AND SPINE THERAPEUTIC PROCEDURES		
SKULL AND BRAIN THERAPEUTIC PROCEDURES		
BRAIN TUMOUR OPERATION	0	0
OBSTETRIC AND GYNAECOLOGICAL THERAPEUTIC PROCEDURES		
FERTILITY AND FERTILISATION INTERVENTIONS FEMALE		
ENDOMETRIAL SCRATCHING	0	0
RESPIRATORY TRACT THERAPEUTIC PROCEDURES		
RESPIRATORY TRACT THERAPEUTIC PROCEDURES NEC		
OXYGEN THERAPY	1	1
THERAPEUTIC PROCEDURES AND SUPPORTIVE CARE NEC		
ANAESTHESIA AND ALLIED PROCEDURES		
NERVE BLOCK	0	0
DIETARY AND NUTRITIONAL THERAPIES		
NOTHING BY MOUTH ORDER	1	1
IMMUNISATIONS		
COVID-19 IMMUNISATION	0	0
THERAPEUTIC PROCEDURES NEC		
INJECTION	0	0

LOCALISED ALTERNATING HOT AND COLD THERAPY	0	0
MASS EXCISION	0	0
REINFUSION	0	0
**VASCULAR DISORDERS**		
ARTERIOSCLEROSIS, STENOSIS, VASCULAR INSUFFICIENCY AND NECROSIS		
NON-SITE SPECIFIC NECROSIS AND VASCULAR INSUFFICIENCY NEC		
VASOSPASM	0	0
PERIPHERAL VASOCONSTRICTION, NECROSIS AND VASCULAR INSUFFICIENCY		
PERIPHERAL COLDNESS	2	2
RAYNAUD'S PHENOMENON	0	0
DECREASED AND NONSPECIFIC BLOOD PRESSURE DISORDERS AND SHOCK		
BLOOD PRESSURE DISORDERS NEC		
BLOOD PRESSURE FLUCTUATION	0	0
VASCULAR HYPOTENSIVE DISORDERS		
CAPILLARY LEAK SYNDROME	0	0
HYPOTENSION	0	0
ORTHOSTATIC HYPOTENSION	0	0
EMBOLISM AND THROMBOSIS		
NON-SITE SPECIFIC EMBOLISM AND THROMBOSIS		
EMBOLISM	0	0
THROMBOSIS	1	1
VENOUS THROMBOSIS	0	0
PERIPHERAL EMBOLISM AND THROMBOSIS		
BLUE TOE SYNDROME	0	0
DEEP VEIN THROMBOSIS	1	1
SUPERFICIAL VEIN THROMBOSIS	1	1
LYMPHATIC VESSEL DISORDERS		
LYMPHOEDEMAS		
LYMPHOEDEMA	0	0
VASCULAR DISORDERS NEC		
NON-SITE SPECIFIC VASCULAR DISORDERS NEC		
VASCULAR PAIN	0	0
VEIN DISCOLOURATION	1	1
VEIN RUPTURE	0	0
PERIPHERAL VASCULAR DISORDERS NEC		
FLUSHING	1	1
HOT FLUSH	8	8
SITE SPECIFIC VASCULAR DISORDERS NEC		
PALLOR	2	2
VASCULAR HAEMORRHAGIC DISORDERS		
HAEMORRHAGES NEC		
HAEMATOMA	0	0
HAEMORRHAGE	1	1
VASCULAR HYPERTENSIVE DISORDERS		
ACCELERATED AND MALIGNANT HYPERTENSION		
HYPERTENSIVE URGENCY	0	0
VASCULAR HYPERTENSIVE DISORDERS NEC		
HYPERTENSION	1	1
SYSTOLIC HYPERTENSION	0	0
VASCULAR INFECTIONS AND INFLAMMATIONS		
ARTERIAL INFECTIONS AND INFLAMMATIONS		
GIANT CELL ARTERITIS	0	0

PHLEBITIS NEC		
PHLEBITIS	0	0
VASCULITIDES NEC		
VASCULITIS	0	0
VENOUS VARICES		
VARICOSE VEINS NEC		
SPIDER VEIN	0	0
VARICOSE VEIN	0	0
**TOTAL ADR EVENTS**	***2102***	***1980***
