# Supplementary material for: Implementation and Results of Active Vaccine Safety Monitoring During the COVID-19 Pandemic in the UK: A Regulatory Perspective
Source: Drug Saf. 2025 Sep 3;48(12):1365–85. doi: 10.1007/s40264-025-01579-w (PMC12605443; doi:10.1007/s40264-025-01579-w)
Supplement: Supplementary file 9 — Supplementary file9 (PDF 661 KB) [file 40264_2025_1579_MOESM9_ESM.pdf]

Online Resource 9

Electronic Supplementary material

Article Title: Implementation and results of active vaccine safety monitoring during the COVID-19 pandemic in the UK: a regulatory perspective

Journal for Submission: Drug Safety (Springer Nature)

Authors: Jenny Wong, Katherine Donegan, Kendal Harrison, Tahira Jan, Alison Cave, and Phil Tregunno

Author Affiliation: Medicines and Healthcare products Regulatory Agency, London, UK

Corresponding Author: Phil Tregunno, phil.tregunno@mhra.gov.uk

Adverse Reactions Reported in Individuals aged under 40 years versus Individuals aged 40 years or over.

Due to the approach taken to rollout the national vaccination campaign to the UK population, the brand and dose analysis is confounded by age. COVID-19 vaccination was prioritised by descending age groups, with the older population being the first to receive Pfizer and AstraZeneca before the younger population. Additionally, the supply availability of different vaccine brands over time would have impacted which vaccine an individual would receive. The brand of vaccine recommended would also be dependent on the age of the individual or if they were clinically vulnerable. The AstraZeneca vaccine was not recommended for use in those aged under 40 years after early May 2021 and therefore there could be differences in the ADR reporting profile between those aged under 40 years and those aged 40 years or older.

In the age-stratified analysis exploring the ADRs reported in those aged under 40 years and in those aged 40 years or older, any differences in the ADR reporting patterns overall between the age groups remained small (Supplementary Table 21).

Supplementary Table 21. Top 10 Adverse Drug Reactions (ADRs) reported by Individuals aged under 40 years compared against those aged 40 years and older.

Dose	Individuals Aged under 40 years	Individuals Aged 40 years and older

	Ranked ADR (MedDRA ® PT) reported	Number of ADRs reported	Percentage of ADRs reported for each dose	Ranked ADR (MedDRA ® PT) reported	Number of ADRs reported	Percentage of ADRs reported for each dose
First dose	Pain in extremity	945	15.3%	Headache	3,033	15.2%
	Headache	730	11.8%	Fatigue	2,323	11.7%
	Fatigue	621	10.1%	Pain in extremity	1,464	7.4%
	Pyrexia	439	7.1%	Pyrexia	1,232	6.2%
	Myalgia	294	4.8%	Chills	1,131	5.7%
	Nausea	240	3.9%	Myalgia	1,000	5.0%
	Pain	219	3.5%	Arthralgia	805	4.0%
	Chills	209	3.4%	Nausea	753	3.8%
	Arthralgia	151	2.4%	Pain	507	2.5%
	Injection site pain	147	2.4%	Influenza	413	2.1%
	TOTAL REACTIONS	***6,179***		***TOTAL REACTIONS***	***19,905***	
Second dose	Fatigue	136	12.7%	Fatigue	495	15.3%
	Headache	120	11.2%	Headache	426	13.2%
	Pain in extremity	108	10.1%	Pain in extremity	345	10.7%
	Pyrexia	91	8.5%	Myalgia	167	5.2%
	Pain	52	4.8%	Arthralgia	120	3.7%
	Nausea	37	3.4%	Pyrexia	110	3.4%
	Myalgia	34	3.2%	Chills	91	2.8%
	Chills	31	2.9%	Nausea	87	2.7%
	Arthralgia	29	2.7%	Limb discomfort	82	2.5%
	Dizziness	26	2.4%	Pain	78	2.4%
	TOTAL REACTIONS	***1,073***		***TOTAL REACTIONS***	***3,238***	
Third dose	Headache	48	12.8%	Pain in extremity	360	14.6%
	Fatigue	47	12.6%	Headache	289	11.8%
	Pain in extremity	40	10.7%	Fatigue	255	10.4%
	Pyrexia	27	7.2%	Pyrexia	107	4.4%
	Chills	22	5.9%	Arthralgia	107	4.4%
	Myalgia	18	4.8%	Chills	104	4.2%
	Nausea	16	4.3%	Myalgia	96	3.9%
	Pain	13	3.5%	Nausea	82	3.3%
	Injection site pain	8	2.1%	Pain	65	2.6%
	Influenza	8	2.1%	Limb discomfort	62	2.5%
	TOTAL REACTIONS	***374***		***TOTAL REACTIONS***	***2,459***	

Abbreviations: *ADR* Adverse Drug Reaction, *MedDRA* Medical Dictionary for Regulatory Activities, *PT* Preferred Term
