# Supplementary material for: Implementation and Results of Active Vaccine Safety Monitoring During the COVID-19 Pandemic in the UK: A Regulatory Perspective
Source: Drug Saf. 2025 Sep 3;48(12):1365–85. doi: 10.1007/s40264-025-01579-w (PMC12605443; doi:10.1007/s40264-025-01579-w)
Supplement: Supplementary file 10 — Supplementary file10 (PDF 780 KB) [file 40264_2025_1579_MOESM10_ESM.pdf]

Online Resource 10

Electronic Supplementary material

Article Title: Implementation and results of active vaccine safety monitoring during the COVID-19 pandemic in the UK: a regulatory perspective

Journal for Submission: Drug Safety (Springer Nature)

Authors: Jenny Wong, Katherine Donegan, Kendal Harrison, Tahira Jan, Alison Cave, and Phil Tregunno

Author Affiliation: Medicines and Healthcare products Regulatory Agency, London, UK

Corresponding Author: Phil Tregunno, phil.tregunno@mhra.gov.uk

Demographics of Registered Females

Supplementary Table 22. Demographics of registered females: female individuals, those reporting being pregnant or breastfeeding at the time of a vaccination.

	Female cohort (n=20,868)	Pregnant female cohort (n=2,517)	Breastfeeding cohort (n=694)
**Age bands (years)**			
Under 12	90	0	0
12-17	1,217	2	1
18-29	1,280	417	81
30-39	3,424	1,937	523
40-49	2,089	159	88
50-59	2,749	2	1
60-69	3,760	0	0
70-79	5,025	0	0
80+	1,221	0	0
Unknown	13	0	0
**Ethnicity**			
White British, White Irish, or any other white background	17,757	1,979	572
Other	1,480	262	78
Unknown	1,631	276	44
**BMI Category**			
Underweight	541	27	5
Normal	6,367	722	222

Overweight	4,430	506	161
Obese	2,908	279	85
Unknown	6,622	983	221
**Immunocompromised**			
Yes	2,195	114	45
No	18,673	2,403	649

Abbreviations: *BMI* Body Mass Index
