# Supplementary material for: Implementation and Results of Active Vaccine Safety Monitoring During the COVID-19 Pandemic in the UK: A Regulatory Perspective
Source: Drug Saf. 2025 Sep 3;48(12):1365–85. doi: 10.1007/s40264-025-01579-w (PMC12605443; doi:10.1007/s40264-025-01579-w)
Supplement: Supplementary file 11 — Supplementary file11 (PDF 4718 KB) [file 40264_2025_1579_MOESM11_ESM.pdf]

Online Resource 11

Electronic Supplementary material

Article Title: Implementation and results of active vaccine safety monitoring during the COVID-19 pandemic in the UK: a regulatory perspective

Journal for Submission: Drug Safety (Springer Nature)

Authors: Jenny Wong, Katherine Donegan, Kendal Harrison, Tahira Jan, Alison Cave, and Phil Tregunno

Author Affiliation: Medicines and Healthcare products Regulatory Agency, London, UK

Corresponding Author: Phil Tregunno, phil.tregunno@mhra.gov.uk

Commonly Reported Adverse Reactions (ADRs) in Pregnant Females

Supplementary Table 23. Most commonly reported ADRs by vaccine dose in the cohort of pregnant females.

Dose	Ranked ADR reported (MedDRA PT level)	Number of ADR reported (% of total ADRs per dose)	
First dose	Pain in extremity	302	(24.1)
	Fatigue	167	(13.3)
	Headache	134	(10.7)
	Pyrexia	74	(5.9)
	Myalgia	50	(4.0)
	Nausea	43	(3.4)
	Injection site pain	36	(2.9)
	Chills	35	(2.8)
	Pain	35	(2.8)
	Limb discomfort	34	(2.7)
	Total ADRs	***1,255***	
Second dose	Fatigue	74	(15.5)
	Headache	58	(12.2)
	Pain in extremity	54	(11.3)
	Pyrexia	33	(6.9)
	Nausea	23	(4.8)
	Pain	22	(4.6)
	Myalgia	21	(4.4)
	Chills	16	(3.4)
	Arthralgia	12	(2.5)
	Dizziness	11	(2.3)
	Total ADRs	***476***	
Third dose	Fatigue	17	(14.4)
	Pain in extremity	16	(13.6)

	Headache	14	(11.9)
	Pyrexia	8	(6.8)
	Chills	7	(5.9)
	Myalgia	5	(4.2)
	Pain	5	(4.2)
	Nausea	4	(3.4)
	Diarrhoea	3	(2.5)
	Dizziness	3	(2.5)
	Influenza	3	(2.5)
	Injection site pain	3	(2.5)
	Total ADRs	***118***	

Abbreviations: *ADR* Adverse drug reaction, *MedDRA* Medical Dictionary for Regulatory Activities, *PT* preferred term
