# Supplementary material for: Implementation and Results of Active Vaccine Safety Monitoring During the COVID-19 Pandemic in the UK: A Regulatory Perspective
Source: Drug Saf. 2025 Sep 3;48(12):1365–85. doi: 10.1007/s40264-025-01579-w (PMC12605443; doi:10.1007/s40264-025-01579-w)
Supplement: Supplementary file 12 — Supplementary file12 (PDF 726 KB) [file 40264_2025_1579_MOESM12_ESM.pdf]

Online Resource 12

Electronic Supplementary material

Article Title: Implementation and results of active vaccine safety monitoring during the COVID-19 pandemic in the UK: a regulatory perspective

Journal for Submission: Drug Safety (Springer Nature)

Authors: Jenny Wong, Katherine Donegan, Kendal Harrison, Tahira Jan, Alison Cave, and Phil Tregunno

Author Affiliation: Medicines and Healthcare products Regulatory Agency, London, UK

Corresponding Author: Phil Tregunno, phil.tregunno@mhra.gov.uk

Adverse Reactions (ADRs) Reported in association with a COVID-19 Vaccination by Pregnant or Breastfeeding Females

Total ADRs reported

A description of the tables is presented below. Each of these tables capture the full listing of ADRs reported by MedDRA SOC, HLGT, HLT and PT level terms for each brand and dose within the cohorts of individuals indicating they were (i) pregnant or (ii) breastfeeding at the time of a vaccination.

PREGNANT FEMALES

**Supplementary Table 24:**	ADR listing for reactions reported by pregnant females, in those reporting any vaccination dose.
**Supplementary Table 25:**	Pfizer BioNTech COVID-19 vaccine: ADR listing for reactions reported by pregnant females, in those reporting any vaccination dose.
**Supplementary Table 26:**	AstraZeneca COVID-19 vaccine: ADR listing for reactions reported by pregnant females, in those reporting any vaccination dose.
**Supplementary Table 27:**	Moderna COVID-19 vaccine: ADR listing for reactions reported by pregnant females, in those reporting any vaccination dose.

**Supplementary Table 28:**	Others/Unknown COVID-19 vaccine: ADR listings for reactions reported by pregnant females, in reporting any vaccination dose.
**Supplementary Table 29:**	COVID-19 vaccine ADR listings for reactions reported by pregnant females with no dose identification information in those reporting any vaccination dose.

BREASTFEEDING FEMALES

**Supplementary Table 30:**	ADR listing for reactions reported by breastfeeding females, in those reporting any vaccination dose.
**Supplementary Table 31:**	Pfizer BioNTech COVID-19 vaccine: ADR listing for reactions reported by breastfeeding females, in those reporting any vaccination dose.
**Supplementary Table 32:**	AstraZeneca COVID-19 vaccine: ADR listing for reactions reported by breastfeeding females, in those reporting any vaccination dose.
**Supplementary Table 33:**	Moderna COVID-19 vaccine: ADR listing for reactions reported by breastfeeding females, in those reporting any vaccination dose.
**Supplementary Table 34:**	Others/Unknown COVID-19 vaccine: ADR listings for reactions reported by breastfeeding females, in reporting any vaccination dose.
**Supplementary Table 35:**	COVID-19 vaccine ADR listings for reactions reported by breastfeeding females with no dose identification information in those reporting any vaccination dose.

Abbreviations

ADR	Adverse drug reaction
MedDRA	Medical Dictionary for Regulatory Activities
SOC	System Organ Class
HLGT	High-Level Group Term
HLT	High-Level Term
PT	Preferred Term

SUPPLEMENTARY TABLE 24. ADR listing for events reported by pregnant females in those reporting any vaccination dose.

	Individuals with any vaccination dose: ADR Counts					
**MEDDRA REACTION TERM (SOC, *HLGT*, *HLT*, PT)**	All doses	1st dose	2nd dose	3rd dose	Other doses	Unknown
(freetext)	0	0	0	0	0	0
**BLOOD AND LYMPHATIC SYSTEM DISORDERS**						
SPLEEN, LYMPHATIC AND RETICULOENDOTHELIAL SYSTEM DISORDERS						
LYMPHATIC SYSTEM DISORDERS NEC						
LYMPH NODE PAIN	4	0	2	1	0	1
LYMPHADENITIS	2	1	1	0	0	0
LYMPHADENOPATHY	11	7	4	0	0	0
**CARDIAC DISORDERS**						
CARDIAC ARRHYTHMIAS						
RATE AND RHYTHM DISORDERS NEC						
TACHYCARDIA	1	0	1	0	0	0
CARDIAC DISORDERS, SIGNS AND SYMPTOMS NEC						
CARDIAC SIGNS AND SYMPTOMS NEC						
PALPITATIONS	5	2	3	0	0	0
**EAR AND LABYRINTH DISORDERS**						
AURAL DISORDERS NEC						
EAR DISORDERS NEC						
EAR PAIN	1	1	0	0	0	0
INNER EAR AND VIIITH CRANIAL NERVE DISORDERS						
INNER EAR SIGNS AND SYMPTOMS						
TINNITUS	2	0	1	1	0	0
VERTIGO	1	1	0	0	0	0
**EYE DISORDERS**						
EYE DISORDERS NEC						
OCULAR DISORDERS NEC						
EYE PAIN	3	2	1	0	0	0
OCULAR SENSORY SYMPTOMS NEC						
OCULAR SENSATION DISORDERS						
ASTHENOPIA	2	1	1	0	0	0
PHOTOPHOBIA	2	2	0	0	0	0
VISION DISORDERS						
VISUAL DISORDERS NEC						
VISION BLURRED	4	2	2	0	0	0
**GASTROINTESTINAL DISORDERS**						
DENTAL AND GINGIVAL CONDITIONS						
DENTAL DISORDERS NEC						
TEETHING	1	1	0	0	0	0
GASTROINTESTINAL INFLAMMATORY CONDITIONS						
GASTROINTESTINAL INFLAMMATORY DISORDERS NEC						
GASTROINTESTINAL TRACT IRRITATION	1	0	0	1	0	0
GASTROINTESTINAL MOTILITY AND DEFAECATION CONDITIONS						
DIARRHOEA (EXCL INFECTIVE)						
DIARRHOEA	15	9	3	3	0	0
GASTROINTESTINAL ATONIC AND HYPOMOTILITY DISORDERS NEC						

CONSTIPATION	1	0	0	1	0	0
GASTROINTESTINAL SIGNS AND SYMPTOMS						
GASTROINTESTINAL AND ABDOMINAL PAINS (EXCL ORAL AND THROAT)						
ABDOMINAL PAIN	1	1	0	0	0	0
ABDOMINAL PAIN UPPER	4	3	1	0	0	0
GASTROINTESTINAL SIGNS AND SYMPTOMS NEC						
ABDOMINAL DISCOMFORT	2	2	0	0	0	0
NAUSEA AND VOMITING SYMPTOMS						
NAUSEA	73	43	23	4	0	3
VOMITING	22	14	7	0	0	1
ORAL SOFT TISSUE CONDITIONS						
ORAL SOFT TISSUE SIGNS AND SYMPTOMS						
HYPOAESTHESIA ORAL	1	1	0	0	0	0
PARAESTHESIA ORAL	1	1	0	0	0	0
TONGUE CONDITIONS						
TONGUE SIGNS AND SYMPTOMS						
GLOSSODYNIA	1	1	0	0	0	0
**GENERAL DISORDERS AND ADMINISTRATION SITE CONDITIONS**						
ADMINISTRATION SITE REACTIONS						
ADMINISTRATION SITE REACTIONS NEC						
PUNCTURE SITE BRUISE	2	0	0	1	0	1
PUNCTURE SITE PAIN	1	1	0	0	0	0
INJECTION SITE REACTIONS						
INJECTION SITE BRUISING	1	0	1	0	0	0
INJECTION SITE ERYTHEMA	1	1	0	0	0	0
INJECTION SITE MASS	3	1	1	0	0	1
INJECTION SITE PAIN	49	36	9	3	0	1
INJECTION SITE REACTION	1	0	1	0	0	0
INJECTION SITE URTICARIA	1	0	1	0	0	0
INJECTION SITE WARMTH	1	1	0	0	0	0
VACCINATION SITE REACTIONS						
VACCINATION SITE PAIN	4	2	1	0	0	1
BODY TEMPERATURE CONDITIONS						
FEBRILE DISORDERS						
PYREXIA	121	74	33	8	0	6
GENERAL SYSTEM DISORDERS NEC						
ASTHENIC CONDITIONS						
ASTHENIA	6	4	0	1	0	1
FATIGUE	276	167	74	17	1	17
MALAISE	16	8	6	1	1	0
FEELINGS AND SENSATIONS NEC						
CHILLS	59	35	16	7	0	1
FEELING ABNORMAL	4	2	1	1	0	0
FEELING COLD	3	2	1	0	0	0
FEELING HOT	1	1	0	0	0	0
FEELING OF BODY TEMPERATURE CHANGE	1	1	0	0	0	0
HANGOVER	1	0	0	1	0	0
HUNGER	1	1	0	0	0	0
THIRST	2	2	0	0	0	0
GENERAL SIGNS AND SYMPTOMS NEC						
CRYING	1	1	0	0	0	0
ILLNESS	12	8	3	1	0	0

INFLUENZA LIKE ILLNESS	16	9	5	1	0	1
LOCAL REACTION	1	0	1	0	0	0
PERIPHERAL SWELLING	4	3	0	1	0	0
SWELLING	4	2	1	1	0	0
PAIN AND DISCOMFORT NEC						
AXILLARY PAIN	10	3	5	2	0	0
CHEST PAIN	1	1	0	0	0	0
PAIN	67	35	22	5	0	5
TENDERNESS	10	8	2	0	0	0
**INFECTIONS AND INFESTATIONS**						
BACTERIAL INFECTIOUS DISORDERS						
BACTERIAL INFECTIONS NEC						
PERIORBITAL CELLULITIS	1	0	1	0	0	0
INFECTIONS - PATHOGEN UNSPECIFIED						
BREAST INFECTIONS						
MASTITIS	1	0	0	1	0	0
UPPER RESPIRATORY TRACT INFECTIONS						
LARYNGITIS	2	1	1	0	0	0
NASOPHARYNGITIS	12	7	4	1	0	0
VIRAL INFECTIOUS DISORDERS						
HERPES VIRAL INFECTIONS						
GENITAL HERPES	1	1	0	0	0	0
HERPES ZOSTER	1	1	0	0	0	0
ORAL HERPES	1	0	1	0	0	0
INFLUENZA VIRAL INFECTIONS						
INFLUENZA	24	12	9	3	0	0
VIRAL INFECTIONS NEC						
VIRAL DIARRHOEA	1	1	0	0	0	0
**INJURY, POISONING AND PROCEDURAL COMPLICATIONS**						
INJURIES NEC						
SKIN INJURIES NEC						
CONTUSION	7	7	0	0	0	0
PROCEDURAL RELATED INJURIES AND COMPLICATIONS NEC						
NON-SITE SPECIFIC PROCEDURAL COMPLICATIONS						
INJECTION RELATED REACTION	1	0	0	1	0	0
**INVESTIGATIONS**						
CARDIAC AND VASCULAR INVESTIGATIONS (EXCL ENZYME TESTS)						
HEART RATE AND PULSE INVESTIGATIONS						
HEART RATE	2	2	0	0	0	0
HEART RATE INCREASED	1	1	0	0	0	0
PHYSICAL EXAMINATION AND ORGAN SYSTEM STATUS TOPICS						
PHYSICAL EXAMINATION PROCEDURES AND ORGAN SYSTEM STATUS						
BODY TEMPERATURE INCREASED	2	1	0	1	0	0
WATER, ELECTROLYTE AND MINERAL INVESTIGATIONS						
WATER AND ELECTROLYTE ANALYSES NEC						
VOLUME BLOOD	1	0	1	0	0	0
**METABOLISM AND NUTRITION DISORDERS**						
APPETITE AND GENERAL NUTRITIONAL DISORDERS						

APPETITE DISORDERS						
DECREASED APPETITE	3	2	1	0	0	0
ELECTROLYTE AND FLUID BALANCE CONDITIONS						
TOTAL FLUID VOLUME DECREASED						
DEHYDRATION	1	1	0	0	0	0
**MUSCULOSKELETAL AND CONNECTIVE TISSUE DISORDERS**						
BONE DISORDERS (EXCL CONGENITAL AND FRACTURES)						
BONE RELATED SIGNS AND SYMPTOMS						
PAIN IN JAW	3	1	2	0	0	0
JOINT DISORDERS						
JOINT RELATED SIGNS AND SYMPTOMS						
ARTHRALGIA	37	24	12	0	0	1
MUSCLE DISORDERS						
MUSCLE PAINS						
MYALGIA	78	50	21	5	0	2
MUSCLE RELATED SIGNS AND SYMPTOMS NEC						
MUSCLE FATIGUE	2	1	0	1	0	0
MUSCLE SPASMS	1	1	0	0	0	0
MUSCLE TONE ABNORMALITIES						
TRISMUS	1	0	1	0	0	0
MUSCULOSKELETAL AND CONNECTIVE TISSUE DISORDERS NEC						
MUSCULOSKELETAL AND CONNECTIVE TISSUE CONDITIONS NEC						
MUSCULOSKELETAL STIFFNESS	7	4	3	0	0	0
BACK PAIN	7	4	2	1	0	0
LIMB DISCOMFORT	44	34	9	1	0	0
NECK PAIN	3	2	1	0	0	0
PAIN IN EXTREMITY	383	302	54	16	1	10
**NERVOUS SYSTEM DISORDERS**						
CRANIAL NERVE DISORDERS (EXCL NEOPLASMS)						
FACIAL CRANIAL NERVE DISORDERS						
BELL'S PALSY	1	0	1	0	0	0
OLFACTORY NERVE DISORDERS						
ANOSMIA	1	1	0	0	0	0
PAROSMIA	2	1	1	0	0	0
HEADACHES						
HEADACHES NEC						
CLUSTER HEADACHE	1	0	1	0	0	0
HEADACHE	220	134	58	14	0	14
SINUS HEADACHE	8	5	3	0	0	0
TENSION HEADACHE	11	8	3	0	0	0
MIGRAINE HEADACHES						
MIGRAINE WITH AURA	1	0	1	0	0	0
TYPICAL AURA WITHOUT HEADACHE	1	0	1	0	0	0
MENTAL IMPAIRMENT DISORDERS						
MENTAL IMPAIRMENT (EXCL DEMENTIA AND MEMORY LOSS)						
DISTURBANCE IN ATTENTION	1	0	1	0	0	0
MOVEMENT DISORDERS (INCL PARKINSONISM)						
DYSKINESIAS AND MOVEMENT DISORDERS NEC						
CLUMSINESS	1	1	0	0	0	0
TREMOR (EXCL CONGENITAL)						
TREMOR	4	3	1	0	0	0
NEUROLOGICAL DISORDERS NEC						
DISTURBANCES IN CONSCIOUSNESS NEC						
LETHARGY	12	8	3	1	0	0
SOMNOLENCE	8	5	1	0	0	2
SYNCOPE	2	2	0	0	0	0

NEUROLOGICAL SIGNS AND SYMPTOMS NEC						
AGITATION NEONATAL	1	0	0	0	0	1
BRAIN FOG	1	1	0	0	0	0
DIZZINESS	37	22	11	3	0	1
DIZZINESS EXERTIONAL	1	1	0	0	0	0
PRESYNCOPE	1	1	0	0	0	0
PARAESTHESIAS AND DYSAESTHESIAS						
PARAESTHESIA	1	1	0	0	0	0
SENSORY ABNORMALITIES NEC						
DYSGEUSIA	1	1	0	0	0	0
NEURALGIA	1	1	0	0	0	0
TASTE DISORDER	1	1	0	0	0	0
SEIZURES (INCL SUBTYPES)						
SEIZURES AND SEIZURE DISORDERS NEC						
SEIZURE	1	1	0	0	0	0
**PREGNANCY, PUERPERIUM AND PERINATAL CONDITIONS**						
ABORTIONS AND STILLBIRTH						
ABORTIONS SPONTANEOUS						
ABORTION SPONTANEOUS	19	11	8	0	0	0
STILLBIRTH AND FOETAL DEATH						
FOETAL DEATH	1	1	0	0	0	0
FOETAL COMPLICATIONS						
FOETAL COMPLICATIONS NEC						
FOETAL DISORDER	1	1	0	0	0	0
FOETAL HYPOKINESIA	2	1	0	1	0	0
FOETAL GROWTH COMPLICATIONS						
FOETAL MACROSOMIA	1	1	0	0	0	0
MATERNAL COMPLICATIONS OF PREGNANCY						
MATERNAL COMPLICATIONS OF PREGNANCY NEC						
MORNING SICKNESS	1	0	0	0	0	1
PLACENTAL, AMNIOTIC AND CAVITY DISORDERS (EXCL HAEMORRHAGES)						
PLACENTAL ABNORMALITIES (EXCL NEOPLASMS)						
PLACENTAL INFARCTION	1	1	0	0	0	0
PREGNANCY, LABOUR, DELIVERY AND POSTPARTUM CONDITIONS						
NORMAL PREGNANCY, LABOUR AND DELIVERY						
PREGNANCY	1	0	0	0	0	1
UTERINE CONTRACTIONS DURING PREGNANCY	1	1	0	0	0	0
**PSYCHIATRIC DISORDERS**						
ANXIETY DISORDERS AND SYMPTOMS						
ANXIETY SYMPTOMS						
AGITATION	1	0	1	0	0	0
ANXIETY	3	1	0	0	0	2
COGNITIVE AND ATTENTION DISORDERS AND DISTURBANCES						
COGNITIVE AND ATTENTION DISORDERS AND DISTURBANCES NEC						
MENTAL FATIGUE	1	1	0	0	0	0
DELIRIA (INCL CONFUSION)						
CONFUSION AND DISORIENTATION						
CONFUSIONAL STATE	1	1	0	0	0	0
DEPRESSED MOOD DISORDERS AND DISTURBANCES						
MOOD ALTERATIONS WITH DEPRESSIVE SYMPTOMS						
DEPRESSED MOOD	1	1	0	0	0	0
DISTURBANCES IN THINKING AND PERCEPTION						
HALLUCINATIONS (EXCL SLEEP-RELATED)						
HALLUCINATION	1	1	0	0	0	0
MOOD DISORDERS AND DISTURBANCES NEC						
EMOTIONAL AND MOOD DISTURBANCES NEC						

EMOTIONAL DISORDER	1	0	1	0	0	0
SLEEP DISORDERS AND DISTURBANCES						
DISTURBANCES IN INITIATING AND MAINTAINING SLEEP						
INITIAL INSOMNIA	1	1	0	0	0	0
INSOMNIA	7	5	2	0	0	0
**REPRODUCTIVE SYSTEM AND BREAST DISORDERS**						
MENSTRUAL CYCLE AND UTERINE BLEEDING DISORDERS						
MENSTRUATION AND UTERINE BLEEDING NEC						
MENSTRUAL DISORDER	1	0	0	1	0	0
MENSTRUATION WITH DECREASED BLEEDING						
MENSTRUATION DELAYED	2	0	1	0	0	1
OLIGOMENORRHOEA	1	0	0	0	0	1
MENSTRUATION WITH INCREASED BLEEDING						
HEAVY MENSTRUAL BLEEDING	2	0	0	1	0	1
VULVOVAGINAL DISORDERS (EXCL INFECTIONS AND INFLAMMATIONS)						
VULVOVAGINAL DISORDERS NEC						
VAGINAL HAEMORRHAGE	6	4	2	0	0	0
**RESPIRATORY, THORACIC AND MEDIASTINAL DISORDERS**						
RESPIRATORY DISORDERS NEC						
BREATHING ABNORMALITIES						
DYSPNOEA	10	5	4	0	0	1
COUGHING AND ASSOCIATED SYMPTOMS						
COUGH	5	4	1	0	0	0
RESPIRATORY TRACT SIGNS AND SYMPTOMS						
UPPER RESPIRATORY TRACT SIGNS AND SYMPTOMS						
DRY THROAT	1	0	1	0	0	0
OROPHARYNGEAL PAIN	18	14	3	0	0	1
PARANASAL SINUS DISCOMFORT	1	1	0	0	0	0
RHINORRHOEA	7	4	2	1	0	0
SINUS PAIN	2	2	0	0	0	0
SNEEZING	1	1	0	0	0	0
UPPER RESPIRATORY TRACT DISORDERS (EXCL INFECTIONS)						
NASAL CONGESTION AND INFLAMMATIONS						
NASAL CONGESTION	2	0	2	0	0	0
**SKIN AND SUBCUTANEOUS TISSUE DISORDERS**						
ANGIOEDEMA AND URTICARIA						
URTICARIAS						
URTICARIA	1	1	0	0	0	0
CORNIFICATION AND DYSTROPHIC SKIN DISORDERS						
SKIN DYSTROPHIES						
HYPERTROPHIC SCAR	1	1	0	0	0	0
EPIDERMAL AND DERMAL CONDITIONS						
DERMAL AND EPIDERMAL CONDITIONS NEC						
SKIN WARM	1	1	0	0	0	0
DERMATITIS AND ECZEMA						
DERMATITIS ALLERGIC	1	1	0	0	0	0
ERYTHEMAS						
ERYTHEMA	3	1	1	0	0	1
PRURITUS NEC						
PRURITUS	5	3	0	1	0	1
RASHES, ERUPTIONS AND EXANTHEMS NEC						
RASH	9	6	1	2	0	0
RASH PRURITIC	1	1	0	0	0	0
SKIN APPENDAGE CONDITIONS						
APOCRINE AND ECCRINE GLAND DISORDERS						
COLD SWEAT	2	2	0	0	0	0

HYPERHIDROSIS	6	6	0	0	0	0
NIGHT SWEATS	2	0	2	0	0	0
**VASCULAR DISORDERS**						
DECREASED AND NONSPECIFIC BLOOD PRESSURE DISORDERS AND SHOCK						
BLOOD PRESSURE DISORDERS NEC						
BLOOD PRESSURE FLUCTUATION	1	1	0	0	0	0
VASCULAR HYPOTENSIVE DISORDERS						
HYPOTENSION	2	2	0	0	0	0
EMBOLISM AND THROMBOSIS						
NON-SITE SPECIFIC EMBOLISM AND THROMBOSIS						
EMBOLISM	1	1	0	0	0	0
VASCULAR DISORDERS NEC						
NON-SITE SPECIFIC VASCULAR DISORDERS NEC						
VASCULAR PAIN	1	0	1	0	0	0
PERIPHERAL VASCULAR DISORDERS NEC						
HOT FLUSH	2	2	0	0	0	0
VASCULAR HAEMORRHAGIC DISORDERS						
HAEMORRHAGES NEC						
HAEMORRHAGE	1	1	0	0	0	0
**TOTAL ADR EVENTS**	***1934***	***1255***	***476***	***118***	***3***	***82***

SUPPLEMENTARY TABLE 25. Pfizer BioNTech COVID-19 vaccine: ADR listing for events reported by pregnant females, in those reporting any vaccination dose

	Individuals with any vaccination dose: ADR Counts				
**MEDDRA REACTION TERM (SOC, *HLGT*, *HLT*, PT)**	All doses	1st dose	2nd dose	3rd dose	Other doses
(freetext)					
**BLOOD AND LYMPHATIC SYSTEM DISORDERS**					
SPLEEN, LYMPHATIC AND RETICULOENDOTHELIAL SYSTEM DISORDERS					
LYMPHATIC SYSTEM DISORDERS NEC					
LYMPH NODE PAIN	3	0	2	1	0
LYMPHADENITIS	1	0	1	0	0
LYMPHADENOPATHY	9	6	3	0	0
**CARDIAC DISORDERS**					
CARDIAC ARRHYTHMIAS					
RATE AND RHYTHM DISORDERS NEC					
TACHYCARDIA	0	0	0	0	0
CARDIAC DISORDERS, SIGNS AND SYMPTOMS NEC					
CARDIAC SIGNS AND SYMPTOMS NEC					
PALPITATIONS	3	1	2	0	0
**EAR AND LABYRINTH DISORDERS**					
AURAL DISORDERS NEC					
EAR DISORDERS NEC					
EAR PAIN	1	1	0	0	0
INNER EAR AND VIIITH CRANIAL NERVE DISORDERS					
INNER EAR SIGNS AND SYMPTOMS					
TINNITUS	1	0	1	0	0
VERTIGO	0	0	0	0	0
**EYE DISORDERS**					
EYE DISORDERS NEC					
OCULAR DISORDERS NEC					
EYE PAIN	1	1	0	0	0
OCULAR SENSORY SYMPTOMS NEC					
OCULAR SENSATION DISORDERS					
ASTHENOPIA	2	1	1	0	0
PHOTOPHOBIA	1	1	0	0	0
VISION DISORDERS					
VISUAL DISORDERS NEC					
VISION BLURRED	2	1	1	0	0
**GASTROINTESTINAL DISORDERS**					
DENTAL AND GINGIVAL CONDITIONS					
DENTAL DISORDERS NEC					
TEETHING	1	1	0	0	0
GASTROINTESTINAL INFLAMMATORY CONDITIONS					
GASTROINTESTINAL INFLAMMATORY DISORDERS NEC					
GASTROINTESTINAL TRACT IRRITATION	1	0	0	1	0
GASTROINTESTINAL MOTILITY AND DEFAECATION CONDITIONS					
DIARRHOEA (EXCL INFECTIVE)					
DIARRHOEA	13	7	3	3	0
GASTROINTESTINAL ATONIC AND HYPOMOTILITY DISORDERS NEC					
CONSTIPATION	1	0	0	1	0

GASTROINTESTINAL SIGNS AND SYMPTOMS					
GASTROINTESTINAL AND ABDOMINAL PAINS (EXCL ORAL AND THROAT)					
ABDOMINAL PAIN	0	0	0	0	0
ABDOMINAL PAIN UPPER	4	3	1	0	0
GASTROINTESTINAL SIGNS AND SYMPTOMS NEC					
ABDOMINAL DISCOMFORT	0	0	0	0	0
NAUSEA AND VOMITING SYMPTOMS					
NAUSEA	45	26	18	1	0
VOMITING	12	9	3	0	0
ORAL SOFT TISSUE CONDITIONS					
ORAL SOFT TISSUE SIGNS AND SYMPTOMS					
HYPOAESTHESIA ORAL	1	1	0	0	0
PARAESTHESIA ORAL	1	1	0	0	0
TONGUE CONDITIONS					
TONGUE SIGNS AND SYMPTOMS					
GLOSSODYNIA	1	1	0	0	0
**GENERAL DISORDERS AND ADMINISTRATION SITE CONDITIONS**					
ADMINISTRATION SITE REACTIONS					
ADMINISTRATION SITE REACTIONS NEC					
PUNCTURE SITE BRUISE	0	0	0	0	0
PUNCTURE SITE PAIN	1	1	0	0	0
INJECTION SITE REACTIONS					
INJECTION SITE BRUISING	1	0	1	0	0
INJECTION SITE ERYTHEMA	1	1	0	0	0
INJECTION SITE MASS	1	0	1	0	0
INJECTION SITE PAIN	39	31	7	1	0
INJECTION SITE REACTION	1	0	1	0	0
INJECTION SITE URTICARIA	1	0	1	0	0
INJECTION SITE WARMTH	1	1	0	0	0
VACCINATION SITE REACTIONS					
VACCINATION SITE PAIN	1	1	0	0	0
BODY TEMPERATURE CONDITIONS					
FEBRILE DISORDERS					
PYREXIA	40	22	17	1	0
GENERAL SYSTEM DISORDERS NEC					
ASTHENIC CONDITIONS					
ASTHENIA	2	2	0	0	0
FATIGUE	166	112	48	6	0
MALAISE	10	4	6	0	0
FEELINGS AND SENSATIONS NEC					
CHILLS	12	4	7	1	0
FEELING ABNORMAL	4	2	1	1	0
FEELING COLD	1	0	1	0	0
FEELING HOT	0	0	0	0	0
FEELING OF BODY TEMPERATURE CHANGE	0	0	0	0	0
HANGOVER	1	0	0	1	0
HUNGER	0	0	0	0	0
THIRST	1	1	0	0	0
GENERAL SIGNS AND SYMPTOMS NEC					
CRYING	0	0	0	0	0
ILLNESS	7	4	3	0	0
INFLUENZA LIKE ILLNESS	7	3	4	0	0

LOCAL REACTION	1	0	1	0	0
PERIPHERAL SWELLING	3	2	0	1	0
SWELLING	2	1	1	0	0
PAIN AND DISCOMFORT NEC					
AXILLARY PAIN	9	2	5	2	0
CHEST PAIN	1	1	0	0	0
PAIN	39	24	12	3	0
TENDERNESS	8	6	2	0	0
**INFECTIONS AND INFESTATIONS**					
BACTERIAL INFECTIOUS DISORDERS					
BACTERIAL INFECTIONS NEC					
PERIORBITAL CELLULITIS	1	0	1	0	0
INFECTIONS - PATHOGEN UNSPECIFIED					
BREAST INFECTIONS					
MASTITIS	1	0	0	1	0
UPPER RESPIRATORY TRACT INFECTIONS					
LARYNGITIS	1	0	1	0	0
NASOPHARYNGITIS	7	4	3	0	0
VIRAL INFECTIOUS DISORDERS					
HERPES VIRAL INFECTIONS					
GENITAL HERPES	1	1	0	0	0
HERPES ZOSTER	1	1	0	0	0
ORAL HERPES	1	0	1	0	0
INFLUENZA VIRAL INFECTIONS					
INFLUENZA	9	4	4	1	0
VIRAL INFECTIONS NEC					
VIRAL DIARRHOEA	1	1	0	0	0
**INJURY, POISONING AND PROCEDURAL COMPLICATIONS**					
INJURIES NEC					
SKIN INJURIES NEC					
CONTUSION	6	6	0	0	0
PROCEDURAL RELATED INJURIES AND COMPLICATIONS NEC					
NON-SITE SPECIFIC PROCEDURAL COMPLICATIONS					
INJECTION RELATED REACTION	0	0	0	0	0
**INVESTIGATIONS**					
CARDIAC AND VASCULAR INVESTIGATIONS (EXCL ENZYME TESTS)					
HEART RATE AND PULSE INVESTIGATIONS					
HEART RATE	1	1	0	0	0
HEART RATE INCREASED	0	0	0	0	0
PHYSICAL EXAMINATION AND ORGAN SYSTEM STATUS TOPICS					
PHYSICAL EXAMINATION PROCEDURES AND ORGAN SYSTEM STATUS					
BODY TEMPERATURE INCREASED	0	0	0	0	0
WATER, ELECTROLYTE AND MINERAL INVESTIGATIONS					
WATER AND ELECTROLYTE ANALYSES NEC					
VOLUME BLOOD	1	0	1	0	0
**METABOLISM AND NUTRITION DISORDERS**					
APPETITE AND GENERAL NUTRITIONAL DISORDERS					
APPETITE DISORDERS					
DECREASED APPETITE	0	0	0	0	0
ELECTROLYTE AND FLUID BALANCE CONDITIONS					
TOTAL FLUID VOLUME DECREASED					
DEHYDRATION	0	0	0	0	0
**MUSCULOSKELETAL AND CONNECTIVE TISSUE DISORDERS**					

BONE DISORDERS (EXCL CONGENITAL AND FRACTURES)					
BONE RELATED SIGNS AND SYMPTOMS					
PAIN IN JAW	1	1	0	0	0
JOINT DISORDERS					
JOINT RELATED SIGNS AND SYMPTOMS					
ARTHRALGIA	26	16	10	0	0
MUSCLE DISORDERS					
MUSCLE PAINS					
MYALGIA	37	25	11	1	0
MUSCLE RELATED SIGNS AND SYMPTOMS NEC					
MUSCLE FATIGUE	0	0	0	0	0
MUSCLE SPASMS	1	1	0	0	0
MUSCLE TONE ABNORMALITIES					
TRISMUS	0	0	0	0	0
MUSCULOSKELETAL AND CONNECTIVE TISSUE DISORDERS NEC					
MUSCULOSKELETAL AND CONNECTIVE TISSUE CONDITIONS NEC					
MUSCULOSKELETAL STIFFNESS	4	3	1	0	0
BACK PAIN	2	1	0	1	0
LIMB DISCOMFORT	37	28	8	1	0
NECK PAIN	2	2	0	0	0
PAIN IN EXTREMITY	293	235	45	12	1
**NERVOUS SYSTEM DISORDERS**					
CRANIAL NERVE DISORDERS (EXCL NEOPLASMS)					
FACIAL CRANIAL NERVE DISORDERS					
BELL'S PALSY	1	0	1	0	0
OLFACTORY NERVE DISORDERS					
ANOSMIA	1	1	0	0	0
PAROSMIA	1	1	0	0	0
HEADACHES					
HEADACHES NEC					
CLUSTER HEADACHE	1	0	1	0	0
HEADACHE	120	79	33	8	0
SINUS HEADACHE	5	3	2	0	0
TENSION HEADACHE	6	4	2	0	0
MIGRAINE HEADACHES					
MIGRAINE WITH AURA	0	0	0	0	0
TYPICAL AURA WITHOUT HEADACHE	1	0	1	0	0
MENTAL IMPAIRMENT DISORDERS					
MENTAL IMPAIRMENT (EXCL DEMENTIA AND MEMORY LOSS)					
DISTURBANCE IN ATTENTION	1	0	1	0	0
MOVEMENT DISORDERS (INCL PARKINSONISM)					
DYSKINESIAS AND MOVEMENT DISORDERS NEC					
CLUMSINESS	0	0	0	0	0
TREMOR (EXCL CONGENITAL)					
TREMOR	0	0	0	0	0
NEUROLOGICAL DISORDERS NEC					
DISTURBANCES IN CONSCIOUSNESS NEC					
LETHARGY	10	6	3	1	0
SOMNOLENCE	4	4	0	0	0
SYNCOPE	2	2	0	0	0
NEUROLOGICAL SIGNS AND SYMPTOMS NEC					
AGITATION NEONATAL	0	0	0	0	0
BRAIN FOG	0	0	0	0	0

DIZZINESS	19	10	8	1	0
DIZZINESS EXERTIONAL	0	0	0	0	0
PRESYNCOPE	0	0	0	0	0
PARAESTHESIAS AND DYSAESTHESIAS					
PARAESTHESIA	0	0	0	0	0
SENSORY ABNORMALITIES NEC					
DYSGEUSIA	0	0	0	0	0
NEURALGIA	1	1	0	0	0
TASTE DISORDER	1	1	0	0	0
SEIZURES (INCL SUBTYPES)					
SEIZURES AND SEIZURE DISORDERS NEC					
SEIZURE	1	1	0	0	0
**PREGNANCY, PUERPERIUM AND PERINATAL CONDITIONS**					
ABORTIONS AND STILLBIRTH					
ABORTIONS SPONTANEOUS					
ABORTION SPONTANEOUS	13	7	6	0	0
STILLBIRTH AND FOETAL DEATH					
FOETAL DEATH	0	0	0	0	0
FOETAL COMPLICATIONS					
FOETAL COMPLICATIONS NEC					
FOETAL DISORDER	0	0	0	0	0
FOETAL HYPOKINESIA	2	1	0	1	0
FOETAL GROWTH COMPLICATIONS					
FOETAL MACROSOMIA	1	1	0	0	0
MATERNAL COMPLICATIONS OF PREGNANCY					
MATERNAL COMPLICATIONS OF PREGNANCY NEC					
MORNING SICKNESS	0	0	0	0	0
PLACENTAL, AMNIOTIC AND CAVITY DISORDERS (EXCL HAEMORRHAGES)					
PLACENTAL ABNORMALITIES (EXCL NEOPLASMS)					
PLACENTAL INFARCTION	0	0	0	0	0
PREGNANCY, LABOUR, DELIVERY AND POSTPARTUM CONDITIONS					
NORMAL PREGNANCY, LABOUR AND DELIVERY					
PREGNANCY	0	0	0	0	0
UTERINE CONTRACTIONS DURING PREGNANCY	1	1	0	0	0
**PSYCHIATRIC DISORDERS**					
ANXIETY DISORDERS AND SYMPTOMS					
ANXIETY SYMPTOMS					
AGITATION	1	0	1	0	0
ANXIETY	1	1	0	0	0
COGNITIVE AND ATTENTION DISORDERS AND DISTURBANCES					
COGNITIVE AND ATTENTION DISORDERS AND DISTURBANCES NEC					
MENTAL FATIGUE	1	1	0	0	0
DELIRIA (INCL CONFUSION)					
CONFUSION AND DISORIENTATION					
CONFUSIONAL STATE	1	1	0	0	0
DEPRESSED MOOD DISORDERS AND DISTURBANCES					
MOOD ALTERATIONS WITH DEPRESSIVE SYMPTOMS					
DEPRESSED MOOD	1	1	0	0	0
DISTURBANCES IN THINKING AND PERCEPTION					
HALLUCINATIONS (EXCL SLEEP-RELATED)					
HALLUCINATION	1	1	0	0	0
MOOD DISORDERS AND DISTURBANCES NEC					
EMOTIONAL AND MOOD DISTURBANCES NEC					

EMOTIONAL DISORDER	1	0	1	0	0
SLEEP DISORDERS AND DISTURBANCES					
DISTURBANCES IN INITIATING AND MAINTAINING SLEEP					
INITIAL INSOMNIA	1	1	0	0	0
INSOMNIA	3	2	1	0	0
**REPRODUCTIVE SYSTEM AND BREAST DISORDERS**					
MENSTRUAL CYCLE AND UTERINE BLEEDING DISORDERS					
MENSTRUATION AND UTERINE BLEEDING NEC					
MENSTRUAL DISORDER	1	0	0	1	0
MENSTRUATION WITH DECREASED BLEEDING					
MENSTRUATION DELAYED	0	0	0	0	0
OLIGOMENORRHOEA	0	0	0	0	0
MENSTRUATION WITH INCREASED BLEEDING					
HEAVY MENSTRUAL BLEEDING	1	0	0	1	0
VULVOVAGINAL DISORDERS (EXCL INFECTIONS AND INFLAMMATIONS)					
VULVOVAGINAL DISORDERS NEC					
VAGINAL HAEMORRHAGE	5	3	2	0	0
**RESPIRATORY, THORACIC AND MEDIASTINAL DISORDERS**					
RESPIRATORY DISORDERS NEC					
BREATHING ABNORMALITIES					
DYSPNOEA	5	4	1	0	0
COUGHING AND ASSOCIATED SYMPTOMS					
COUGH	4	3	1	0	0
RESPIRATORY TRACT SIGNS AND SYMPTOMS					
UPPER RESPIRATORY TRACT SIGNS AND SYMPTOMS					
DRY THROAT	1	0	1	0	0
OROPHARYNGEAL PAIN	12	9	3	0	0
PARANASAL SINUS DISCOMFORT	1	1	0	0	0
RHINORRHOEA	5	3	2	0	0
SINUS PAIN	1	1	0	0	0
SNEEZING	1	1	0	0	0
UPPER RESPIRATORY TRACT DISORDERS (EXCL INFECTIONS)					
NASAL CONGESTION AND INFLAMMATIONS					
NASAL CONGESTION	1	0	1	0	0
**SKIN AND SUBCUTANEOUS TISSUE DISORDERS**					
ANGIOEDEMA AND URTICARIA					
URTICARIAS					
URTICARIA	1	1	0	0	0
CORNIFICATION AND DYSTROPHIC SKIN DISORDERS					
SKIN DYSTROPHIES					
HYPERTROPHIC SCAR	1	1	0	0	0
EPIDERMAL AND DERMAL CONDITIONS					
DERMAL AND EPIDERMAL CONDITIONS NEC					
SKIN WARM	1	1	0	0	0
DERMATITIS AND ECZEMA					
DERMATITIS ALLERGIC	0	0	0	0	0
ERYTHEMAS					
ERYTHEMA	2	1	1	0	0
PRURITUS NEC					
PRURITUS	1	1	0	0	0
RASHES, ERUPTIONS AND EXANTHEMS NEC					
RASH	5	4	1	0	0
RASH PRURITIC	0	0	0	0	0

SKIN APPENDAGE CONDITIONS					
APOCRINE AND ECCRINE GLAND DISORDERS					
COLD SWEAT	0	0	0	0	0
HYPERHIDROSIS	3	3	0	0	0
NIGHT SWEATS	1	0	1	0	0
**VASCULAR DISORDERS**					
DECREASED AND NONSPECIFIC BLOOD PRESSURE DISORDERS AND SHOCK					
BLOOD PRESSURE DISORDERS NEC					
BLOOD PRESSURE FLUCTUATION	0	0	0	0	0
VASCULAR HYPOTENSIVE DISORDERS					
HYPOTENSION	2	2	0	0	0
EMBOLISM AND THROMBOSIS					
NON-SITE SPECIFIC EMBOLISM AND THROMBOSIS					
EMBOLISM	0	0	0	0	0
VASCULAR DISORDERS NEC					
NON-SITE SPECIFIC VASCULAR DISORDERS NEC					
VASCULAR PAIN	1	0	1	0	0
PERIPHERAL VASCULAR DISORDERS NEC					
HOT FLUSH	2	2	0	0	0
VASCULAR HAEMORRHAGIC DISORDERS					
HAEMORRHAGES NEC					
HAEMORRHAGE	1	1	0	0	0
**TOTAL ADR EVENTS**	***1161***	***789***	***317***	***54***	***1***

SUPPLEMENTARY TABLE 26. AstraZeneca COVID-19 vaccine: ADR listing for events reported by pregnant females, in those reporting any vaccination dose

**MEDDRA REACTION TERM (SOC, *HLGT*, *HLT*, PT)**	Individuals with any vaccination dose: ADR Counts				
	All doses	1st dose	2nd dose	3rd dose	Other doses
(freetext)	0	0	0	0	0
**BLOOD AND LYMPHATIC SYSTEM DISORDERS**					
SPLEEN, LYMPHATIC AND RETICULOENDOTHELIAL SYSTEM DISORDERS					
LYMPHATIC SYSTEM DISORDERS NEC					
LYMPH NODE PAIN	0	0	0	0	0
LYMPHADENITIS	0	0	0	0	0
LYMPHADENOPATHY	0	0	0	0	0
**CARDIAC DISORDERS**					
CARDIAC ARRHYTHMIAS					
RATE AND RHYTHM DISORDERS NEC					
TACHYCARDIA	1	0	1	0	0
CARDIAC DISORDERS, SIGNS AND SYMPTOMS NEC					
CARDIAC SIGNS AND SYMPTOMS NEC					
PALPITATIONS	0	0	0	0	0
**EAR AND LABYRINTH DISORDERS**					
AURAL DISORDERS NEC					
EAR DISORDERS NEC					
EAR PAIN	0	0	0	0	0
INNER EAR AND VIIITH CRANIAL NERVE DISORDERS					
INNER EAR SIGNS AND SYMPTOMS					
TINNITUS	0	0	0	0	0
VERTIGO	1	1	0	0	0
**EYE DISORDERS**					
EYE DISORDERS NEC					
OCULAR DISORDERS NEC					
EYE PAIN	1	0	1	0	0
OCULAR SENSORY SYMPTOMS NEC					
OCULAR SENSATION DISORDERS					
ASTHENOPIA	0	0	0	0	0
PHOTOPHOBIA	1	1	0	0	0
VISION DISORDERS					
VISUAL DISORDERS NEC					
VISION BLURRED	1	0	1	0	0
**GASTROINTESTINAL DISORDERS**					
DENTAL AND GINGIVAL CONDITIONS					
DENTAL DISORDERS NEC					
TEETHING	0	0	0	0	0
GASTROINTESTINAL INFLAMMATORY CONDITIONS					
GASTROINTESTINAL INFLAMMATORY DISORDERS NEC					
GASTROINTESTINAL TRACT IRRITATION	0	0	0	0	0
GASTROINTESTINAL MOTILITY AND DEFAECATION CONDITIONS					
DIARRHOEA (EXCL INFECTIVE)					
DIARRHOEA	1	1	0	0	0
GASTROINTESTINAL ATONIC AND HYPOMOTILITY DISORDERS NEC					
CONSTIPATION	0	0	0	0	0

GASTROINTESTINAL SIGNS AND SYMPTOMS					
GASTROINTESTINAL AND ABDOMINAL PAINS (EXCL ORAL AND THROAT)					
ABDOMINAL PAIN	1	1	0	0	0
ABDOMINAL PAIN UPPER	0	0	0	0	0
GASTROINTESTINAL SIGNS AND SYMPTOMS NEC					
ABDOMINAL DISCOMFORT	2	2	0	0	0
NAUSEA AND VOMITING SYMPTOMS					
NAUSEA	13	11	2	0	0
VOMITING	5	4	1	0	0
ORAL SOFT TISSUE CONDITIONS					
ORAL SOFT TISSUE SIGNS AND SYMPTOMS					
HYPOAESTHESIA ORAL	0	0	0	0	0
PARAESTHESIA ORAL	0	0	0	0	0
TONGUE CONDITIONS					
TONGUE SIGNS AND SYMPTOMS					
GLOSSODYNIA	0	0	0	0	0
**GENERAL DISORDERS AND ADMINISTRATION SITE CONDITIONS**					
ADMINISTRATION SITE REACTIONS					
ADMINISTRATION SITE REACTIONS NEC					
PUNCTURE SITE BRUISE	0	0	0	0	0
PUNCTURE SITE PAIN	0	0	0	0	0
INJECTION SITE REACTIONS					
INJECTION SITE BRUISING	0	0	0	0	0
INJECTION SITE ERYTHEMA	0	0	0	0	0
INJECTION SITE MASS	0	0	0	0	0
INJECTION SITE PAIN	5	4	1	0	0
INJECTION SITE REACTION	0	0	0	0	0
INJECTION SITE URTICARIA	0	0	0	0	0
INJECTION SITE WARMTH	0	0	0	0	0
VACCINATION SITE REACTIONS					
VACCINATION SITE PAIN	2	1	1	0	0
BODY TEMPERATURE CONDITIONS					
FEBRILE DISORDERS					
PYREXIA	51	47	4	0	0
GENERAL SYSTEM DISORDERS NEC					
ASTHENIC CONDITIONS					
ASTHENIA	0	0	0	0	0
FATIGUE	46	34	12	0	0
MALAISE	2	2	0	0	0
FEELINGS AND SENSATIONS NEC					
CHILLS	32	29	3	0	0
FEELING ABNORMAL	0	0	0	0	0
FEELING COLD	2	2	0	0	0
FEELING HOT	1	1	0	0	0
FEELING OF BODY TEMPERATURE CHANGE	1	1	0	0	0
HANGOVER	0	0	0	0	0
HUNGER	1	1	0	0	0
THIRST	1	1	0	0	0
GENERAL SIGNS AND SYMPTOMS NEC					
CRYING	1	1	0	0	0
ILLNESS	3	3	0	0	0
INFLUENZA LIKE ILLNESS	6	6	0	0	0

LOCAL REACTION	0	0	0	0	0
PERIPHERAL SWELLING	0	0	0	0	0
SWELLING	0	0	0	0	0
PAIN AND DISCOMFORT NEC					
AXILLARY PAIN	0	0	0	0	0
CHEST PAIN	0	0	0	0	0
PAIN	11	8	3	0	0
TENDERNESS	2	2	0	0	0
**INFECTIONS AND INFESTATIONS**					
BACTERIAL INFECTIOUS DISORDERS					
BACTERIAL INFECTIONS NEC					
PERIORBITAL CELLULITIS	0	0	0	0	0
INFECTIONS - PATHOGEN UNSPECIFIED					
BREAST INFECTIONS					
MASTITIS	0	0	0	0	0
UPPER RESPIRATORY TRACT INFECTIONS					
LARYNGITIS	0	0	0	0	0
NASOPHARYNGITIS	2	2	0	0	0
VIRAL INFECTIOUS DISORDERS					
HERPES VIRAL INFECTIONS					
GENITAL HERPES	0	0	0	0	0
HERPES ZOSTER	0	0	0	0	0
ORAL HERPES	0	0	0	0	0
INFLUENZA VIRAL INFECTIONS					
INFLUENZA	7	6	1	0	0
VIRAL INFECTIONS NEC					
VIRAL DIARRHOEA	0	0	0	0	0
**INJURY, POISONING AND PROCEDURAL COMPLICATIONS**					
INJURIES NEC					
SKIN INJURIES NEC					
CONTUSION	1	1	0	0	0
PROCEDURAL RELATED INJURIES AND COMPLICATIONS NEC					
NON-SITE SPECIFIC PROCEDURAL COMPLICATIONS					
INJECTION RELATED REACTION	0	0	0	0	0
**INVESTIGATIONS**					
CARDIAC AND VASCULAR INVESTIGATIONS (EXCL ENZYME TESTS)					
HEART RATE AND PULSE INVESTIGATIONS					
HEART RATE	1	1	0	0	0
HEART RATE INCREASED	1	1	0	0	0
PHYSICAL EXAMINATION AND ORGAN SYSTEM STATUS TOPICS					
PHYSICAL EXAMINATION PROCEDURES AND ORGAN SYSTEM STATUS					
BODY TEMPERATURE INCREASED	1	1	0	0	0
WATER, ELECTROLYTE AND MINERAL INVESTIGATIONS					
WATER AND ELECTROLYTE ANALYSES NEC					
VOLUME BLOOD	0	0	0	0	0
**METABOLISM AND NUTRITION DISORDERS**					
APPETITE AND GENERAL NUTRITIONAL DISORDERS					
APPETITE DISORDERS					
DECREASED APPETITE	3	2	1	0	0
ELECTROLYTE AND FLUID BALANCE CONDITIONS					
TOTAL FLUID VOLUME DECREASED					
DEHYDRATION	1	1	0	0	0
**MUSCULOSKELETAL AND CONNECTIVE TISSUE DISORDERS**					

BONE DISORDERS (EXCL CONGENITAL AND FRACTURES)					
BONE RELATED SIGNS AND SYMPTOMS					
PAIN IN JAW	1	0	1	0	0
JOINT DISORDERS					
JOINT RELATED SIGNS AND SYMPTOMS					
ARTHRALGIA	5	5	0	0	0
MUSCLE DISORDERS					
MUSCLE PAINS					
MYALGIA	24	20	4	0	0
MUSCLE RELATED SIGNS AND SYMPTOMS NEC					
MUSCLE FATIGUE	1	1	0	0	0
MUSCLE SPASMS	0	0	0	0	0
MUSCLE TONE ABNORMALITIES					
TRISMUS	0	0	0	0	0
MUSCULOSKELETAL AND CONNECTIVE TISSUE DISORDERS NEC					
MUSCULOSKELETAL AND CONNECTIVE TISSUE CONDITIONS NEC					
MUSCULOSKELETAL STIFFNESS	0	0	0	0	0
BACK PAIN	2	2	0	0	0
LIMB DISCOMFORT	4	4	0	0	0
NECK PAIN	0	0	0	0	0
PAIN IN EXTREMITY	21	18	3	0	0
**NERVOUS SYSTEM DISORDERS**					
CRANIAL NERVE DISORDERS (EXCL NEOPLASMS)					
FACIAL CRANIAL NERVE DISORDERS					
BELL'S PALSY	0	0	0	0	0
OLFACTORY NERVE DISORDERS					
ANOSMIA	0	0	0	0	0
PAROSMIA	1	0	1	0	0
HEADACHES					
HEADACHES NEC					
CLUSTER HEADACHE	0	0	0	0	0
HEADACHE	54	43	11	0	0
SINUS HEADACHE	3	2	1	0	0
TENSION HEADACHE	3	3	0	0	0
MIGRAINE HEADACHES					
MIGRAINE WITH AURA	0	0	0	0	0
TYPICAL AURA WITHOUT HEADACHE	0	0	0	0	0
MENTAL IMPAIRMENT DISORDERS					
MENTAL IMPAIRMENT (EXCL DEMENTIA AND MEMORY LOSS)					
DISTURBANCE IN ATTENTION	0	0	0	0	0
MOVEMENT DISORDERS (INCL PARKINSONISM)					
DYSKINESIAS AND MOVEMENT DISORDERS NEC					
CLUMSINESS	1	1	0	0	0
TREMOR (EXCL CONGENITAL)					
TREMOR	3	3	0	0	0
NEUROLOGICAL DISORDERS NEC					
DISTURBANCES IN CONSCIOUSNESS NEC					
LETHARGY	2	2	0	0	0
SOMNOLENCE	1	1	0	0	0
SYNCOPE	0	0	0	0	0
NEUROLOGICAL SIGNS AND SYMPTOMS NEC					
AGITATION NEONATAL	0	0	0	0	0
BRAIN FOG	1	1	0	0	0

DIZZINESS	9	8	1	0	0
DIZZINESS EXERTIONAL	0	0	0	0	0
PRESYNCOPE	1	1	0	0	0
PARAESTHESIAS AND DYSAESTHESIAS					
PARAESTHESIA	1	1	0	0	0
SENSORY ABNORMALITIES NEC					
DYSGEUSIA	1	1	0	0	0
NEURALGIA	0	0	0	0	0
TASTE DISORDER	0	0	0	0	0
SEIZURES (INCL SUBTYPES)					
SEIZURES AND SEIZURE DISORDERS NEC					
SEIZURE	0	0	0	0	0
**PREGNANCY, PUERPERIUM AND PERINATAL CONDITIONS**					
ABORTIONS AND STILLBIRTH					
ABORTIONS SPONTANEOUS					
ABORTION SPONTANEOUS	5	3	2	0	0
STILLBIRTH AND FOETAL DEATH					
FOETAL DEATH	1	1	0	0	0
FOETAL COMPLICATIONS					
FOETAL COMPLICATIONS NEC					
FOETAL DISORDER	0	0	0	0	0
FOETAL HYPOKINESIA	0	0	0	0	0
FOETAL GROWTH COMPLICATIONS					
FOETAL MACROSOMIA	0	0	0	0	0
MATERNAL COMPLICATIONS OF PREGNANCY					
MATERNAL COMPLICATIONS OF PREGNANCY NEC					
MORNING SICKNESS	0	0	0	0	0
PLACENTAL, AMNIOTIC AND CAVITY DISORDERS (EXCL HAEMORRHAGES)					
PLACENTAL ABNORMALITIES (EXCL NEOPLASMS)					
PLACENTAL INFARCTION	1	1	0	0	0
PREGNANCY, LABOUR, DELIVERY AND POSTPARTUM CONDITIONS					
NORMAL PREGNANCY, LABOUR AND DELIVERY					
PREGNANCY	0	0	0	0	0
UTERINE CONTRACTIONS DURING PREGNANCY	0	0	0	0	0
**PSYCHIATRIC DISORDERS**					
ANXIETY DISORDERS AND SYMPTOMS					
ANXIETY SYMPTOMS					
AGITATION	0	0	0	0	0
ANXIETY	0	0	0	0	0
COGNITIVE AND ATTENTION DISORDERS AND DISTURBANCES					
COGNITIVE AND ATTENTION DISORDERS AND DISTURBANCES NEC					
MENTAL FATIGUE	0	0	0	0	0
DELIRIA (INCL CONFUSION)					
CONFUSION AND DISORIENTATION					
CONFUSIONAL STATE	0	0	0	0	0
DEPRESSED MOOD DISORDERS AND DISTURBANCES					
MOOD ALTERATIONS WITH DEPRESSIVE SYMPTOMS					
DEPRESSED MOOD	0	0	0	0	0
DISTURBANCES IN THINKING AND PERCEPTION					
HALLUCINATIONS (EXCL SLEEP-RELATED)					
HALLUCINATION	0	0	0	0	0
MOOD DISORDERS AND DISTURBANCES NEC					
EMOTIONAL AND MOOD DISTURBANCES NEC					

EMOTIONAL DISORDER	0	0	0	0	0
SLEEP DISORDERS AND DISTURBANCES					
DISTURBANCES IN INITIATING AND MAINTAINING SLEEP					
INITIAL INSOMNIA	0	0	0	0	0
INSOMNIA	3	2	1	0	0
**REPRODUCTIVE SYSTEM AND BREAST DISORDERS**					
MENSTRUAL CYCLE AND UTERINE BLEEDING DISORDERS					
MENSTRUATION AND UTERINE BLEEDING NEC					
MENSTRUAL DISORDER	0	0	0	0	0
MENSTRUATION WITH DECREASED BLEEDING					
MENSTRUATION DELAYED	1	0	1	0	0
OLIGOMENORRHOEA	0	0	0	0	0
MENSTRUATION WITH INCREASED BLEEDING					
HEAVY MENSTRUAL BLEEDING	0	0	0	0	0
VULVOVAGINAL DISORDERS (EXCL INFECTIONS AND INFLAMMATIONS)					
VULVOVAGINAL DISORDERS NEC					
VAGINAL HAEMORRHAGE	1	1	0	0	0
**RESPIRATORY, THORACIC AND MEDIASTINAL DISORDERS**					
RESPIRATORY DISORDERS NEC					
BREATHING ABNORMALITIES					
DYSPNOEA	1	0	1	0	0
COUGHING AND ASSOCIATED SYMPTOMS					
COUGH	1	1	0	0	0
RESPIRATORY TRACT SIGNS AND SYMPTOMS					
UPPER RESPIRATORY TRACT SIGNS AND SYMPTOMS					
DRY THROAT	0	0	0	0	0
OROPHARYNGEAL PAIN	3	3	0	0	0
PARANASAL SINUS DISCOMFORT	0	0	0	0	0
RHINORRHOEA	1	1	0	0	0
SINUS PAIN	0	0	0	0	0
SNEEZING	0	0	0	0	0
UPPER RESPIRATORY TRACT DISORDERS (EXCL INFECTIONS)					
NASAL CONGESTION AND INFLAMMATIONS					
NASAL CONGESTION	0	0	0	0	0
**SKIN AND SUBCUTANEOUS TISSUE DISORDERS**					
ANGIOEDEMA AND URTICARIA					
URTICARIAS					
URTICARIA	0	0	0	0	0
CORNIFICATION AND DYSTROPHIC SKIN DISORDERS					
SKIN DYSTROPHIES					
HYPERTROPHIC SCAR	0	0	0	0	0
EPIDERMAL AND DERMAL CONDITIONS					
DERMAL AND EPIDERMAL CONDITIONS NEC					
SKIN WARM	0	0	0	0	0
DERMATITIS AND ECZEMA					
DERMATITIS ALLERGIC	1	1	0	0	0
ERYTHEMAS					
ERYTHEMA	0	0	0	0	0
PRURITUS NEC					
PRURITUS	1	1	0	0	0
RASHES, ERUPTIONS AND EXANTHEMS NEC					
RASH	1	1	0	0	0
RASH PRURITIC	1	1	0	0	0

SKIN APPENDAGE CONDITIONS					
APOCRINE AND ECCRINE GLAND DISORDERS					
COLD SWEAT	1	1	0	0	0
HYPERHIDROSIS	3	3	0	0	0
NIGHT SWEATS	0	0	0	0	0
**VASCULAR DISORDERS**					
DECREASED AND NONSPECIFIC BLOOD PRESSURE DISORDERS AND SHOCK					
BLOOD PRESSURE DISORDERS NEC					
BLOOD PRESSURE FLUCTUATION	1	1	0	0	0
VASCULAR HYPOTENSIVE DISORDERS					
HYPOTENSION	0	0	0	0	0
EMBOLISM AND THROMBOSIS					
NON-SITE SPECIFIC EMBOLISM AND THROMBOSIS					
EMBOLISM	0	0	0	0	0
VASCULAR DISORDERS NEC					
NON-SITE SPECIFIC VASCULAR DISORDERS NEC					
VASCULAR PAIN	0	0	0	0	0
PERIPHERAL VASCULAR DISORDERS NEC					
HOT FLUSH	0	0	0	0	0
VASCULAR HAEMORRHAGIC DISORDERS					
HAEMORRHAGES NEC					
HAEMORRHAGE	0	0	0	0	0
**TOTAL ADR EVENTS**	***377***	***318***	***59***	***0***	***0***

SUPPLEMENTARY TABLE 27. Moderna COVID-19 vaccine: ADR listing for events reported by pregnant females, in those reporting any vaccination dose

	Individuals with any vaccination dose: ADR Counts				
**MEDDRA REACTION TERM (SOC, *HLGT*, *HLT*, PT)**	All doses	1st dose	2nd dose	3rd dose	Other doses
(freetext)	0	0	0	0	0
**BLOOD AND LYMPHATIC SYSTEM DISORDERS**					
SPLEEN, LYMPHATIC AND RETICULOENDOTHELIAL SYSTEM DISORDERS					
LYMPHATIC SYSTEM DISORDERS NEC	0	0	0	0	0
LYMPH NODE PAIN	1	1	0	0	0
LYMPHADENITIS	2	1	1	0	0
LYMPHADENOPATHY					
**CARDIAC DISORDERS**					
CARDIAC ARRHYTHMIAS					
RATE AND RHYTHM DISORDERS NEC					
TACHYCARDIA	0	0	0	0	0
CARDIAC DISORDERS, SIGNS AND SYMPTOMS NEC					
CARDIAC SIGNS AND SYMPTOMS NEC					
PALPITATIONS	2	1	1	0	0
**EAR AND LABYRINTH DISORDERS**					
AURAL DISORDERS NEC					
EAR DISORDERS NEC					
EAR PAIN	0	0	0	0	0
INNER EAR AND VIIITH CRANIAL NERVE DISORDERS					
INNER EAR SIGNS AND SYMPTOMS					
TINNITUS	1	0	0	1	0
VERTIGO	0	0	0	0	0
**EYE DISORDERS**					
EYE DISORDERS NEC					
OCULAR DISORDERS NEC					
EYE PAIN	1	1	0	0	0
OCULAR SENSORY SYMPTOMS NEC					
OCULAR SENSATION DISORDERS					
ASTHENOPIA	0	0	0	0	0
PHOTOPHOBIA	0	0	0	0	0
VISION DISORDERS					
VISUAL DISORDERS NEC					
VISION BLURRED	1	1	0	0	0
**GASTROINTESTINAL DISORDERS**					
DENTAL AND GINGIVAL CONDITIONS					
DENTAL DISORDERS NEC					
TEETHING	0	0	0	0	0
GASTROINTESTINAL INFLAMMATORY CONDITIONS					
GASTROINTESTINAL INFLAMMATORY DISORDERS NEC					
GASTROINTESTINAL TRACT IRRITATION	0	0	0	0	0
GASTROINTESTINAL MOTILITY AND DEFAECATION CONDITIONS					
DIARRHOEA (EXCL INFECTIVE)					
DIARRHOEA	1	1	0	0	0
GASTROINTESTINAL ATONIC AND HYPOMOTILITY DISORDERS NEC					
CONSTIPATION	0	0	0	0	0

GASTROINTESTINAL SIGNS AND SYMPTOMS					
GASTROINTESTINAL AND ABDOMINAL PAINS (EXCL ORAL AND THROAT)					
ABDOMINAL PAIN	0	0	0	0	0
ABDOMINAL PAIN UPPER	0	0	0	0	0
GASTROINTESTINAL SIGNS AND SYMPTOMS NEC					
ABDOMINAL DISCOMFORT	0	0	0	0	0
NAUSEA AND VOMITING SYMPTOMS					
NAUSEA	11	6	3	2	0
VOMITING	4	1	3	0	0
ORAL SOFT TISSUE CONDITIONS					
ORAL SOFT TISSUE SIGNS AND SYMPTOMS					
HYPOAESTHESIA ORAL					
PARAESTHESIA ORAL	0	0	0	0	0
TONGUE CONDITIONS					
TONGUE SIGNS AND SYMPTOMS					
GLOSSODYNIA	0	0	0	0	0
**GENERAL DISORDERS AND ADMINISTRATION SITE CONDITIONS**					
ADMINISTRATION SITE REACTIONS					
ADMINISTRATION SITE REACTIONS NEC					
PUNCTURE SITE BRUISE	1	0	0	1	0
PUNCTURE SITE PAIN	0	0	0	0	0
INJECTION SITE REACTIONS					
INJECTION SITE BRUISING	0	0	0	0	0
INJECTION SITE ERYTHEMA	0	0	0	0	0
INJECTION SITE MASS	1	1	0	0	0
INJECTION SITE PAIN	4	1	1	2	0
INJECTION SITE REACTION	0	0	0	0	0
INJECTION SITE URTICARIA	0	0	0	0	0
INJECTION SITE WARMTH	0	0	0	0	0
VACCINATION SITE REACTIONS					
VACCINATION SITE PAIN	0	0	0	0	0
BODY TEMPERATURE CONDITIONS					
FEBRILE DISORDERS					
PYREXIA	24	5	12	7	0
GENERAL SYSTEM DISORDERS NEC					
ASTHENIC CONDITIONS					
ASTHENIA	2	1	0	1	0
FATIGUE	47	21	14	11	1
MALAISE	4	2	0	1	1
FEELINGS AND SENSATIONS NEC					
CHILLS	14	2	6	6	0
FEELING ABNORMAL	0	0	0	0	0
FEELING COLD	0	0	0	0	0
FEELING HOT	0	0	0	0	0
FEELING OF BODY TEMPERATURE CHANGE	0	0	0	0	0
HANGOVER	0	0	0	0	0
HUNGER	0	0	0	0	0
THIRST	0	0	0	0	0
GENERAL SIGNS AND SYMPTOMS NEC					
CRYING	0	0	0	0	0
ILLNESS	2	1	0	1	0
INFLUENZA LIKE ILLNESS	2	0	1	1	0

LOCAL REACTION	0	0	0	0	0
PERIPHERAL SWELLING	1	1	0	0	0
SWELLING	2	1	0	1	0
PAIN AND DISCOMFORT NEC					
AXILLARY PAIN	1	1	0	0	0
CHEST PAIN	0	0	0	0	0
PAIN	11	3	7	1	0
TENDERNESS	0	0	0	0	0
**INFECTIONS AND INFESTATIONS**					
BACTERIAL INFECTIOUS DISORDERS					
BACTERIAL INFECTIONS NEC					
PERIORBITAL CELLULITIS	0	0	0	0	0
INFECTIONS - PATHOGEN UNSPECIFIED					
BREAST INFECTIONS					
MASTITIS	0	0	0	0	0
UPPER RESPIRATORY TRACT INFECTIONS					
LARYNGITIS	1	1	0	0	0
NASOPHARYNGITIS	3	1	1	1	0
VIRAL INFECTIOUS DISORDERS					
HERPES VIRAL INFECTIONS					
GENITAL HERPES	0	0	0	0	0
HERPES ZOSTER	0	0	0	0	0
ORAL HERPES	0	0	0	0	0
INFLUENZA VIRAL INFECTIONS					
INFLUENZA	8	2	4	2	0
VIRAL INFECTIONS NEC					
VIRAL DIARRHOEA	0	0	0	0	0
**INJURY, POISONING AND PROCEDURAL COMPLICATIONS**					
INJURIES NEC					
SKIN INJURIES NEC					
CONTUSION	0	0	0	0	0
PROCEDURAL RELATED INJURIES AND COMPLICATIONS NEC					
NON-SITE SPECIFIC PROCEDURAL COMPLICATIONS					
INJECTION RELATED REACTION	1	0	0	1	0
**INVESTIGATIONS**					
CARDIAC AND VASCULAR INVESTIGATIONS (EXCL ENZYME TESTS)					
HEART RATE AND PULSE INVESTIGATIONS					
HEART RATE	0	0	0	0	0
HEART RATE INCREASED	0	0	0	0	0
PHYSICAL EXAMINATION AND ORGAN SYSTEM STATUS TOPICS					
PHYSICAL EXAMINATION PROCEDURES AND ORGAN SYSTEM STATUS					
BODY TEMPERATURE INCREASED	1	0	0	1	0
WATER, ELECTROLYTE AND MINERAL INVESTIGATIONS					
WATER AND ELECTROLYTE ANALYSES NEC					
VOLUME BLOOD	0	0	0	0	0
**METABOLISM AND NUTRITION DISORDERS**					
APPETITE AND GENERAL NUTRITIONAL DISORDERS					
APPETITE DISORDERS					
DECREASED APPETITE	0	0	0	0	0
ELECTROLYTE AND FLUID BALANCE CONDITIONS					
TOTAL FLUID VOLUME DECREASED					
DEHYDRATION	0	0	0	0	0
**MUSCULOSKELETAL AND CONNECTIVE TISSUE DISORDERS**					

BONE DISORDERS (EXCL CONGENITAL AND FRACTURES)					
BONE RELATED SIGNS AND SYMPTOMS					
PAIN IN JAW	1	0	1	0	0
JOINT DISORDERS					
JOINT RELATED SIGNS AND SYMPTOMS					
ARTHRALGIA	5	3	2	0	0
MUSCLE DISORDERS					
MUSCLE PAINS					
MYALGIA	15	5	6	4	0
MUSCLE RELATED SIGNS AND SYMPTOMS NEC					
MUSCLE FATIGUE	1	0	0	1	0
MUSCLE SPASMS	0	0	0	0	0
MUSCLE TONE ABNORMALITIES					
TRISMUS	1	0	1	0	0
MUSCULOSKELETAL AND CONNECTIVE TISSUE DISORDERS NEC					
MUSCULOSKELETAL AND CONNECTIVE TISSUE CONDITIONS NEC					
MUSCULOSKELETAL STIFFNESS	3	1	2	0	0
BACK PAIN	3	1	2	0	0
LIMB DISCOMFORT	3	2	1	0	0
NECK PAIN	1	0	1	0	0
PAIN IN EXTREMITY	59	49	6	4	0
**NERVOUS SYSTEM DISORDERS**					
CRANIAL NERVE DISORDERS (EXCL NEOPLASMS)					
FACIAL CRANIAL NERVE DISORDERS					
BELL'S PALSY	0	0	0	0	0
OLFACTORY NERVE DISORDERS					
ANOSMIA	0	0	0	0	0
PAROSMIA	0	0	0	0	0
HEADACHES					
HEADACHES NEC					
CLUSTER HEADACHE	0	0	0	0	0
HEADACHE	31	12	14	5	0
SINUS HEADACHE	0	0	0	0	0
TENSION HEADACHE	2	1	1	0	0
MIGRAINE HEADACHES					
MIGRAINE WITH AURA	1	0	1	0	0
TYPICAL AURA WITHOUT HEADACHE	0	0	0	0	0
MENTAL IMPAIRMENT DISORDERS					
MENTAL IMPAIRMENT (EXCL DEMENTIA AND MEMORY LOSS)					
DISTURBANCE IN ATTENTION	0	0	0	0	0
MOVEMENT DISORDERS (INCL PARKINSONISM)					
DYSKINESIAS AND MOVEMENT DISORDERS NEC					
CLUMSINESS	0	0	0	0	0
TREMOR (EXCL CONGENITAL)					
TREMOR	1	0	1	0	0
NEUROLOGICAL DISORDERS NEC					
DISTURBANCES IN CONSCIOUSNESS NEC					
LETHARGY	0	0	0	0	0
SOMNOLENCE	1	0	1	0	0
SYNCOPE	0	0	0	0	0
NEUROLOGICAL SIGNS AND SYMPTOMS NEC					
AGITATION NEONATAL	0	0	0	0	0
BRAIN FOG	0	0	0	0	0

DIZZINESS	8	4	2	2	0
DIZZINESS EXERTIONAL	1	1	0	0	0
PRESYNCOPE	0	0	0	0	0
PARAESTHESIAS AND DYSAESTHESIAS					
PARAESTHESIA	0	0	0	0	0
SENSORY ABNORMALITIES NEC					
DYSGEUSIA	0	0	0	0	0
NEURALGIA	0	0	0	0	0
TASTE DISORDER	0	0	0	0	0
SEIZURES (INCL SUBTYPES)					
SEIZURES AND SEIZURE DISORDERS NEC					
SEIZURE	0	0	0	0	0
**PREGNANCY, PUERPERIUM AND PERINATAL CONDITIONS**					
ABORTIONS AND STILLBIRTH					
ABORTIONS SPONTANEOUS					
ABORTION SPONTANEOUS	1	1	0	0	0
STILLBIRTH AND FOETAL DEATH					
FOETAL DEATH	0	0	0	0	0
FOETAL COMPLICATIONS					
FOETAL COMPLICATIONS NEC					
FOETAL DISORDER	0	0	0	0	0
FOETAL HYPOKINESIA	0	0	0	0	0
FOETAL GROWTH COMPLICATIONS					
FOETAL MACROSOMIA	0	0	0	0	0
MATERNAL COMPLICATIONS OF PREGNANCY					
MATERNAL COMPLICATIONS OF PREGNANCY NEC					
MORNING SICKNESS	0	0	0	0	0
PLACENTAL, AMNIOTIC AND CAVITY DISORDERS (EXCL HAEMORRHAGES)					
PLACENTAL ABNORMALITIES (EXCL NEOPLASMS)					
PLACENTAL INFARCTION	0	0	0	0	0
PREGNANCY, LABOUR, DELIVERY AND POSTPARTUM CONDITIONS					
NORMAL PREGNANCY, LABOUR AND DELIVERY					
PREGNANCY	0	0	0	0	0
UTERINE CONTRACTIONS DURING PREGNANCY	0	0	0	0	0
**PSYCHIATRIC DISORDERS**					
ANXIETY DISORDERS AND SYMPTOMS					
ANXIETY SYMPTOMS					
AGITATION	0	0	0	0	0
ANXIETY	0	0	0	0	0
COGNITIVE AND ATTENTION DISORDERS AND DISTURBANCES					
COGNITIVE AND ATTENTION DISORDERS AND DISTURBANCES NEC					
MENTAL FATIGUE	0	0	0	0	0
DELIRIA (INCL CONFUSION)					
CONFUSION AND DISORIENTATION					
CONFUSIONAL STATE	0	0	0	0	0
DEPRESSED MOOD DISORDERS AND DISTURBANCES					
MOOD ALTERATIONS WITH DEPRESSIVE SYMPTOMS					
DEPRESSED MOOD	0	0	0	0	0
DISTURBANCES IN THINKING AND PERCEPTION					
HALLUCINATIONS (EXCL SLEEP-RELATED)					
HALLUCINATION	0	0	0	0	0
MOOD DISORDERS AND DISTURBANCES NEC					
EMOTIONAL AND MOOD DISTURBANCES NEC					

EMOTIONAL DISORDER	0	0	0	0	0
SLEEP DISORDERS AND DISTURBANCES					
DISTURBANCES IN INITIATING AND MAINTAINING SLEEP					
INITIAL INSOMNIA	0	0	0	0	0
INSOMNIA	1	1	0	0	0
**REPRODUCTIVE SYSTEM AND BREAST DISORDERS**					
MENSTRUAL CYCLE AND UTERINE BLEEDING DISORDERS					
MENSTRUATION AND UTERINE BLEEDING NEC					
MENSTRUAL DISORDER	0	0	0	0	0
MENSTRUATION WITH DECREASED BLEEDING					
MENSTRUATION DELAYED	0	0	0	0	0
OLIGOMENORRHOEA	0	0	0	0	0
MENSTRUATION WITH INCREASED BLEEDING					
HEAVY MENSTRUAL BLEEDING	0	0	0	0	0
VULVOVAGINAL DISORDERS (EXCL INFECTIONS AND INFLAMMATIONS)					
VULVOVAGINAL DISORDERS NEC					
VAGINAL HAEMORRHAGE	0	0	0	0	0
**RESPIRATORY, THORACIC AND MEDIASTINAL DISORDERS**					
RESPIRATORY DISORDERS NEC					
BREATHING ABNORMALITIES					
DYSPNOEA	3	1	2	0	0
COUGHING AND ASSOCIATED SYMPTOMS					
COUGH	0	0	0	0	0
RESPIRATORY TRACT SIGNS AND SYMPTOMS					
UPPER RESPIRATORY TRACT SIGNS AND SYMPTOMS					
DRY THROAT	0	0	0	0	0
OROPHARYNGEAL PAIN	2	2	0	0	0
PARANASAL SINUS DISCOMFORT	0	0	0	0	0
RHINORRHOEA	1	0	0	1	0
SINUS PAIN	1	1	0	0	0
SNEEZING	0	0	0	0	0
UPPER RESPIRATORY TRACT DISORDERS (EXCL INFECTIONS)					
NASAL CONGESTION AND INFLAMMATIONS					
NASAL CONGESTION	1	0	1	0	0
**SKIN AND SUBCUTANEOUS TISSUE DISORDERS**					
ANGIOEDEMA AND URTICARIA					
URTICARIAS					
URTICARIA	0	0	0	0	0
CORNIFICATION AND DYSTROPHIC SKIN DISORDERS					
SKIN DYSTROPHIES					
HYPERTROPHIC SCAR	0	0	0	0	0
EPIDERMAL AND DERMAL CONDITIONS					
DERMAL AND EPIDERMAL CONDITIONS NEC					
SKIN WARM	0	0	0	0	0
DERMATITIS AND ECZEMA					
DERMATITIS ALLERGIC	0	0	0	0	0
ERYTHEMAS					
ERYTHEMA	0	0	0	0	0
PRURITUS NEC					
PRURITUS	2	1	0	1	0
RASHES, ERUPTIONS AND EXANTHEMS NEC					
RASH	3	1	0	2	0
RASH PRURITIC	0	0	0	0	0

SKIN APPENDAGE CONDITIONS					
APOCRINE AND ECCRINE GLAND DISORDERS					
COLD SWEAT	1	1	0	0	0
HYPERHIDROSIS	0	0	0	0	0
NIGHT SWEATS	1	0	1	0	0
**VASCULAR DISORDERS**					
DECREASED AND NONSPECIFIC BLOOD PRESSURE DISORDERS AND SHOCK					
BLOOD PRESSURE DISORDERS NEC					
BLOOD PRESSURE FLUCTUATION	0	0	0	0	0
VASCULAR HYPOTENSIVE DISORDERS					
HYPOTENSION	0	0	0	0	0
EMBOLISM AND THROMBOSIS					
NON-SITE SPECIFIC EMBOLISM AND THROMBOSIS					
EMBOLISM	1	1	0	0	0
VASCULAR DISORDERS NEC					
NON-SITE SPECIFIC VASCULAR DISORDERS NEC					
VASCULAR PAIN	0	0	0	0	0
PERIPHERAL VASCULAR DISORDERS NEC					
HOT FLUSH	0	0	0	0	0
VASCULAR HAEMORRHAGIC DISORDERS					
HAEMORRHAGES NEC					
HAEMORRHAGE	0	0	0	0	0
**TOTAL ADR EVENTS**	***309***	***146***	***100***	***61***	***2***

SUPPLEMENTARY TABLE 28. Others/Unknown COVID-19 vaccine: ADR listing for events reported by pregnant females, in those reporting any vaccination dose

	Individuals with any vaccination dose: ADR Counts				
**MEDDRA REACTION TERM (SOC, *HLGT*, *HLT*, PT)**	All doses	1st dose	2nd dose	3rd dose	Other doses
(freetext)	0	0	0	0	0
**BLOOD AND LYMPHATIC SYSTEM DISORDERS**					
SPLEEN, LYMPHATIC AND RETICULOENDOTHELIAL SYSTEM DISORDERS					
LYMPHATIC SYSTEM DISORDERS NEC					
LYMPH NODE PAIN	0	0	0	0	0
LYMPHADENITIS	0	0	0	0	0
LYMPHADENOPATHY	0	0	0	0	0
**CARDIAC DISORDERS**					
CARDIAC ARRHYTHMIAS					
RATE AND RHYTHM DISORDERS NEC					
TACHYCARDIA	0	0	0	0	0
CARDIAC DISORDERS, SIGNS AND SYMPTOMS NEC					
CARDIAC SIGNS AND SYMPTOMS NEC					
PALPITATIONS	0	0	0	0	0
**EAR AND LABYRINTH DISORDERS**					
AURAL DISORDERS NEC					
EAR DISORDERS NEC					
EAR PAIN	0	0	0	0	0
INNER EAR AND VIIITH CRANIAL NERVE DISORDERS					
INNER EAR SIGNS AND SYMPTOMS					
TINNITUS	0	0	0	0	0
VERTIGO	0	0	0	0	0
**EYE DISORDERS**					
EYE DISORDERS NEC					
OCULAR DISORDERS NEC					
EYE PAIN	0	0	0	0	0
OCULAR SENSORY SYMPTOMS NEC					
OCULAR SENSATION DISORDERS					
ASTHENOPIA	0	0	0	0	0
PHOTOPHOBIA	0	0	0	0	0
VISION DISORDERS					
VISUAL DISORDERS NEC					
VISION BLURRED	0	0	0	0	0
**GASTROINTESTINAL DISORDERS**					
DENTAL AND GINGIVAL CONDITIONS					
DENTAL DISORDERS NEC					
TEETHING	0	0	0	0	0
GASTROINTESTINAL INFLAMMATORY CONDITIONS					
GASTROINTESTINAL INFLAMMATORY DISORDERS NEC					
GASTROINTESTINAL TRACT IRRITATION	0	0	0	0	0
GASTROINTESTINAL MOTILITY AND DEFAECATION CONDITIONS					
DIARRHOEA (EXCL INFECTIVE)					
DIARRHOEA	0	0	0	0	0
GASTROINTESTINAL ATONIC AND HYPOMOTILITY DISORDERS NEC					
CONSTIPATION	0	0	0	0	0

GASTROINTESTINAL SIGNS AND SYMPTOMS					
GASTROINTESTINAL AND ABDOMINAL PAINS (EXCL ORAL AND THROAT)					
ABDOMINAL PAIN	0	0	0	0	0
ABDOMINAL PAIN UPPER	0	0	0	0	0
GASTROINTESTINAL SIGNS AND SYMPTOMS NEC					
ABDOMINAL DISCOMFORT	0	0	0	0	0
NAUSEA AND VOMITING SYMPTOMS					
NAUSEA	1	0	0	1	0
VOMITING	0	0	0	0	0
ORAL SOFT TISSUE CONDITIONS					
ORAL SOFT TISSUE SIGNS AND SYMPTOMS					
HYPOAESTHESIA ORAL	0	0	0	0	0
PARAESTHESIA ORAL	0	0	0	0	0
TONGUE CONDITIONS					
TONGUE SIGNS AND SYMPTOMS					
GLOSSODYNIA	0	0	0	0	0
**GENERAL DISORDERS AND ADMINISTRATION SITE CONDITIONS**					
ADMINISTRATION SITE REACTIONS					
ADMINISTRATION SITE REACTIONS NEC					
PUNCTURE SITE BRUISE	0	0	0	0	0
PUNCTURE SITE PAIN	0	0	0	0	0
INJECTION SITE REACTIONS					
INJECTION SITE BRUISING	0	0	0	0	0
INJECTION SITE ERYTHEMA	0	0	0	0	0
INJECTION SITE MASS	0	0	0	0	0
INJECTION SITE PAIN	0	0	0	0	0
INJECTION SITE REACTION	0	0	0	0	0
INJECTION SITE URTICARIA	0	0	0	0	0
INJECTION SITE WARMTH	0	0	0	0	0
VACCINATION SITE REACTIONS					
VACCINATION SITE PAIN	0	0	0	0	0
BODY TEMPERATURE CONDITIONS					
FEBRILE DISORDERS					
PYREXIA	0	0	0	0	0
GENERAL SYSTEM DISORDERS NEC					
ASTHENIC CONDITIONS					
ASTHENIA	1	1	0	0	0
FATIGUE	0	0	0	0	0
MALAISE	0	0	0	0	0
FEELINGS AND SENSATIONS NEC					
CHILLS	0	0	0	0	0
FEELING ABNORMAL	0	0	0	0	0
FEELING COLD	0	0	0	0	0
FEELING HOT	0	0	0	0	0
FEELING OF BODY TEMPERATURE CHANGE	0	0	0	0	0
HANGOVER	0	0	0	0	0
HUNGER	0	0	0	0	0
THIRST	0	0	0	0	0
GENERAL SIGNS AND SYMPTOMS NEC					
CRYING	0	0	0	0	0
ILLNESS	0	0	0	0	0

INFLUENZA LIKE ILLNESS	0	0	0	0	0
LOCAL REACTION	0	0	0	0	0
PERIPHERAL SWELLING	0	0	0	0	0
SWELLING	0	0	0	0	0
PAIN AND DISCOMFORT NEC					
AXILLARY PAIN	0	0	0	0	0
CHEST PAIN	0	0	0	0	0
PAIN	1	0	0	1	0
TENDERNESS	0	0	0	0	0
**INFECTIONS AND INFESTATIONS**					
BACTERIAL INFECTIOUS DISORDERS					
BACTERIAL INFECTIONS NEC					
PERIORBITAL CELLULITIS	0	0	0	0	0
INFECTIONS - PATHOGEN UNSPECIFIED					
BREAST INFECTIONS					
MASTITIS	0	0	0	0	0
UPPER RESPIRATORY TRACT INFECTIONS					
LARYNGITIS	0	0	0	0	0
NASOPHARYNGITIS	0	0	0	0	0
VIRAL INFECTIOUS DISORDERS					
HERPES VIRAL INFECTIONS					
GENITAL HERPES	0	0	0	0	0
HERPES ZOSTER	0	0	0	0	0
ORAL HERPES	0	0	0	0	0
INFLUENZA VIRAL INFECTIONS					
INFLUENZA	0	0	0	0	0
VIRAL INFECTIONS NEC					
VIRAL DIARRHOEA	0	0	0	0	0
**INJURY, POISONING AND PROCEDURAL COMPLICATIONS**					
INJURIES NEC					
SKIN INJURIES NEC					
CONTUSION	0	0	0	0	0
PROCEDURAL RELATED INJURIES AND COMPLICATIONS NEC					
NON-SITE SPECIFIC PROCEDURAL COMPLICATIONS					
INJECTION RELATED REACTION	0	0	0	0	0
**INVESTIGATIONS**					
CARDIAC AND VASCULAR INVESTIGATIONS (EXCL ENZYME TESTS)					
HEART RATE AND PULSE INVESTIGATIONS					
HEART RATE	0	0	0	0	0
HEART RATE INCREASED	0	0	0	0	0
PHYSICAL EXAMINATION AND ORGAN SYSTEM STATUS TOPICS					
PHYSICAL EXAMINATION PROCEDURES AND ORGAN SYSTEM STATUS					
BODY TEMPERATURE INCREASED	0	0	0	0	0
WATER, ELECTROLYTE AND MINERAL INVESTIGATIONS					
WATER AND ELECTROLYTE ANALYSES NEC					
VOLUME BLOOD	0	0	0	0	0
**METABOLISM AND NUTRITION DISORDERS**					
APPETITE AND GENERAL NUTRITIONAL DISORDERS					
APPETITE DISORDERS					
DECREASED APPETITE	0	0	0	0	0
ELECTROLYTE AND FLUID BALANCE CONDITIONS					
TOTAL FLUID VOLUME DECREASED					
DEHYDRATION	0	0	0	0	0

**MUSCULOSKELETAL AND CONNECTIVE TISSUE DISORDERS**					
BONE DISORDERS (EXCL CONGENITAL AND FRACTURES)					
BONE RELATED SIGNS AND SYMPTOMS					
PAIN IN JAW	0	0	0	0	0
JOINT DISORDERS					
JOINT RELATED SIGNS AND SYMPTOMS					
ARTHRALGIA	0	0	0	0	0
MUSCLE DISORDERS					
MUSCLE PAINS					
MYALGIA	0	0	0	0	0
MUSCLE RELATED SIGNS AND SYMPTOMS NEC					
MUSCLE FATIGUE	0	0	0	0	0
MUSCLE SPASMS	0	0	0	0	0
MUSCLE TONE ABNORMALITIES					
TRISMUS	0	0	0	0	0
MUSCULOSKELETAL AND CONNECTIVE TISSUE DISORDERS NEC					
MUSCULOSKELETAL AND CONNECTIVE TISSUE CONDITIONS NEC					
MUSCULOSKELETAL STIFFNESS	0	0	0	0	0
BACK PAIN	0	0	0	0	0
LIMB DISCOMFORT	0	0	0	0	0
NECK PAIN	0	0	0	0	0
PAIN IN EXTREMITY	0	0	0	0	0
**NERVOUS SYSTEM DISORDERS**					
CRANIAL NERVE DISORDERS (EXCL NEOPLASMS)					
FACIAL CRANIAL NERVE DISORDERS					
BELL'S PALSY	0	0	0	0	0
OLFACTORY NERVE DISORDERS					
ANOSMIA	0	0	0	0	0
PAROSMIA	0	0	0	0	0
HEADACHES					
HEADACHES NEC					
CLUSTER HEADACHE	0	0	0	0	0
HEADACHE	1	0	0	1	0
SINUS HEADACHE	0	0	0	0	0
TENSION HEADACHE	0	0	0	0	0
MIGRAINE HEADACHES					
MIGRAINE WITH AURA	0	0	0	0	0
TYPICAL AURA WITHOUT HEADACHE	0	0	0	0	0
MENTAL IMPAIRMENT DISORDERS					
MENTAL IMPAIRMENT (EXCL DEMENTIA AND MEMORY LOSS)					
DISTURBANCE IN ATTENTION	0	0	0	0	0
MOVEMENT DISORDERS (INCL PARKINSONISM)					
DYSKINESIAS AND MOVEMENT DISORDERS NEC					
CLUMSINESS	0	0	0	0	0
TREMOR (EXCL CONGENITAL)					
TREMOR	0	0	0	0	0
NEUROLOGICAL DISORDERS NEC					
DISTURBANCES IN CONSCIOUSNESS NEC					
LETHARGY	0	0	0	0	0
SOMNOLENCE	0	0	0	0	0
SYNCOPE	0	0	0	0	0
NEUROLOGICAL SIGNS AND SYMPTOMS NEC					
AGITATION NEONATAL	0	0	0	0	0

BRAIN FOG	0	0	0	0	0
DIZZINESS	0	0	0	0	0
DIZZINESS EXERTIONAL	0	0	0	0	0
PRESYNCOPE	0	0	0	0	0
PARAESTHESIAS AND DYSAESTHESIAS					
PARAESTHESIA	0	0	0	0	0
SENSORY ABNORMALITIES NEC					
DYSGEUSIA	0	0	0	0	0
NEURALGIA	0	0	0	0	0
TASTE DISORDER	0	0	0	0	0
SEIZURES (INCL SUBTYPES)					
SEIZURES AND SEIZURE DISORDERS NEC					
SEIZURE	0	0	0	0	0
**PREGNANCY, PUERPERIUM AND PERINATAL CONDITIONS**					
ABORTIONS AND STILLBIRTH					
ABORTIONS SPONTANEOUS					
ABORTION SPONTANEOUS	0	0	0	0	0
STILLBIRTH AND FOETAL DEATH					
FOETAL DEATH	0	0	0	0	0
FOETAL COMPLICATIONS					
FOETAL COMPLICATIONS NEC					
FOETAL DISORDER	1	1	0	0	0
FOETAL HYPOKINESIA	0	0	0	0	0
FOETAL GROWTH COMPLICATIONS					
FOETAL MACROSOMIA	0	0	0	0	0
MATERNAL COMPLICATIONS OF PREGNANCY					
MATERNAL COMPLICATIONS OF PREGNANCY NEC					
MORNING SICKNESS	0	0	0	0	0
PLACENTAL, AMNIOTIC AND CAVITY DISORDERS (EXCL HAEMORRHAGES)					
PLACENTAL ABNORMALITIES (EXCL NEOPLASMS)					
PLACENTAL INFARCTION	0	0	0	0	0
PREGNANCY, LABOUR, DELIVERY AND POSTPARTUM CONDITIONS					
NORMAL PREGNANCY, LABOUR AND DELIVERY					
PREGNANCY	0	0	0	0	0
UTERINE CONTRACTIONS DURING PREGNANCY	0	0	0	0	0
**PSYCHIATRIC DISORDERS**					
ANXIETY DISORDERS AND SYMPTOMS					
ANXIETY SYMPTOMS					
AGITATION	0	0	0	0	0
ANXIETY	0	0	0	0	0
COGNITIVE AND ATTENTION DISORDERS AND DISTURBANCES					
COGNITIVE AND ATTENTION DISORDERS AND DISTURBANCES NEC					
MENTAL FATIGUE	0	0	0	0	0
DELIRIA (INCL CONFUSION)					
CONFUSION AND DISORIENTATION					
CONFUSIONAL STATE	0	0	0	0	0
DEPRESSED MOOD DISORDERS AND DISTURBANCES					
MOOD ALTERATIONS WITH DEPRESSIVE SYMPTOMS					
DEPRESSED MOOD	0	0	0	0	0
DISTURBANCES IN THINKING AND PERCEPTION					
HALLUCINATIONS (EXCL SLEEP-RELATED)					
HALLUCINATION	0	0	0	0	0
MOOD DISORDERS AND DISTURBANCES NEC					

EMOTIONAL AND MOOD DISTURBANCES NEC					
EMOTIONAL DISORDER	0	0	0	0	0
SLEEP DISORDERS AND DISTURBANCES					
DISTURBANCES IN INITIATING AND MAINTAINING SLEEP					
INITIAL INSOMNIA	0	0	0	0	0
INSOMNIA	0	0	0	0	0
**REPRODUCTIVE SYSTEM AND BREAST DISORDERS**					
MENSTRUAL CYCLE AND UTERINE BLEEDING DISORDERS					
MENSTRUATION AND UTERINE BLEEDING NEC					
MENSTRUAL DISORDER	0	0	0	0	0
MENSTRUATION WITH DECREASED BLEEDING					
MENSTRUATION DELAYED	0	0	0	0	0
OLIGOMENORRHOEA	0	0	0	0	0
MENSTRUATION WITH INCREASED BLEEDING					
HEAVY MENSTRUAL BLEEDING	0	0	0	0	0
VULVOVAGINAL DISORDERS (EXCL INFECTIONS AND INFLAMMATIONS)					
VULVOVAGINAL DISORDERS NEC					
VAGINAL HAEMORRHAGE	0	0	0	0	0
**RESPIRATORY, THORACIC AND MEDIASTINAL DISORDERS**					
RESPIRATORY DISORDERS NEC					
BREATHING ABNORMALITIES					
DYSPNOEA	0	0	0	0	0
COUGHING AND ASSOCIATED SYMPTOMS					
COUGH	0	0	0	0	0
RESPIRATORY TRACT SIGNS AND SYMPTOMS					
UPPER RESPIRATORY TRACT SIGNS AND SYMPTOMS					
DRY THROAT	0	0	0	0	0
OROPHARYNGEAL PAIN	0	0	0	0	0
PARANASAL SINUS DISCOMFORT	0	0	0	0	0
RHINORRHOEA	0	0	0	0	0
SINUS PAIN	0	0	0	0	0
SNEEZING	0	0	0	0	0
UPPER RESPIRATORY TRACT DISORDERS (EXCL INFECTIONS)					
NASAL CONGESTION AND INFLAMMATIONS					
NASAL CONGESTION	0	0	0	0	0
**SKIN AND SUBCUTANEOUS TISSUE DISORDERS**					
ANGIOEDEMA AND URTICARIA					
URTICARIAS					
URTICARIA	0	0	0	0	0
CORNIFICATION AND DYSTROPHIC SKIN DISORDERS					
SKIN DYSTROPHIES					
HYPERTROPHIC SCAR	0	0	0	0	0
EPIDERMAL AND DERMAL CONDITIONS					
DERMAL AND EPIDERMAL CONDITIONS NEC					
SKIN WARM	0	0	0	0	0
DERMATITIS AND ECZEMA					
DERMATITIS ALLERGIC	0	0	0	0	0
ERYTHEMAS					
ERYTHEMA	0	0	0	0	0
PRURITUS NEC					
PRURITUS	0	0	0	0	0
RASHES, ERUPTIONS AND EXANTHEMS NEC					
RASH	0	0	0	0	0

RASH PRURITIC	0	0	0	0	0
SKIN APPENDAGE CONDITIONS					
APOCRINE AND ECCRINE GLAND DISORDERS					
COLD SWEAT	0	0	0	0	0
HYPERHIDROSIS	0	0	0	0	0
NIGHT SWEATS	0	0	0	0	0
**VASCULAR DISORDERS**					
DECREASED AND NONSPECIFIC BLOOD PRESSURE DISORDERS AND SHOCK					
BLOOD PRESSURE DISORDERS NEC					
BLOOD PRESSURE FLUCTUATION	0	0	0	0	0
VASCULAR HYPOTENSIVE DISORDERS					
HYPOTENSION	0	0	0	0	0
EMBOLISM AND THROMBOSIS					
NON-SITE SPECIFIC EMBOLISM AND THROMBOSIS					
EMBOLISM	0	0	0	0	0
VASCULAR DISORDERS NEC					
NON-SITE SPECIFIC VASCULAR DISORDERS NEC					
VASCULAR PAIN	0	0	0	0	0
PERIPHERAL VASCULAR DISORDERS NEC					
HOT FLUSH	0	0	0	0	0
VASCULAR HAEMORRHAGIC DISORDERS					
HAEMORRHAGES NEC					
HAEMORRHAGE	0	0	0	0	0
**TOTAL ADR EVENTS**	***5***	***2***	***0***	***3***	***0***

SUPPLEMENTARY TABLE 29. COVID-19 vaccine ADR listings for events reported by pregnant females with no dose identification information in those reporting any vaccination dose

	Individuals with any vaccination dose: ADR Counts
**MEDDRA REACTION TERM (SOC, *HLGT*, *HLT*, PT)**	Unknown dose
(freetext)	0
**BLOOD AND LYMPHATIC SYSTEM DISORDERS**	
SPLEEN, LYMPHATIC AND RETICULOENDOTHELIAL SYSTEM DISORDERS	
LYMPHATIC SYSTEM DISORDERS NEC	
LYMPH NODE PAIN	1
LYMPHADENITIS	0
LYMPHADENOPATHY	0
**CARDIAC DISORDERS**	
CARDIAC ARRHYTHMIAS	
RATE AND RHYTHM DISORDERS NEC	
TACHYCARDIA	0
CARDIAC DISORDERS, SIGNS AND SYMPTOMS NEC	
CARDIAC SIGNS AND SYMPTOMS NEC	
PALPITATIONS	0
**EAR AND LABYRINTH DISORDERS**	
AURAL DISORDERS NEC	
EAR DISORDERS NEC	
EAR PAIN	0
INNER EAR AND VIIITH CRANIAL NERVE DISORDERS	
INNER EAR SIGNS AND SYMPTOMS	
TINNITUS	0
VERTIGO	0
**EYE DISORDERS**	
EYE DISORDERS NEC	
OCULAR DISORDERS NEC	
EYE PAIN	0
OCULAR SENSORY SYMPTOMS NEC	
OCULAR SENSATION DISORDERS	
ASTHENOPIA	0
PHOTOPHOBIA	0
VISION DISORDERS	
VISUAL DISORDERS NEC	
VISION BLURRED	0
**GASTROINTESTINAL DISORDERS**	
DENTAL AND GINGIVAL CONDITIONS	
DENTAL DISORDERS NEC	
TEETHING	0
GASTROINTESTINAL INFLAMMATORY CONDITIONS	
GASTROINTESTINAL INFLAMMATORY DISORDERS NEC	
GASTROINTESTINAL TRACT IRRITATION	0
GASTROINTESTINAL MOTILITY AND DEFAECATION CONDITIONS	
DIARRHOEA (EXCL INFECTIVE)	
DIARRHOEA	0
GASTROINTESTINAL ATONIC AND HYPOMOTILITY DISORDERS NEC	

CONSTIPATION	0
GASTROINTESTINAL SIGNS AND SYMPTOMS	
GASTROINTESTINAL AND ABDOMINAL PAINS (EXCL ORAL AND THROAT)	
ABDOMINAL PAIN	0
ABDOMINAL PAIN UPPER	0
GASTROINTESTINAL SIGNS AND SYMPTOMS NEC	
ABDOMINAL DISCOMFORT	0
NAUSEA AND VOMITING SYMPTOMS	
NAUSEA	3
VOMITING	1
ORAL SOFT TISSUE CONDITIONS	
ORAL SOFT TISSUE SIGNS AND SYMPTOMS	
HYPOAESTHESIA ORAL	0
PARAESTHESIA ORAL	0
TONGUE CONDITIONS	
TONGUE SIGNS AND SYMPTOMS	
GLOSSODYNIA	0
**GENERAL DISORDERS AND ADMINISTRATION SITE CONDITIONS**	
ADMINISTRATION SITE REACTIONS	
ADMINISTRATION SITE REACTIONS NEC	
PUNCTURE SITE BRUISE	1
PUNCTURE SITE PAIN	0
INJECTION SITE REACTIONS	
INJECTION SITE BRUISING	0
INJECTION SITE ERYTHEMA	0
INJECTION SITE MASS	1
INJECTION SITE PAIN	1
INJECTION SITE REACTION	0
INJECTION SITE URTICARIA	0
INJECTION SITE WARMTH	0
VACCINATION SITE REACTIONS	
VACCINATION SITE PAIN	1
BODY TEMPERATURE CONDITIONS	
FEBRILE DISORDERS	
PYREXIA	6
GENERAL SYSTEM DISORDERS NEC	
ASTHENIC CONDITIONS	
ASTHENIA	1
FATIGUE	17
MALAISE	0
FEELINGS AND SENSATIONS NEC	
CHILLS	1
FEELING ABNORMAL	0
FEELING COLD	0
FEELING HOT	0
FEELING OF BODY TEMPERATURE CHANGE	0
HANGOVER	0
HUNGER	0
THIRST	0
GENERAL SIGNS AND SYMPTOMS NEC	
CRYING	0
ILLNESS	0
INFLUENZA LIKE ILLNESS	1
LOCAL REACTION	0
PERIPHERAL SWELLING	0
SWELLING	0

PAIN AND DISCOMFORT NEC	
AXILLARY PAIN	0
CHEST PAIN	0
PAIN	5
TENDERNESS	0
**INFECTIONS AND INFESTATIONS**	
BACTERIAL INFECTIOUS DISORDERS	
BACTERIAL INFECTIONS NEC	
PERIORBITAL CELLULITIS	0
INFECTIONS - PATHOGEN UNSPECIFIED	
BREAST INFECTIONS	
MASTITIS	0
UPPER RESPIRATORY TRACT INFECTIONS	
LARYNGITIS	0
NASOPHARYNGITIS	0
VIRAL INFECTIOUS DISORDERS	
HERPES VIRAL INFECTIONS	
GENITAL HERPES	0
HERPES ZOSTER	0
ORAL HERPES	0
INFLUENZA VIRAL INFECTIONS	
INFLUENZA	0
VIRAL INFECTIONS NEC	
VIRAL DIARRHOEA	0
**INJURY, POISONING AND PROCEDURAL COMPLICATIONS**	
INJURIES NEC	
SKIN INJURIES NEC	
CONTUSION	0
PROCEDURAL RELATED INJURIES AND COMPLICATIONS NEC	
NON-SITE SPECIFIC PROCEDURAL COMPLICATIONS	
INJECTION RELATED REACTION	0
**INVESTIGATIONS**	
CARDIAC AND VASCULAR INVESTIGATIONS (EXCL ENZYME TESTS)	
HEART RATE AND PULSE INVESTIGATIONS	
HEART RATE	0
HEART RATE INCREASED	0
PHYSICAL EXAMINATION AND ORGAN SYSTEM STATUS TOPICS	
PHYSICAL EXAMINATION PROCEDURES AND ORGAN SYSTEM STATUS	
BODY TEMPERATURE INCREASED	0
WATER, ELECTROLYTE AND MINERAL INVESTIGATIONS	
WATER AND ELECTROLYTE ANALYSES NEC	
VOLUME BLOOD	0
**METABOLISM AND NUTRITION DISORDERS**	
APPETITE AND GENERAL NUTRITIONAL DISORDERS	
APPETITE DISORDERS	
DECREASED APPETITE	0
ELECTROLYTE AND FLUID BALANCE CONDITIONS	
TOTAL FLUID VOLUME DECREASED	
DEHYDRATION	0
**MUSCULOSKELETAL AND CONNECTIVE TISSUE DISORDERS**	
BONE DISORDERS (EXCL CONGENITAL AND FRACTURES)	
BONE RELATED SIGNS AND SYMPTOMS	
PAIN IN JAW	0
JOINT DISORDERS	
JOINT RELATED SIGNS AND SYMPTOMS	
ARTHRALGIA	1

MUSCLE DISORDERS	
MUSCLE PAINS	
MYALGIA	2
MUSCLE RELATED SIGNS AND SYMPTOMS NEC	
MUSCLE FATIGUE	0
MUSCLE SPASMS	0
MUSCLE TONE ABNORMALITIES	
TRISMUS	0
MUSCULOSKELETAL AND CONNECTIVE TISSUE DISORDERS NEC	
MUSCULOSKELETAL AND CONNECTIVE TISSUE CONDITIONS NEC	
MUSCULOSKELETAL STIFFNESS	0
BACK PAIN	0
LIMB DISCOMFORT	0
NECK PAIN	0
PAIN IN EXTREMITY	10
**NERVOUS SYSTEM DISORDERS**	
CRANIAL NERVE DISORDERS (EXCL NEOPLASMS)	
FACIAL CRANIAL NERVE DISORDERS	
BELL'S PALSY	0
OLFACTORY NERVE DISORDERS	
ANOSMIA	0
PAROSMIA	0
HEADACHES	
HEADACHES NEC	
CLUSTER HEADACHE	0
HEADACHE	14
SINUS HEADACHE	0
TENSION HEADACHE	0
MIGRAINE HEADACHES	
MIGRAINE WITH AURA	0
TYPICAL AURA WITHOUT HEADACHE	0
MENTAL IMPAIRMENT DISORDERS	
MENTAL IMPAIRMENT (EXCL DEMENTIA AND MEMORY LOSS)	
DISTURBANCE IN ATTENTION	0
MOVEMENT DISORDERS (INCL PARKINSONISM)	
DYSKINESIAS AND MOVEMENT DISORDERS NEC	
CLUMSINESS	0
TREMOR (EXCL CONGENITAL)	
TREMOR	0
NEUROLOGICAL DISORDERS NEC	
DISTURBANCES IN CONSCIOUSNESS NEC	
LETHARGY	0
SOMNOLENCE	2
SYNCOPE	0
NEUROLOGICAL SIGNS AND SYMPTOMS NEC	
AGITATION NEONATAL	1
BRAIN FOG	0
DIZZINESS	1
DIZZINESS EXERTIONAL	0
PRESYNCOPE	0
PARAESTHESIAS AND DYSAESTHESIAS	
PARAESTHESIA	0
SENSORY ABNORMALITIES NEC	

DYSGEUSIA	0
NEURALGIA	0
TASTE DISORDER	0
SEIZURES (INCL SUBTYPES)	
SEIZURES AND SEIZURE DISORDERS NEC	
SEIZURE	0
**PREGNANCY, PUERPERIUM AND PERINATAL CONDITIONS**	
ABORTIONS AND STILLBIRTH	
ABORTIONS SPONTANEOUS	
ABORTION SPONTANEOUS	0
STILLBIRTH AND FOETAL DEATH	
FOETAL DEATH	0
FOETAL COMPLICATIONS	
FOETAL COMPLICATIONS NEC	
FOETAL DISORDER	0
FOETAL HYPOKINESIA	0
FOETAL GROWTH COMPLICATIONS	
FOETAL MACROSOMIA	0
MATERNAL COMPLICATIONS OF PREGNANCY	
MATERNAL COMPLICATIONS OF PREGNANCY NEC	
MORNING SICKNESS	1
PLACENTAL, AMNIOTIC AND CAVITY DISORDERS (EXCL HAEMORRHAGES)	
PLACENTAL ABNORMALITIES (EXCL NEOPLASMS)	
PLACENTAL INFARCTION	0
PREGNANCY, LABOUR, DELIVERY AND POSTPARTUM CONDITIONS	
NORMAL PREGNANCY, LABOUR AND DELIVERY	
PREGNANCY	1
UTERINE CONTRACTIONS DURING PREGNANCY	0
**PSYCHIATRIC DISORDERS**	
ANXIETY DISORDERS AND SYMPTOMS	
ANXIETY SYMPTOMS	
AGITATION	0
ANXIETY	2
COGNITIVE AND ATTENTION DISORDERS AND DISTURBANCES	
COGNITIVE AND ATTENTION DISORDERS AND DISTURBANCES NEC	
MENTAL FATIGUE	0
DELIRIA (INCL CONFUSION)	
CONFUSION AND DISORIENTATION	
CONFUSIONAL STATE	0
DEPRESSED MOOD DISORDERS AND DISTURBANCES	
MOOD ALTERATIONS WITH DEPRESSIVE SYMPTOMS	
DEPRESSED MOOD	0
DISTURBANCES IN THINKING AND PERCEPTION	
HALLUCINATIONS (EXCL SLEEP-RELATED)	
HALLUCINATION	0
MOOD DISORDERS AND DISTURBANCES NEC	
EMOTIONAL AND MOOD DISTURBANCES NEC	
EMOTIONAL DISORDER	0
SLEEP DISORDERS AND DISTURBANCES	
DISTURBANCES IN INITIATING AND MAINTAINING SLEEP	
INITIAL INSOMNIA	0
INSOMNIA	0
**REPRODUCTIVE SYSTEM AND BREAST DISORDERS**	
MENSTRUAL CYCLE AND UTERINE BLEEDING DISORDERS	

MENSTRUATION AND UTERINE BLEEDING NEC	
MENSTRUAL DISORDER	0
MENSTRUATION WITH DECREASED BLEEDING	
MENSTRUATION DELAYED	1
OLIGOMENORRHOEA	1
MENSTRUATION WITH INCREASED BLEEDING	
HEAVY MENSTRUAL BLEEDING	1
VULVOVAGINAL DISORDERS (EXCL INFECTIONS AND INFLAMMATIONS)	
VULVOVAGINAL DISORDERS NEC	
VAGINAL HAEMORRHAGE	0
**RESPIRATORY, THORACIC AND MEDIASTINAL DISORDERS**	
RESPIRATORY DISORDERS NEC	
BREATHING ABNORMALITIES	
DYSPNOEA	1
COUGHING AND ASSOCIATED SYMPTOMS	
COUGH	0
RESPIRATORY TRACT SIGNS AND SYMPTOMS	
UPPER RESPIRATORY TRACT SIGNS AND SYMPTOMS	
DRY THROAT	0
OROPHARYNGEAL PAIN	1
PARANASAL SINUS DISCOMFORT	0
RHINORRHOEA	0
SINUS PAIN	0
SNEEZING	0
UPPER RESPIRATORY TRACT DISORDERS (EXCL INFECTIONS)	
NASAL CONGESTION AND INFLAMMATIONS	
NASAL CONGESTION	0
**SKIN AND SUBCUTANEOUS TISSUE DISORDERS**	
ANGIOEDEMA AND URTICARIA	
URTICARIAS	
URTICARIA	0
CORNIFICATION AND DYSTROPHIC SKIN DISORDERS	
SKIN DYSTROPHIES	
HYPERTROPHIC SCAR	0
EPIDERMAL AND DERMAL CONDITIONS	
DERMAL AND EPIDERMAL CONDITIONS NEC	
SKIN WARM	0
DERMATITIS AND ECZEMA	
DERMATITIS ALLERGIC	0
ERYTHEMAS	
ERYTHEMA	1
PRURITUS NEC	
PRURITUS	1
RASHES, ERUPTIONS AND EXANTHEMS NEC	
RASH	0
RASH PRURITIC	0
SKIN APPENDAGE CONDITIONS	
APOCRINE AND ECCRINE GLAND DISORDERS	
COLD SWEAT	
HYPERHIDROSIS	0
NIGHT SWEATS	0
**VASCULAR DISORDERS**	
DECREASED AND NONSPECIFIC BLOOD PRESSURE DISORDERS AND SHOCK	

BLOOD PRESSURE DISORDERS NEC	
BLOOD PRESSURE FLUCTUATION	0
VASCULAR HYPOTENSIVE DISORDERS	
HYPOTENSION	0
EMBOLISM AND THROMBOSIS	
NON-SITE SPECIFIC EMBOLISM AND THROMBOSIS	
EMBOLISM	0
VASCULAR DISORDERS NEC	
NON-SITE SPECIFIC VASCULAR DISORDERS NEC	
VASCULAR PAIN	0
PERIPHERAL VASCULAR DISORDERS NEC	
HOT FLUSH	0
VASCULAR HAEMORRHAGIC DISORDERS	
HAEMORRHAGES NEC	
HAEMORRHAGE	0
**TOTAL ADR EVENTS**	***82***

SUPPLEMENTARY TABLE 30. ADR listing for events reported by breastfeeding females, in those reporting any vaccination dose

	Individuals with any vaccination dose: ADR Counts					
**MEDDRA REACTION TERM (SOC, *HLGT*, *HLT*, PT)**	All doses	1st dose	2nd dose	3rd dose	Other doses	Unknown
(freetext)	1	1	0	0	0	0
**BLOOD AND LYMPHATIC SYSTEM DISORDERS**						
LYMPHATIC SYSTEM DISORDERS NEC						
LYMPH NODE PAIN	2	0	1	1	0	0
LYMPHADENOPATHY	5	1	3	0	0	1
**CARDIAC DISORDERS**						
CARDIAC DISORDERS, SIGNS AND SYMPTOMS NEC						
CARDIAC SIGNS AND SYMPTOMS NEC						
PALPITATIONS	6	2	2	0	0	2
**EAR AND LABYRINTH DISORDERS**						
AURAL DISORDERS NEC						
EAR DISORDERS NEC						
EAR PAIN	1	1	0	0	0	0
INNER EAR AND VIIITH CRANIAL NERVE DISORDERS						
INNER EAR SIGNS AND SYMPTOMS						
TINNITUS	2	0	1	1	0	0
**EYE DISORDERS**						
EYE DISORDERS NEC						
LACRIMATION DISORDERS						
DRY EYE	1	1	0	0	0	0
OCULAR DISORDERS NEC						
EYE PAIN	1	1	0	0	0	0
OCULAR INFECTIONS, IRRITATIONS AND INFLAMMATIONS						
OCULAR INFECTIONS, INFLAMMATIONS AND ASSOCIATED MANIFESTATIONS						
EYE IRRITATION	1	1	0	0	0	0
OCULAR SENSORY SYMPTOMS NEC						
OCULAR SENSATION DISORDERS						
PHOTOPHOBIA	2	2	0	0	0	0
VISION DISORDERS						
VISUAL DISORDERS NEC						
VISION BLURRED	2	2	0	0	0	0
**GASTROINTESTINAL DISORDERS**						
GASTROINTESTINAL INFLAMMATORY CONDITIONS						
GASTROINTESTINAL INFLAMMATORY DISORDERS NEC						
GASTROINTESTINAL TRACT IRRITATION	1	0	0	1	0	0
GASTROINTESTINAL MOTILITY AND DEFAECATION CONDITIONS						
DIARRHOEA (EXCL INFECTIVE)						
DIARRHOEA	11	7	2	2	0	0
GASTROINTESTINAL SIGNS AND SYMPTOMS						
FLATULENCE, BLOATING AND DISTENSION						
ABDOMINAL DISTENSION	1	1	0	0	0	0
FLATULENCE	1	1	0	0	0	0
GASTROINTESTINAL AND ABDOMINAL PAINS (EXCL ORAL AND THROAT)						
ABDOMINAL PAIN	5	5	0	0	0	0
ABDOMINAL PAIN UPPER	3	2	1	0	0	0

NAUSEA AND VOMITING SYMPTOMS						
NAUSEA	39	22	8	5	0	4
RETCHING	1	1	0	0	0	0
VOMITING	6	4	2	0	0	0
ORAL SOFT TISSUE CONDITIONS						
ORAL SOFT TISSUE SIGNS AND SYMPTOMS						
PARAESTHESIA ORAL	1	1	0	0	0	0
LIP SWELLING	1	0	1	0	0	0
**GENERAL DISORDERS AND ADMINISTRATION SITE CONDITIONS**						
ADMINISTRATION SITE REACTIONS						
ADMINISTRATION SITE REACTIONS NEC						
PUNCTURE SITE BRUISE	1	0	0	1	0	0
INJECTION SITE REACTIONS						
INJECTION SITE ERYTHEMA	1	1	0	0	0	0
INJECTION SITE MASS	1	0	0	0	0	1
INJECTION SITE PAIN	23	16	3	1	0	3
INJECTION SITE WARMTH	1	1	0	0	0	0
VACCINATION SITE REACTIONS						
VACCINATION SITE PAIN	1	0	1	0	0	0
BODY TEMPERATURE CONDITIONS						
FEBRILE DISORDERS						
PYREXIA	70	44	16	5	0	5
GENERAL SYSTEM DISORDERS NEC						
ASTHENIC CONDITIONS						
FATIGUE	113	60	35	10	0	8
MALAISE	6	3	3	0	0	0
FEELINGS AND SENSATIONS NEC						
CHILLS	42	27	6	6	0	3
FEELING ABNORMAL	1	0	0	1	0	0
FEELING COLD	9	7	2	0	0	0
FEELING HOT	1	1	0	0	0	0
HANGOVER	1	0	0	1	0	0
GENERAL SIGNS AND SYMPTOMS NEC						
INFLUENZA LIKE ILLNESS	8	4	1	2	0	1
LOCAL REACTION	1	0	1	0	0	0
PERIPHERAL SWELLING	4	3	1	0	0	0
SWELLING	1	1	0	0	0	0
PAIN AND DISCOMFORT NEC						
AXILLARY PAIN	4	1	3	0	0	0
CHEST DISCOMFORT	1	1	0	0	0	0
PAIN	29	16	6	5	0	2
TENDERNESS	4	2	2	0	0	0
**INFECTIONS AND INFESTATIONS**						
INFECTIONS - PATHOGEN UNSPECIFIED						
BREAST INFECTIONS						
MASTITIS	1	0	0	1	0	0
SKIN STRUCTURES AND SOFT TISSUE INFECTIONS						
INJECTION SITE PUSTULE	1	0	1	0	0	0
UPPER RESPIRATORY TRACT INFECTIONS						
LARYNGITIS	1	0	1	0	0	0
NASOPHARYNGITIS	3	2	1	0	0	0
VIRAL INFECTIOUS DISORDERS						
HERPES VIRAL INFECTIONS						

ORAL HERPES	1	0	1	0	0	0
INFLUENZA VIRAL INFECTIONS						
INFLUENZA	11	6	4	1	0	0
**INJURY, POISONING AND PROCEDURAL COMPLICATIONS**						
INJURIES NEC						
SKIN INJURIES NEC						
CONTUSION	2	2	0	0	0	0
**INVESTIGATIONS**						
CARDIAC AND VASCULAR INVESTIGATIONS (EXCL ENZYME TESTS)						
HEART RATE AND PULSE INVESTIGATIONS						
HEART RATE	1	1	0	0	0	0
PHYSICAL EXAMINATION AND ORGAN SYSTEM STATUS TOPICS						
PHYSICAL EXAMINATION PROCEDURES AND ORGAN SYSTEM STATUS						
BODY TEMPERATURE	3	3	0	0	0	0
BODY TEMPERATURE INCREASED	2	1	0	1	0	0
WATER, ELECTROLYTE AND MINERAL INVESTIGATIONS						
WATER AND ELECTROLYTE ANALYSES NEC						
VOLUME BLOOD	1	0	1	0	0	0
**METABOLISM AND NUTRITION DISORDERS**						
APPETITE AND GENERAL NUTRITIONAL DISORDERS						
APPETITE DISORDERS						
DECREASED APPETITE	4	3	1	0	0	0
**MUSCULOSKELETAL AND CONNECTIVE TISSUE DISORDERS**						
BONE DISORDERS (EXCL CONGENITAL AND FRACTURES)						
BONE RELATED SIGNS AND SYMPTOMS						
BONE PAIN	1	1	0	0	0	0
PAIN IN JAW	1	0	1	0	0	0
JOINT DISORDERS						
JOINT RELATED SIGNS AND SYMPTOMS						
ARTHRALGIA	17	9	5	1	0	2
JOINT STIFFNESS	1	1	0	0	0	0
MUSCLE DISORDERS						
MUSCLE PAINS						
MYALGIA	51	35	9	5	0	2
MUSCLE RELATED SIGNS AND SYMPTOMS NEC						
MUSCLE FATIGUE	1	0	0	1	0	0
MUSCLE SPASMS	1	1	0	0	0	0
MUSCULOSKELETAL AND CONNECTIVE TISSUE DISORDERS NEC						
MUSCULOSKELETAL AND CONNECTIVE TISSUE CONDITIONS NEC						
MUSCULOSKELETAL STIFFNESS	2	0	0	2	0	0
BACK PAIN	3	2	0	1	0	0
LIMB DISCOMFORT	25	19	6	0	0	0
PAIN IN EXTREMITY	134	100	23	8	0	3
**NERVOUS SYSTEM DISORDERS**						
CRANIAL NERVE DISORDERS (EXCL NEOPLASMS)						
OLFACTORY NERVE DISORDERS						
ANOSMIA	1	1	0	0	0	0
PAROSMIA	1	0	1	0	0	0
HEADACHES						
HEADACHES NEC						
HEADACHE	101	64	16	13	0	8
SINUS HEADACHE	3	1	2	0	0	0
TENSION HEADACHE	5	5	0	0	0	0

MIGRAINE HEADACHES						
MIGRAINE	2	1	0	0	0	1
TYPICAL AURA WITHOUT HEADACHE	1	0	1	0	0	0
MOVEMENT DISORDERS (INCL PARKINSONISM)						
TREMOR (EXCL CONGENITAL)						
TREMOR	2	1	0	0	0	1
NEUROLOGICAL DISORDERS NEC						
DISTURBANCES IN CONSCIOUSNESS NEC						
LETHARGY	5	1	4	0	0	0
SOMNOLENCE	5	3	2	0	0	0
NEUROLOGICAL SIGNS AND SYMPTOMS NEC						
BRAIN FOG	1	1	0	0	0	0
DIZZINESS	18	6	7	2	0	3
INFANT IRRITABILITY	1	0	1	0	0	0
NEUROLOGICAL SYMPTOM	1	1	0	0	0	0
PRESYNCOPE	1	1	0	0	0	0
PARAESTHESIAS AND DYSAESTHESIAS						
HYPOAESTHESIA	1	1	0	0	0	0
PARAESTHESIA	2	2	0	0	0	0
SENSORY ABNORMALITIES NEC						
AGEUSIA	1	1	0	0	0	0
DYSGEUSIA	2	2	0	0	0	0
SEIZURES (INCL SUBTYPES)						
SEIZURES AND SEIZURE DISORDERS NEC						
SEIZURE	2	2	0	0	0	0
**PREGNANCY, PUERPERIUM AND PERINATAL CONDITIONS**						
PLACENTAL, AMNIOTIC AND CAVITY DISORDERS (EXCL HAEMORRHAGES)						
PLACENTAL ABNORMALITIES (EXCL NEOPLASMS)						
PLACENTAL INFARCTION	1	1	0	0	0	0
**PSYCHIATRIC DISORDERS**						
ANXIETY DISORDERS AND SYMPTOMS						
ANXIETY SYMPTOMS						
AGITATION	1	0	1	0	0	0
ANXIETY	2	0	0	0	0	2
DELIRIA (INCL CONFUSION)						
CONFUSION AND DISORIENTATION						
CONFUSIONAL STATE	2	2	0	0	0	0
DEPRESSED MOOD DISORDERS AND DISTURBANCES						
MOOD ALTERATIONS WITH DEPRESSIVE SYMPTOMS						
DEPRESSED MOOD	1	1	0	0	0	0
MOOD DISORDERS AND DISTURBANCES NEC						
EMOTIONAL AND MOOD DISTURBANCES NEC						
EMOTIONAL DISORDER	1	0	1	0	0	0
IRRITABILITY	1	1	0	0	0	0
MOOD ALTERED	1	0	0	1	0	0
SLEEP DISORDERS AND DISTURBANCES						
DISTURBANCES IN INITIATING AND MAINTAINING SLEEP						
INSOMNIA	2	2	0	0	0	0
**REPRODUCTIVE SYSTEM AND BREAST DISORDERS**						
MENSTRUAL CYCLE AND UTERINE BLEEDING DISORDERS						
MENSTRUATION AND UTERINE BLEEDING NEC						
MENSTRUATION IRREGULAR	1	1	0	0	0	0
MENSTRUATION WITH DECREASED BLEEDING						

HYPOMENORRHOEA	1	0	1	0	0	0
MENSTRUATION DELAYED	1	0	1	0	0	0
OLIGOMENORRHOEA	1	0	0	0	0	1
MENSTRUATION WITH INCREASED BLEEDING						
HEAVY MENSTRUAL BLEEDING	1	1	0	0	0	0
VULVOVAGINAL DISORDERS (EXCL INFECTIONS AND INFLAMMATIONS)						
VULVOVAGINAL DISORDERS NEC						
VAGINAL HAEMORRHAGE	1	1	0	0	0	0
**RESPIRATORY, THORACIC AND MEDIASTINAL DISORDERS**						
RESPIRATORY DISORDERS NEC						
BREATHING ABNORMALITIES						
DYSPNOEA	4	3	1	0	0	0
COUGHING AND ASSOCIATED SYMPTOMS						
COUGH	2	2	0	0	0	0
RESPIRATORY TRACT SIGNS AND SYMPTOMS						
UPPER RESPIRATORY TRACT SIGNS AND SYMPTOMS						
OROPHARYNGEAL PAIN	9	7	2	0	0	0
PARANASAL SINUS DISCOMFORT	2	2	0	0	0	0
RHINORRHOEA	2	1	0	1	0	0
**SKIN AND SUBCUTANEOUS TISSUE DISORDERS**						
EPIDERMAL AND DERMAL CONDITIONS						
DERMAL AND EPIDERMAL CONDITIONS NEC						
SENSITIVE SKIN	1	1	0	0	0	0
DERMATITIS AND ECZEMA						
DERMATITIS ALLERGIC	1	1	0	0	0	0
ERYTHEMAS						
ERYTHEMA	3	1	0	0	0	2
PRURITUS NEC						
PRURITUS	4	4	0	0	0	0
RASHES, ERUPTIONS AND EXANTHEMS NEC						
RASH	4	2	1	1	0	0
SKIN APPENDAGE CONDITIONS						
APOCRINE AND ECCRINE GLAND DISORDERS						
COLD SWEAT	1	0	1	0	0	0
HYPERHIDROSIS	4	3	0	0	0	1
NIGHT SWEATS	2	1	1	0	0	0
**VASCULAR DISORDERS**						
EMBOLISM AND THROMBOSIS						
NON-SITE SPECIFIC EMBOLISM AND THROMBOSIS						
EMBOLISM	1	1	0	0	0	0
VASCULAR DISORDERS NEC						
PERIPHERAL VASCULAR DISORDERS NEC						
HOT FLUSH	1	1	0	0	0	0
**TOTAL ADR EVENTS**	***900***	***564***	***199***	***81***	***0***	***56***

SUPPLEMENTARY TABLE 31. Pfizer BioNTech COVID-19 vaccine: ADR listing for events reported by breastfeeding females, in those reporting any vaccination dose

	Individuals with any vaccination dose: ADR Counts				
**MEDDRA REACTION TERM (SOC, *HLGT*, *HLT*, PT)**	All doses	1st dose	2nd dose	3rd dose	Other doses
(freetext)	1	1	0	0	0
**BLOOD AND LYMPHATIC SYSTEM DISORDERS**					
LYMPHATIC SYSTEM DISORDERS NEC					
LYMPH NODE PAIN	2	0	1	1	0
LYMPHADENOPATHY	2	1	1	0	0
**CARDIAC DISORDERS**					
CARDIAC DISORDERS, SIGNS AND SYMPTOMS NEC					
CARDIAC SIGNS AND SYMPTOMS NEC					
PALPITATIONS	3	1	2	0	0
**EAR AND LABYRINTH DISORDERS**					
AURAL DISORDERS NEC					
EAR DISORDERS NEC					
EAR PAIN	0	0	0	0	0
INNER EAR AND VIIITH CRANIAL NERVE DISORDERS					
INNER EAR SIGNS AND SYMPTOMS					
TINNITUS	1	0	1	0	0
**EYE DISORDERS**					
EYE DISORDERS NEC					
LACRIMATION DISORDERS					
DRY EYE	0	0	0	0	0
OCULAR DISORDERS NEC					
EYE PAIN	0	0	0	0	0
OCULAR INFECTIONS, IRRITATIONS AND INFLAMMATIONS					
OCULAR INFECTIONS, INFLAMMATIONS AND ASSOCIATED MANIFESTATIONS					
EYE IRRITATION	0	0	0	0	0
OCULAR SENSORY SYMPTOMS NEC					
OCULAR SENSATION DISORDERS					
PHOTOPHOBIA	1	1	0	0	0
VISION DISORDERS					
VISUAL DISORDERS NEC					
VISION BLURRED	0	0	0	0	0
**GASTROINTESTINAL DISORDERS**					
GASTROINTESTINAL INFLAMMATORY CONDITIONS					
GASTROINTESTINAL INFLAMMATORY DISORDERS NEC					
GASTROINTESTINAL TRACT IRRITATION	1	0	0	1	0
GASTROINTESTINAL MOTILITY AND DEFAECATION CONDITIONS					
DIARRHOEA (EXCL INFECTIVE)					
DIARRHOEA	8	4	2	2	0
GASTROINTESTINAL SIGNS AND SYMPTOMS					
FLATULENCE, BLOATING AND DISTENSION					
ABDOMINAL DISTENSION	0	0	0	0	0
FLATULENCE	0	0	0	0	0
GASTROINTESTINAL AND ABDOMINAL PAINS (EXCL ORAL AND THROAT)					
ABDOMINAL PAIN	0	0	0	0	0
ABDOMINAL PAIN UPPER	1	1	0	0	0

NAUSEA AND VOMITING SYMPTOMS					
NAUSEA	11	5	6	0	0
RETCHING	0	0	0	0	0
VOMITING	2	1	1	0	0
ORAL SOFT TISSUE CONDITIONS					
ORAL SOFT TISSUE SIGNS AND SYMPTOMS					
PARAESTHESIA ORAL	0	0	0	0	0
LIP SWELLING	0	0	0	0	0
**GENERAL DISORDERS AND ADMINISTRATION SITE CONDITIONS**					
ADMINISTRATION SITE REACTIONS					
ADMINISTRATION SITE REACTIONS NEC					
PUNCTURE SITE BRUISE	0	0	0	0	0
INJECTION SITE REACTIONS					
INJECTION SITE ERYTHEMA	1	1	0	0	0
INJECTION SITE MASS	0	0	0	0	0
INJECTION SITE PAIN	15	12	3	0	0
INJECTION SITE WARMTH	1	1	0	0	0
VACCINATION SITE REACTIONS					
VACCINATION SITE PAIN	0	0	0	0	0
BODY TEMPERATURE CONDITIONS					
FEBRILE DISORDERS					
PYREXIA	14	5	8	1	0
GENERAL SYSTEM DISORDERS NEC					
ASTHENIC CONDITIONS					
FATIGUE	47	26	18	3	0
MALAISE	2	0	2	0	0
FEELINGS AND SENSATIONS NEC					
CHILLS	6	2	3	1	0
FEELING ABNORMAL	1	0	0	1	0
FEELING COLD	1	0	1	0	0
FEELING HOT	0	0	0	0	0
HANGOVER	1	0	0	1	0
GENERAL SIGNS AND SYMPTOMS NEC					
INFLUENZA LIKE ILLNESS	1	0	1	0	0
LOCAL REACTION	1	0	1	0	0
PERIPHERAL SWELLING	2	1	1	0	0
SWELLING	0	0	0	0	0
PAIN AND DISCOMFORT NEC					
AXILLARY PAIN	4	1	3	0	0
CHEST DISCOMFORT	0	0	0	0	0
PAIN	17	10	4	3	0
TENDERNESS	2	0	2	0	0
**INFECTIONS AND INFESTATIONS**					
INFECTIONS - PATHOGEN UNSPECIFIED					
BREAST INFECTIONS					
MASTITIS	0	0	0	0	0
SKIN STRUCTURES AND SOFT TISSUE INFECTIONS					
INJECTION SITE PUSTULE	0	0	0	0	0
UPPER RESPIRATORY TRACT INFECTIONS					
LARYNGITIS	1	0	1	0	0
NASOPHARYNGITIS	1	0	1	0	0
VIRAL INFECTIOUS DISORDERS					
HERPES VIRAL INFECTIONS					

ORAL HERPES	1	0	1	0	0
INFLUENZA VIRAL INFECTIONS					
INFLUENZA	3	0	2	1	0
**INJURY, POISONING AND PROCEDURAL COMPLICATIONS**					
INJURIES NEC					
SKIN INJURIES NEC					
CONTUSION	1	1	0	0	0
**INVESTIGATIONS**					
CARDIAC AND VASCULAR INVESTIGATIONS (EXCL ENZYME TESTS)					
HEART RATE AND PULSE INVESTIGATIONS					
HEART RATE	0	0	0	0	0
PHYSICAL EXAMINATION AND ORGAN SYSTEM STATUS TOPICS					
PHYSICAL EXAMINATION PROCEDURES AND ORGAN SYSTEM STATUS					
BODY TEMPERATURE	0	0	0	0	0
BODY TEMPERATURE INCREASED	0	0	0	0	0
WATER, ELECTROLYTE AND MINERAL INVESTIGATIONS					
WATER AND ELECTROLYTE ANALYSES NEC					
VOLUME BLOOD	1	0	1	0	0
**METABOLISM AND NUTRITION DISORDERS**					
APPETITE AND GENERAL NUTRITIONAL DISORDERS					
APPETITE DISORDERS					
DECREASED APPETITE	0	0	0	0	0
**MUSCULOSKELETAL AND CONNECTIVE TISSUE DISORDERS**					
BONE DISORDERS (EXCL CONGENITAL AND FRACTURES)					
BONE RELATED SIGNS AND SYMPTOMS					
BONE PAIN	0	0	0	0	0
PAIN IN JAW	0	0	0	0	0
JOINT DISORDERS					
JOINT RELATED SIGNS AND SYMPTOMS					
ARTHRALGIA	6	3	3	0	0
JOINT STIFFNESS	0	0	0	0	0
MUSCLE DISORDERS					
MUSCLE PAINS					
MYALGIA	10	5	4	1	0
MUSCLE RELATED SIGNS AND SYMPTOMS NEC					
MUSCLE FATIGUE	0	0	0	0	0
MUSCLE SPASMS	0	0	0	0	0
MUSCULOSKELETAL AND CONNECTIVE TISSUE DISORDERS NEC					
MUSCULOSKELETAL AND CONNECTIVE TISSUE CONDITIONS NEC					
MUSCULOSKELETAL STIFFNESS	2	0	0	2	0
BACK PAIN	1	0	0	1	0
LIMB DISCOMFORT	19	14	5	0	0
PAIN IN EXTREMITY	89	69	14	6	0
**NERVOUS SYSTEM DISORDERS**					
CRANIAL NERVE DISORDERS (EXCL NEOPLASMS)					
OLFACTORY NERVE DISORDERS					
ANOSMIA	1	1	0	0	0
PAROSMIA	0	0	0	0	0
HEADACHES					
HEADACHES NEC					
HEADACHE	40	24	9	7	0
SINUS HEADACHE	2	0	2	0	0
TENSION HEADACHE	1	1	0	0	0

MIGRAINE HEADACHES					
MIGRAINE	0	0	0	0	0
TYPICAL AURA WITHOUT HEADACHE	1	0	1	0	0
MOVEMENT DISORDERS (INCL PARKINSONISM)					
TREMOR (EXCL CONGENITAL)					
TREMOR	0	0	0	0	0
NEUROLOGICAL DISORDERS NEC					
DISTURBANCES IN CONSCIOUSNESS NEC					
LETHARGY	3	0	3	0	0
SOMNOLENCE	2	2	0	0	0
NEUROLOGICAL SIGNS AND SYMPTOMS NEC					
BRAIN FOG	0	0	0	0	0
DIZZINESS	8	2	5	1	0
INFANT IRRITABILITY	0	0	0	0	0
NEUROLOGICAL SYMPTOM	0	0	0	0	0
PRESYNCOPE	0	0	0	0	0
PARAESTHESIAS AND DYSAESTHESIAS					
HYPOAESTHESIA	1	1	0	0	0
PARAESTHESIA	1	1	0	0	0
SENSORY ABNORMALITIES NEC					
AGEUSIA	0	0	0	0	0
DYSGEUSIA	0	0	0	0	0
SEIZURES (INCL SUBTYPES)					
SEIZURES AND SEIZURE DISORDERS NEC					
SEIZURE	0	0	0	0	0
**PREGNANCY, PUERPERIUM AND PERINATAL CONDITIONS**					
PLACENTAL, AMNIOTIC AND CAVITY DISORDERS (EXCL HAEMORRHAGES)					
PLACENTAL ABNORMALITIES (EXCL NEOPLASMS)					
PLACENTAL INFARCTION	0	0	0	0	0
**PSYCHIATRIC DISORDERS**					
ANXIETY DISORDERS AND SYMPTOMS					
ANXIETY SYMPTOMS					
AGITATION	1	0	1	0	0
ANXIETY	0	0	0	0	0
DELIRIA (INCL CONFUSION)					
CONFUSION AND DISORIENTATION					
CONFUSIONAL STATE	1	1	0	0	0
DEPRESSED MOOD DISORDERS AND DISTURBANCES					
MOOD ALTERATIONS WITH DEPRESSIVE SYMPTOMS					
DEPRESSED MOOD	1	1	0	0	0
MOOD DISORDERS AND DISTURBANCES NEC					
EMOTIONAL AND MOOD DISTURBANCES NEC					
EMOTIONAL DISORDER	1	0	1	0	0
IRRITABILITY	0	0	0	0	0
MOOD ALTERED	0	0	0	0	0
SLEEP DISORDERS AND DISTURBANCES					
DISTURBANCES IN INITIATING AND MAINTAINING SLEEP					
INSOMNIA	0	0	0	0	0
**REPRODUCTIVE SYSTEM AND BREAST DISORDERS**					
MENSTRUAL CYCLE AND UTERINE BLEEDING DISORDERS					
MENSTRUATION AND UTERINE BLEEDING NEC					
MENSTRUATION IRREGULAR	1	1	0	0	0
MENSTRUATION WITH DECREASED BLEEDING					

HYPOMENORRHOEA	1	0	1	0	0
MENSTRUATION DELAYED	0	0	0	0	0
OLIGOMENORRHOEA	0	0	0	0	0
MENSTRUATION WITH INCREASED BLEEDING					
HEAVY MENSTRUAL BLEEDING	0	0	0	0	0
VULVOVAGINAL DISORDERS (EXCL INFECTIONS AND INFLAMMATIONS)					
VULVOVAGINAL DISORDERS NEC					
VAGINAL HAEMORRHAGE	1	1	0	0	0
**RESPIRATORY, THORACIC AND MEDIASTINAL DISORDERS**					
RESPIRATORY DISORDERS NEC					
BREATHING ABNORMALITIES					
DYSPNOEA	2	2	0	0	0
COUGHING AND ASSOCIATED SYMPTOMS					
COUGH	2	2	0	0	0
RESPIRATORY TRACT SIGNS AND SYMPTOMS					
UPPER RESPIRATORY TRACT SIGNS AND SYMPTOMS					
OROPHARYNGEAL PAIN	5	3	2	0	0
PARANASAL SINUS DISCOMFORT	1	1	0	0	0
RHINORRHOEA	1	1	0	0	0
**SKIN AND SUBCUTANEOUS TISSUE DISORDERS**					
EPIDERMAL AND DERMAL CONDITIONS					
DERMAL AND EPIDERMAL CONDITIONS NEC					
SENSITIVE SKIN	0	0	0	0	0
DERMATITIS AND ECZEMA					
DERMATITIS ALLERGIC	0	0	0	0	0
ERYTHEMAS					
ERYTHEMA	0	0	0	0	0
PRURITUS NEC					
PRURITUS	1	1	0	0	0
RASHES, ERUPTIONS AND EXANTHEMS NEC					
RASH	2	1	1	0	0
SKIN APPENDAGE CONDITIONS					
APOCRINE AND ECCRINE GLAND DISORDERS					
COLD SWEAT	0	0	0	0	0
HYPERHIDROSIS	0	0	0	0	0
NIGHT SWEATS	1	0	1	0	0
**VASCULAR DISORDERS**					
EMBOLISM AND THROMBOSIS					
NON-SITE SPECIFIC EMBOLISM AND THROMBOSIS					
EMBOLISM	0	0	0	0	0
VASCULAR DISORDERS NEC					
PERIPHERAL VASCULAR DISORDERS NEC					
HOT FLUSH	0	0	0	0	0
**TOTAL ADR EVENTS**	***366***	***213***	***120***	***33***	***0***

SUPPLEMENTARY TABLE 32. AstraZeneca COVID-19 vaccine: ADR listing for events reported by breastfeeding females, in those reporting any vaccination dose

	Individuals with any vaccination dose: ADR Counts				
**MEDDRA REACTION TERM (SOC, *HLGT*, *HLT*, PT)**	All doses	1st dose	2nd dose	3rd dose	Other doses
(freetext)	0	0	0	0	0
**BLOOD AND LYMPHATIC SYSTEM DISORDERS**					
LYMPHATIC SYSTEM DISORDERS NEC					
LYMPH NODE PAIN	0	0	0	0	0
LYMPHADENOPATHY	1	0	1	0	0
**CARDIAC DISORDERS**					
CARDIAC DISORDERS, SIGNS AND SYMPTOMS NEC					
CARDIAC SIGNS AND SYMPTOMS NEC					
PALPITATIONS	1	1	0	0	0
**EAR AND LABYRINTH DISORDERS**					
AURAL DISORDERS NEC					
EAR DISORDERS NEC					
EAR PAIN	1	1	0	0	0
INNER EAR AND VIIITH CRANIAL NERVE DISORDERS					
INNER EAR SIGNS AND SYMPTOMS					
TINNITUS	0	0	0	0	0
**EYE DISORDERS**					
EYE DISORDERS NEC					
LACRIMATION DISORDERS					
DRY EYE	1	1	0	0	0
OCULAR DISORDERS NEC					
EYE PAIN	1	1	0	0	0
OCULAR INFECTIONS, IRRITATIONS AND INFLAMMATIONS					
OCULAR INFECTIONS, INFLAMMATIONS AND ASSOCIATED MANIFESTATIONS					
EYE IRRITATION	0	0	0	0	0
OCULAR SENSORY SYMPTOMS NEC					
OCULAR SENSATION DISORDERS					
PHOTOPHOBIA	1	1	0	0	0
VISION DISORDERS					
VISUAL DISORDERS NEC					
VISION BLURRED	2	2	0	0	0
**GASTROINTESTINAL DISORDERS**					
GASTROINTESTINAL INFLAMMATORY CONDITIONS					
GASTROINTESTINAL INFLAMMATORY DISORDERS NEC					
GASTROINTESTINAL TRACT IRRITATION	0	0	0	0	0
GASTROINTESTINAL MOTILITY AND DEFAECATION CONDITIONS					
DIARRHOEA (EXCL INFECTIVE)					
DIARRHOEA	3	3	0	0	0
GASTROINTESTINAL SIGNS AND SYMPTOMS					
FLATULENCE, BLOATING AND DISTENSION					
ABDOMINAL DISTENSION	1	1	0	0	0
FLATULENCE	1	1	0	0	0
GASTROINTESTINAL AND ABDOMINAL PAINS (EXCL ORAL AND THROAT)					
ABDOMINAL PAIN	5	5	0	0	0
ABDOMINAL PAIN UPPER	2	1	1	0	0

NAUSEA AND VOMITING SYMPTOMS					
NAUSEA	17	15	2	0	0
RETCHING	1	1	0	0	0
VOMITING	3	3	0	0	0
ORAL SOFT TISSUE CONDITIONS					
ORAL SOFT TISSUE SIGNS AND SYMPTOMS					
PARAESTHESIA ORAL	1	1	0	0	0
LIP SWELLING	0	0	0	0	0
**GENERAL DISORDERS AND ADMINISTRATION SITE CONDITIONS**					
ADMINISTRATION SITE REACTIONS					
ADMINISTRATION SITE REACTIONS NEC					
PUNCTURE SITE BRUISE	0	0	0	0	0
INJECTION SITE REACTIONS					
INJECTION SITE ERYTHEMA	0	0	0	0	0
INJECTION SITE MASS	0	0	0	0	0
INJECTION SITE PAIN	4	4	0	0	0
INJECTION SITE WARMTH	0	0	0	0	0
VACCINATION SITE REACTIONS					
VACCINATION SITE PAIN	1	0	1	0	0
BODY TEMPERATURE CONDITIONS					
FEBRILE DISORDERS					
PYREXIA	41	38	3	0	0
GENERAL SYSTEM DISORDERS NEC					
ASTHENIC CONDITIONS					
FATIGUE	38	29	9	0	0
MALAISE	2	2	0	0	0
FEELINGS AND SENSATIONS NEC					
CHILLS	25	25	0	0	0
FEELING ABNORMAL	0	0	0	0	0
FEELING COLD	7	7	0	0	0
FEELING HOT	1	1	0	0	0
HANGOVER	0	0	0	0	0
GENERAL SIGNS AND SYMPTOMS NEC					
INFLUENZA LIKE ILLNESS	4	4	0	0	0
LOCAL REACTION	0	0	0	0	0
PERIPHERAL SWELLING	1	1	0	0	0
SWELLING	0	0	0	0	0
PAIN AND DISCOMFORT NEC					
AXILLARY PAIN	0	0	0	0	0
CHEST DISCOMFORT	1	1	0	0	0
PAIN	7	6	1	0	0
TENDERNESS	2	2	0	0	0
**INFECTIONS AND INFESTATIONS**					
INFECTIONS - PATHOGEN UNSPECIFIED					
BREAST INFECTIONS					
MASTITIS	0	0	0	0	0
SKIN STRUCTURES AND SOFT TISSUE INFECTIONS					
INJECTION SITE PUSTULE	1	0	1	0	0
UPPER RESPIRATORY TRACT INFECTIONS					
LARYNGITIS	0	0	0	0	0
NASOPHARYNGITIS	2	2	0	0	0
VIRAL INFECTIOUS DISORDERS					
HERPES VIRAL INFECTIONS					

ORAL HERPES	0	0	0	0	0
INFLUENZA VIRAL INFECTIONS					
INFLUENZA	5	5	0	0	0
**INJURY, POISONING AND PROCEDURAL COMPLICATIONS**					
INJURIES NEC					
SKIN INJURIES NEC					
CONTUSION	1	1	0	0	0
**INVESTIGATIONS**					
CARDIAC AND VASCULAR INVESTIGATIONS (EXCL ENZYME TESTS)					
HEART RATE AND PULSE INVESTIGATIONS					
HEART RATE	1	1	0	0	0
PHYSICAL EXAMINATION AND ORGAN SYSTEM STATUS TOPICS					
PHYSICAL EXAMINATION PROCEDURES AND ORGAN SYSTEM STATUS					
BODY TEMPERATURE	3	3	0	0	0
BODY TEMPERATURE INCREASED	1	1	0	0	0
WATER, ELECTROLYTE AND MINERAL INVESTIGATIONS					
WATER AND ELECTROLYTE ANALYSES NEC					
VOLUME BLOOD	0	0	0	0	0
**METABOLISM AND NUTRITION DISORDERS**					
APPETITE AND GENERAL NUTRITIONAL DISORDERS					
APPETITE DISORDERS					
DECREASED APPETITE	4	3	1	0	0
**MUSCULOSKELETAL AND CONNECTIVE TISSUE DISORDERS**					
BONE DISORDERS (EXCL CONGENITAL AND FRACTURES)					
BONE RELATED SIGNS AND SYMPTOMS					
BONE PAIN	1	1	0	0	0
PAIN IN JAW	1	0	1	0	0
JOINT DISORDERS					
JOINT RELATED SIGNS AND SYMPTOMS					
ARTHRALGIA	6	6	0	0	0
JOINT STIFFNESS	1	1	0	0	0
MUSCLE DISORDERS					
MUSCLE PAINS					
MYALGIA	30	28	2	0	0
MUSCLE RELATED SIGNS AND SYMPTOMS NEC					
MUSCLE FATIGUE	0	0	0	0	0
MUSCLE SPASMS	1	1	0	0	0
MUSCULOSKELETAL AND CONNECTIVE TISSUE DISORDERS NEC					
MUSCULOSKELETAL AND CONNECTIVE TISSUE CONDITIONS NEC					
MUSCULOSKELETAL STIFFNESS	0	0	0	0	0
BACK PAIN	2	2	0	0	0
LIMB DISCOMFORT	4	4	0	0	0
PAIN IN EXTREMITY	24	20	4	0	0
**NERVOUS SYSTEM DISORDERS**					
CRANIAL NERVE DISORDERS (EXCL NEOPLASMS)					
OLFACTORY NERVE DISORDERS					
ANOSMIA	0	0	0	0	0
PAROSMIA	1	0	1	0	0
HEADACHES					
HEADACHES NEC					
HEADACHE	40	37	3	0	0
SINUS HEADACHE	1	1	0	0	0
TENSION HEADACHE	4	4	0	0	0

MIGRAINE HEADACHES					
MIGRAINE	1	1	0	0	0
TYPICAL AURA WITHOUT HEADACHE	0	0	0	0	0
MOVEMENT DISORDERS (INCL PARKINSONISM)					
TREMOR (EXCL CONGENITAL)					
TREMOR	1	1	0	0	0
NEUROLOGICAL DISORDERS NEC					
DISTURBANCES IN CONSCIOUSNESS NEC					
LETHARGY	2	1	1	0	0
SOMNOLENCE	2	1	1	0	0
NEUROLOGICAL SIGNS AND SYMPTOMS NEC					
BRAIN FOG	1	1	0	0	0
DIZZINESS	4	3	1	0	0
INFANT IRRITABILITY	1	0	1	0	0
NEUROLOGICAL SYMPTOM	1	1	0	0	0
PRESYNCOPE	1	1	0	0	0
PARAESTHESIAS AND DYSAESTHESIAS					
HYPOAESTHESIA	0	0	0	0	0
PARAESTHESIA	1	1	0	0	0
SENSORY ABNORMALITIES NEC					
AGEUSIA	1	1	0	0	0
DYSGEUSIA	2	2	0	0	0
SEIZURES (INCL SUBTYPES)					
SEIZURES AND SEIZURE DISORDERS NEC					
SEIZURE	2	2	0	0	0
**PREGNANCY, PUERPERIUM AND PERINATAL CONDITIONS**					
PLACENTAL, AMNIOTIC AND CAVITY DISORDERS (EXCL HAEMORRHAGES)					
PLACENTAL ABNORMALITIES (EXCL NEOPLASMS)					
PLACENTAL INFARCTION	1	1	0	0	0
**PSYCHIATRIC DISORDERS**					
ANXIETY DISORDERS AND SYMPTOMS					
ANXIETY SYMPTOMS					
AGITATION	0	0	0	0	0
ANXIETY	0	0	0	0	0
DELIRIA (INCL CONFUSION)					
CONFUSION AND DISORIENTATION					
CONFUSIONAL STATE	1	1	0	0	0
DEPRESSED MOOD DISORDERS AND DISTURBANCES					
MOOD ALTERATIONS WITH DEPRESSIVE SYMPTOMS					
DEPRESSED MOOD	0	0	0	0	0
MOOD DISORDERS AND DISTURBANCES NEC					
EMOTIONAL AND MOOD DISTURBANCES NEC					
EMOTIONAL DISORDER	0	0	0	0	0
IRRITABILITY	1	1	0	0	0
MOOD ALTERED	0	0	0	0	0
SLEEP DISORDERS AND DISTURBANCES					
DISTURBANCES IN INITIATING AND MAINTAINING SLEEP					
INSOMNIA	2	2	0	0	0
**REPRODUCTIVE SYSTEM AND BREAST DISORDERS**					
MENSTRUAL CYCLE AND UTERINE BLEEDING DISORDERS					
MENSTRUATION AND UTERINE BLEEDING NEC					
MENSTRUATION IRREGULAR	0	0	0	0	0
MENSTRUATION WITH DECREASED BLEEDING					

HYPOMENORRHOEA	0	0	0	0	0
MENSTRUATION DELAYED	1	0	1	0	0
OLIGOMENORRHOEA	0	0	0	0	0
MENSTRUATION WITH INCREASED BLEEDING					
HEAVY MENSTRUAL BLEEDING	1	1	0	0	0
VULVOVAGINAL DISORDERS (EXCL INFECTIONS AND INFLAMMATIONS)					
VULVOVAGINAL DISORDERS NEC					
VAGINAL HAEMORRHAGE	0	0	0	0	0
**RESPIRATORY, THORACIC AND MEDIASTINAL DISORDERS**					
RESPIRATORY DISORDERS NEC					
BREATHING ABNORMALITIES					
DYSPNOEA	1	0	1	0	0
COUGHING AND ASSOCIATED SYMPTOMS					
COUGH	0	0	0	0	0
RESPIRATORY TRACT SIGNS AND SYMPTOMS					
UPPER RESPIRATORY TRACT SIGNS AND SYMPTOMS					
OROPHARYNGEAL PAIN	4	4	0	0	0
PARANASAL SINUS DISCOMFORT	1	1	0	0	0
RHINORRHOEA	0	0	0	0	0
**SKIN AND SUBCUTANEOUS TISSUE DISORDERS**					
EPIDERMAL AND DERMAL CONDITIONS					
DERMAL AND EPIDERMAL CONDITIONS NEC					
SENSITIVE SKIN	1	1	0	0	0
DERMATITIS AND ECZEMA					
DERMATITIS ALLERGIC	1	1	0	0	0
ERYTHEMAS					
ERYTHEMA	1	1	0	0	0
PRURITUS NEC					
PRURITUS	1	1	0	0	0
RASHES, ERUPTIONS AND EXANTHEMS NEC					
RASH	0	0	0	0	0
SKIN APPENDAGE CONDITIONS					
APOCRINE AND ECCRINE GLAND DISORDERS					
COLD SWEAT	1	0	1	0	0
HYPERHIDROSIS	3	3	0	0	0
NIGHT SWEATS	1	1	0	0	0
**VASCULAR DISORDERS**					
EMBOLISM AND THROMBOSIS					
NON-SITE SPECIFIC EMBOLISM AND THROMBOSIS					
EMBOLISM	0	0	0	0	0
VASCULAR DISORDERS NEC					
PERIPHERAL VASCULAR DISORDERS NEC					
HOT FLUSH	1	1	0	0	0
**TOTAL ADR EVENTS**	***353***	***315***	***38***	***0***	***0***

SUPPLEMENTARY TABLE 33. Moderna COVID-19 vaccine: ADR listing for events reported by breastfeeding females, in those reporting any vaccination dose

	Individuals with any vaccination dose: ADR Counts				
**MEDDRA REACTION TERM (SOC, *HLGT*, *HLT*, PT)**	All doses	1st dose	2nd dose	3rd dose	Other doses
(freetext)	0	0	0	0	0
**BLOOD AND LYMPHATIC SYSTEM DISORDERS**					
LYMPHATIC SYSTEM DISORDERS NEC					
LYMPH NODE PAIN	0	0	0	0	0
LYMPHADENOPATHY	1	0	1	0	0
**CARDIAC DISORDERS**					
CARDIAC DISORDERS, SIGNS AND SYMPTOMS NEC					
CARDIAC SIGNS AND SYMPTOMS NEC					
PALPITATIONS	0	0	0	0	0
**EAR AND LABYRINTH DISORDERS**					
AURAL DISORDERS NEC					
EAR DISORDERS NEC					
EAR PAIN	0	0	0	0	0
INNER EAR AND VIIITH CRANIAL NERVE DISORDERS					
INNER EAR SIGNS AND SYMPTOMS					
TINNITUS	1	0	0	1	0
**EYE DISORDERS**					
EYE DISORDERS NEC					
LACRIMATION DISORDERS					
DRY EYE	0	0	0	0	0
OCULAR DISORDERS NEC					
EYE PAIN	0	0	0	0	0
OCULAR INFECTIONS, IRRITATIONS AND INFLAMMATIONS					
OCULAR INFECTIONS, INFLAMMATIONS AND ASSOCIATED MANIFESTATIONS					
EYE IRRITATION	1	1	0	0	0
OCULAR SENSORY SYMPTOMS NEC					
OCULAR SENSATION DISORDERS					
PHOTOPHOBIA	0	0	0	0	0
VISION DISORDERS					
VISUAL DISORDERS NEC					
VISION BLURRED	0	0	0	0	0
**GASTROINTESTINAL DISORDERS**					
GASTROINTESTINAL INFLAMMATORY CONDITIONS					
GASTROINTESTINAL INFLAMMATORY DISORDERS NEC					
GASTROINTESTINAL TRACT IRRITATION	0	0	0	0	0
GASTROINTESTINAL MOTILITY AND DEFAECATION CONDITIONS					
DIARRHOEA (EXCL INFECTIVE)					
DIARRHOEA	0	0	0	0	0
GASTROINTESTINAL SIGNS AND SYMPTOMS					
FLATULENCE, BLOATING AND DISTENSION					
ABDOMINAL DISTENSION	0	0	0	0	0
FLATULENCE	0	0	0	0	0
GASTROINTESTINAL AND ABDOMINAL PAINS (EXCL ORAL AND THROAT)					
ABDOMINAL PAIN	0	0	0	0	0
ABDOMINAL PAIN UPPER	0	0	0	0	0

NAUSEA AND VOMITING SYMPTOMS					
NAUSEA	6	2	0	4	0
RETCHING	0	0	0	0	0
VOMITING	1	0	1	0	0
ORAL SOFT TISSUE CONDITIONS					
ORAL SOFT TISSUE SIGNS AND SYMPTOMS					
PARAESTHESIA ORAL	0	0	0	0	0
LIP SWELLING	1	0	1	0	0
**GENERAL DISORDERS AND ADMINISTRATION SITE CONDITIONS**					
ADMINISTRATION SITE REACTIONS					
ADMINISTRATION SITE REACTIONS NEC					
PUNCTURE SITE BRUISE	1	0	0	1	0
INJECTION SITE REACTIONS					
INJECTION SITE ERYTHEMA	0	0	0	0	0
INJECTION SITE MASS	0	0	0	0	0
INJECTION SITE PAIN	1	0	0	1	0
INJECTION SITE WARMTH	0	0	0	0	0
VACCINATION SITE REACTIONS					
VACCINATION SITE PAIN	0	0	0	0	0
BODY TEMPERATURE CONDITIONS					
FEBRILE DISORDERS					
PYREXIA	10	1	5	4	0
GENERAL SYSTEM DISORDERS NEC					
ASTHENIC CONDITIONS					
FATIGUE	20	5	8	7	0
MALAISE	2	1	1	0	0
FEELINGS AND SENSATIONS NEC					
CHILLS	8	0	3	5	0
FEELING ABNORMAL	0	0	0	0	0
FEELING COLD	1	0	1	0	0
FEELING HOT	0	0	0	0	0
HANGOVER	0	0	0	0	0
GENERAL SIGNS AND SYMPTOMS NEC					
INFLUENZA LIKE ILLNESS	2	0	0	2	0
LOCAL REACTION	0	0	0	0	0
PERIPHERAL SWELLING	1	1	0	0	0
SWELLING	1	1	0	0	0
PAIN AND DISCOMFORT NEC					
AXILLARY PAIN	0	0	0	0	0
CHEST DISCOMFORT	0	0	0	0	0
PAIN	2	0	1	1	0
TENDERNESS	0	0	0	0	0
**INFECTIONS AND INFESTATIONS**					
INFECTIONS - PATHOGEN UNSPECIFIED					
BREAST INFECTIONS					
MASTITIS	1	0	0	1	0
SKIN STRUCTURES AND SOFT TISSUE INFECTIONS					
INJECTION SITE PUSTULE	0	0	0	0	0
UPPER RESPIRATORY TRACT INFECTIONS					
LARYNGITIS	0	0	0	0	0
NASOPHARYNGITIS	0	0	0	0	0
VIRAL INFECTIOUS DISORDERS					
HERPES VIRAL INFECTIONS					

ORAL HERPES	0	0	0	0	0
INFLUENZA VIRAL INFECTIONS					
INFLUENZA	3	1	2	0	0
**INJURY, POISONING AND PROCEDURAL COMPLICATIONS**					
INJURIES NEC					
SKIN INJURIES NEC					
CONTUSION	0	0	0	0	0
**INVESTIGATIONS**					
CARDIAC AND VASCULAR INVESTIGATIONS (EXCL ENZYME TESTS)					
HEART RATE AND PULSE INVESTIGATIONS					
HEART RATE	0	0	0	0	0
PHYSICAL EXAMINATION AND ORGAN SYSTEM STATUS TOPICS					
PHYSICAL EXAMINATION PROCEDURES AND ORGAN SYSTEM STATUS					
BODY TEMPERATURE	0	0	0	0	0
BODY TEMPERATURE INCREASED	1	0	0	1	0
WATER, ELECTROLYTE AND MINERAL INVESTIGATIONS					
WATER AND ELECTROLYTE ANALYSES NEC					
VOLUME BLOOD	0	0	0	0	0
**METABOLISM AND NUTRITION DISORDERS**					
APPETITE AND GENERAL NUTRITIONAL DISORDERS					
APPETITE DISORDERS					
DECREASED APPETITE	0	0	0	0	0
**MUSCULOSKELETAL AND CONNECTIVE TISSUE DISORDERS**					
BONE DISORDERS (EXCL CONGENITAL AND FRACTURES)					
BONE RELATED SIGNS AND SYMPTOMS					
BONE PAIN	0	0	0	0	0
PAIN IN JAW	0	0	0	0	0
JOINT DISORDERS					
JOINT RELATED SIGNS AND SYMPTOMS					
ARTHRALGIA	3	0	2	1	0
JOINT STIFFNESS	0	0	0	0	0
MUSCLE DISORDERS					
MUSCLE PAINS					
MYALGIA	9	2	3	4	0
MUSCLE RELATED SIGNS AND SYMPTOMS NEC					
MUSCLE FATIGUE	1	0	0	1	0
MUSCLE SPASMS	0	0	0	0	0
MUSCULOSKELETAL AND CONNECTIVE TISSUE DISORDERS NEC					
MUSCULOSKELETAL AND CONNECTIVE TISSUE CONDITIONS NEC					
MUSCULOSKELETAL STIFFNESS	0	0	0	0	0
BACK PAIN	0	0	0	0	0
LIMB DISCOMFORT	2	1	1	0	0
PAIN IN EXTREMITY	18	11	5	2	0
**NERVOUS SYSTEM DISORDERS**					
CRANIAL NERVE DISORDERS (EXCL NEOPLASMS)					
OLFACTORY NERVE DISORDERS					
ANOSMIA	0	0	0	0	0
PAROSMIA	0	0	0	0	0
HEADACHES					
HEADACHES NEC					
HEADACHE	12	3	4	5	0
SINUS HEADACHE	0	0	0	0	0
TENSION HEADACHE	0	0	0	0	0

MIGRAINE HEADACHES					
MIGRAINE	0	0	0	0	0
TYPICAL AURA WITHOUT HEADACHE	0	0	0	0	0
MOVEMENT DISORDERS (INCL PARKINSONISM)					
TREMOR (EXCL CONGENITAL)					
TREMOR	0	0	0	0	0
NEUROLOGICAL DISORDERS NEC					
DISTURBANCES IN CONSCIOUSNESS NEC					
LETHARGY	0	0	0	0	0
SOMNOLENCE	1	0	1	0	0
NEUROLOGICAL SIGNS AND SYMPTOMS NEC					
BRAIN FOG	0	0	0	0	0
DIZZINESS	3	1	1	1	0
INFANT IRRITABILITY	0	0	0	0	0
NEUROLOGICAL SYMPTOM	0	0	0	0	0
PRESYNCOPE	0	0	0	0	0
PARAESTHESIAS AND DYSAESTHESIAS					
HYPOAESTHESIA	0	0	0	0	0
PARAESTHESIA	0	0	0	0	0
SENSORY ABNORMALITIES NEC					
AGEUSIA	0	0	0	0	0
DYSGEUSIA	0	0	0	0	0
SEIZURES (INCL SUBTYPES)					
SEIZURES AND SEIZURE DISORDERS NEC					
SEIZURE	0	0	0	0	0
**PREGNANCY, PUERPERIUM AND PERINATAL CONDITIONS**					
PLACENTAL, AMNIOTIC AND CAVITY DISORDERS (EXCL HAEMORRHAGES)					
PLACENTAL ABNORMALITIES (EXCL NEOPLASMS)					
PLACENTAL INFARCTION	0	0	0	0	0
**PSYCHIATRIC DISORDERS**					
ANXIETY DISORDERS AND SYMPTOMS					
ANXIETY SYMPTOMS					
AGITATION	0	0	0	0	0
ANXIETY	0	0	0	0	0
DELIRIA (INCL CONFUSION)					
CONFUSION AND DISORIENTATION					
CONFUSIONAL STATE	0	0	0	0	0
DEPRESSED MOOD DISORDERS AND DISTURBANCES					
MOOD ALTERATIONS WITH DEPRESSIVE SYMPTOMS					
DEPRESSED MOOD	0	0	0	0	0
MOOD DISORDERS AND DISTURBANCES NEC					
EMOTIONAL AND MOOD DISTURBANCES NEC					
EMOTIONAL DISORDER	0	0	0	0	0
IRRITABILITY	0	0	0	0	0
MOOD ALTERED	1	0	0	1	0
SLEEP DISORDERS AND DISTURBANCES					
DISTURBANCES IN INITIATING AND MAINTAINING SLEEP					
INSOMNIA	0	0	0	0	0
**REPRODUCTIVE SYSTEM AND BREAST DISORDERS**					
MENSTRUAL CYCLE AND UTERINE BLEEDING DISORDERS					
MENSTRUATION AND UTERINE BLEEDING NEC					
MENSTRUATION IRREGULAR	0	0	0	0	0
MENSTRUATION WITH DECREASED BLEEDING					

HYPOMENORRHOEA	0	0	0	0	0
MENSTRUATION DELAYED	0	0	0	0	0
OLIGOMENORRHOEA	0	0	0	0	0
MENSTRUATION WITH INCREASED BLEEDING					
HEAVY MENSTRUAL BLEEDING	0	0	0	0	0
VULVOVAGINAL DISORDERS (EXCL INFECTIONS AND INFLAMMATIONS)					
VULVOVAGINAL DISORDERS NEC					
VAGINAL HAEMORRHAGE	0	0	0	0	0
**RESPIRATORY, THORACIC AND MEDIASTINAL DISORDERS**					
RESPIRATORY DISORDERS NEC					
BREATHING ABNORMALITIES					
DYSPNOEA	1	1	0	0	0
COUGHING AND ASSOCIATED SYMPTOMS					
COUGH	0	0	0	0	0
RESPIRATORY TRACT SIGNS AND SYMPTOMS					
UPPER RESPIRATORY TRACT SIGNS AND SYMPTOMS					
OROPHARYNGEAL PAIN	0	0	0	0	0
PARANASAL SINUS DISCOMFORT	0	0	0	0	0
RHINORRHOEA	1	0	0	1	0
**SKIN AND SUBCUTANEOUS TISSUE DISORDERS**					
EPIDERMAL AND DERMAL CONDITIONS					
DERMAL AND EPIDERMAL CONDITIONS NEC					
SENSITIVE SKIN	0	0	0	0	0
DERMATITIS AND ECZEMA					
DERMATITIS ALLERGIC	0	0	0	0	0
ERYTHEMAS					
ERYTHEMA	0	0	0	0	0
PRURITUS NEC					
PRURITUS	2	2	0	0	0
RASHES, ERUPTIONS AND EXANTHEMS NEC					
RASH	2	1	0	1	0
SKIN APPENDAGE CONDITIONS					
APOCRINE AND ECCRINE GLAND DISORDERS					
COLD SWEAT	0	0	0	0	0
HYPERHIDROSIS	0	0	0	0	0
NIGHT SWEATS	0	0	0	0	0
**VASCULAR DISORDERS**					
EMBOLISM AND THROMBOSIS					
NON-SITE SPECIFIC EMBOLISM AND THROMBOSIS					
EMBOLISM	1	1	0	0	0
VASCULAR DISORDERS NEC					
PERIPHERAL VASCULAR DISORDERS NEC					
HOT FLUSH	0	0	0	0	0
**TOTAL ADR EVENTS**	***122***	***36***	***41***	***45***	***0***

SUPPLEMENTARY TABLE 34. Others/Unknown COVID-19 vaccine: ADR listing for events reported by breastfeeding females, in those reporting any vaccination dose

	Individuals with any vaccination dose: ADR Counts				
**MEDDRA REACTION TERM (SOC, *HLGT*, HLT, PT)**	All doses	1st dose	2nd dose	3rd dose	Other doses
(freetext)	0	0	0	0	0
**BLOOD AND LYMPHATIC SYSTEM DISORDERS**					
LYMPHATIC SYSTEM DISORDERS NEC					
LYMPH NODE PAIN	0	0	0	0	0
LYMPHADENOPATHY	0	0	0	0	0
**CARDIAC DISORDERS**					
CARDIAC DISORDERS, SIGNS AND SYMPTOMS NEC					
CARDIAC SIGNS AND SYMPTOMS NEC					
PALPITATIONS	0	0	0	0	0
**EAR AND LABYRINTH DISORDERS**					
AURAL DISORDERS NEC					
EAR DISORDERS NEC					
EAR PAIN	0	0	0	0	0
INNER EAR AND VIIITH CRANIAL NERVE DISORDERS					
INNER EAR SIGNS AND SYMPTOMS					
TINNITUS	0	0	0	0	0
**EYE DISORDERS**					
EYE DISORDERS NEC					
LACRIMATION DISORDERS					
DRY EYE	0	0	0	0	0
OCULAR DISORDERS NEC					
EYE PAIN	0	0	0	0	0
OCULAR INFECTIONS, IRRITATIONS AND INFLAMMATIONS					
OCULAR INFECTIONS, INFLAMMATIONS AND ASSOCIATED MANIFESTATIONS					
EYE IRRITATION	0	0	0	0	0
OCULAR SENSORY SYMPTOMS NEC					
OCULAR SENSATION DISORDERS					
PHOTOPHOBIA	0	0	0	0	0
VISION DISORDERS					
VISUAL DISORDERS NEC					
VISION BLURRED	0	0	0	0	0
**GASTROINTESTINAL DISORDERS**					
GASTROINTESTINAL INFLAMMATORY CONDITIONS					
GASTROINTESTINAL INFLAMMATORY DISORDERS NEC					
GASTROINTESTINAL TRACT IRRITATION	0	0	0	0	0
GASTROINTESTINAL MOTILITY AND DEFAECATION CONDITIONS					
DIARRHOEA (EXCL INFECTIVE)					
DIARRHOEA	0	0	0	0	0
GASTROINTESTINAL SIGNS AND SYMPTOMS					
FLATULENCE, BLOATING AND DISTENSION					
ABDOMINAL DISTENSION	0	0	0	0	0
FLATULENCE	0	0	0	0	0
GASTROINTESTINAL AND ABDOMINAL PAINS (EXCL ORAL AND THROAT)					
ABDOMINAL PAIN	0	0	0	0	0
ABDOMINAL PAIN UPPER	0	0	0	0	0

NAUSEA AND VOMITING SYMPTOMS					
NAUSEA	1	0	0	1	0
RETCHING	0	0	0	0	0
VOMITING	0	0	0	0	0
ORAL SOFT TISSUE CONDITIONS					
ORAL SOFT TISSUE SIGNS AND SYMPTOMS					
PARAESTHESIA ORAL	0	0	0	0	0
LIP SWELLING	0	0	0	0	0
**GENERAL DISORDERS AND ADMINISTRATION SITE CONDITIONS**					
ADMINISTRATION SITE REACTIONS					
ADMINISTRATION SITE REACTIONS NEC					
PUNCTURE SITE BRUISE	0	0	0	0	0
INJECTION SITE REACTIONS					
INJECTION SITE ERYTHEMA	0	0	0	0	0
INJECTION SITE MASS	0	0	0	0	0
INJECTION SITE PAIN	0	0	0	0	0
INJECTION SITE WARMTH	0	0	0	0	0
VACCINATION SITE REACTIONS					
VACCINATION SITE PAIN	0	0	0	0	0
BODY TEMPERATURE CONDITIONS					
FEBRILE DISORDERS					
PYREXIA	0	0	0	0	0
GENERAL SYSTEM DISORDERS NEC					
ASTHENIC CONDITIONS					
FATIGUE	0	0	0	0	0
MALAISE	0	0	0	0	0
FEELINGS AND SENSATIONS NEC					
CHILLS	0	0	0	0	0
FEELING ABNORMAL	0	0	0	0	0
FEELING COLD	0	0	0	0	0
FEELING HOT	0	0	0	0	0
HANGOVER	0	0	0	0	0
GENERAL SIGNS AND SYMPTOMS NEC					
INFLUENZA LIKE ILLNESS	0	0	0	0	0
LOCAL REACTION	0	0	0	0	0
PERIPHERAL SWELLING	0	0	0	0	0
SWELLING	0	0	0	0	0
PAIN AND DISCOMFORT NEC					
AXILLARY PAIN	0	0	0	0	0
CHEST DISCOMFORT	0	0	0	0	0
PAIN	1	0	0	1	0
TENDERNESS	0	0	0	0	0
**INFECTIONS AND INFESTATIONS**					
INFECTIONS - PATHOGEN UNSPECIFIED					
BREAST INFECTIONS					
MASTITIS	0	0	0	0	0
SKIN STRUCTURES AND SOFT TISSUE INFECTIONS					
INJECTION SITE PUSTULE	0	0	0	0	0
UPPER RESPIRATORY TRACT INFECTIONS					
LARYNGITIS	0	0	0	0	0
NASOPHARYNGITIS	0	0	0	0	0
VIRAL INFECTIOUS DISORDERS					
HERPES VIRAL INFECTIONS					

ORAL HERPES	0	0	0	0	0
INFLUENZA VIRAL INFECTIONS					
INFLUENZA	0	0	0	0	0
**INJURY, POISONING AND PROCEDURAL COMPLICATIONS**					
INJURIES NEC					
SKIN INJURIES NEC					
CONTUSION	0	0	0	0	0
**INVESTIGATIONS**					
CARDIAC AND VASCULAR INVESTIGATIONS (EXCL ENZYME TESTS)					
HEART RATE AND PULSE INVESTIGATIONS					
HEART RATE	0	0	0	0	0
PHYSICAL EXAMINATION AND ORGAN SYSTEM STATUS TOPICS					
PHYSICAL EXAMINATION PROCEDURES AND ORGAN SYSTEM STATUS					
BODY TEMPERATURE	0	0	0	0	0
BODY TEMPERATURE INCREASED	0	0	0	0	0
WATER, ELECTROLYTE AND MINERAL INVESTIGATIONS					
WATER AND ELECTROLYTE ANALYSES NEC					
VOLUME BLOOD	0	0	0	0	0
**METABOLISM AND NUTRITION DISORDERS**					
APPETITE AND GENERAL NUTRITIONAL DISORDERS					
APPETITE DISORDERS					
DECREASED APPETITE	0	0	0	0	0
**MUSCULOSKELETAL AND CONNECTIVE TISSUE DISORDERS**					
BONE DISORDERS (EXCL CONGENITAL AND FRACTURES)					
BONE RELATED SIGNS AND SYMPTOMS					
BONE PAIN	0	0	0	0	0
PAIN IN JAW	0	0	0	0	0
JOINT DISORDERS					
JOINT RELATED SIGNS AND SYMPTOMS					
ARTHRALGIA	0	0	0	0	0
JOINT STIFFNESS	0	0	0	0	0
MUSCLE DISORDERS					
MUSCLE PAINS					
MYALGIA	0	0	0	0	0
MUSCLE RELATED SIGNS AND SYMPTOMS NEC					
MUSCLE FATIGUE	0	0	0	0	0
MUSCLE SPASMS	0	0	0	0	0
MUSCULOSKELETAL AND CONNECTIVE TISSUE DISORDERS NEC					
MUSCULOSKELETAL AND CONNECTIVE TISSUE CONDITIONS NEC					
MUSCULOSKELETAL STIFFNESS	0	0	0	0	0
BACK PAIN	0	0	0	0	0
LIMB DISCOMFORT	0	0	0	0	0
PAIN IN EXTREMITY	0	0	0	0	0
**NERVOUS SYSTEM DISORDERS**					
CRANIAL NERVE DISORDERS (EXCL NEOPLASMS)					
OLFACTORY NERVE DISORDERS					
ANOSMIA	0	0	0	0	0
PAROSMIA	0	0	0	0	0
HEADACHES					
HEADACHES NEC					
HEADACHE	1	0	0	1	0
SINUS HEADACHE	0	0	0	0	0
TENSION HEADACHE	0	0	0	0	0

MIGRAINE HEADACHES					
MIGRAINE	0	0	0	0	0
TYPICAL AURA WITHOUT HEADACHE	0	0	0	0	0
MOVEMENT DISORDERS (INCL PARKINSONISM)					
TREMOR (EXCL CONGENITAL)					
TREMOR	0	0	0	0	0
NEUROLOGICAL DISORDERS NEC					
DISTURBANCES IN CONSCIOUSNESS NEC					
LETHARGY	0	0	0	0	0
SOMNOLENCE	0	0	0	0	0
NEUROLOGICAL SIGNS AND SYMPTOMS NEC					
BRAIN FOG	0	0	0	0	0
DIZZINESS	0	0	0	0	0
INFANT IRRITABILITY	0	0	0	0	0
NEUROLOGICAL SYMPTOM	0	0	0	0	0
PRESYNCOPE	0	0	0	0	0
PARAESTHESIAS AND DYSAESTHESIAS					
HYPOAESTHESIA	0	0	0	0	0
PARAESTHESIA	0	0	0	0	0
SENSORY ABNORMALITIES NEC					
AGEUSIA	0	0	0	0	0
DYSGEUSIA	0	0	0	0	0
SEIZURES (INCL SUBTYPES)					
SEIZURES AND SEIZURE DISORDERS NEC					
SEIZURE	0	0	0	0	0
**PREGNANCY, PUERPERIUM AND PERINATAL CONDITIONS**					
PLACENTAL, AMNIOTIC AND CAVITY DISORDERS (EXCL HAEMORRHAGES)					
PLACENTAL ABNORMALITIES (EXCL NEOPLASMS)					
PLACENTAL INFARCTION	0	0	0	0	0
**PSYCHIATRIC DISORDERS**					
ANXIETY DISORDERS AND SYMPTOMS					
ANXIETY SYMPTOMS					
AGITATION	0	0	0	0	0
ANXIETY	0	0	0	0	0
DELIRIA (INCL CONFUSION)					
CONFUSION AND DISORIENTATION					
CONFUSIONAL STATE	0	0	0	0	0
DEPRESSED MOOD DISORDERS AND DISTURBANCES					
MOOD ALTERATIONS WITH DEPRESSIVE SYMPTOMS					
DEPRESSED MOOD	0	0	0	0	0
MOOD DISORDERS AND DISTURBANCES NEC					
EMOTIONAL AND MOOD DISTURBANCES NEC					
EMOTIONAL DISORDER	0	0	0	0	0
IRRITABILITY	0	0	0	0	0
MOOD ALTERED	0	0	0	0	0
SLEEP DISORDERS AND DISTURBANCES					
DISTURBANCES IN INITIATING AND MAINTAINING SLEEP					
INSOMNIA	0	0	0	0	0
**REPRODUCTIVE SYSTEM AND BREAST DISORDERS**					
MENSTRUAL CYCLE AND UTERINE BLEEDING DISORDERS					
MENSTRUATION AND UTERINE BLEEDING NEC					
MENSTRUATION IRREGULAR	0	0	0	0	0
MENSTRUATION WITH DECREASED BLEEDING					

HYPOMENORRHOEA	0	0	0	0	0
MENSTRUATION DELAYED	0	0	0	0	0
OLIGOMENORRHOEA	0	0	0	0	0
MENSTRUATION WITH INCREASED BLEEDING					
HEAVY MENSTRUAL BLEEDING	0	0	0	0	0
VULVOVAGINAL DISORDERS (EXCL INFECTIONS AND INFLAMMATIONS)					
VULVOVAGINAL DISORDERS NEC					
VAGINAL HAEMORRHAGE	0	0	0	0	0
**RESPIRATORY, THORACIC AND MEDIASTINAL DISORDERS**					
RESPIRATORY DISORDERS NEC					
BREATHING ABNORMALITIES					
DYSPNOEA	0	0	0	0	0
COUGHING AND ASSOCIATED SYMPTOMS					
COUGH	0	0	0	0	0
RESPIRATORY TRACT SIGNS AND SYMPTOMS					
UPPER RESPIRATORY TRACT SIGNS AND SYMPTOMS					
OROPHARYNGEAL PAIN	0	0	0	0	0
PARANASAL SINUS DISCOMFORT	0	0	0	0	0
RHINORRHOEA	0	0	0	0	0
**SKIN AND SUBCUTANEOUS TISSUE DISORDERS**					
EPIDERMAL AND DERMAL CONDITIONS					
DERMAL AND EPIDERMAL CONDITIONS NEC					
SENSITIVE SKIN	0	0	0	0	0
DERMATITIS AND ECZEMA					
DERMATITIS ALLERGIC	0	0	0	0	0
ERYTHEMAS					
ERYTHEMA	0	0	0	0	0
PRURITUS NEC					
PRURITUS	0	0	0	0	0
RASHES, ERUPTIONS AND EXANTHEMS NEC					
RASH	0	0	0	0	0
SKIN APPENDAGE CONDITIONS					
APOCRINE AND ECCRINE GLAND DISORDERS					
COLD SWEAT	0	0	0	0	0
HYPERHIDROSIS	0	0	0	0	0
NIGHT SWEATS	0	0	0	0	0
**VASCULAR DISORDERS**					
EMBOLISM AND THROMBOSIS					
NON-SITE SPECIFIC EMBOLISM AND THROMBOSIS					
EMBOLISM	0	0	0	0	0
VASCULAR DISORDERS NEC					
PERIPHERAL VASCULAR DISORDERS NEC					
HOT FLUSH	0	0	0	0	0
**TOTAL ADR EVENTS**	***3***	***0***	***0***	***3***	***0***

SUPPLEMENTARY TABLE 35. COVID-19 vaccine ADR listings for events reported by breastfeeding females with no dose identification information in those reporting any vaccination dose

	Individuals with any vaccination dose: ADR Counts
**MEDDRA REACTION TERM (SOC, *HLGT*, *HLT*, PT)**	Unknown dose
(freetext)	
**BLOOD AND LYMPHATIC SYSTEM DISORDERS**	
LYMPHATIC SYSTEM DISORDERS NEC	
LYMPH NODE PAIN	0
LYMPHADENOPATHY	1
**CARDIAC DISORDERS**	
CARDIAC DISORDERS, SIGNS AND SYMPTOMS NEC	
CARDIAC SIGNS AND SYMPTOMS NEC	
PALPITATIONS	2
**EAR AND LABYRINTH DISORDERS**	
AURAL DISORDERS NEC	
EAR DISORDERS NEC	
EAR PAIN	0
INNER EAR AND VIIITH CRANIAL NERVE DISORDERS	
INNER EAR SIGNS AND SYMPTOMS	
TINNITUS	0
**EYE DISORDERS**	
EYE DISORDERS NEC	
LACRIMATION DISORDERS	
DRY EYE	0
OCULAR DISORDERS NEC	
EYE PAIN	0
OCULAR INFECTIONS, IRRITATIONS AND INFLAMMATIONS	
OCULAR INFECTIONS, INFLAMMATIONS AND ASSOCIATED MANIFESTATIONS	
EYE IRRITATION	0
OCULAR SENSORY SYMPTOMS NEC	
OCULAR SENSATION DISORDERS	
PHOTOPHOBIA	0
VISION DISORDERS	
VISUAL DISORDERS NEC	
VISION BLURRED	0
**GASTROINTESTINAL DISORDERS**	
GASTROINTESTINAL INFLAMMATORY CONDITIONS	
GASTROINTESTINAL INFLAMMATORY DISORDERS NEC	
GASTROINTESTINAL TRACT IRRITATION	0
GASTROINTESTINAL MOTILITY AND DEFAECATION CONDITIONS	
DIARRHOEA (EXCL INFECTIVE)	
DIARRHOEA	0
GASTROINTESTINAL SIGNS AND SYMPTOMS	
FLATULENCE, BLOATING AND DISTENSION	
ABDOMINAL DISTENSION	0
FLATULENCE	0
GASTROINTESTINAL AND ABDOMINAL PAINS (EXCL ORAL AND THROAT)	
ABDOMINAL PAIN	0

ABDOMINAL PAIN UPPER	0
NAUSEA AND VOMITING SYMPTOMS	
NAUSEA	4
RETCHING	0
VOMITING	0
ORAL SOFT TISSUE CONDITIONS	
ORAL SOFT TISSUE SIGNS AND SYMPTOMS	
PARAESTHESIA ORAL	0
LIP SWELLING	0
**GENERAL DISORDERS AND ADMINISTRATION SITE CONDITIONS**	
ADMINISTRATION SITE REACTIONS	
ADMINISTRATION SITE REACTIONS NEC	
PUNCTURE SITE BRUISE	0
INJECTION SITE REACTIONS	
INJECTION SITE ERYTHEMA	0
INJECTION SITE MASS	1
INJECTION SITE PAIN	3
INJECTION SITE WARMTH	0
VACCINATION SITE REACTIONS	
VACCINATION SITE PAIN	0
BODY TEMPERATURE CONDITIONS	
FEBRILE DISORDERS	
PYREXIA	5
GENERAL SYSTEM DISORDERS NEC	
ASTHENIC CONDITIONS	
FATIGUE	8
MALAISE	0
FEELINGS AND SENSATIONS NEC	
CHILLS	3
FEELING ABNORMAL	0
FEELING COLD	0
FEELING HOT	0
HANGOVER	0
GENERAL SIGNS AND SYMPTOMS NEC	
INFLUENZA LIKE ILLNESS	1
LOCAL REACTION	0
PERIPHERAL SWELLING	0
SWELLING	0
PAIN AND DISCOMFORT NEC	
AXILLARY PAIN	0
CHEST DISCOMFORT	0
PAIN	2
TENDERNESS	0
**INFECTIONS AND INFESTATIONS**	
INFECTIONS - PATHOGEN UNSPECIFIED	
BREAST INFECTIONS	
MASTITIS	0
SKIN STRUCTURES AND SOFT TISSUE INFECTIONS	
INJECTION SITE PUSTULE	0
UPPER RESPIRATORY TRACT INFECTIONS	
LARYNGITIS	0
NASOPHARYNGITIS	0
VIRAL INFECTIOUS DISORDERS	
HERPES VIRAL INFECTIONS	
ORAL HERPES	0
INFLUENZA VIRAL INFECTIONS	

INFLUENZA	0
**INJURY, POISONING AND PROCEDURAL COMPLICATIONS**	
INJURIES NEC	
SKIN INJURIES NEC	
CONTUSION	0
**INVESTIGATIONS**	
CARDIAC AND VASCULAR INVESTIGATIONS (EXCL ENZYME TESTS)	
HEART RATE AND PULSE INVESTIGATIONS	
HEART RATE	0
PHYSICAL EXAMINATION AND ORGAN SYSTEM STATUS TOPICS	
PHYSICAL EXAMINATION PROCEDURES AND ORGAN SYSTEM STATUS	
BODY TEMPERATURE	0
BODY TEMPERATURE INCREASED	0
WATER, ELECTROLYTE AND MINERAL INVESTIGATIONS	
WATER AND ELECTROLYTE ANALYSES NEC	
VOLUME BLOOD	0
**METABOLISM AND NUTRITION DISORDERS**	
APPETITE AND GENERAL NUTRITIONAL DISORDERS	
APPETITE DISORDERS	
DECREASED APPETITE	0
**MUSCULOSKELETAL AND CONNECTIVE TISSUE DISORDERS**	
BONE DISORDERS (EXCL CONGENITAL AND FRACTURES)	
BONE RELATED SIGNS AND SYMPTOMS	
BONE PAIN	0
PAIN IN JAW	0
JOINT DISORDERS	
JOINT RELATED SIGNS AND SYMPTOMS	
ARTHRALGIA	2
JOINT STIFFNESS	0
MUSCLE DISORDERS	
MUSCLE PAINS	
MYALGIA	2
MUSCLE RELATED SIGNS AND SYMPTOMS NEC	
MUSCLE FATIGUE	0
MUSCLE SPASMS	0
MUSCULOSKELETAL AND CONNECTIVE TISSUE DISORDERS NEC	
MUSCULOSKELETAL AND CONNECTIVE TISSUE CONDITIONS NEC	
MUSCULOSKELETAL STIFFNESS	0
BACK PAIN	0
LIMB DISCOMFORT	0
PAIN IN EXTREMITY	3
**NERVOUS SYSTEM DISORDERS**	
CRANIAL NERVE DISORDERS (EXCL NEOPLASMS)	
OLFACTORY NERVE DISORDERS	
ANOSMIA	0
PAROSMIA	0
HEADACHES	
HEADACHES NEC	
HEADACHE	8
SINUS HEADACHE	0
TENSION HEADACHE	0
MIGRAINE HEADACHES	
MIGRAINE	1
TYPICAL AURA WITHOUT HEADACHE	0
MOVEMENT DISORDERS (INCL PARKINSONISM)	
TREMOR (EXCL CONGENITAL)	

TREMOR	1
NEUROLOGICAL DISORDERS NEC	
DISTURBANCES IN CONSCIOUSNESS NEC	
LETHARGY	0
SOMNOLENCE	0
NEUROLOGICAL SIGNS AND SYMPTOMS NEC	
BRAIN FOG	0
DIZZINESS	3
INFANT IRRITABILITY	0
NEUROLOGICAL SYMPTOM	0
PRESYNCOPE	0
PARAESTHESIAS AND DYSAESTHESIAS	
HYPOAESTHESIA	0
PARAESTHESIA	0
SENSORY ABNORMALITIES NEC	
AGEUSIA	0
DYSGEUSIA	0
SEIZURES (INCL SUBTYPES)	
SEIZURES AND SEIZURE DISORDERS NEC	
SEIZURE	0
**PREGNANCY, PUERPERIUM AND PERINATAL CONDITIONS**	
PLACENTAL, AMNIOTIC AND CAVITY DISORDERS (EXCL HAEMORRHAGES)	
PLACENTAL ABNORMALITIES (EXCL NEOPLASMS)	
PLACENTAL INFARCTION	0
**PSYCHIATRIC DISORDERS**	
ANXIETY DISORDERS AND SYMPTOMS	
ANXIETY SYMPTOMS	
AGITATION	0
ANXIETY	2
DELIRIA (INCL CONFUSION)	
CONFUSION AND DISORIENTATION	
CONFUSIONAL STATE	0
DEPRESSED MOOD DISORDERS AND DISTURBANCES	
MOOD ALTERATIONS WITH DEPRESSIVE SYMPTOMS	
DEPRESSED MOOD	0
MOOD DISORDERS AND DISTURBANCES NEC	
EMOTIONAL AND MOOD DISTURBANCES NEC	
EMOTIONAL DISORDER	0
IRRITABILITY	0
MOOD ALTERED	0
SLEEP DISORDERS AND DISTURBANCES	
DISTURBANCES IN INITIATING AND MAINTAINING SLEEP	
INSOMNIA	0
**REPRODUCTIVE SYSTEM AND BREAST DISORDERS**	
MENSTRUAL CYCLE AND UTERINE BLEEDING DISORDERS	
MENSTRUATION AND UTERINE BLEEDING NEC	
MENSTRUATION IRREGULAR	0
MENSTRUATION WITH DECREASED BLEEDING	
HYPOMENORRHOEA	0
MENSTRUATION DELAYED	0
OLIGOMENORRHOEA	1
MENSTRUATION WITH INCREASED BLEEDING	
HEAVY MENSTRUAL BLEEDING	0
VULVOVAGINAL DISORDERS (EXCL INFECTIONS AND INFLAMMATIONS)	
VULVOVAGINAL DISORDERS NEC	
VAGINAL HAEMORRHAGE	0

**RESPIRATORY, THORACIC AND MEDIASTINAL DISORDERS**	
RESPIRATORY DISORDERS NEC	
BREATHING ABNORMALITIES	
DYSPNOEA	0
COUGHING AND ASSOCIATED SYMPTOMS	
COUGH	0
RESPIRATORY TRACT SIGNS AND SYMPTOMS	
UPPER RESPIRATORY TRACT SIGNS AND SYMPTOMS	
OROPHARYNGEAL PAIN	0
PARANASAL SINUS DISCOMFORT	0
RHINORRHOEA	0
**SKIN AND SUBCUTANEOUS TISSUE DISORDERS**	
EPIDERMAL AND DERMAL CONDITIONS	
DERMAL AND EPIDERMAL CONDITIONS NEC	
SENSITIVE SKIN	0
DERMATITIS AND ECZEMA	
DERMATITIS ALLERGIC	0
ERYTHEMAS	
ERYTHEMA	2
PRURITUS NEC	
PRURITUS	0
RASHES, ERUPTIONS AND EXANTHEMS NEC	
RASH	0
SKIN APPENDAGE CONDITIONS	
APOCRINE AND ECCRINE GLAND DISORDERS	
COLD SWEAT	0
HYPERHIDROSIS	1
NIGHT SWEATS	0
**VASCULAR DISORDERS**	
EMBOLISM AND THROMBOSIS	
NON-SITE SPECIFIC EMBOLISM AND THROMBOSIS	
EMBOLISM	0
VASCULAR DISORDERS NEC	
PERIPHERAL VASCULAR DISORDERS NEC	
HOT FLUSH	0
**TOTAL ADR EVENTS**	***56***
